# Supplementary material for: Contraceptive use and needs among adolescent women aged 15–19: Regional and global estimates and projections from 1990 to 2030 from a Bayesian hierarchical modelling study
Source: PLoS One. 2021 Mar 4;16(3):e0247479. doi: 10.1371/journal.pone.0247479 (PMC7932081; doi:10.1371/journal.pone.0247479)
Supplement: S1 Checklist — (DOCX) [file pone.0247479.s001.docx]

**S1 GATHER Checklist**

Contraceptive Use and Needs among Adolescent Women Aged 15-19:
Regional and Global Estimates and Projections from 1990 to 2030 from Bayesian Hierarchical Modelling Study

| **Item number** | **Checklist item** |
| --- | --- |
| **Objectives and funding** | |
| 1 | Define the indicator(s), populations (including age, sex, and geographic entities), and time period(s) for which estimates were made.  The indicators, population, and time period for which estimates were made are defined in Subsection ‘Definitions’ of Section ‘Methods’. |
| 2 | List the funding sources for the work.  Listed with the article online. |
| **Data inputs** | |
| *For all data inputs from multiple sources that are synthesised as part of the study:* | |
| 3 | Describe how the data were identified and how the data were accessed.  Described in Subsection ‘Data’ of Section ‘Data and methods’. |
| 4 | Specify the inclusion and exclusion criteria. Identify all ad-hoc exclusions.  Described in Subsection ‘Data’ of Section ‘Data and methods’. |
| 5 | Provide information about all included data sources and their main characteristics. For each data source used, report reference information or contact name/institution, population represented, data collection method, year(s) of data collection, sex and age range, diagnostic criteria or measurement method, and sample size, as relevant.  A narrative description of the data sources is given in Subsection ‘Data’ of Section ‘Data and methods’.  The input data themselves are included in S1 Data and S2 Data. Meta-data are contained the online open-access publication: United Nations, Department of Economic and Social Affairs, Population Division. World Contraceptive Use 2019. New York: 2019. https://www.un.org/en/development/desa/population/publications/dataset/contraception/wcu2019.asp |
| 6 | Identify and describe any categories of input data that have potentially important biases (eg, based on characteristics listed in item 5).  Biases and misclassifications in the input data are discussed in Subsection ‘Accounting for bias due to sampling of non-baseline groups and misclassifications’ of Section ‘Statistical Methods’ and in previous work referenced therein. |
| *For data inputs that contribute to the analysis but were not synthesised as part of the study:* | |
| 7 | Describe and give sources for any other data inputs.  These are data on the national population sizes of women of reproductive age and the proportion of women married/in-union. These are cited in Subsection ‘Data’ of Section ‘Data and methods’. |
| *For all data inputs:* | |
| 8 | Provide all data inputs in a file format from which data can be efficiently extracted (eg, a spreadsheet rather than a PDF), including all relevant meta-data listed in item 5. For any data inputs that cannot be shared because of ethical or legal reasons, such as third-party ownership, provide a contact name or the name of the institution that retains the right to the data.  Data inputs are provided as spreadsheets in supplementary materials S1 Data and S2 Data. |
| **Data analysis** | |
| 9 | Provide a conceptual overview of the data analysis method. A diagram may be helpful.  An overview is provided in the first paragraph of Subsection ‘Modelling trends in contraceptive use and unmet need for family planning’ of Section ‘Statistical Methods’. |
| 10 | Provide a detailed description of all steps of the analysis, including mathematical formulae. This description should cover, as relevant, data cleaning, data pre-processing, data adjustments and weighting of data sources, and mathematical or statistical model(s).  A detailed description is given Section ‘Statistical Methods’ and earlier work referenced therein. |
| 11 | Describe how candidate models were evaluated and how the final model(s) were selected.  Model development is described in Section ‘Statistical Methods’ and earlier work referenced therein . |
| 12 | Provide the results of an evaluation of model performance, if done, as well as the results of any relevant sensitivity analysis.  These can be provided upon request. |
| 13 | Describe methods of calculating uncertainty of the estimates. State which sources of uncertainty were, and were not, accounted for in the uncertainty analysis.  A detailed description is given Section ‘Statistical Methods’ and earlier work referenced therein. |
| 14 | State how analytical or statistical source code used to generate estimates can be accessed.  Noted in Subsection ‘Parameter Estimation and Software’ of Section ‘Statistical Methods’ in the main article. |
| **Results and discussion** | |
| 15 | Provide published estimates in a file format from which data can be efficiently extracted.  The published estimates are provided as supplementary materials in spreadsheet format. |
| 16 | Report a quantitative measure of the uncertainty of the estimates (eg, uncertainty intervals).  We report 95 percent (Bayesian) uncertainty intervals throughout the article. |
| 17 | Interpret results in light of existing evidence. If updating a previous set of estimates, describe the reasons for changes in estimates.  Provided in the ‘Discussion’ section of the main article. |
| 18 | Discuss limitations of the estimates. Include a discussion of any modelling assumptions or data limitations that affect interpretation of the estimates.  Provided in the ‘Discussion’ section of the main article. |
