# Supplementary material for: Contraceptive use and needs among adolescent women aged 15–19: Regional and global estimates and projections from 1990 to 2030 from a Bayesian hierarchical modelling study
Source: PLoS One. 2021 Mar 4;16(3):e0247479. doi: 10.1371/journal.pone.0247479 (PMC7932081; doi:10.1371/journal.pone.0247479)
Supplement: S1 Appendix — (PDF) [file pone.0247479.s002.pdf]

Contraceptive Use and Needs among Adolescent Women Aged 15-19: Regional and Global Estimates and Projections from 1990 to 2030 from a Bayesian Hierarchical Modeling Study

---

S1 APPENDIX

Vladimíra Kantorová*[1], Mark C. Wheldon[1], Aisha N. Z. Dasgupta[1], Philipp Ueffing[1], and Helena Cruz Castanheira[2]

[1]United Nations, Department of Economic and Social Affairs, Population Division, New York, NY, United States of America[†]
[2]United Nations, Economic Commission for Latin America and the Caribbean, Santiago de Chile, Chile[†]

December 14, 2020

*Corresponding author. Vladimíra Kantorová, Population Division, Department of Economic and Social Affairs, United Nations, kantorova@un.org

[†]The views and opinions expressed in this paper are those of the authors and do not necessarily represent those of the United Nations. This paper has not been formally edited and cleared by the United Nations.

Contents

1 Data and Country Classifications **1**
1.1 Data on Contraceptive Prevalence and Unmet Need for Family Planning 1
1.2 Classification of Countries Based on Sexual Activity 1

2 Statistical Methods **4**
2.1 Hierarchical Model Structures 4
2.2 Model Validation 4

3 Results **7**
3.1 Model Validation 7
3.2 Estimates and Projections for Level 2 Regions 10
3.3 Ternary Color Maps with Re-centered Color Scales 10

Appendix **15**

List of Tables

A Hierarchical structures used in the married and unmarried women models. 4
B Summary of model validation results. 8
C Country and region classifications used in model hierarchies. 15

List of Figures

A Data availability by region, time-period and marital status. 2
B Classification of countries by sexual activity among unmarried women. 3
C Hierarchical structures used in the married and unmarried women models. 5
D Estimates and projections of family planning indicators by Level 2 regions. 11
E Choropleth map with centered ternary color scheme, unmarried women aged 15–19, 2019. . . 13
F Choropleth map with centered ternary color scheme, all women aged 15–19, 2019. 14

This appendix contains details on data and methods, and additional results, that supplement those given in the main manuscript.

1 Data and Country Classifications

The term "country" as used in this study also refers, as appropriate, to territories or areas.

1.1 Data on Contraceptive Prevalence and Unmet Need for Family Planning

Data availability by region, time-period, and marital status is summarized in Figure A.

1.2 Classification of Countries Based on Sexual Activity

There are large differences across countries in the prevalence of sex among unmarried and not in-union women of reproductive age (UWRA) (Ueffing et al., 2019). We accounted for this by incorporating a country classification based on sexual activity in the hierarchical structure of our model for unmarried and not in-union women (see Section 2.1). The classification consisted of two groups defined as follows:

Sexual Activity Group 0 (SA0) Countries with very low levels of sexual activity, i.e., recent sexual activity (sexual intercourse in past four weeks) among UWRA was less than 2 percent;

Sexual Activity Group 1 (SA1) All other countries.

Countries were assigned to the same sexual activity group for the entire time period of estimation and projection.

Data on sexual activity among UWRA were available in 81 Demographic and Health Surveys (DHS) and Multiple Indicator Cluster Surveys (MICS). Countries with less than 2 percent sexually active (defined as having sexual intercourse in past 28 days) among UWRA were assigned to Group 0, the rest to Group 1. Other data were used as proxies for countries with neither a DHS nor a MICS. For 43 countries, we used information on the acceptance of sex between unmarried adults from Pew Research Center (2014) (29 countries) and Inglehart et al. (2013) (14 countries). We used the most recently available data for each county. The remaining 71 countries, all in Asia and Northern Africa, were assigned on the basis of the proportion religious in the population using information from Pew Research Center (2012).

In total, 45 countries (23 percent) were classified as having low sexual activity among unmarried women. All of these countries were in either Africa or Asia, predominantly in the following subregions: Northern Africa, Western Asia, South-Central Asia and South-Eastern Asia (Figure B). A list of all countries by sexual activity group is in Table C. A complete discussion of the classification scheme, and the data sources used, can be found in Kantorová et al. (2020, S1 Appendix, Subsection 2.4).

This classification was done using information about sexual activity among UWRA, that is aged 15–49 years. We compared it to a classification based on sexual activity among women aged 15–19 years for the 81 countries with data from DHS or MICS. A small number of countries switched groups but these were countries with enough data on the family planning indicators such that their sexual activity grouping would have minimal impact on the posterior estimates. Given this finding, and the fact that we did not have information for the remaining 114 countries, we retained the classification based on sexual activity among UWRA.

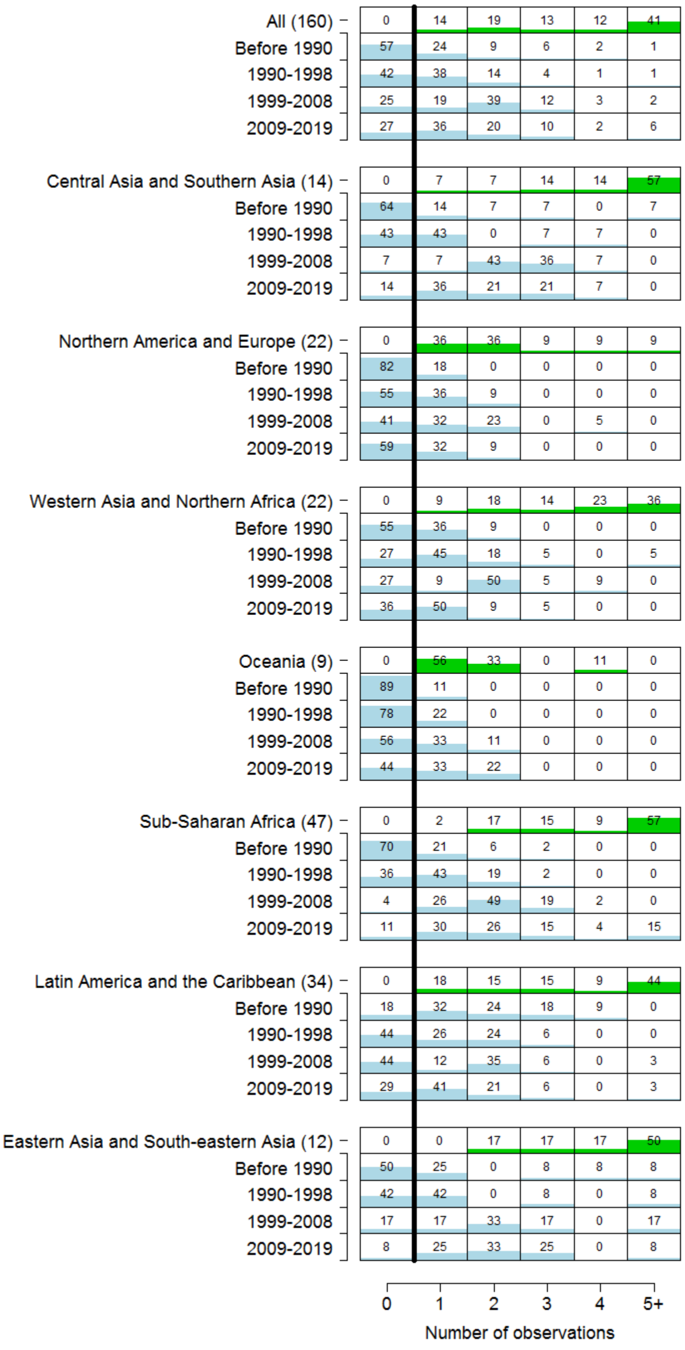

(i) Married women

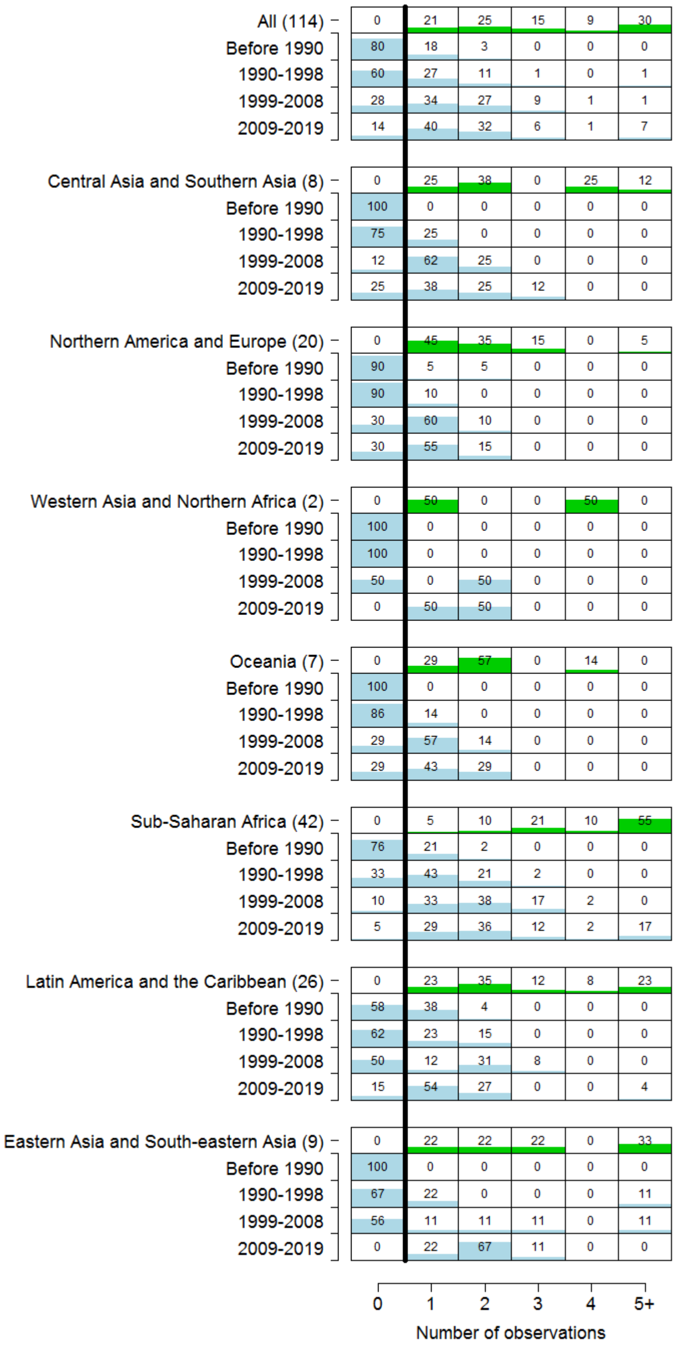

(ii) Unmarried women

Figure A. Data availability by region, time-period and marital status. Proportion of countries by number of observations of contraceptive prevalence (any method) by region and time period for (i) married and (ii) unmarried women aged 15–19. The cells give the percentage of all countries for the given region and time period with the number of observations according to column. The cells sum to 100 (within rounding) across each row. The numbers in parentheses next to the region names are the number of countries in that region with at least one observation.

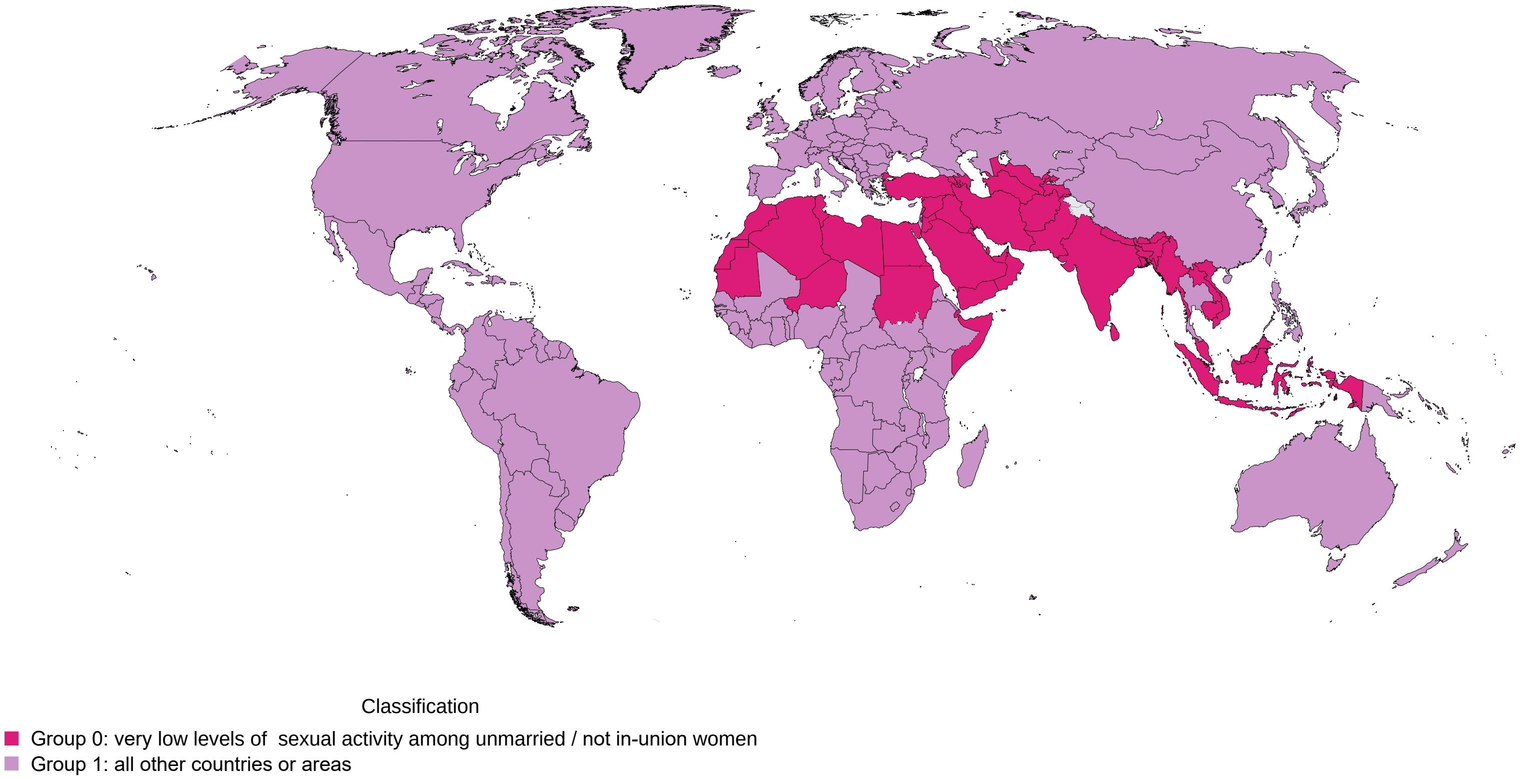

Figure B. Classification of countries by sexual activity among unmarried women. Sexual Activity Group 0, countries with low levels of sexual activity among unmarried women; Sexual Activity Group 1, countries with higher levels of sexual activity among unmarried women. Classification was based on the information about the level of, acceptance of, or justification for sexual activity among unmarried women from a variety of data sources, including DHS and MICS. The boundaries and names shown and the designations used on this map do not imply official endorsement or acceptance by the United Nations.

2 Statistical Methods

2.1 Hierarchical Model Structures

We used the same hierarchical structures as (Kantorová et al., 2020). Different structures were used to model married and unmarried women, and each marital group's structure had two variants, giving a total of four structures. The married women model used a geographic structure for the majority of model parameters and a combination of development status and geography for the remainder. The unmarried women model used a combination of the sexual activity classification (Section 1.2) and geography for both variants.

Each marital group's main variant had four levels, with the highest, Level 4, being "World", the cluster to which all countries belonged. The secondary variant in both models had no Level 4; this variant allowed a greater degree of divergence between country parameter estimates depending on the grouping at lower levels. The structure used in the unmarried women model was motivated by the *a priori* expectation that variation in contraceptive use among unmarried women would be related to variation in sexual activity. In the married women model, being married was taken as a reasonable proxy for being sexually active.

The four hierarchical structures thus defined are summarized in Table A and Figure C. For extended discussions of the structures and model parameters of the married women model see Alkema et al. (2013, Supplementary appendix, Subsections 2.1–2.2) and Cahill et al. (2017, Supplementary appendix, Subsections 2.1–2.3); for the unmarried women model see Kantorová et al. (2020, S1 Appendix, Subsection 3.4). For a list of countries by region, subregion, and sexual activity group, see Table C.

While it might be possible to explore different hierarchical structures, out-of-sample validation exercises (see Sections 2.2 and 3.1) indicated that the structure defined here performed well in terms of predictive accuracy. Therefore, we would expect any improvements under other structures to be limited.

Table A. Comparison of the hierarchical structures used in the married (i & ii) and unmarried (iii & iv) models for estimating contraceptive prevalence among women aged 15–19. The term "country" as used in this study also refers, as appropriate, to territories or areas. The terms "developed" and "less developed" are used for statistical convenience and do not necessarily express a judgment about the stage reached by a particular country or area in the development process. See also Figure C.

	Classification Scheme			
	(i) Geographic	(ii) Development / Geographic	(iii) Sexual Activity / Geographic	(iv) Sexual Activity / Geographic
Level 1	Country	Country	Country	Country
Level 2	Subregion	Region / —*	Region + India / Subregion†	Region‡
Level 3	Region	Development status	Sexual activity group	Sexual activity group
Level 4	World	—	World	—

* Only for Less Developed Countries; no Level 2 for Developed Countries.
† Region + India for Sexual Activity Group 0; Subregion for Sexual Activity Group 1.
‡ No Level 2 for Sexual Activity Group 0; Region for Sexual Activity Group 1.

2.2 Model Validation

Model performance was assessed using cross-validation exercises like those employed by Kantorová et al. (2020, S1 Appendix, Sect. 4.3) and Alkema et al. (2013, Supplementary Appendix, Sect. 2.6). In each exercise the input data set was partitioned into a *training set* and a *test set*. The model was then re-run on the training set and the results evaluated against the test set. The exercises used were:

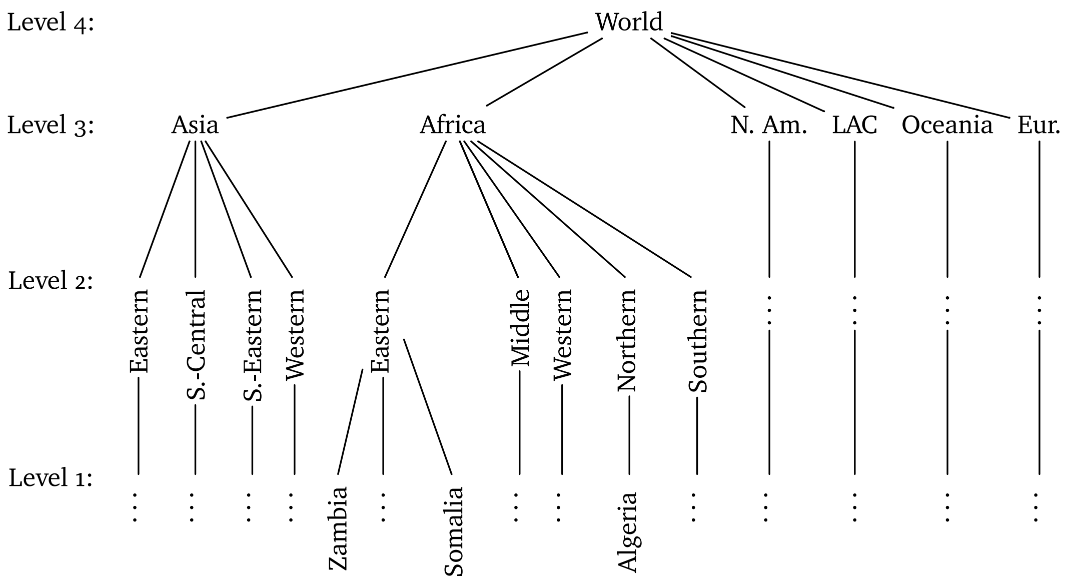

(i) Geographic model used for the majority of parameters in the married women model. All clusters at Levels 3 and 4 are shown but, for clarity, only a subset of clusters at Level 2 (sub-regions) and Level 1 (countries) are.

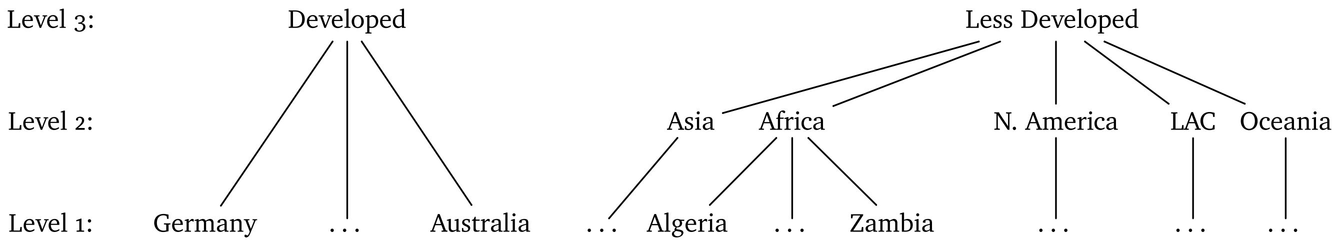

(ii) Development / Geographic model used for the remainder of parameters in the married women model. All clusters at Levels 2–4 are shown but, for clarity, only a few example Level 1 clusters (countries) are. The terms “developed” and “less developed” are used for statistical convenience and do not necessarily express a judgment about the stage reached by a particular country or area in the development process. The developed countries group comprises all regions of Europe plus Northern America, Australia/New Zealand and Japan. The developing countries group comprises all regions of Africa, Asia (excluding Japan) and Latin America and the Caribbean, as well as Melanesia, Micronesia and Polynesia (Alkema et al., 2013, Supplementary appendix, Subsection 1.1).

Figure C. Hierarchical structures used in the married and unmarried women models (see also Table A).

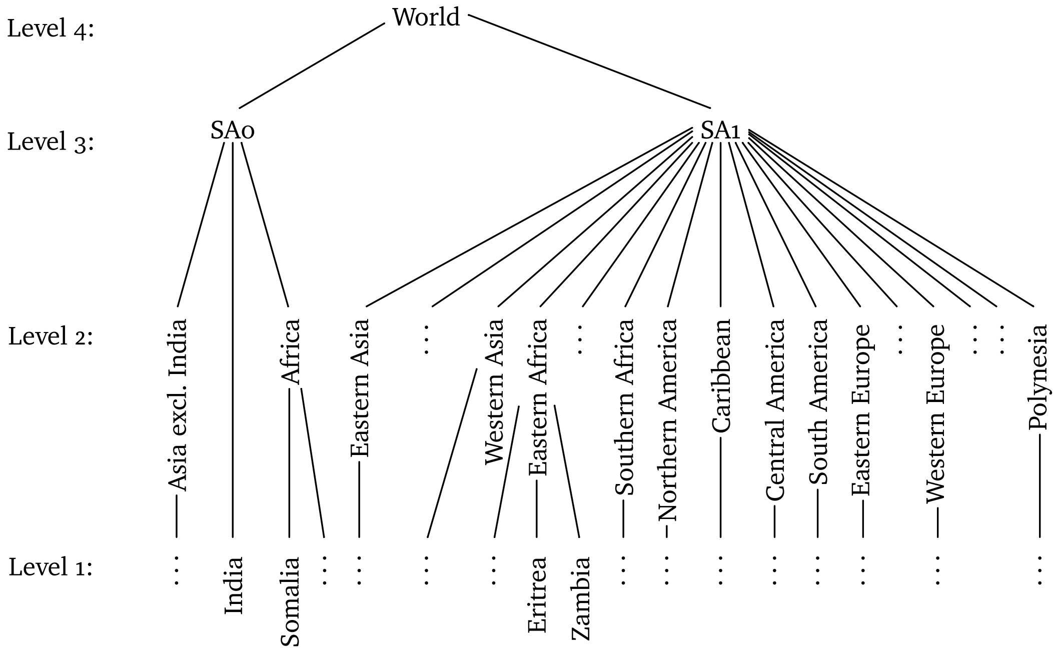

(iii) Hierarchical structure used for the majority of parameters in the unmarried women model. Level 1 consists of individual countries which, save the examples, are omitted due to lack of space. "SA0" and "SA1" are Sexual Activity Groups 0 and 1, respectively.

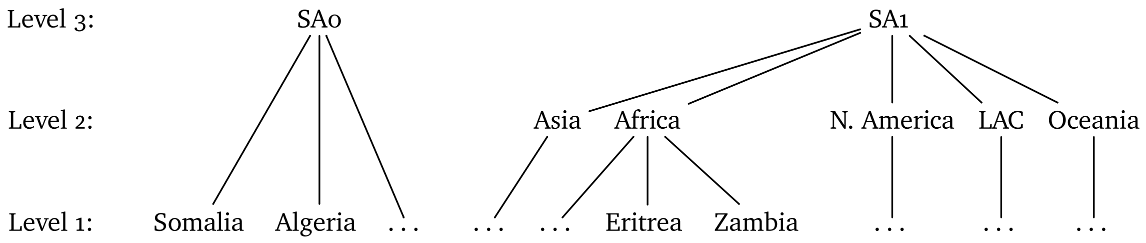

(iv) Hierarchical structure used for the remainder of parameters in the unmarried women model. Level 1 consists of individual countries which, save the examples, are omitted due to lack of space. "SA0" and "SA1" are Sexual Activity Groups 0 and 1, respectively.

Figure C. (cont'd)

Exercise 1 Leave out 20 percent of the observations within each country at random.

Exercise 2 Leave out approximately the most recent 20 percent of observations. For married women, this meant leaving out all observations after 2012 (22 percent) and, for unmarried women, all observations after 2014 (23 percent).

Exercise 3 Leave out all unmet need observations from 20 percent of countries, randomly chosen from the set of all countries with at least one such observation.

Exercise 1 assesses general out-of-sample performance across the time period of estimation and indicator components. Exercise 3 does the same but is based only on observations of unmet need. Exercise 2 assesses forecast performance within the period of data availability by leaving out all observations after a given year. We used the mid-year of each observation's reference period for this purpose. We wanted to keep the training and test set proportions as similar as possible across married and unmarried models. Differences in data availability meant that this required choosing slightly different cut-off years.

Random cross-validation exercises such as these are subject to noise, and this can be significant in reasonably small datasets such as ours. We attempted to ameliorate this by repeating Exercises 1 and 3 five times, each time with a different randomly chosen test set, and averaging over the results. Exercise 2 could not be replicated as the test set is chosen deterministically.

The following measures were used to summarize the results:

1. Median prediction error (MPE) and median absolute prediction error (MAPE).
   For example, the error in predicting test-set observation y_i was computed as
   $$e_i = y_i - \hat{y}_i,$$
   where $\hat{y}_i$ is the predictive posterior median of y_i from the model fitted to the training set. The MPE was computed as the median of the e_i, the MAPE the median of the $|e_i|$, both taken over the observations in the test set.

2. Proportion of the left out observations less than their posterior predictive median. If the model is well calibrated, we expect this to be around 50 percent.

3. Coverage of 95 percent prediction intervals with respect to the left out observations.
   This was defined as the proportion of the left out observations that fell inside the respective posterior predictive intervals. If the model is well calibrated, and if the left out observations are independent from one another, we expect this to be the nominal level (e.g,. for 95 percent intervals, this should be close to 0.95).

Only one left out observation per country was used to calculate the above measures so as to reduce bias due to dependence among observations within country.

3 Results

3.1 Model Validation

Validation results are shown in Table B for (i) the married women model and (ii) the unmarried women model. They show that the uncertainty intervals have good coverage over the different indicator components for both married and unmarried women when observations are excluded at random (Exercise 1). This is the most general of the Exercises performed.

Exercise 2 was designed to assess the coverage of forecasts of future values. Only one replicate per marital status model was possible due to the deterministic definition of the test set, so results are noisier than for the

other two exercises which were able to be replicated. Nevertheless, the coverages of the 95 percent posterior uncertainty intervals are good and the proportions below the median for the different components are close to the nominal value of 50 percent in most cases. The values for the married women model are higher for the "Total" and "Traditional" components (65% and 64% resp.), however, the prediction errors are, again, quite small. In this work, though, we are primarily interested in the use of modern contraceptive methods and the proportion below the median was very good for this component (55%).

In both models, the numbers of observations upon which the Exercise 3 replicates were based were very small (20 and 15 for the married and unmarried models, resp.). Nevertheless, the coverages of the uncertainty intervals were close to the nominal value of 95 percent. For the married women model, the proportion below the median was acceptable at 45 percent. The MAPE was 6.1 percent but the MPE was 1.9, suggesting the prediction errors were dispersed but centered only a couple of percentage points above zero. The proportion below the median was further from the nominal value for the unmarried women model but both the MPE and MAPE were small.

Table B. Summary of model validation results based on out-of-sample validation experiments; see Section 2.2 for a description of the exercises. For each exercise and component, the values are the proportion of left out observations that fall outside, or inside, the respective 95% prediction intervals, and below their posterior predictive median estimate, and their median error (ME) and median absolute error (MAE). The '# Obs' column gives the number of observations in the test set in each replication of each exercise. Exercises 1 and 3 were repeated five times with different randomly chosen test sets of size '# Obs'.

(i) Married women model

		95% prediction interval			Median	Prediction Errors (%)	
Component	# Obs	%Below	%Within	%Above	% Below	MPE	MAPE
		Exercise 1 (leave out 20% of obs.)					
Total	80	3.7	94.3	2.0	53.1	-0.1	3.5
Modern	80	1.5	95.3	3.3	49.1	-0.0	2.7
Traditional	80	4.4	92.8	2.8	49.1	0.1	1.4
Unmet	54	2.2	94.6	3.1	49.1	0.2	4.4
		Exercise 2 (leave out all obs. after 2012)					
Total	84	4.8	95.2	0.0	65.5	-2.0	4.7
Modern	84	1.2	98.8	0.0	54.8	-1.0	3.4
Traditional	84	2.4	97.6	0.0	64.3	-0.5	1.1
Unmet	67	1.5	94.0	4.5	43.3	1.2	5.2
		Exercise 3 (leave out all unmet need obs. from 20% of countries)					
Unmet	20	3.0	96.0	1.0	45.0	1.9	6.1
		Values Expected					
		2.5	95.0	2.5	50.0		

Table B. (cont'd).

(ii) Unmarried women model

Component	# Obs	95% prediction interval %Below	%Within	%Above	Median % Below	Prediction Errors (%) MPE	MAPE
		Exercise 1 (leave out 20% of obs.)					
Total	53	3.4	94.7	1.9	56.3	-0.1	1.1
Modern	53	3.4	94.3	2.3	54.8	-0.0	1.1
Traditional	53	3.9	95.0	1.2	54.7	-0.0	0.3
Unmet	35	2.3	96.0	1.7	41.2	0.3	0.9
		Exercise 2 (leave out all obs. after 2014)					
Total	60	1.7	96.7	1.7	56.7	-0.1	1.3
Modern	60	3.3	96.7	0.0	53.3	-0.0	1.7
Traditional	60	0.0	98.3	1.7	51.7	-0.0	0.2
Unmet	49	6.1	93.9	0.0	51.0	-0.0	1.1
		Exercise 3 (leave out all unmet need obs. from 20% of countries)					
Unmet	15	4.0	96.0	0.0	41.3	0.7	1.7
		Values Expected					
		2.5	95.0	2.5	50.0		

3.2 Estimates and Projections for Level 2 Regions

Estimates and projections of the family planning indicators are plotted for Level 2 regions in Figure D. These results are supplementary to the results for Level 1 regions presented in the main manuscript.

3.3 Ternary Color Maps with Re-centered Color Scales

In most countries, the majority of adolescent women aged 15–19 who are unmarried and not in-union experienced no need for family planning in 2019. This makes is difficult to identify relative differences among countries from a plot using a ternary color scale centered at the point (1/3, 1/3, 1/3), as in Figures 8 and 9 in the main article. Relative differences can be highlighted by re-centering the color scale to the compositional mean (e.g., Pawlowsky-Glahn et al., 2015). We do this for unmarried and all women in Figures E and F. Maps with centered color schemes are useful for comparing countries in the same population (i.e., in the same map). They cannot be used for cross-population comparisons.

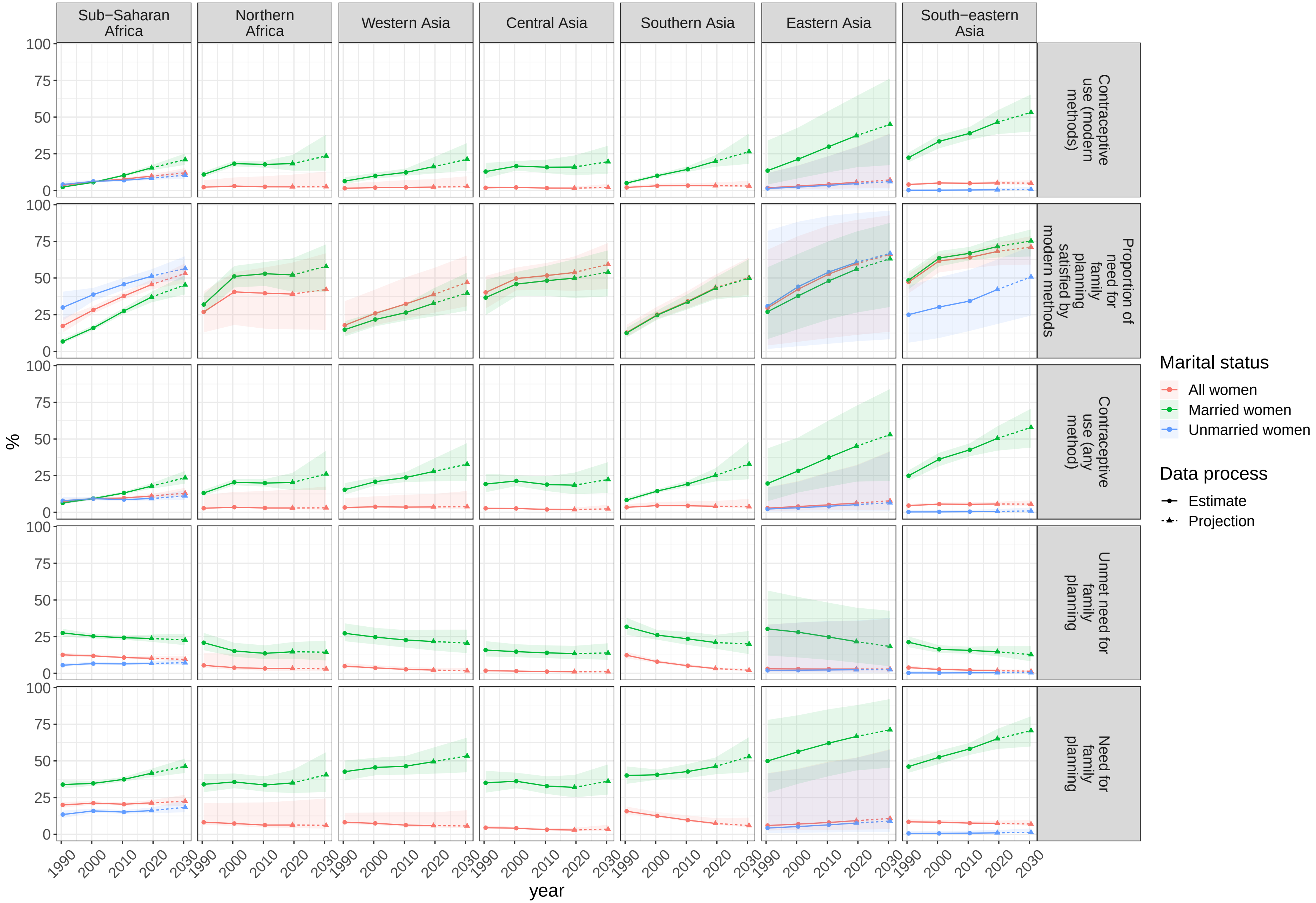

(i)

Figure D. Estimates and projections of the proportion of adolescent women (15-19 years) using contraception (any method and modern methods) and having unmet need for family planning, by marital status and regions, 1990–2030. Given are the posterior medians (solid lines) posterior 95% uncertainty intervals (ribbons).

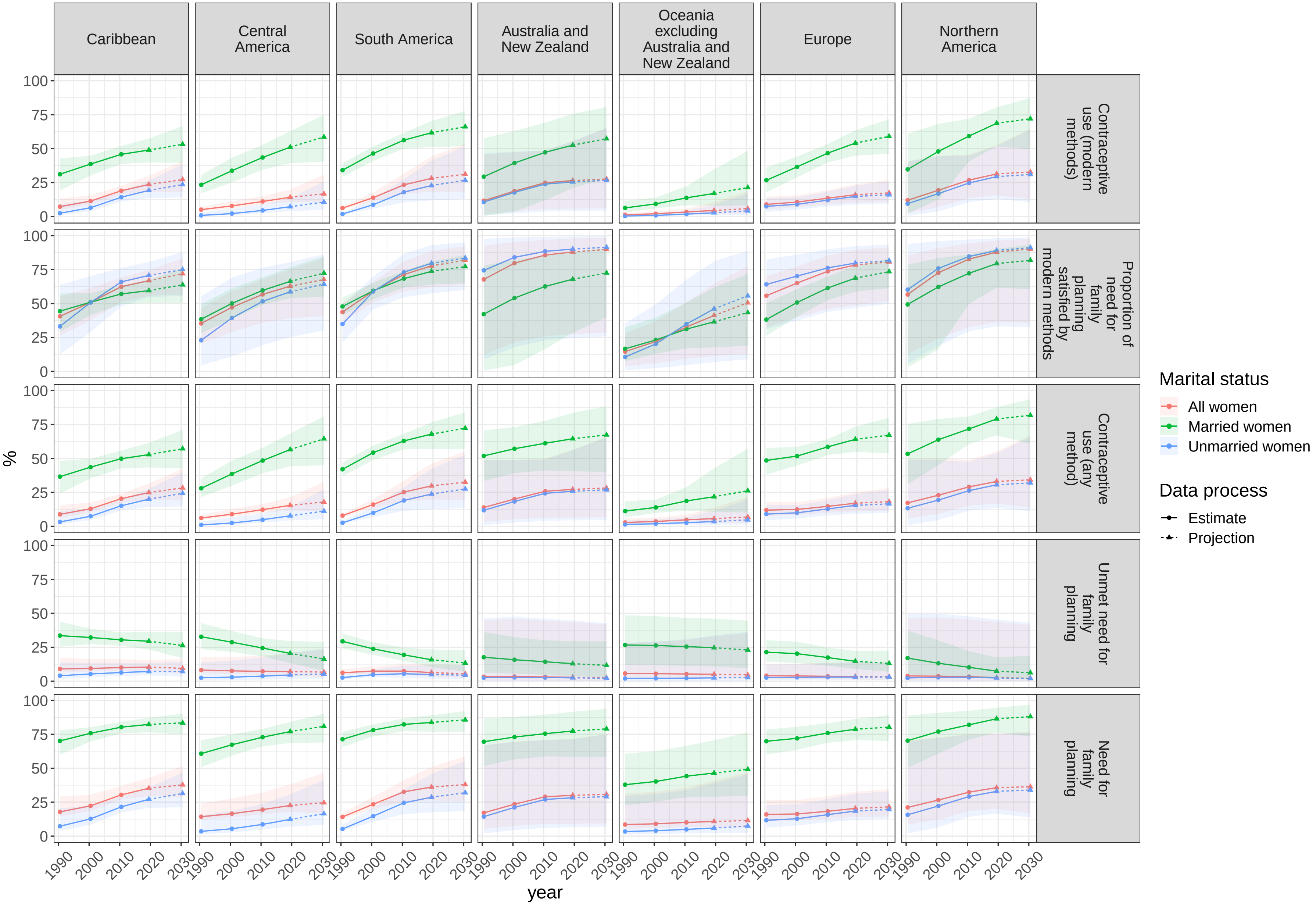

(ii)

Figure D. (cont'd).

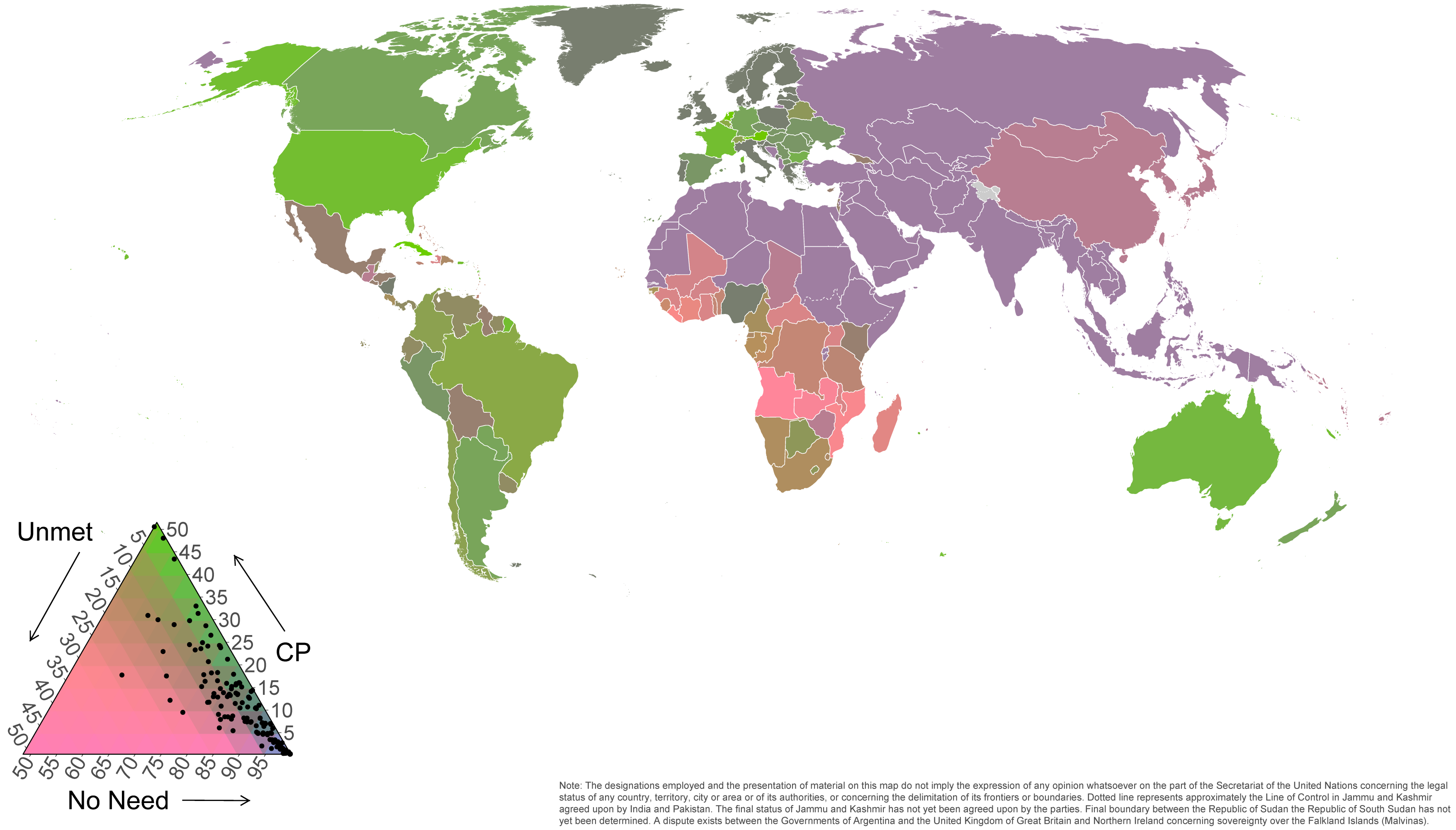

Figure E. Ternary color scheme map with centered color scheme for unmarried adolescent women aged 15–19, 2019. The map shows posterior median estimates of contraceptive use (any method), unmet need for family planning, and no need for family planning. The color scheme is centered at the compositional mean so that relative differences among countries are highlighted. However, it also means that this map is not directly comparable with the other ternary color scheme maps. Note that the legend has been cropped.

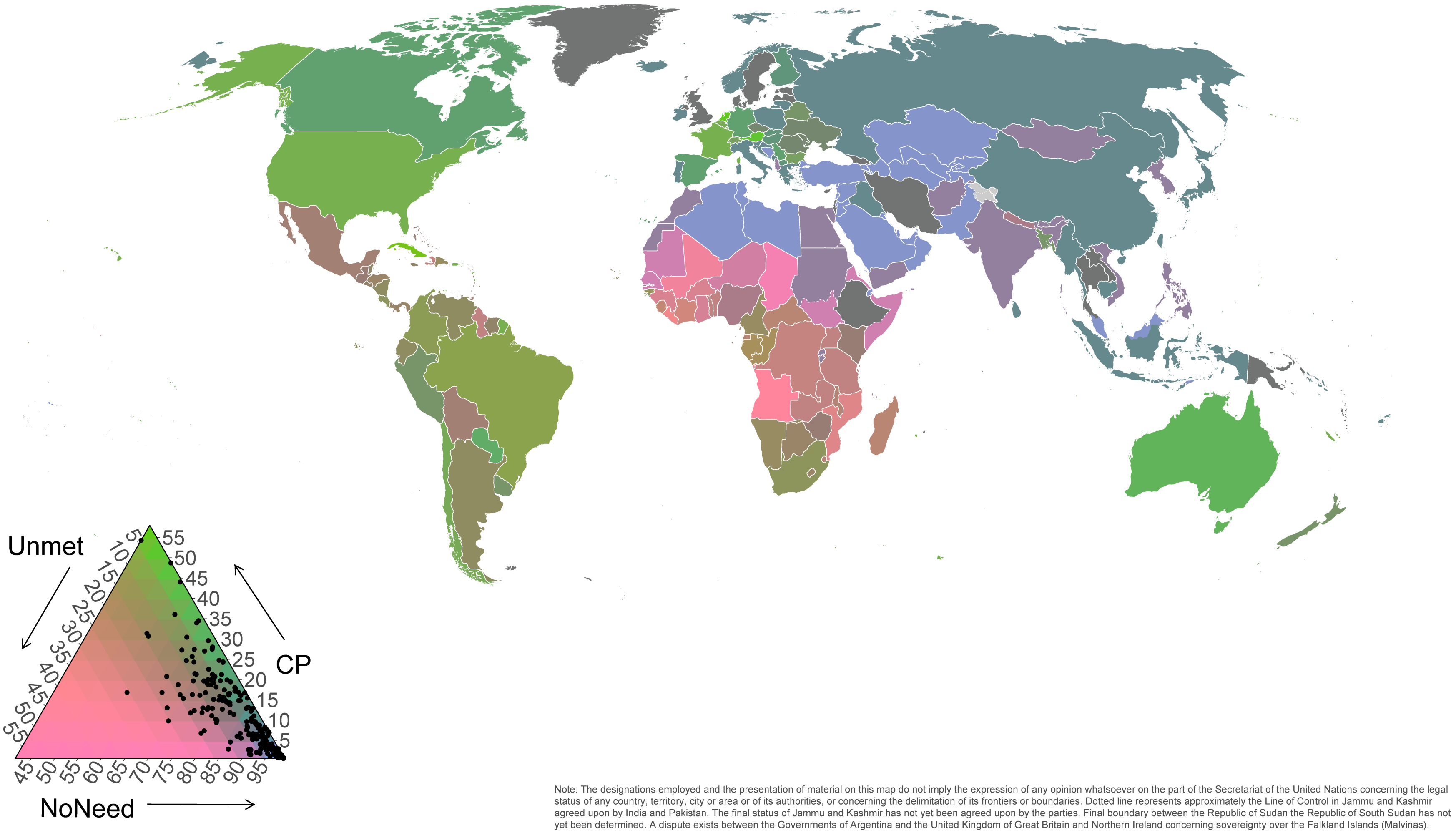

Figure F. Ternary color scheme map with centered color scheme for all adolescent women aged 15–19, 2019. The map shows posterior median estimates of contraceptive use (any method), unmet need for family planning, and no need for family planning. The color scheme is centered at the compositional mean so that relative differences among countries are highlighted. However, it also means that this map is not directly comparable with the other ternary color scheme maps. Note that the legend has been cropped.

Appendix

Table C. Country and region classifications used in the married and unmarried model hierarchies. Sub-Saharan Africa consists of all countries in Africa except those in Northern Africa. "Lat. Am. & Caribb." is Latin America and the Caribbean; "PEW GRL" = Pew Global Religious Landscape Survey (Pew Research Center, 2012); "PEW GAS" = Pew Global Attitudes Survey (Pew Research Center, 2014). The terms "developed" and "less developed" are used for statistical convenience and do not necessarily express a judgment about the stage reached by a particular country or area in the development process. The developed countries group comprises all regions of Europe plus Northern America, Australia/New Zealand and Japan. The developing countries group comprises all regions of Africa, Asia (excluding Japan) and Latin America and the Caribbean, as well as Melanesia, Micronesia and Polynesia. The term "country" as used in this study also refers, as appropriate, to territories or areas.

Region	Subregion	Name	Development Group	Sexual Activity Group	Source data for Sexual Activity group
Africa	Eastern Africa	Djibouti	Less developed	0	PEW GRL
		Somalia	Less developed	0	PEW GRL
		South Sudan	Less developed	0	DHS/MICS
		Burundi	Less developed	1	DHS/MICS
		Comoros	Less developed	1	DHS/MICS
		Eritrea	Less developed	1	DHS/MICS
		Ethiopia	Less developed	1	DHS/MICS
		Kenya	Less developed	1	DHS/MICS
		Madagascar	Less developed	1	DHS/MICS
		Malawi	Less developed	1	DHS/MICS
		Mauritius	Less developed	1	PEW GRL
		Mozambique	Less developed	1	DHS/MICS
		Réunion	Less developed	1	PEW GRL
		Rwanda	Less developed	1	DHS/MICS
		Uganda	Less developed	1	DHS/MICS
		United Rep. of Tanzania	Less developed	1	DHS/MICS
		Zambia	Less developed	1	DHS/MICS
		Zimbabwe	Less developed	1	DHS/MICS
	Middle Africa	Angola	Less developed	1	PEW GRL
		Cameroon	Less developed	1	DHS/MICS
		Central African Republic	Less developed	1	DHS/MICS
		Chad	Less developed	1	DHS/MICS
		Congo	Less developed	1	DHS/MICS
		Democratic Rep. of the Congo	Less developed	1	DHS/MICS
		Equatorial Guinea	Less developed	1	PEW GRL
		Gabon	Less developed	1	DHS/MICS

Table C. Country and region classifications (cont'd).

Region	Subregion	Name	Development Group	Sexual Activity Group	Source data for Sexual Activity group
		Sao Tome and Principe	Less developed	1	DHS/MICS
	Northern Africa	Algeria	Less developed	0	PEW GRL
		Egypt	Less developed	0	PEW GAS
		Libya	Less developed	0	World Values Survey
		Morocco	Less developed	0	DHS/MICS
		Sudan	Less developed	0	PEW GRL
		Tunisia	Less developed	0	PEW GAS
	Southern Africa	Botswana	Less developed	1	DHS/MICS
		Eswatini	Less developed	1	DHS/MICS
		Lesotho	Less developed	1	DHS/MICS
		Namibia	Less developed	1	DHS/MICS
		South Africa	Less developed	1	DHS/MICS
	Western Africa	Mauritania	Less developed	0	DHS/MICS
		Niger	Less developed	0	DHS/MICS
		Benin	Less developed	1	DHS/MICS
		Burkina Faso	Less developed	1	DHS/MICS
		Cabo Verde	Less developed	1	PEW GRL
		Côte d'Ivoire	Less developed	1	DHS/MICS
		Gambia	Less developed	1	DHS/MICS
		Ghana	Less developed	1	DHS/MICS
		Guinea	Less developed	1	DHS/MICS
		Guinea-Bissau	Less developed	1	DHS/MICS
		Liberia	Less developed	1	DHS/MICS
		Mali	Less developed	1	DHS/MICS
		Nigeria	Less developed	1	DHS/MICS
		Senegal	Less developed	1	DHS/MICS
		Sierra Leone	Less developed	1	DHS/MICS
		Togo	Less developed	1	DHS/MICS
Asia	Central Asia	Tajikistan	Less developed	0	DHS/MICS
		Turkmenistan	Less developed	0	DHS/MICS
		Uzbekistan	Less developed	0	DHS/MICS
		Kazakhstan	Less developed	1	DHS/MICS
		Kyrgyzstan	Less developed	1	DHS/MICS
	Eastern Asia	Japan	Developed	1	PEW GAS

Table C. Country and region classifications (cont'd).

Region	Subregion	Name	Development Group	Sexual Activity Group	Source data for Sexual Activity group
		China	Less developed	1	PEW GAS
		China, Hong Kong SAR	Less developed	1	World Values Survey
		Democratic People's Rep. of Korea	Less developed	1	PEW GRL
		Mongolia	Less developed	1	PEW GRL
		Republic of Korea	Less developed	1	PEW GAS
	South-eastern Asia	Cambodia	Less developed	0	DHS/MICS
		Indonesia	Less developed	0	DHS/MICS
		Lao People's Dem. Republic	Less developed	0	DHS/MICS
		Malaysia	Less developed	0	PEW GAS
		Myanmar	Less developed	0	PEW GRL
		Democratic Rep. of Timor-Leste	Less developed	0	DHS/MICS
		Viet Nam	Less developed	0	DHS/MICS
		Philippines	Less developed	1	DHS/MICS
		Singapore	Less developed	1	World Values Survey
		Thailand	Less developed	1	World Values Survey
	Southern Asia	Afghanistan	Less developed	0	PEW GRL
		Bangladesh	Less developed	0	PEW GRL
		Bhutan	Less developed	0	DHS/MICS
		India	Less developed	0	DHS/MICS
		Iran, Islamic Republic of	Less developed	0	PEW GRL
		Maldives	Less developed	0	PEW GRL
		Nepal	Less developed	0	DHS/MICS
		Pakistan	Less developed	0	PEW GAS
		Sri Lanka	Less developed	0	PEW GRL
	Western Asia	Israel	Developed	1	PEW GAS
		Armenia	Less developed	0	DHS/MICS
		Azerbaijan	Less developed	0	DHS/MICS
		Bahrain	Less developed	0	PEW GRL
		Iraq	Less developed	0	PEW GRL
		Jordan	Less developed	0	PEW GAS
		Kuwait	Less developed	0	PEW GRL
		Lebanon	Less developed	0	PEW GAS
		Oman	Less developed	0	PEW GRL
		Qatar	Less developed	0	World Values Survey
		Saudi Arabia	Less developed	0	PEW GRL

Table C. Country and region classifications (cont'd).

Region	Subregion	Name	Development Group	Sexual Activity Group	Source data for Sexual Activity group
		State of Palestine	Less developed	0	PEW GAS
		Syrian Arab Republic	Less developed	0	PEW GRL
		Turkey	Less developed	0	PEW GAS
		United Arab Emirates	Less developed	0	PEW GRL
		Yemen	Less developed	0	PEW GRL
		Georgia	Less developed	1	World Values Survey
Europe	Eastern Europe	Belarus	Developed	1	DHS/MICS
		Bulgaria	Developed	1	PEW GRL
		Czechia	Developed	1	PEW GAS
		Hungary	Developed	1	PEW GRL
		Poland	Developed	1	PEW GAS
		Republic of Moldova	Developed	1	DHS/MICS
		Romania	Developed	1	World Values Survey
		Russian Federation	Developed	1	PEW GAS
		Slovakia	Developed	1	PEW GRL
		Ukraine	Developed	1	DHS/MICS
	Northern Europe	Denmark	Developed	1	PEW GRL
		Estonia	Developed	1	World Values Survey
		Finland	Developed	1	PEW GRL
		Ireland	Developed	1	PEW GRL
		Latvia	Developed	1	PEW GRL
		Lithuania	Developed	1	PEW GRL
		Norway	Developed	1	PEW GRL
		Sweden	Developed	1	World Values Survey
		United Kingdom	Developed	1	PEW GAS
	Southern Europe	Albania	Developed	1	DHS/MICS
		Bosnia and Herzegovina	Developed	1	DHS/MICS
		Croatia	Developed	1	PEW GRL
		Greece	Developed	1	PEW GAS
		Italy	Developed	1	PEW GAS
		Malta	Developed	1	PEW GRL
		Montenegro	Developed	1	DHS/MICS
		Portugal	Developed	1	PEW GRL
		Serbia	Developed	1	DHS/MICS
		Slovenia	Developed	1	World Values Survey

Table C. Country and region classifications (cont'd).

Region	Subregion	Name	Development Group	Sexual Activity Group	Source data for Sexual Activity group
		Spain	Developed	1	PEW GAS
		TFYR Macedonia	Developed	1	DHS/MICS
	Western Europe	Austria	Developed	1	PEW GRL
		Belgium	Developed	1	PEW GRL
		France	Developed	1	PEW GAS
		Germany	Developed	1	PEW GAS
		Netherlands	Developed	1	World Values Survey
		Switzerland	Developed	1	PEW GRL
Lat. Am. & Caribb.	Caribbean	Anguilla	Less developed	1	PEW GRL
		Antigua and Barbuda	Less developed	1	PEW GRL
		Bahamas	Less developed	1	PEW GRL
		Barbados	Less developed	1	DHS/MICS
		Cuba	Less developed	1	PEW GRL
		Dominica	Less developed	1	PEW GRL
		Dominican Republic	Less developed	1	DHS/MICS
		Grenada	Less developed	1	PEW GRL
		Guadeloupe	Less developed	1	PEW GRL
		Haiti	Less developed	1	DHS/MICS
		Jamaica	Less developed	1	PEW GRL
		Martinique	Less developed	1	PEW GRL
		Montserrat	Less developed	1	PEW GRL
		Puerto Rico	Less developed	1	PEW GRL
		Saint Kitts and Nevis	Less developed	1	PEW GRL
		St. Lucia	Less developed	1	DHS/MICS
		St. Vincent and the Grenadines	Less developed	1	PEW GRL
		Trinidad and Tobago	Less developed	1	DHS/MICS
		United States Virgin Islands	Less developed	1	PEW GRL
	Central America	Belize	Less developed	1	DHS/MICS
		Costa Rica	Less developed	1	PEW GRL
		El Salvador	Less developed	1	PEW GAS
		Guatemala	Less developed	1	DHS/MICS
		Honduras	Less developed	1	DHS/MICS
		Mexico	Less developed	1	PEW GAS
		Nicaragua	Less developed	1	PEW GRL
		Panama	Less developed	1	PEW GRL

Table C. Country and region classifications (cont'd).

Region	Subregion	Name	Development Group	Sexual Activity Group	Source data for Sexual Activity group
	South America	Argentina	Less developed	1	PEW GAS
		Bolivia, Plurinational State of	Less developed	1	DHS/MICS
		Brazil	Less developed	1	DHS/MICS
		Chile	Less developed	1	PEW GAS
		Colombia	Less developed	1	DHS/MICS
		Ecuador	Less developed	1	World Values Survey
		Guyana	Less developed	1	DHS/MICS
		Paraguay	Less developed	1	DHS/MICS
		Peru	Less developed	1	DHS/MICS
		Suriname	Less developed	1	DHS/MICS
		Uruguay	Less developed	1	World Values Survey
		Venezuela, Bolivarian Republic of	Less developed	1	PEW GAS
Northern America	Northern America	Canada	Developed	1	PEW GAS
		United States of America	Developed	1	PEW GAS
Oceania	Australia and New Zealand	Australia	Developed	1	PEW GAS
		New Zealand	Developed	1	World Values Survey
	Melanesia	Fiji	Less developed	1	PEW GRL
		Papua New Guinea	Less developed	1	PEW GRL
		Solomon Islands	Less developed	1	PEW GRL
		Vanuatu	Less developed	1	PEW GRL
	Micronesia	Guam	Less developed	1	PEW GRL
		Kiribati	Less developed	1	PEW GRL
		Marshall Islands	Less developed	1	PEW GRL
		Nauru	Less developed	1	PEW GRL
		Palau	Less developed	1	PEW GRL
	Polynesia	Cook Islands	Less developed	1	PEW GRL
		Samoa	Less developed	1	PEW GRL
		Tonga	Less developed	1	PEW GRL
		Tuvalu	Less developed	1	PEW GRL

References

Alkema, L., Kantorova, V., Menozzi, C., and Biddlecom, A. (2013). National, regional, and global rates and trends in contraceptive prevalence and unmet need for family planning between 1990 and 2015: a systematic and comprehensive analysis. *The Lancet* 381 (9878), pp. 1642–1652. DOI: 10.1016/S0140-6736(12)62204-1.

Cahill, N., Sonneveldt, E., Stover, J., Weinberger, M., Williamson, J., Wei, C., Brown, W., and Alkema, L. (2017). Modern contraceptive use, unmet need, and demand satisfied among women of reproductive age who are married or in a union in the focus countries of the family planning 2020 initiative: a systematic analysis using the family planning estimation tool. *The Lancet* 391 (10123), pp. 870–882. DOI: 10.1016/S0140-6736(17)33104-5.

Inglehart, R., Haerpfer, C., Moreno, A., Welze, C., Kizilova, K., Diez-Medrano, J., Lagos, M., Norris, P., Ponarin, E., and Puranen, B. (2013). *World Values Survey: Round 6*. URL: http://www.worldvaluessurvey.org/WVSDocumentationWV6.jsp (visited on 14 July 2017).

Kantorová, V., Wheldon, M. C., Ueffing, P., and Dasgupta, A. N. Z. (2020). Estimating progress towards meeting women's contraceptive needs in 185 countries: a Bayesian hierarchical modelling study. *PLOS Medicine* 17 (2), e1003026. DOI: 10/ggk3cf.

Pawlowsky-Glahn, V., Egozcue, J. J., and Tolosana-Delgado, R. (2015). *Modeling and Analysis of Compositional Data: Modeling and Analysis of Compositional Data*. New York, NY: John Wiley & Sons, Incorporated.

Pew Research Center (2012). *The Global Religious Landscape: A Report on the Size and Distribution of the World's Major Religious Groups*. URL: http://www.pewforum.org/2012/12/18/global-religious-landscape-exec/ (visited on 16 Aug. 2017).

Pew Research Center (2014). *Global Attitudes Survey Spring 2013*. URL: http://www.pewglobal.org/2014/04/15/global-morality/table/premarital-sex/ (visited on 14 July 2017).

Ueffing, P., Dasgupta, A. N. Z., and Kantorová, V. (2019). Sexual activity by marital status and age: a comparative perspective. *Journal of Biosocial Science*, pp. 1–25. DOI: 10.1017/S002193201900083X.
