# Supplementary material for: Contraceptive use and needs among adolescent women aged 15–19: Regional and global estimates and projections from 1990 to 2030 from a Bayesian hierarchical modelling study
Source: PLoS One. 2021 Mar 4;16(3):e0247479. doi: 10.1371/journal.pone.0247479 (PMC7932081; doi:10.1371/journal.pone.0247479)
Supplement: S1 File — (PDF) [file pone.0247479.s011.pdf]

Contraceptive Use and Needs among Adolescent Women Aged 15-19: Regional and Global Estimates and Projections from 1990 to 2030 from a Bayesian Hierarchical Modelling Study

S1 APPENDIX: Supplementary Figures

Vladimíra Kantorová*[1], Mark C. Wheldon[1], Aisha N. Z. Dasgupta[1], Philipp Ueffing[1], and Helena Cruz Castanheira[2]

[1]United Nations, Department of Economic and Social Affairs, Population Division, New York, NY, United States of America†
[2]United Nations, Economic Commission for Latin America and the Caribbean, Santiago de Chile, Chile†

6th May 2020

*Corresponding author. Vladimíra Kantorová, Population Division, Department of Economic and Social Affairs, United Nations, kantorova@un.org

†The views and opinions expressed in this paper are those of the authors and do not necessarily represent those of the United Nations. This paper has not been formally edited and cleared by the United Nations.

Contents

1 **Data and Methods** 1

2 **Estimates and Projections for Level 2 Regions** 1

3 **Ternary Colour Maps with Re-centred Colour Scales** 1

4 **Country-Specific Estimates and Projections** 7
- 4.1 Unmarried Adolescent Women . . . 7
- 4.2 Married Adolescent Women . . . 94
- 4.3 All Adolescent Women . . . 225

List of Figures

- A Data availability by region, time-period and marital status. . . . 2
- B Estimates and projections by Level 2 regions. . . . 3
- B (cont'd). . . . 4
- C Ternary colour scheme map with centred colour scheme, unmarried adolescent women aged 15–19. . . . 5
- D Ternary colour scheme map with centred colour scheme, all adolescent women aged 15–19. . . 6

1 Data and Methods

Data availability by region, time-period, and marital status is summarized in Figure A.

2 Estimates and Projections for Level 2 Regions

Estimates and projections are plotted for Level 2 regions in Figure B.

3 Ternary Colour Maps with Re-centred Colour Scales

In most countries, the majority of adolescent women aged 15–19 who are unmarried and not in-union experienced no need for family planning in 2019. This makes is difficult to identify relative differences among countries from a plot using a ternary colour scale centred at the point (1/3, 1/3, 1/3), as in Figures 8 and 9 in the main article. Relative differences can be highlighted by re-centring the colour scale to the compositional mean (e.g., Pawlowsky-Glahn et al., 2015) of the data. We do this for unmarried and all women in Figures C and D. Maps with centred colour schemes are useful for comparing countries in the same population (i.e., in the same map). They cannot be used for cross-population comparisons.

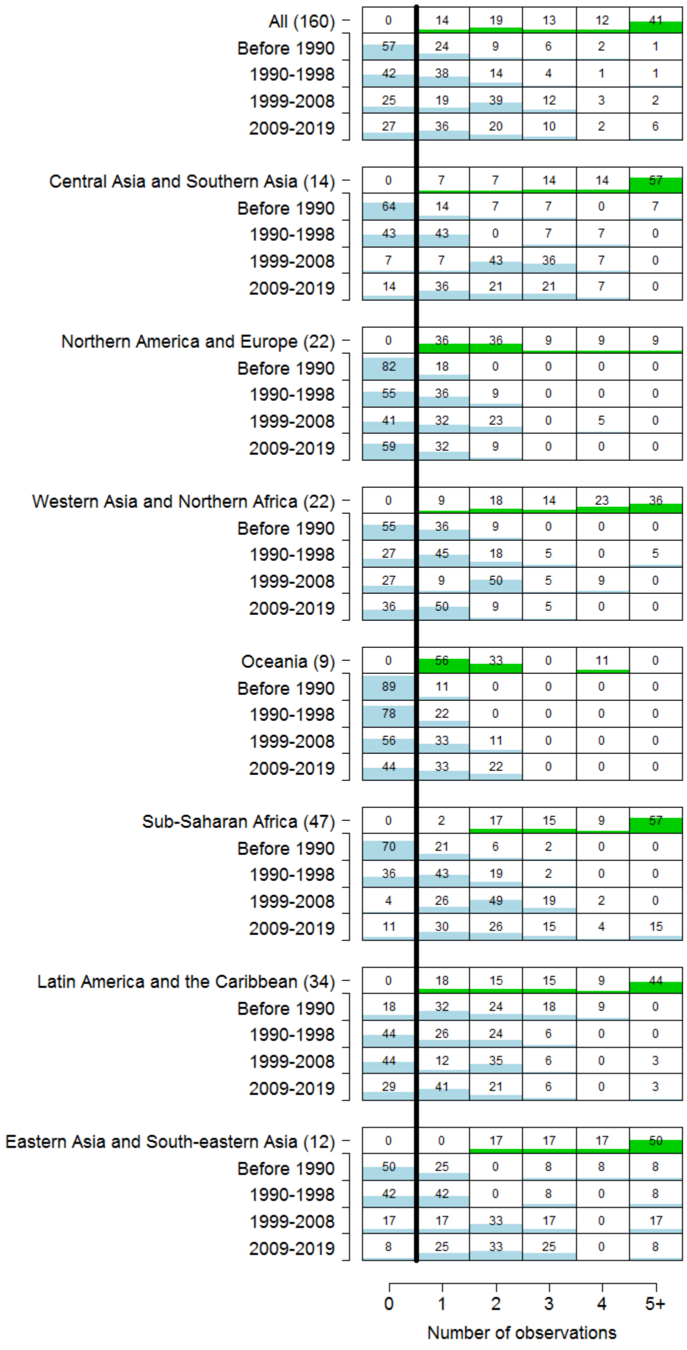

(i) Married women

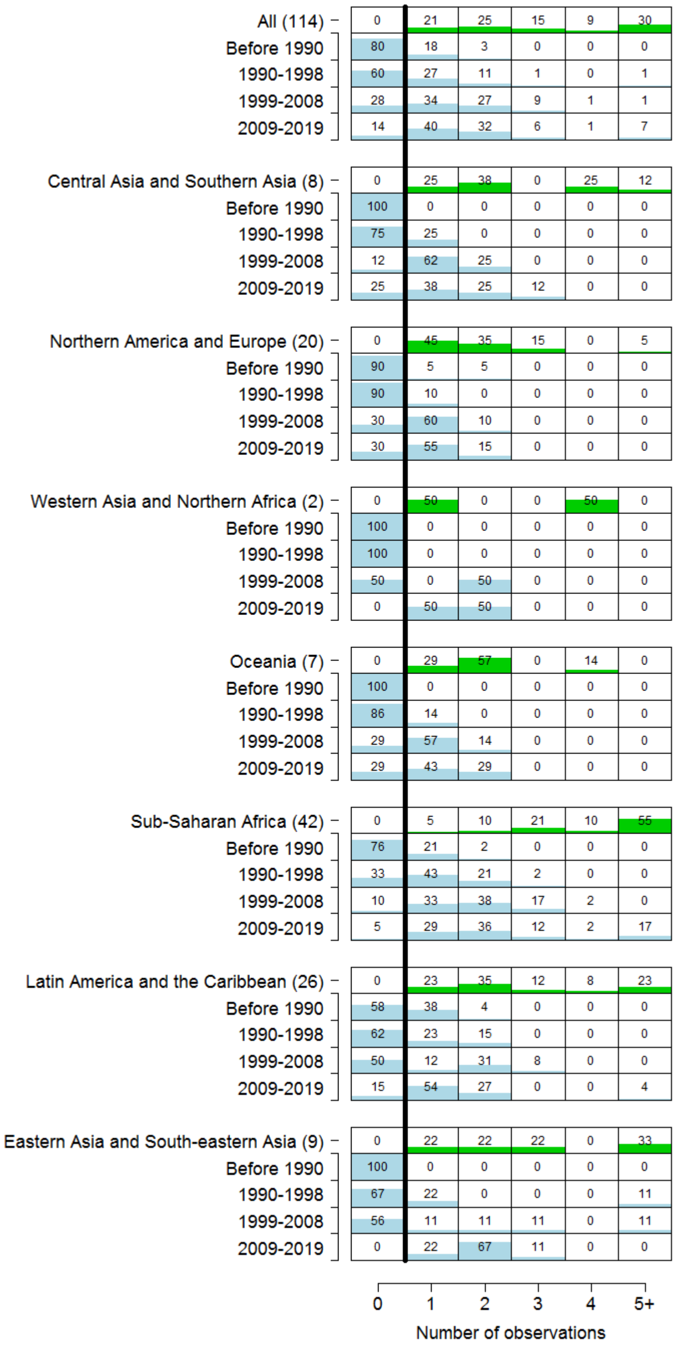

(ii) Unmarried women

Figure A. Data availability by region, time-period and marital status. Proportion of countries by number of observations of contraceptive prevalence (any method) by region and time period for (i) married and (ii) unmarried women aged 15–19. The cells give the percentage of all countries for the given region and time period with the number of observations according to column. The cells sum to 100 (within rounding) across each row. The numbers in parentheses next to the region names are the number of countries in that region with at least one observation

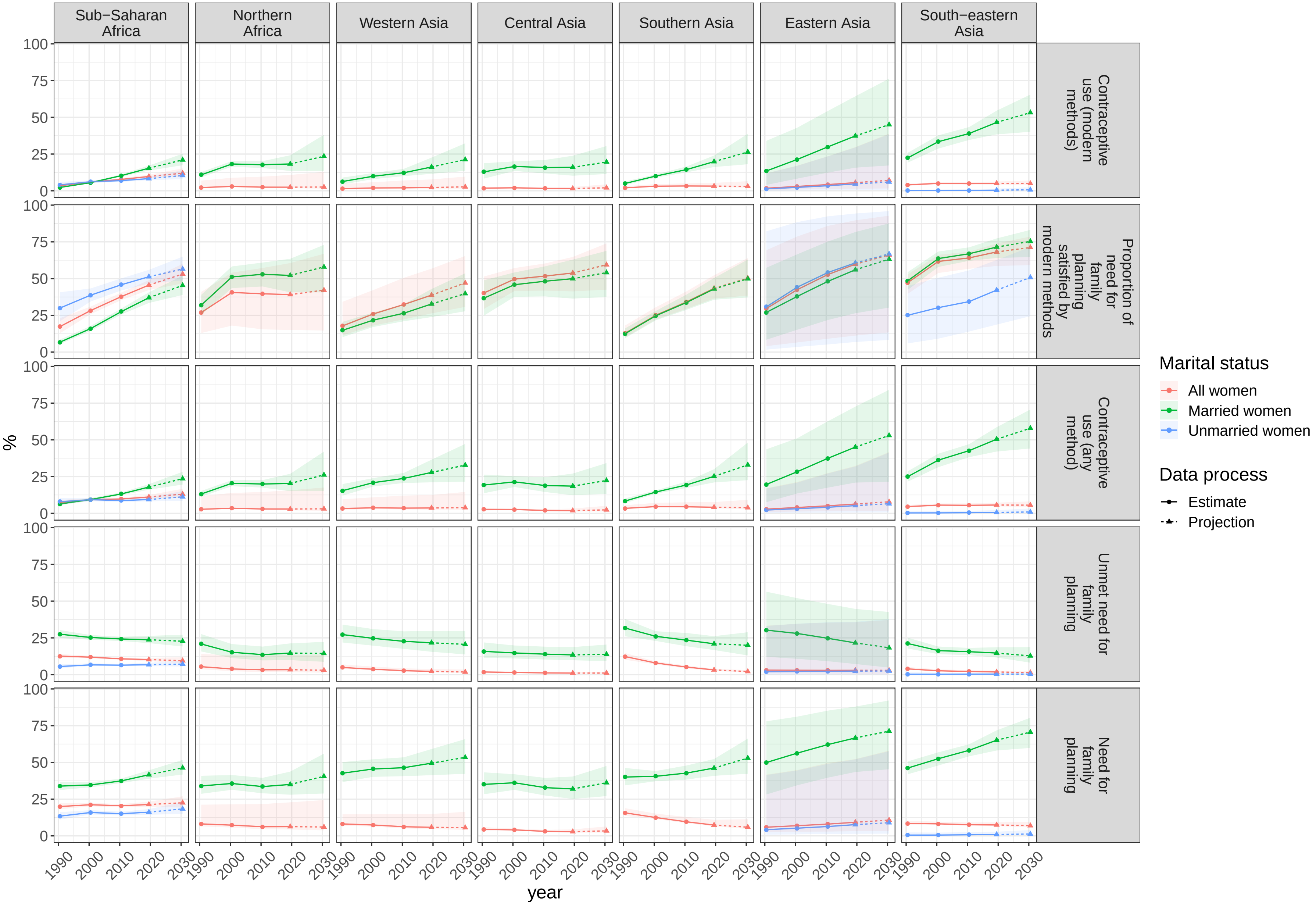

(i)

Figure B. Estimates and projections of the proportion of adolescent women (15-19 years) using contraception (any method and modern methods) and having unmet need for family planning, by marital status and regions, 1990–2030. Given are the posterior medians (solid lines) posterior 95% uncertainty intervals (ribbons).

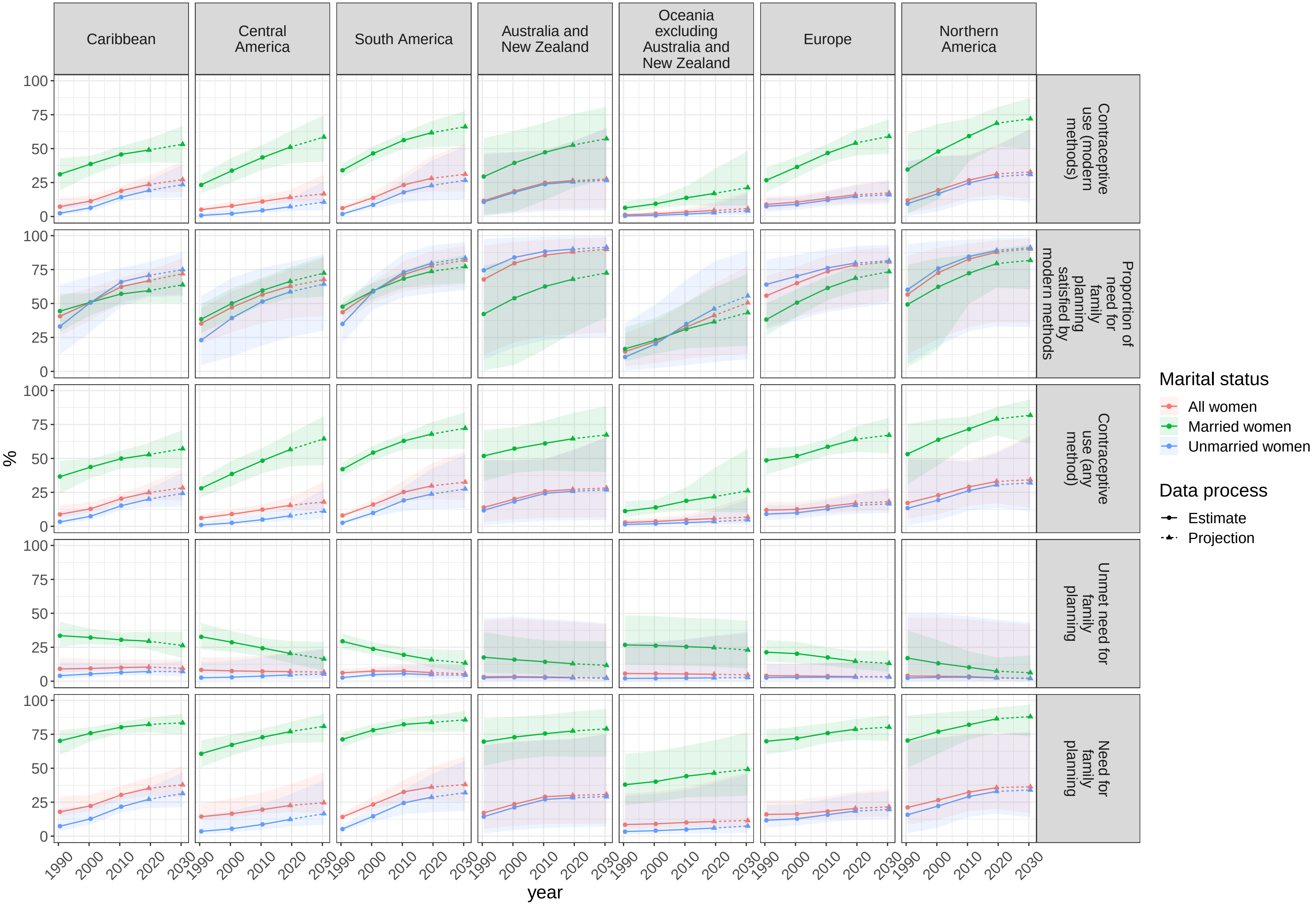

(ii)

Figure B. (cont'd).

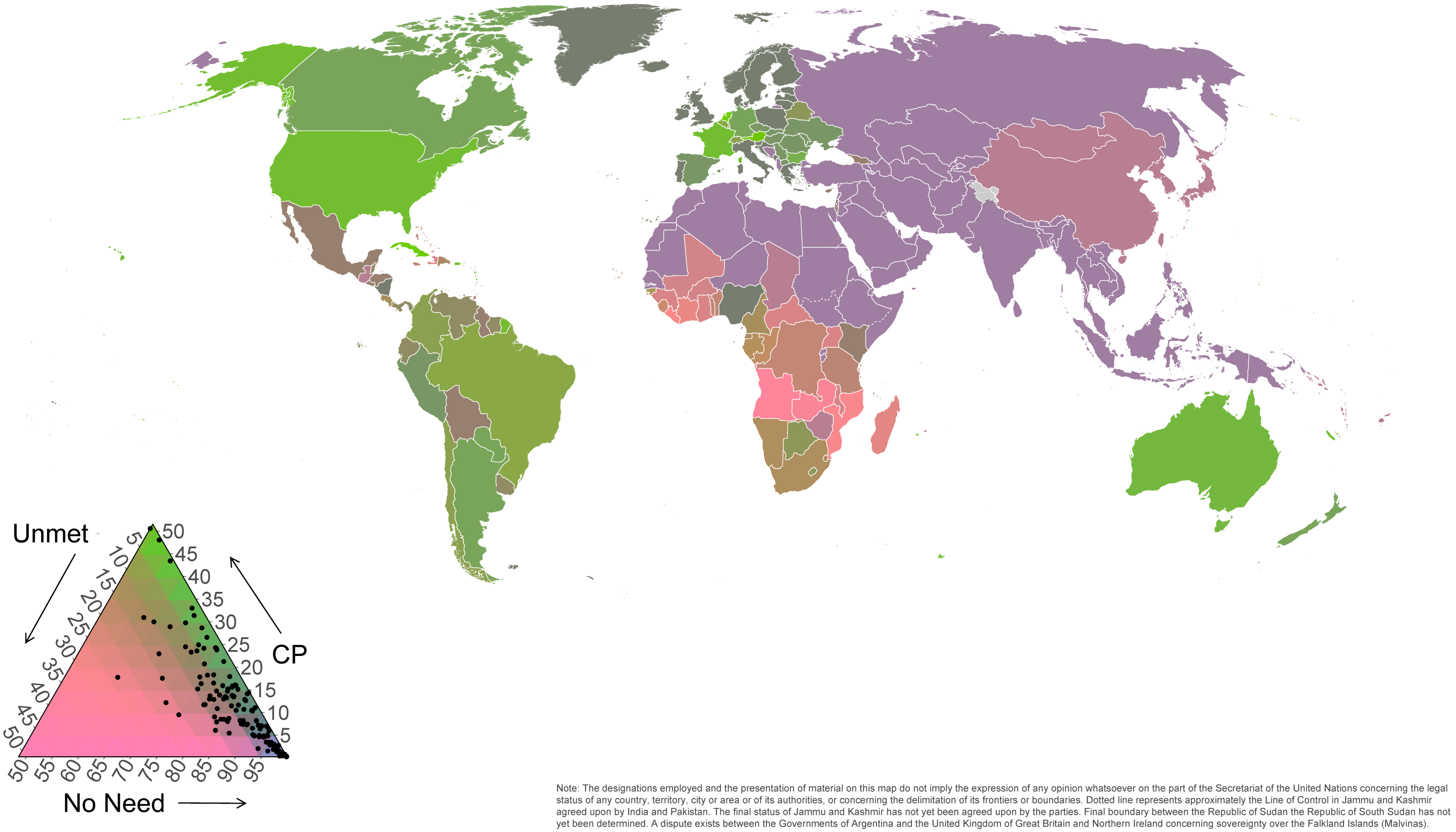

Figure C. Ternary colour scheme map for unmarried adolescent women aged 15–19, with centred colour scheme. The map shows contraceptive use (any method), unmet need for family planning, and no need for family planning for unmarried women aged 15–19 years, 2019. The colour scheme is centred at the compositional mean so that relative differences among countries are highlighted. However, it also means that this map is not directly comparable with the other ternary colour scheme maps. Note that the legend has been cropped.

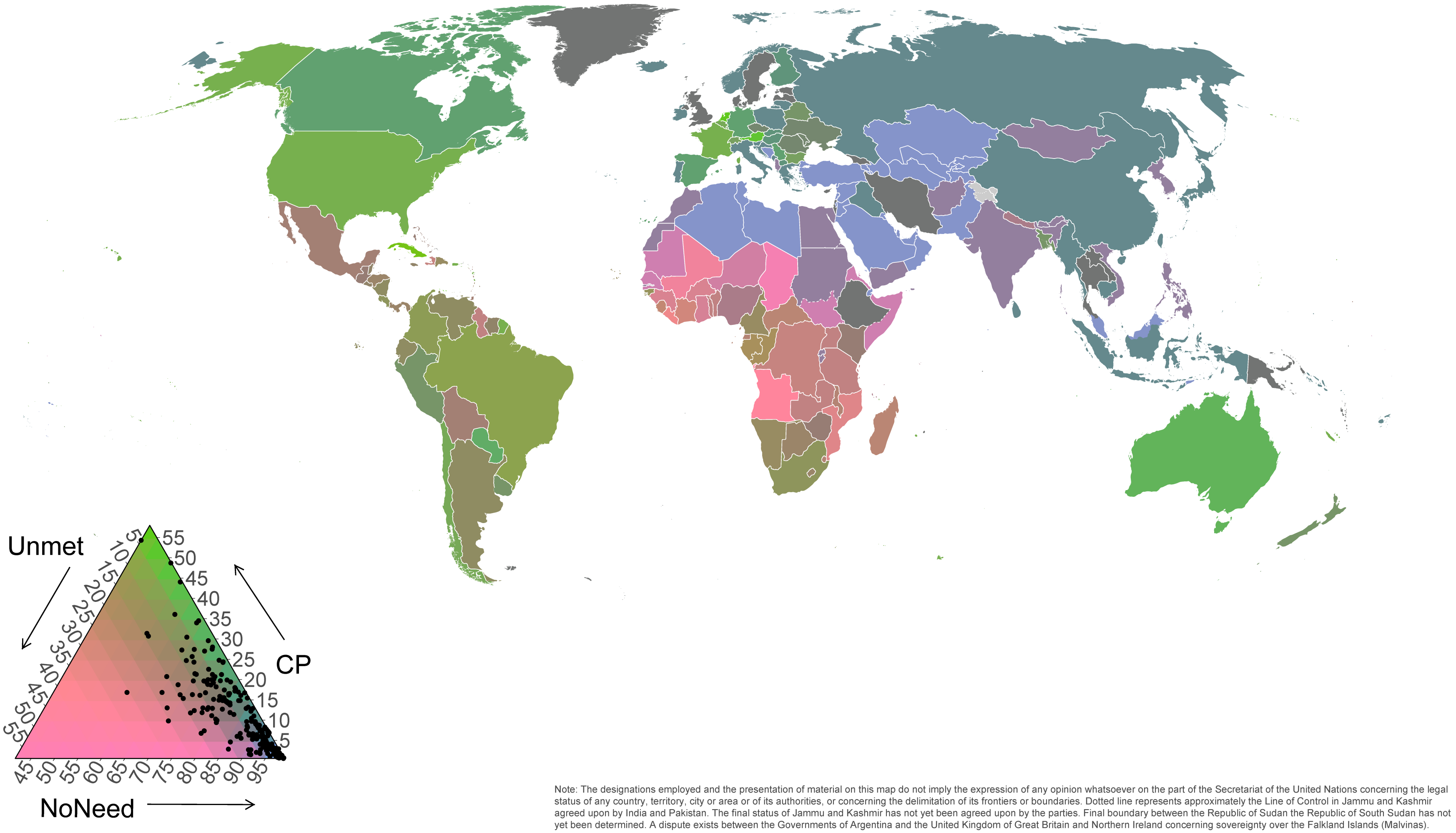

Figure D. Ternary colour scheme map for all adolescent women aged 15–19, with centred colour scheme. The map shows contraceptive use (any method), unmet need for family planning, and no need for family planning for unmarried women aged 15–19 years, 2019. The colour scheme is centred at the compositional mean so that relative differences among countries are highlighted. However, it also means that this map is not directly comparable with the other ternary colour scheme maps. Note that the legend has been cropped.

4 Country-Specific Estimates and Projections of Family-Planning Indicators

These figures contain a systematic and comprehensive set of annual, model-based estimates and projections, and underlying survey-based observations, for a collection of family planning indicators, including contraceptive prevalence, the unmet need for family planning, and the demand for family planning satisfied by use of contraception (any method or modern methods alone). They pertain to the population of adolescent women aged 15–19 years.

The results pertain to adolescent women aged 15–19 years who are unmarried and not in a union ('unmarried'), married or in a union ('married'), and all adolescent women aged 15–19. They cover the period from 1970 to 2030. Estimates based on medians, as well as 80 per cent uncertainty intervals (represented by lines) and 95 per cent uncertainty intervals (represented by shaded areas), are provided for 185 countries or areas. The results are based on data available as of February 2019.

Note: The designations employed and the material presented in this publication do not imply the expression of any opinion whatsoever on the part of the Secretariat of the United Nations concerning the legal status of any country, territory or area or of its authorities, or concerning the delimitation of its frontiers or boundaries. The term "country" as used in this publication also refers, as appropriate, to territories or areas. Countries or areas listed individually are only those with 90,000 inhabitants or more in 2017 and ; the rest are included in the aggregates but are not listed separately.

4.1 Unmarried Adolescent Women

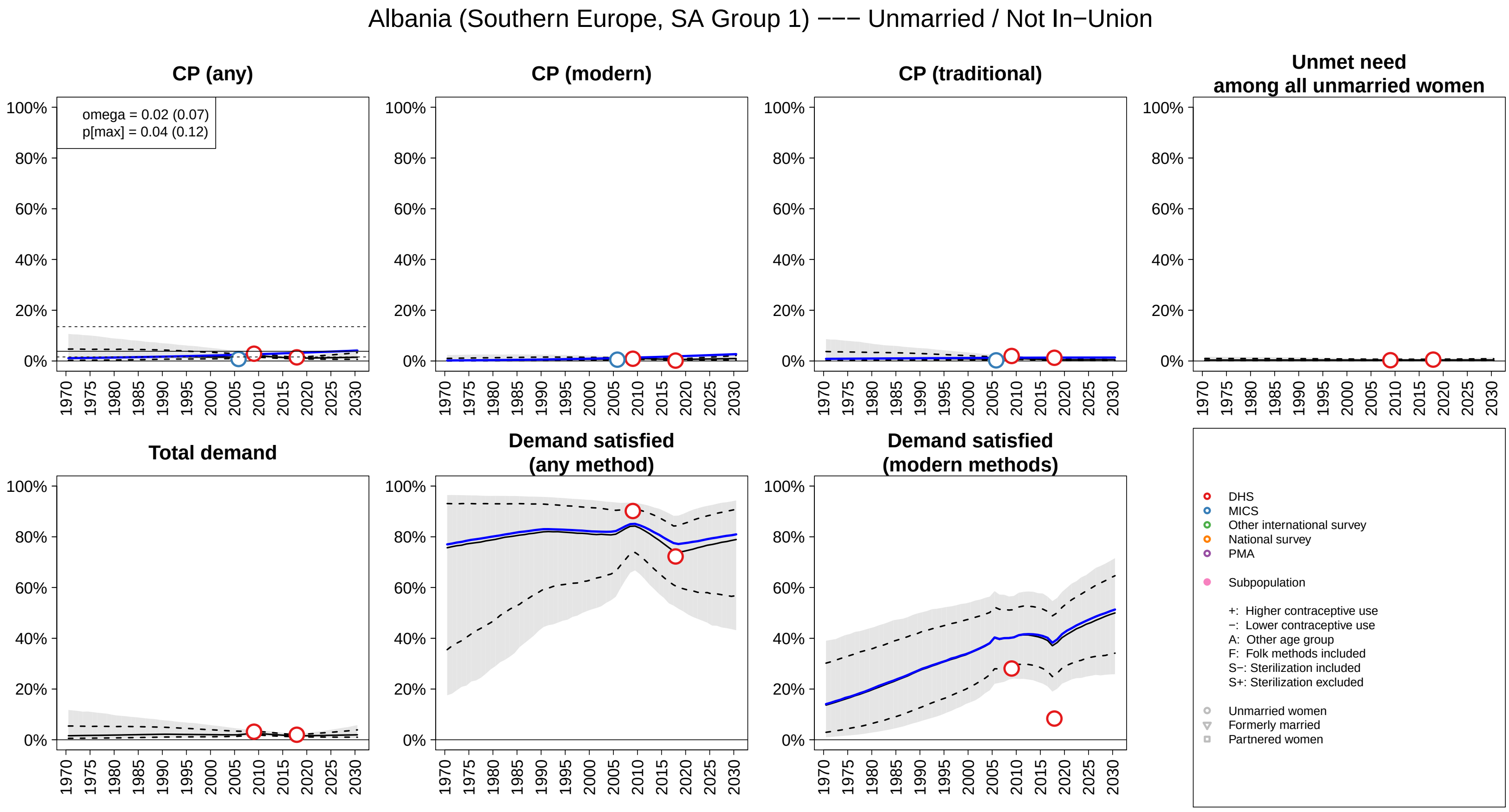
Albania (Southern Europe, SA Group 1) ––– Unmarried / Not In−Union
CP (any)
omega = 0.02 (0.07)
p[max] = 0.04 (0.12)
CP (modern)
CP (traditional)
Unmet need among all unmarried women
Total demand
Demand satisfied (any method)
Demand satisfied (modern methods)
DHS
MICS
Other international survey
National survey
PMA
Subpopulation
+: Higher contraceptive use
−: Lower contraceptive use
A: Other age group
F: Folk methods included
S−: Sterilization included
S+: Sterilization excluded
Unmarried women
Formerly married
Partnered women

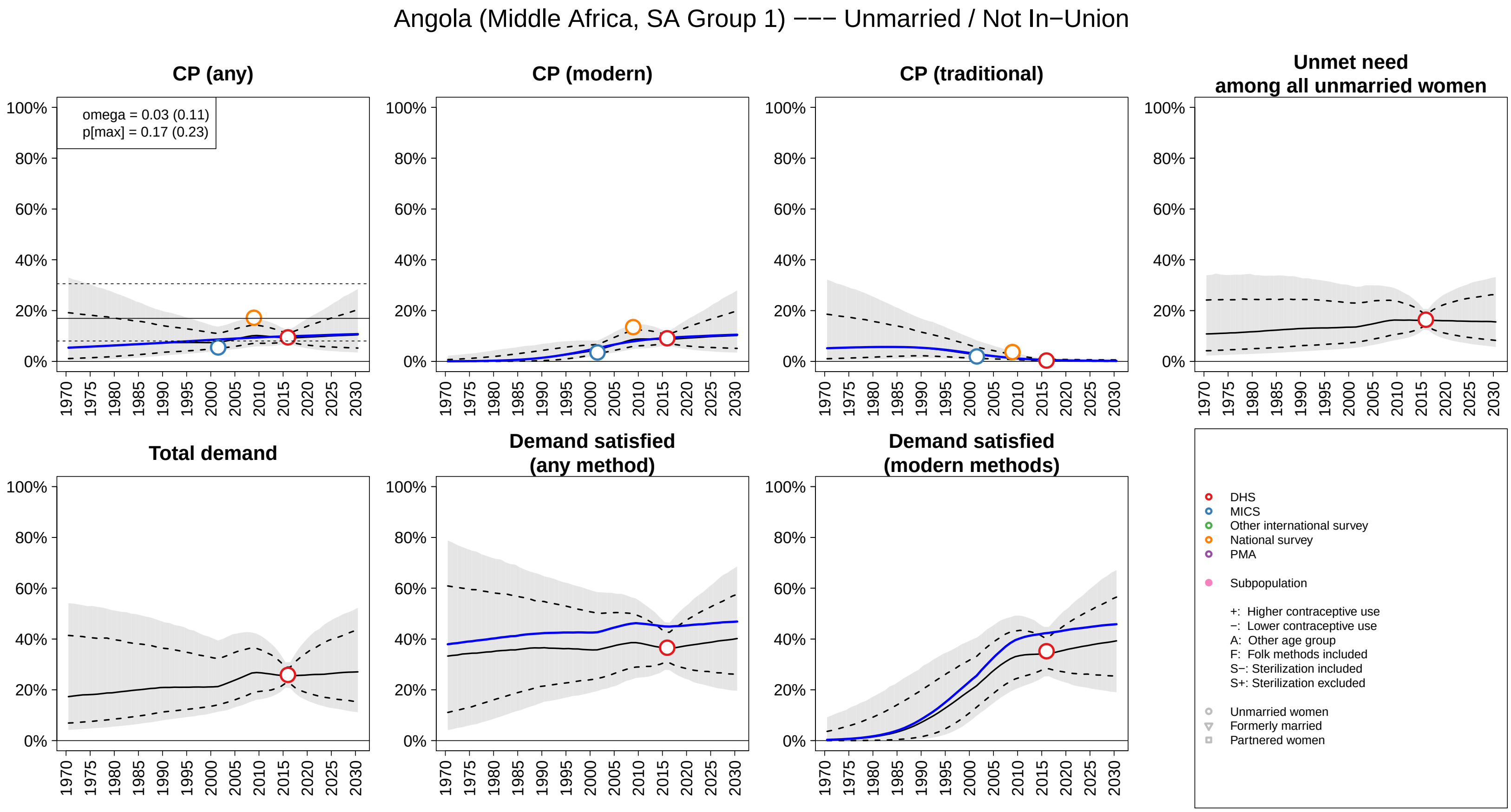
Angola (Middle Africa, SA Group 1) ––– Unmarried / Not In−Union
CP (any)
omega = 0.03 (0.11)
p[max] = 0.17 (0.23)
CP (modern)
CP (traditional)
Unmet need among all unmarried women
Total demand
Demand satisfied (any method)
Demand satisfied (modern methods)
DHS
MICS
Other international survey
National survey
PMA
Subpopulation
+: Higher contraceptive use
−: Lower contraceptive use
A: Other age group
F: Folk methods included
S−: Sterilization included
S+: Sterilization excluded
Unmarried women
Formerly married
Partnered women

Armenia (Western Asia, SA Group 0) −−− Unmarried / Not In−Union

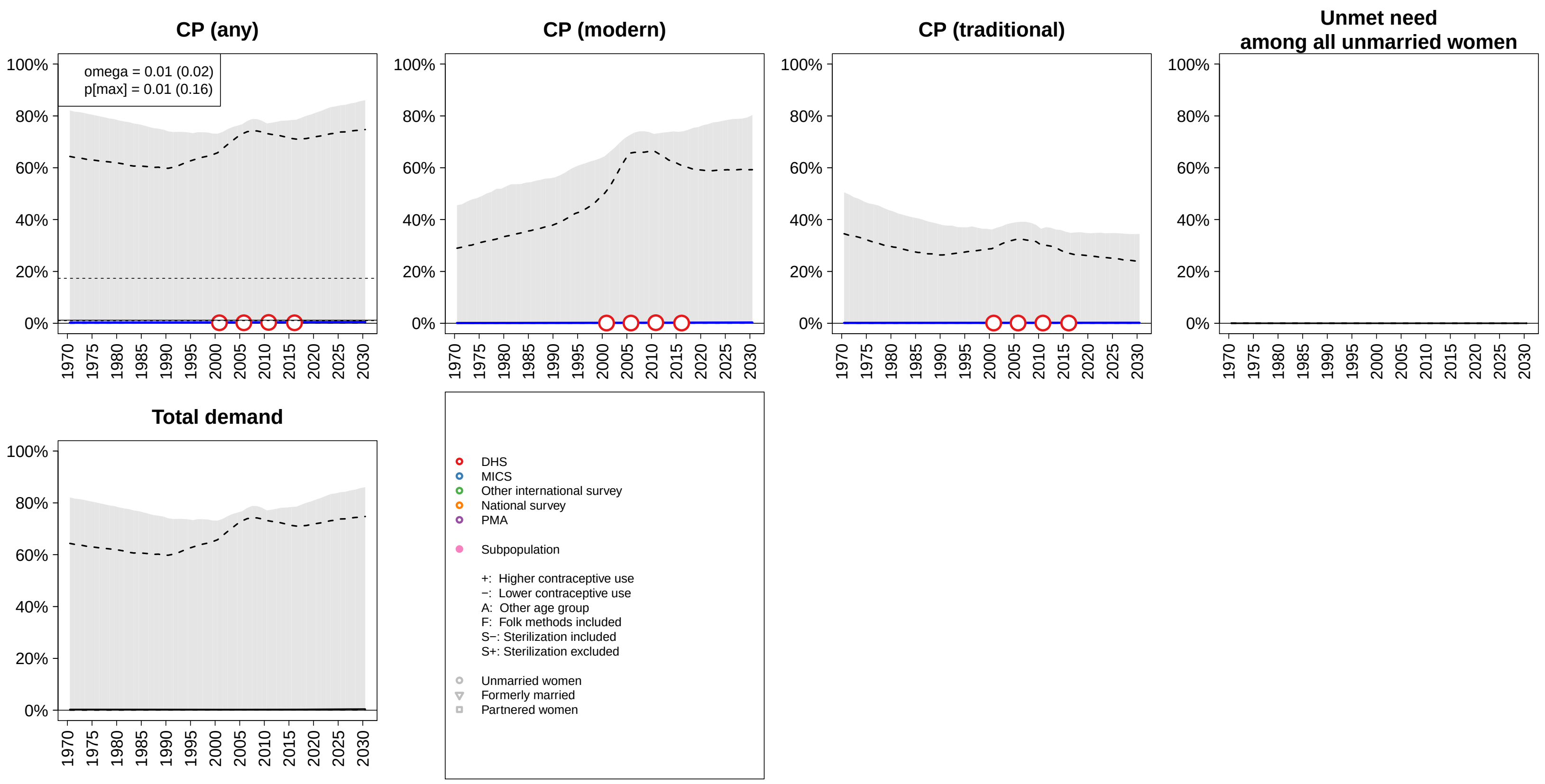

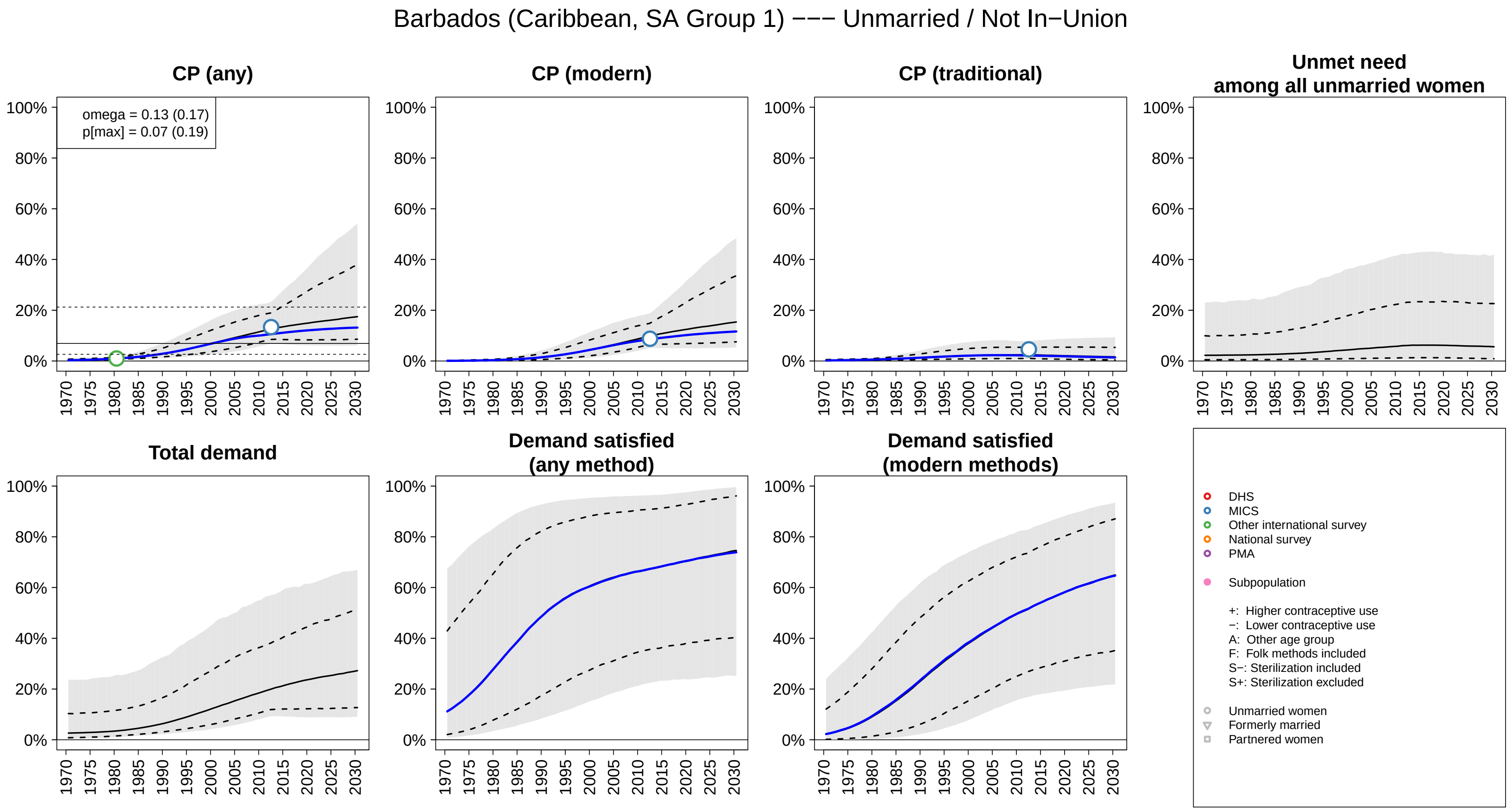
Barbados (Caribbean, SA Group 1) ––– Unmarried / Not In–Union
CP (any)
omega = 0.13 (0.17)
p[max] = 0.07 (0.19)
CP (modern)
CP (traditional)
Unmet need among all unmarried women
Total demand
Demand satisfied (any method)
Demand satisfied (modern methods)
DHS
MICS
Other international survey
National survey
PMA
Subpopulation
+: Higher contraceptive use
−: Lower contraceptive use
A: Other age group
F: Folk methods included
S−: Sterilization included
S+: Sterilization excluded
Unmarried women
Formerly married
Partnered women

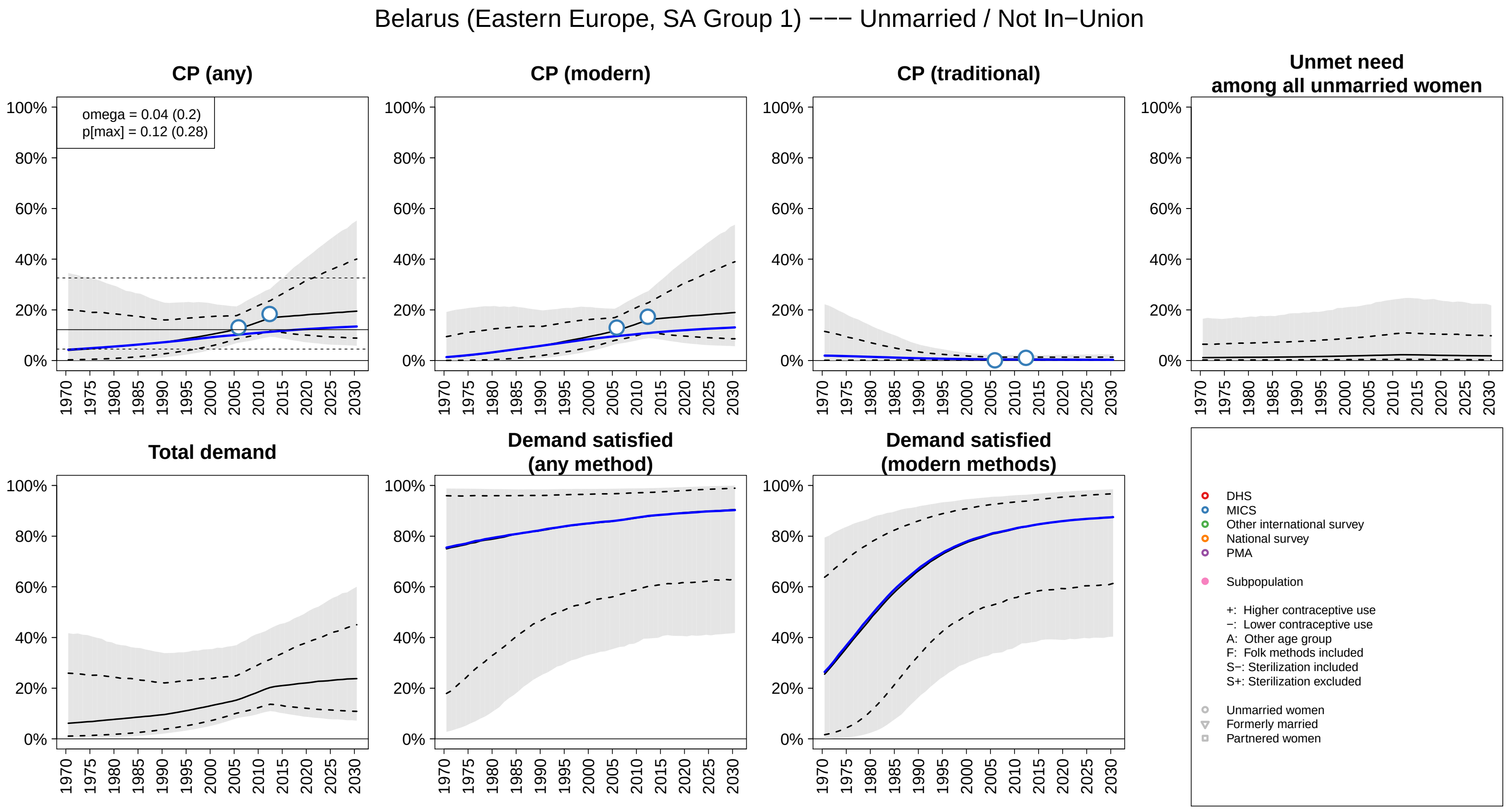
Belarus (Eastern Europe, SA Group 1) --- Unmarried / Not In-Union
CP (any)
omega = 0.04 (0.2)
p[max] = 0.12 (0.28)
CP (modern)
CP (traditional)
Unmet need
among all unmarried women
Total demand
Demand satisfied
(any method)
Demand satisfied
(modern methods)
DHS
MICS
Other international survey
National survey
PMA
Subpopulation
+: Higher contraceptive use
−: Lower contraceptive use
A: Other age group
F: Folk methods included
S−: Sterilization included
S+: Sterilization excluded
Unmarried women
Formerly married
Partnered women

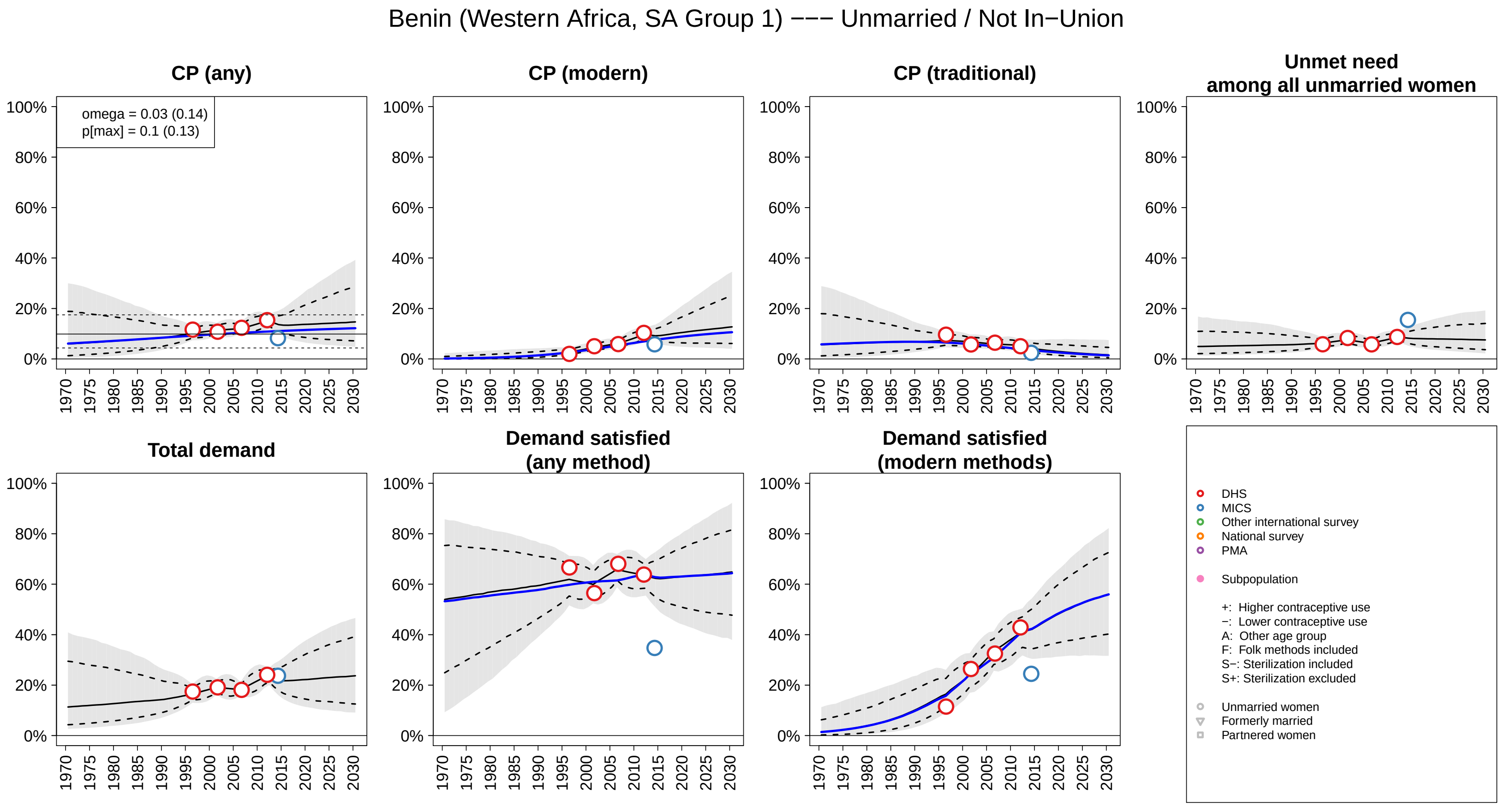
Benin (Western Africa, SA Group 1) −−− Unmarried / Not In−Union
CP (any)
omega = 0.03 (0.14)
p[max] = 0.1 (0.13)
CP (modern)
CP (traditional)
Unmet need among all unmarried women
Total demand
Demand satisfied (any method)
Demand satisfied (modern methods)
DHS
MICS
Other international survey
National survey
PMA
Subpopulation
+: Higher contraceptive use
−: Lower contraceptive use
A: Other age group
F: Folk methods included
S−: Sterilization included
S+: Sterilization excluded
Unmarried women
Formerly married
Partnered women

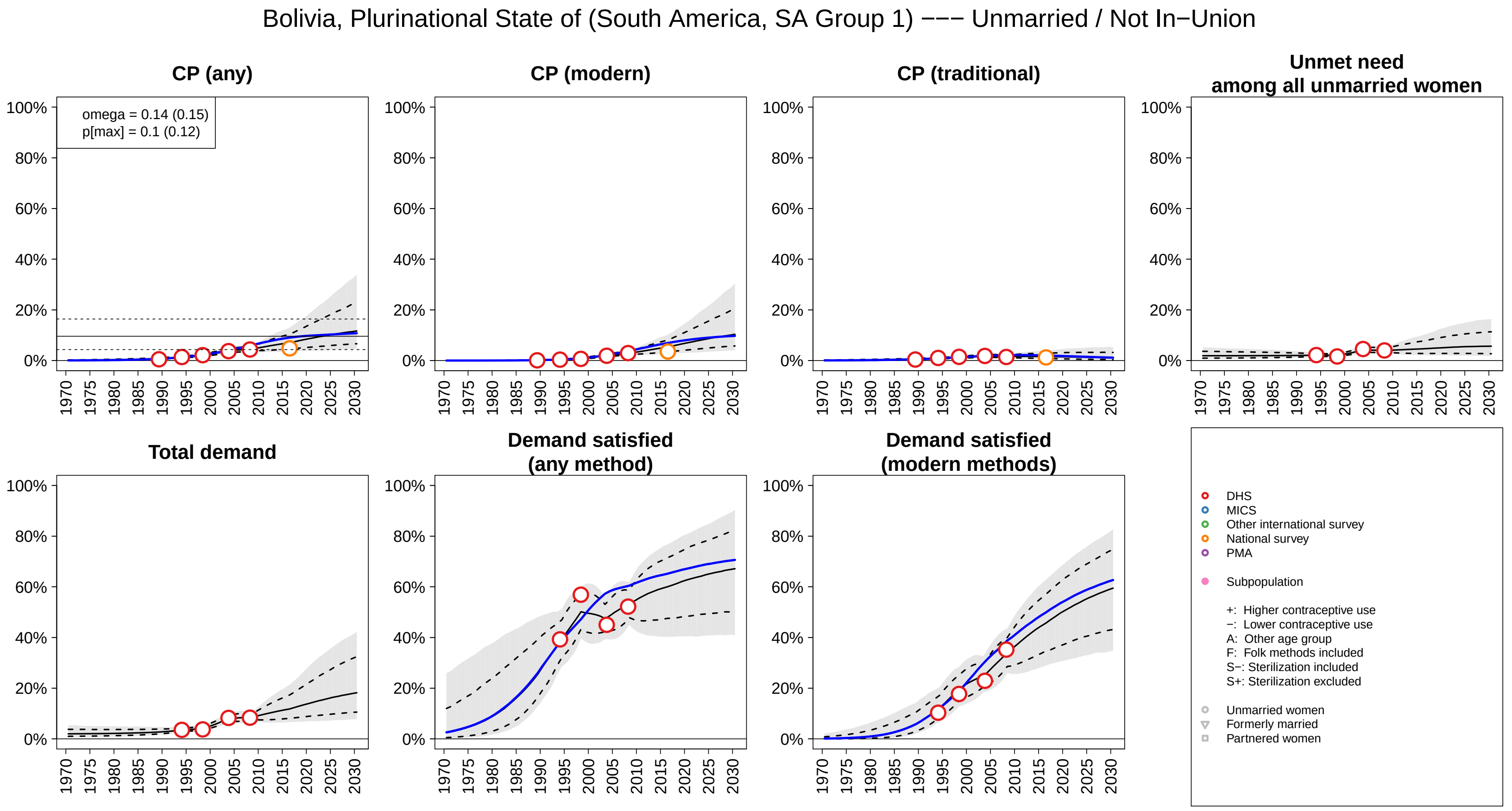

Bolivia, Plurinational State of (South America, SA Group 1) ––– Unmarried / Not In−Union
CP (any)
omega = 0.14 (0.15)
p[max] = 0.1 (0.12)
CP (modern)
CP (traditional)
Unmet need among all unmarried women
Total demand
Demand satisfied (any method)
Demand satisfied (modern methods)
DHS
MICS
Other international survey
National survey
PMA
Subpopulation
+: Higher contraceptive use
−: Lower contraceptive use
A: Other age group
F: Folk methods included
S−: Sterilization included
S+: Sterilization excluded
Unmarried women
Formerly married
Partnered women

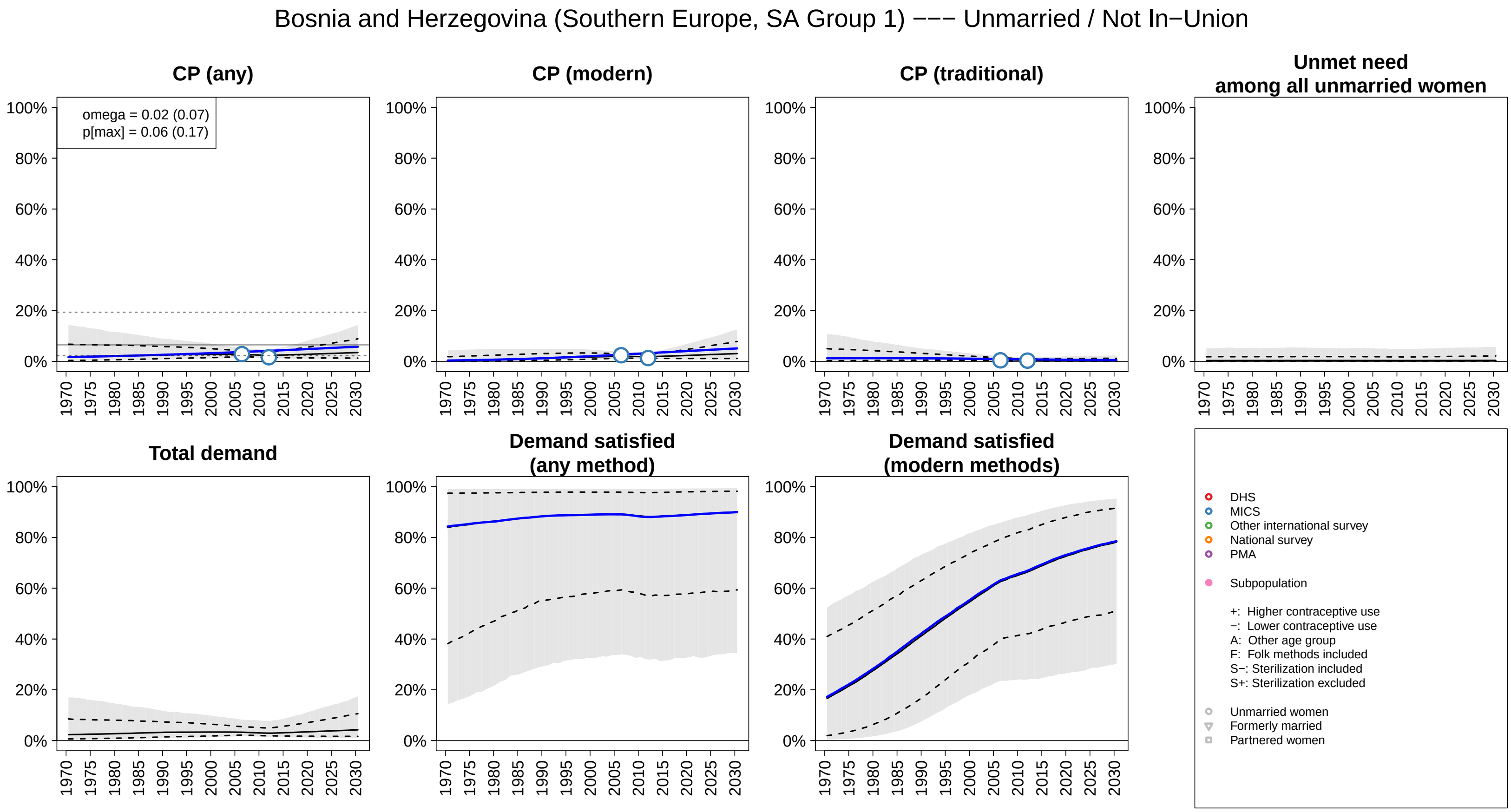
Bosnia and Herzegovina (Southern Europe, SA Group 1) −−− Unmarried / Not In−Union
CP (any)
omega = 0.02 (0.07)
p[max] = 0.06 (0.17)
CP (modern)
CP (traditional)
Unmet need among all unmarried women
Total demand
Demand satisfied (any method)
Demand satisfied (modern methods)
DHS
MICS
Other international survey
National survey
PMA
Subpopulation
+: Higher contraceptive use
−: Lower contraceptive use
A: Other age group
F: Folk methods included
S−: Sterilization included
S+: Sterilization excluded
Unmarried women
Formerly married
Partnered women

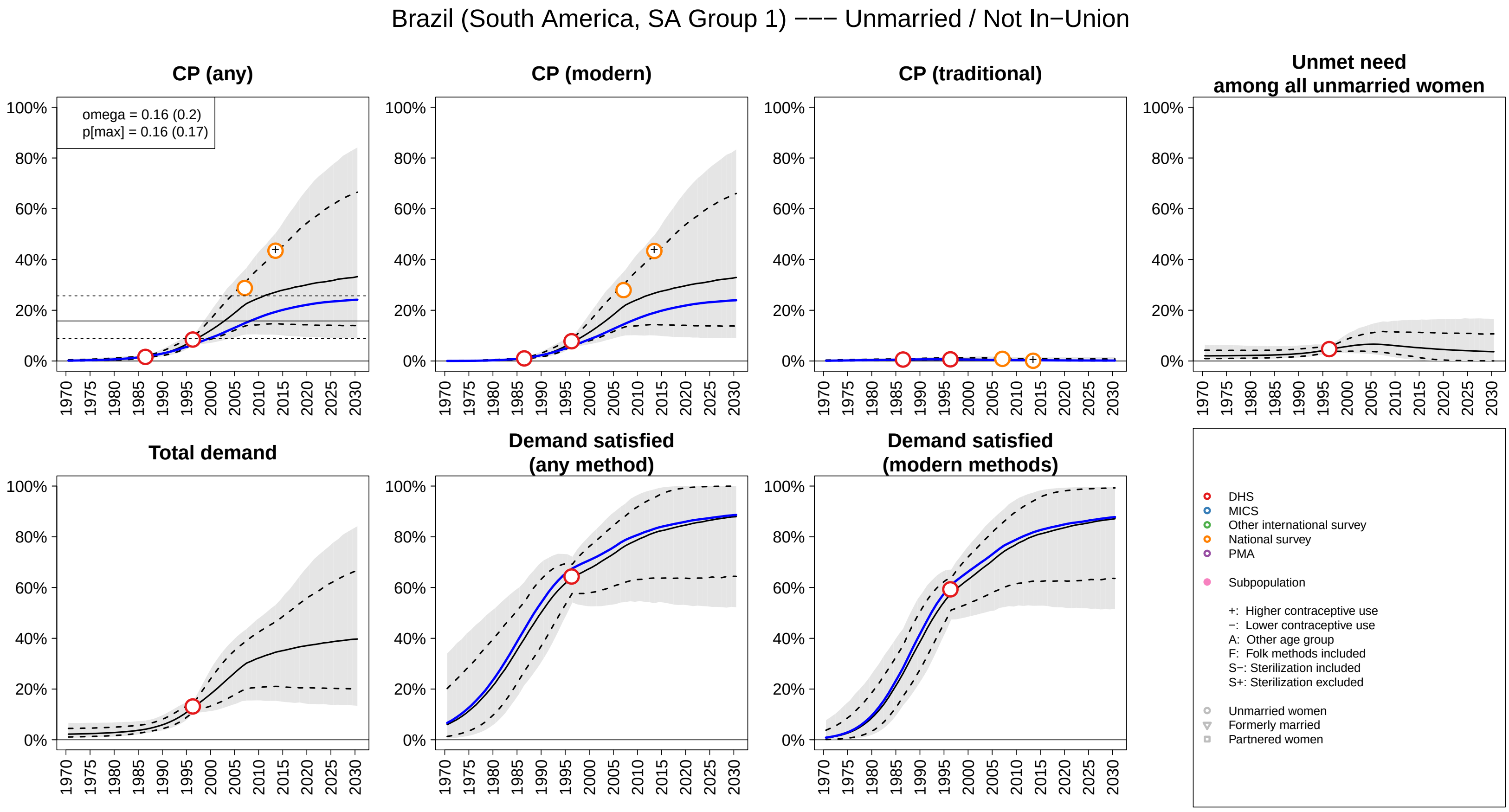
Brazil (South America, SA Group 1) −−− Unmarried / Not In−Union
CP (any)
omega = 0.16 (0.2)
p[max] = 0.16 (0.17)
CP (modern)
CP (traditional)
Unmet need
among all unmarried women
Total demand
Demand satisfied
(any method)
Demand satisfied
(modern methods)
DHS
MICS
Other international survey
National survey
PMA
Subpopulation
+: Higher contraceptive use
−: Lower contraceptive use
A: Other age group
F: Folk methods included
S−: Sterilization included
S+: Sterilization excluded
Unmarried women
Formerly married
Partnered women

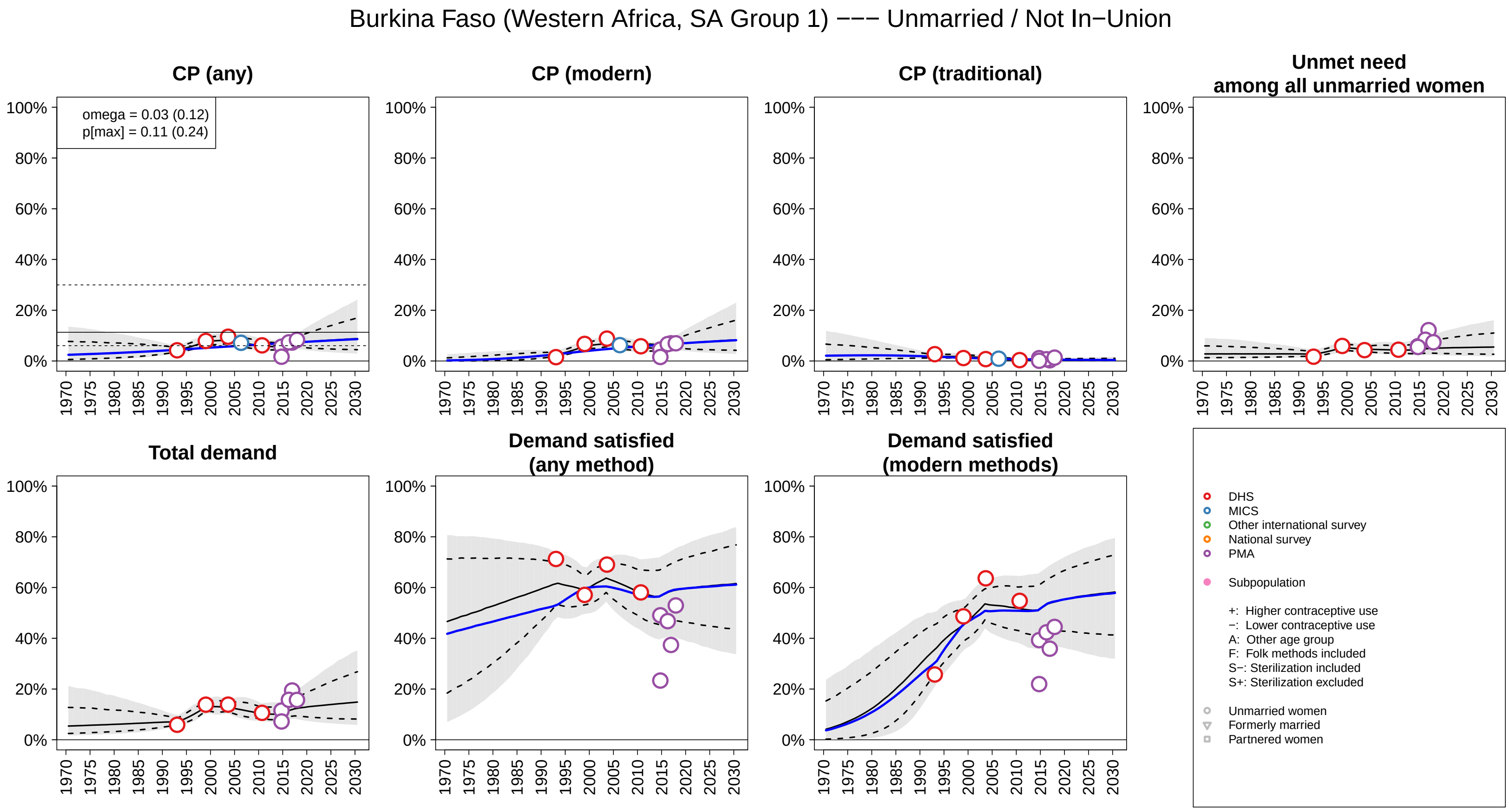
Burkina Faso (Western Africa, SA Group 1) −−− Unmarried / Not In−Union
CP (any)
omega = 0.03 (0.12)
p[max] = 0.11 (0.24)
CP (modern)
CP (traditional)
Unmet need among all unmarried women
Total demand
Demand satisfied (any method)
Demand satisfied (modern methods)
DHS
MICS
Other international survey
National survey
PMA
Subpopulation
+: Higher contraceptive use
−: Lower contraceptive use
A: Other age group
F: Folk methods included
S−: Sterilization included
S+: Sterilization excluded
Unmarried women
Formerly married
Partnered women

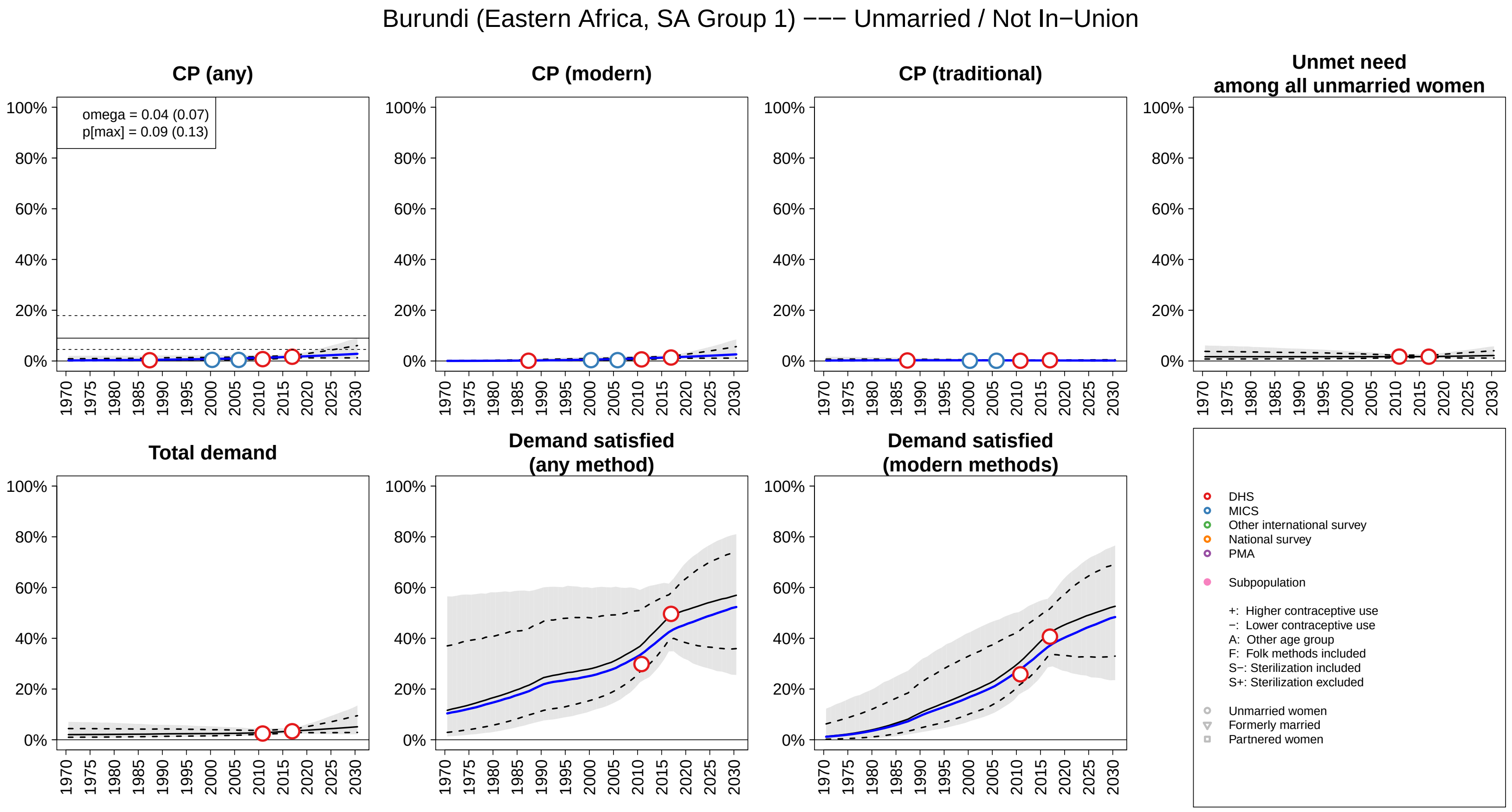
Burundi (Eastern Africa, SA Group 1) --- Unmarried / Not In−Union
CP (any)
omega = 0.04 (0.07)
p[max] = 0.09 (0.13)
CP (modern)
CP (traditional)
Unmet need among all unmarried women
Total demand
Demand satisfied (any method)
Demand satisfied (modern methods)
DHS
MICS
Other international survey
National survey
PMA
Subpopulation
+: Higher contraceptive use
−: Lower contraceptive use
A: Other age group
F: Folk methods included
S−: Sterilization included
S+: Sterilization excluded
Unmarried women
Formerly married
Partnered women

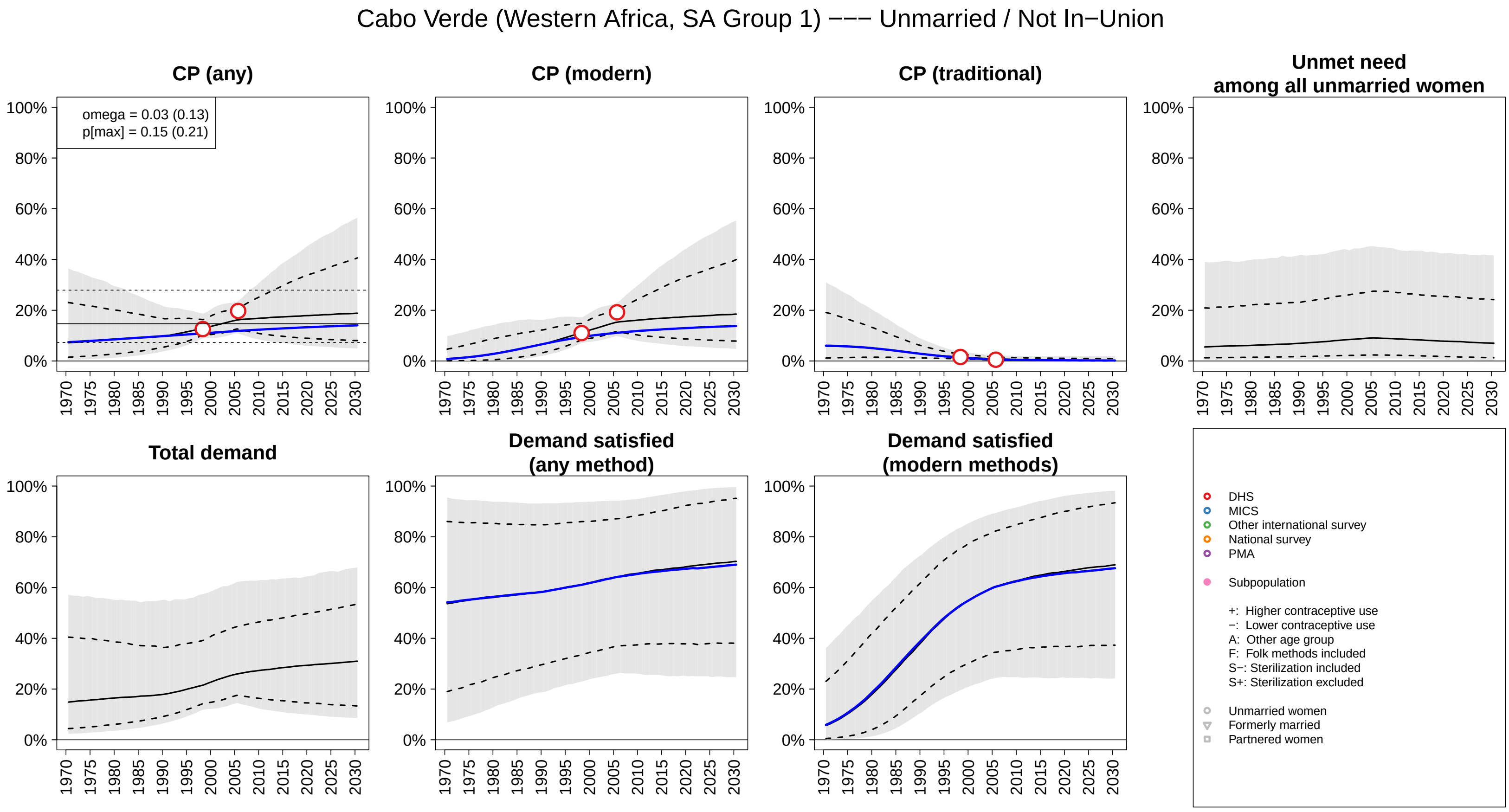
Cabo Verde (Western Africa, SA Group 1) −−− Unmarried / Not In−Union
CP (any)
omega = 0.03 (0.13)
p[max] = 0.15 (0.21)
CP (modern)
CP (traditional)
Unmet need among all unmarried women
Total demand
Demand satisfied (any method)
Demand satisfied (modern methods)
DHS
MICS
Other international survey
National survey
PMA
Subpopulation
+: Higher contraceptive use
−: Lower contraceptive use
A: Other age group
F: Folk methods included
S−: Sterilization included
S+: Sterilization excluded
Unmarried women
Formerly married
Partnered women

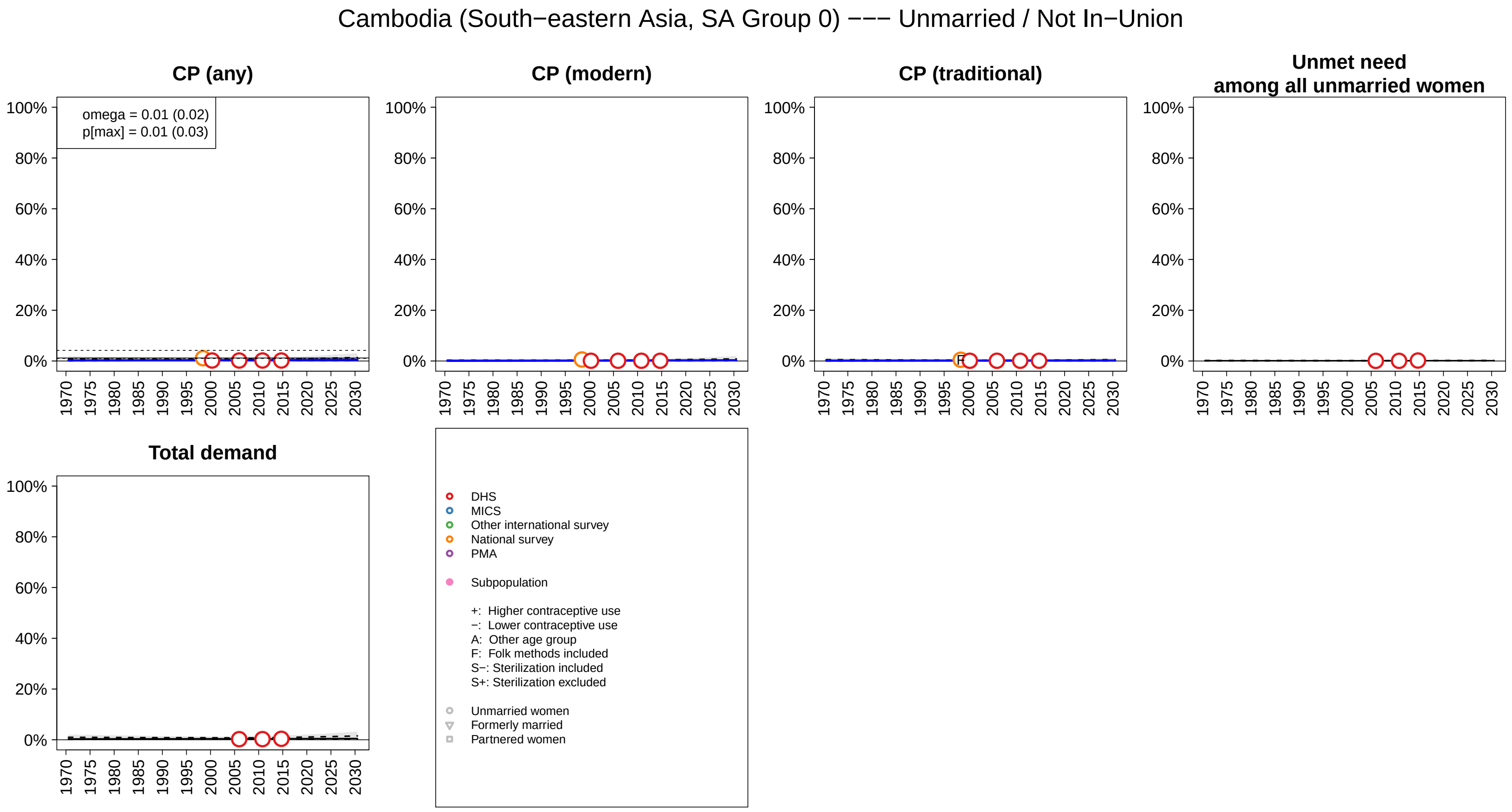
Cambodia (South−eastern Asia, SA Group 0) −−− Unmarried / Not In−Union
CP (any)
omega = 0.01 (0.02)
p[max] = 0.01 (0.03)
CP (modern)
CP (traditional)
Unmet need
among all unmarried women
Total demand
DHS
MICS
Other international survey
National survey
PMA
Subpopulation
+: Higher contraceptive use
−: Lower contraceptive use
A: Other age group
F: Folk methods included
S−: Sterilization included
S+: Sterilization excluded
Unmarried women
Formerly married
Partnered women

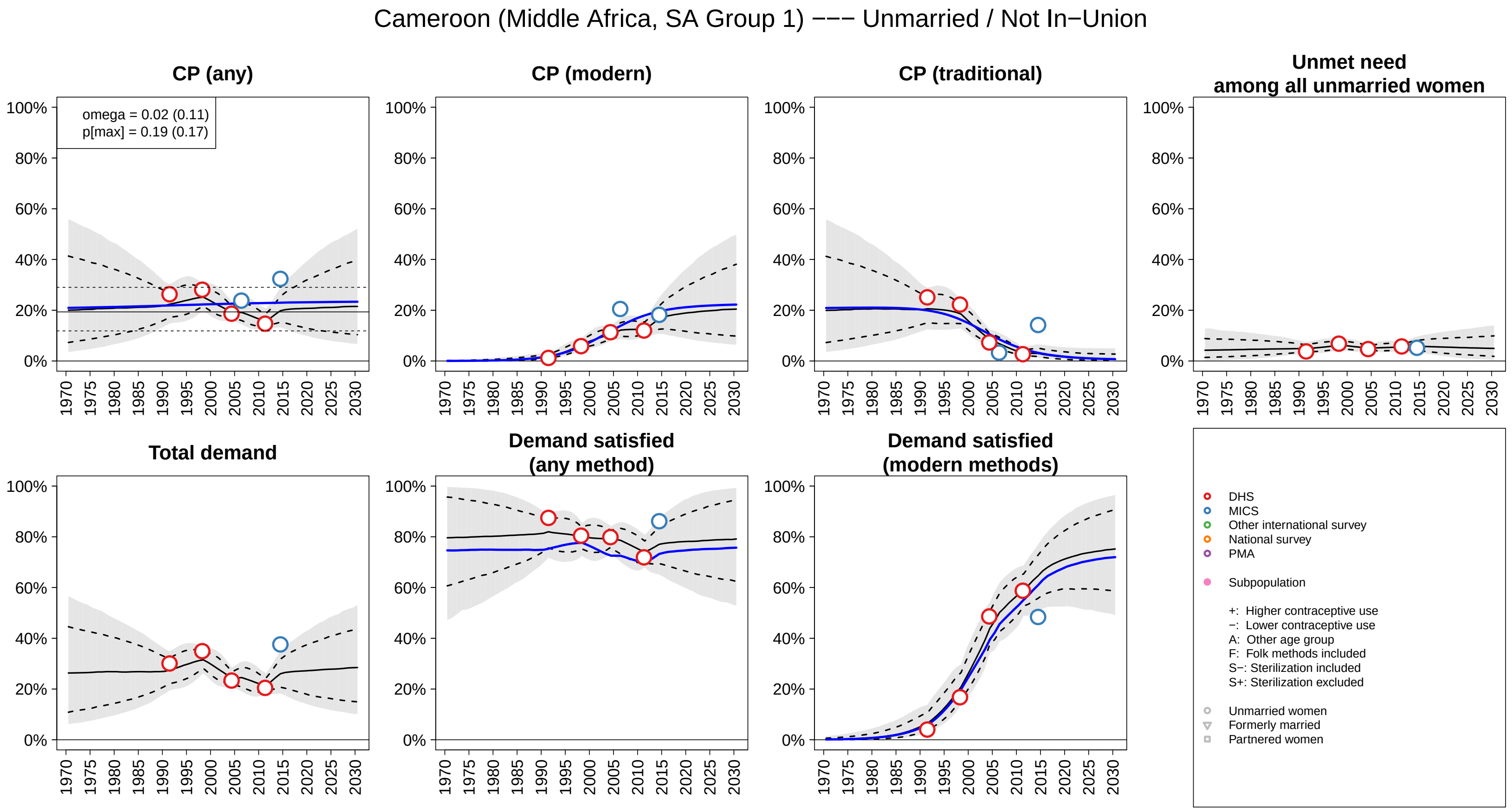
Cameroon (Middle Africa, SA Group 1) −−− Unmarried / Not In−Union
CP (any)
omega = 0.02 (0.11)
p[max] = 0.19 (0.17)
CP (modern)
CP (traditional)
Unmet need among all unmarried women
Total demand
Demand satisfied (any method)
Demand satisfied (modern methods)
DHS
MICS
Other international survey
National survey
PMA
Subpopulation
+: Higher contraceptive use
−: Lower contraceptive use
A: Other age group
F: Folk methods included
S−: Sterilization included
S+: Sterilization excluded
Unmarried women
Formerly married
Partnered women

Central African Republic (Middle Africa, SA Group 1) −−− Unmarried / Not In−Union

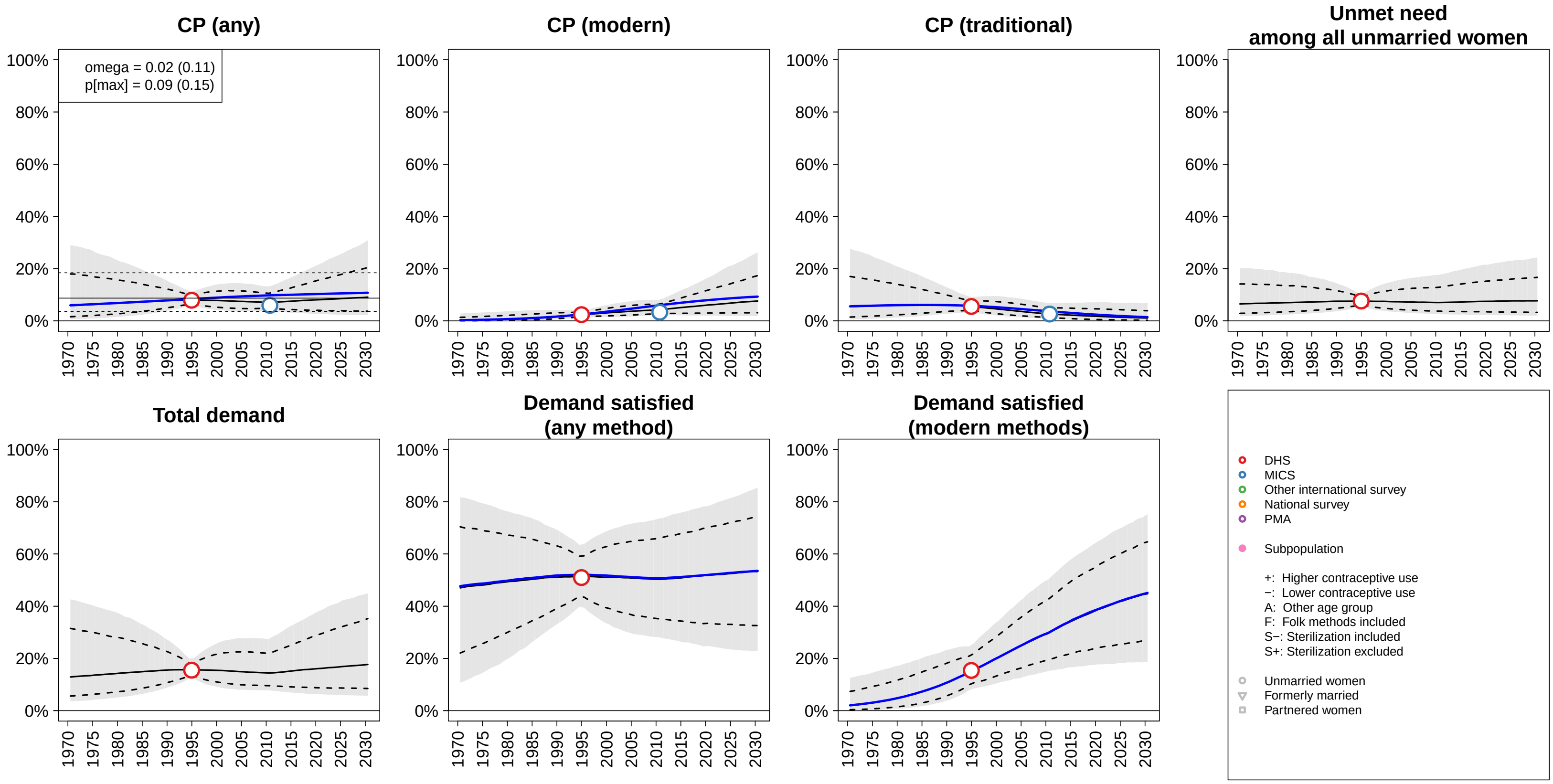

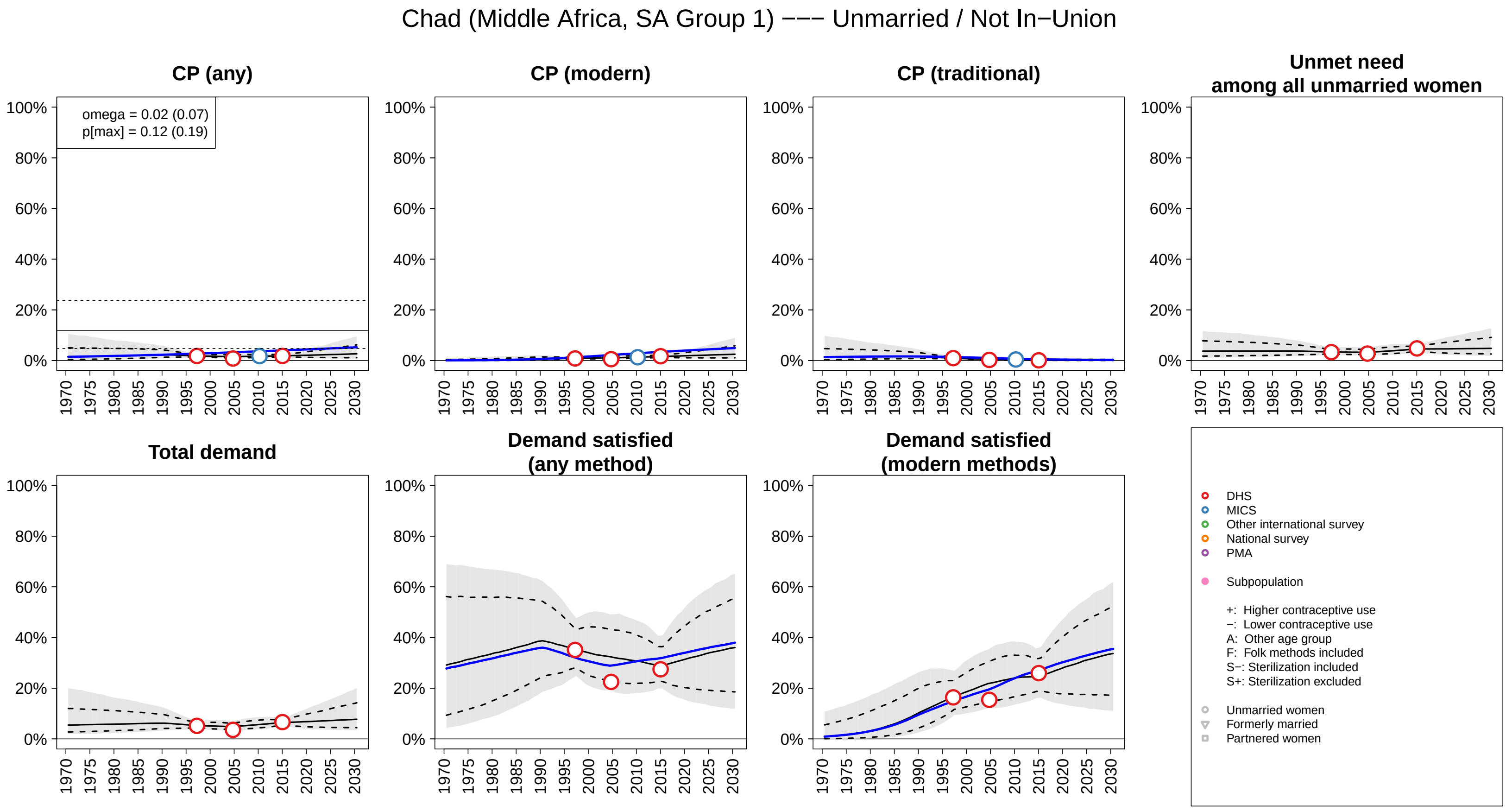
Chad (Middle Africa, SA Group 1) ——— Unmarried / Not In−Union
CP (any)
omega = 0.02 (0.07)
p[max] = 0.12 (0.19)
CP (modern)
CP (traditional)
Unmet need among all unmarried women
Total demand
Demand satisfied (any method)
Demand satisfied (modern methods)
DHS
MICS
Other international survey
National survey
PMA
Subpopulation
+: Higher contraceptive use
−: Lower contraceptive use
A: Other age group
F: Folk methods included
S−: Sterilization included
S+: Sterilization excluded
Unmarried women
Formerly married
Partnered women

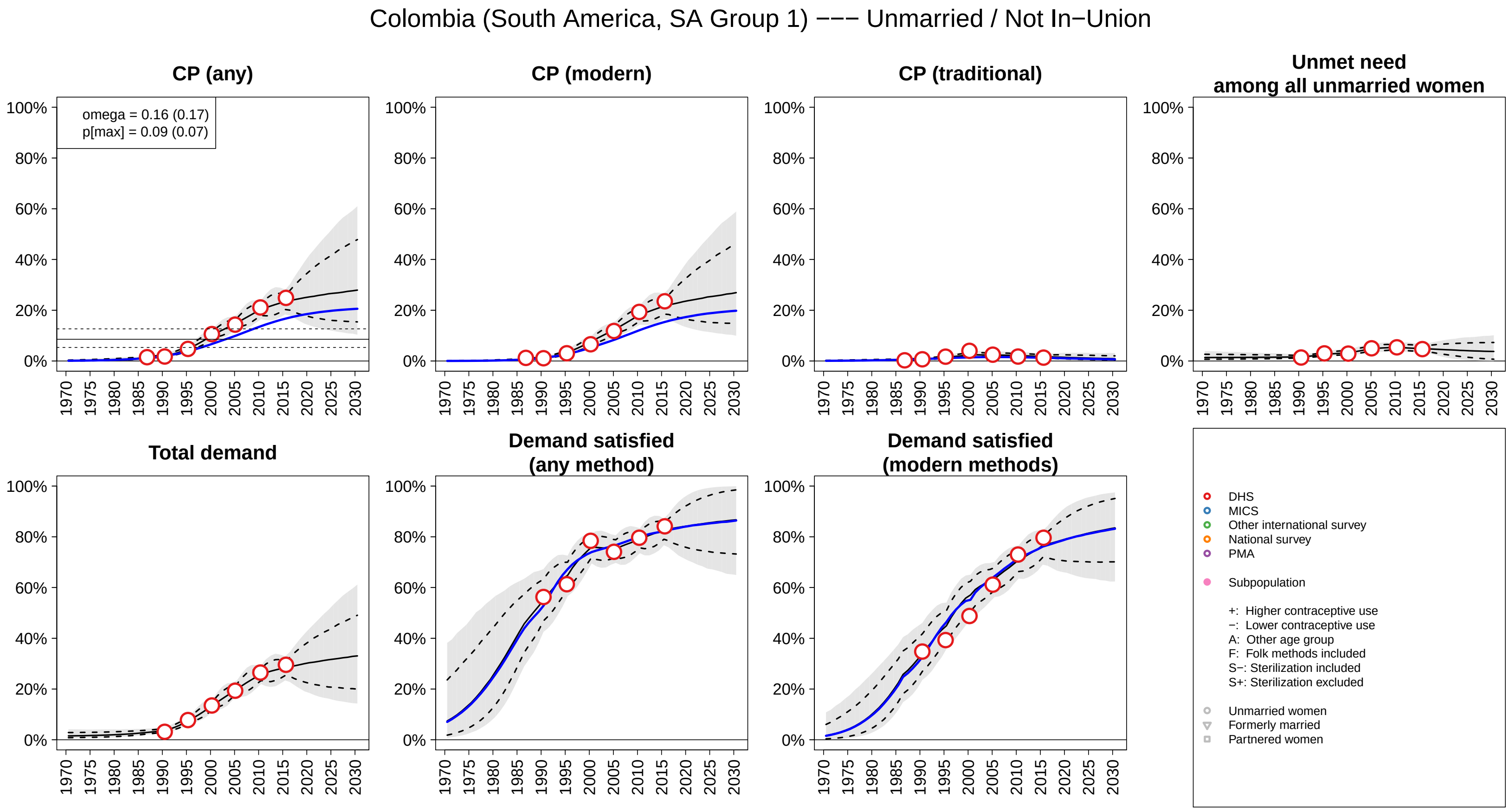
Colombia (South America, SA Group 1) −−− Unmarried / Not In−Union
CP (any)
omega = 0.16 (0.17)
p[max] = 0.09 (0.07)
CP (modern)
CP (traditional)
Unmet need among all unmarried women
Total demand
Demand satisfied (any method)
Demand satisfied (modern methods)
DHS
MICS
Other international survey
National survey
PMA
Subpopulation
+: Higher contraceptive use
−: Lower contraceptive use
A: Other age group
F: Folk methods included
S−: Sterilization included
S+: Sterilization excluded
Unmarried women
Formerly married
Partnered women

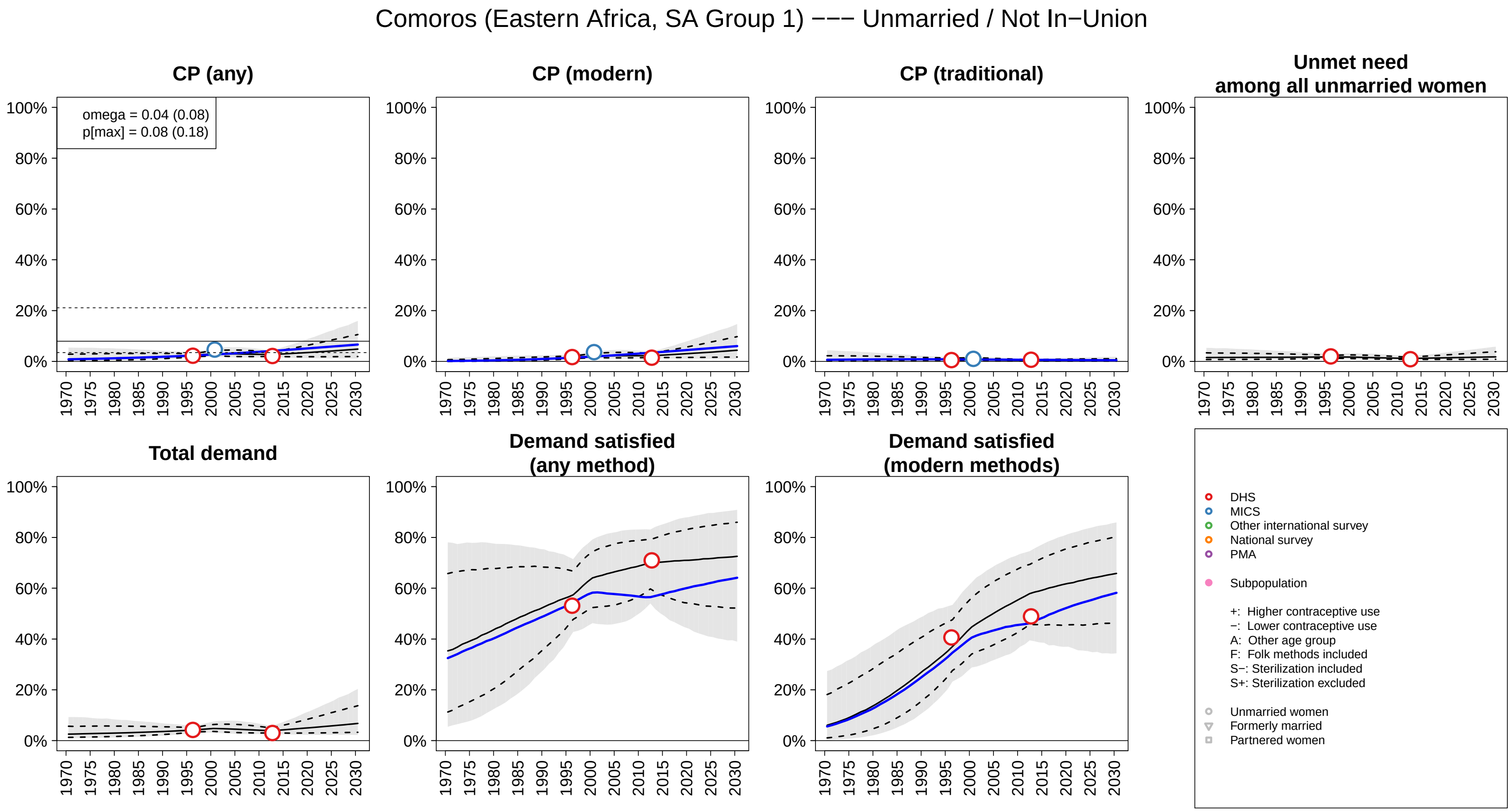
Comoros (Eastern Africa, SA Group 1) −−− Unmarried / Not In−Union
CP (any)
omega = 0.04 (0.08)
p[max] = 0.08 (0.18)
CP (modern)
CP (traditional)
Unmet need among all unmarried women
Total demand
Demand satisfied (any method)
Demand satisfied (modern methods)
DHS
MICS
Other international survey
National survey
PMA
Subpopulation
+: Higher contraceptive use
−: Lower contraceptive use
A: Other age group
F: Folk methods included
S−: Sterilization included
S+: Sterilization excluded
Unmarried women
Formerly married
Partnered women

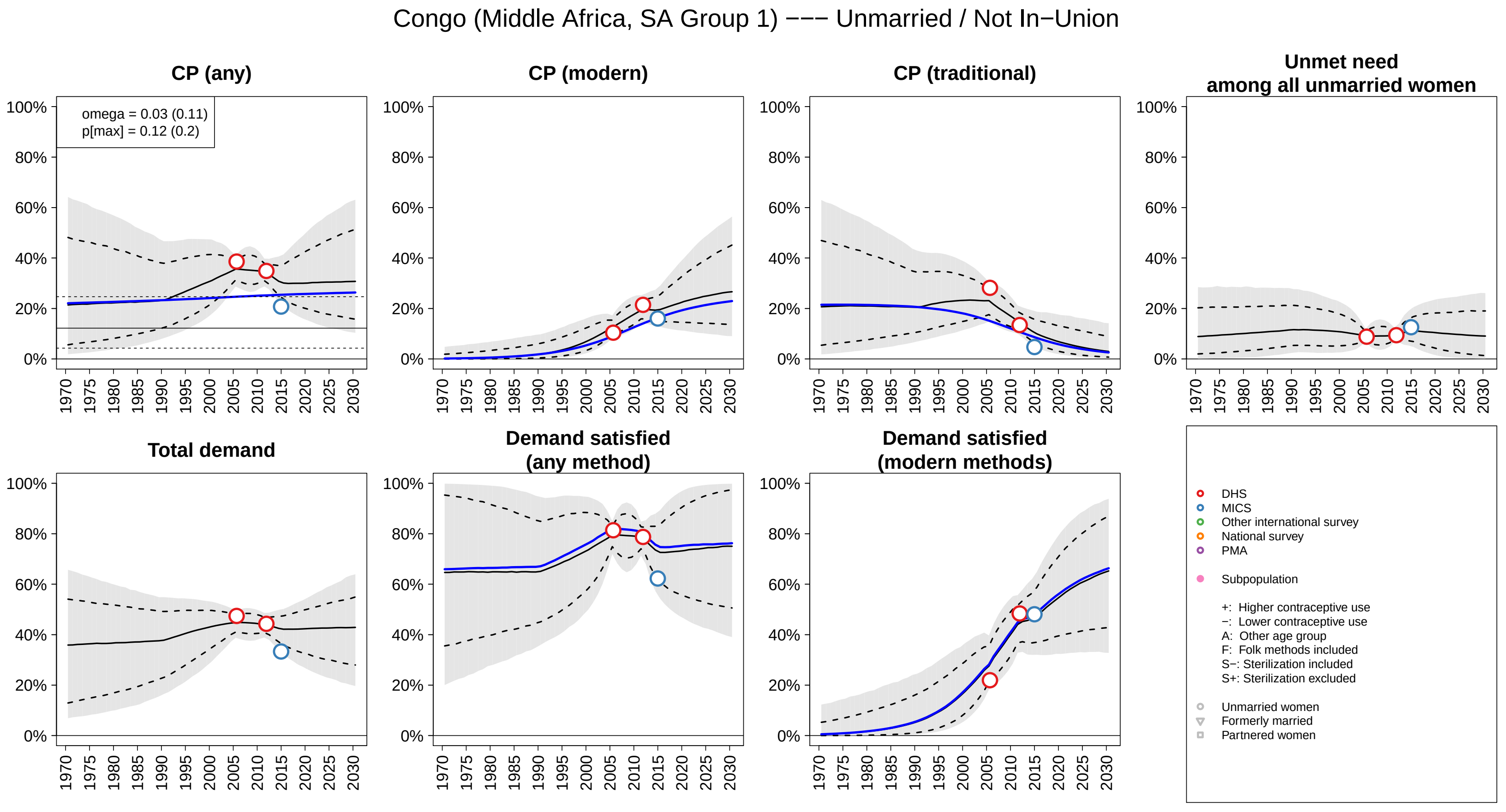
Congo (Middle Africa, SA Group 1) −−− Unmarried / Not In−Union
CP (any)
omega = 0.03 (0.11)
p[max] = 0.12 (0.2)
CP (modern)
CP (traditional)
Unmet need
among all unmarried women
Total demand
Demand satisfied
(any method)
Demand satisfied
(modern methods)
DHS
MICS
Other international survey
National survey
PMA
Subpopulation
+: Higher contraceptive use
−: Lower contraceptive use
A: Other age group
F: Folk methods included
S−: Sterilization included
S+: Sterilization excluded
Unmarried women
Formerly married
Partnered women

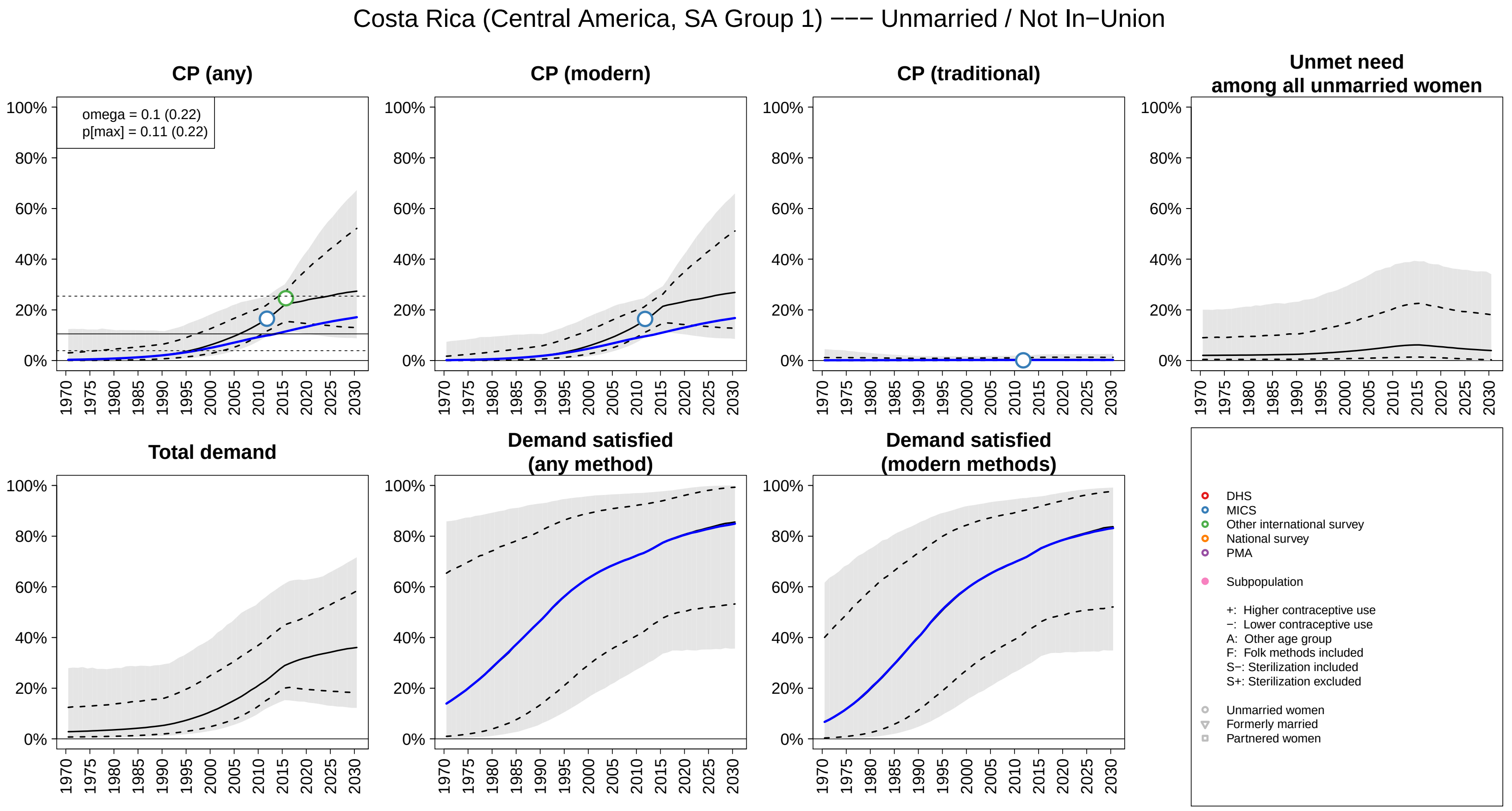
Costa Rica (Central America, SA Group 1) −−− Unmarried / Not In−Union
CP (any)
omega = 0.1 (0.22)
p[max] = 0.11 (0.22)
CP (modern)
CP (traditional)
Unmet need among all unmarried women
Total demand
Demand satisfied (any method)
Demand satisfied (modern methods)
DHS
MICS
Other international survey
National survey
PMA
Subpopulation
+: Higher contraceptive use
−: Lower contraceptive use
A: Other age group
F: Folk methods included
S−: Sterilization included
S+: Sterilization excluded
Unmarried women
Formerly married
Partnered women

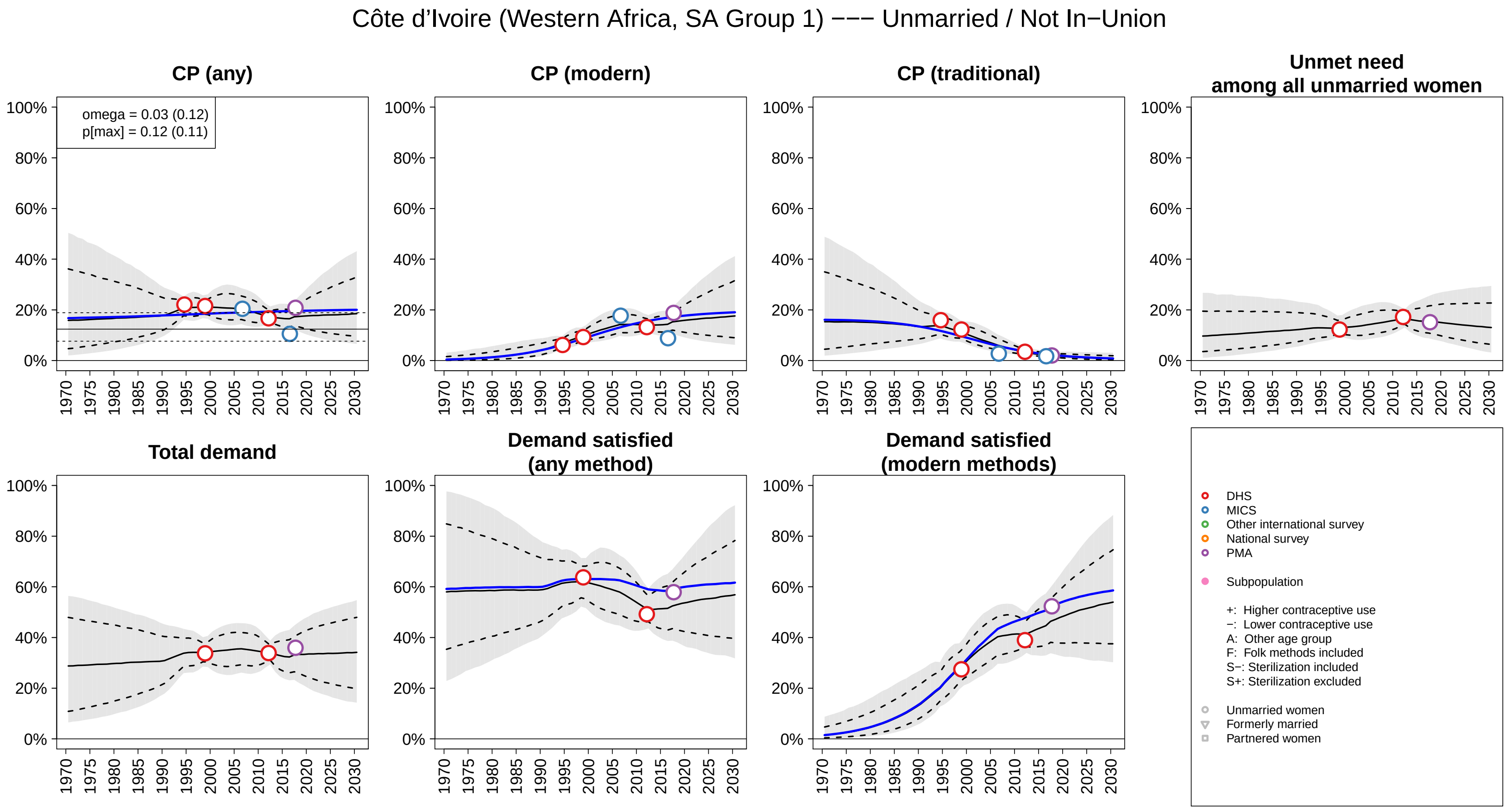
Côte d'Ivoire (Western Africa, SA Group 1) --- Unmarried / Not In-Union
CP (any)
omega = 0.03 (0.12)
p[max] = 0.12 (0.11)
CP (modern)
CP (traditional)
Unmet need among all unmarried women
Total demand
Demand satisfied (any method)
Demand satisfied (modern methods)
DHS
MICS
Other international survey
National survey
PMA
Subpopulation
+: Higher contraceptive use
−: Lower contraceptive use
A: Other age group
F: Folk methods included
S−: Sterilization included
S+: Sterilization excluded
Unmarried women
Formerly married
Partnered women

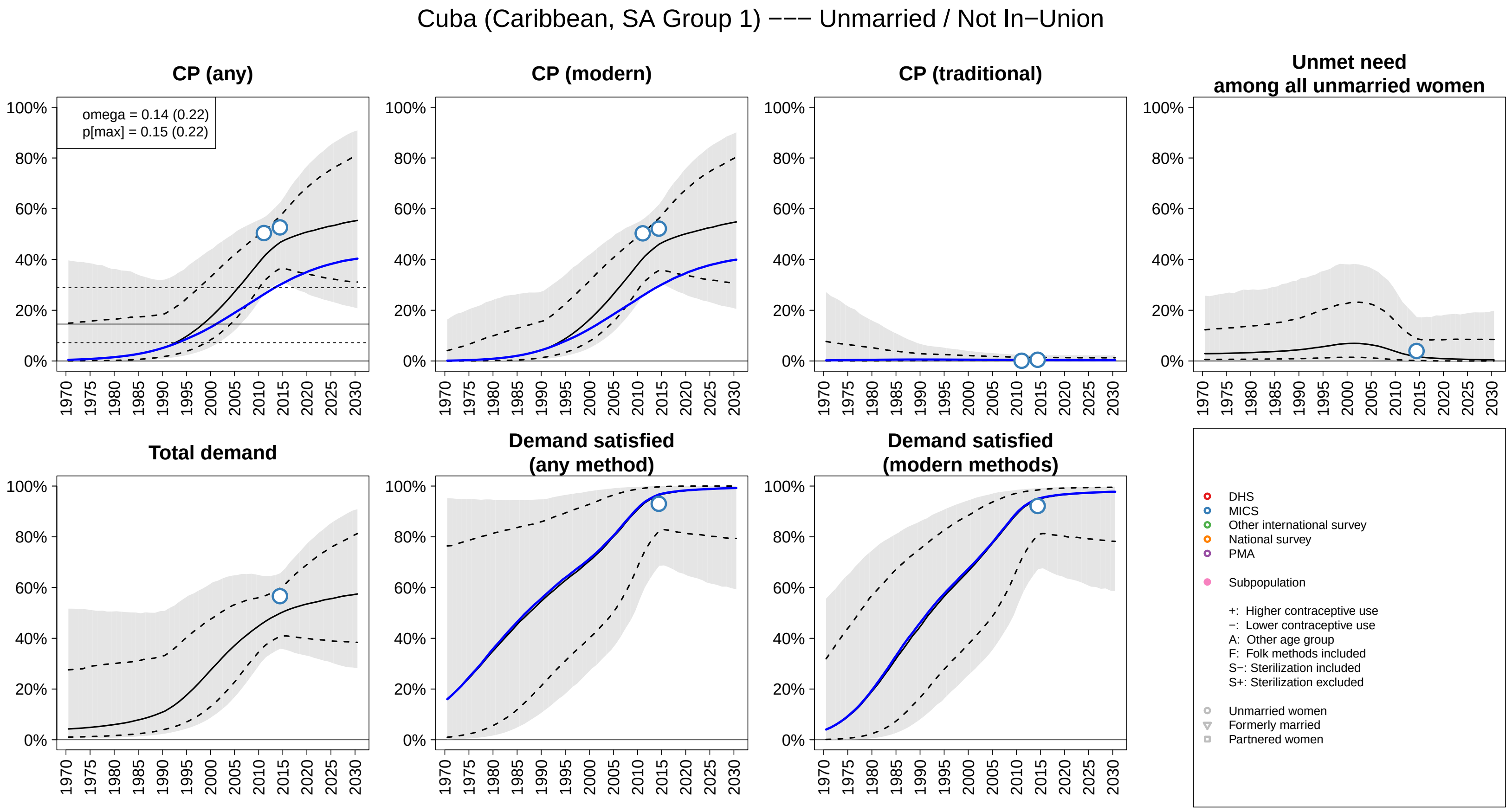

Cuba (Caribbean, SA Group 1) −−− Unmarried / Not In−Union
CP (any)
omega = 0.14 (0.22)
p[max] = 0.15 (0.22)
CP (modern)
CP (traditional)
Unmet need among all unmarried women
Total demand
Demand satisfied (any method)
Demand satisfied (modern methods)
DHS
MICS
Other international survey
National survey
PMA
Subpopulation
+: Higher contraceptive use
−: Lower contraceptive use
A: Other age group
F: Folk methods included
S−: Sterilization included
S+: Sterilization excluded
Unmarried women
Formerly married
Partnered women

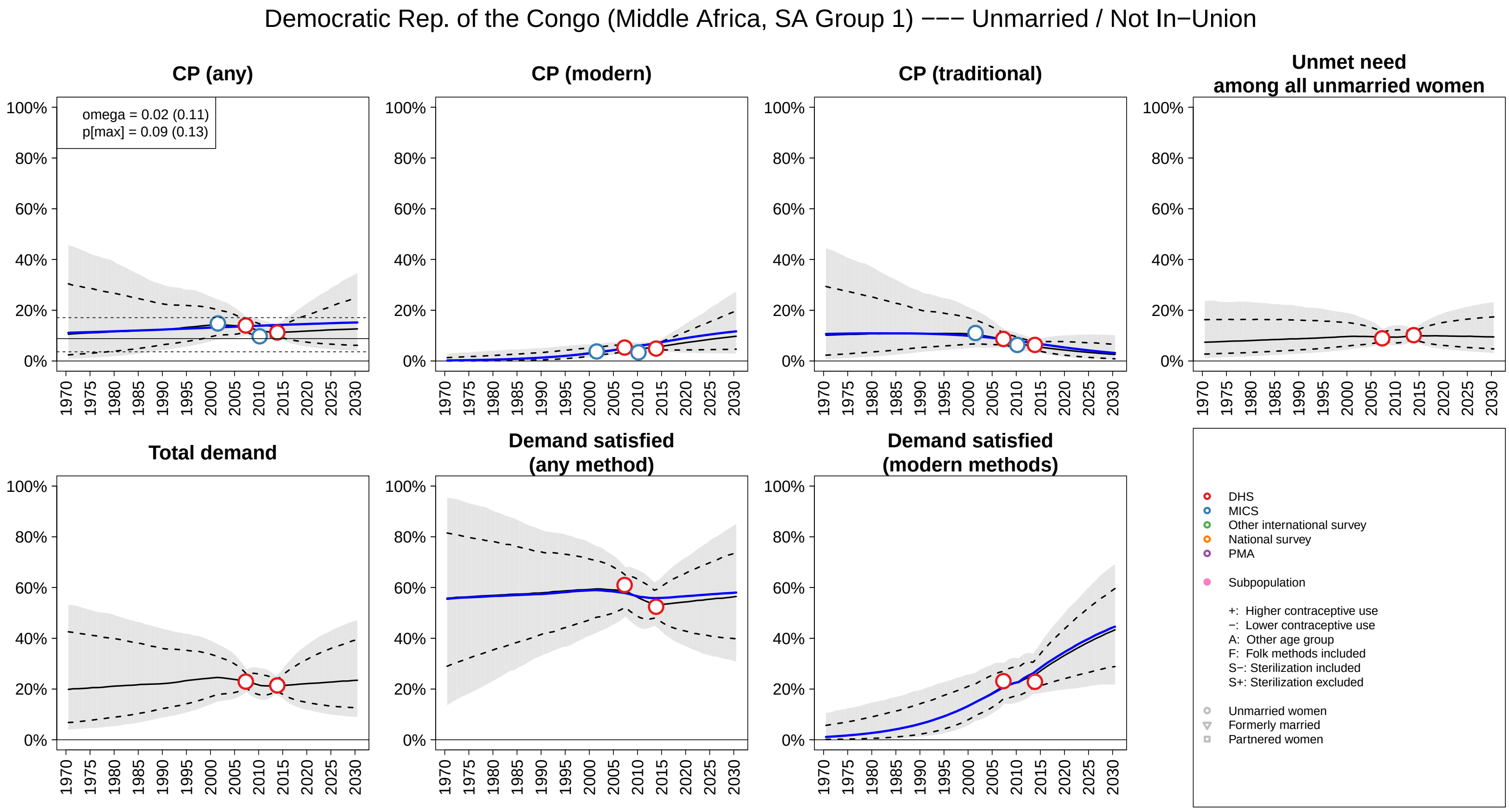
Democratic Rep. of the Congo (Middle Africa, SA Group 1) −−− Unmarried / Not In−Union
CP (any)
omega = 0.02 (0.11)
p[max] = 0.09 (0.13)
CP (modern)
CP (traditional)
Unmet need among all unmarried women
Total demand
Demand satisfied (any method)
Demand satisfied (modern methods)
DHS
MICS
Other international survey
National survey
PMA
Subpopulation
+: Higher contraceptive use
−: Lower contraceptive use
A: Other age group
F: Folk methods included
S−: Sterilization included
S+: Sterilization excluded
Unmarried women
Formerly married
Partnered women

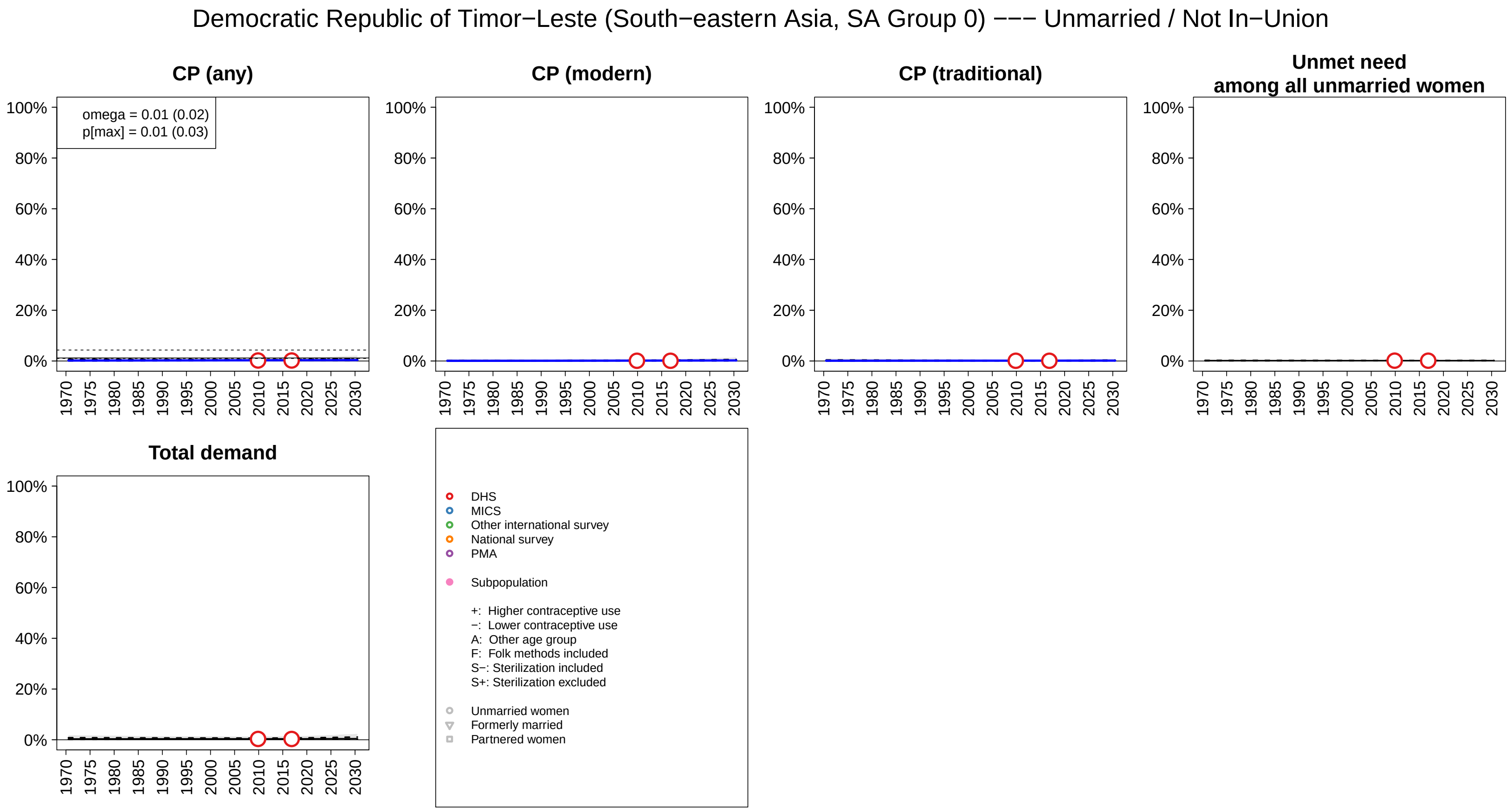
Democratic Republic of Timor−Leste (South−eastern Asia, SA Group 0) −−− Unmarried / Not In−Union
CP (any)
omega = 0.01 (0.02)
p[max] = 0.01 (0.03)
CP (modern)
CP (traditional)
Unmet need
among all unmarried women
Total demand
DHS
MICS
Other international survey
National survey
PMA
Subpopulation
+: Higher contraceptive use
−: Lower contraceptive use
A: Other age group
F: Folk methods included
S−: Sterilization included
S+: Sterilization excluded
Unmarried women
Formerly married
Partnered women

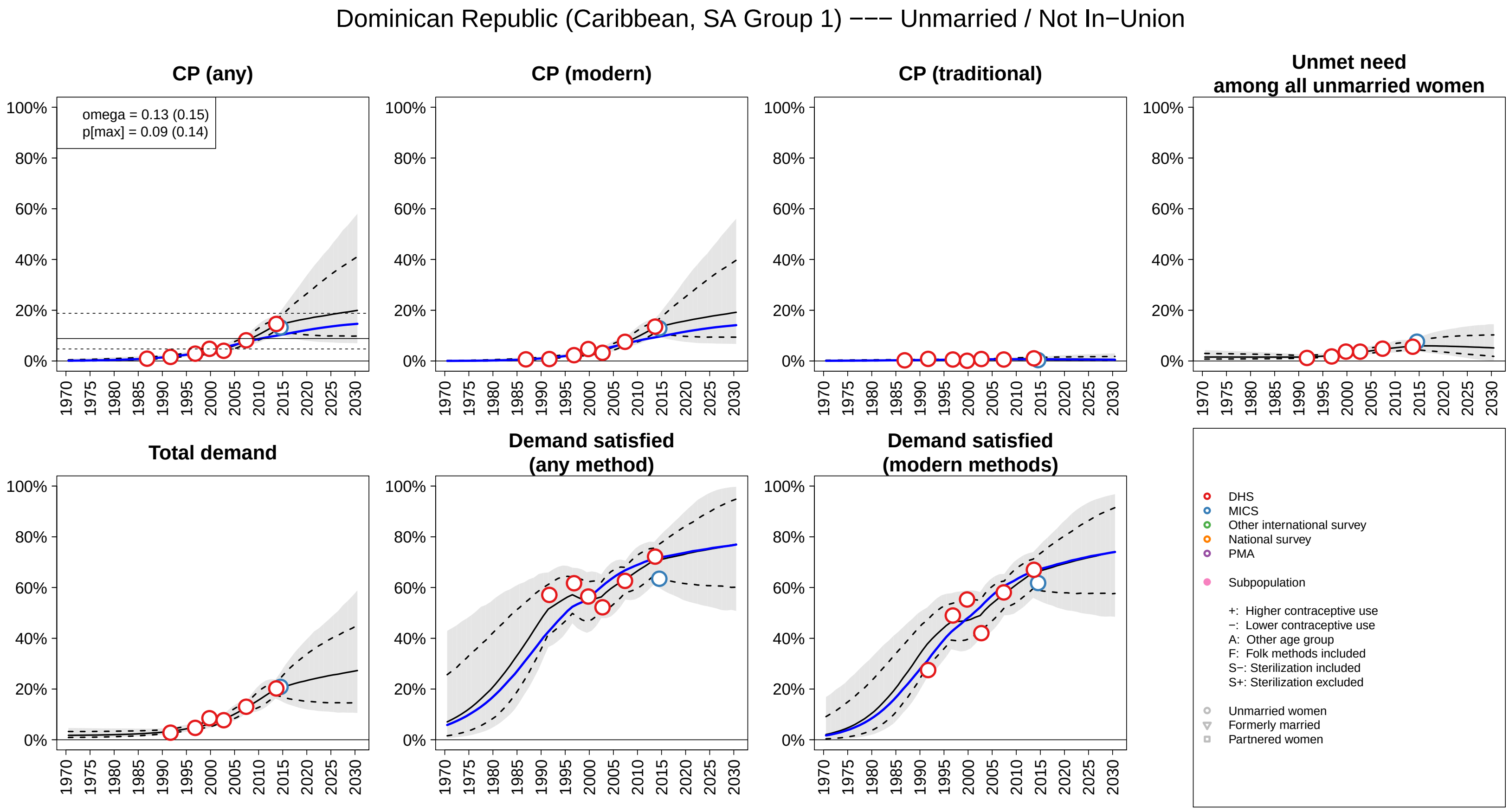
Dominican Republic (Caribbean, SA Group 1) --- Unmarried / Not In-Union
CP (any)
omega = 0.13 (0.15)
p[max] = 0.09 (0.14)
CP (modern)
CP (traditional)
Unmet need among all unmarried women
Total demand
Demand satisfied (any method)
Demand satisfied (modern methods)
DHS
MICS
Other international survey
National survey
PMA
Subpopulation
+: Higher contraceptive use
−: Lower contraceptive use
A: Other age group
F: Folk methods included
S−: Sterilization included
S+: Sterilization excluded
Unmarried women
Formerly married
Partnered women

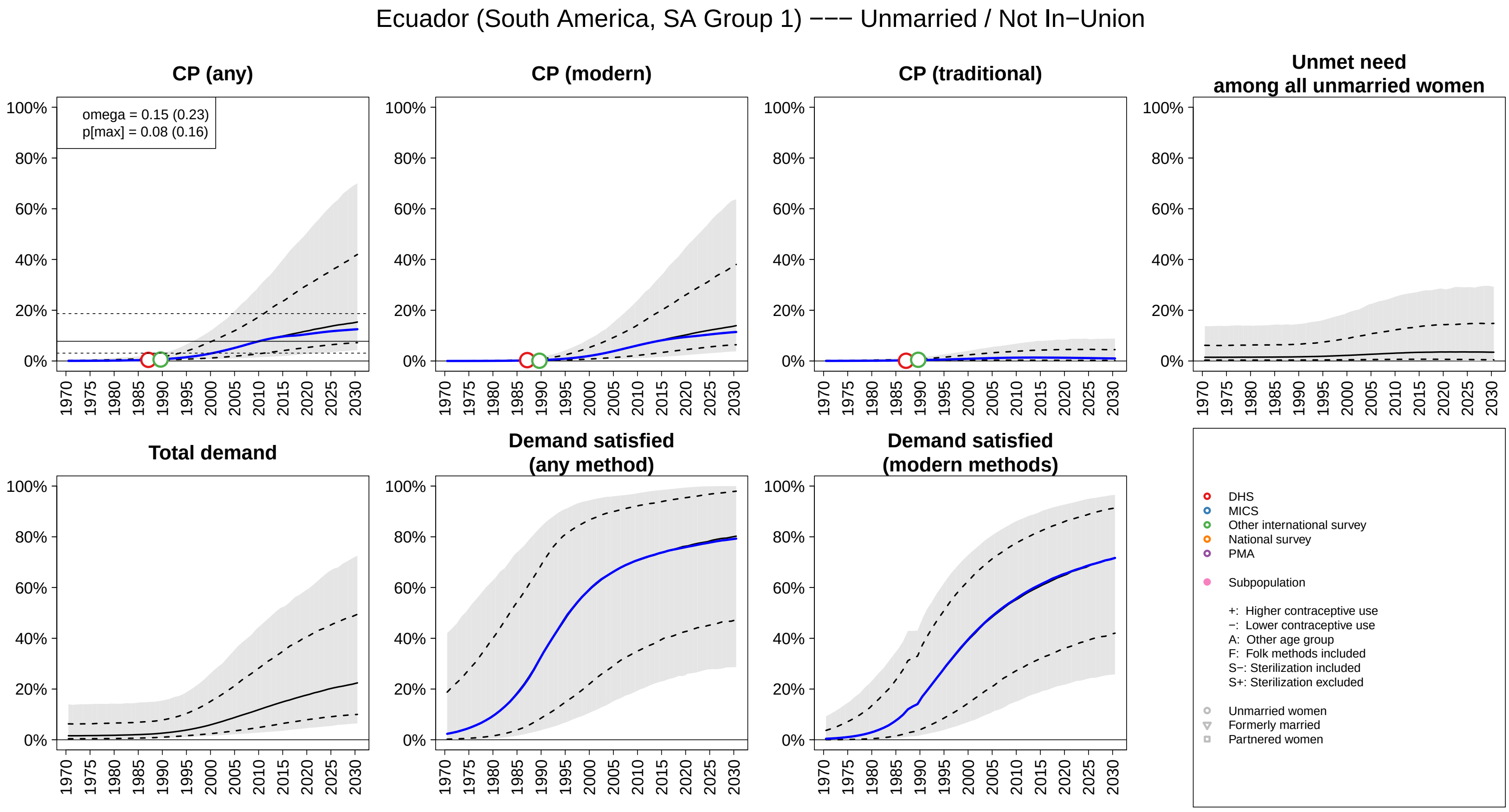
Ecuador (South America, SA Group 1) −−− Unmarried / Not In−Union
CP (any)
omega = 0.15 (0.23)
p[max] = 0.08 (0.16)
CP (modern)
CP (traditional)
Unmet need among all unmarried women
Total demand
Demand satisfied (any method)
Demand satisfied (modern methods)
DHS
MICS
Other international survey
National survey
PMA
Subpopulation
+: Higher contraceptive use
−: Lower contraceptive use
A: Other age group
F: Folk methods included
S−: Sterilization included
S+: Sterilization excluded
Unmarried women
Formerly married
Partnered women

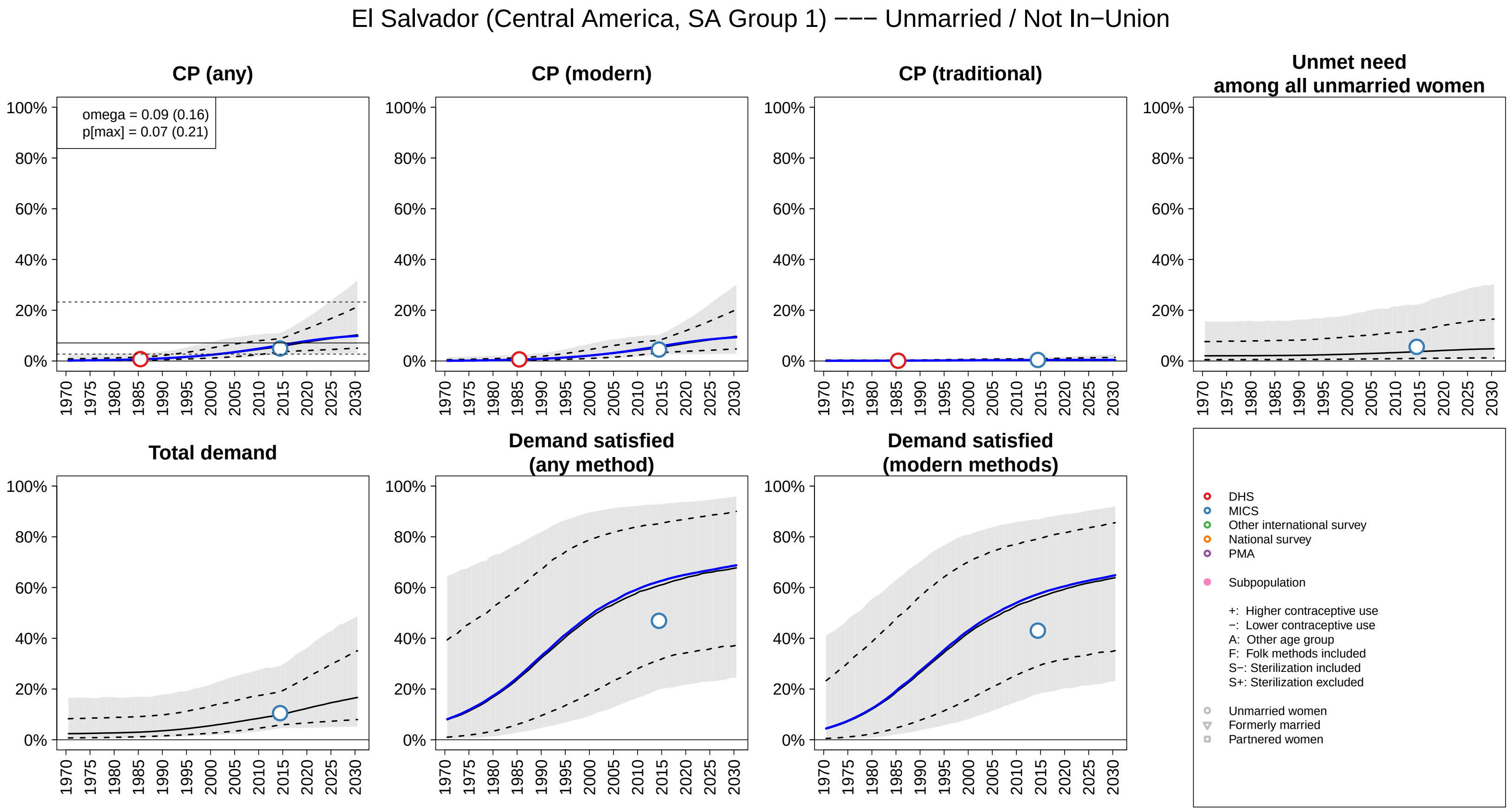
El Salvador (Central America, SA Group 1) --- Unmarried / Not In-Union
CP (any)
omega = 0.09 (0.16)
p[max] = 0.07 (0.21)
CP (modern)
CP (traditional)
Unmet need among all unmarried women
Total demand
Demand satisfied (any method)
Demand satisfied (modern methods)
DHS
MICS
Other international survey
National survey
PMA
Subpopulation
+: Higher contraceptive use
−: Lower contraceptive use
A: Other age group
F: Folk methods included
S−: Sterilization included
S+: Sterilization excluded
Unmarried women
Formerly married
Partnered women

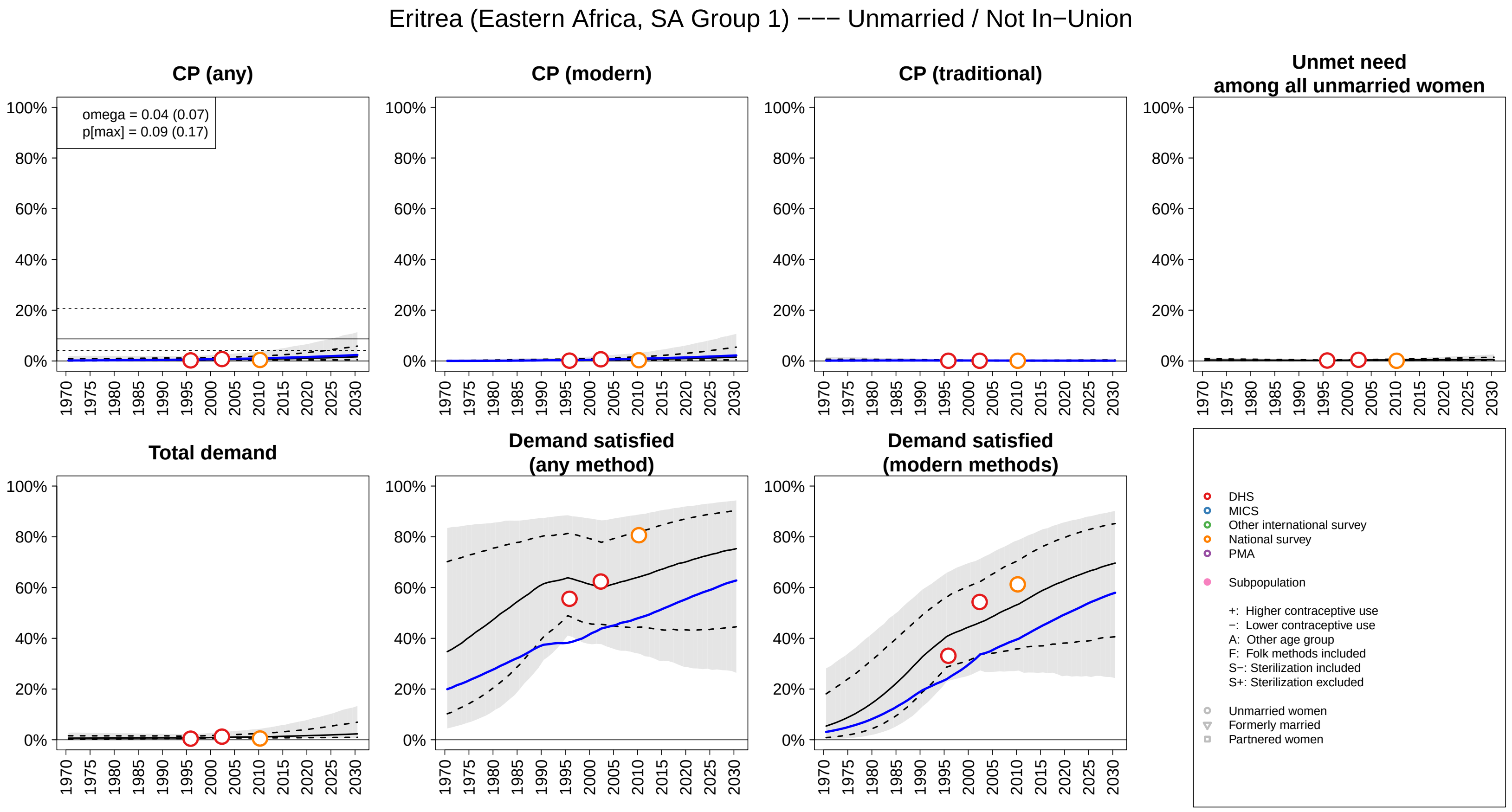
Eritrea (Eastern Africa, SA Group 1) --- Unmarried / Not In−Union
CP (any)
omega = 0.04 (0.07)
p[max] = 0.09 (0.17)
CP (modern)
CP (traditional)
Unmet need among all unmarried women
Total demand
Demand satisfied (any method)
Demand satisfied (modern methods)
DHS
MICS
Other international survey
National survey
PMA
Subpopulation
+: Higher contraceptive use
−: Lower contraceptive use
A: Other age group
F: Folk methods included
S−: Sterilization included
S+: Sterilization excluded
Unmarried women
Formerly married
Partnered women

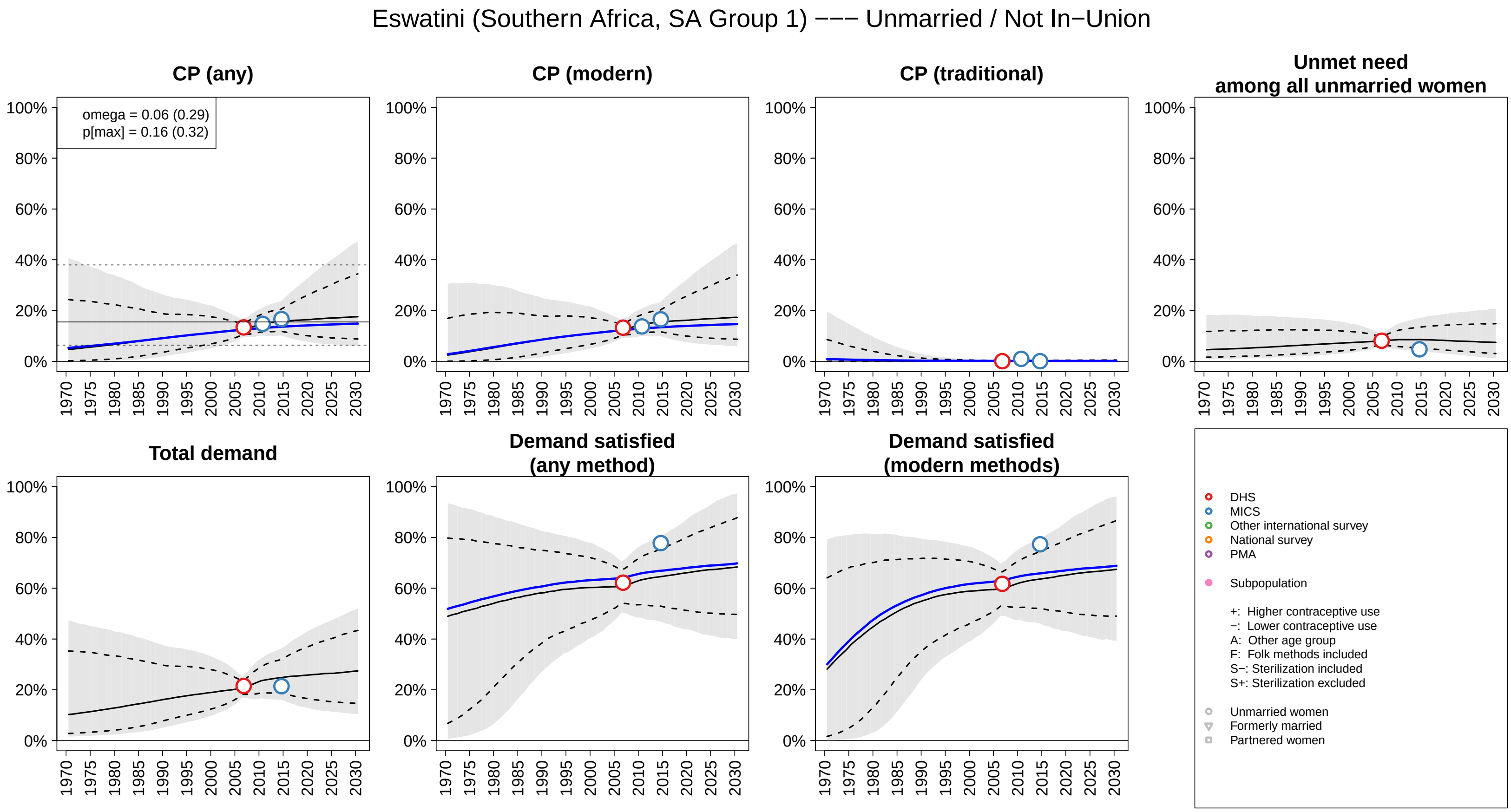

Eswatini (Southern Africa, SA Group 1) −−− Unmarried / Not In−Union
CP (any)
omega = 0.06 (0.29)
p[max] = 0.16 (0.32)
CP (modern)
CP (traditional)
Unmet need among all unmarried women
Total demand
Demand satisfied (any method)
Demand satisfied (modern methods)
DHS
MICS
Other international survey
National survey
PMA
Subpopulation
+: Higher contraceptive use
−: Lower contraceptive use
A: Other age group
F: Folk methods included
S−: Sterilization included
S+: Sterilization excluded
Unmarried women
Formerly married
Partnered women

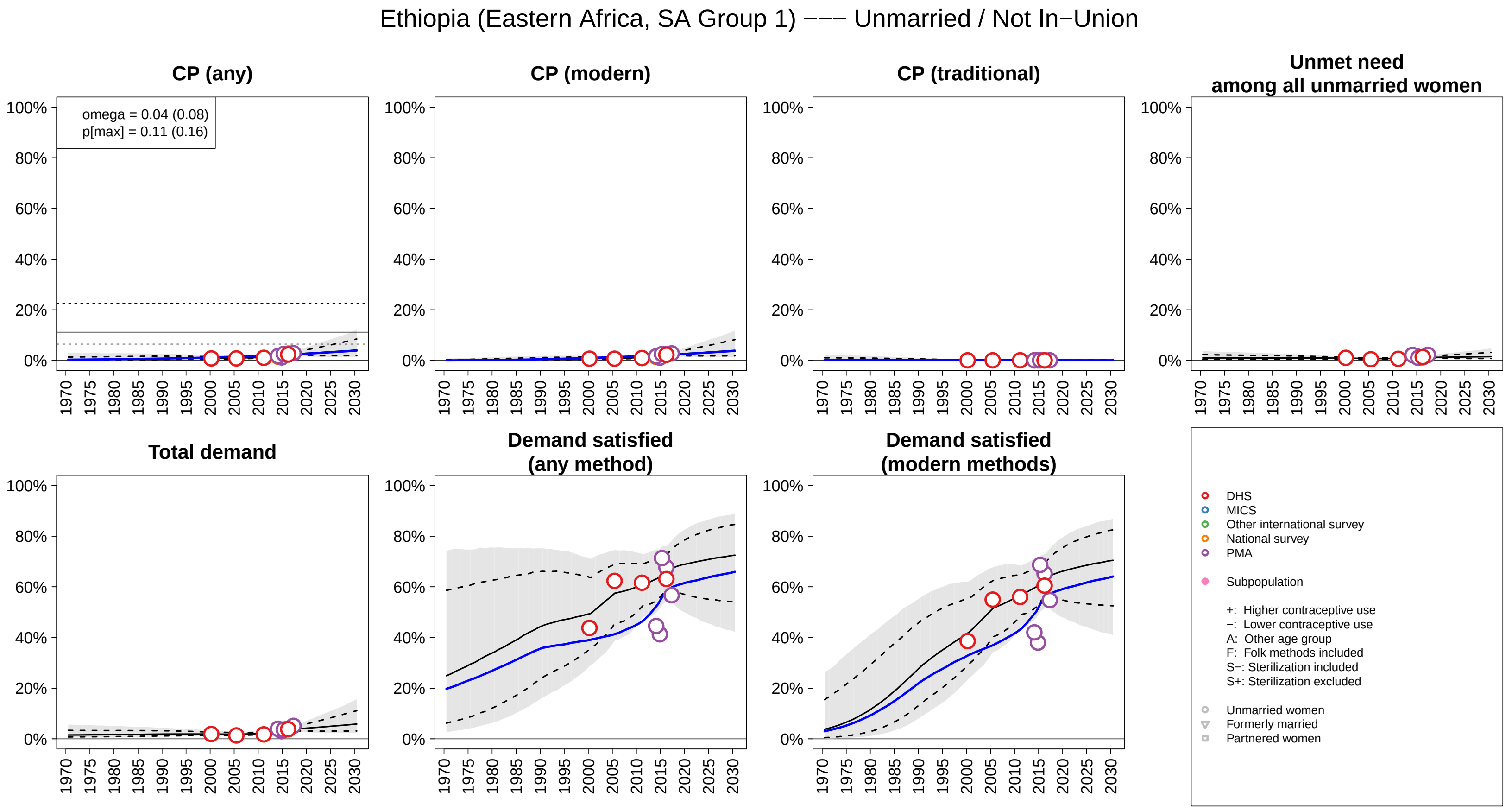
Ethiopia (Eastern Africa, SA Group 1) ––– Unmarried / Not In−Union
CP (any)
omega = 0.04 (0.08)
p[max] = 0.11 (0.16)
CP (modern)
CP (traditional)
Unmet need among all unmarried women
Total demand
Demand satisfied (any method)
Demand satisfied (modern methods)
DHS
MICS
Other international survey
National survey
PMA
Subpopulation
+: Higher contraceptive use
−: Lower contraceptive use
A: Other age group
F: Folk methods included
S−: Sterilization included
S+: Sterilization excluded
Unmarried women
Formerly married
Partnered women

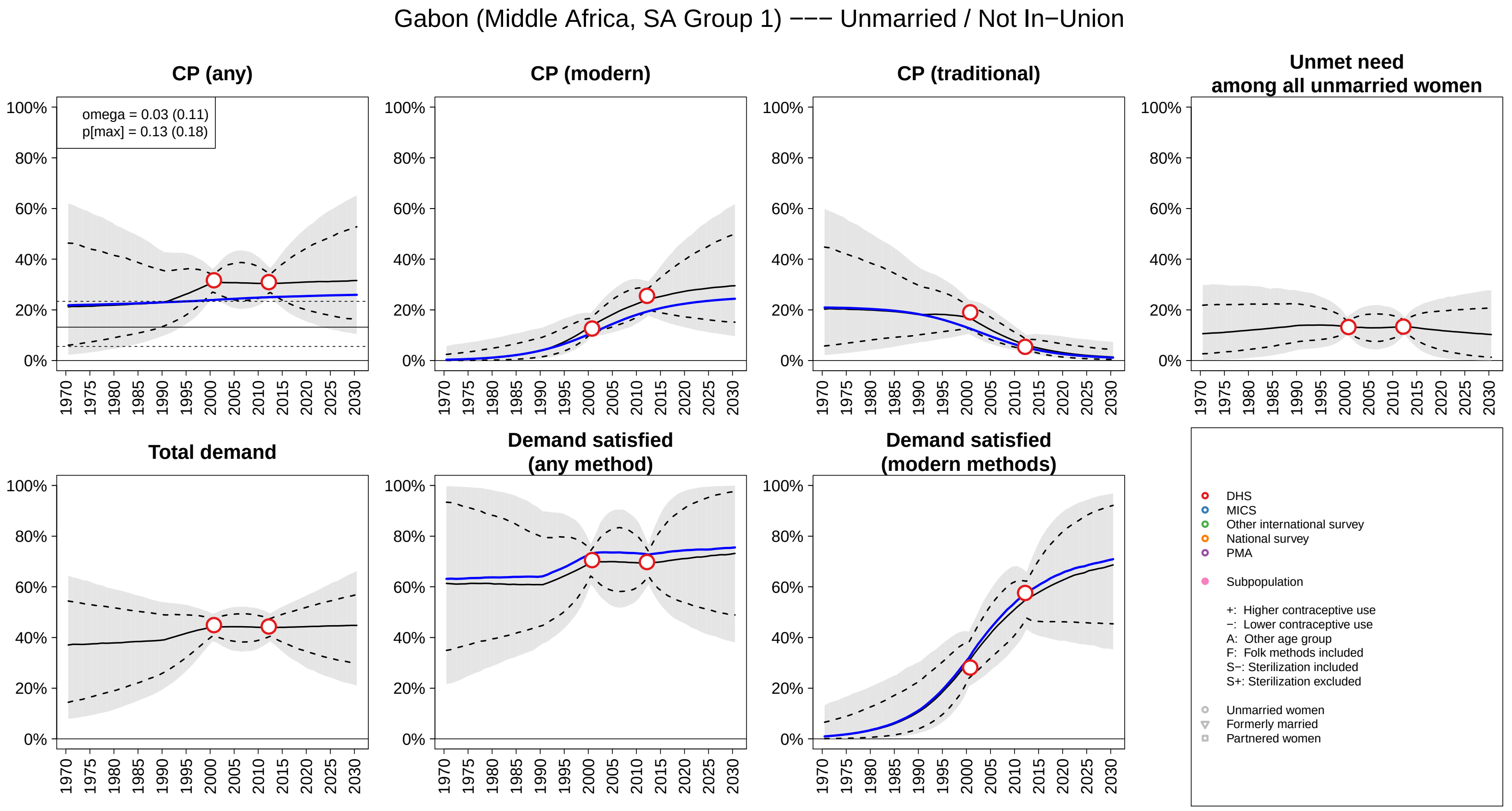
Gabon (Middle Africa, SA Group 1) −−− Unmarried / Not In−Union
CP (any)
omega = 0.03 (0.11)
p[max] = 0.13 (0.18)
CP (modern)
CP (traditional)
Unmet need
among all unmarried women
Total demand
Demand satisfied
(any method)
Demand satisfied
(modern methods)
DHS
MICS
Other international survey
National survey
PMA
Subpopulation
+: Higher contraceptive use
−: Lower contraceptive use
A: Other age group
F: Folk methods included
S−: Sterilization included
S+: Sterilization excluded
Unmarried women
Formerly married
Partnered women

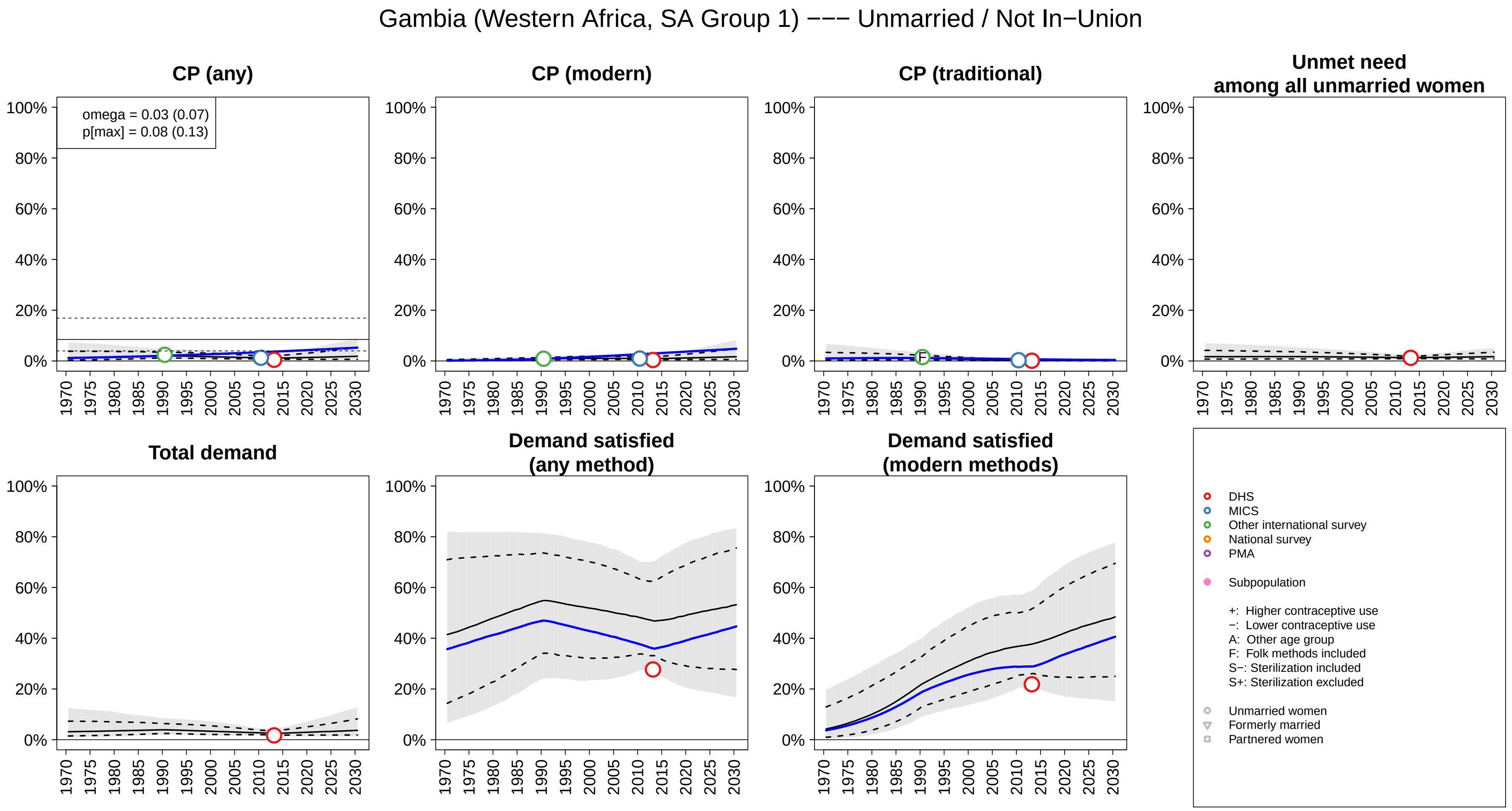
Gambia (Western Africa, SA Group 1) −−− Unmarried / Not In−Union
CP (any)
omega = 0.03 (0.07)
p[max] = 0.08 (0.13)
CP (modern)
CP (traditional)
Unmet need among all unmarried women
Total demand
Demand satisfied (any method)
Demand satisfied (modern methods)
DHS
MICS
Other international survey
National survey
PMA
Subpopulation
+: Higher contraceptive use
−: Lower contraceptive use
A: Other age group
F: Folk methods included
S−: Sterilization included
S+: Sterilization excluded
Unmarried women
Formerly married
Partnered women

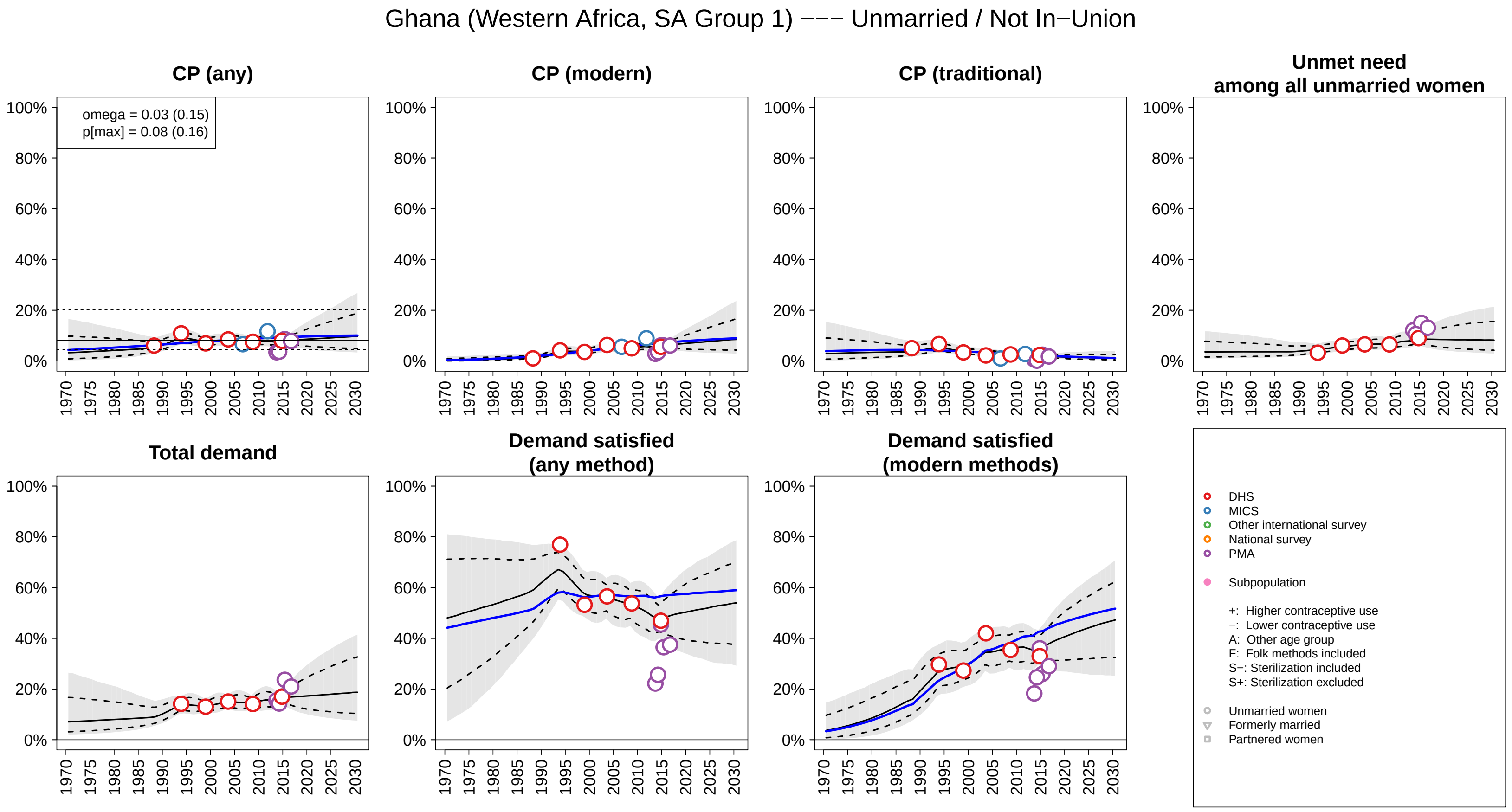
Ghana (Western Africa, SA Group 1) --- Unmarried / Not In-Union
CP (any)
omega = 0.03 (0.15)
p[max] = 0.08 (0.16)
CP (modern)
CP (traditional)
Unmet need among all unmarried women
Total demand
Demand satisfied (any method)
Demand satisfied (modern methods)
DHS
MICS
Other international survey
National survey
PMA
Subpopulation
+: Higher contraceptive use
−: Lower contraceptive use
A: Other age group
F: Folk methods included
S−: Sterilization included
S+: Sterilization excluded
Unmarried women
Formerly married
Partnered women

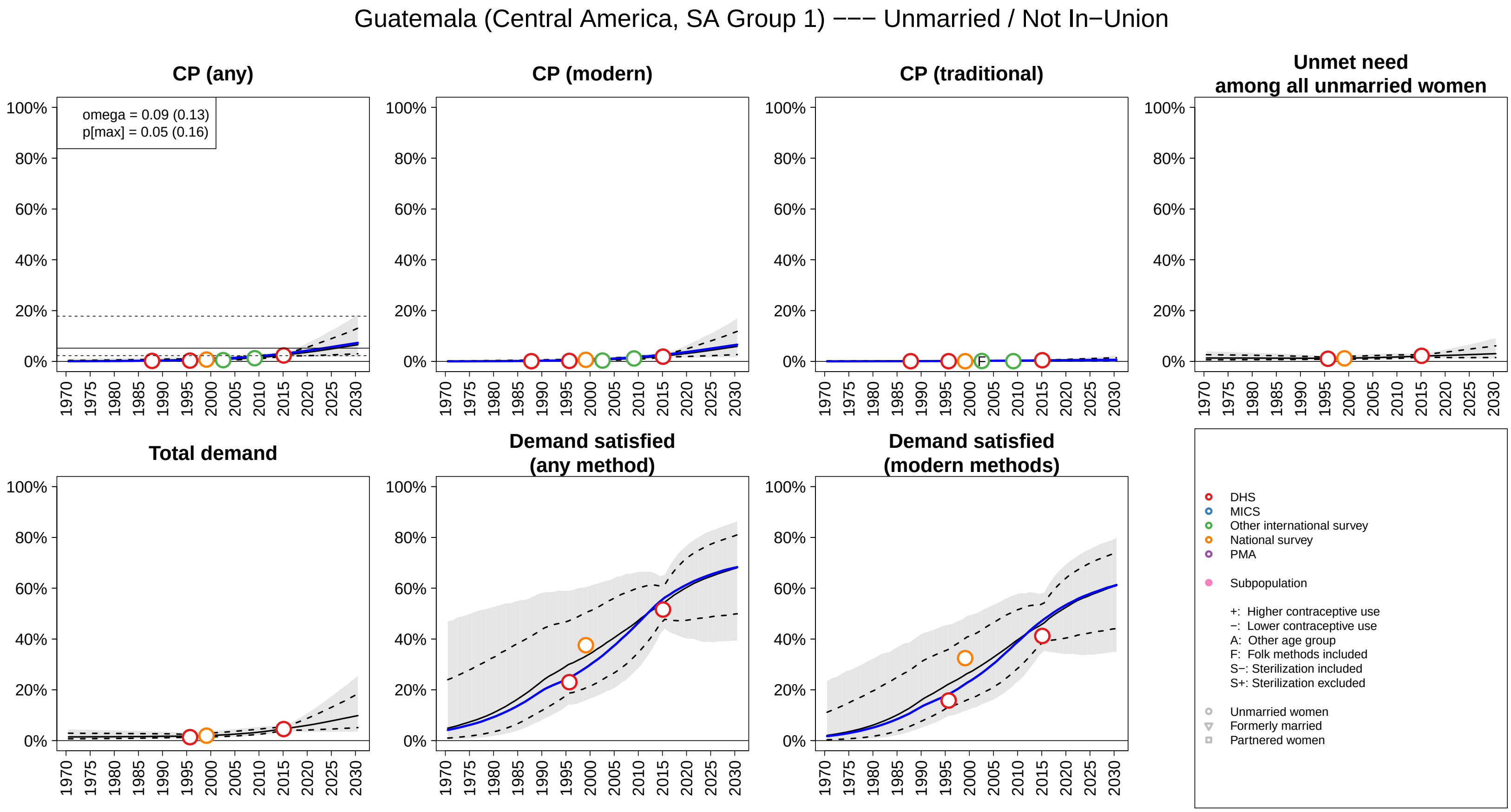
Guatemala (Central America, SA Group 1) ––– Unmarried / Not In−Union
CP (any)
omega = 0.09 (0.13)
p[max] = 0.05 (0.16)
CP (modern)
CP (traditional)
Unmet need among all unmarried women
Total demand
Demand satisfied (any method)
Demand satisfied (modern methods)
DHS
MICS
Other international survey
National survey
PMA
Subpopulation
+: Higher contraceptive use
−: Lower contraceptive use
A: Other age group
F: Folk methods included
S−: Sterilization included
S+: Sterilization excluded
Unmarried women
Formerly married
Partnered women

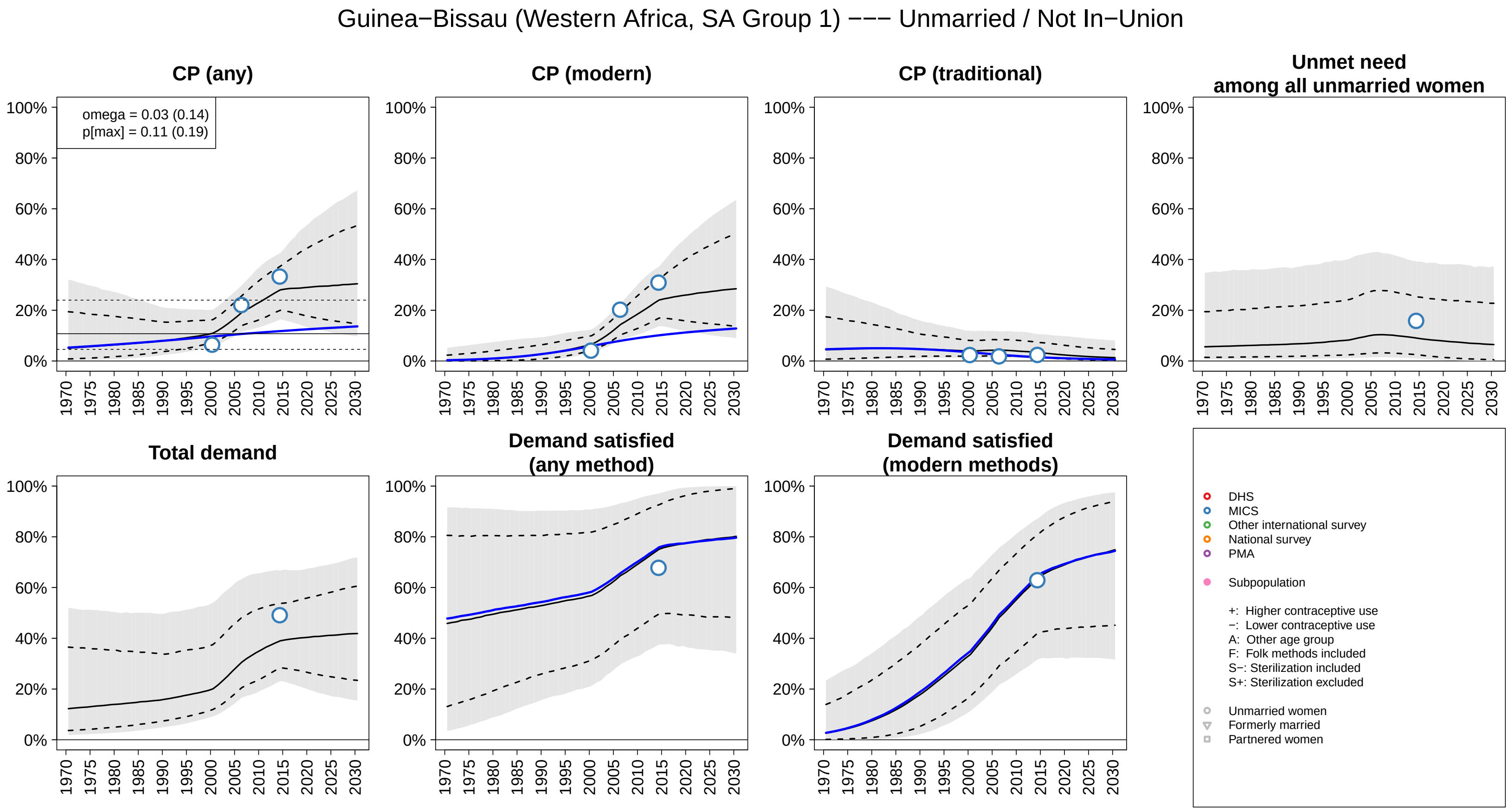
Guinea−Bissau (Western Africa, SA Group 1) −−− Unmarried / Not In−Union
CP (any)
omega = 0.03 (0.14)
p[max] = 0.11 (0.19)
CP (modern)
CP (traditional)
Unmet need among all unmarried women
Total demand
Demand satisfied (any method)
Demand satisfied (modern methods)
DHS
MICS
Other international survey
National survey
PMA
Subpopulation
+: Higher contraceptive use
−: Lower contraceptive use
A: Other age group
F: Folk methods included
S−: Sterilization included
S+: Sterilization excluded
Unmarried women
Formerly married
Partnered women

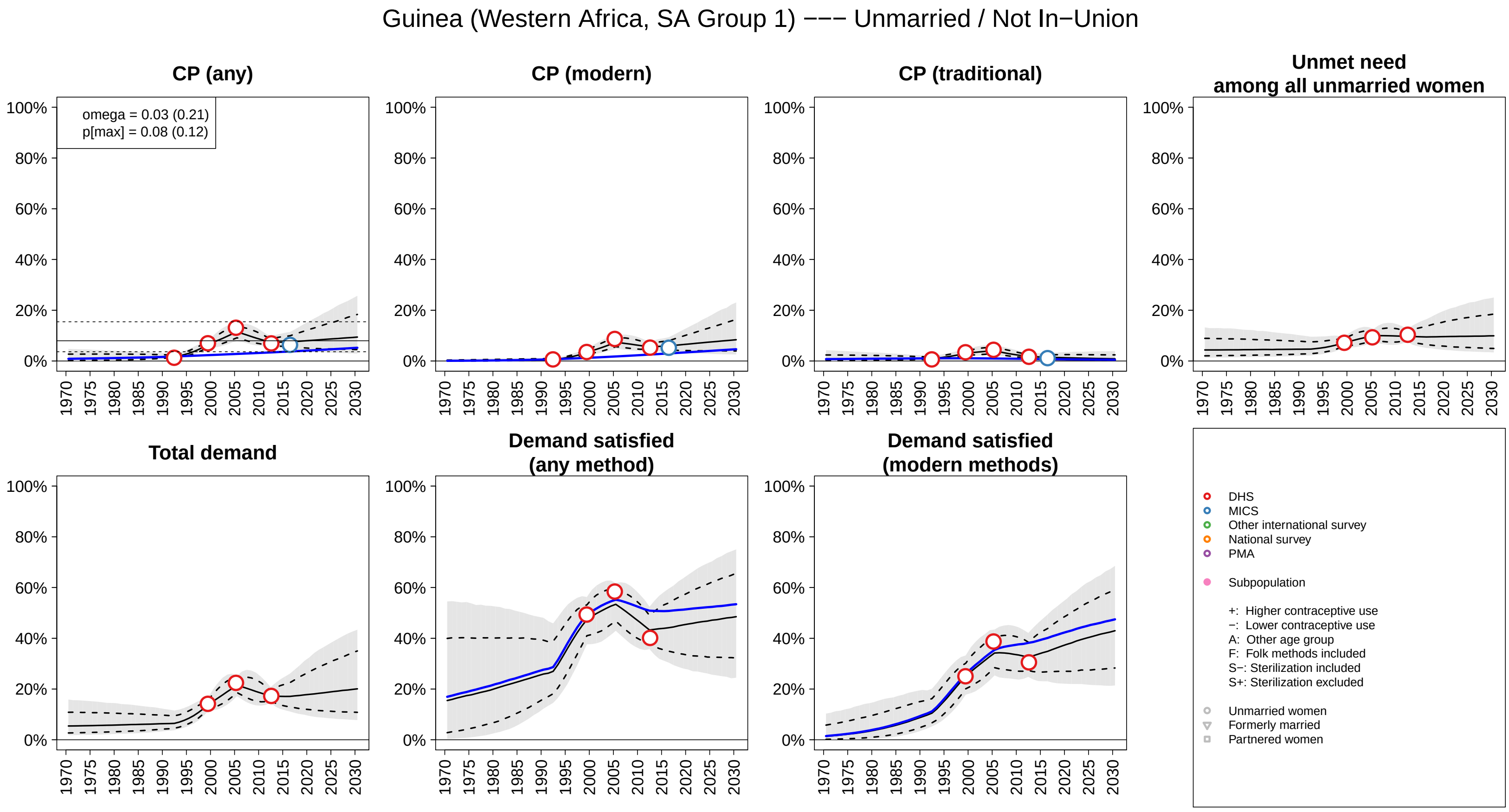
Guinea (Western Africa, SA Group 1) −−− Unmarried / Not In−Union
CP (any)
omega = 0.03 (0.21)
p[max] = 0.08 (0.12)
CP (modern)
CP (traditional)
Unmet need among all unmarried women
Total demand
Demand satisfied (any method)
Demand satisfied (modern methods)
DHS
MICS
Other international survey
National survey
PMA
Subpopulation
+: Higher contraceptive use
−: Lower contraceptive use
A: Other age group
F: Folk methods included
S−: Sterilization included
S+: Sterilization excluded
Unmarried women
Formerly married
Partnered women

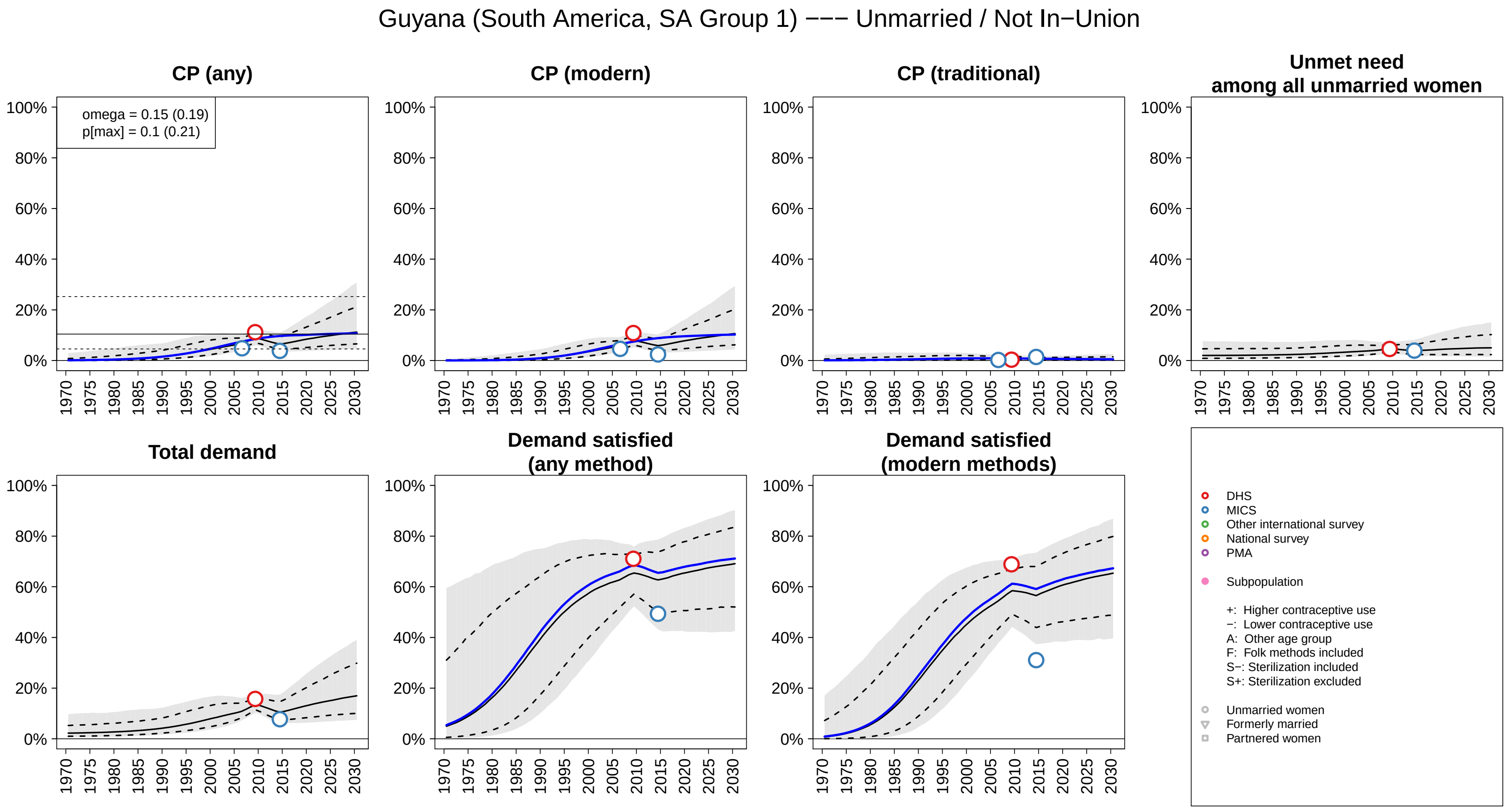
Guyana (South America, SA Group 1) −−− Unmarried / Not In−Union
CP (any)
omega = 0.15 (0.19)
p[max] = 0.1 (0.21)
CP (modern)
CP (traditional)
Unmet need among all unmarried women
Total demand
Demand satisfied (any method)
Demand satisfied (modern methods)
DHS
MICS
Other international survey
National survey
PMA
Subpopulation
+: Higher contraceptive use
−: Lower contraceptive use
A: Other age group
F: Folk methods included
S−: Sterilization included
S+: Sterilization excluded
Unmarried women
Formerly married
Partnered women

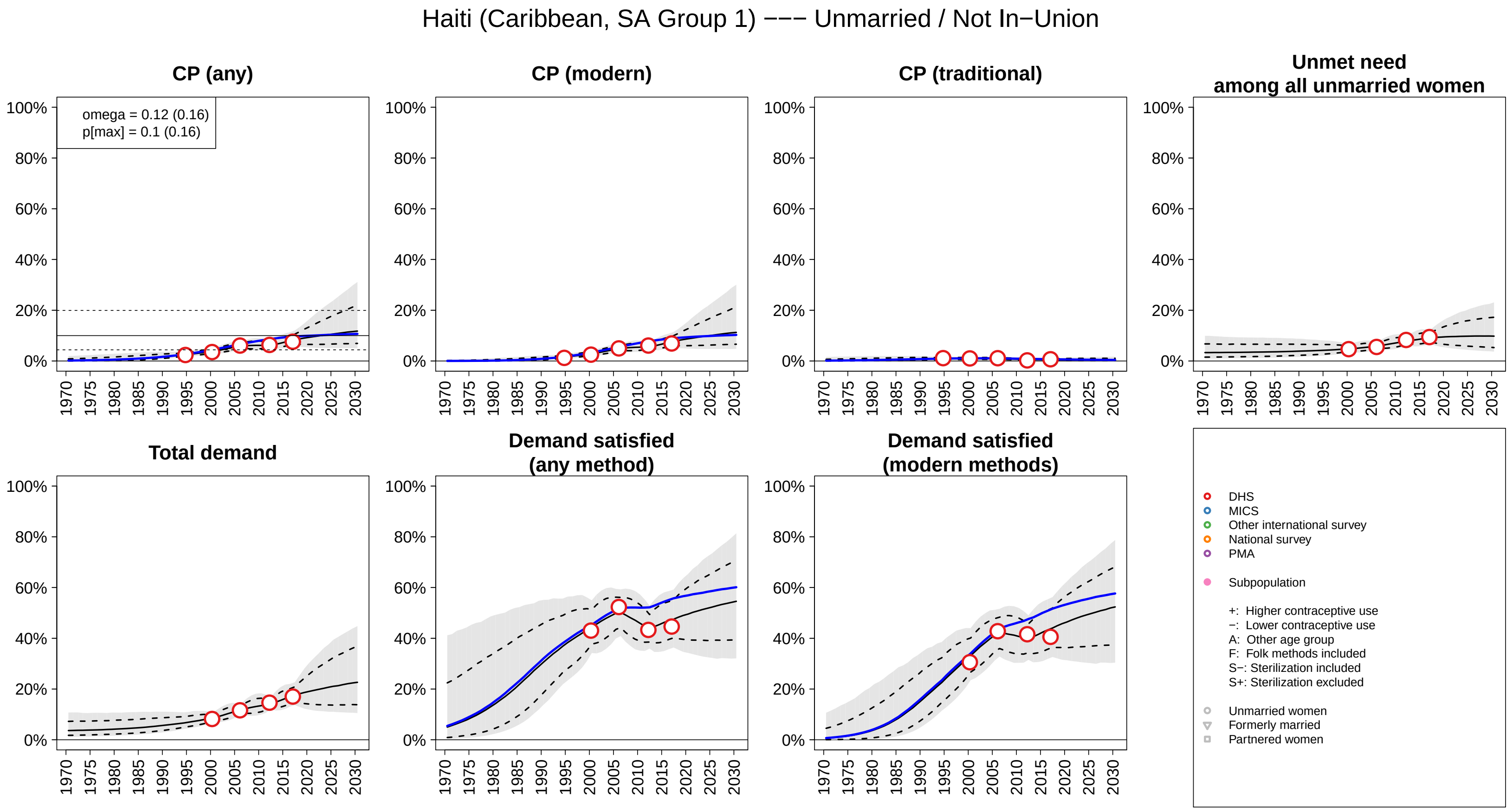
Haiti (Caribbean, SA Group 1) −−− Unmarried / Not In−Union
CP (any)
omega = 0.12 (0.16)
p[max] = 0.1 (0.16)
CP (modern)
CP (traditional)
Unmet need among all unmarried women
Total demand
Demand satisfied (any method)
Demand satisfied (modern methods)
DHS
MICS
Other international survey
National survey
PMA
Subpopulation
+: Higher contraceptive use
−: Lower contraceptive use
A: Other age group
F: Folk methods included
S−: Sterilization included
S+: Sterilization excluded
Unmarried women
Formerly married
Partnered women

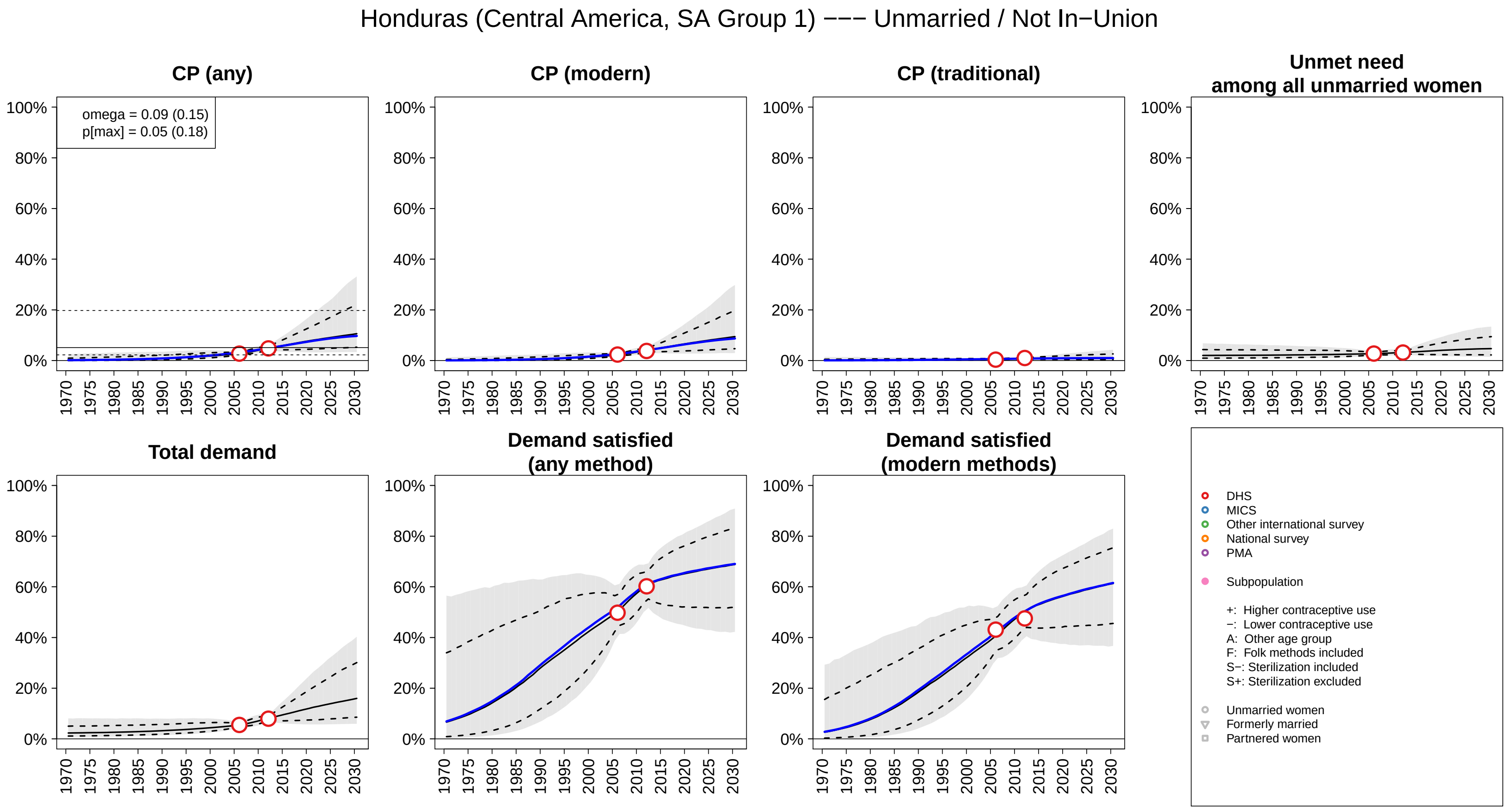
Honduras (Central America, SA Group 1) --- Unmarried / Not In−Union
CP (any)
omega = 0.09 (0.15)
p[max] = 0.05 (0.18)
CP (modern)
CP (traditional)
Unmet need among all unmarried women
Total demand
Demand satisfied (any method)
Demand satisfied (modern methods)
DHS
MICS
Other international survey
National survey
PMA
Subpopulation
+: Higher contraceptive use
−: Lower contraceptive use
A: Other age group
F: Folk methods included
S−: Sterilization included
S+: Sterilization excluded
Unmarried women
Formerly married
Partnered women

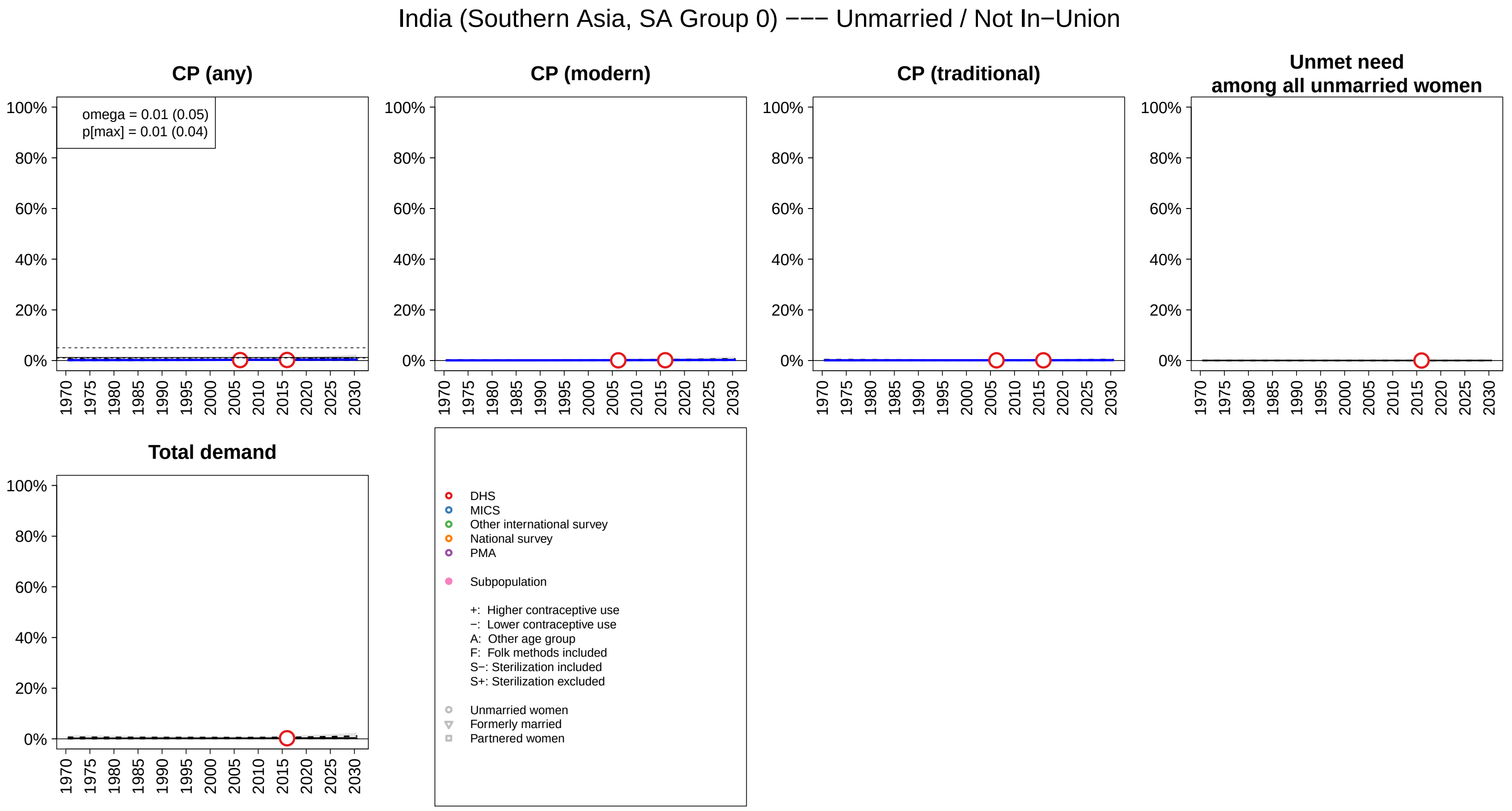
India (Southern Asia, SA Group 0) ––– Unmarried / Not In−Union
CP (any)
omega = 0.01 (0.05)
p[max] = 0.01 (0.04)
CP (modern)
CP (traditional)
Unmet need among all unmarried women
Total demand
0%
20%
40%
60%
80%
100%
1970
1975
1980
1985
1990
1995
2000
2005
2010
2015
2020
2025
2030
DHS
MICS
Other international survey
National survey
PMA
Subpopulation
+: Higher contraceptive use
−: Lower contraceptive use
A: Other age group
F: Folk methods included
S−: Sterilization included
S+: Sterilization excluded
Unmarried women
Formerly married
Partnered women

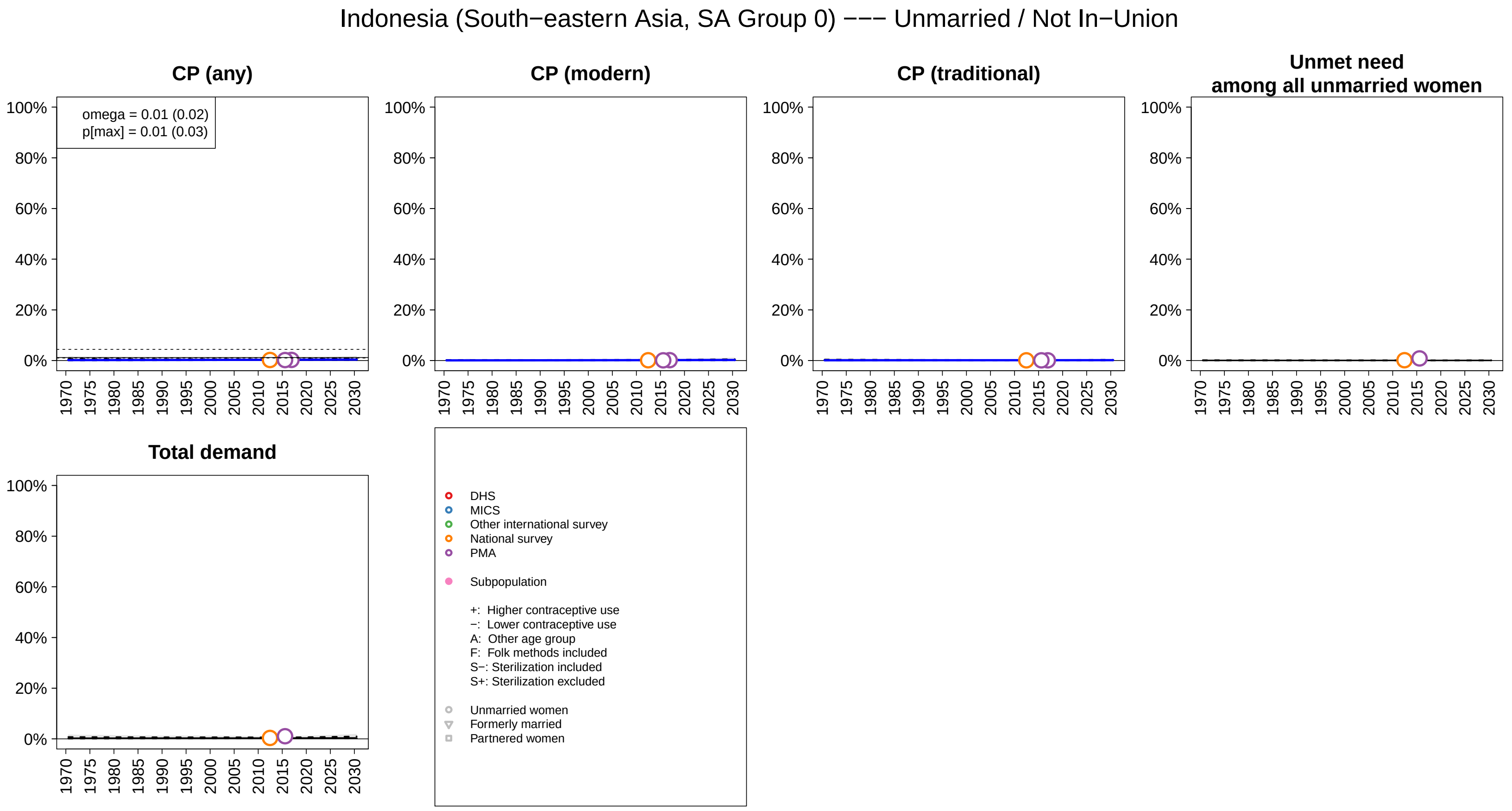
Indonesia (South−eastern Asia, SA Group 0) −−− Unmarried / Not In−Union
CP (any)
omega = 0.01 (0.02)
p[max] = 0.01 (0.03)
CP (modern)
CP (traditional)
Unmet need
among all unmarried women
Total demand
100%
80%
60%
40%
20%
0%
1970
1975
1980
1985
1990
1995
2000
2005
2010
2015
2020
2025
2030
DHS
MICS
Other international survey
National survey
PMA
Subpopulation
+: Higher contraceptive use
−: Lower contraceptive use
A: Other age group
F: Folk methods included
S−: Sterilization included
S+: Sterilization excluded
Unmarried women
Formerly married
Partnered women

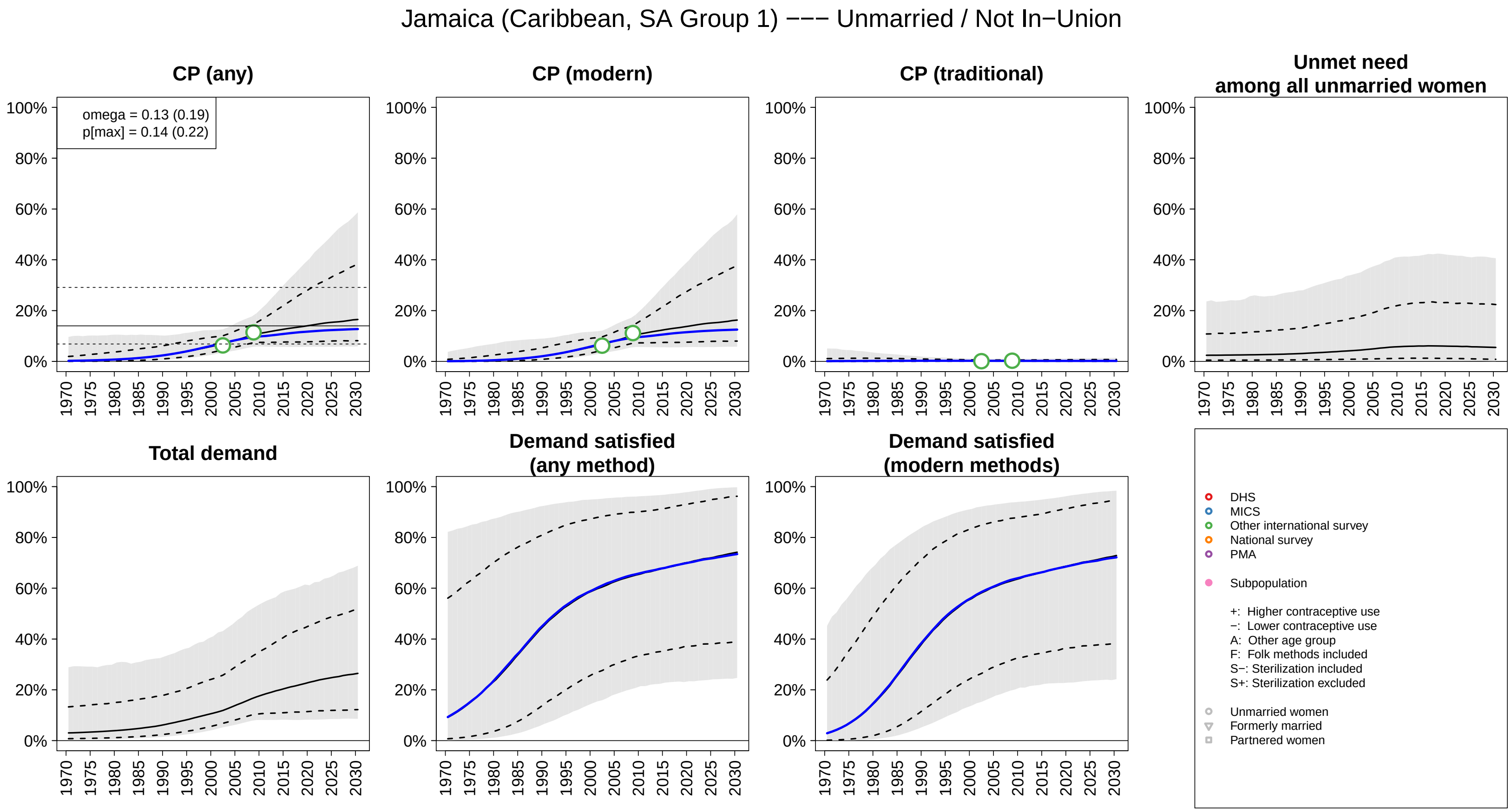
Jamaica (Caribbean, SA Group 1) ––– Unmarried / Not In–Union
CP (any)
omega = 0.13 (0.19)
p[max] = 0.14 (0.22)
CP (modern)
CP (traditional)
Unmet need among all unmarried women
Total demand
Demand satisfied (any method)
Demand satisfied (modern methods)
DHS
MICS
Other international survey
National survey
PMA
Subpopulation
+: Higher contraceptive use
−: Lower contraceptive use
A: Other age group
F: Folk methods included
S−: Sterilization included
S+: Sterilization excluded
Unmarried women
Formerly married
Partnered women

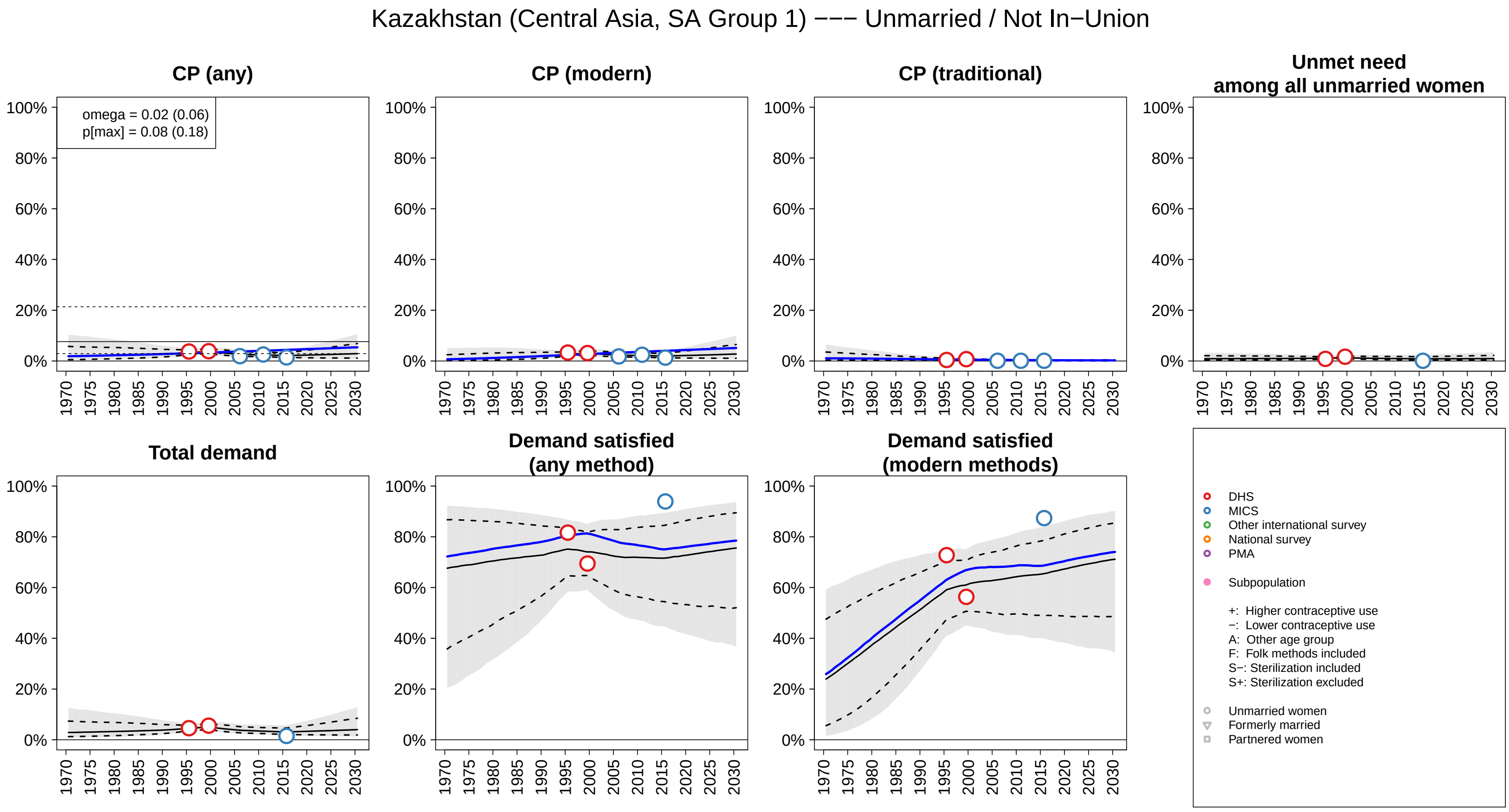

Kazakhstan (Central Asia, SA Group 1) −−− Unmarried / Not In−Union
CP (any)
omega = 0.02 (0.06)
p[max] = 0.08 (0.18)
CP (modern)
CP (traditional)
Unmet need among all unmarried women
Total demand
Demand satisfied (any method)
Demand satisfied (modern methods)
DHS
MICS
Other international survey
National survey
PMA
Subpopulation
+: Higher contraceptive use
−: Lower contraceptive use
A: Other age group
F: Folk methods included
S−: Sterilization included
S+: Sterilization excluded
Unmarried women
Formerly married
Partnered women

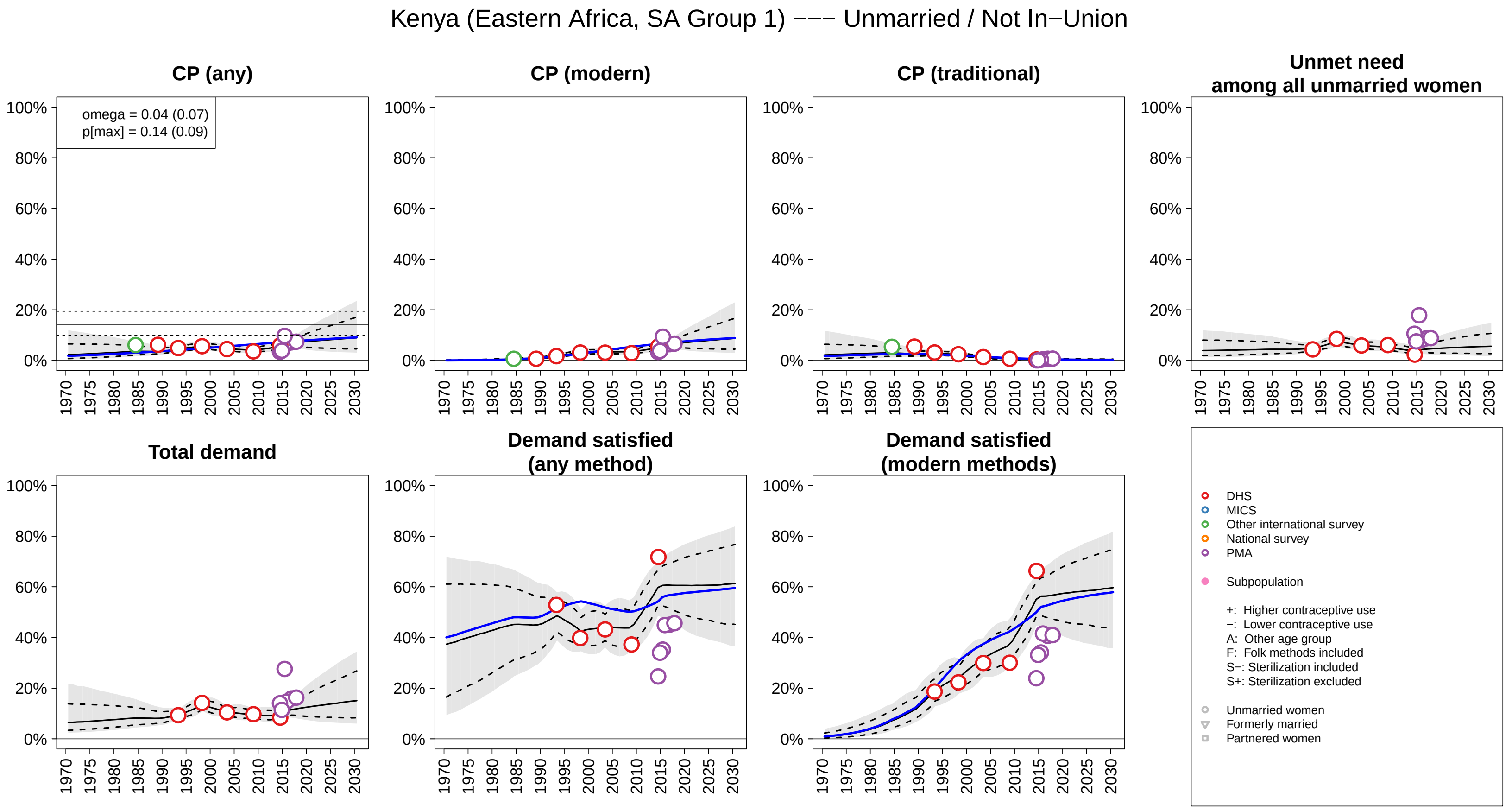

Kenya (Eastern Africa, SA Group 1) −−− Unmarried / Not In−Union
CP (any)
omega = 0.04 (0.07)
p[max] = 0.14 (0.09)
CP (modern)
CP (traditional)
Unmet need among all unmarried women
Total demand
Demand satisfied (any method)
Demand satisfied (modern methods)
DHS
MICS
Other international survey
National survey
PMA
Subpopulation
+: Higher contraceptive use
−: Lower contraceptive use
A: Other age group
F: Folk methods included
S−: Sterilization included
S+: Sterilization excluded
Unmarried women
Formerly married
Partnered women

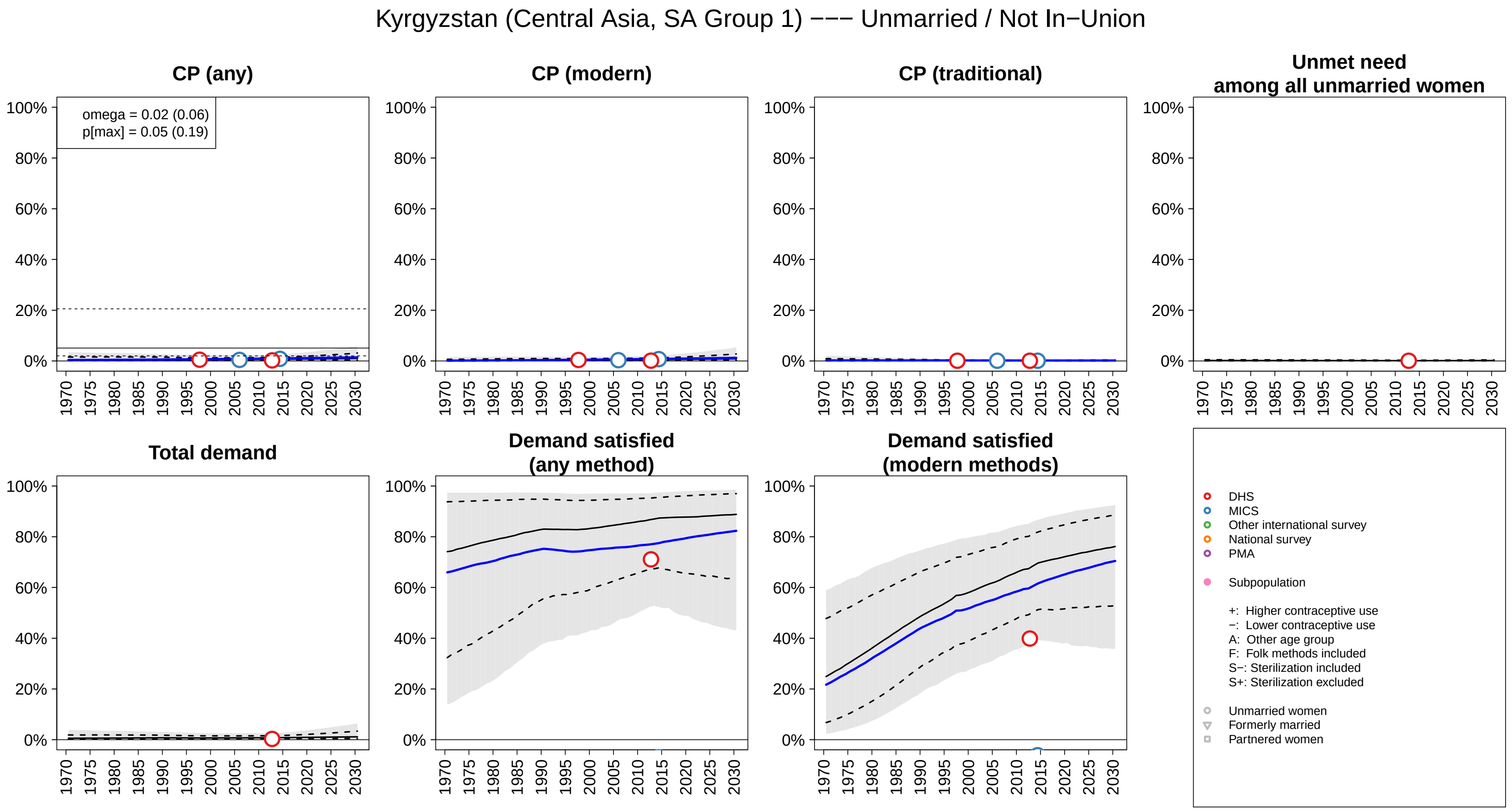
Kyrgyzstan (Central Asia, SA Group 1) −−− Unmarried / Not In−Union
CP (any)
omega = 0.02 (0.06)
p[max] = 0.05 (0.19)
CP (modern)
CP (traditional)
Unmet need among all unmarried women
Total demand
Demand satisfied (any method)
Demand satisfied (modern methods)
0%
20%
40%
60%
80%
100%
1970 1975 1980 1985 1990 1995 2000 2005 2010 2015 2020 2025 2030
DHS
MICS
Other international survey
National survey
PMA
Subpopulation
+: Higher contraceptive use
−: Lower contraceptive use
A: Other age group
F: Folk methods included
S−: Sterilization included
S+: Sterilization excluded
Unmarried women
Formerly married
Partnered women

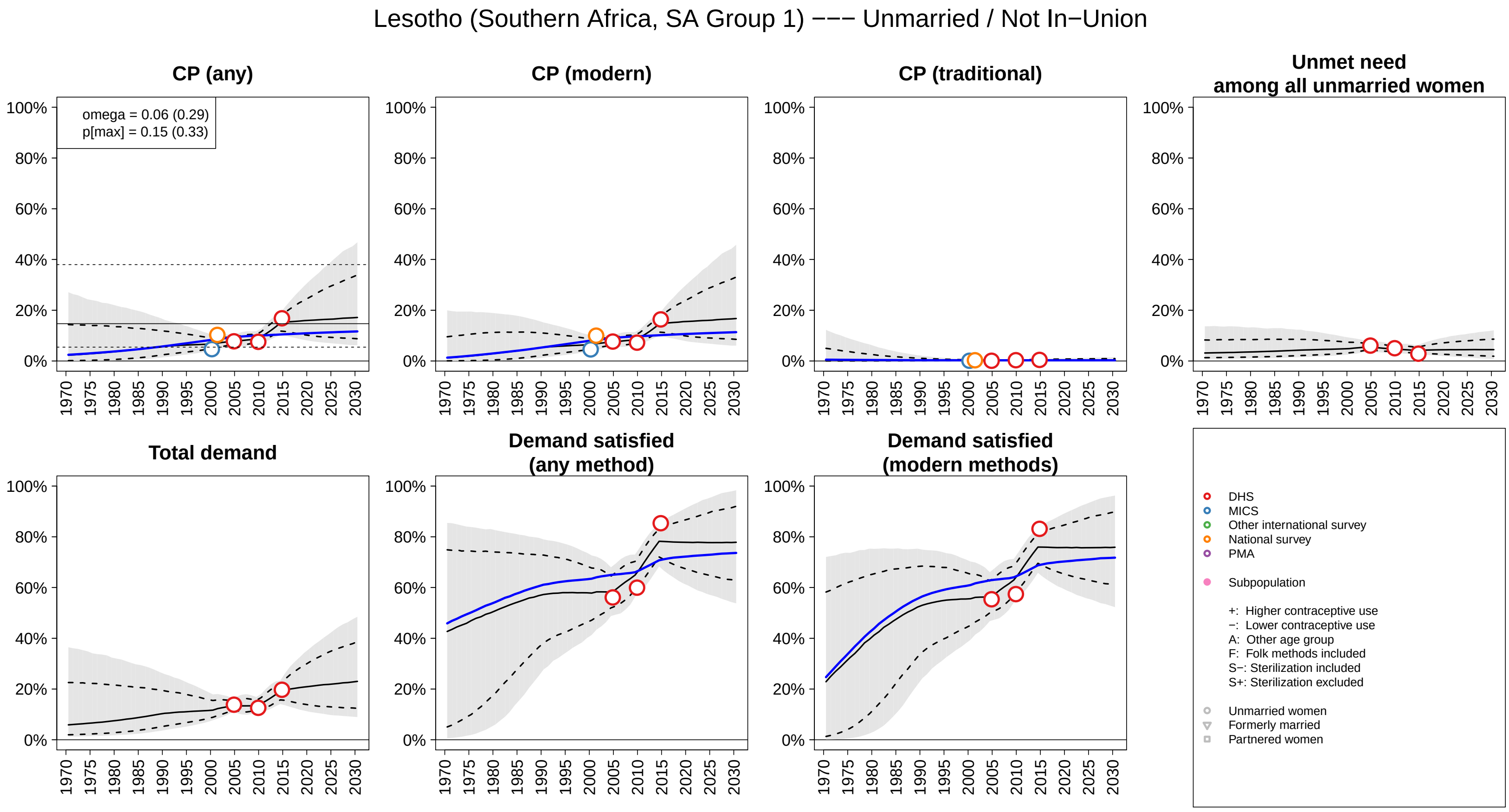
Lesotho (Southern Africa, SA Group 1) −−− Unmarried / Not In−Union
CP (any)
omega = 0.06 (0.29)
p[max] = 0.15 (0.33)
CP (modern)
CP (traditional)
Unmet need among all unmarried women
Total demand
Demand satisfied (any method)
Demand satisfied (modern methods)
DHS
MICS
Other international survey
National survey
PMA
Subpopulation
+: Higher contraceptive use
−: Lower contraceptive use
A: Other age group
F: Folk methods included
S−: Sterilization included
S+: Sterilization excluded
Unmarried women
Formerly married
Partnered women

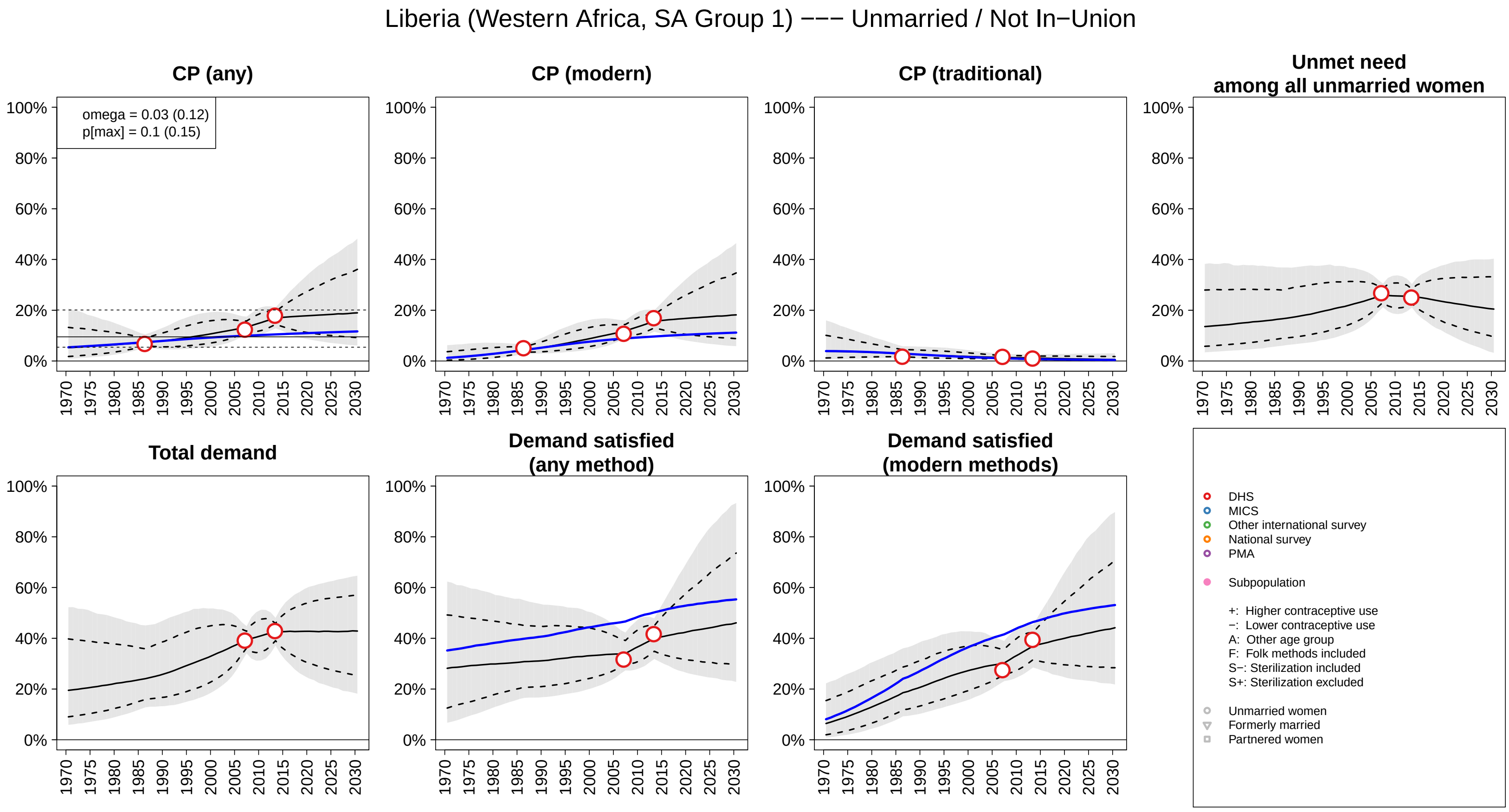
Liberia (Western Africa, SA Group 1) −−− Unmarried / Not In−Union
CP (any)
omega = 0.03 (0.12)
p[max] = 0.1 (0.15)
CP (modern)
CP (traditional)
Unmet need among all unmarried women
Total demand
Demand satisfied (any method)
Demand satisfied (modern methods)
DHS
MICS
Other international survey
National survey
PMA
Subpopulation
+: Higher contraceptive use
−: Lower contraceptive use
A: Other age group
F: Folk methods included
S−: Sterilization included
S+: Sterilization excluded
Unmarried women
Formerly married
Partnered women

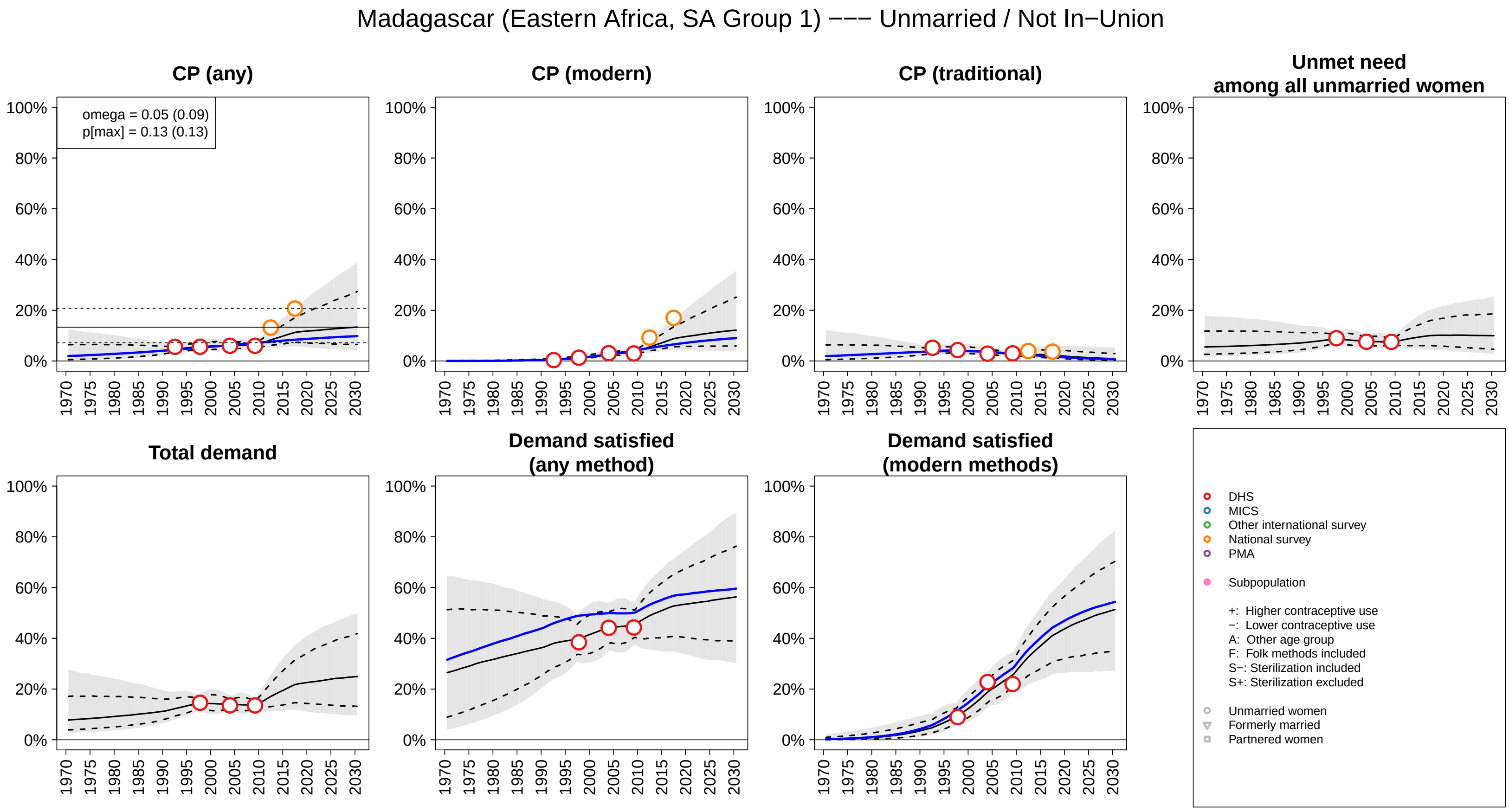
Madagascar (Eastern Africa, SA Group 1) −−− Unmarried / Not In−Union
CP (any)
omega = 0.05 (0.09)
p[max] = 0.13 (0.13)
CP (modern)
CP (traditional)
Unmet need among all unmarried women
Total demand
Demand satisfied (any method)
Demand satisfied (modern methods)
DHS
MICS
Other international survey
National survey
PMA
Subpopulation
+: Higher contraceptive use
−: Lower contraceptive use
A: Other age group
F: Folk methods included
S−: Sterilization included
S+: Sterilization excluded
Unmarried women
Formerly married
Partnered women

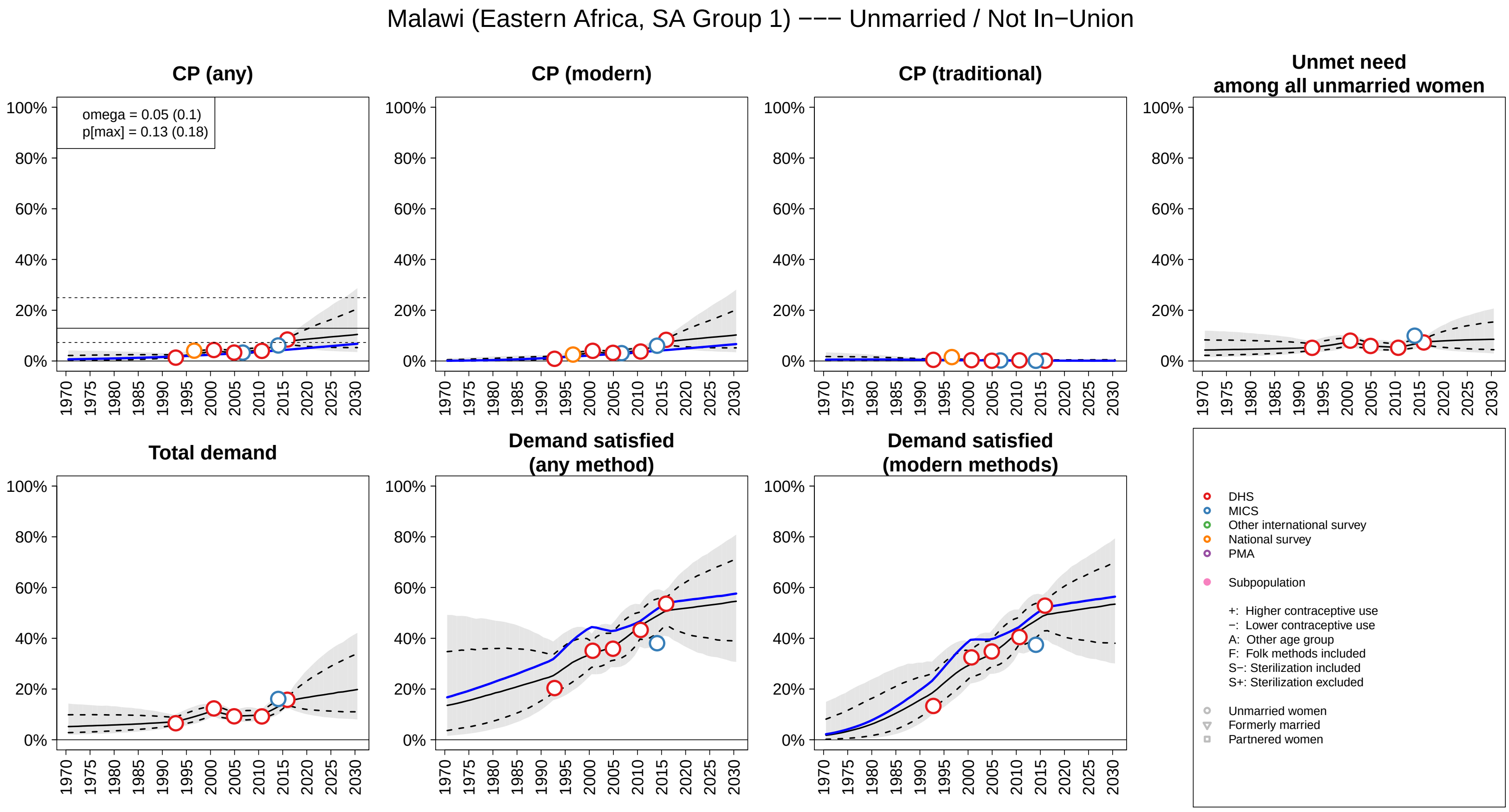

Malawi (Eastern Africa, SA Group 1) −−− Unmarried / Not In−Union
CP (any)
omega = 0.05 (0.1)
p[max] = 0.13 (0.18)
CP (modern)
CP (traditional)
Unmet need among all unmarried women
Total demand
Demand satisfied (any method)
Demand satisfied (modern methods)
DHS
MICS
Other international survey
National survey
PMA
Subpopulation
+: Higher contraceptive use
−: Lower contraceptive use
A: Other age group
F: Folk methods included
S−: Sterilization included
S+: Sterilization excluded
Unmarried women
Formerly married
Partnered women

Mali (Western Africa, SA Group 1) −−− Unmarried / Not In−Union

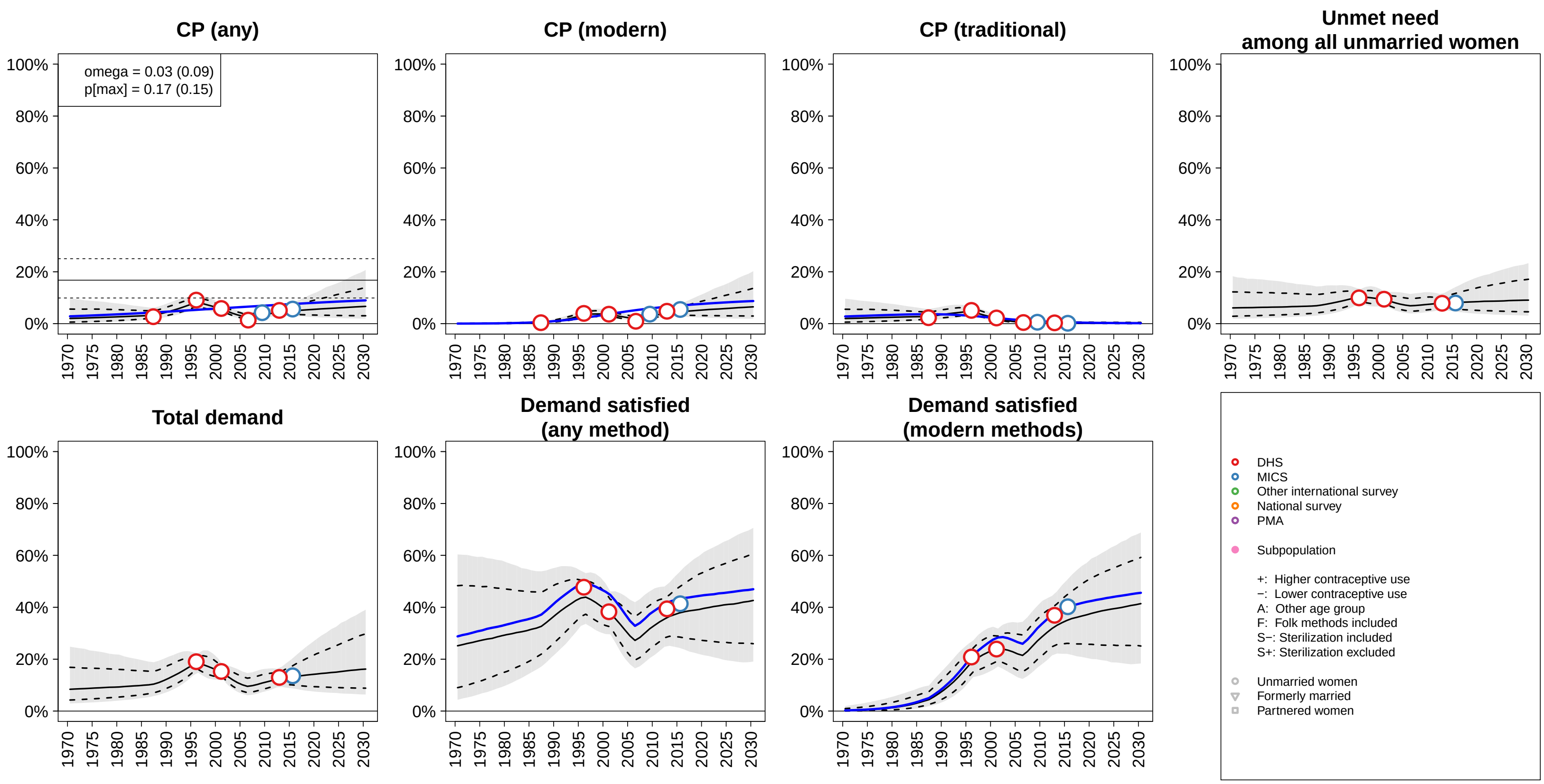

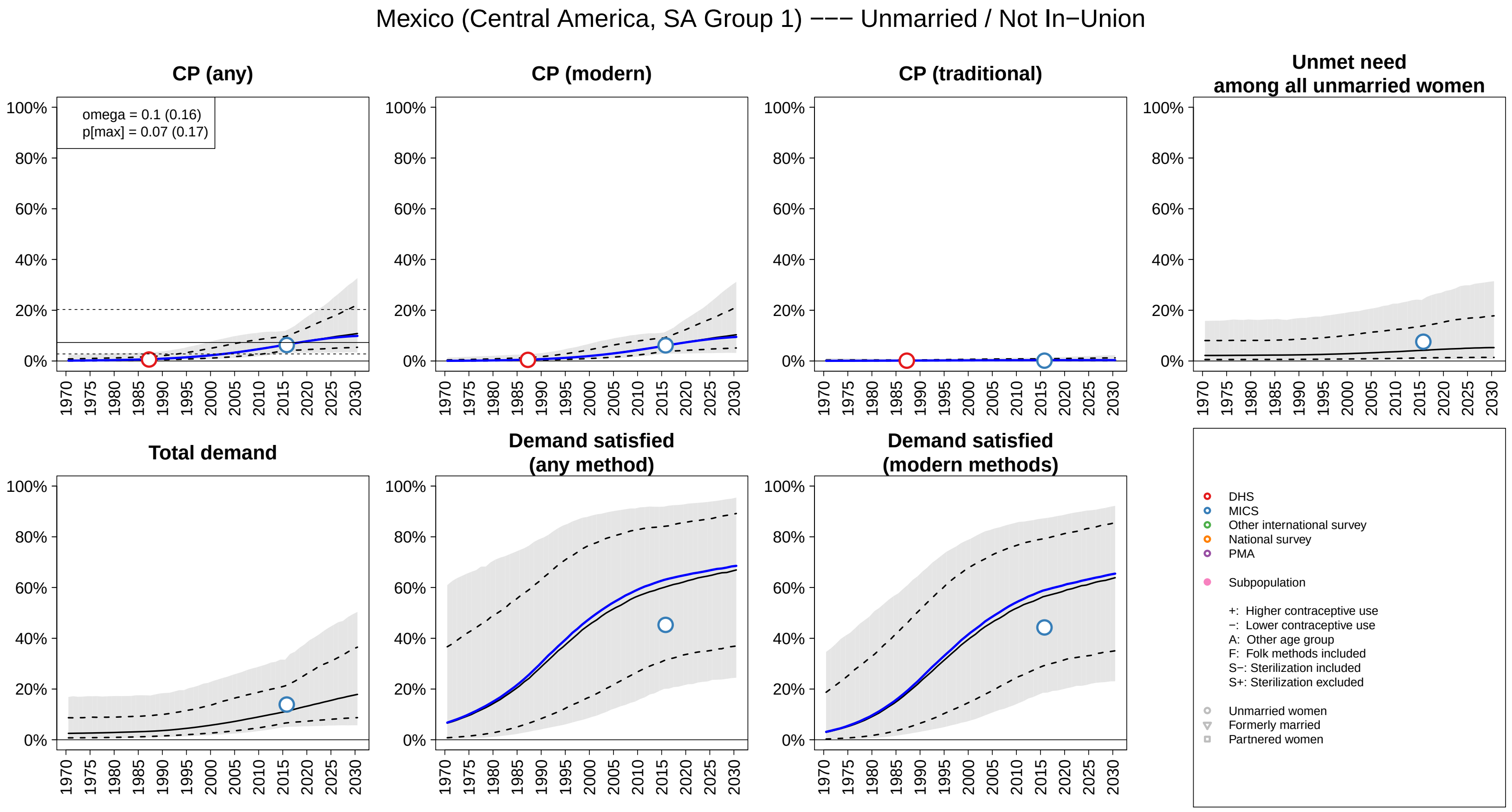
Mexico (Central America, SA Group 1) −−− Unmarried / Not In−Union
CP (any)
omega = 0.1 (0.16)
p[max] = 0.07 (0.17)
CP (modern)
CP (traditional)
Unmet need
among all unmarried women
Total demand
Demand satisfied
(any method)
Demand satisfied
(modern methods)
0%
20%
40%
60%
80%
100%
1970
1975
1980
1985
1990
1995
2000
2005
2010
2015
2020
2025
2030
DHS
MICS
Other international survey
National survey
PMA
Subpopulation
+: Higher contraceptive use
−: Lower contraceptive use
A: Other age group
F: Folk methods included
S−: Sterilization included
S+: Sterilization excluded
Unmarried women
Formerly married
Partnered women

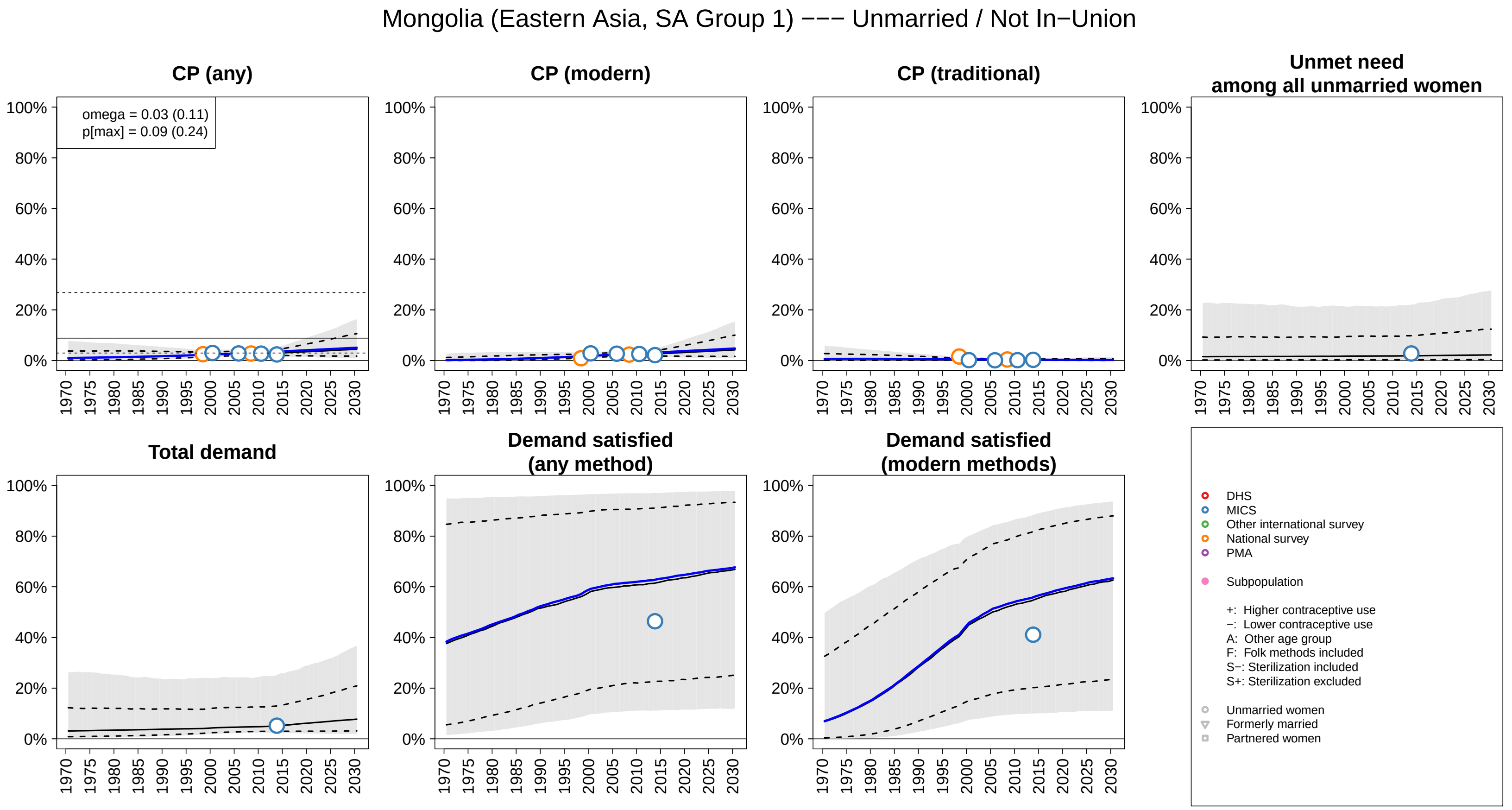
Mongolia (Eastern Asia, SA Group 1) −−− Unmarried / Not In−Union
CP (any)
omega = 0.03 (0.11)
p[max] = 0.09 (0.24)
CP (modern)
CP (traditional)
Unmet need among all unmarried women
Total demand
Demand satisfied (any method)
Demand satisfied (modern methods)
DHS
MICS
Other international survey
National survey
PMA
Subpopulation
+: Higher contraceptive use
−: Lower contraceptive use
A: Other age group
F: Folk methods included
S−: Sterilization included
S+: Sterilization excluded
Unmarried women
Formerly married
Partnered women

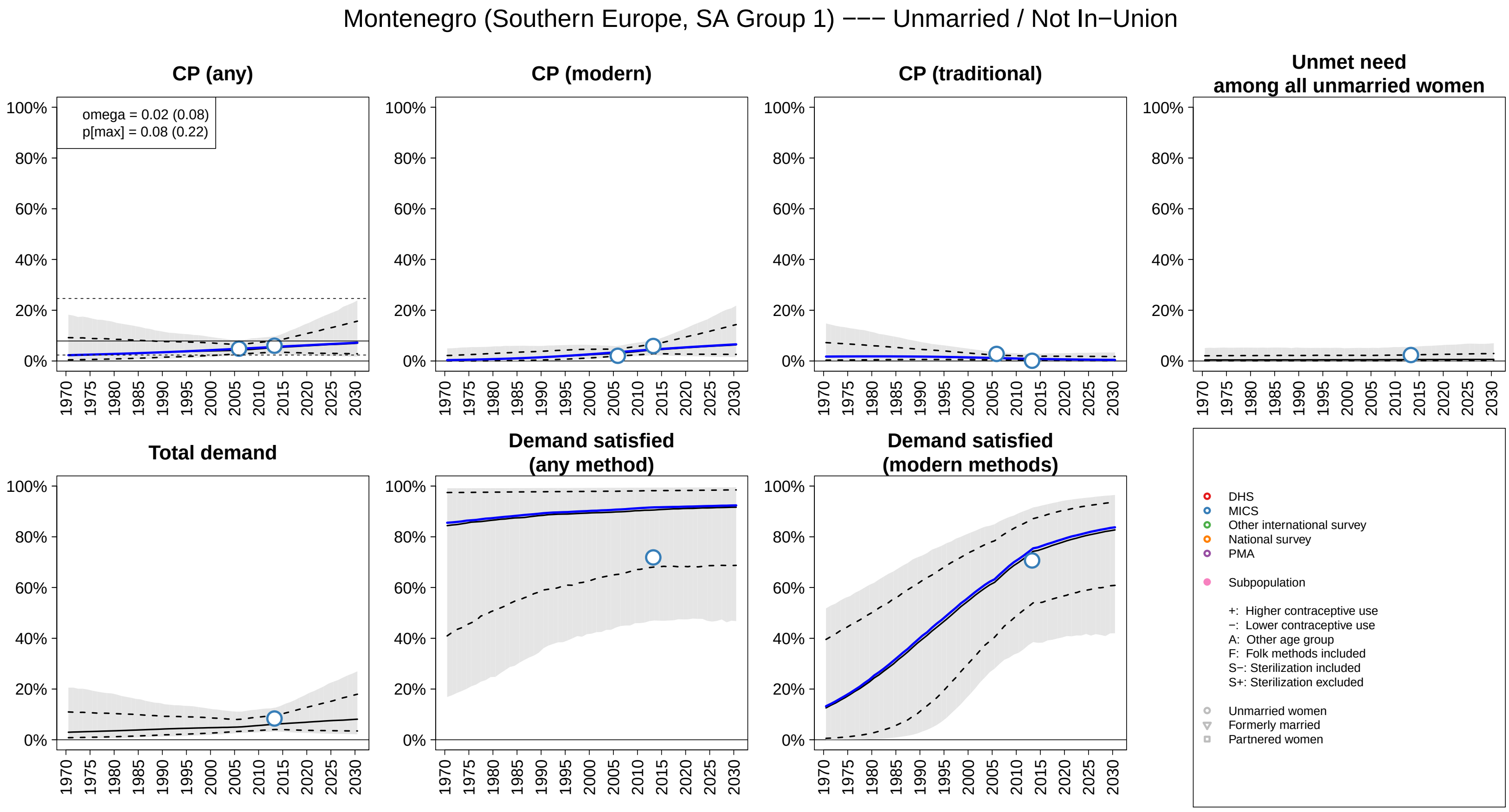
Montenegro (Southern Europe, SA Group 1) −−− Unmarried / Not In−Union
CP (any)
omega = 0.02 (0.08)
p[max] = 0.08 (0.22)
CP (modern)
CP (traditional)
Unmet need among all unmarried women
Total demand
Demand satisfied (any method)
Demand satisfied (modern methods)
DHS
MICS
Other international survey
National survey
PMA
Subpopulation
+: Higher contraceptive use
−: Lower contraceptive use
A: Other age group
F: Folk methods included
S−: Sterilization included
S+: Sterilization excluded
Unmarried women
Formerly married
Partnered women

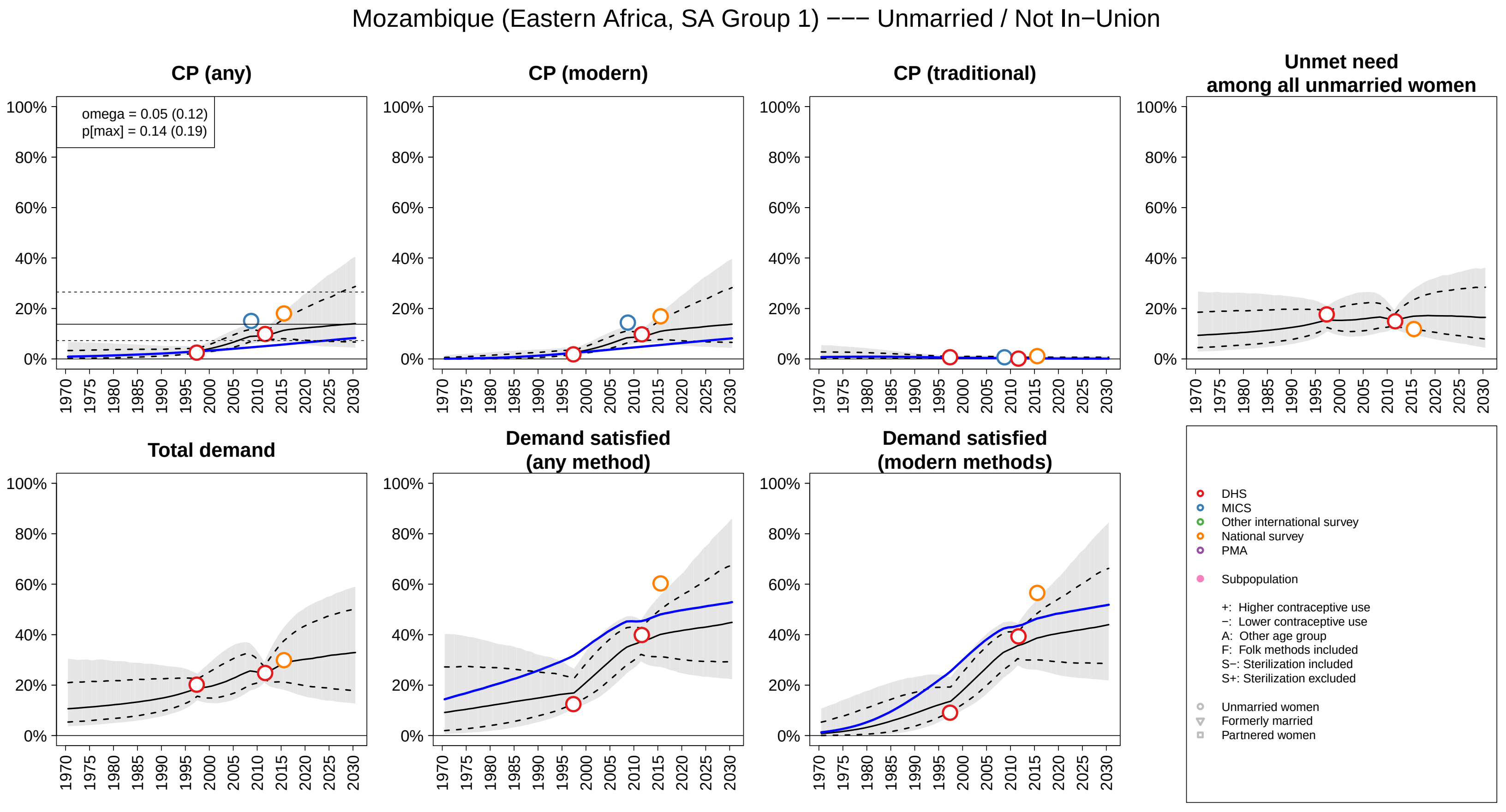
Mozambique (Eastern Africa, SA Group 1) −−− Unmarried / Not In−Union
CP (any)
omega = 0.05 (0.12)
p[max] = 0.14 (0.19)
CP (modern)
CP (traditional)
Unmet need among all unmarried women
Total demand
Demand satisfied (any method)
Demand satisfied (modern methods)
0%
20%
40%
60%
80%
100%
1970
1975
1980
1985
1990
1995
2000
2005
2010
2015
2020
2025
2030
DHS
MICS
Other international survey
National survey
PMA
Subpopulation
+: Higher contraceptive use
−: Lower contraceptive use
A: Other age group
F: Folk methods included
S−: Sterilization included
S+: Sterilization excluded
Unmarried women
Formerly married
Partnered women

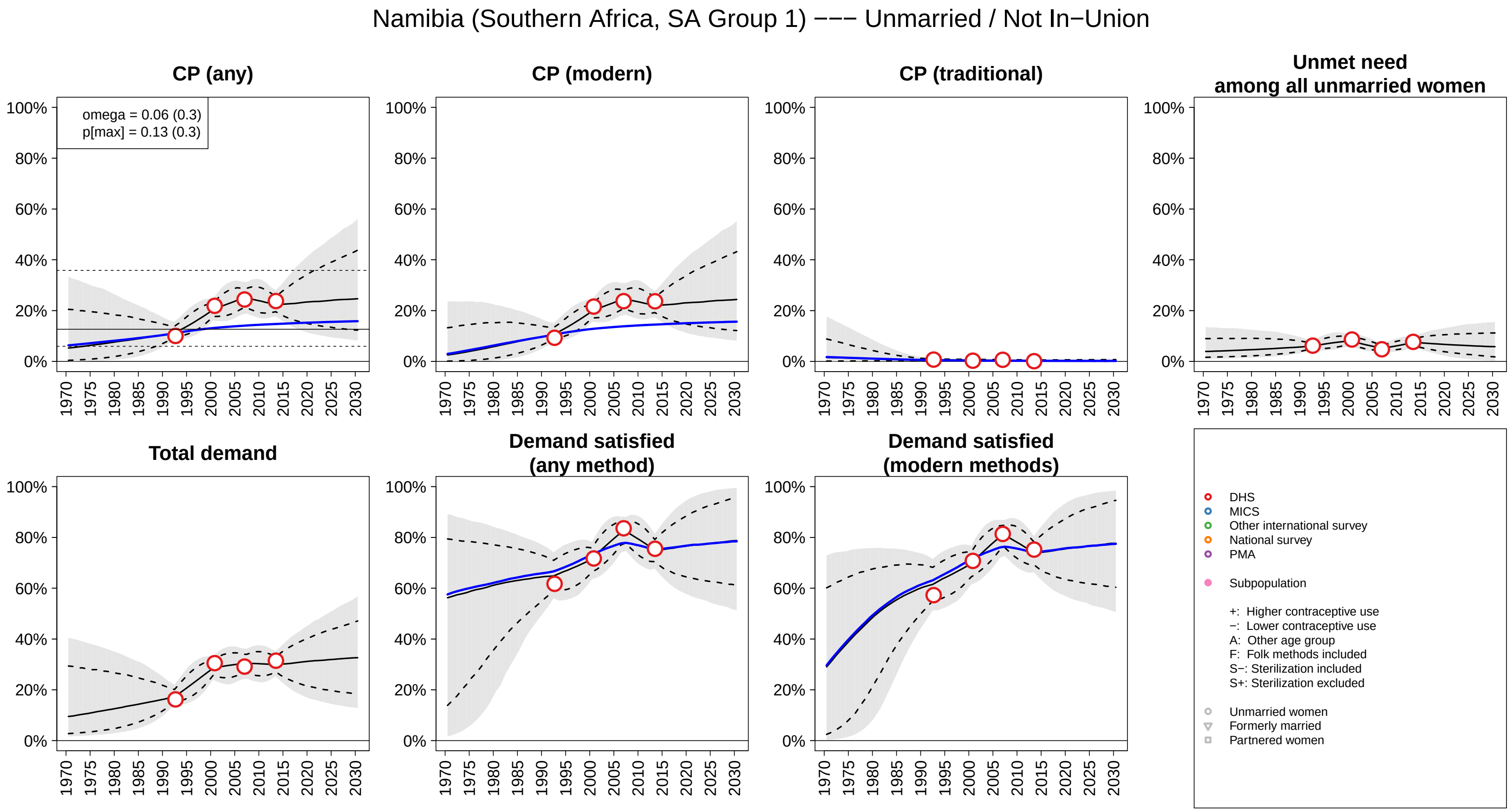

Namibia (Southern Africa, SA Group 1) −−− Unmarried / Not In−Union
CP (any)
omega = 0.06 (0.3)
p[max] = 0.13 (0.3)
CP (modern)
CP (traditional)
Unmet need among all unmarried women
Total demand
Demand satisfied (any method)
Demand satisfied (modern methods)
0%
20%
40%
60%
80%
100%
1970
1975
1980
1985
1990
1995
2000
2005
2010
2015
2020
2025
2030
DHS
MICS
Other international survey
National survey
PMA
Subpopulation
+: Higher contraceptive use
−: Lower contraceptive use
A: Other age group
F: Folk methods included
S−: Sterilization included
S+: Sterilization excluded
Unmarried women
Formerly married
Partnered women

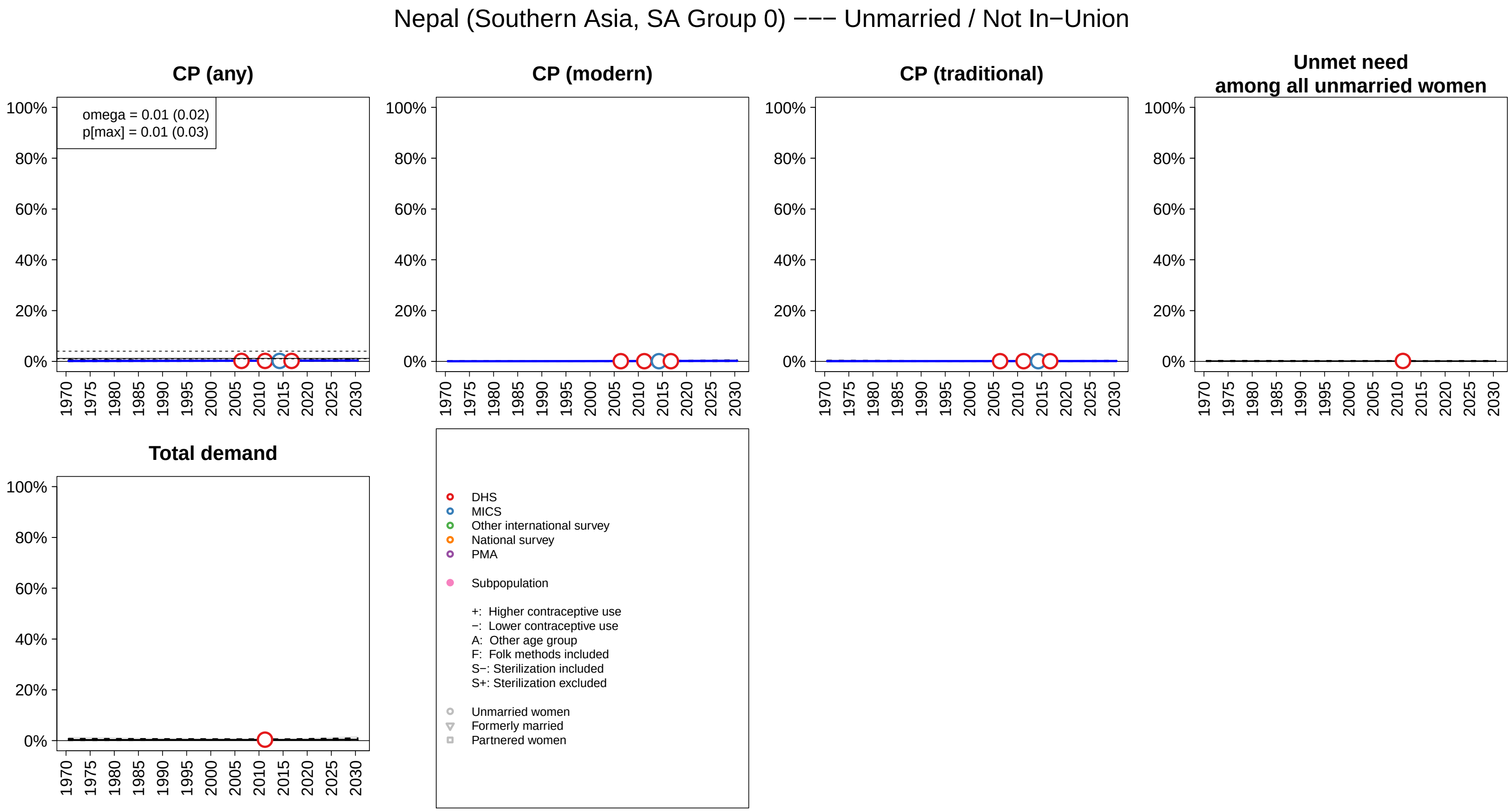
Nepal (Southern Asia, SA Group 0) −−− Unmarried / Not In−Union
CP (any)
omega = 0.01 (0.02)
p[max] = 0.01 (0.03)
CP (modern)
CP (traditional)
Unmet need
among all unmarried women
Total demand
100%
80%
60%
40%
20%
0%
1970
1975
1980
1985
1990
1995
2000
2005
2010
2015
2020
2025
2030
DHS
MICS
Other international survey
National survey
PMA
Subpopulation
+: Higher contraceptive use
−: Lower contraceptive use
A: Other age group
F: Folk methods included
S−: Sterilization included
S+: Sterilization excluded
Unmarried women
Formerly married
Partnered women

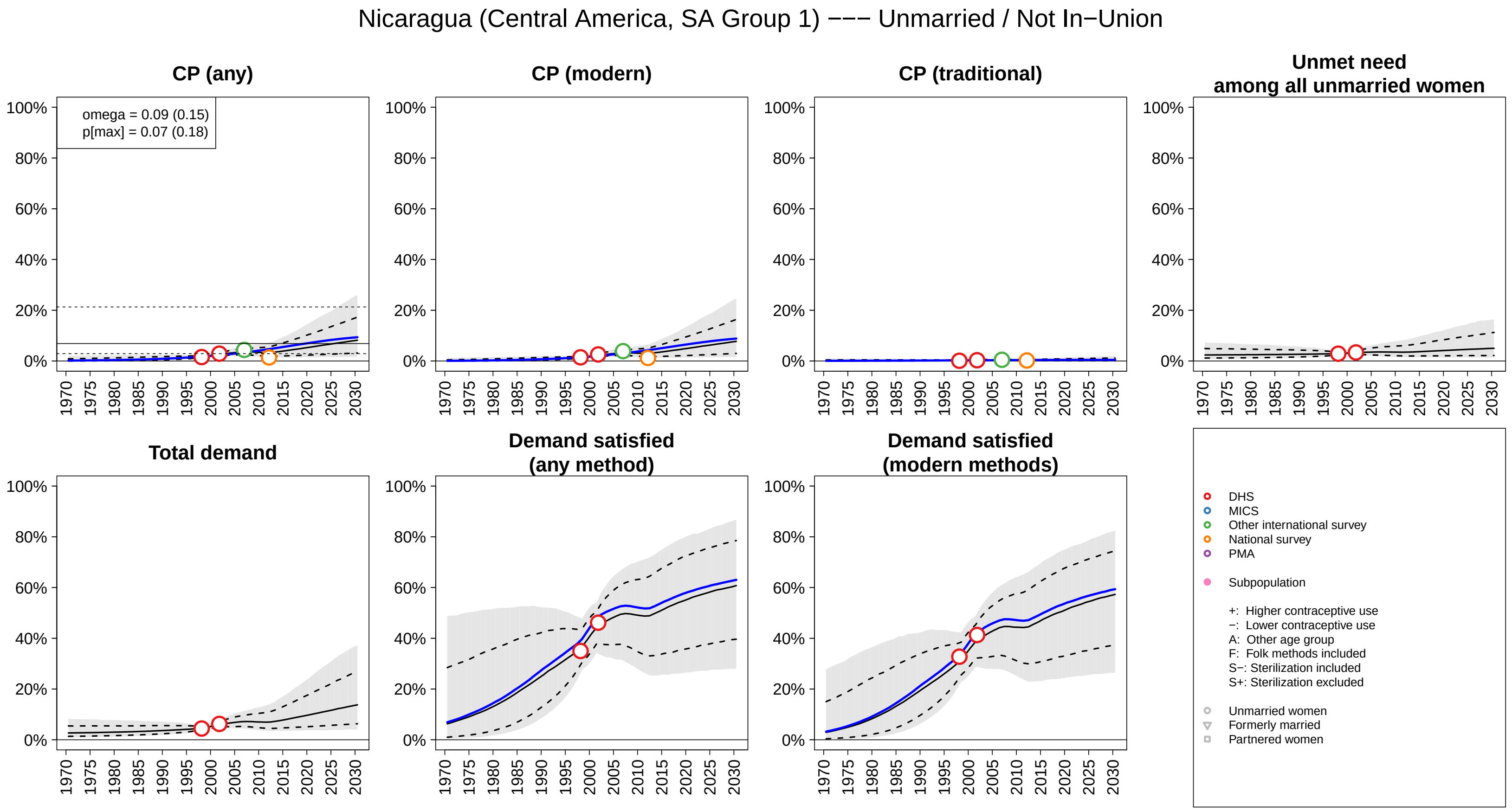
Nicaragua (Central America, SA Group 1) --- Unmarried / Not In−Union
CP (any)
omega = 0.09 (0.15)
p[max] = 0.07 (0.18)
CP (modern)
CP (traditional)
Unmet need among all unmarried women
Total demand
Demand satisfied (any method)
Demand satisfied (modern methods)
DHS
MICS
Other international survey
National survey
PMA
Subpopulation
+: Higher contraceptive use
−: Lower contraceptive use
A: Other age group
F: Folk methods included
S−: Sterilization included
S+: Sterilization excluded
Unmarried women
Formerly married
Partnered women

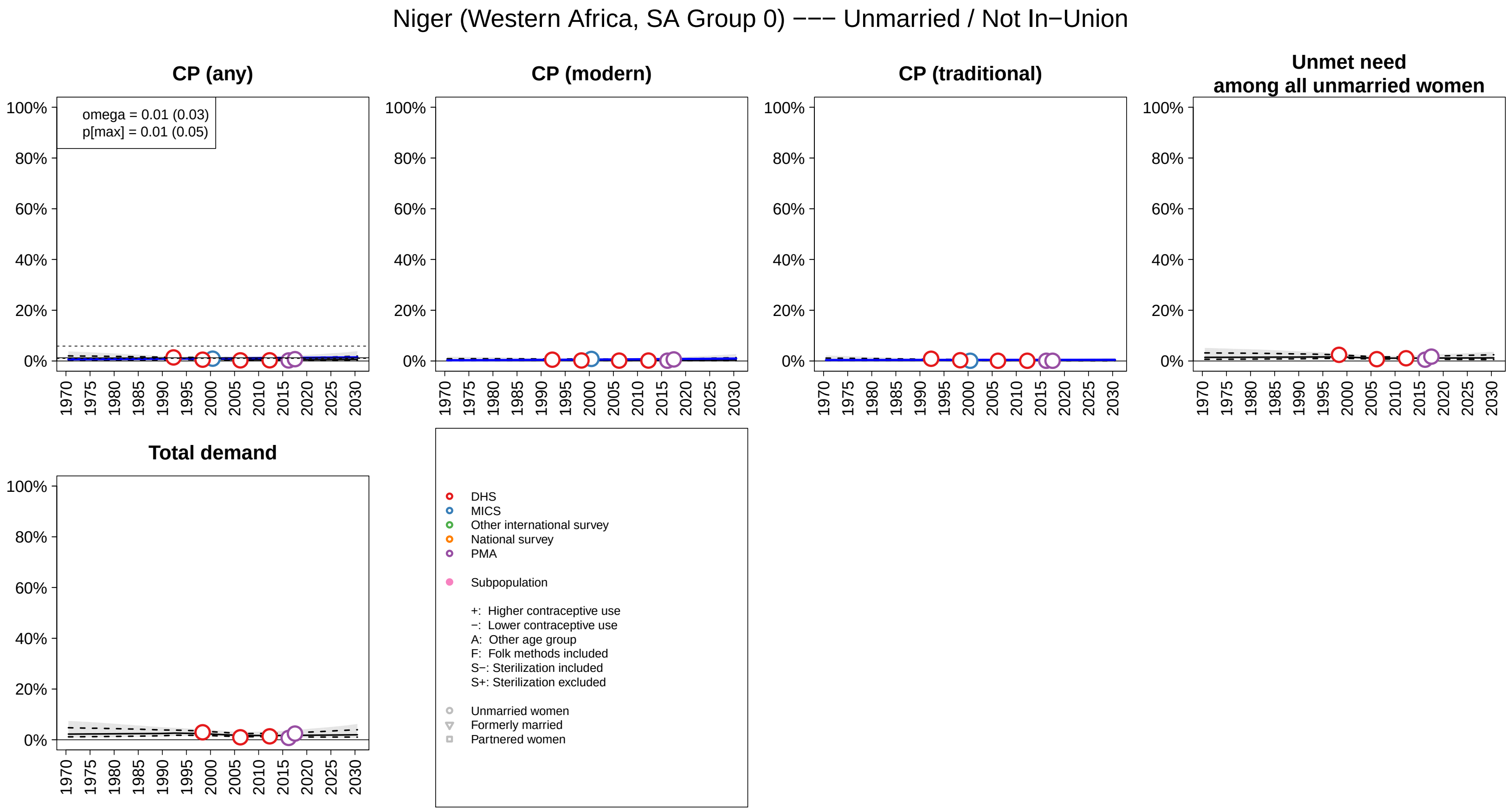
Niger (Western Africa, SA Group 0) ––– Unmarried / Not In–Union
CP (any)
omega = 0.01 (0.03)
p[max] = 0.01 (0.05)
CP (modern)
CP (traditional)
Unmet need among all unmarried women
Total demand
DHS
MICS
Other international survey
National survey
PMA
Subpopulation
+: Higher contraceptive use
−: Lower contraceptive use
A: Other age group
F: Folk methods included
S−: Sterilization included
S+: Sterilization excluded
Unmarried women
Formerly married
Partnered women

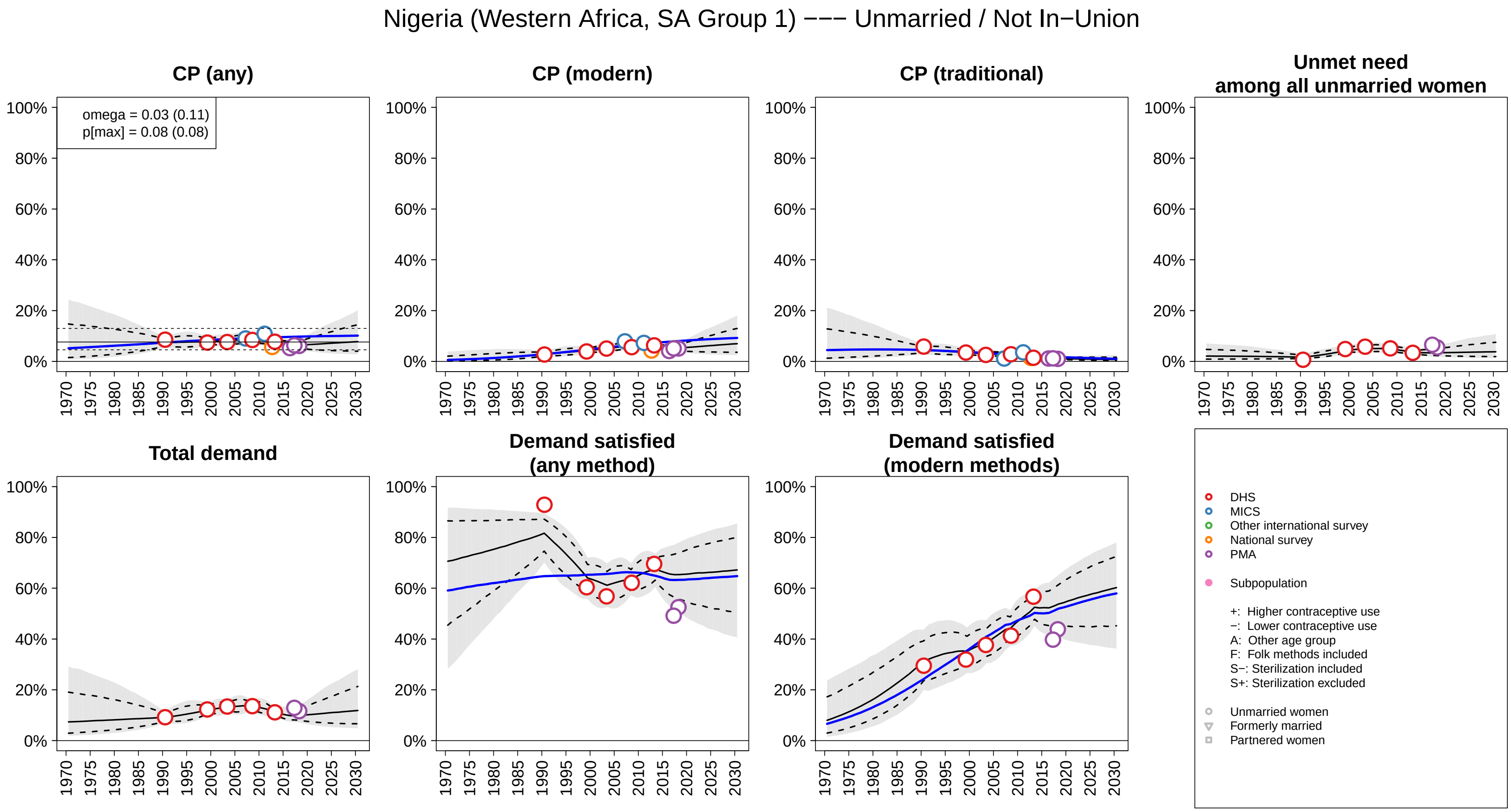

Nigeria (Western Africa, SA Group 1) ––– Unmarried / Not In−Union
CP (any)
omega = 0.03 (0.11)
p[max] = 0.08 (0.08)
CP (modern)
CP (traditional)
Unmet need among all unmarried women
Total demand
Demand satisfied (any method)
Demand satisfied (modern methods)
0%
20%
40%
60%
80%
100%
1970 1975 1980 1985 1990 1995 2000 2005 2010 2015 2020 2025 2030
DHS
MICS
Other international survey
National survey
PMA
Subpopulation
+: Higher contraceptive use
−: Lower contraceptive use
A: Other age group
F: Folk methods included
S−: Sterilization included
S+: Sterilization excluded
Unmarried women
Formerly married
Partnered women

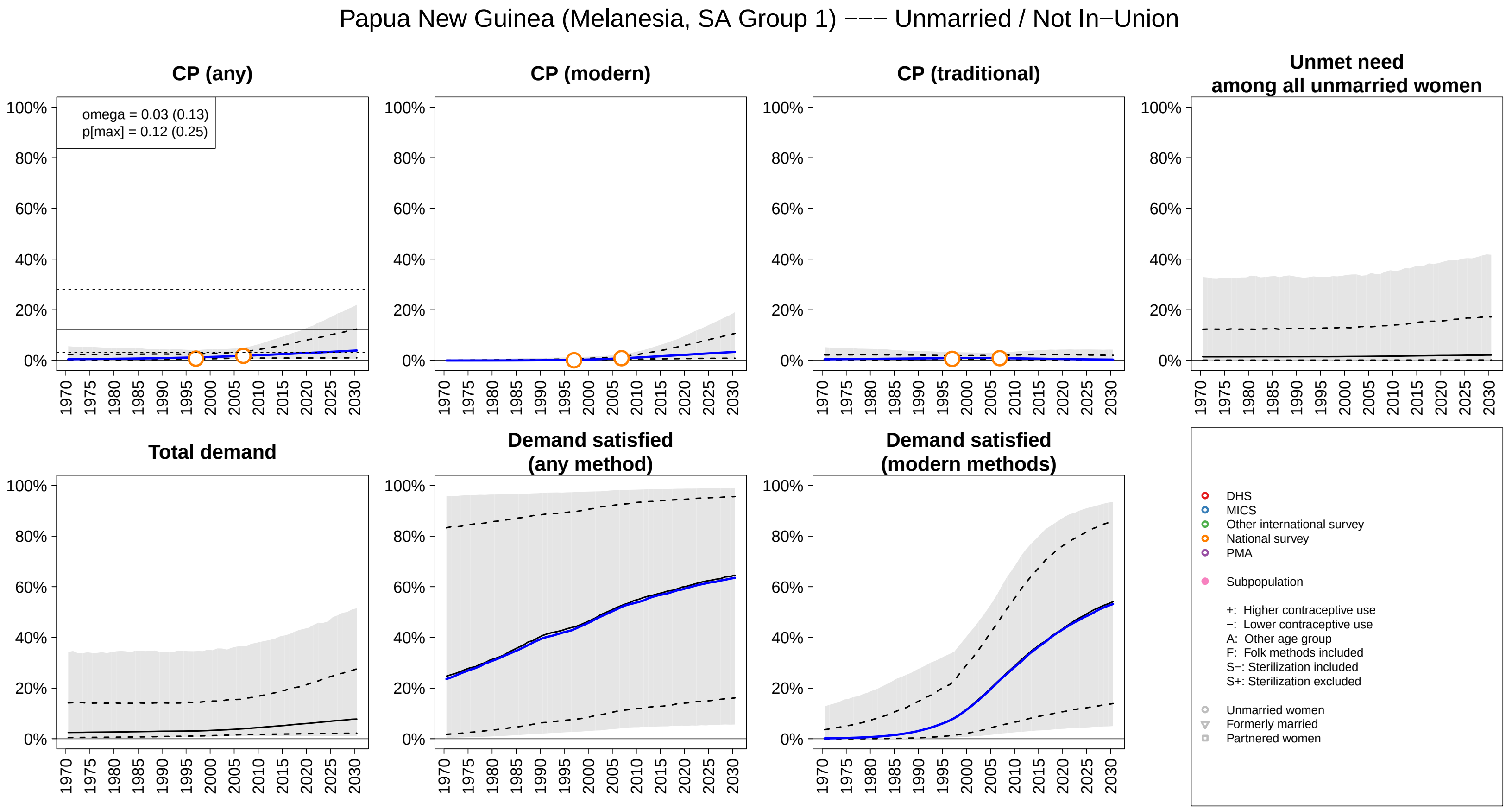

Papua New Guinea (Melanesia, SA Group 1) --- Unmarried / Not In-Union
CP (any)
omega = 0.03 (0.13)
p[max] = 0.12 (0.25)
CP (modern)
CP (traditional)
Unmet need among all unmarried women
Total demand
Demand satisfied (any method)
Demand satisfied (modern methods)
DHS
MICS
Other international survey
National survey
PMA
Subpopulation
+: Higher contraceptive use
−: Lower contraceptive use
A: Other age group
F: Folk methods included
S−: Sterilization included
S+: Sterilization excluded
Unmarried women
Formerly married
Partnered women

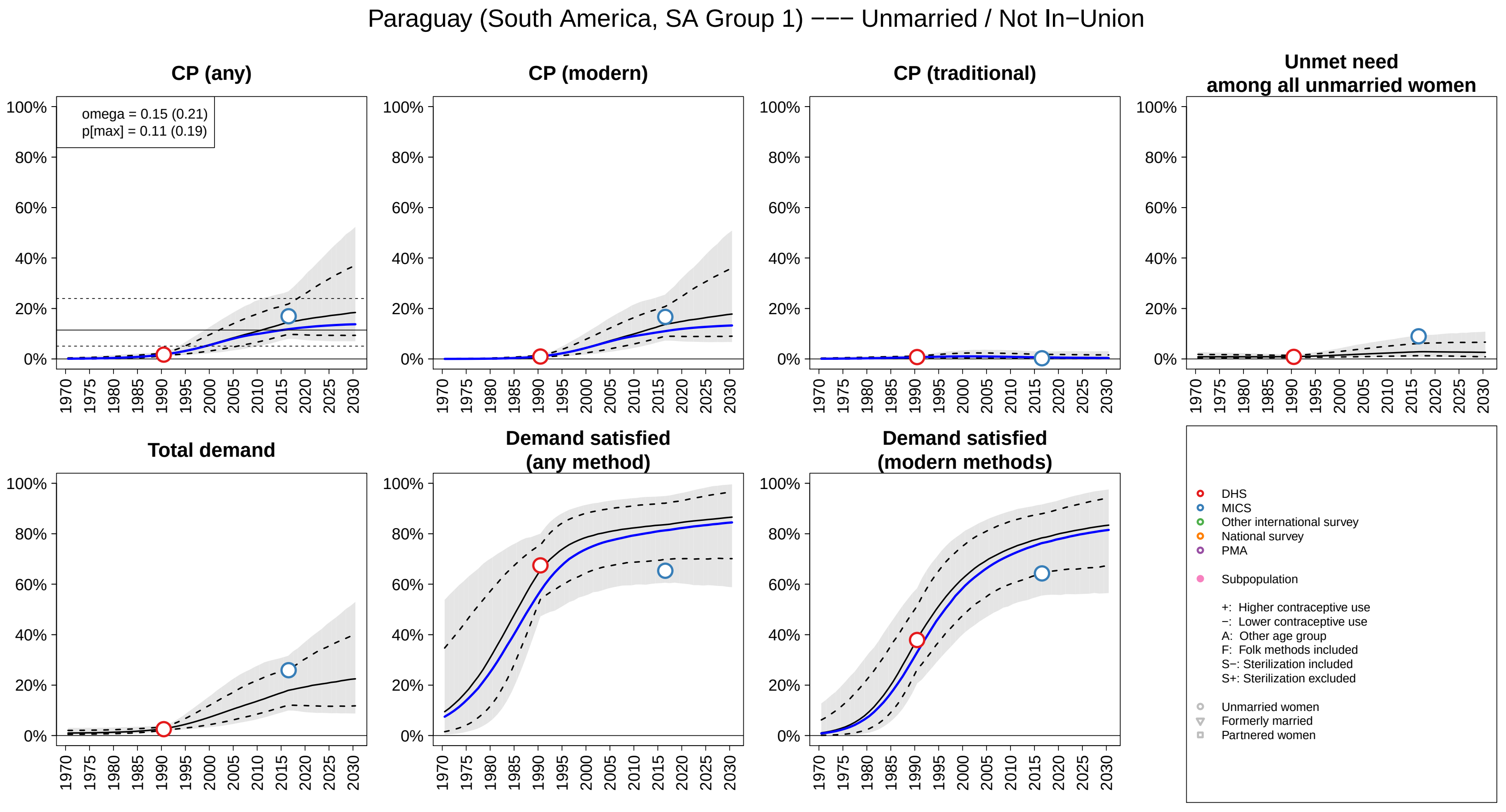
Paraguay (South America, SA Group 1) −−− Unmarried / Not In−Union
CP (any)
omega = 0.15 (0.21)
p[max] = 0.11 (0.19)
CP (modern)
CP (traditional)
Unmet need among all unmarried women
Total demand
Demand satisfied (any method)
Demand satisfied (modern methods)
DHS
MICS
Other international survey
National survey
PMA
Subpopulation
+: Higher contraceptive use
−: Lower contraceptive use
A: Other age group
F: Folk methods included
S−: Sterilization included
S+: Sterilization excluded
Unmarried women
Formerly married
Partnered women

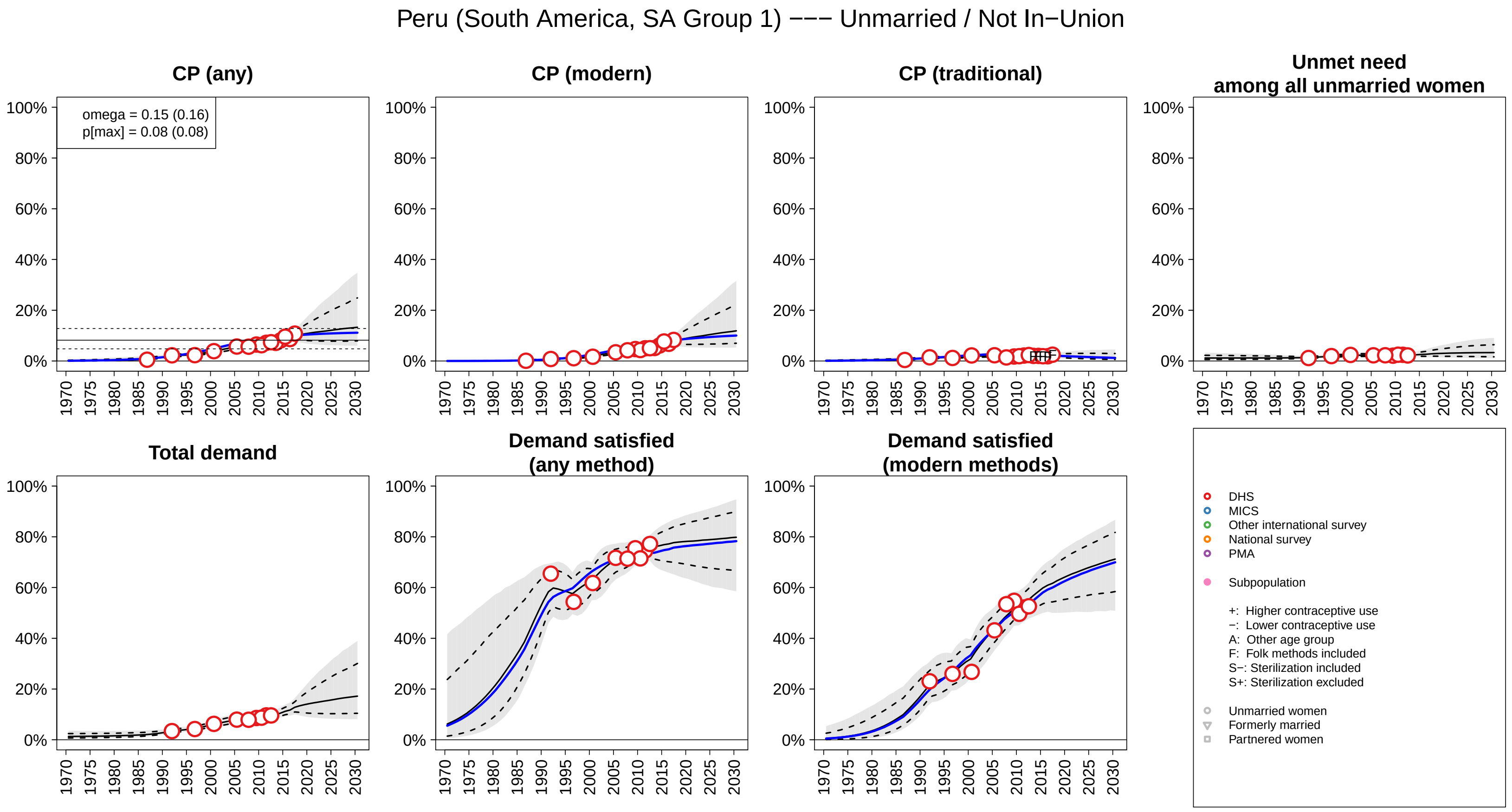
Peru (South America, SA Group 1) --- Unmarried / Not In-Union
CP (any)
omega = 0.15 (0.16)
p[max] = 0.08 (0.08)
CP (modern)
CP (traditional)
Unmet need among all unmarried women
Total demand
Demand satisfied (any method)
Demand satisfied (modern methods)
DHS
MICS
Other international survey
National survey
PMA
Subpopulation
+: Higher contraceptive use
−: Lower contraceptive use
A: Other age group
F: Folk methods included
S−: Sterilization included
S+: Sterilization excluded
Unmarried women
Formerly married
Partnered women

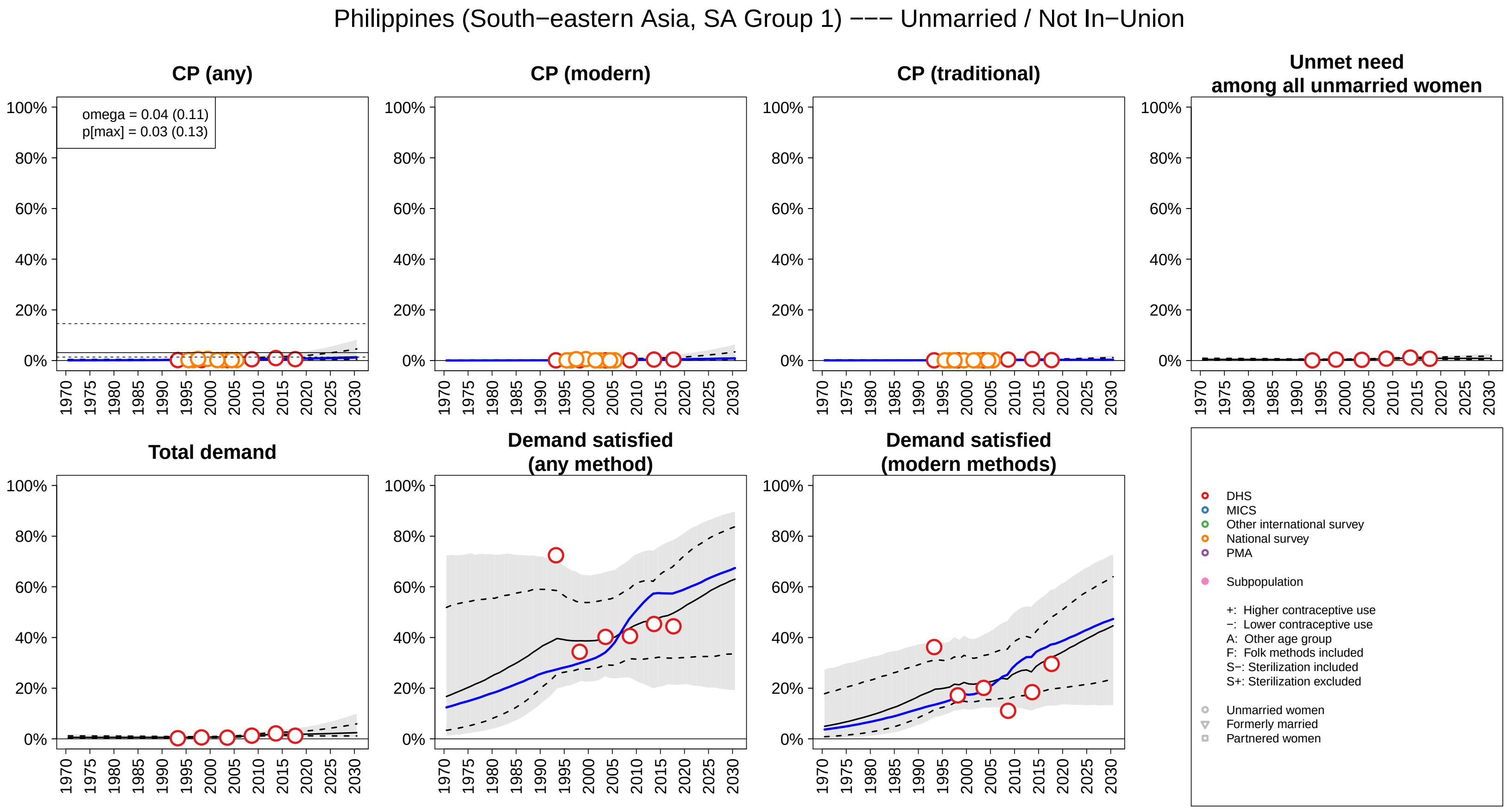

Philippines (South−eastern Asia, SA Group 1) −−− Unmarried / Not In−Union
CP (any)
omega = 0.04 (0.11)
p[max] = 0.03 (0.13)
CP (modern)
CP (traditional)
Unmet need among all unmarried women
Total demand
Demand satisfied (any method)
Demand satisfied (modern methods)
100%
80%
60%
40%
20%
0%
1970 1975 1980 1985 1990 1995 2000 2005 2010 2015 2020 2025 2030
DHS
MICS
Other international survey
National survey
PMA
Subpopulation
+: Higher contraceptive use
−: Lower contraceptive use
A: Other age group
F: Folk methods included
S−: Sterilization included
S+: Sterilization excluded
Unmarried women
Formerly married
Partnered women

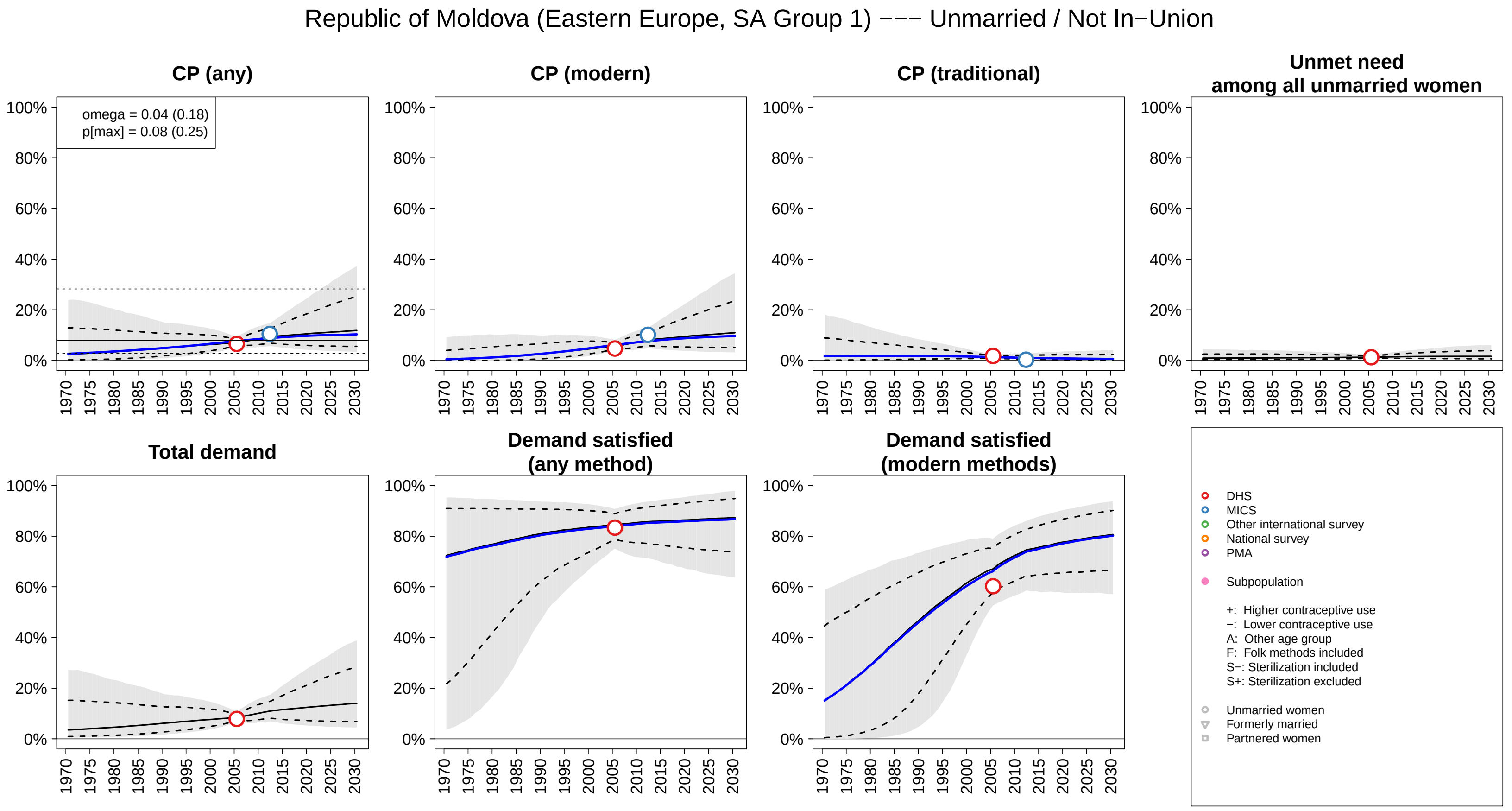
Republic of Moldova (Eastern Europe, SA Group 1) −−− Unmarried / Not In−Union
CP (any)
omega = 0.04 (0.18)
p[max] = 0.08 (0.25)
CP (modern)
CP (traditional)
Unmet need among all unmarried women
Total demand
Demand satisfied (any method)
Demand satisfied (modern methods)
DHS
MICS
Other international survey
National survey
PMA
Subpopulation
+: Higher contraceptive use
−: Lower contraceptive use
A: Other age group
F: Folk methods included
S−: Sterilization included
S+: Sterilization excluded
Unmarried women
Formerly married
Partnered women

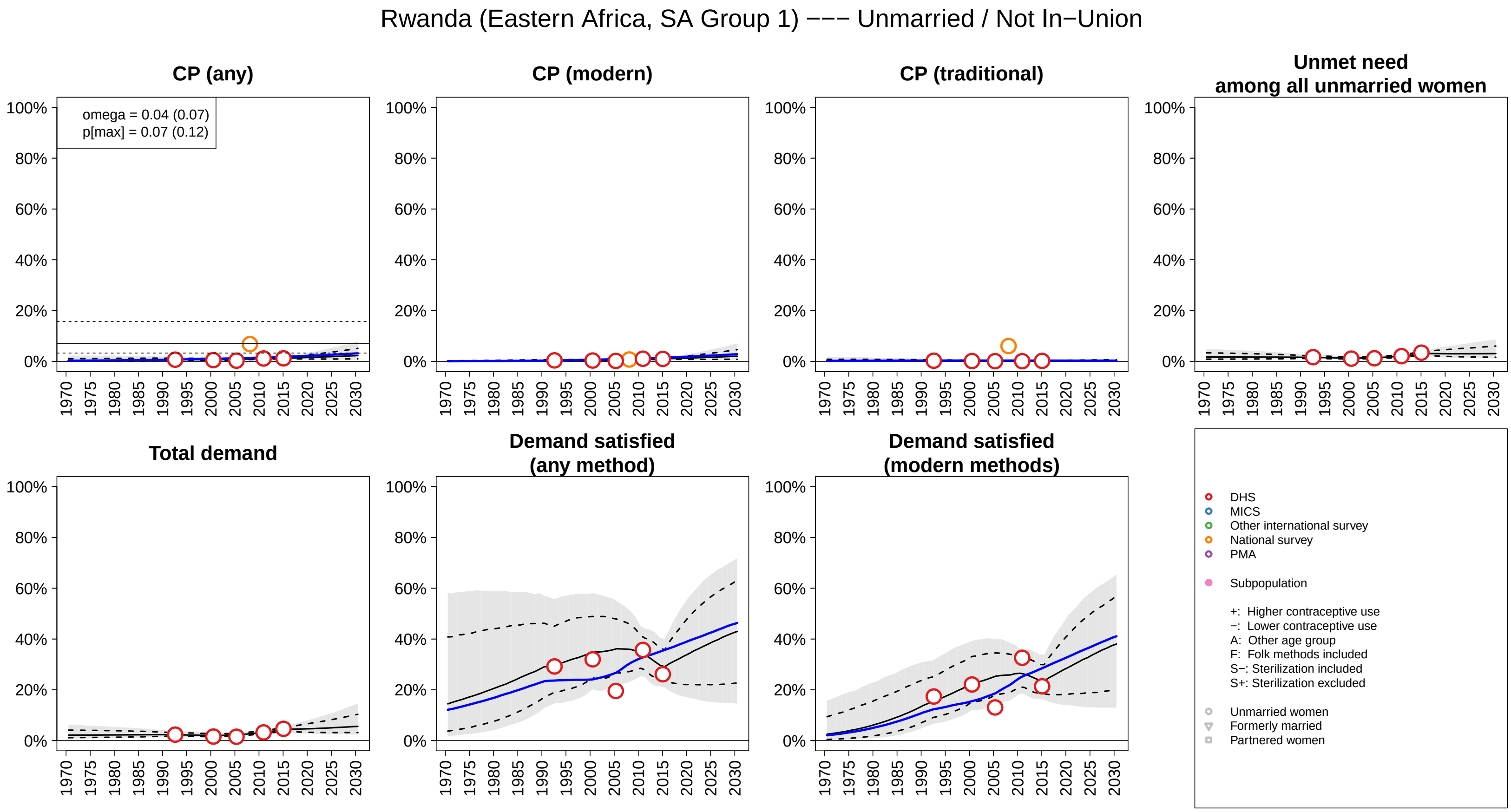

Rwanda (Eastern Africa, SA Group 1) ––– Unmarried / Not In−Union
CP (any)
omega = 0.04 (0.07)
p[max] = 0.07 (0.12)
CP (modern)
CP (traditional)
Unmet need among all unmarried women
Total demand
Demand satisfied (any method)
Demand satisfied (modern methods)
DHS
MICS
Other international survey
National survey
PMA
Subpopulation
+: Higher contraceptive use
−: Lower contraceptive use
A: Other age group
F: Folk methods included
S−: Sterilization included
S+: Sterilization excluded
Unmarried women
Formerly married
Partnered women

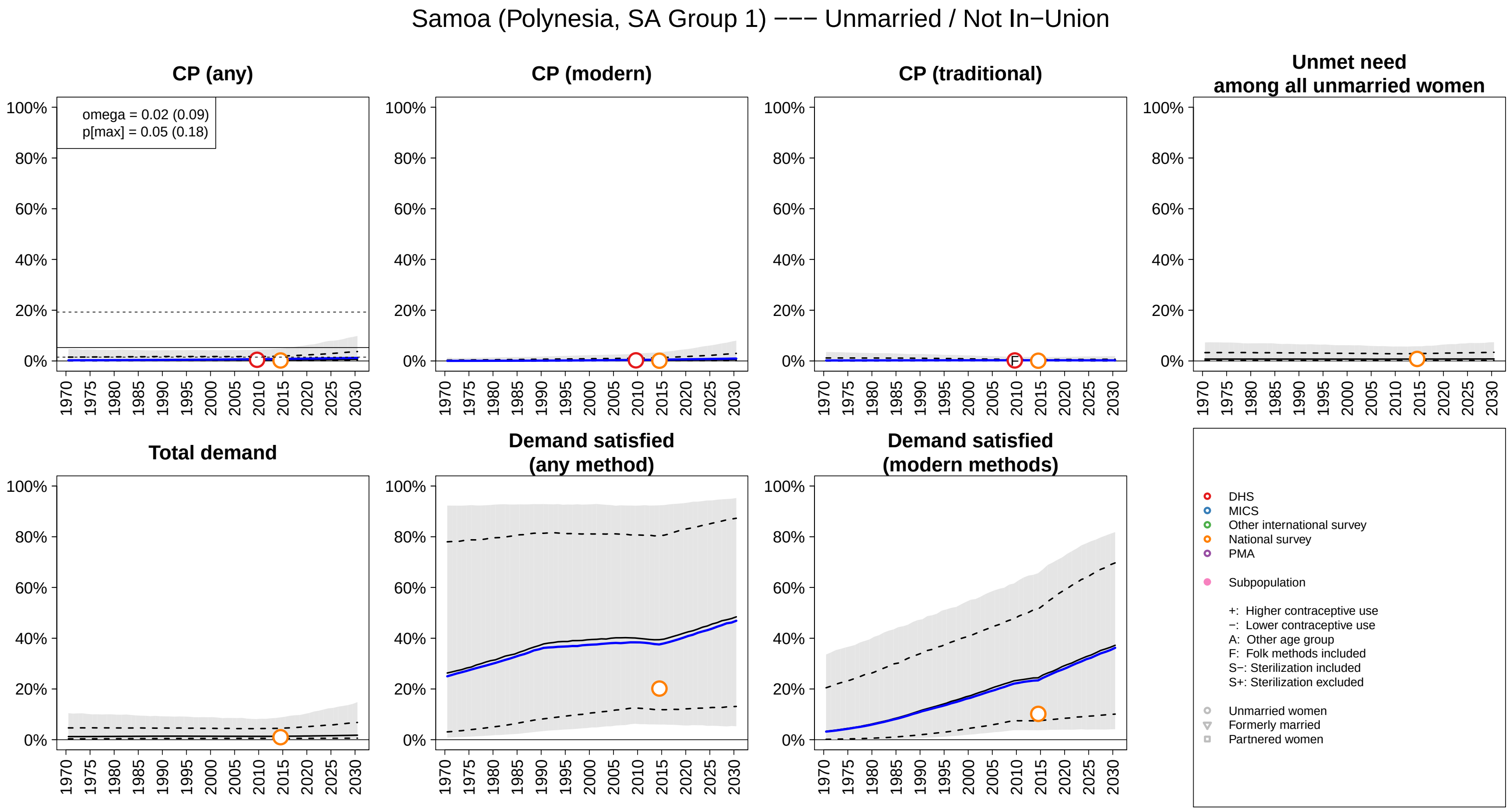
Samoa (Polynesia, SA Group 1) −−− Unmarried / Not In−Union
CP (any)
omega = 0.02 (0.09)
p[max] = 0.05 (0.18)
CP (modern)
CP (traditional)
Unmet need among all unmarried women
Total demand
Demand satisfied (any method)
Demand satisfied (modern methods)
100%
80%
60%
40%
20%
0%
1970 1975 1980 1985 1990 1995 2000 2005 2010 2015 2020 2025 2030
DHS
MICS
Other international survey
National survey
PMA
Subpopulation
+: Higher contraceptive use
−: Lower contraceptive use
A: Other age group
F: Folk methods included
S−: Sterilization included
S+: Sterilization excluded
Unmarried women
Formerly married
Partnered women

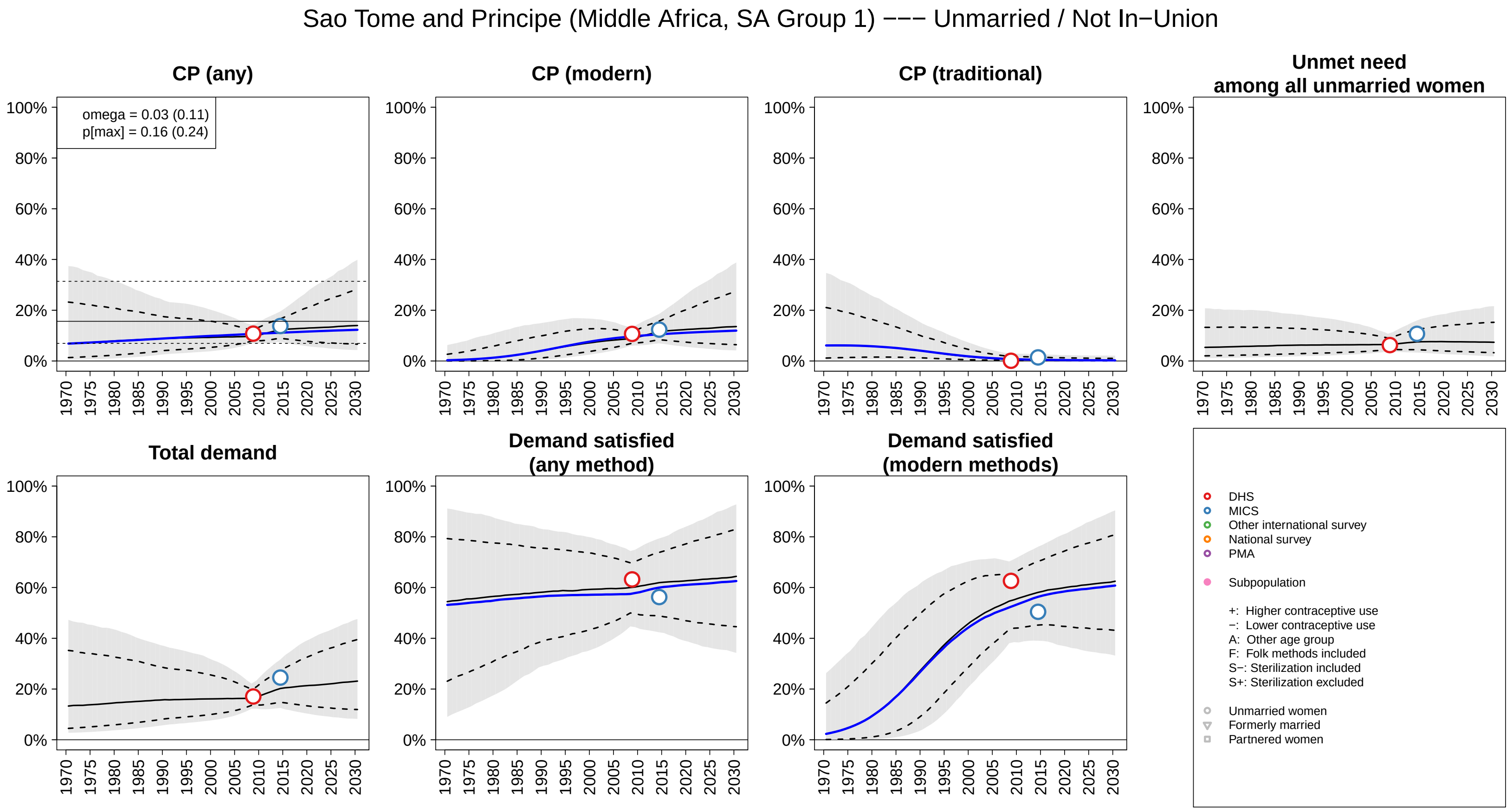
Sao Tome and Principe (Middle Africa, SA Group 1) −−− Unmarried / Not In−Union
CP (any)
omega = 0.03 (0.11)
p[max] = 0.16 (0.24)
CP (modern)
CP (traditional)
Unmet need among all unmarried women
Total demand
Demand satisfied (any method)
Demand satisfied (modern methods)
DHS
MICS
Other international survey
National survey
PMA
Subpopulation
+: Higher contraceptive use
−: Lower contraceptive use
A: Other age group
F: Folk methods included
S−: Sterilization included
S+: Sterilization excluded
Unmarried women
Formerly married
Partnered women

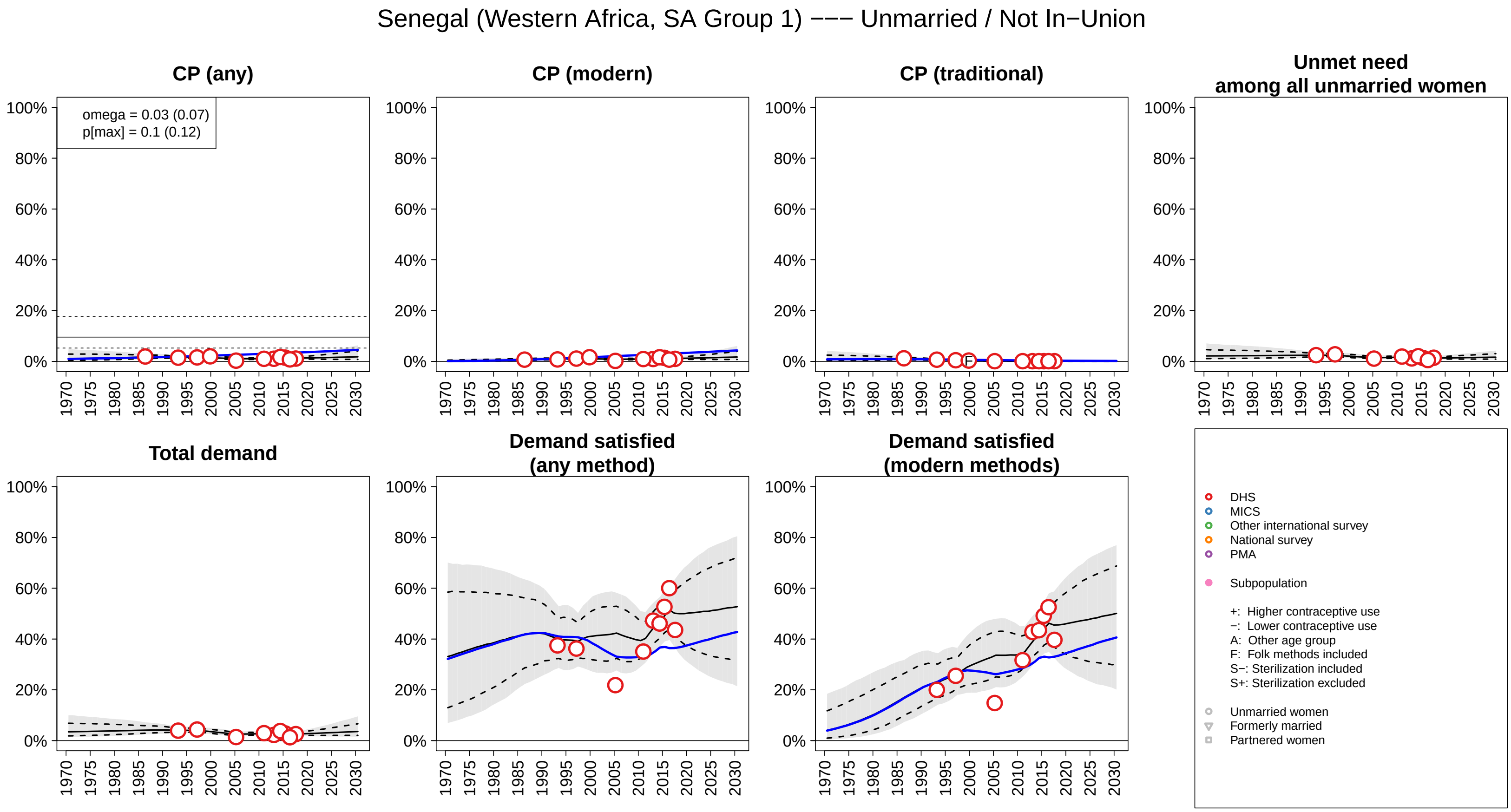
Senegal (Western Africa, SA Group 1) −−− Unmarried / Not In−Union
CP (any)
omega = 0.03 (0.07)
p[max] = 0.1 (0.12)
CP (modern)
CP (traditional)
Unmet need
among all unmarried women
Total demand
Demand satisfied
(any method)
Demand satisfied
(modern methods)
DHS
MICS
Other international survey
National survey
PMA
Subpopulation
+: Higher contraceptive use
−: Lower contraceptive use
A: Other age group
F: Folk methods included
S−: Sterilization included
S+: Sterilization excluded
Unmarried women
Formerly married
Partnered women

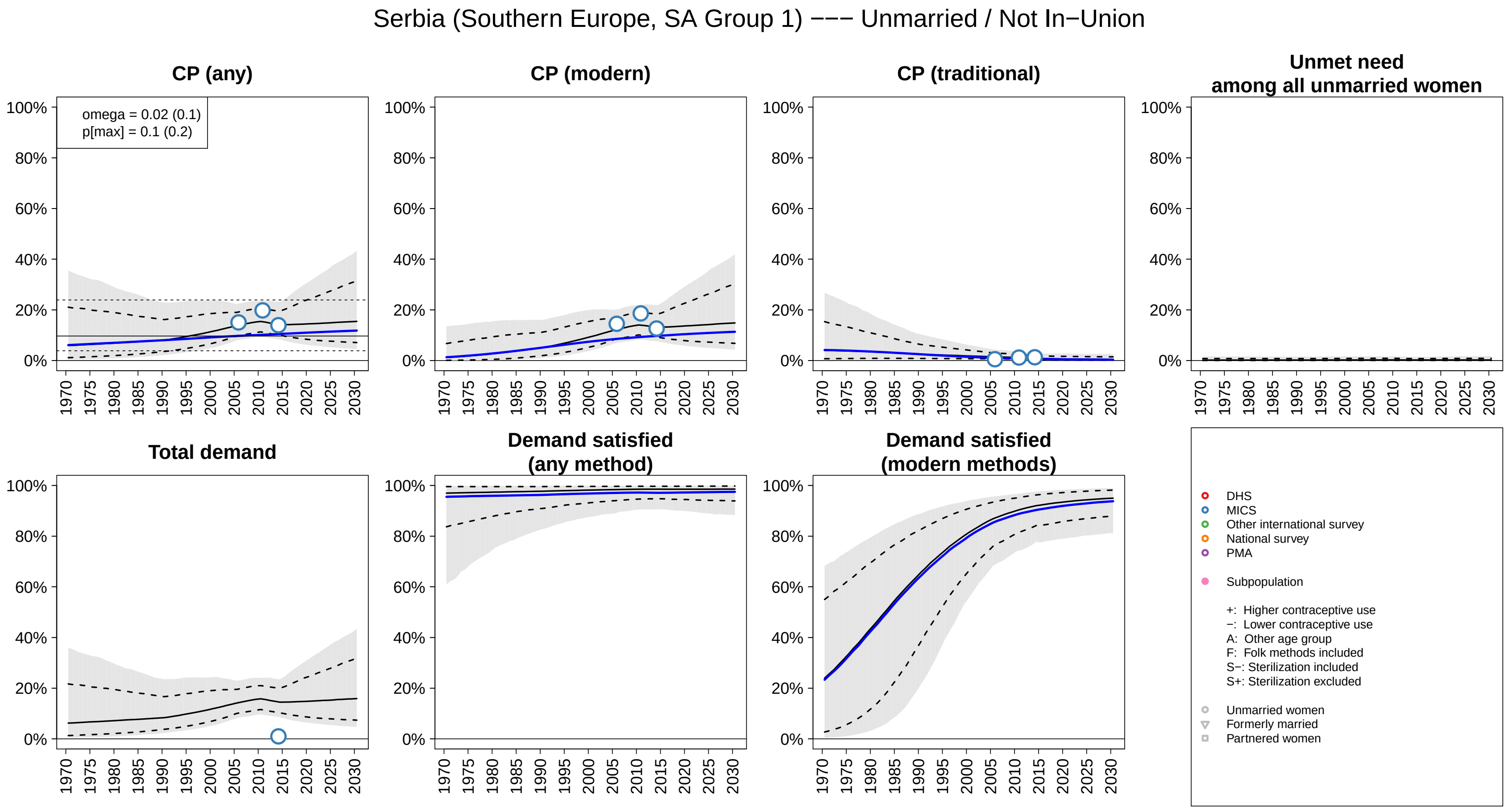
Serbia (Southern Europe, SA Group 1) −−− Unmarried / Not In−Union
CP (any)
omega = 0.02 (0.1)
p[max] = 0.1 (0.2)
CP (modern)
CP (traditional)
Unmet need among all unmarried women
Total demand
Demand satisfied (any method)
Demand satisfied (modern methods)
DHS
MICS
Other international survey
National survey
PMA
Subpopulation
+: Higher contraceptive use
−: Lower contraceptive use
A: Other age group
F: Folk methods included
S−: Sterilization included
S+: Sterilization excluded
Unmarried women
Formerly married
Partnered women

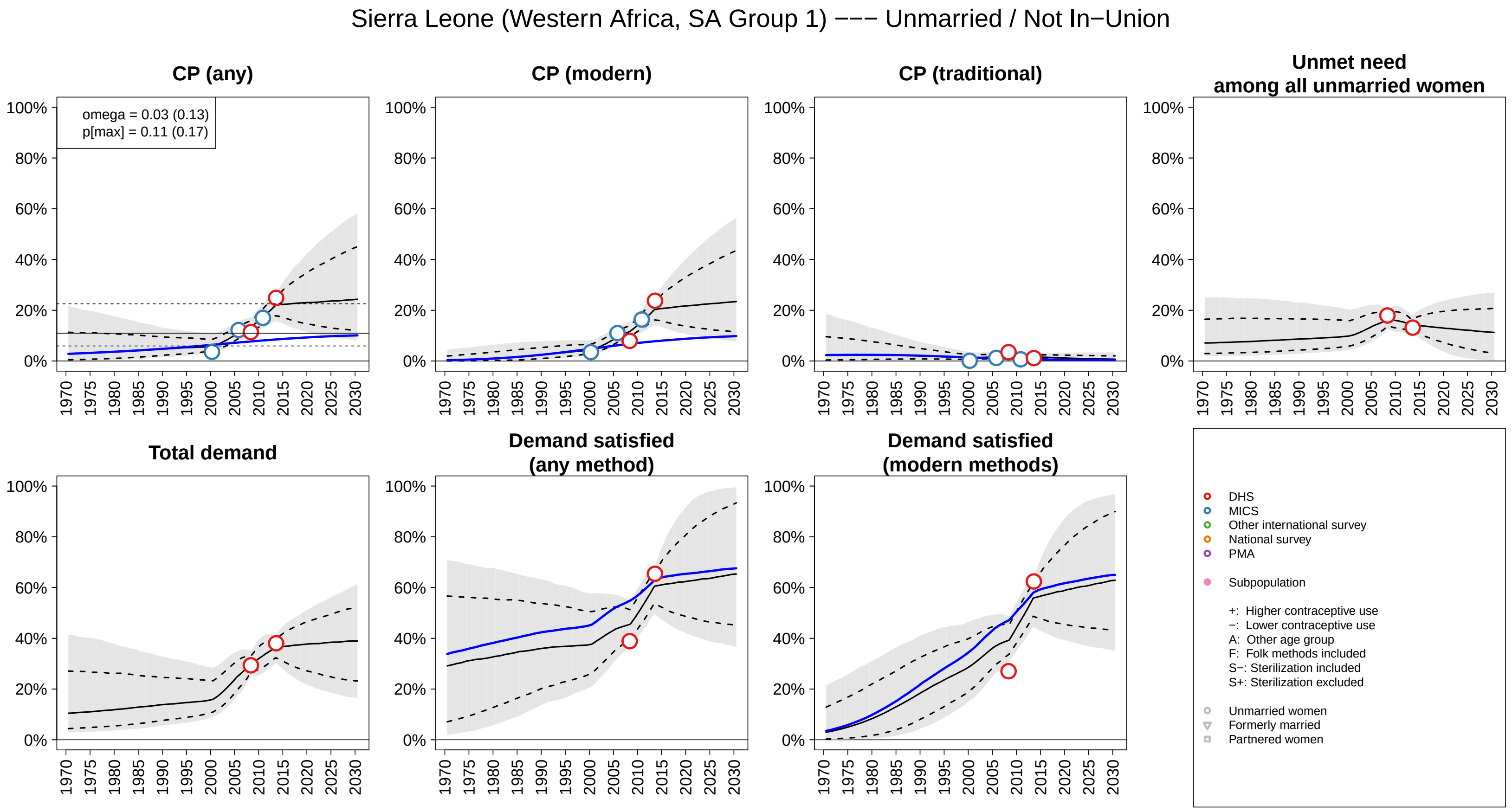
Sierra Leone (Western Africa, SA Group 1) −−− Unmarried / Not In−Union
CP (any)
omega = 0.03 (0.13)
p[max] = 0.11 (0.17)
CP (modern)
CP (traditional)
Unmet need among all unmarried women
Total demand
Demand satisfied (any method)
Demand satisfied (modern methods)
0%
20%
40%
60%
80%
100%
1970 1975 1980 1985 1990 1995 2000 2005 2010 2015 2020 2025 2030
DHS
MICS
Other international survey
National survey
PMA
Subpopulation
+: Higher contraceptive use
−: Lower contraceptive use
A: Other age group
F: Folk methods included
S−: Sterilization included
S+: Sterilization excluded
Unmarried women
Formerly married
Partnered women

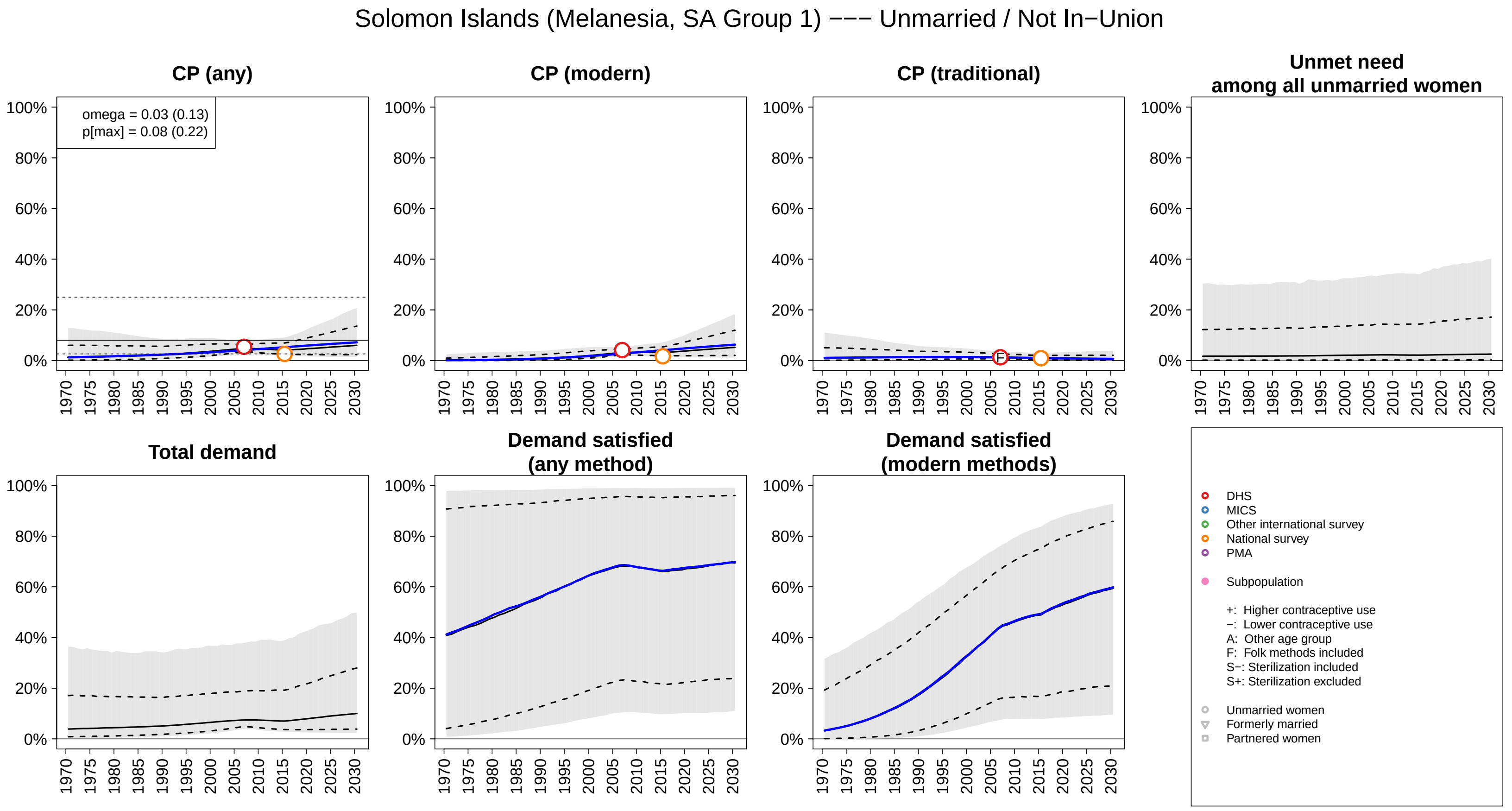
Solomon Islands (Melanesia, SA Group 1) −−− Unmarried / Not In−Union
CP (any)
omega = 0.03 (0.13)
p[max] = 0.08 (0.22)
CP (modern)
CP (traditional)
Unmet need among all unmarried women
Total demand
Demand satisfied (any method)
Demand satisfied (modern methods)
DHS
MICS
Other international survey
National survey
PMA
Subpopulation
+: Higher contraceptive use
−: Lower contraceptive use
A: Other age group
F: Folk methods included
S−: Sterilization included
S+: Sterilization excluded
Unmarried women
Formerly married
Partnered women

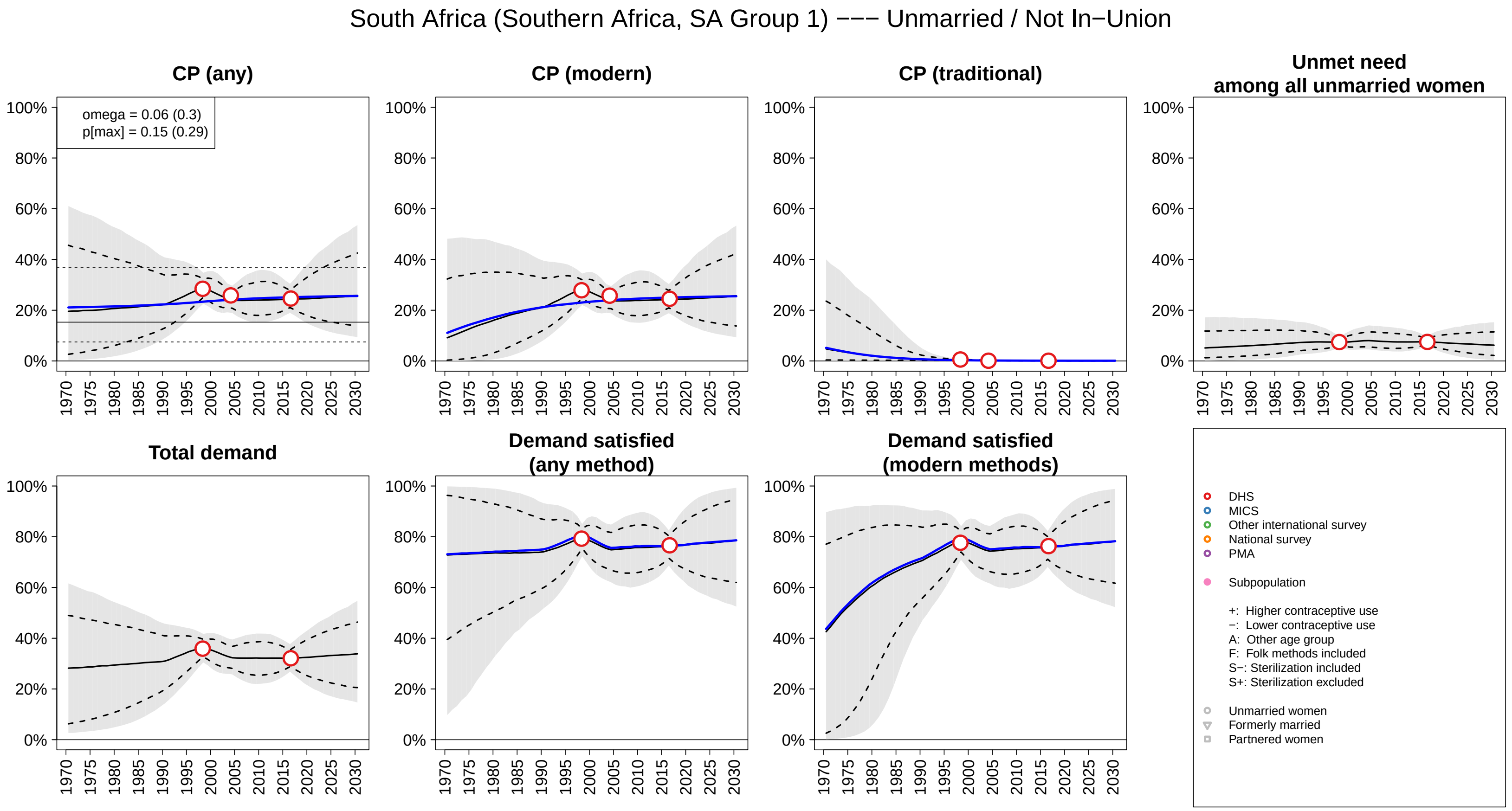
South Africa (Southern Africa, SA Group 1) −−− Unmarried / Not In−Union
CP (any)
omega = 0.06 (0.3)
p[max] = 0.15 (0.29)
CP (modern)
CP (traditional)
Unmet need among all unmarried women
Total demand
Demand satisfied (any method)
Demand satisfied (modern methods)
DHS
MICS
Other international survey
National survey
PMA
Subpopulation
+: Higher contraceptive use
−: Lower contraceptive use
A: Other age group
F: Folk methods included
S−: Sterilization included
S+: Sterilization excluded
Unmarried women
Formerly married
Partnered women

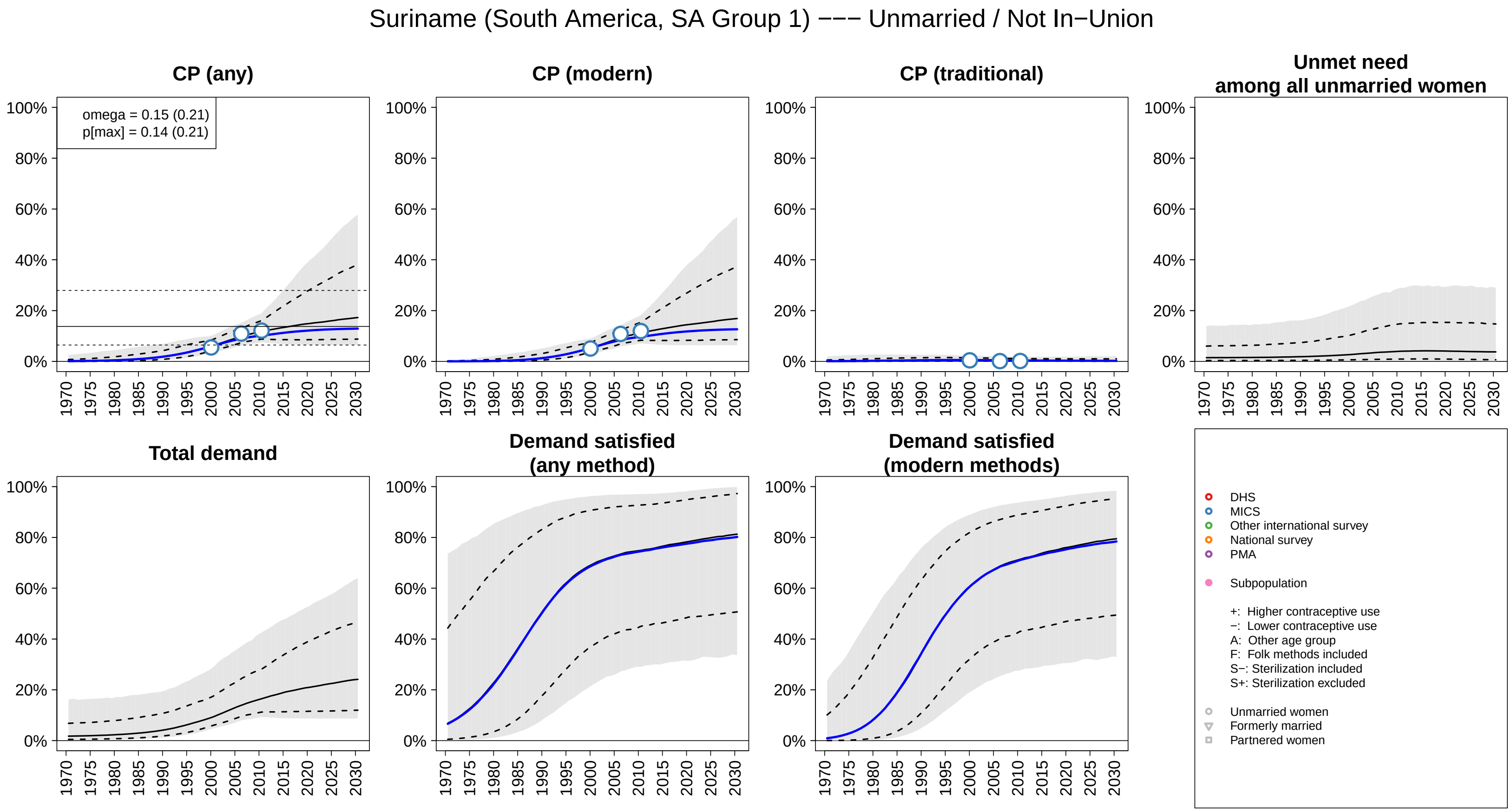
Suriname (South America, SA Group 1) ––– Unmarried / Not In−Union
CP (any)
omega = 0.15 (0.21)
p[max] = 0.14 (0.21)
CP (modern)
CP (traditional)
Unmet need among all unmarried women
Total demand
Demand satisfied (any method)
Demand satisfied (modern methods)
100%
80%
60%
40%
20%
0%
1970 1975 1980 1985 1990 1995 2000 2005 2010 2015 2020 2025 2030
DHS
MICS
Other international survey
National survey
PMA
Subpopulation
+: Higher contraceptive use
−: Lower contraceptive use
A: Other age group
F: Folk methods included
S−: Sterilization included
S+: Sterilization excluded
Unmarried women
Formerly married
Partnered women

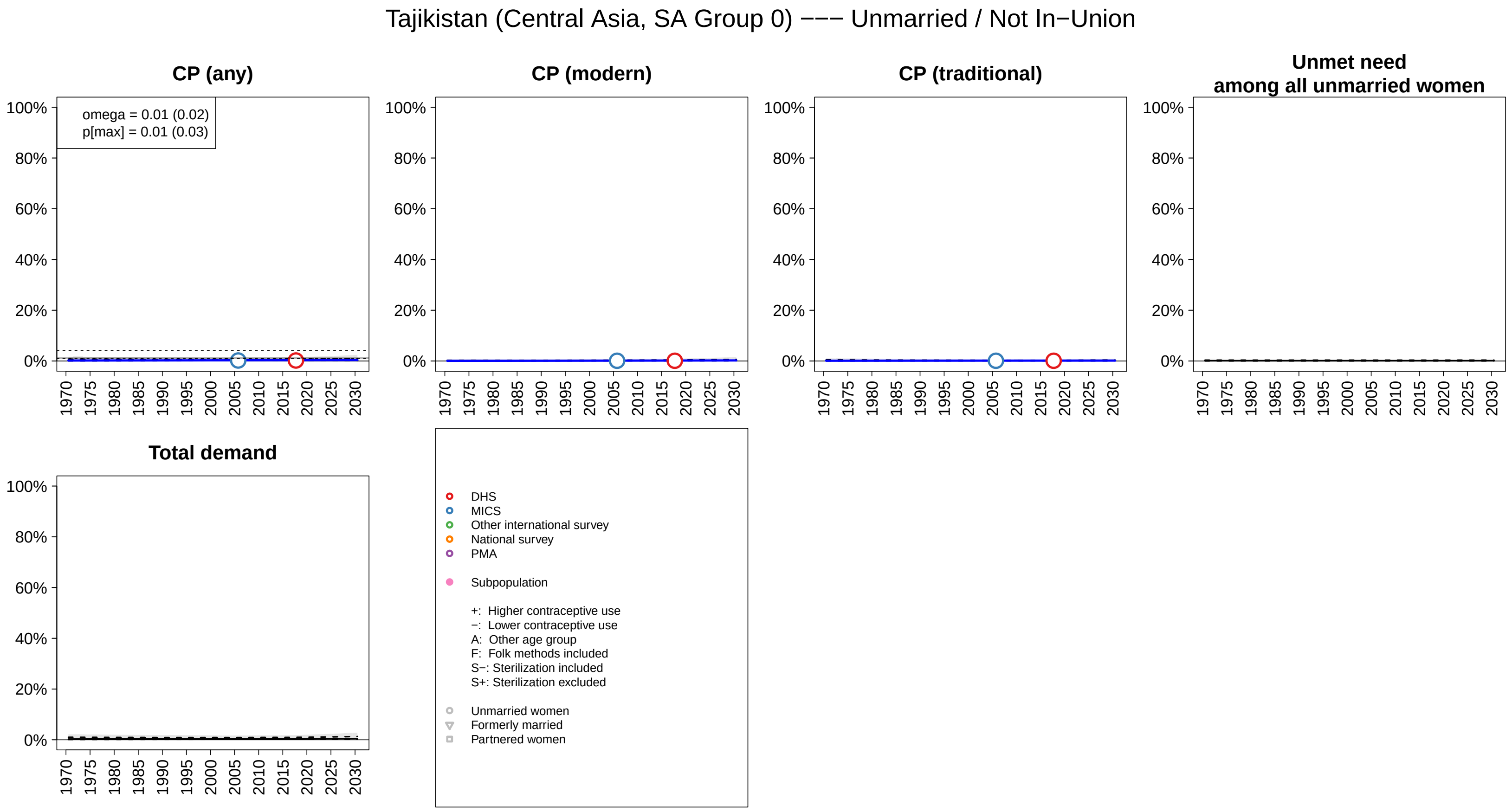
Tajikistan (Central Asia, SA Group 0) −−− Unmarried / Not In−Union
CP (any)
CP (modern)
CP (traditional)
Unmet need among all unmarried women
Total demand
omega = 0.01 (0.02)
p[max] = 0.01 (0.03)
DHS
MICS
Other international survey
National survey
PMA
Subpopulation
+: Higher contraceptive use
−: Lower contraceptive use
A: Other age group
F: Folk methods included
S−: Sterilization included
S+: Sterilization excluded
Unmarried women
Formerly married
Partnered women

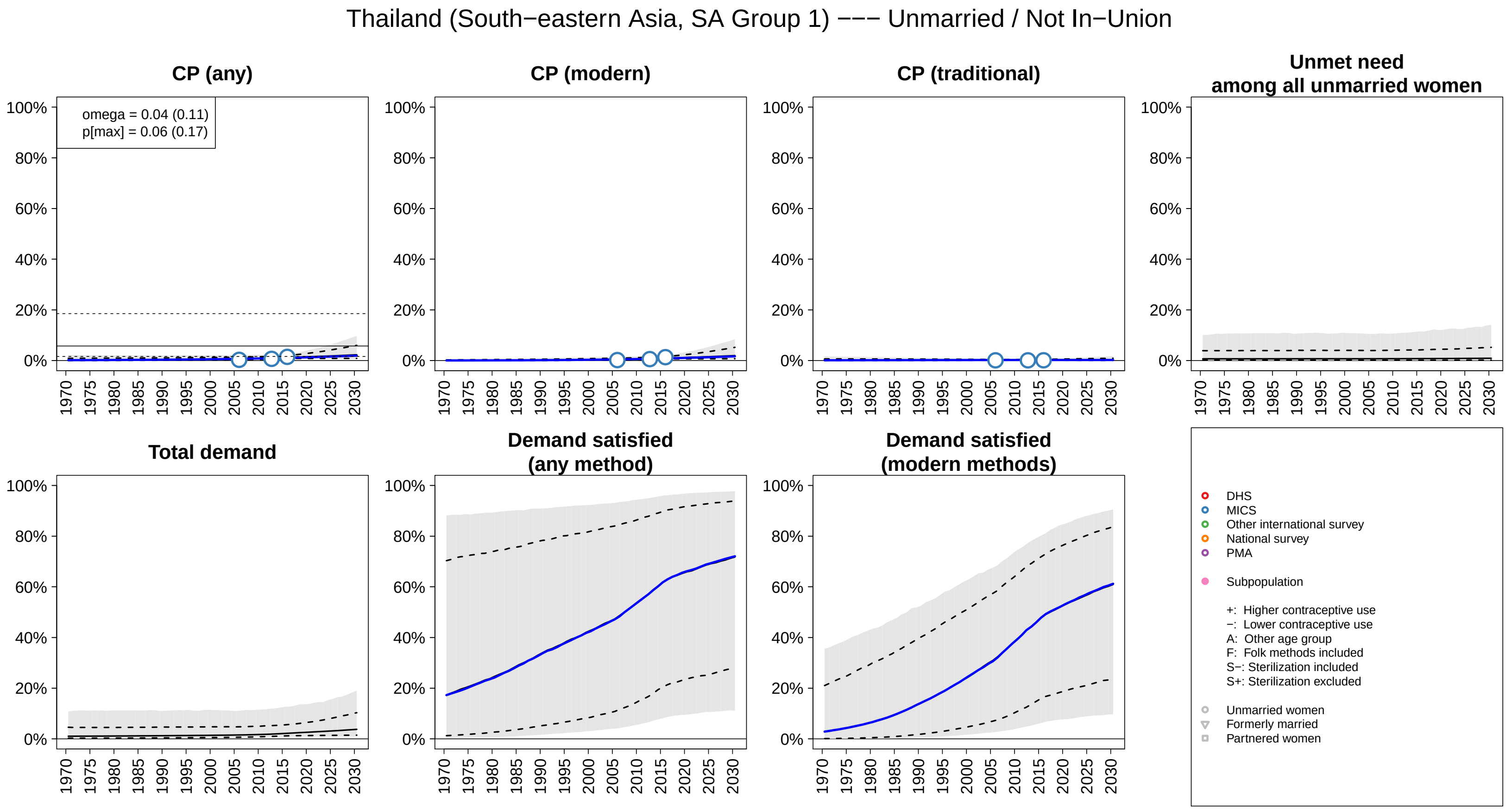

Thailand (South−eastern Asia, SA Group 1) −−− Unmarried / Not In−Union
CP (any)
omega = 0.04 (0.11)
p[max] = 0.06 (0.17)
CP (modern)
CP (traditional)
Unmet need among all unmarried women
Total demand
Demand satisfied (any method)
Demand satisfied (modern methods)
DHS
MICS
Other international survey
National survey
PMA
Subpopulation
+: Higher contraceptive use
−: Lower contraceptive use
A: Other age group
F: Folk methods included
S−: Sterilization included
S+: Sterilization excluded
Unmarried women
Formerly married
Partnered women

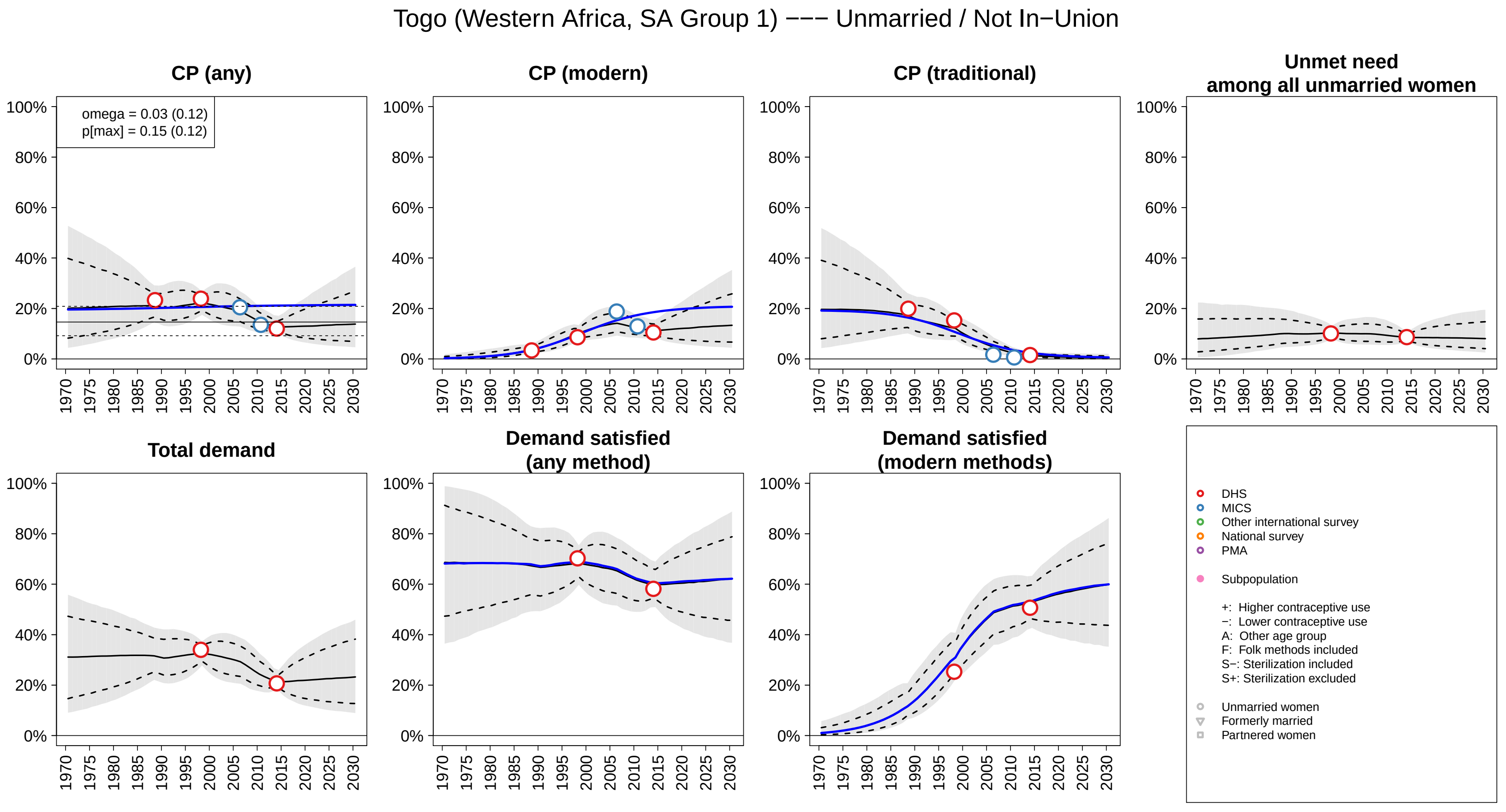
Togo (Western Africa, SA Group 1) ––– Unmarried / Not In–Union
CP (any)
omega = 0.03 (0.12)
p[max] = 0.15 (0.12)
CP (modern)
CP (traditional)
Unmet need among all unmarried women
Total demand
Demand satisfied (any method)
Demand satisfied (modern methods)
DHS
MICS
Other international survey
National survey
PMA
Subpopulation
+: Higher contraceptive use
−: Lower contraceptive use
A: Other age group
F: Folk methods included
S−: Sterilization included
S+: Sterilization excluded
Unmarried women
Formerly married
Partnered women

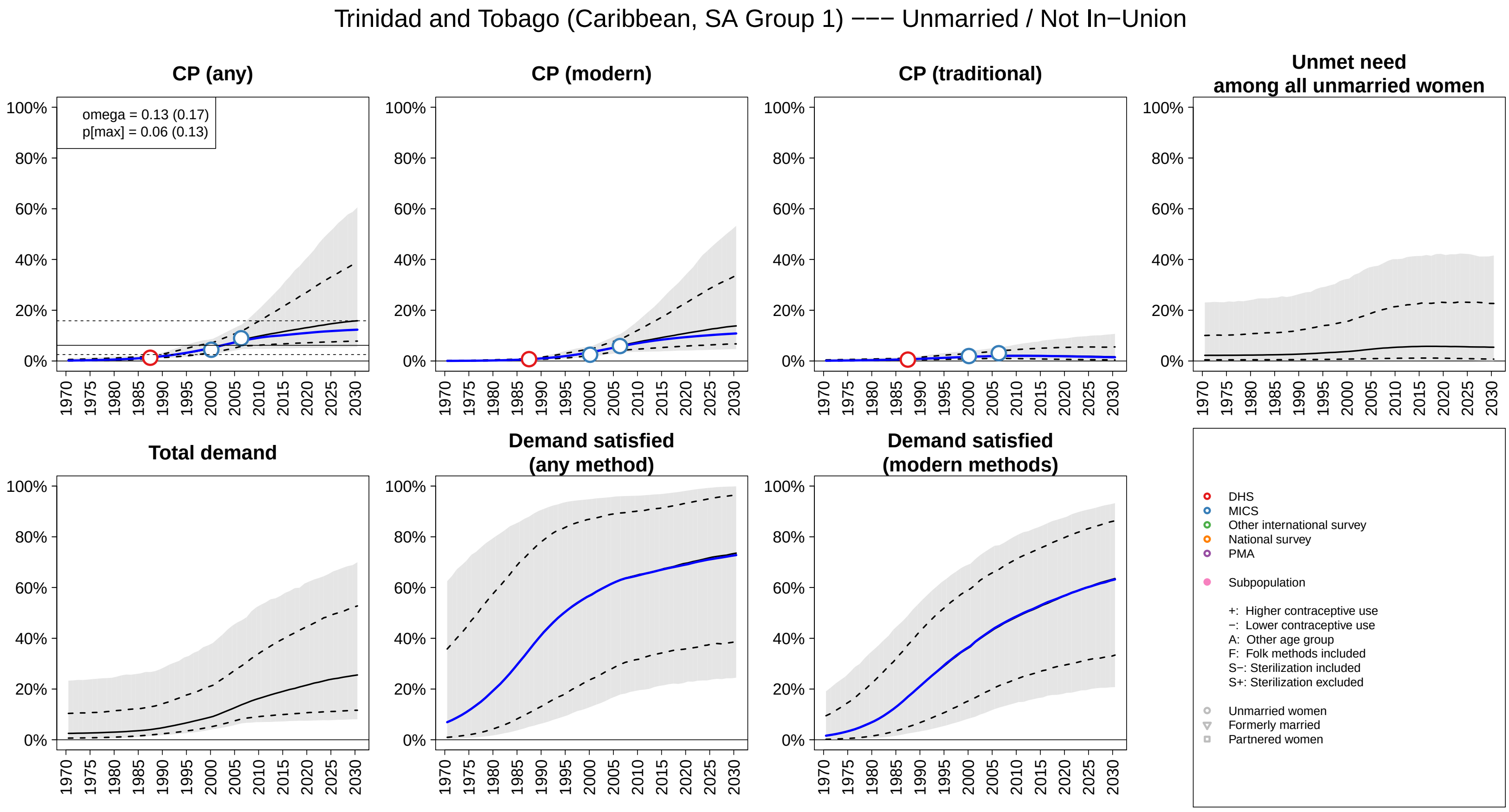
Trinidad and Tobago (Caribbean, SA Group 1) −−− Unmarried / Not In−Union
CP (any)
omega = 0.13 (0.17)
p[max] = 0.06 (0.13)
CP (modern)
CP (traditional)
Unmet need among all unmarried women
Total demand
Demand satisfied (any method)
Demand satisfied (modern methods)
DHS
MICS
Other international survey
National survey
PMA
Subpopulation
+: Higher contraceptive use
−: Lower contraceptive use
A: Other age group
F: Folk methods included
S−: Sterilization included
S+: Sterilization excluded
Unmarried women
Formerly married
Partnered women

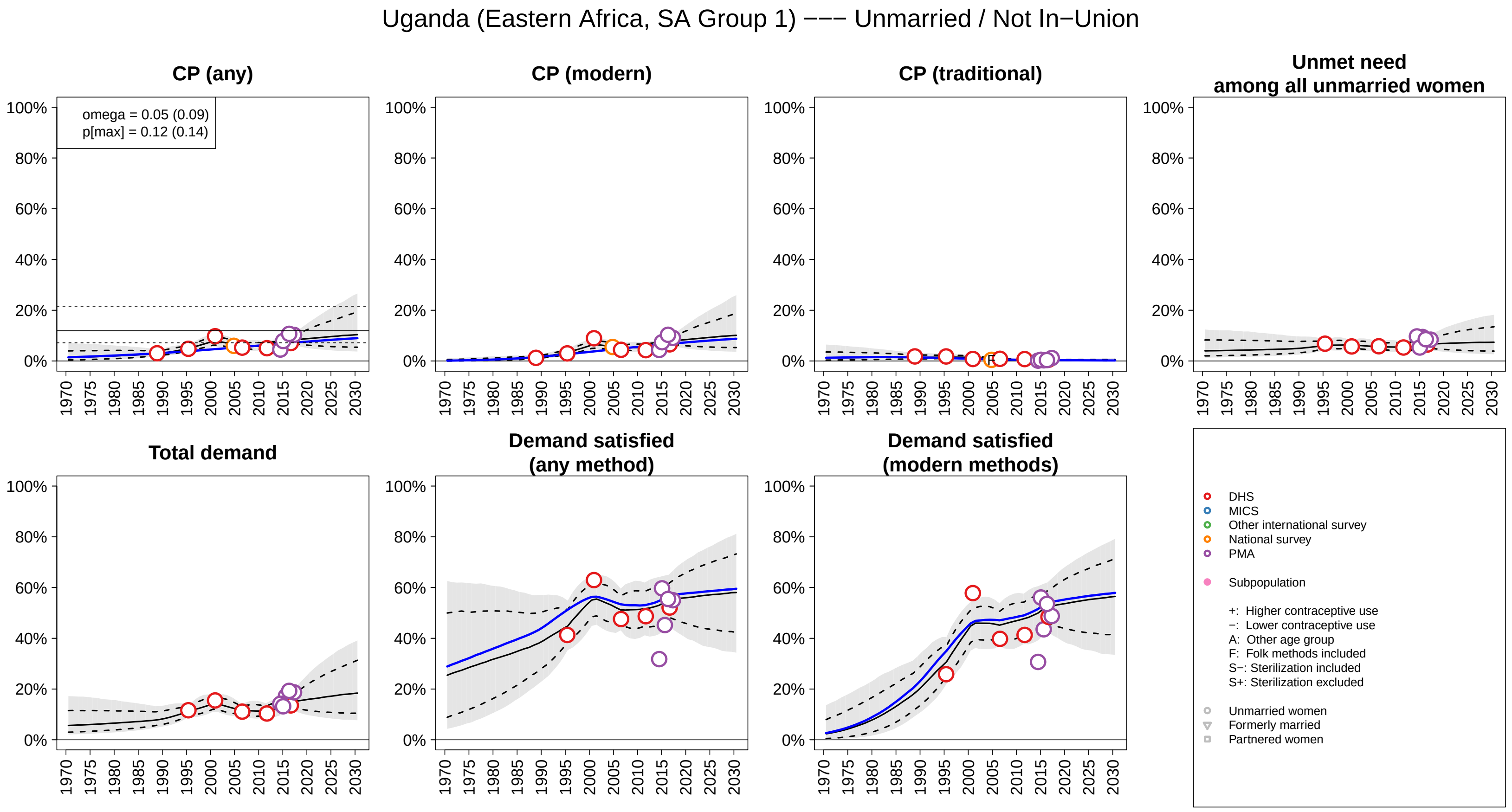
Uganda (Eastern Africa, SA Group 1) ––– Unmarried / Not In−Union
CP (any)
omega = 0.05 (0.09)
p[max] = 0.12 (0.14)
CP (modern)
CP (traditional)
Unmet need among all unmarried women
Total demand
Demand satisfied (any method)
Demand satisfied (modern methods)
DHS
MICS
Other international survey
National survey
PMA
Subpopulation
+: Higher contraceptive use
−: Lower contraceptive use
A: Other age group
F: Folk methods included
S−: Sterilization included
S+: Sterilization excluded
Unmarried women
Formerly married
Partnered women

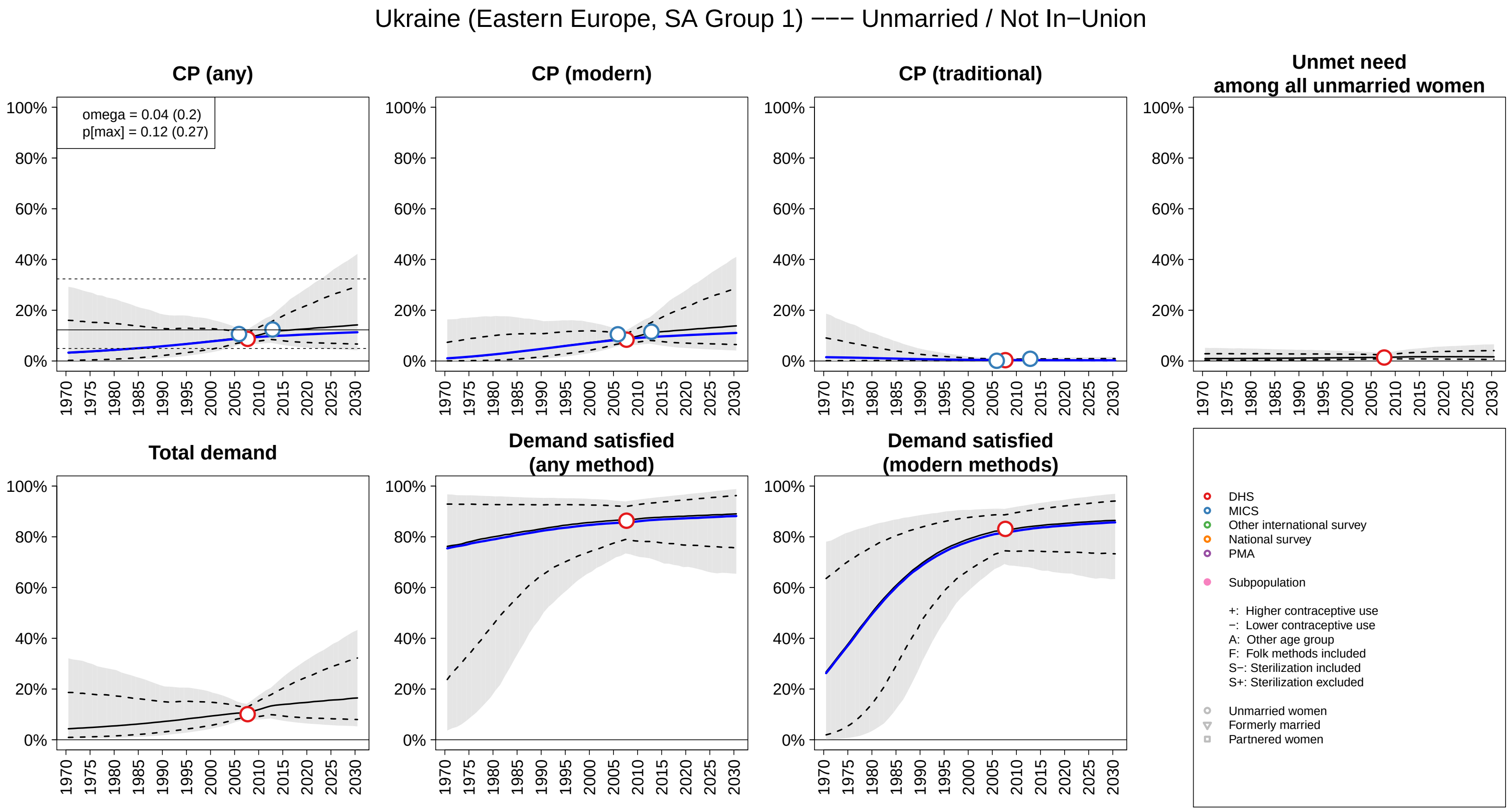
Ukraine (Eastern Europe, SA Group 1) --- Unmarried / Not In−Union
CP (any)
omega = 0.04 (0.2)
p[max] = 0.12 (0.27)
CP (modern)
CP (traditional)
Unmet need among all unmarried women
Total demand
Demand satisfied (any method)
Demand satisfied (modern methods)
DHS
MICS
Other international survey
National survey
PMA
Subpopulation
+: Higher contraceptive use
−: Lower contraceptive use
A: Other age group
F: Folk methods included
S−: Sterilization included
S+: Sterilization excluded
Unmarried women
Formerly married
Partnered women

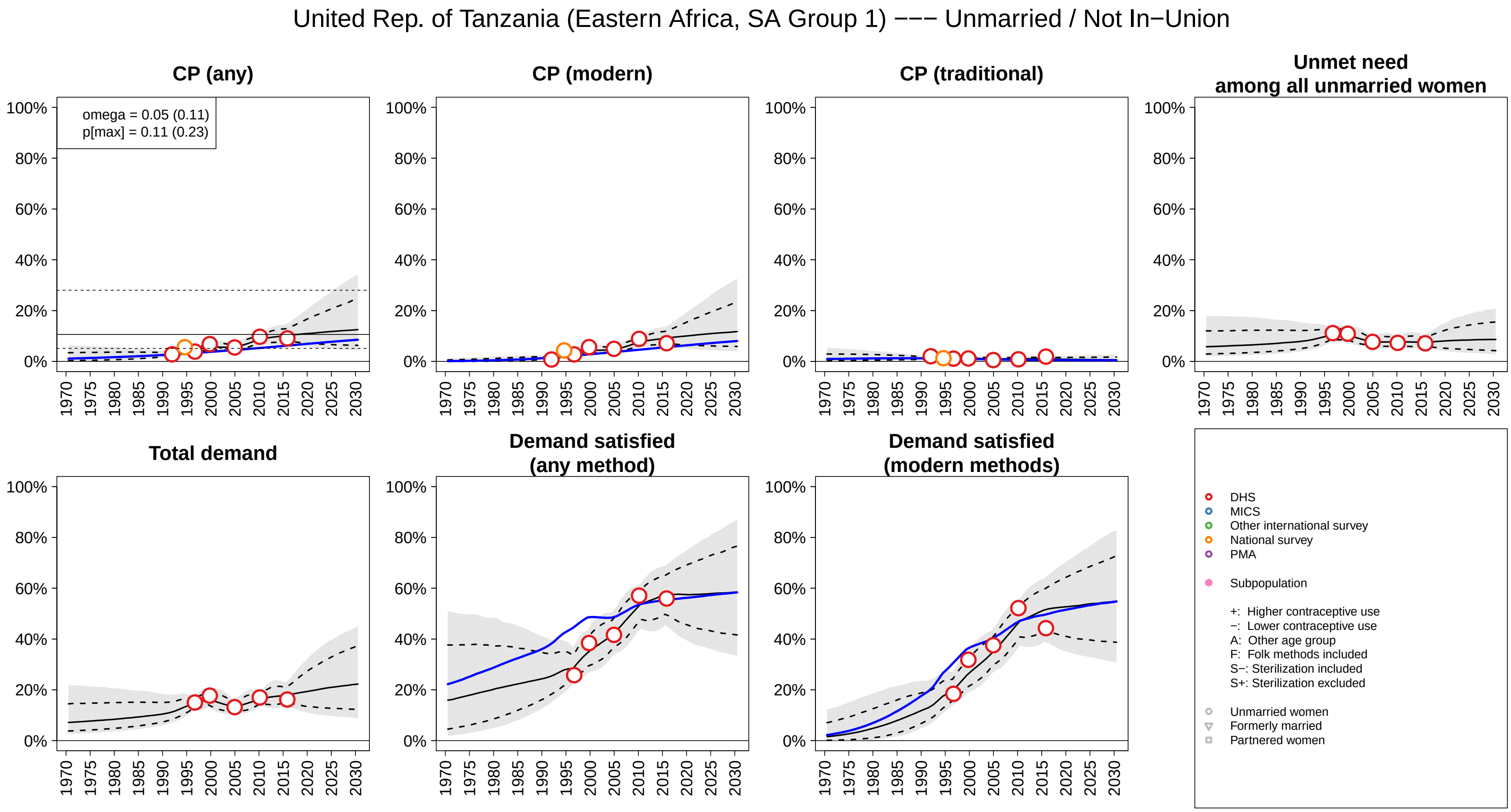
United Rep. of Tanzania (Eastern Africa, SA Group 1) ––– Unmarried / Not In−Union
CP (any)
omega = 0.05 (0.11)
p[max] = 0.11 (0.23)
CP (modern)
CP (traditional)
Unmet need among all unmarried women
Total demand
Demand satisfied (any method)
Demand satisfied (modern methods)
DHS
MICS
Other international survey
National survey
PMA
Subpopulation
+: Higher contraceptive use
−: Lower contraceptive use
A: Other age group
F: Folk methods included
S−: Sterilization included
S+: Sterilization excluded
Unmarried women
Formerly married
Partnered women

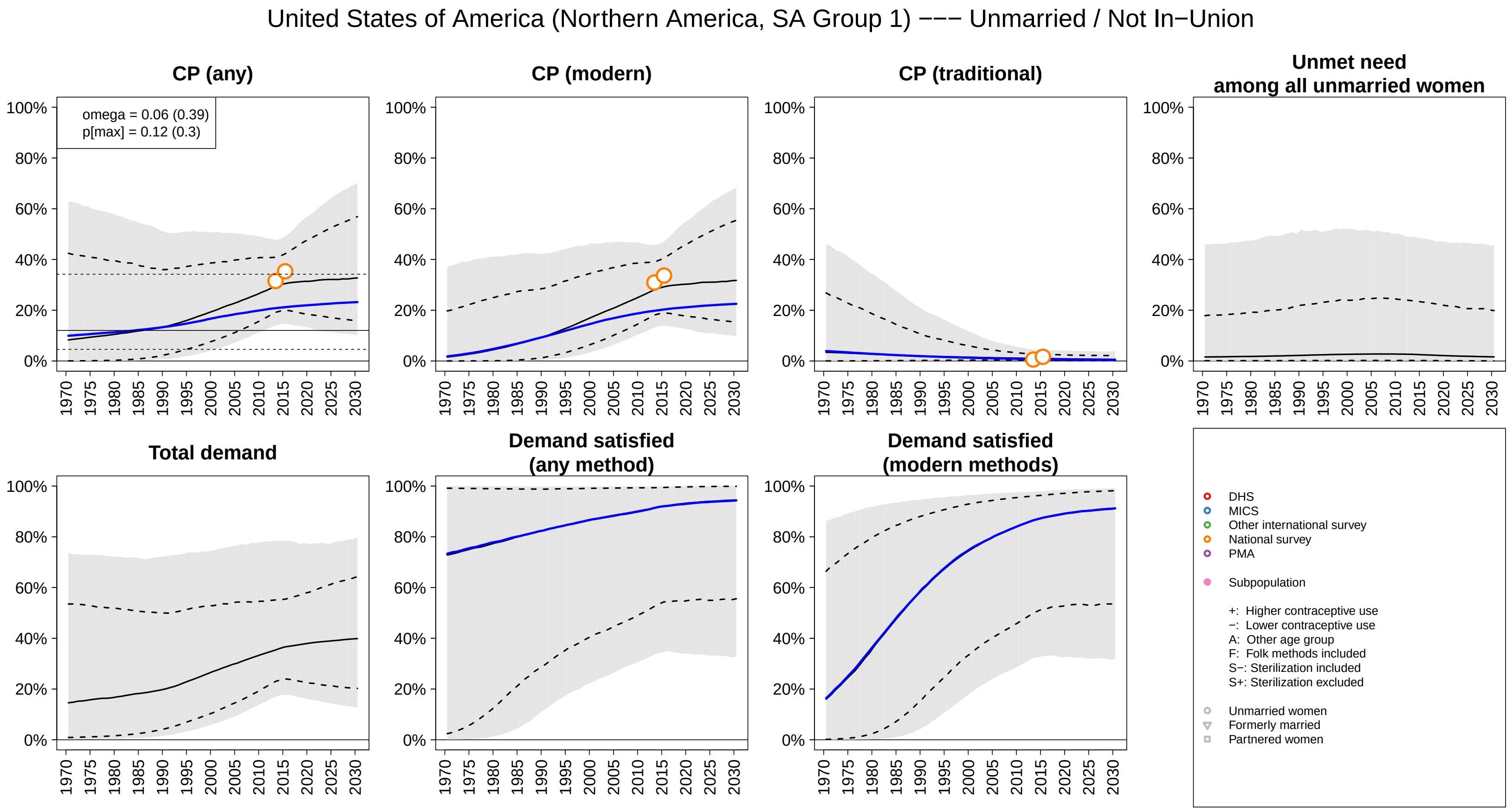
United States of America (Northern America, SA Group 1) −−− Unmarried / Not In−Union
CP (any)
omega = 0.06 (0.39)
p[max] = 0.12 (0.3)
CP (modern)
CP (traditional)
Unmet need among all unmarried women
Total demand
Demand satisfied (any method)
Demand satisfied (modern methods)
DHS
MICS
Other international survey
National survey
PMA
Subpopulation
+: Higher contraceptive use
−: Lower contraceptive use
A: Other age group
F: Folk methods included
S−: Sterilization included
S+: Sterilization excluded
Unmarried women
Formerly married
Partnered women

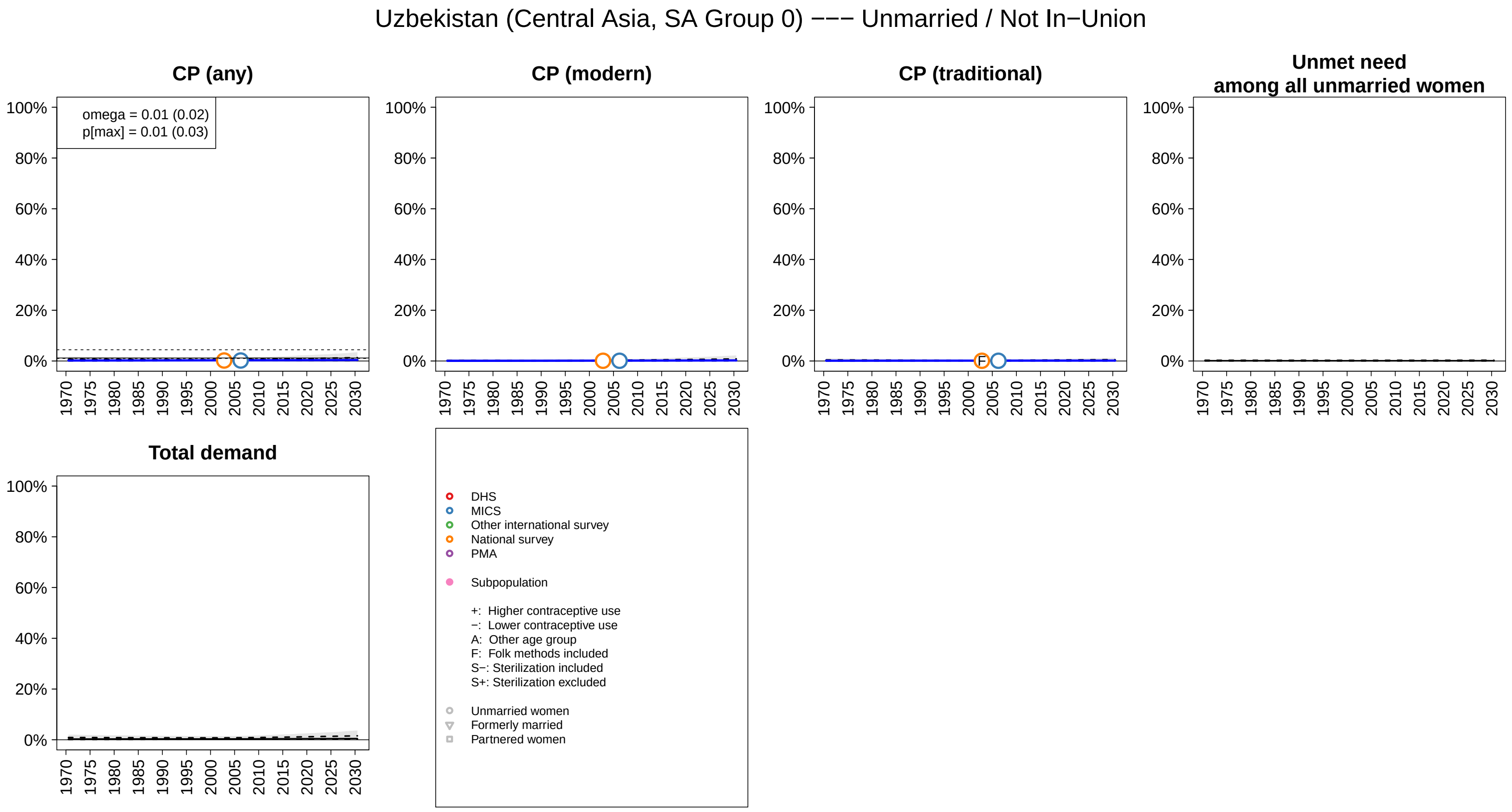
Uzbekistan (Central Asia, SA Group 0) −−− Unmarried / Not In−Union
CP (any)
omega = 0.01 (0.02)
p[max] = 0.01 (0.03)
CP (modern)
CP (traditional)
Unmet need among all unmarried women
Total demand
DHS
MICS
Other international survey
National survey
PMA
Subpopulation
+: Higher contraceptive use
−: Lower contraceptive use
A: Other age group
F: Folk methods included
S−: Sterilization included
S+: Sterilization excluded
Unmarried women
Formerly married
Partnered women

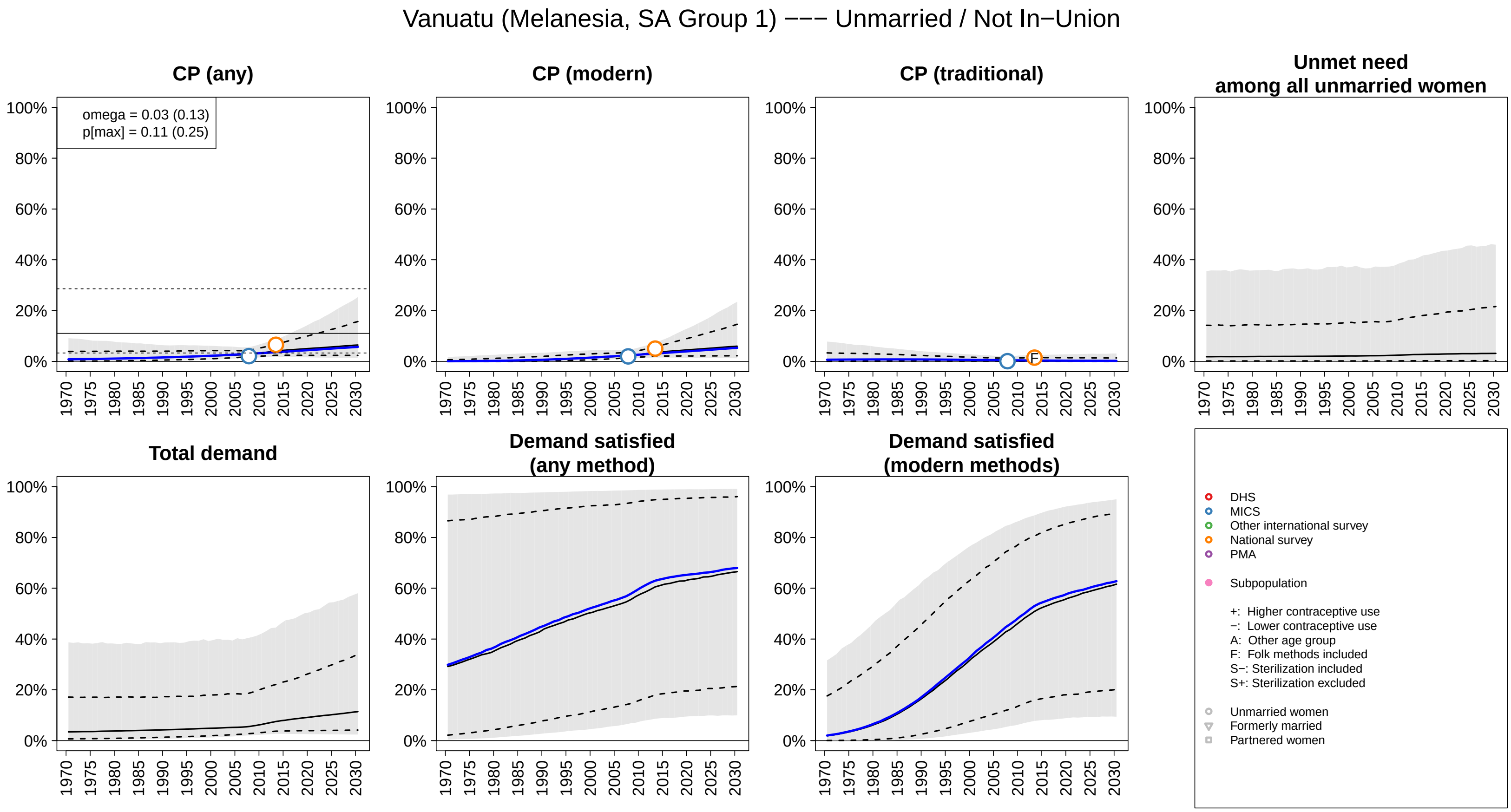
Vanuatu (Melanesia, SA Group 1) −−− Unmarried / Not In−Union
CP (any)
omega = 0.03 (0.13)
p[max] = 0.11 (0.25)
CP (modern)
CP (traditional)
Unmet need among all unmarried women
Total demand
Demand satisfied (any method)
Demand satisfied (modern methods)
DHS
MICS
Other international survey
National survey
PMA
Subpopulation
+: Higher contraceptive use
−: Lower contraceptive use
A: Other age group
F: Folk methods included
S−: Sterilization included
S+: Sterilization excluded
Unmarried women
Formerly married
Partnered women

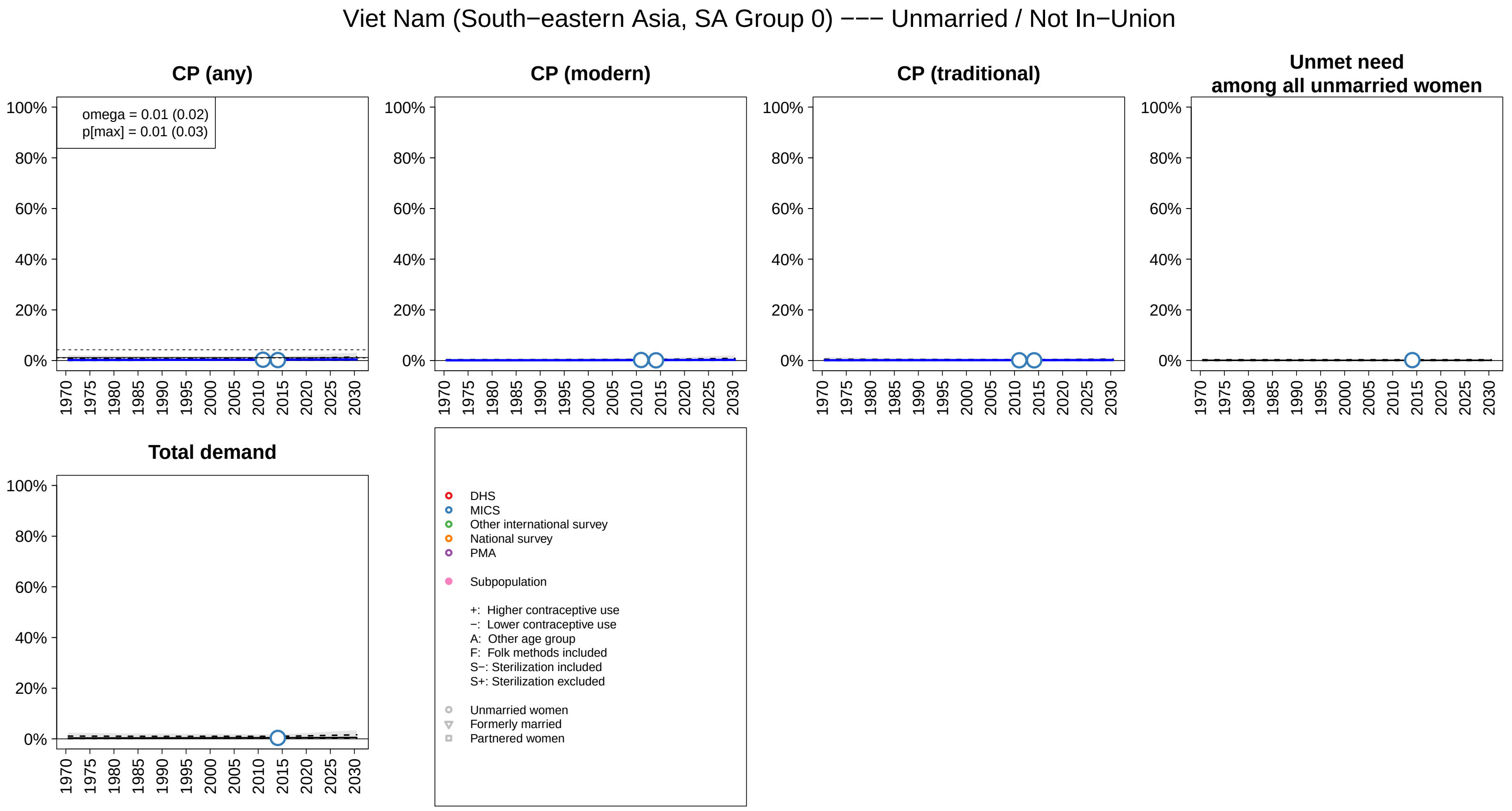

Viet Nam (South−eastern Asia, SA Group 0) −−− Unmarried / Not In−Union
CP (any)
CP (modern)
CP (traditional)
Unmet need
among all unmarried women
Total demand
omega = 0.01 (0.02)
p[max] = 0.01 (0.03)
100%
80%
60%
40%
20%
0%
1970
1975
1980
1985
1990
1995
2000
2005
2010
2015
2020
2025
2030
DHS
MICS
Other international survey
National survey
PMA
Subpopulation
+: Higher contraceptive use
−: Lower contraceptive use
A: Other age group
F: Folk methods included
S−: Sterilization included
S+: Sterilization excluded
Unmarried women
Formerly married
Partnered women

Zambia (Eastern Africa, SA Group 1) --- Unmarried / Not In−Union

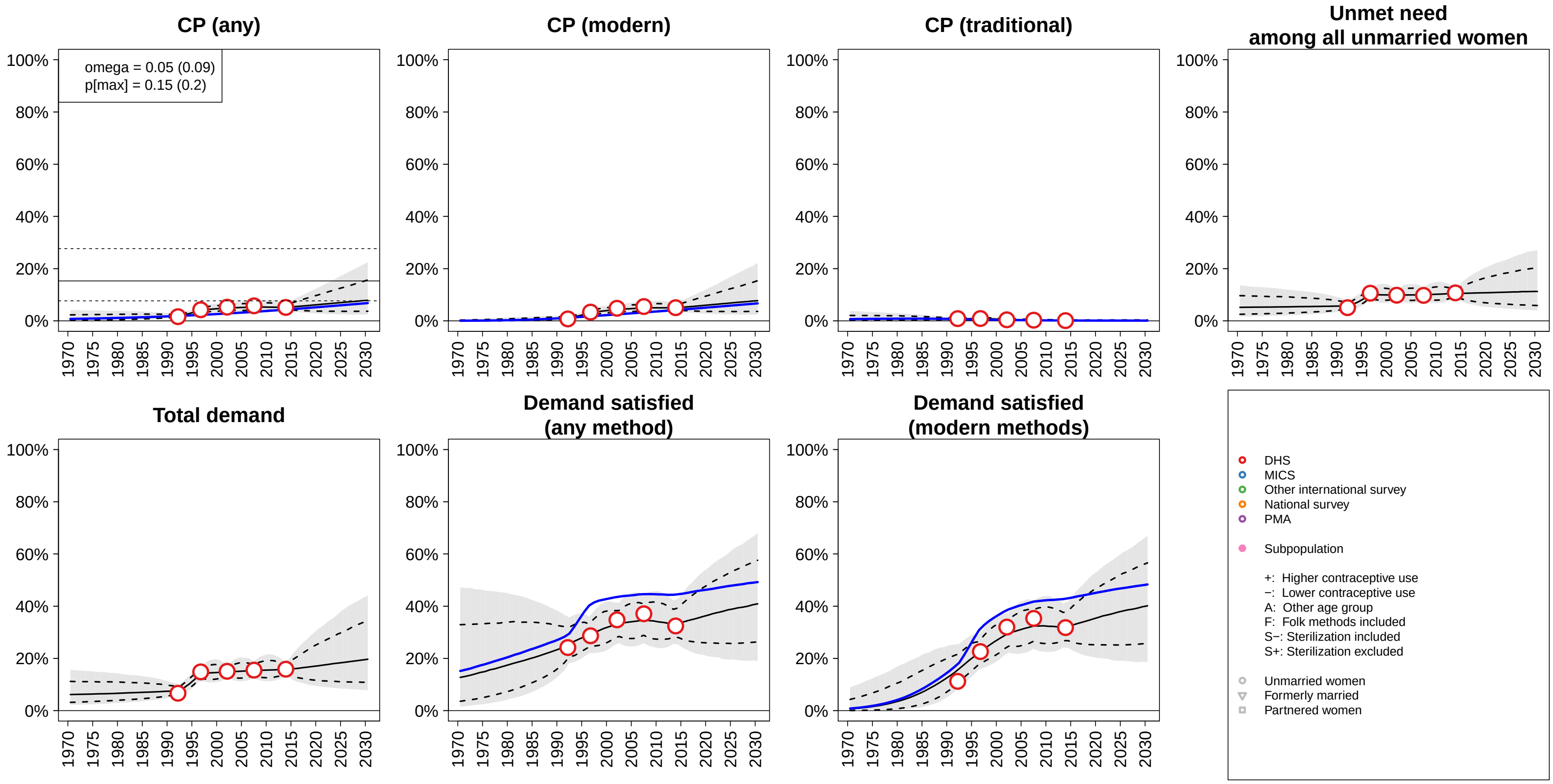

Zimbabwe (Eastern Africa, SA Group 1) −−− Unmarried / Not In−Union

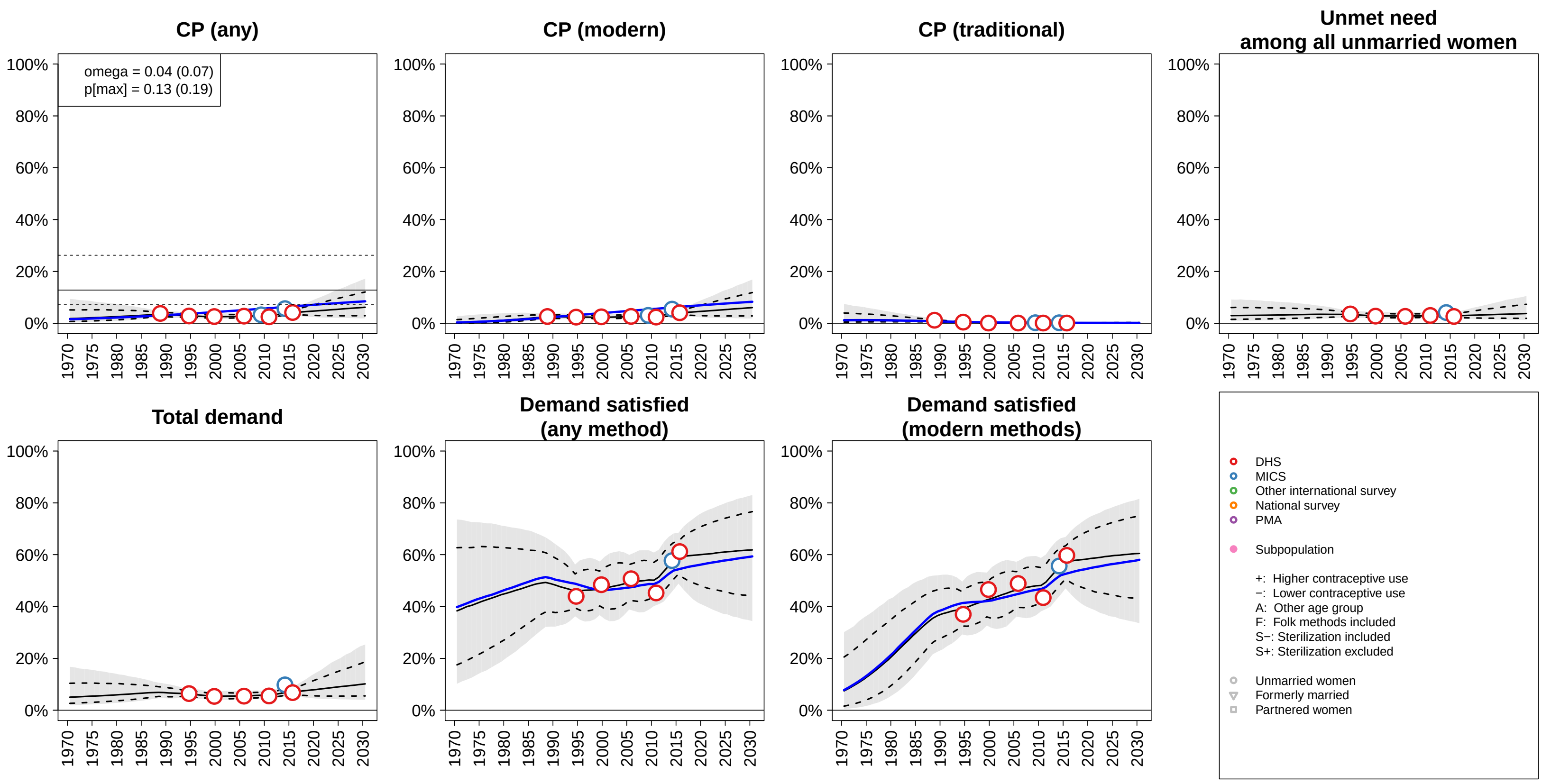

4.2 Married Adolescent Women

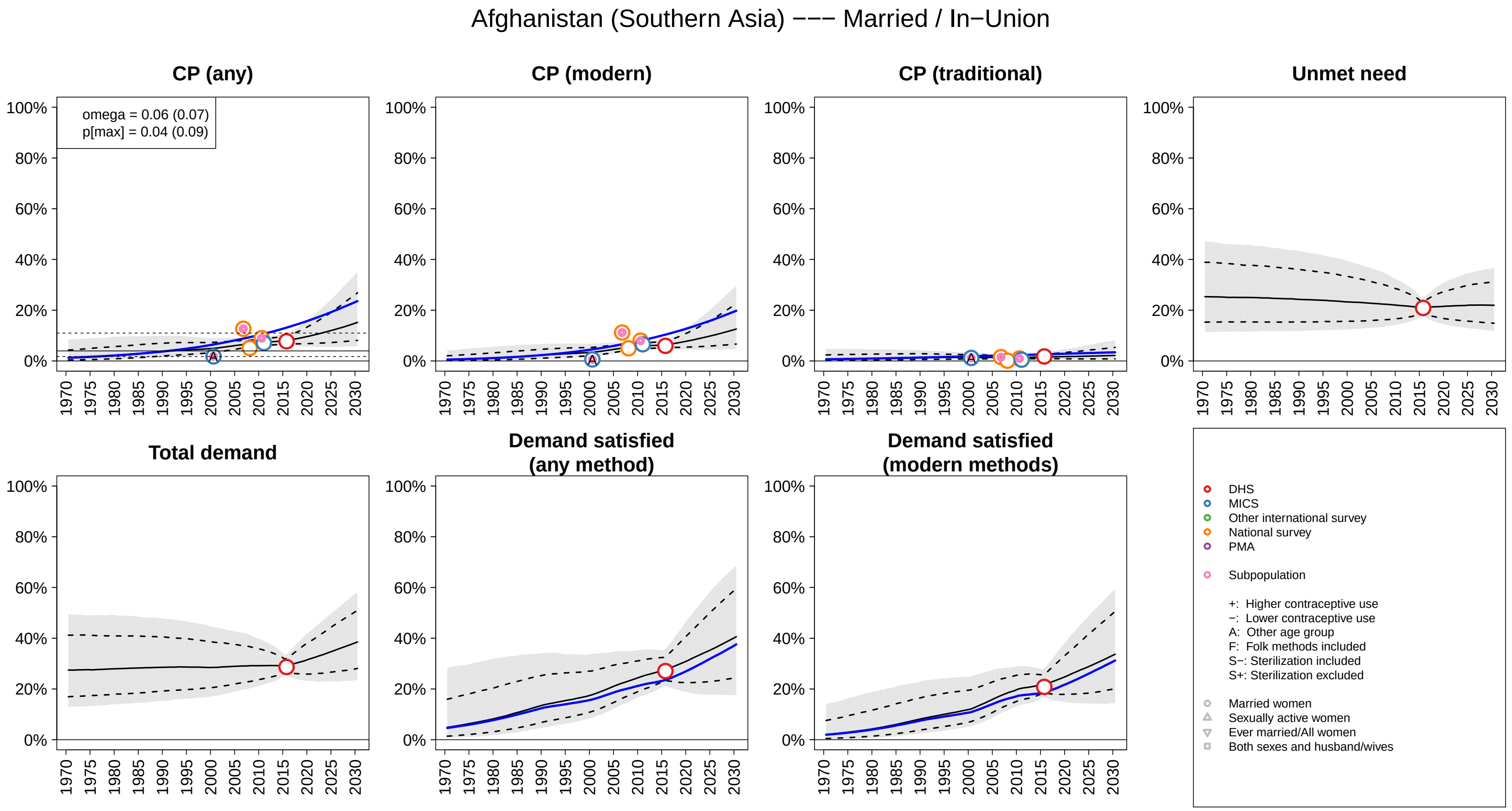

Afghanistan (Southern Asia) −−− Married / In−Union
CP (any)
omega = 0.06 (0.07)
p[max] = 0.04 (0.09)
CP (modern)
CP (traditional)
Unmet need
Total demand
Demand satisfied (any method)
Demand satisfied (modern methods)
DHS
MICS
Other international survey
National survey
PMA
Subpopulation
+: Higher contraceptive use
−: Lower contraceptive use
A: Other age group
F: Folk methods included
S−: Sterilization included
S+: Sterilization excluded
Married women
Sexually active women
Ever married/All women
Both sexes and husband/wives

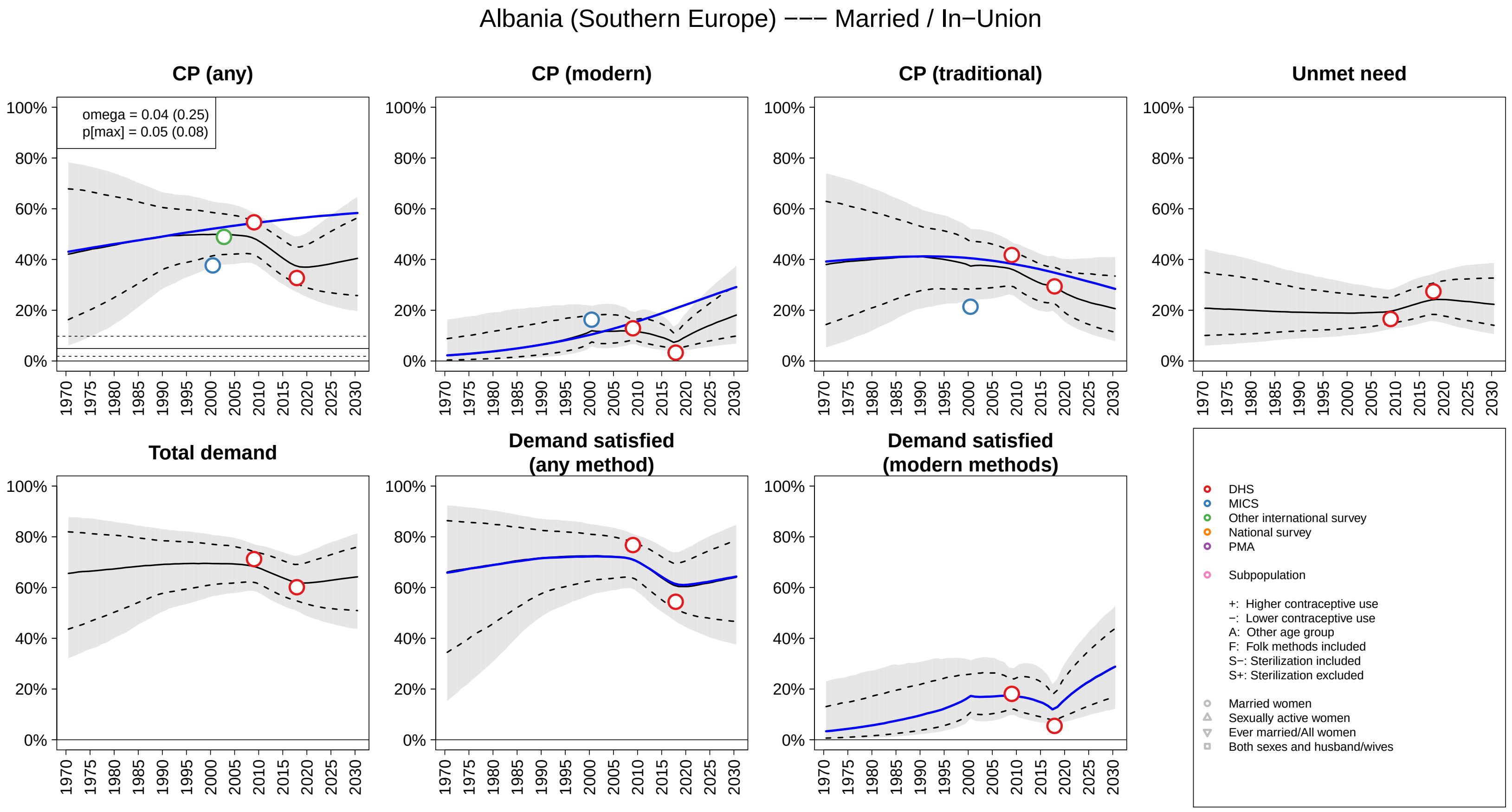
Albania (Southern Europe) --- Married / In−Union
CP (any)
omega = 0.04 (0.25)
p[max] = 0.05 (0.08)
CP (modern)
CP (traditional)
Unmet need
Total demand
Demand satisfied (any method)
Demand satisfied (modern methods)
DHS
MICS
Other international survey
National survey
PMA
Subpopulation
+: Higher contraceptive use
−: Lower contraceptive use
A: Other age group
F: Folk methods included
S−: Sterilization included
S+: Sterilization excluded
Married women
Sexually active women
Ever married/All women
Both sexes and husband/wives

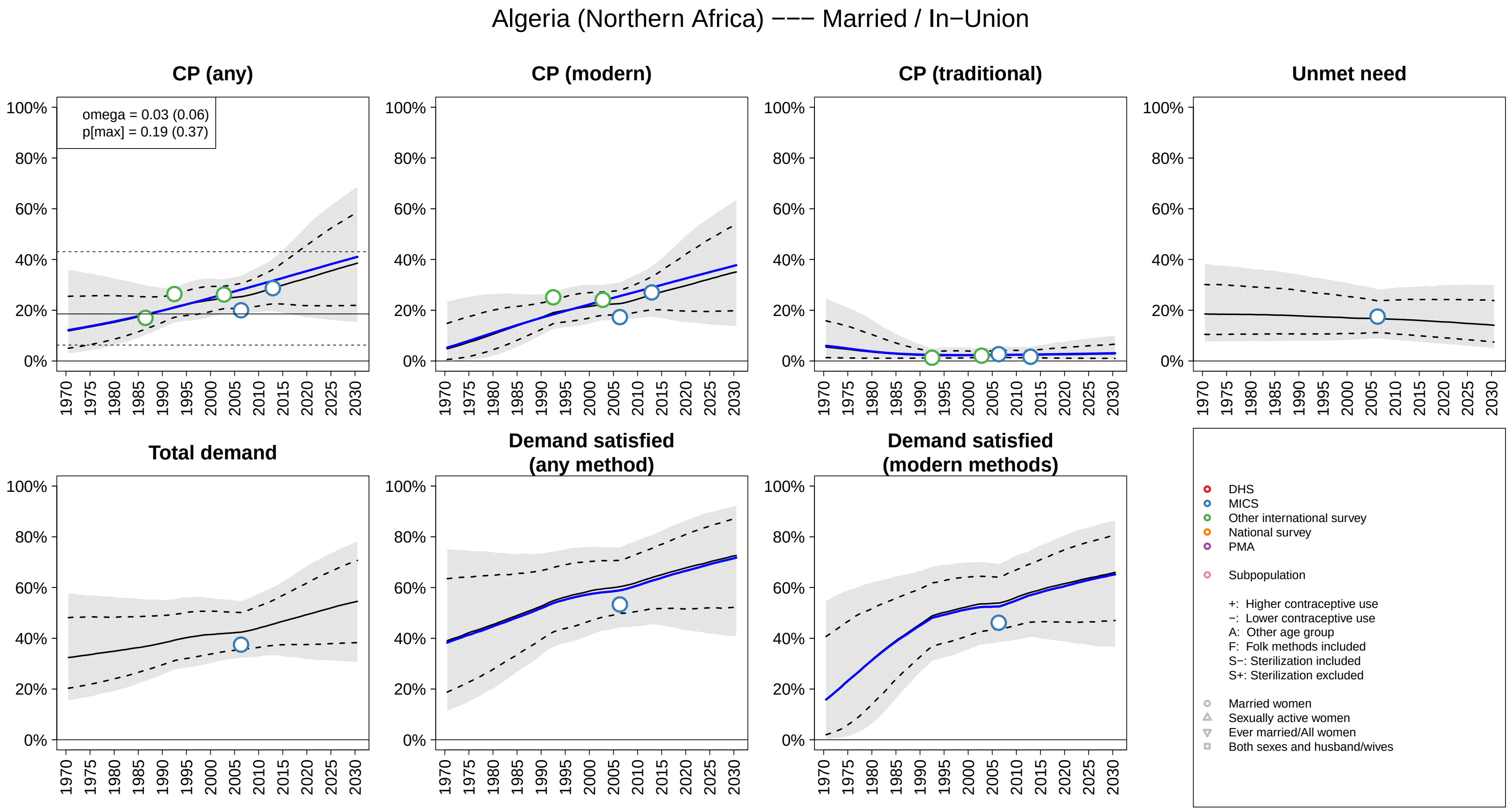
Algeria (Northern Africa) −−− Married / In−Union
CP (any)
omega = 0.03 (0.06)
p[max] = 0.19 (0.37)
CP (modern)
CP (traditional)
Unmet need
Total demand
Demand satisfied (any method)
Demand satisfied (modern methods)
DHS
MICS
Other international survey
National survey
PMA
Subpopulation
+: Higher contraceptive use
−: Lower contraceptive use
A: Other age group
F: Folk methods included
S−: Sterilization included
S+: Sterilization excluded
Married women
Sexually active women
Ever married/All women
Both sexes and husband/wives

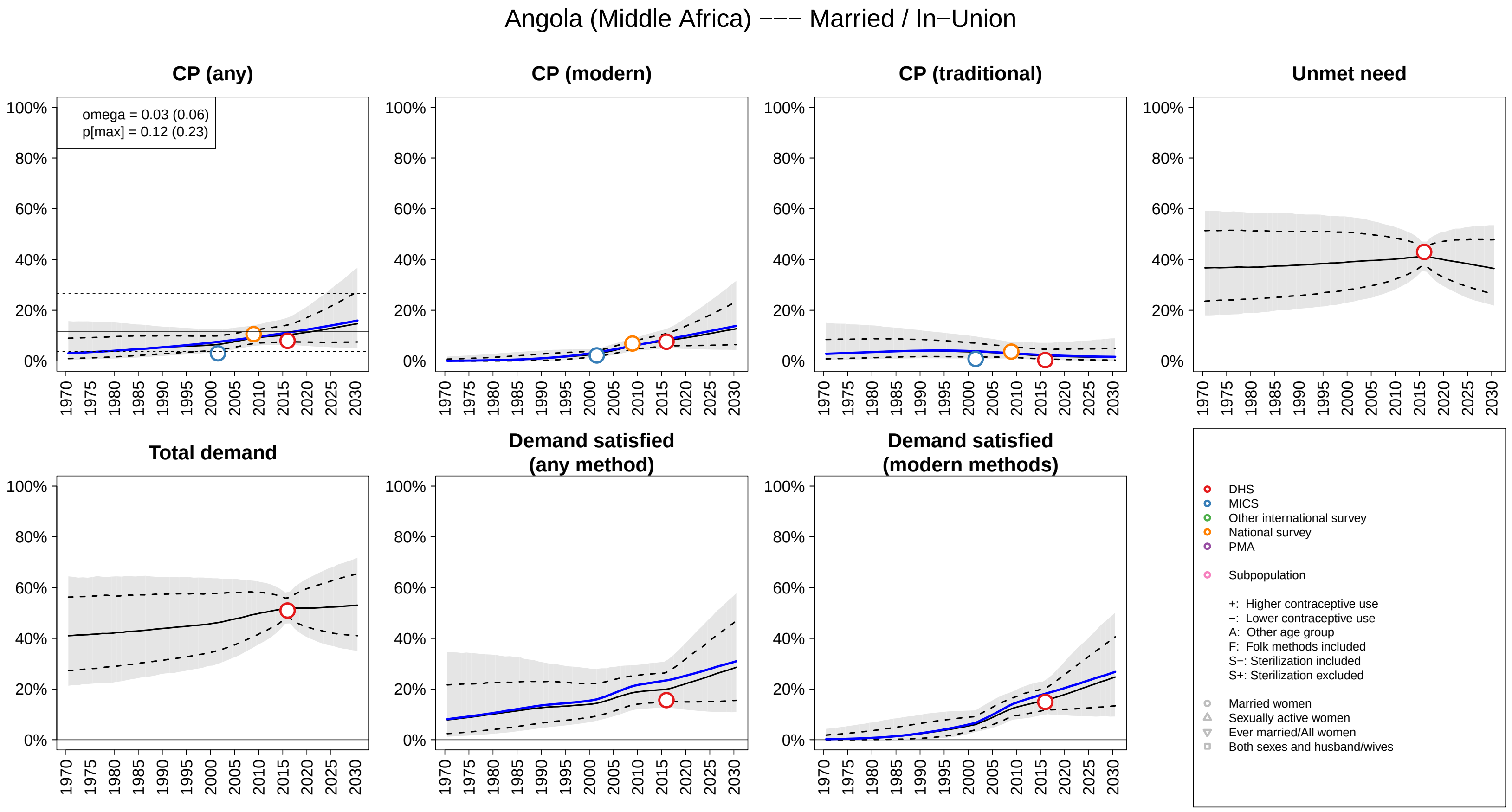
Angola (Middle Africa) −−− Married / In−Union
CP (any)
omega = 0.03 (0.06)
p[max] = 0.12 (0.23)
CP (modern)
CP (traditional)
Unmet need
Total demand
Demand satisfied (any method)
Demand satisfied (modern methods)
0%
20%
40%
60%
80%
100%
1970
1975
1980
1985
1990
1995
2000
2005
2010
2015
2020
2025
2030
DHS
MICS
Other international survey
National survey
PMA
Subpopulation
+: Higher contraceptive use
−: Lower contraceptive use
A: Other age group
F: Folk methods included
S−: Sterilization included
S+: Sterilization excluded
Married women
Sexually active women
Ever married/All women
Both sexes and husband/wives

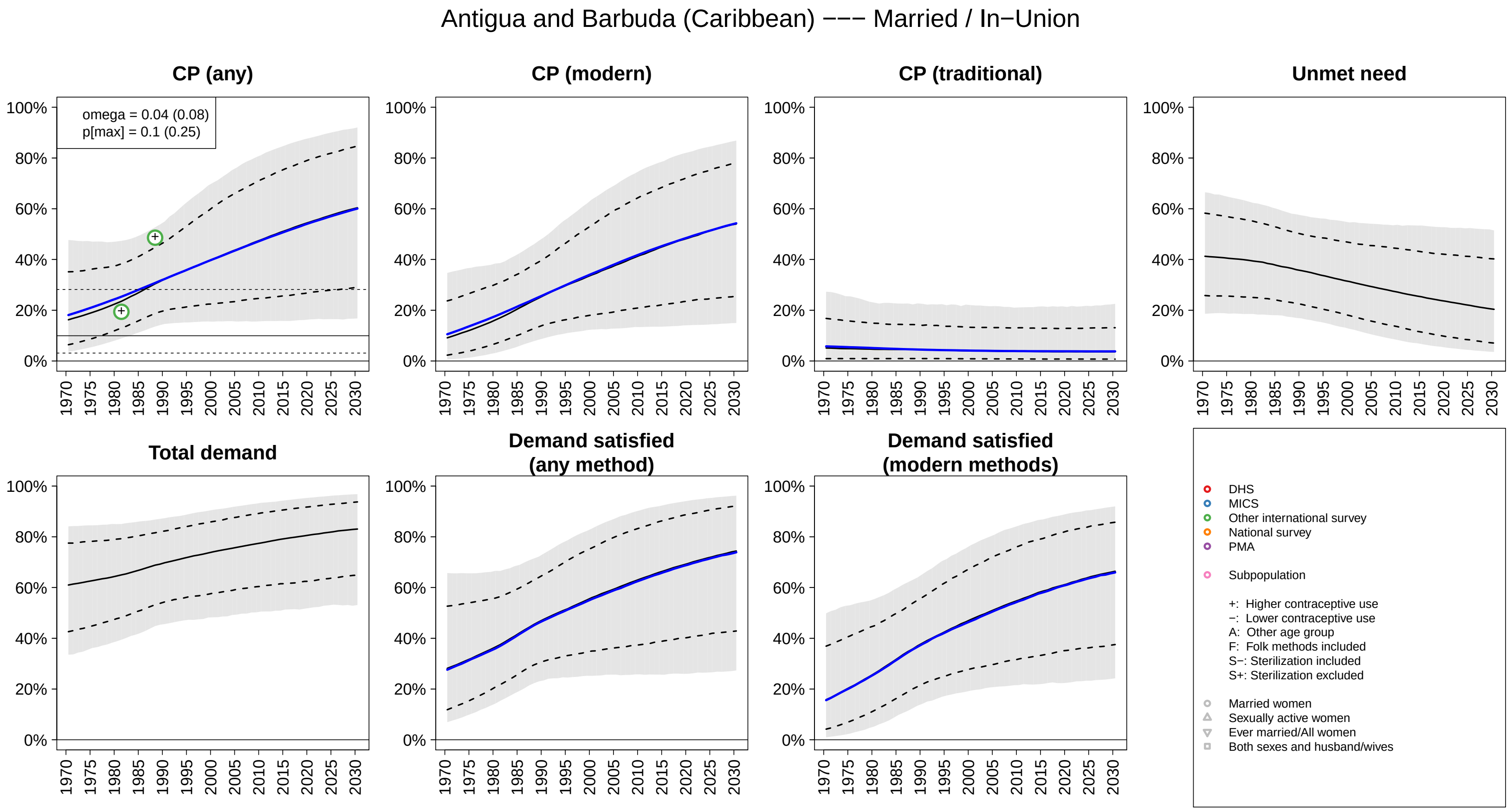
Antigua and Barbuda (Caribbean) −−− Married / In−Union
CP (any)
omega = 0.04 (0.08)
p[max] = 0.1 (0.25)
CP (modern)
CP (traditional)
Unmet need
Total demand
Demand satisfied
(any method)
Demand satisfied
(modern methods)
100%
80%
60%
40%
20%
0%
1970
1975
1980
1985
1990
1995
2000
2005
2010
2015
2020
2025
2030
DHS
MICS
Other international survey
National survey
PMA
Subpopulation
+: Higher contraceptive use
−: Lower contraceptive use
A: Other age group
F: Folk methods included
S−: Sterilization included
S+: Sterilization excluded
Married women
Sexually active women
Ever married/All women
Both sexes and husband/wives

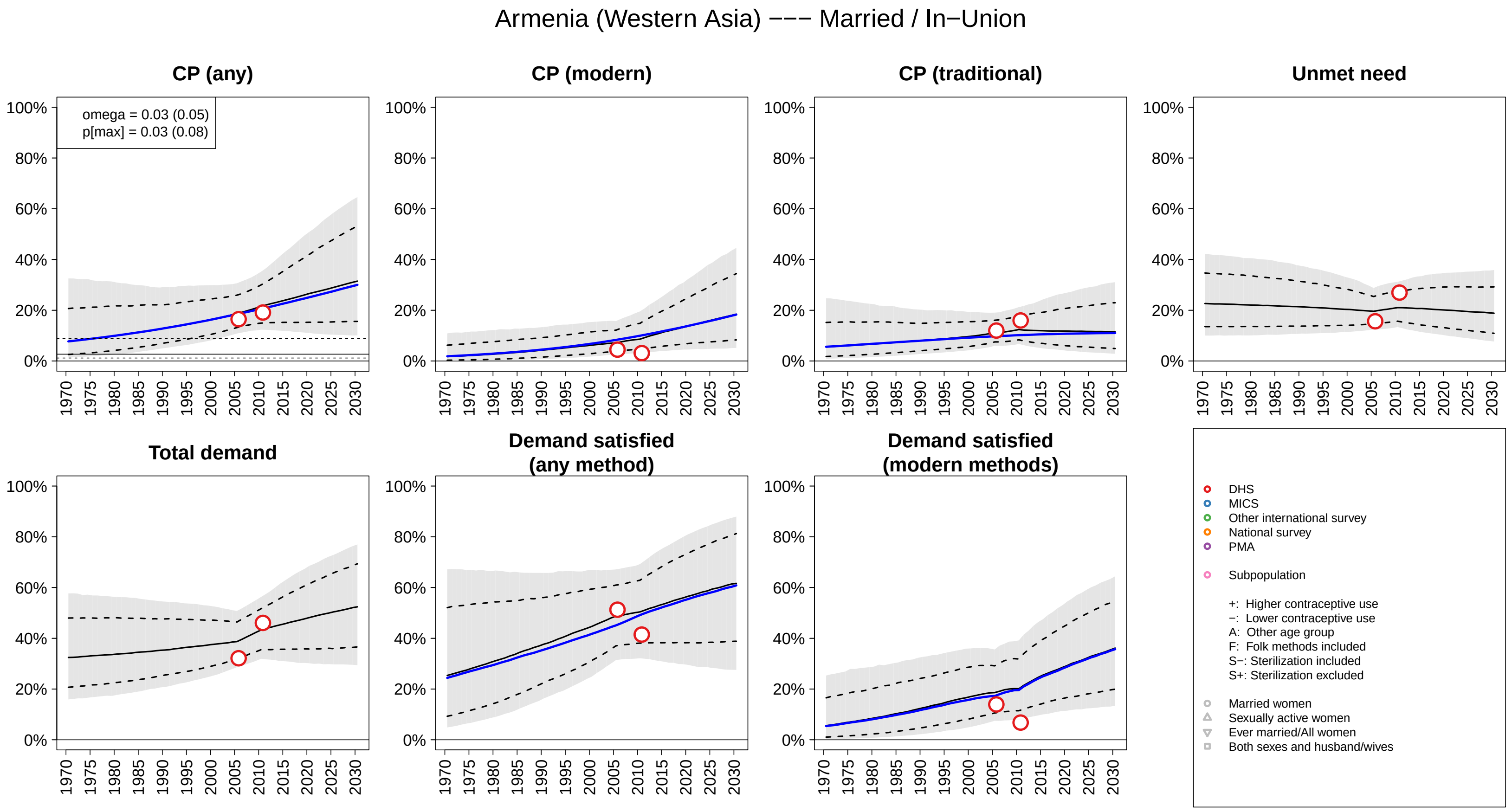
Armenia (Western Asia) --- Married / In−Union
CP (any)
omega = 0.03 (0.05)
p[max] = 0.03 (0.08)
CP (modern)
CP (traditional)
Unmet need
Total demand
Demand satisfied (any method)
Demand satisfied (modern methods)
DHS
MICS
Other international survey
National survey
PMA
Subpopulation
+: Higher contraceptive use
−: Lower contraceptive use
A: Other age group
F: Folk methods included
S−: Sterilization included
S+: Sterilization excluded
Married women
Sexually active women
Ever married/All women
Both sexes and husband/wives

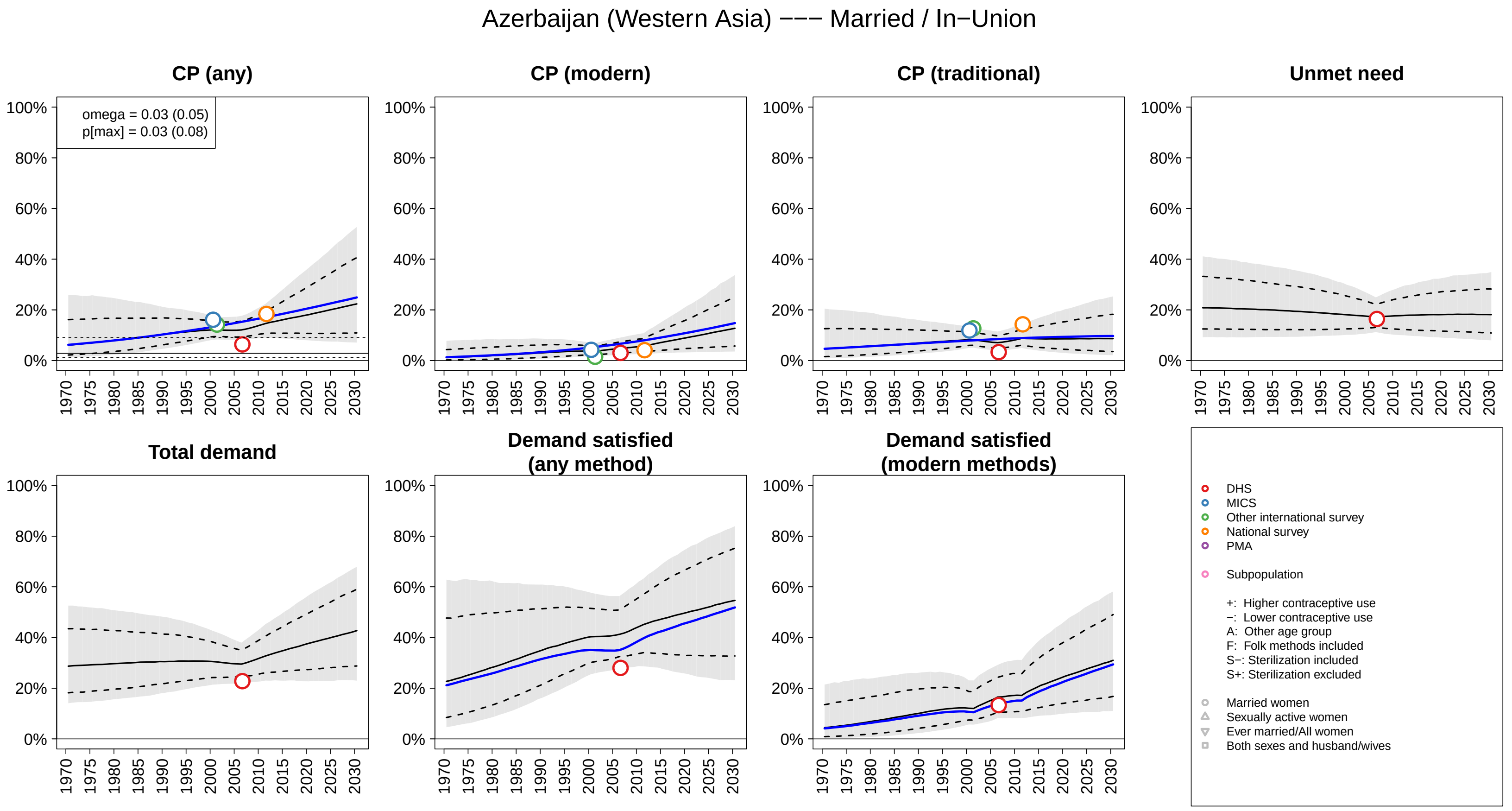
Azerbaijan (Western Asia) ––– Married / In−Union
CP (any)
omega = 0.03 (0.05)
p[max] = 0.03 (0.08)
CP (modern)
CP (traditional)
Unmet need
Total demand
Demand satisfied (any method)
Demand satisfied (modern methods)
DHS
MICS
Other international survey
National survey
PMA
Subpopulation
+: Higher contraceptive use
−: Lower contraceptive use
A: Other age group
F: Folk methods included
S−: Sterilization included
S+: Sterilization excluded
Married women
Sexually active women
Ever married/All women
Both sexes and husband/wives

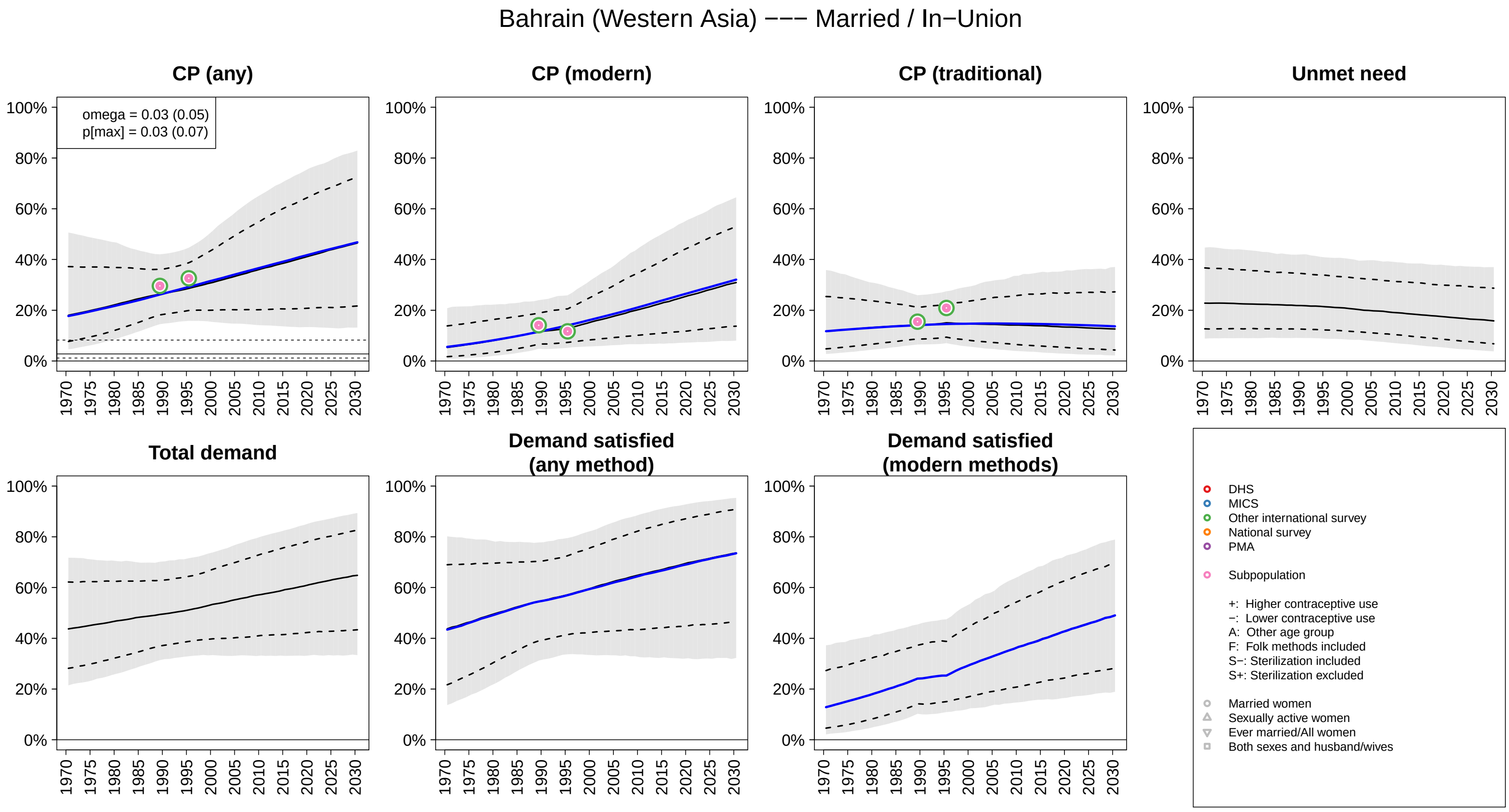

Bahrain (Western Asia) −−− Married / In−Union
CP (any)
omega = 0.03 (0.05)
p[max] = 0.03 (0.07)
CP (modern)
CP (traditional)
Unmet need
Total demand
Demand satisfied (any method)
Demand satisfied (modern methods)
0%
20%
40%
60%
80%
100%
1970
1975
1980
1985
1990
1995
2000
2005
2010
2015
2020
2025
2030
DHS
MICS
Other international survey
National survey
PMA
Subpopulation
+: Higher contraceptive use
−: Lower contraceptive use
A: Other age group
F: Folk methods included
S−: Sterilization included
S+: Sterilization excluded
Married women
Sexually active women
Ever married/All women
Both sexes and husband/wives

Bangladesh (Southern Asia) −−− Married / In−Union

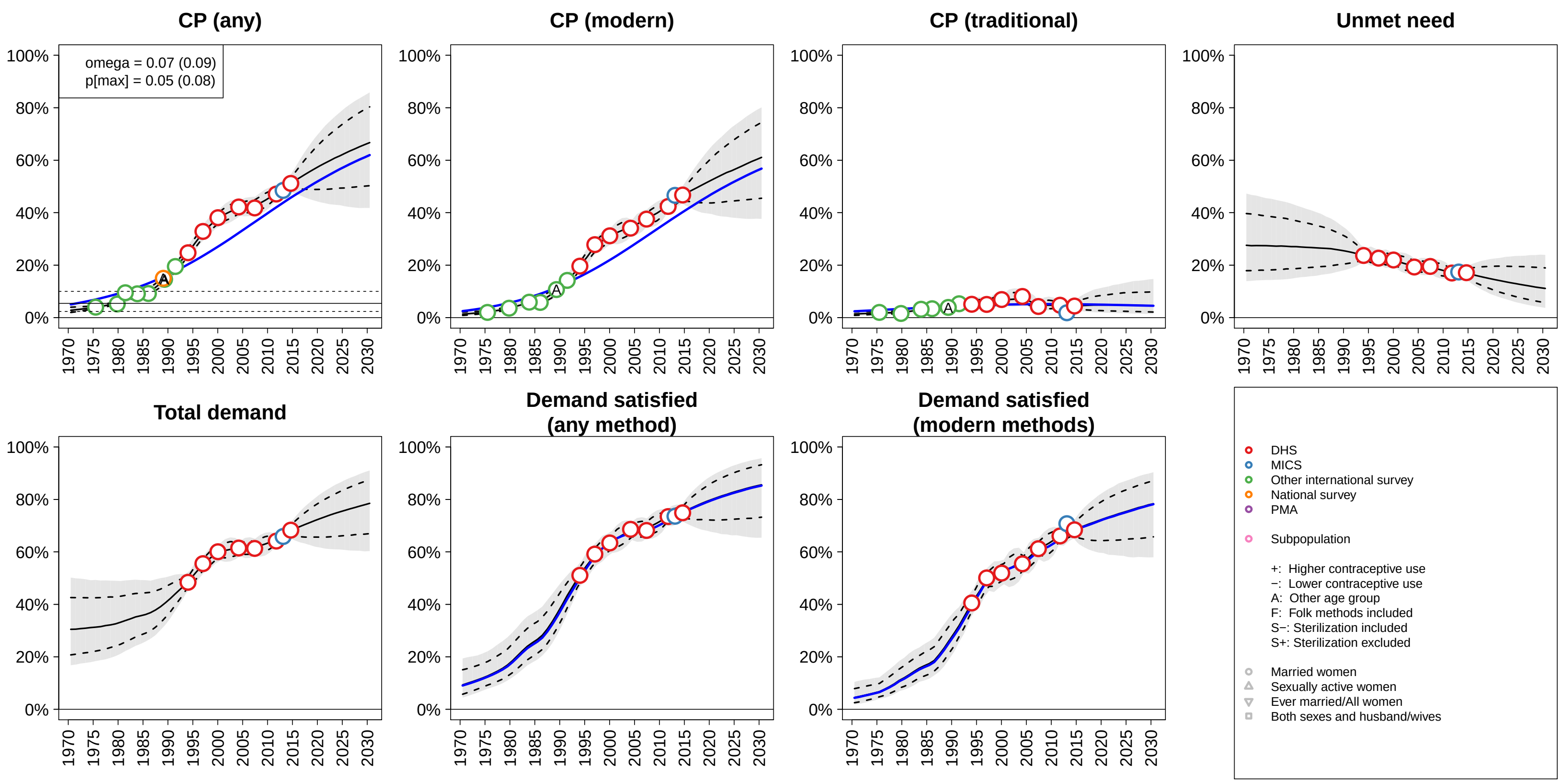

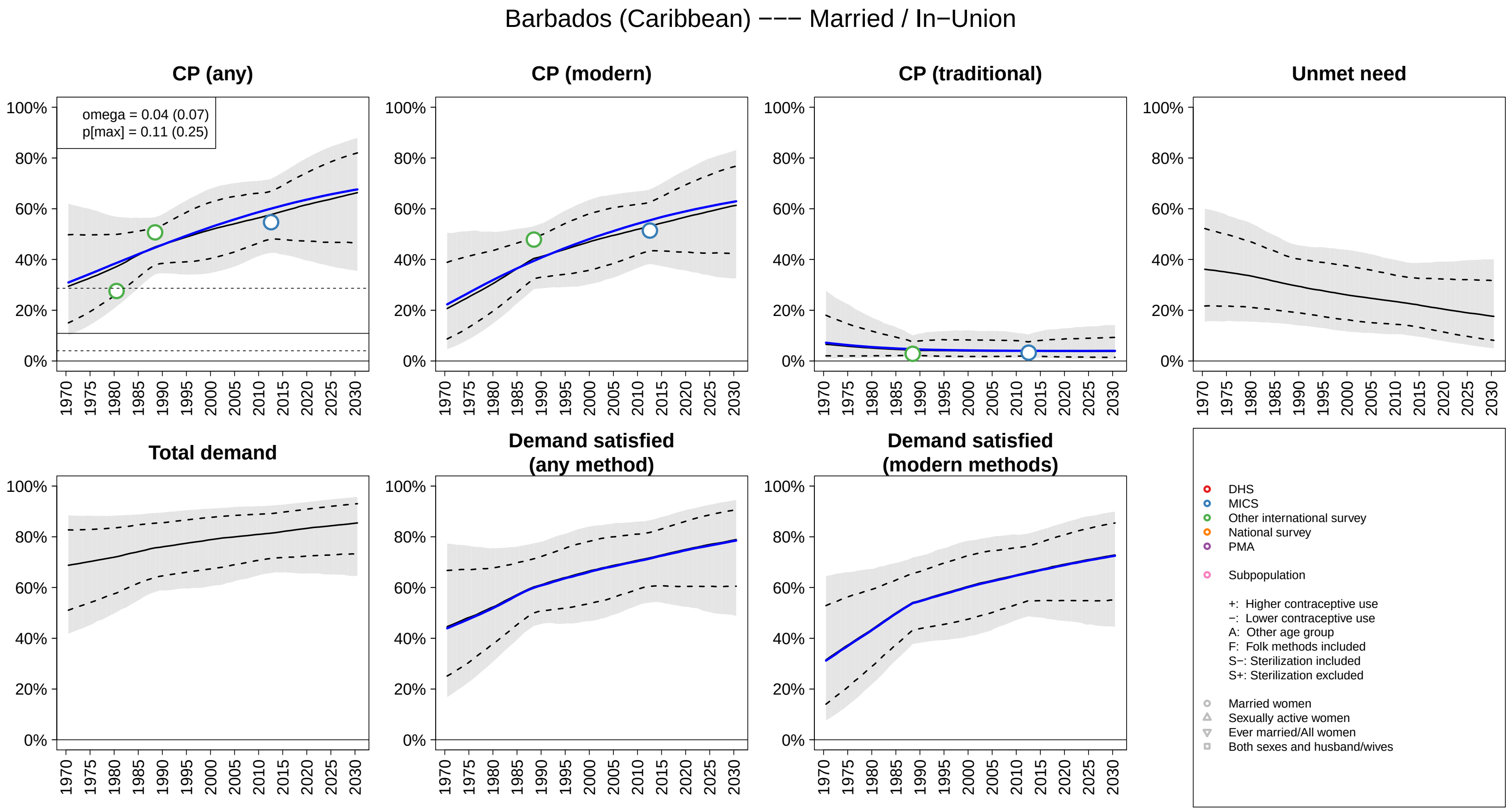
Barbados (Caribbean) ––– Married / In–Union
CP (any)
omega = 0.04 (0.07)
p[max] = 0.11 (0.25)
CP (modern)
CP (traditional)
Unmet need
Total demand
Demand satisfied (any method)
Demand satisfied (modern methods)
DHS
MICS
Other international survey
National survey
PMA
Subpopulation
+: Higher contraceptive use
−: Lower contraceptive use
A: Other age group
F: Folk methods included
S−: Sterilization included
S+: Sterilization excluded
Married women
Sexually active women
Ever married/All women
Both sexes and husband/wives

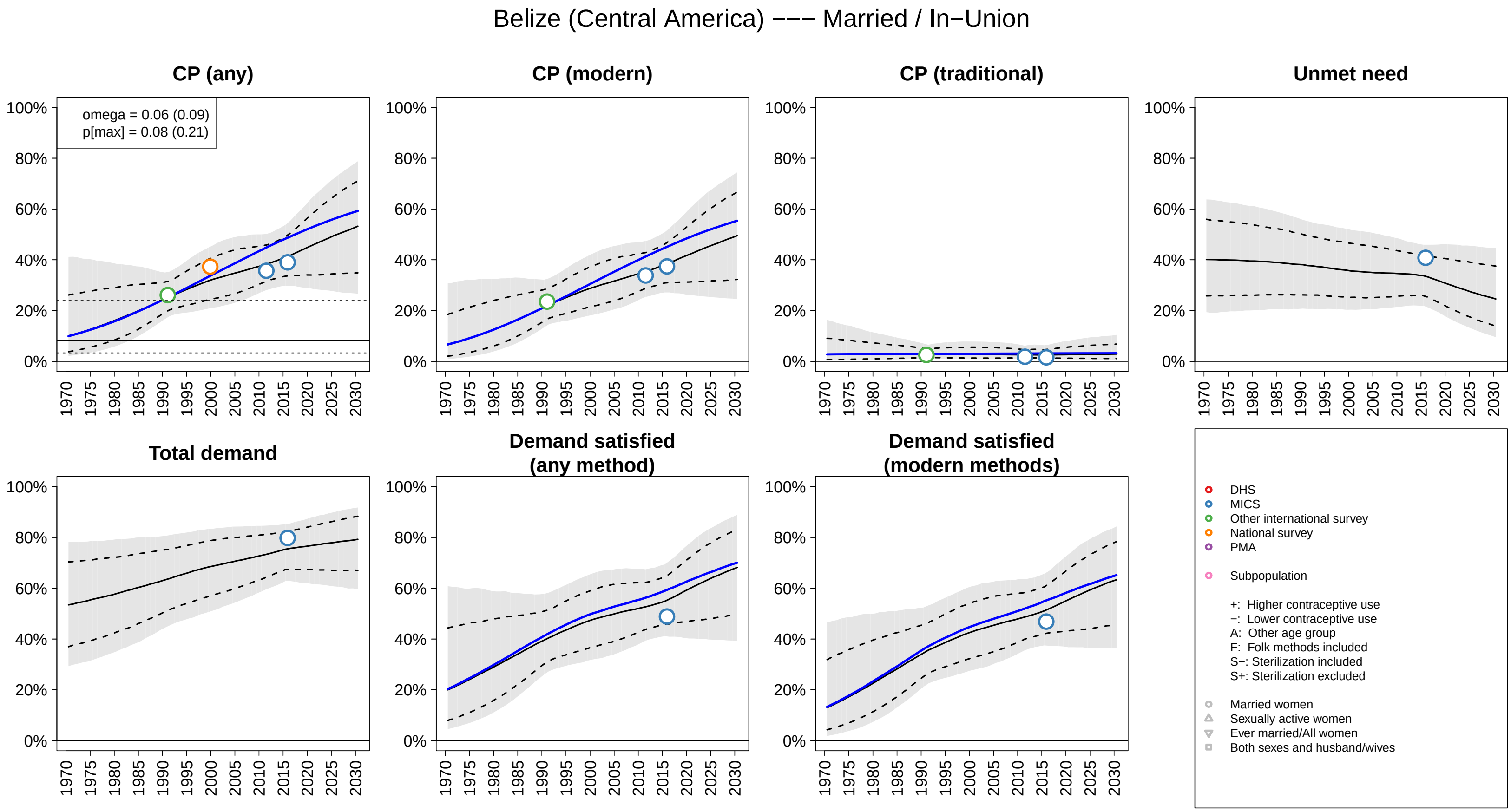

Belize (Central America) --- Married / In−Union
CP (any)
omega = 0.06 (0.09)
p[max] = 0.08 (0.21)
CP (modern)
CP (traditional)
Unmet need
Total demand
Demand satisfied (any method)
Demand satisfied (modern methods)
DHS
MICS
Other international survey
National survey
PMA
Subpopulation
+: Higher contraceptive use
−: Lower contraceptive use
A: Other age group
F: Folk methods included
S−: Sterilization included
S+: Sterilization excluded
Married women
Sexually active women
Ever married/All women
Both sexes and husband/wives

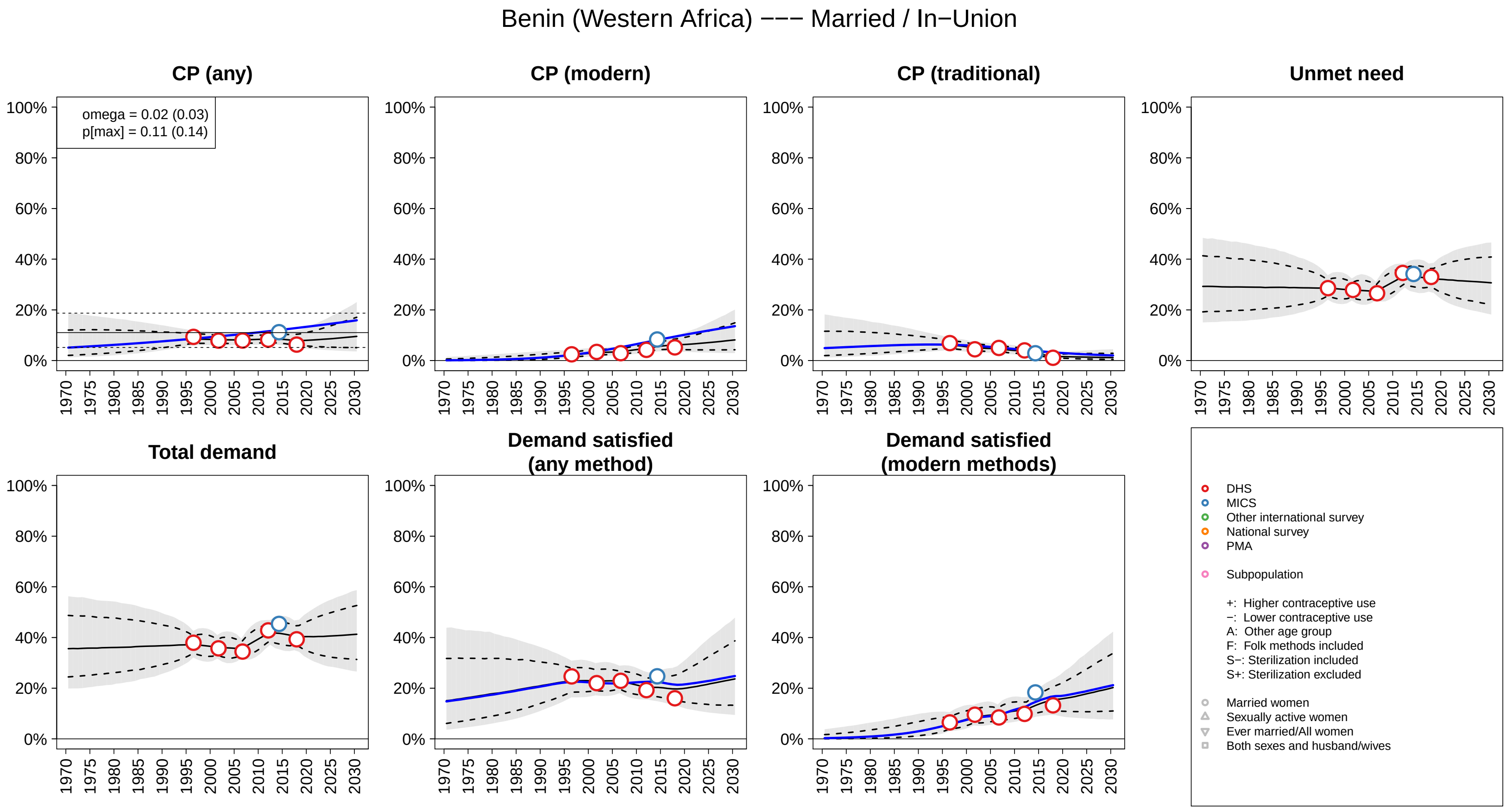
Benin (Western Africa) --- Married / In−Union
CP (any)
omega = 0.02 (0.03)
p[max] = 0.11 (0.14)
CP (modern)
CP (traditional)
Unmet need
Total demand
Demand satisfied (any method)
Demand satisfied (modern methods)
DHS
MICS
Other international survey
National survey
PMA
Subpopulation
+: Higher contraceptive use
−: Lower contraceptive use
A: Other age group
F: Folk methods included
S−: Sterilization included
S+: Sterilization excluded
Married women
Sexually active women
Ever married/All women
Both sexes and husband/wives

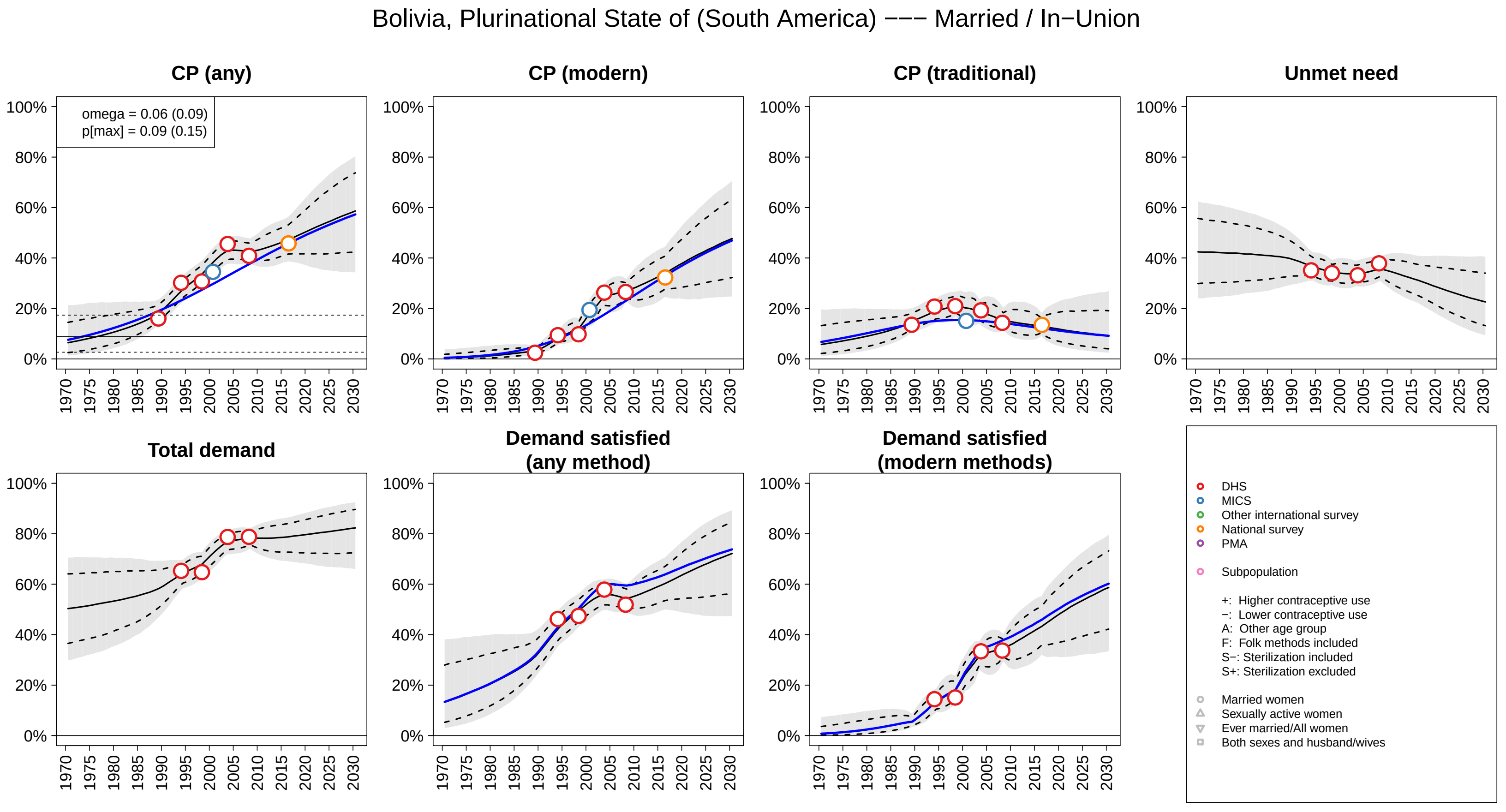
Bolivia, Plurinational State of (South America) --- Married / In−Union
CP (any)
omega = 0.06 (0.09)
p[max] = 0.09 (0.15)
CP (modern)
CP (traditional)
Unmet need
Total demand
Demand satisfied (any method)
Demand satisfied (modern methods)
DHS
MICS
Other international survey
National survey
PMA
Subpopulation
+: Higher contraceptive use
−: Lower contraceptive use
A: Other age group
F: Folk methods included
S−: Sterilization included
S+: Sterilization excluded
Married women
Sexually active women
Ever married/All women
Both sexes and husband/wives

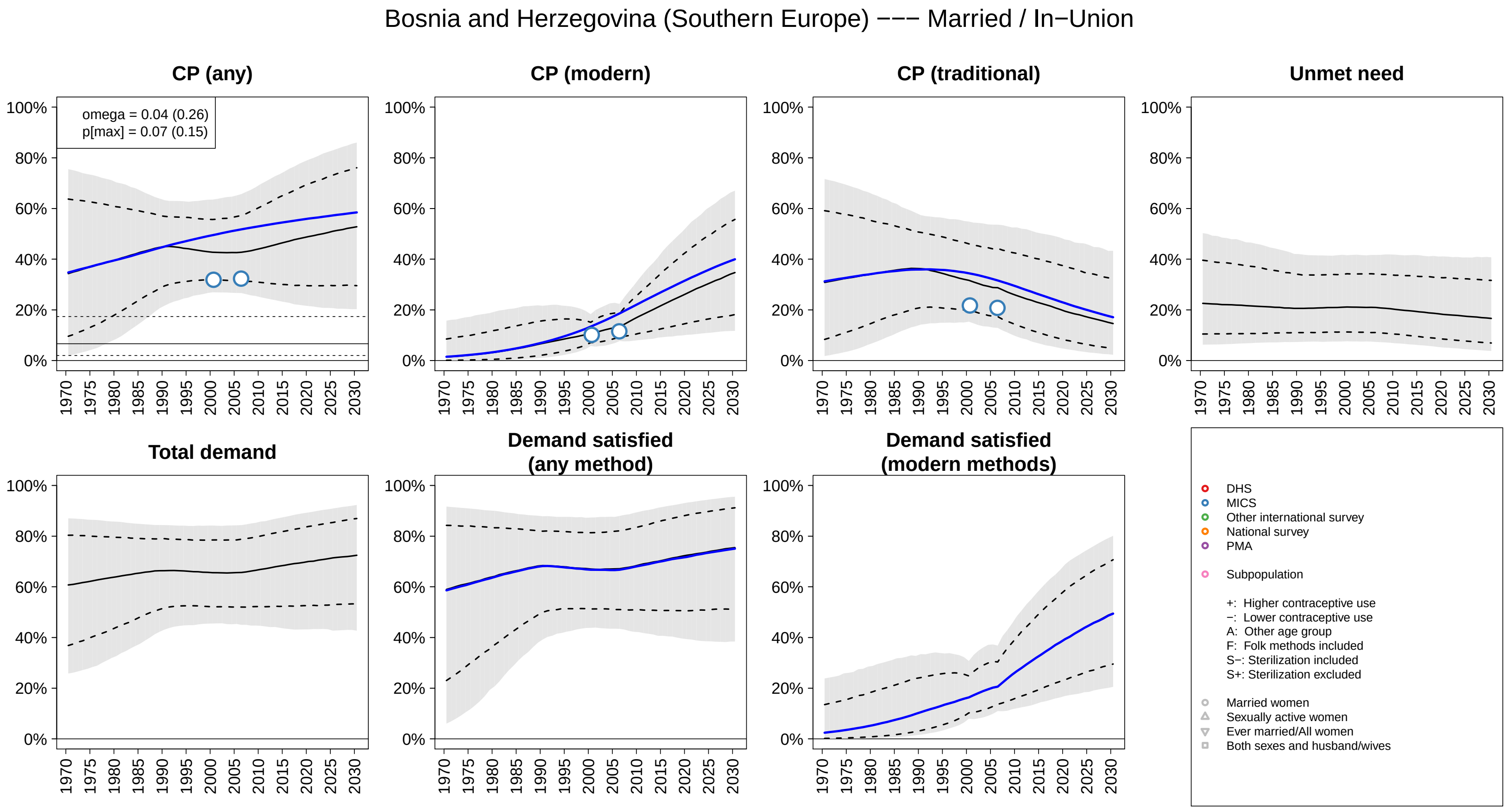
Bosnia and Herzegovina (Southern Europe) −−− Married / In−Union
CP (any)
omega = 0.04 (0.26)
p[max] = 0.07 (0.15)
CP (modern)
CP (traditional)
Unmet need
Total demand
Demand satisfied (any method)
Demand satisfied (modern methods)
DHS
MICS
Other international survey
National survey
PMA
Subpopulation
+: Higher contraceptive use
−: Lower contraceptive use
A: Other age group
F: Folk methods included
S−: Sterilization included
S+: Sterilization excluded
Married women
Sexually active women
Ever married/All women
Both sexes and husband/wives

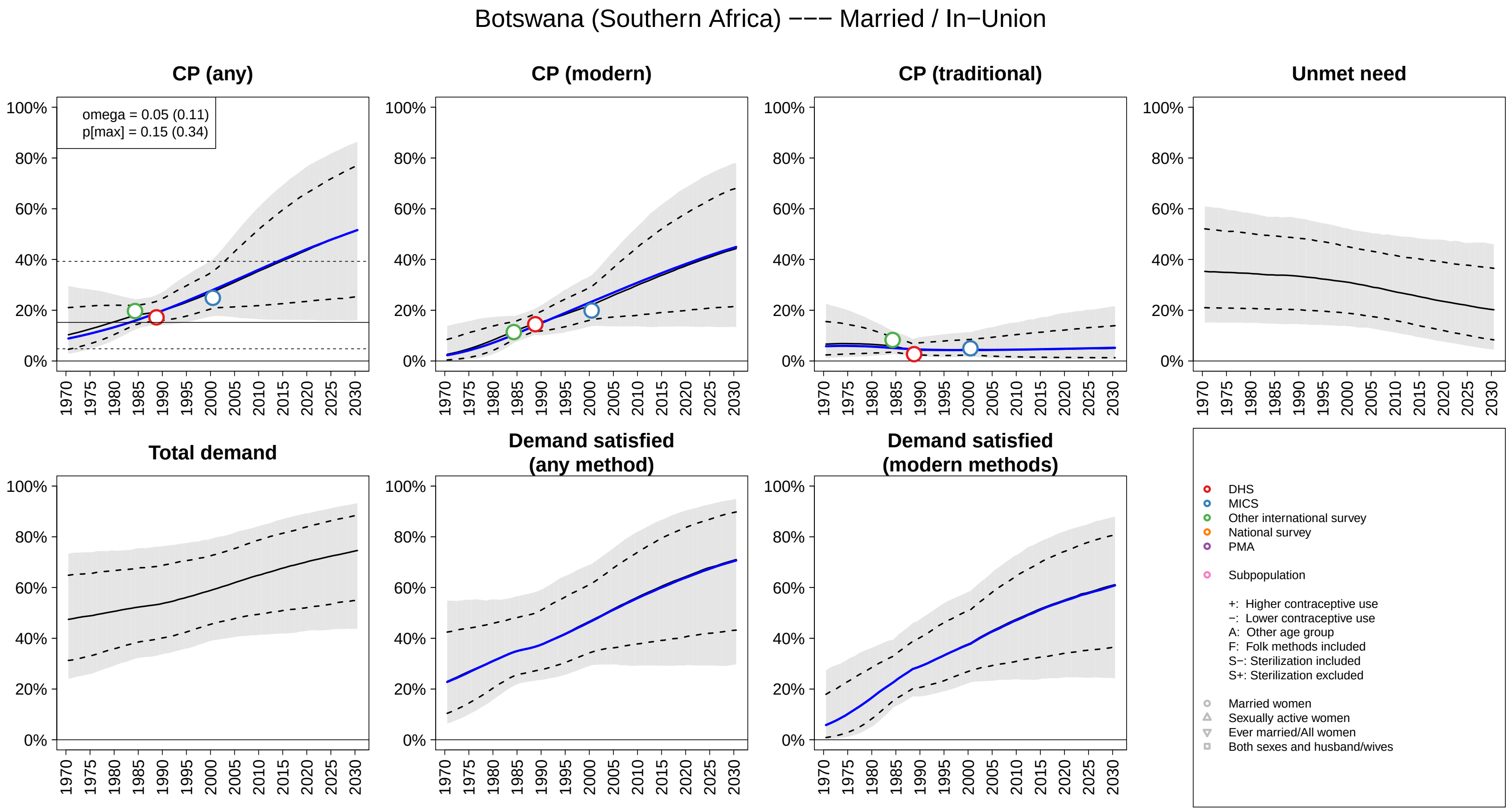
Botswana (Southern Africa) --- Married / In−Union
CP (any)
omega = 0.05 (0.11)
p[max] = 0.15 (0.34)
CP (modern)
CP (traditional)
Unmet need
Total demand
Demand satisfied (any method)
Demand satisfied (modern methods)
0%
20%
40%
60%
80%
100%
1970
1975
1980
1985
1990
1995
2000
2005
2010
2015
2020
2025
2030
DHS
MICS
Other international survey
National survey
PMA
Subpopulation
+: Higher contraceptive use
−: Lower contraceptive use
A: Other age group
F: Folk methods included
S−: Sterilization included
S+: Sterilization excluded
Married women
Sexually active women
Ever married/All women
Both sexes and husband/wives

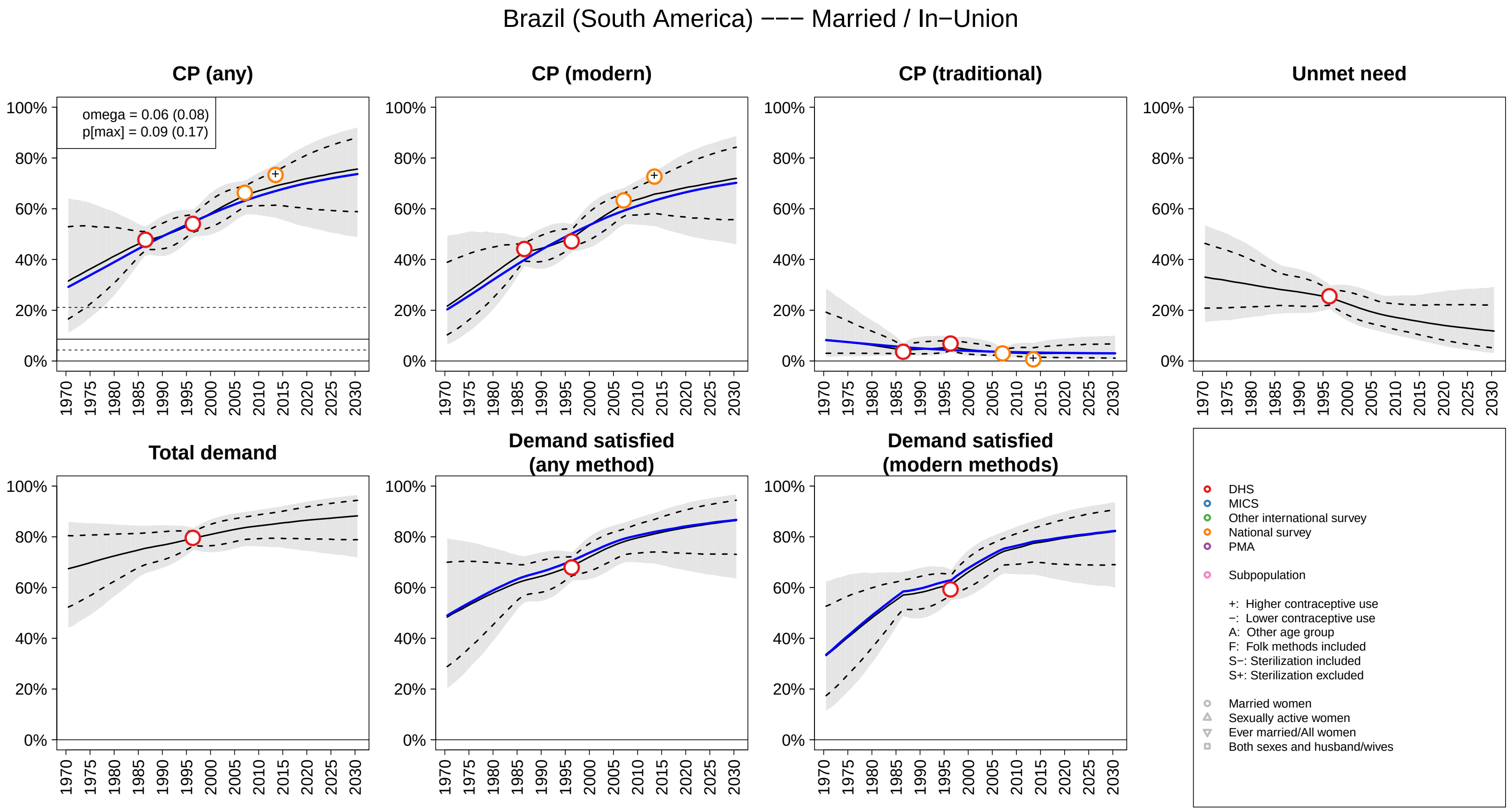
Brazil (South America) −−− Married / In−Union
CP (any)
omega = 0.06 (0.08)
p[max] = 0.09 (0.17)
CP (modern)
CP (traditional)
Unmet need
Total demand
Demand satisfied (any method)
Demand satisfied (modern methods)
DHS
MICS
Other international survey
National survey
PMA
Subpopulation
+: Higher contraceptive use
−: Lower contraceptive use
A: Other age group
F: Folk methods included
S−: Sterilization included
S+: Sterilization excluded
Married women
Sexually active women
Ever married/All women
Both sexes and husband/wives

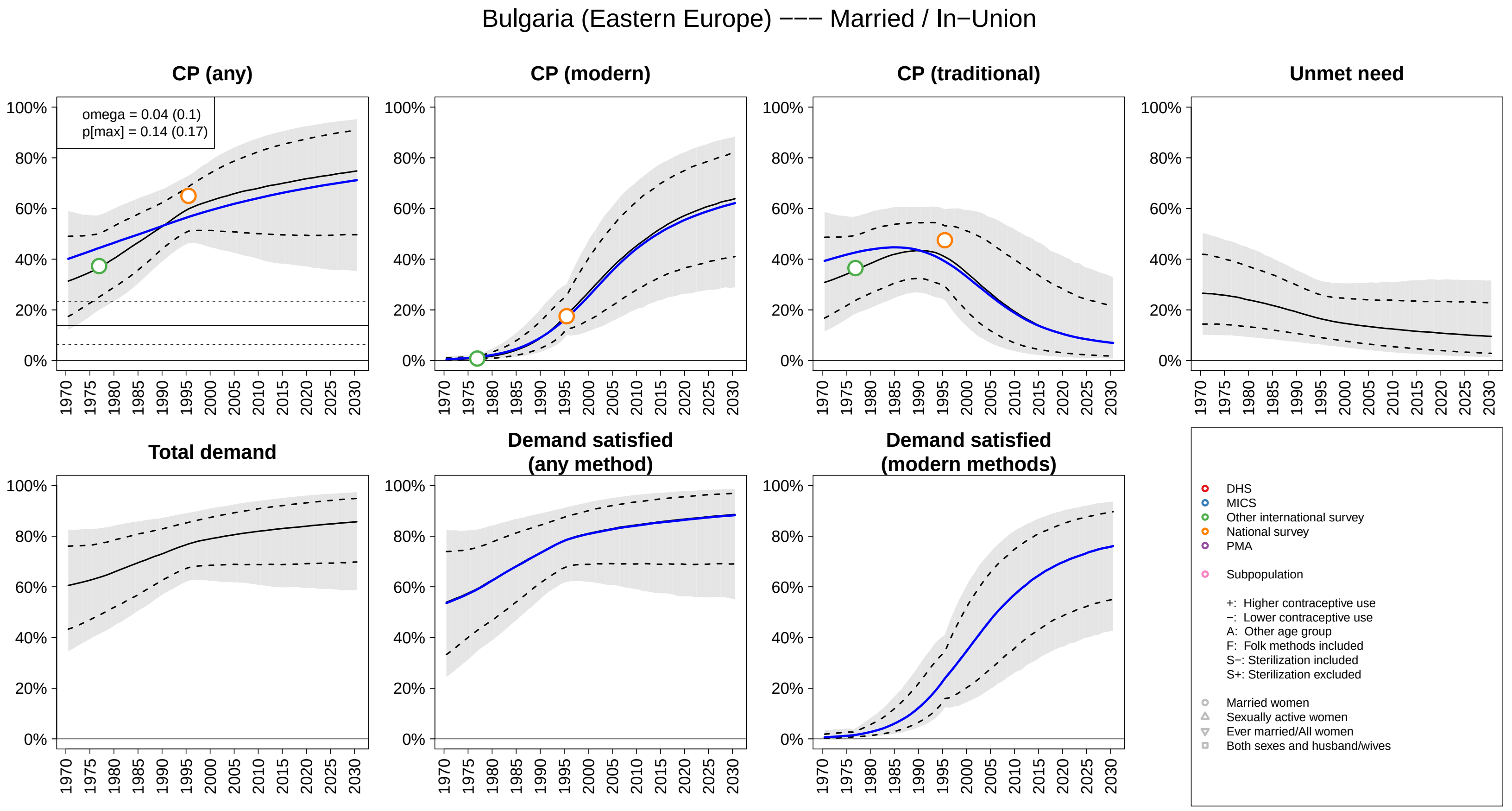
Bulgaria (Eastern Europe) −−− Married / In−Union
CP (any)
omega = 0.04 (0.1)
p[max] = 0.14 (0.17)
CP (modern)
CP (traditional)
Unmet need
Total demand
Demand satisfied (any method)
Demand satisfied (modern methods)
DHS
MICS
Other international survey
National survey
PMA
Subpopulation
+: Higher contraceptive use
−: Lower contraceptive use
A: Other age group
F: Folk methods included
S−: Sterilization included
S+: Sterilization excluded
Married women
Sexually active women
Ever married/All women
Both sexes and husband/wives

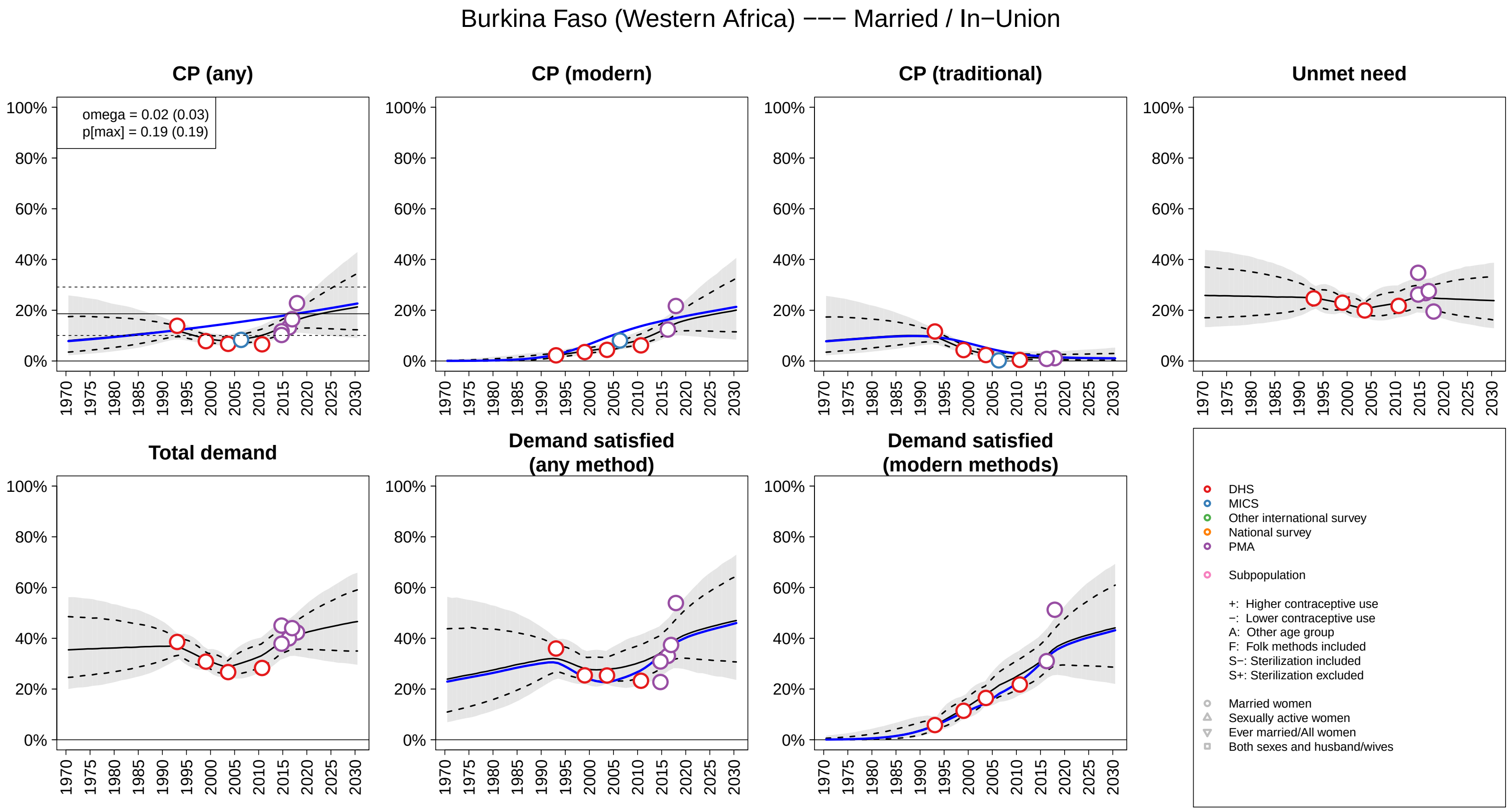
Burkina Faso (Western Africa) --- Married / In−Union
CP (any)
omega = 0.02 (0.03)
p[max] = 0.19 (0.19)
CP (modern)
CP (traditional)
Unmet need
Total demand
Demand satisfied (any method)
Demand satisfied (modern methods)
DHS
MICS
Other international survey
National survey
PMA
Subpopulation
+: Higher contraceptive use
−: Lower contraceptive use
A: Other age group
F: Folk methods included
S−: Sterilization included
S+: Sterilization excluded
Married women
Sexually active women
Ever married/All women
Both sexes and husband/wives

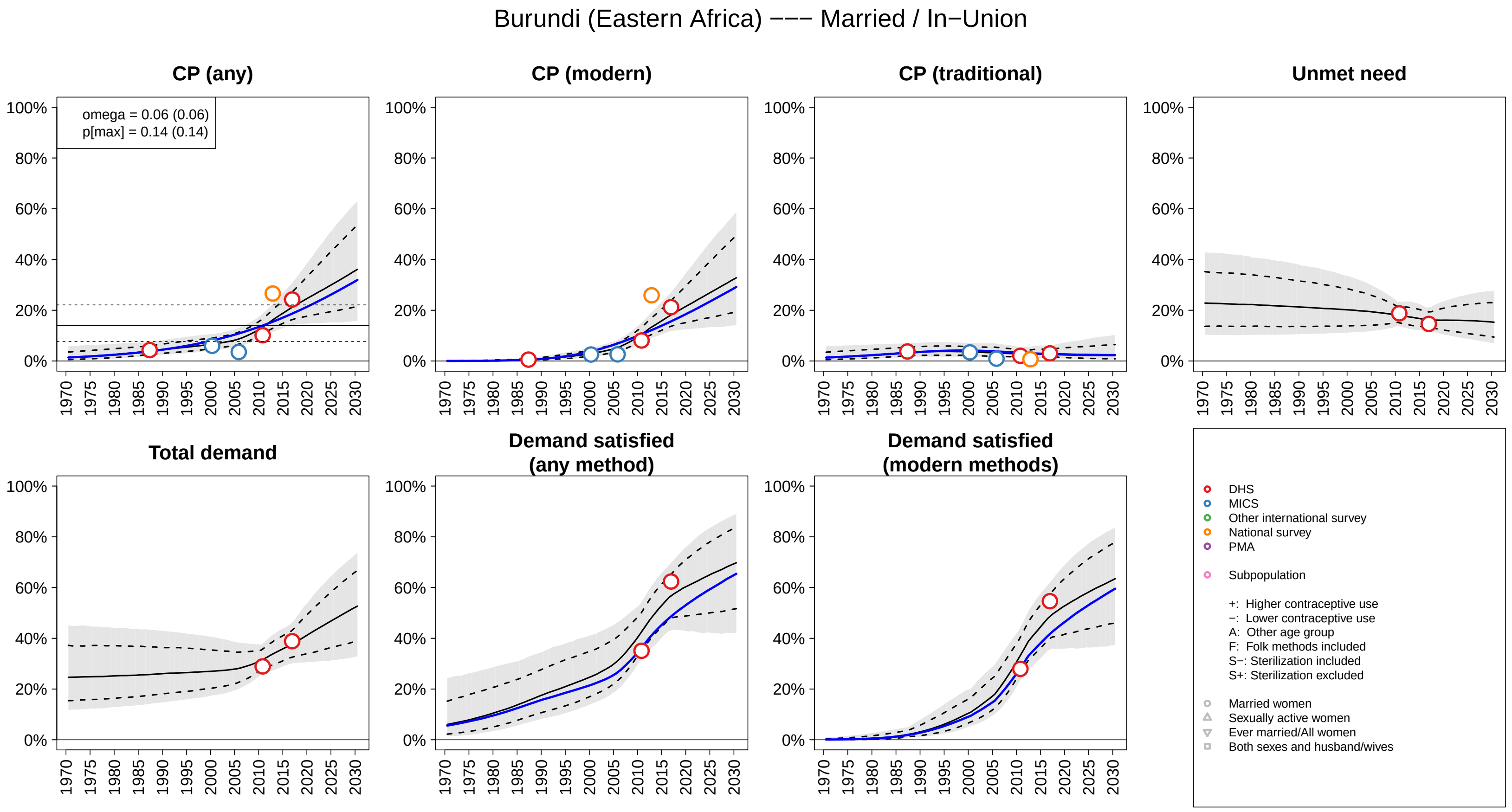

Burundi (Eastern Africa) −−− Married / In−Union
CP (any)
omega = 0.06 (0.06)
p[max] = 0.14 (0.14)
CP (modern)
CP (traditional)
Unmet need
Total demand
Demand satisfied (any method)
Demand satisfied (modern methods)
DHS
MICS
Other international survey
National survey
PMA
Subpopulation
+: Higher contraceptive use
−: Lower contraceptive use
A: Other age group
F: Folk methods included
S−: Sterilization included
S+: Sterilization excluded
Married women
Sexually active women
Ever married/All women
Both sexes and husband/wives

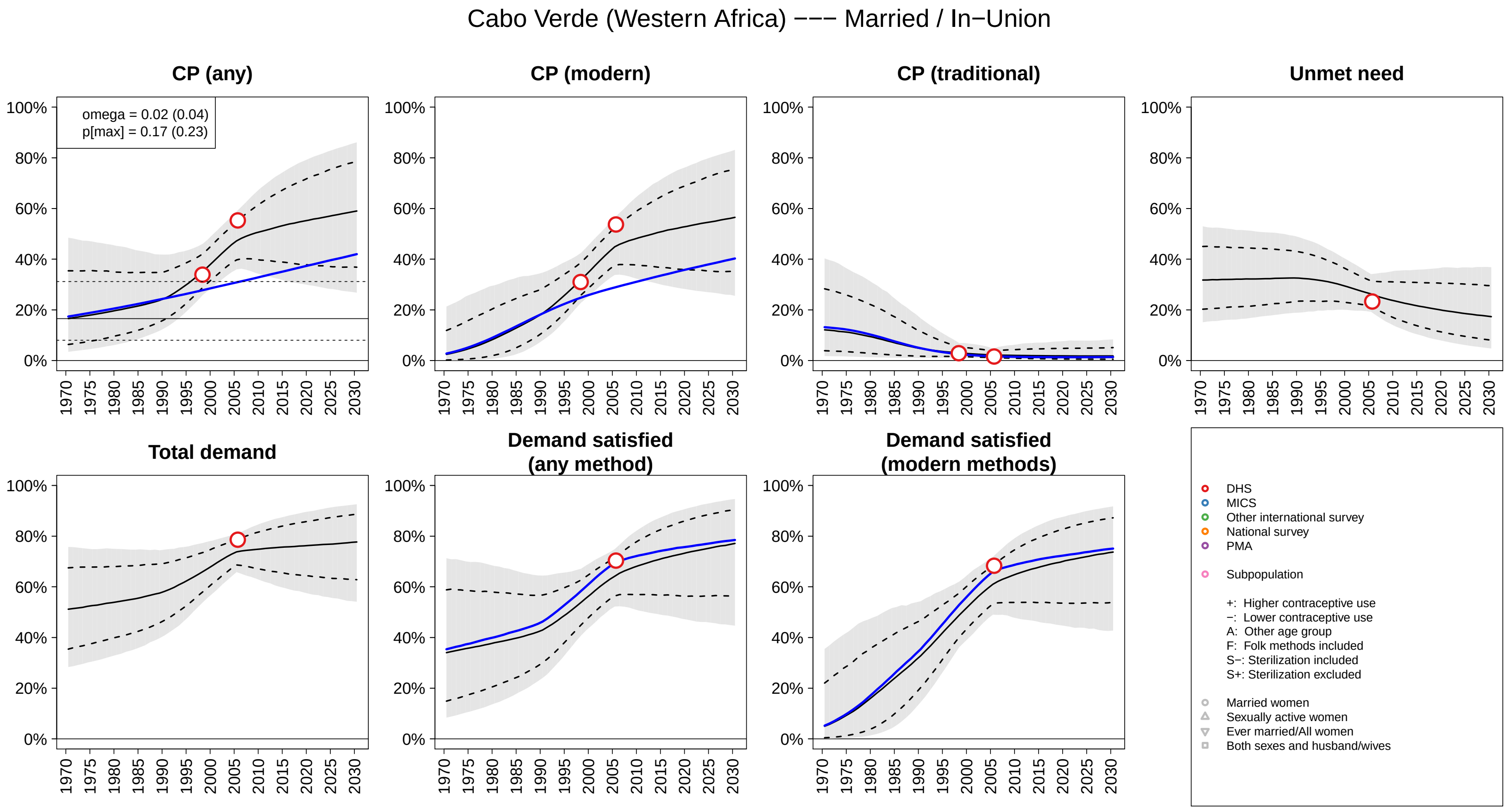
Cabo Verde (Western Africa) --- Married / In−Union
CP (any)
omega = 0.02 (0.04)
p[max] = 0.17 (0.23)
CP (modern)
CP (traditional)
Unmet need
Total demand
Demand satisfied (any method)
Demand satisfied (modern methods)
DHS
MICS
Other international survey
National survey
PMA
Subpopulation
+: Higher contraceptive use
−: Lower contraceptive use
A: Other age group
F: Folk methods included
S−: Sterilization included
S+: Sterilization excluded
Married women
Sexually active women
Ever married/All women
Both sexes and husband/wives

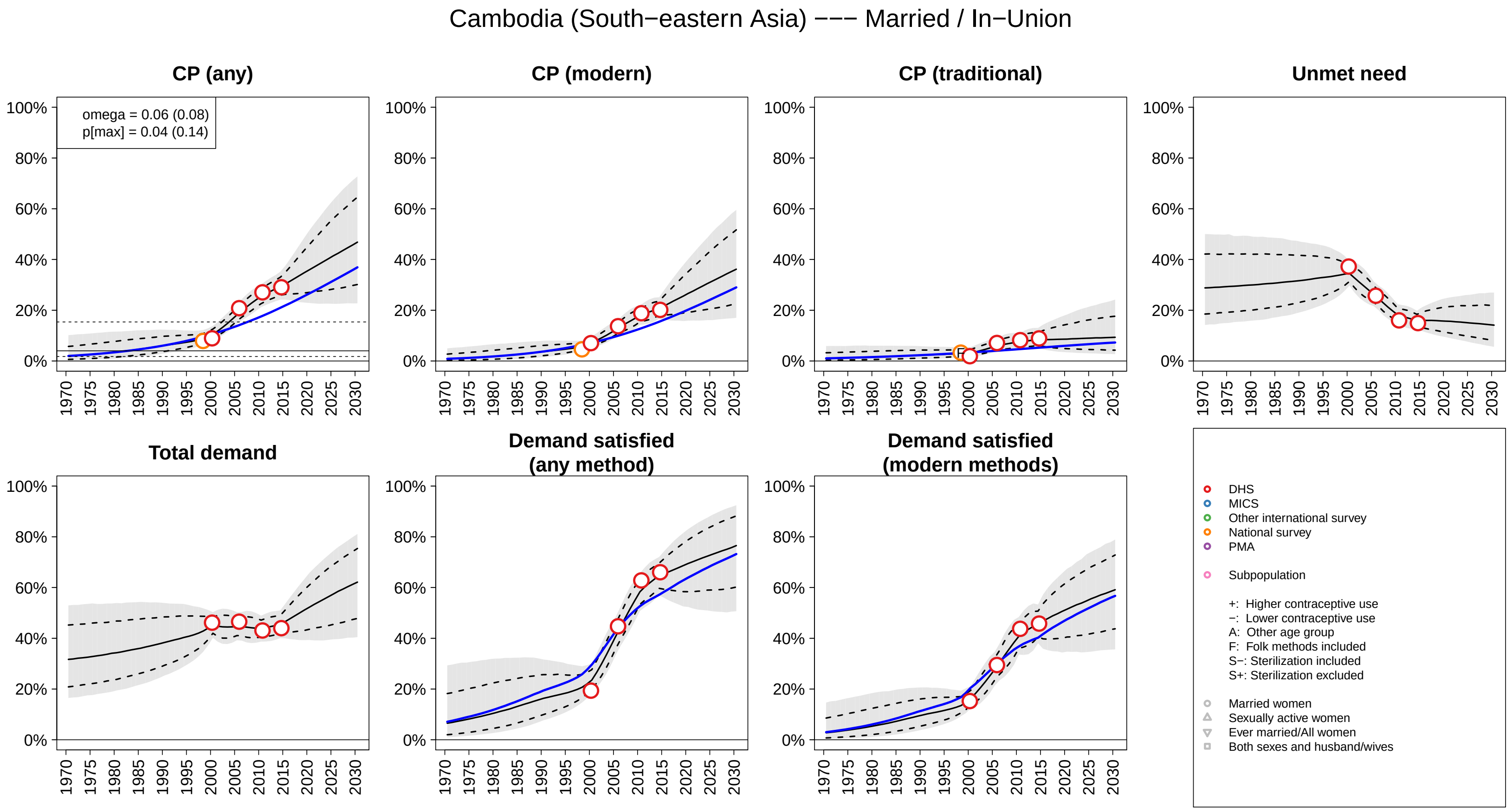
Cambodia (South−eastern Asia) −−− Married / In−Union
CP (any)
omega = 0.06 (0.08)
p[max] = 0.04 (0.14)
CP (modern)
CP (traditional)
Unmet need
Total demand
Demand satisfied (any method)
Demand satisfied (modern methods)
DHS
MICS
Other international survey
National survey
PMA
Subpopulation
+: Higher contraceptive use
−: Lower contraceptive use
A: Other age group
F: Folk methods included
S−: Sterilization included
S+: Sterilization excluded
Married women
Sexually active women
Ever married/All women
Both sexes and husband/wives

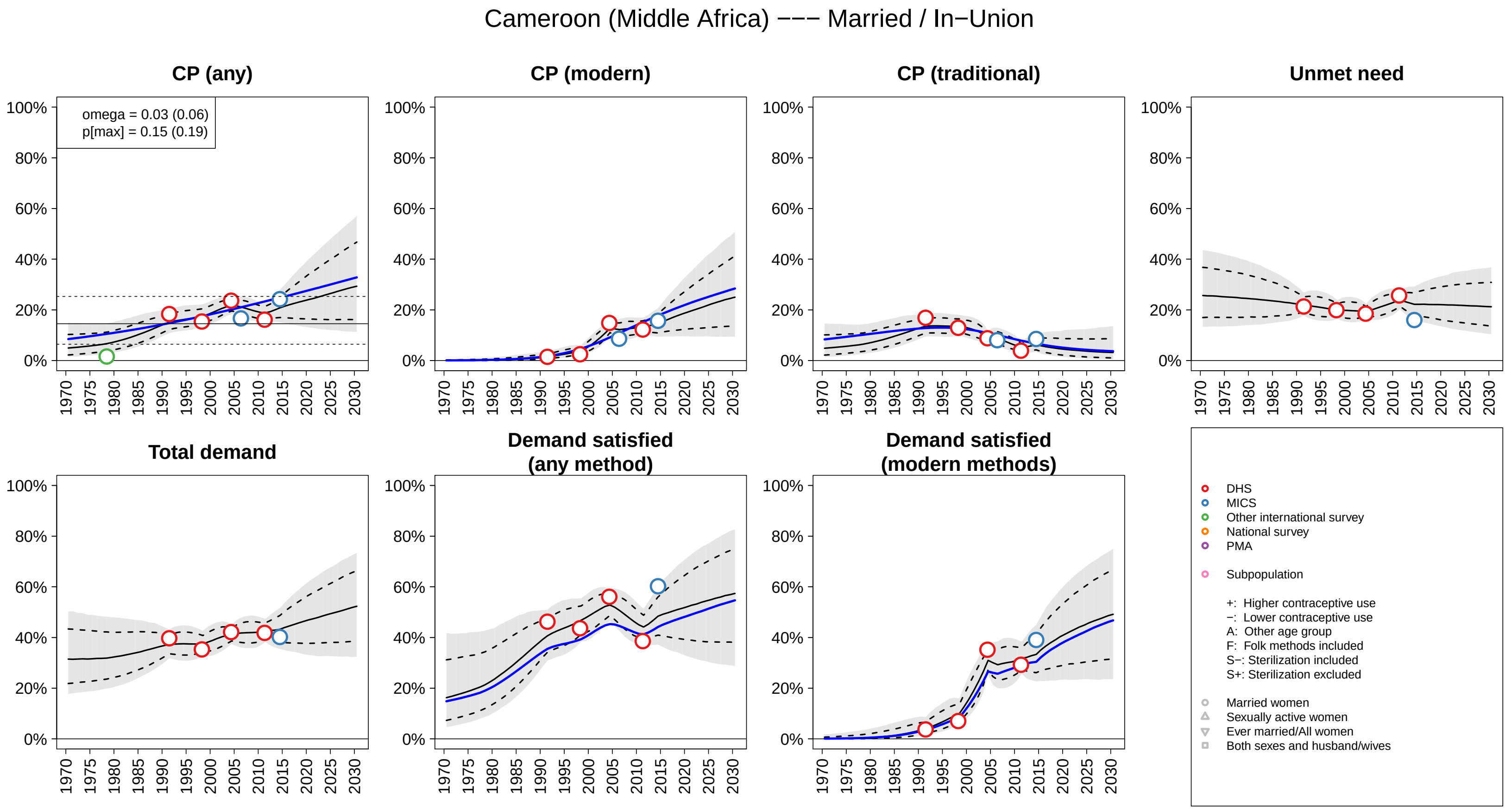
Cameroon (Middle Africa) −−− Married / In−Union
CP (any)
omega = 0.03 (0.06)
p[max] = 0.15 (0.19)
CP (modern)
CP (traditional)
Unmet need
Total demand
Demand satisfied (any method)
Demand satisfied (modern methods)
DHS
MICS
Other international survey
National survey
PMA
Subpopulation
+: Higher contraceptive use
−: Lower contraceptive use
A: Other age group
F: Folk methods included
S−: Sterilization included
S+: Sterilization excluded
Married women
Sexually active women
Ever married/All women
Both sexes and husband/wives

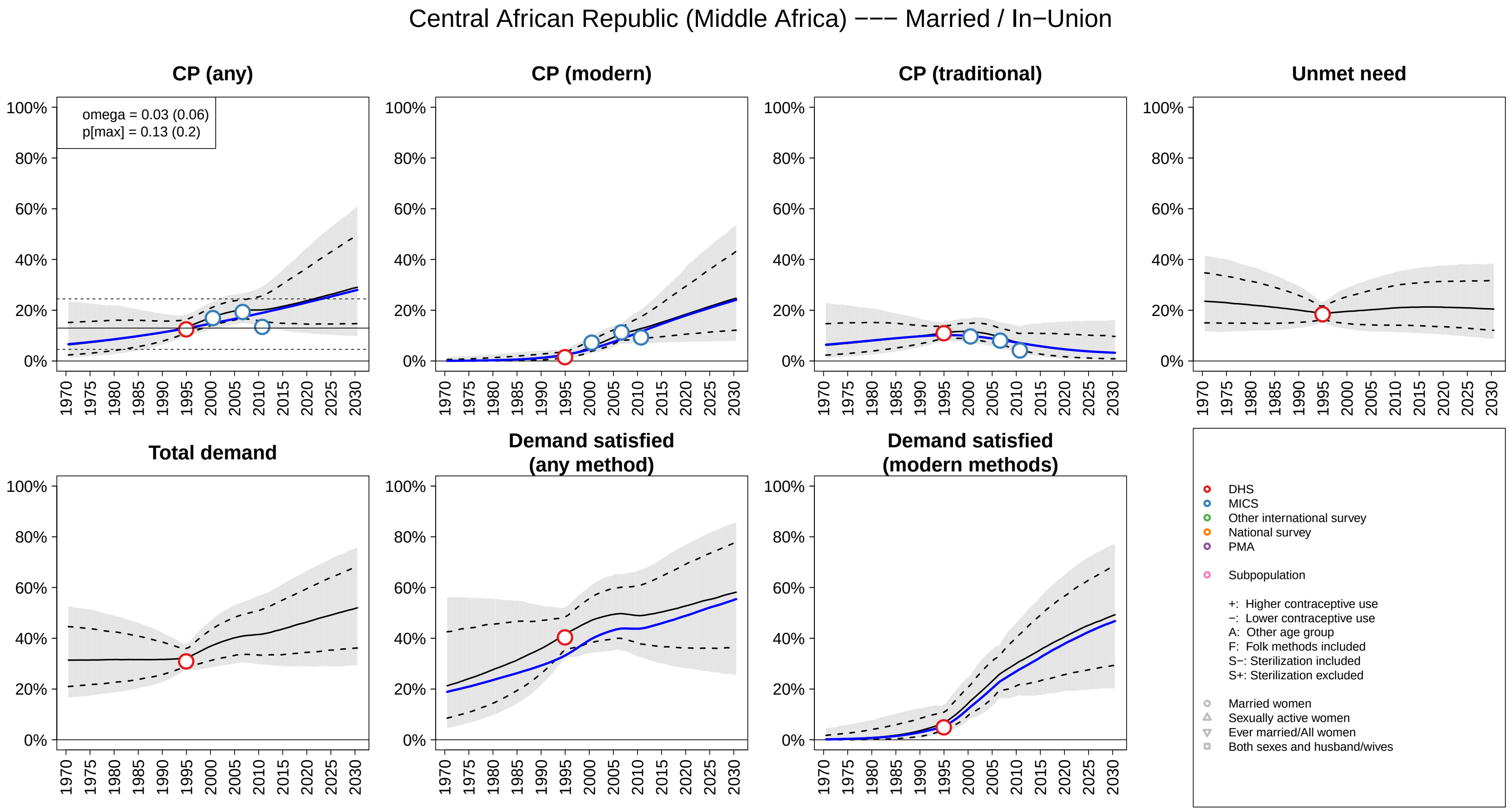

Central African Republic (Middle Africa) --- Married / In−Union
CP (any)
omega = 0.03 (0.06)
p[max] = 0.13 (0.2)
CP (modern)
CP (traditional)
Unmet need
Total demand
Demand satisfied (any method)
Demand satisfied (modern methods)
DHS
MICS
Other international survey
National survey
PMA
Subpopulation
+: Higher contraceptive use
−: Lower contraceptive use
A: Other age group
F: Folk methods included
S−: Sterilization included
S+: Sterilization excluded
Married women
Sexually active women
Ever married/All women
Both sexes and husband/wives

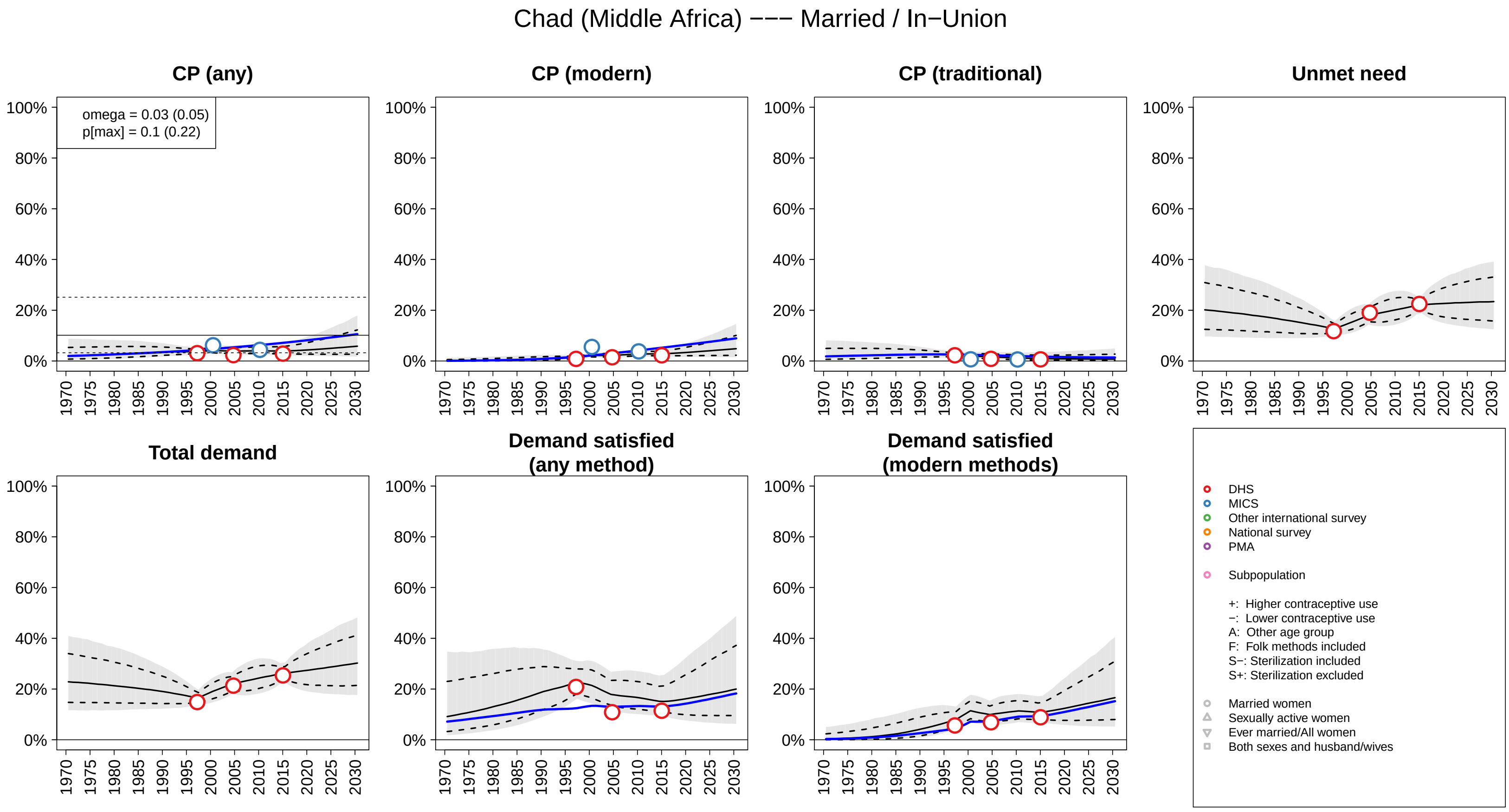
Chad (Middle Africa) --- Married / In−Union
CP (any)
omega = 0.03 (0.05)
p[max] = 0.1 (0.22)
CP (modern)
CP (traditional)
Unmet need
Total demand
Demand satisfied (any method)
Demand satisfied (modern methods)
0%
20%
40%
60%
80%
100%
1970
1975
1980
1985
1990
1995
2000
2005
2010
2015
2020
2025
2030
DHS
MICS
Other international survey
National survey
PMA
Subpopulation
+: Higher contraceptive use
−: Lower contraceptive use
A: Other age group
F: Folk methods included
S−: Sterilization included
S+: Sterilization excluded
Married women
Sexually active women
Ever married/All women
Both sexes and husband/wives

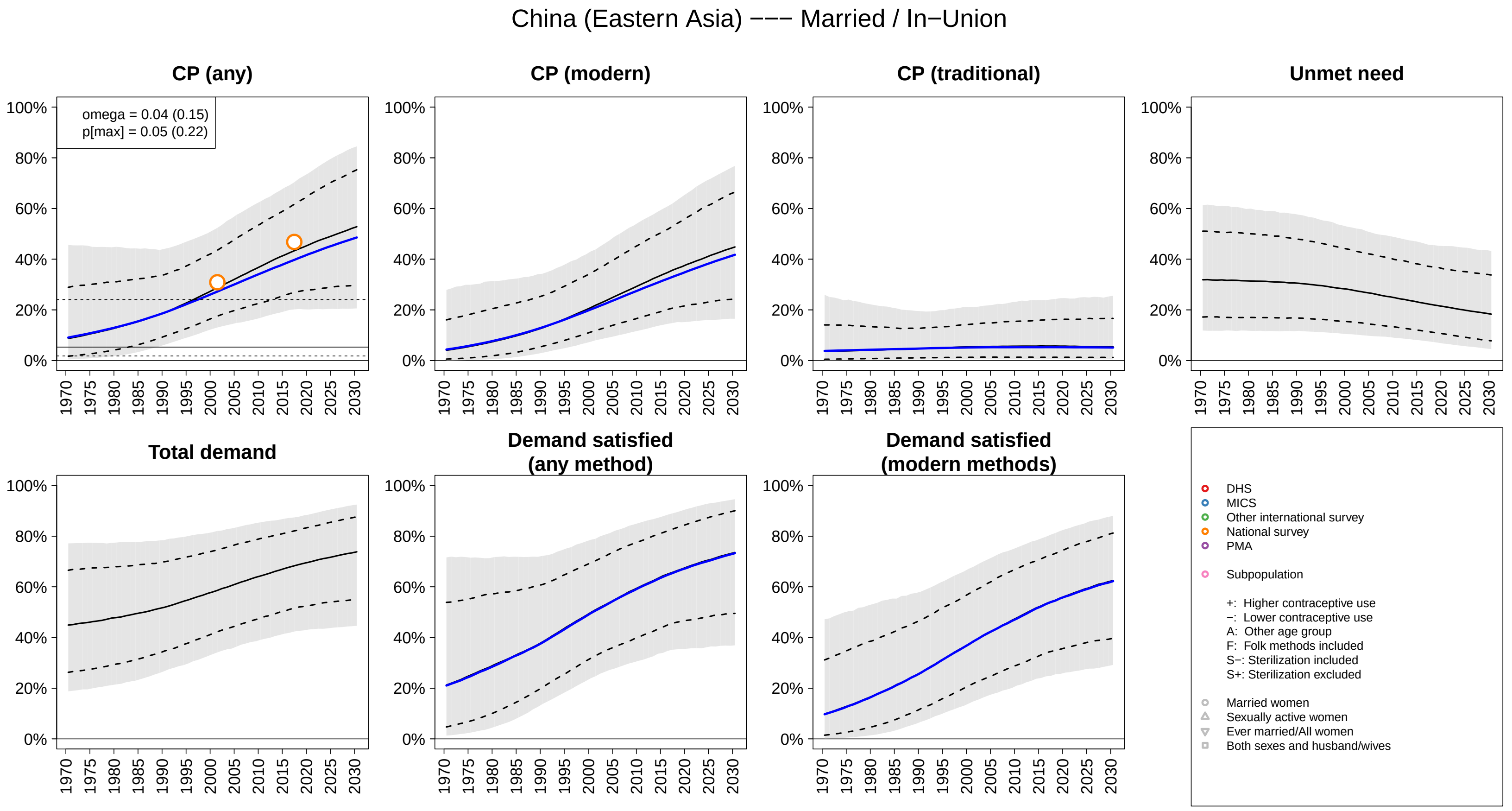
China (Eastern Asia) ––– Married / In−Union
CP (any)
omega = 0.04 (0.15)
p[max] = 0.05 (0.22)
CP (modern)
CP (traditional)
Unmet need
Total demand
Demand satisfied (any method)
Demand satisfied (modern methods)
DHS
MICS
Other international survey
National survey
PMA
Subpopulation
+: Higher contraceptive use
−: Lower contraceptive use
A: Other age group
F: Folk methods included
S−: Sterilization included
S+: Sterilization excluded
Married women
Sexually active women
Ever married/All women
Both sexes and husband/wives

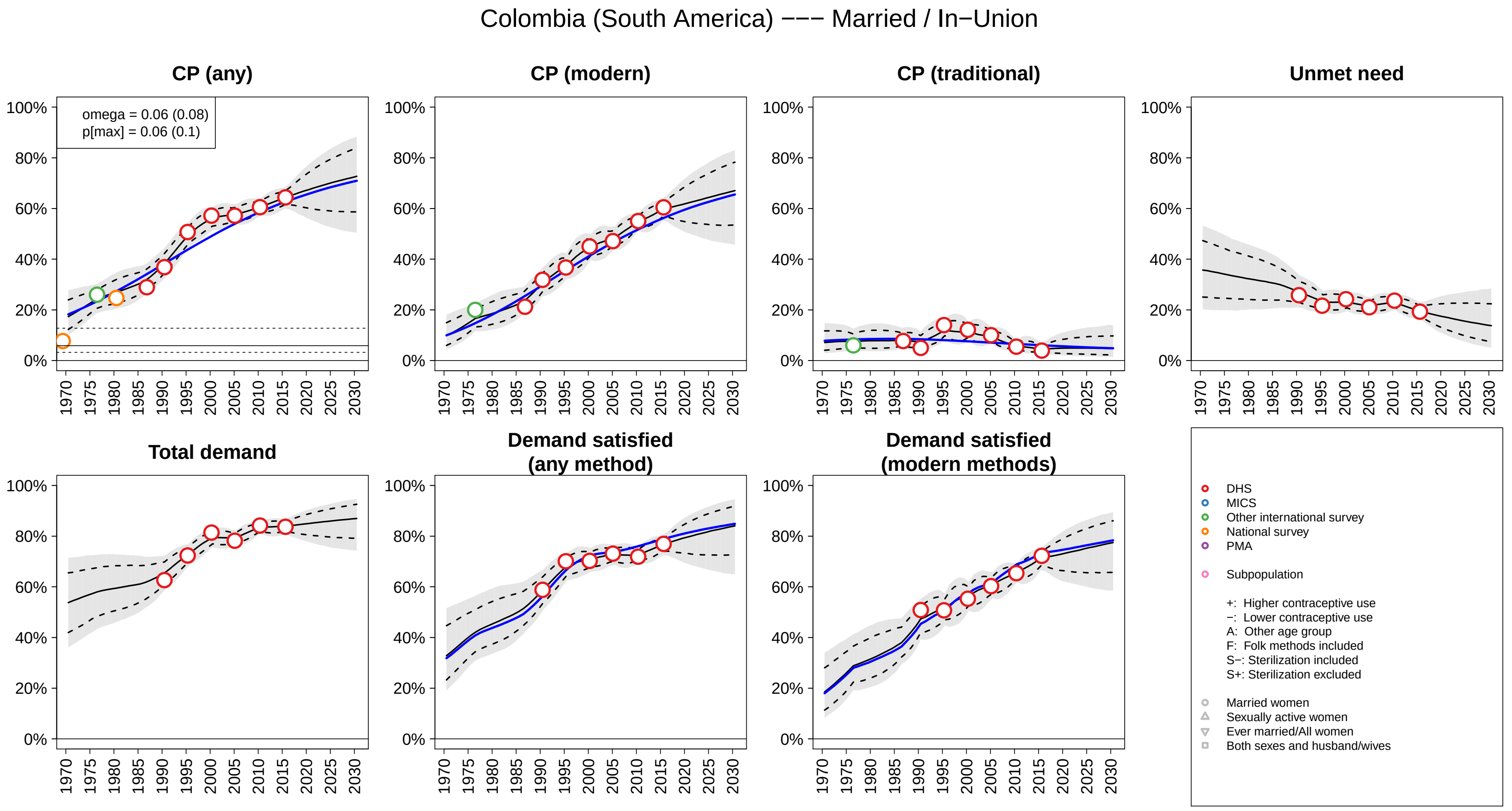
Colombia (South America) ––– Married / In–Union
CP (any)
omega = 0.06 (0.08)
p[max] = 0.06 (0.1)
CP (modern)
CP (traditional)
Unmet need
Total demand
Demand satisfied (any method)
Demand satisfied (modern methods)
DHS
MICS
Other international survey
National survey
PMA
Subpopulation
+: Higher contraceptive use
−: Lower contraceptive use
A: Other age group
F: Folk methods included
S−: Sterilization included
S+: Sterilization excluded
Married women
Sexually active women
Ever married/All women
Both sexes and husband/wives

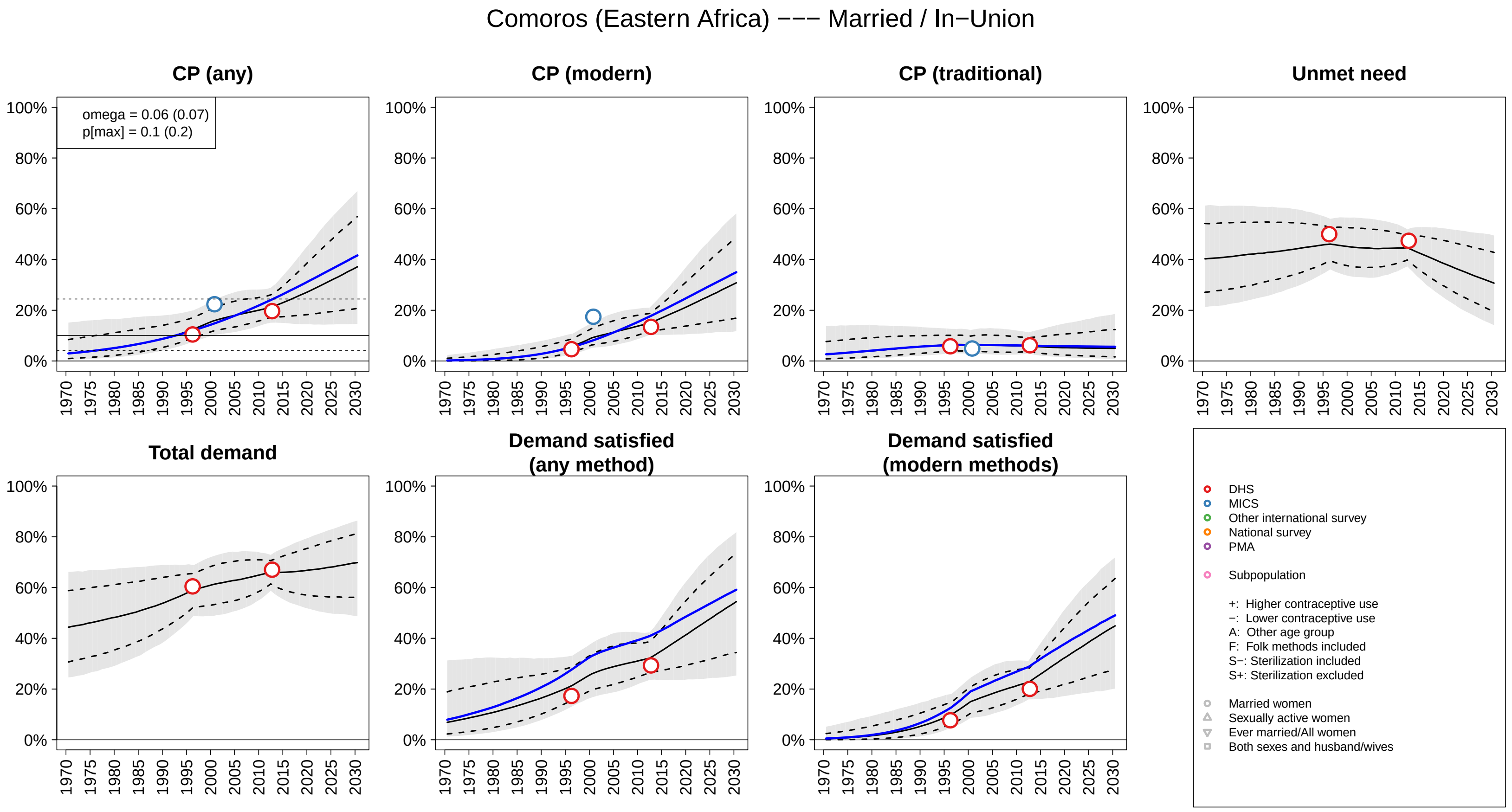
Comoros (Eastern Africa) --- Married / In−Union
CP (any)
omega = 0.06 (0.07)
p[max] = 0.1 (0.2)
CP (modern)
CP (traditional)
Unmet need
Total demand
Demand satisfied (any method)
Demand satisfied (modern methods)
DHS
MICS
Other international survey
National survey
PMA
Subpopulation
+: Higher contraceptive use
−: Lower contraceptive use
A: Other age group
F: Folk methods included
S−: Sterilization included
S+: Sterilization excluded
Married women
Sexually active women
Ever married/All women
Both sexes and husband/wives

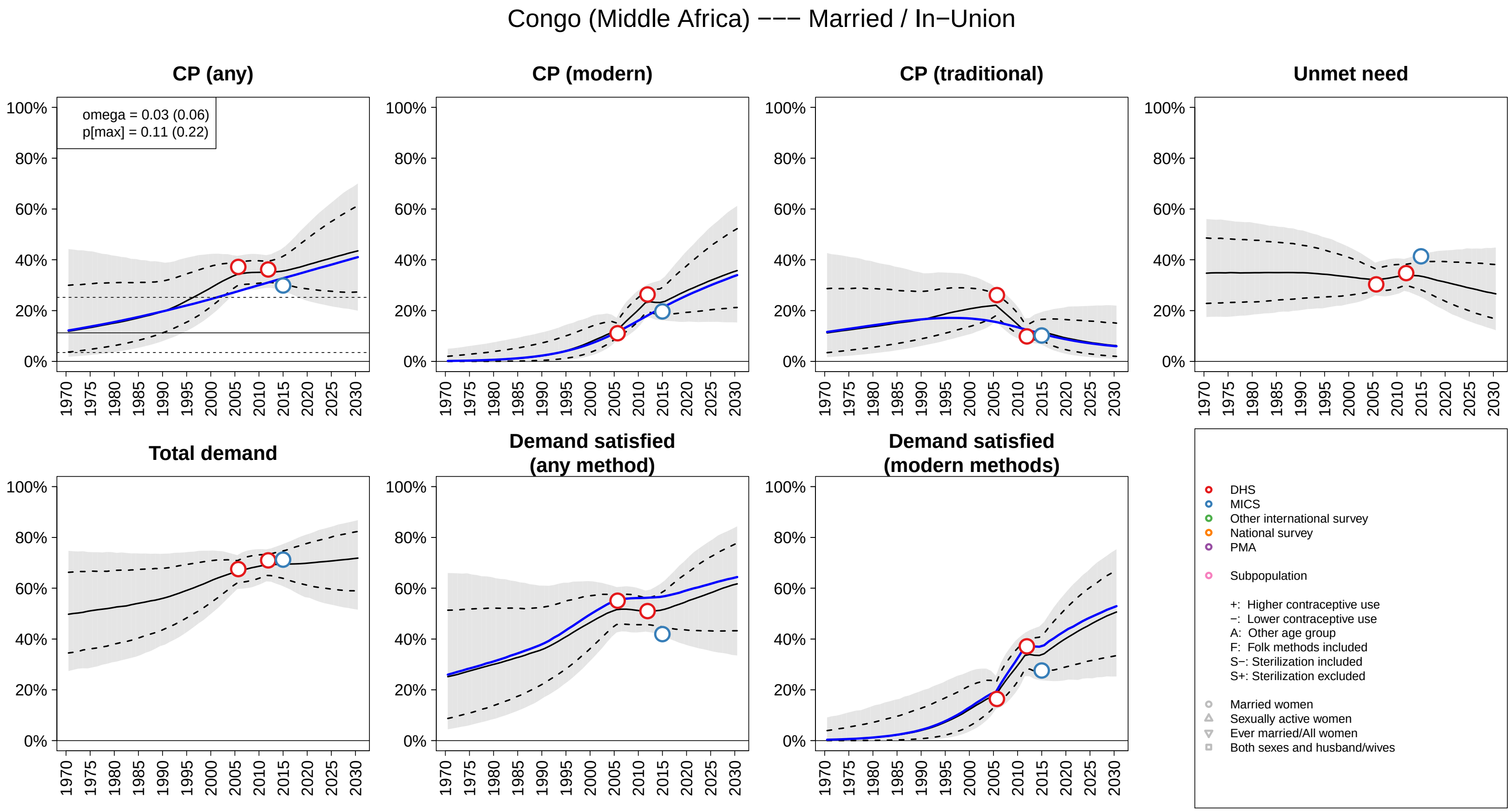
Congo (Middle Africa) −−− Married / In−Union
CP (any)
omega = 0.03 (0.06)
p[max] = 0.11 (0.22)
CP (modern)
CP (traditional)
Unmet need
Total demand
Demand satisfied (any method)
Demand satisfied (modern methods)
DHS
MICS
Other international survey
National survey
PMA
Subpopulation
+: Higher contraceptive use
−: Lower contraceptive use
A: Other age group
F: Folk methods included
S−: Sterilization included
S+: Sterilization excluded
Married women
Sexually active women
Ever married/All women
Both sexes and husband/wives

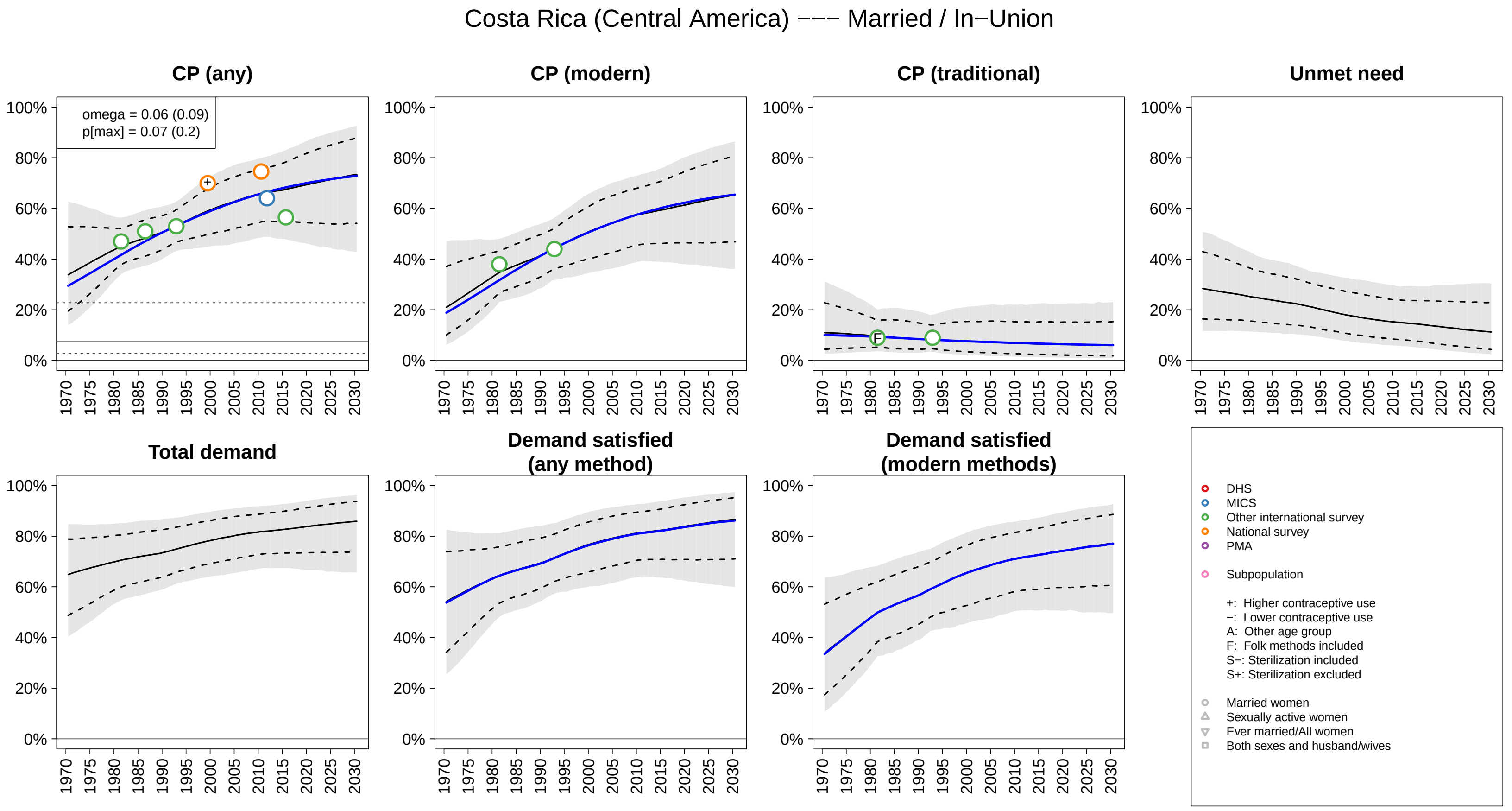
Costa Rica (Central America) --- Married / In−Union
CP (any)
omega = 0.06 (0.09)
p[max] = 0.07 (0.2)
CP (modern)
CP (traditional)
Unmet need
Total demand
Demand satisfied (any method)
Demand satisfied (modern methods)
DHS
MICS
Other international survey
National survey
PMA
Subpopulation
+: Higher contraceptive use
−: Lower contraceptive use
A: Other age group
F: Folk methods included
S−: Sterilization included
S+: Sterilization excluded
Married women
Sexually active women
Ever married/All women
Both sexes and husband/wives

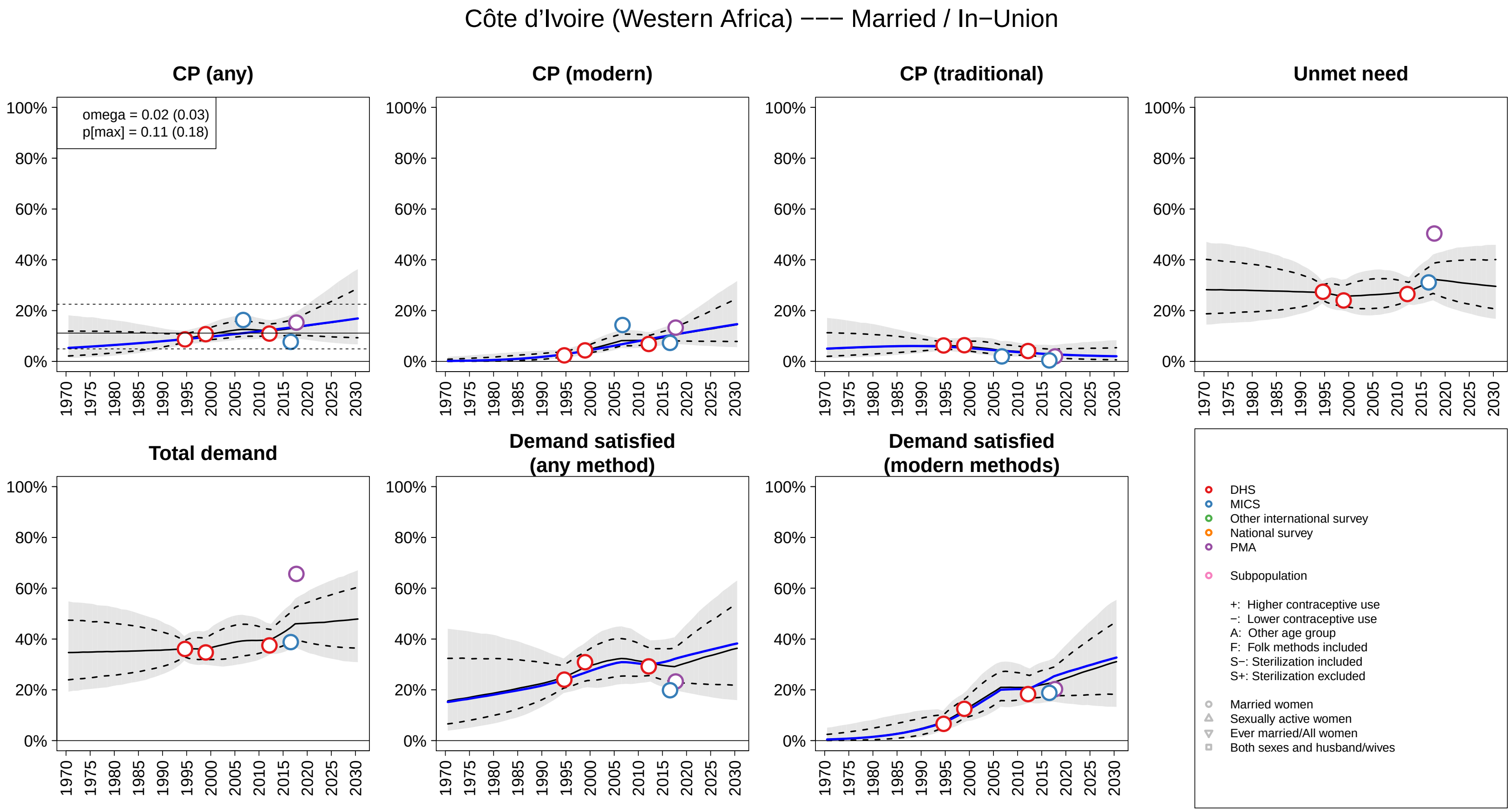
Côte d'Ivoire (Western Africa) --- Married / In−Union
CP (any)
omega = 0.02 (0.03)
p[max] = 0.11 (0.18)
CP (modern)
CP (traditional)
Unmet need
Total demand
Demand satisfied (any method)
Demand satisfied (modern methods)
DHS
MICS
Other international survey
National survey
PMA
Subpopulation
+: Higher contraceptive use
−: Lower contraceptive use
A: Other age group
F: Folk methods included
S−: Sterilization included
S+: Sterilization excluded
Married women
Sexually active women
Ever married/All women
Both sexes and husband/wives

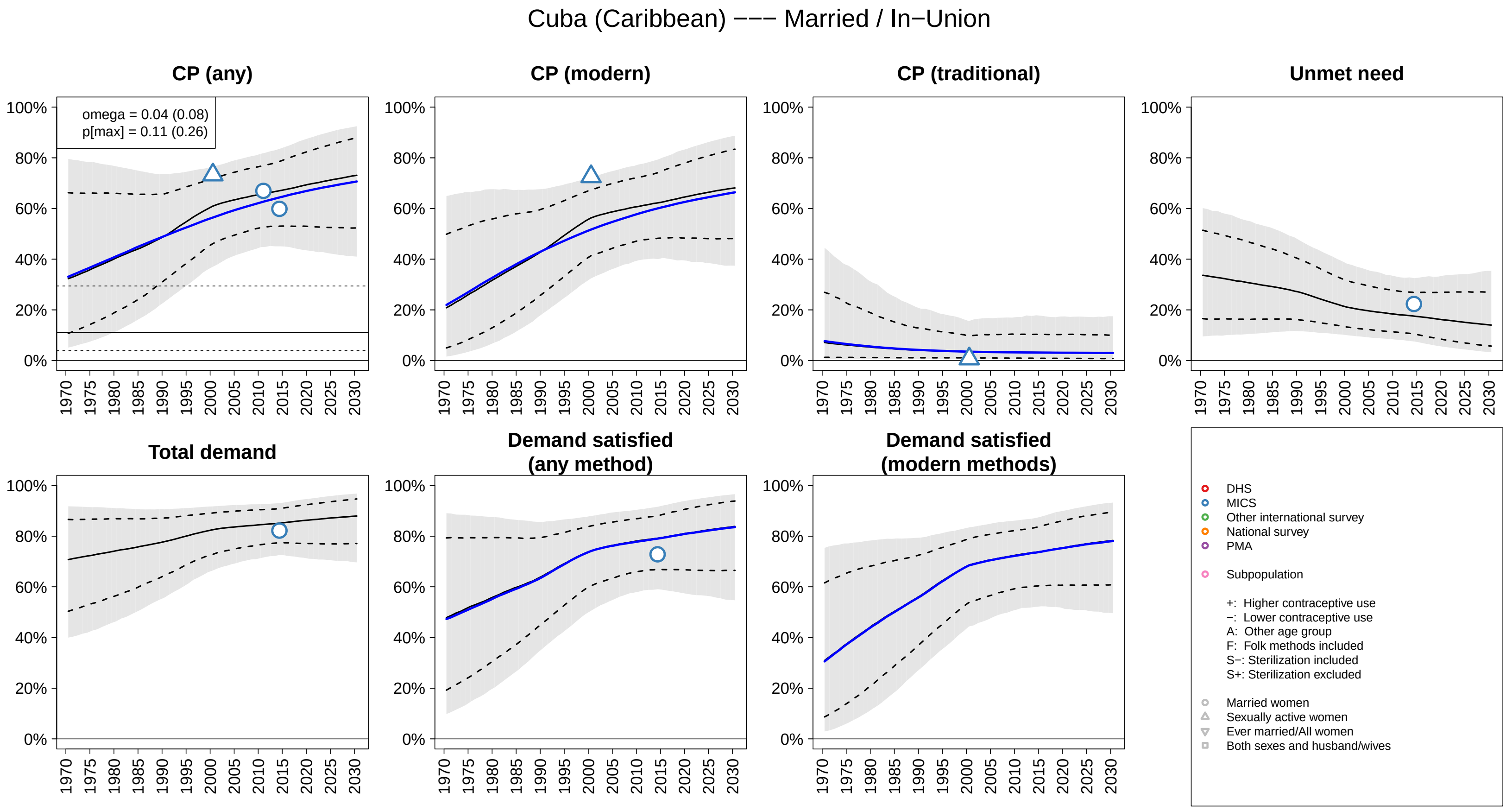
Cuba (Caribbean) --- Married / In−Union
CP (any)
omega = 0.04 (0.08)
p[max] = 0.11 (0.26)
CP (modern)
CP (traditional)
Unmet need
Total demand
Demand satisfied (any method)
Demand satisfied (modern methods)
0%
20%
40%
60%
80%
100%
1970 1975 1980 1985 1990 1995 2000 2005 2010 2015 2020 2025 2030
DHS
MICS
Other international survey
National survey
PMA
Subpopulation
+: Higher contraceptive use
−: Lower contraceptive use
A: Other age group
F: Folk methods included
S−: Sterilization included
S+: Sterilization excluded
Married women
Sexually active women
Ever married/All women
Both sexes and husband/wives

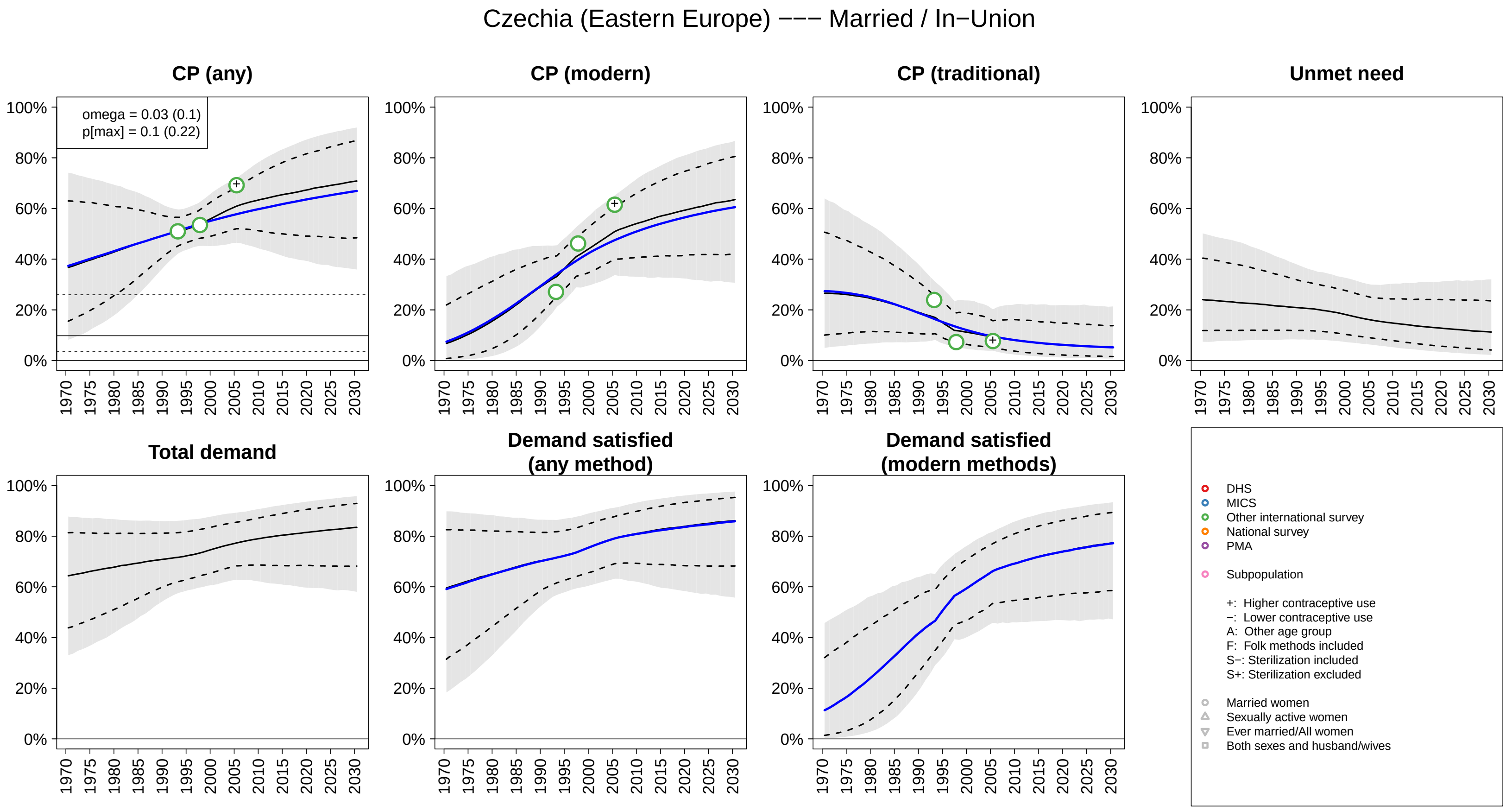
Czechia (Eastern Europe) ––– Married / In−Union
CP (any)
omega = 0.03 (0.1)
p[max] = 0.1 (0.22)
CP (modern)
CP (traditional)
Unmet need
Total demand
Demand satisfied (any method)
Demand satisfied (modern methods)
0%
20%
40%
60%
80%
100%
1970 1975 1980 1985 1990 1995 2000 2005 2010 2015 2020 2025 2030
DHS
MICS
Other international survey
National survey
PMA
Subpopulation
+: Higher contraceptive use
−: Lower contraceptive use
A: Other age group
F: Folk methods included
S−: Sterilization included
S+: Sterilization excluded
Married women
Sexually active women
Ever married/All women
Both sexes and husband/wives

Democratic Rep. of the Congo (Middle Africa) --- Married / In−Union

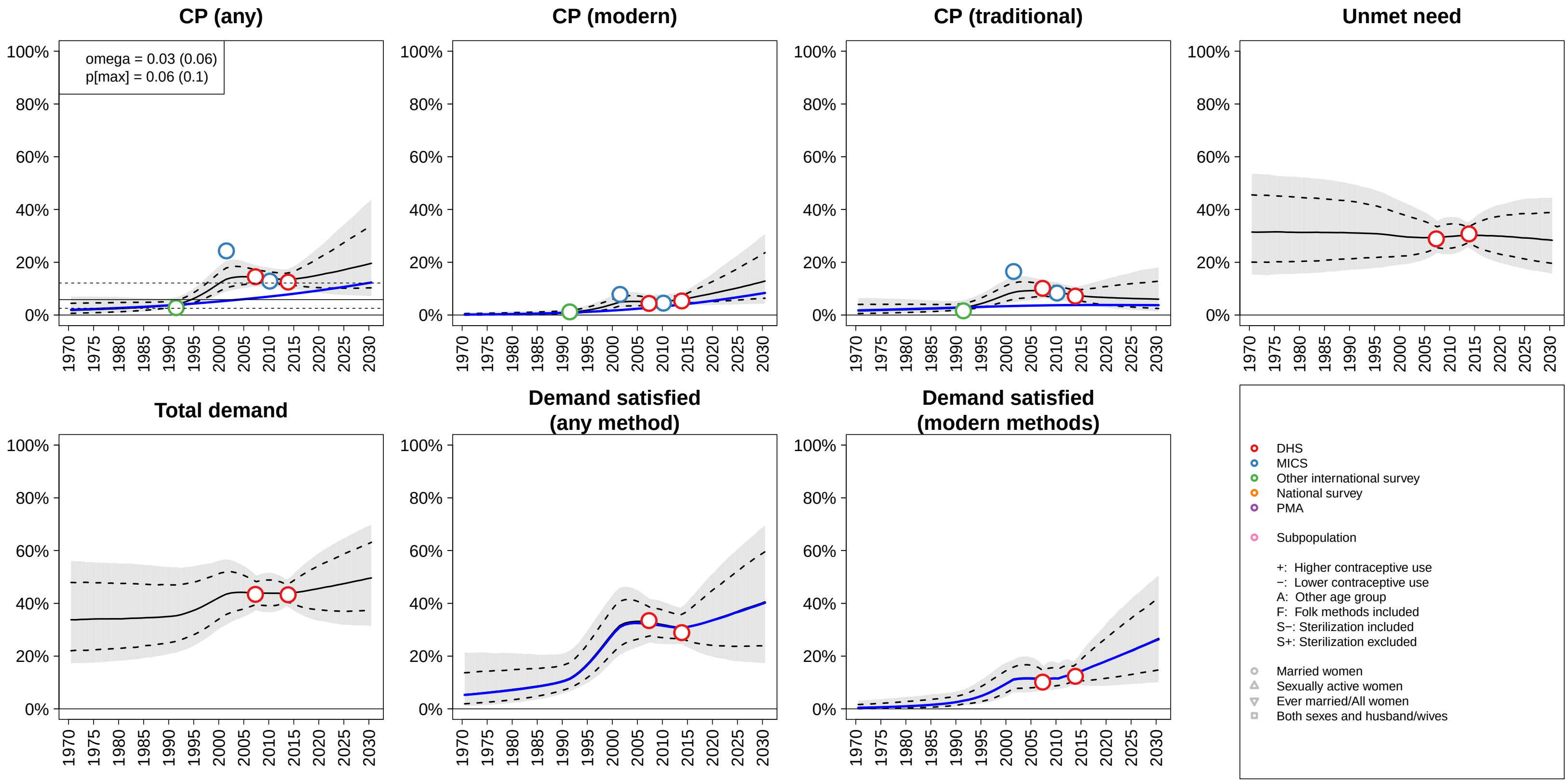

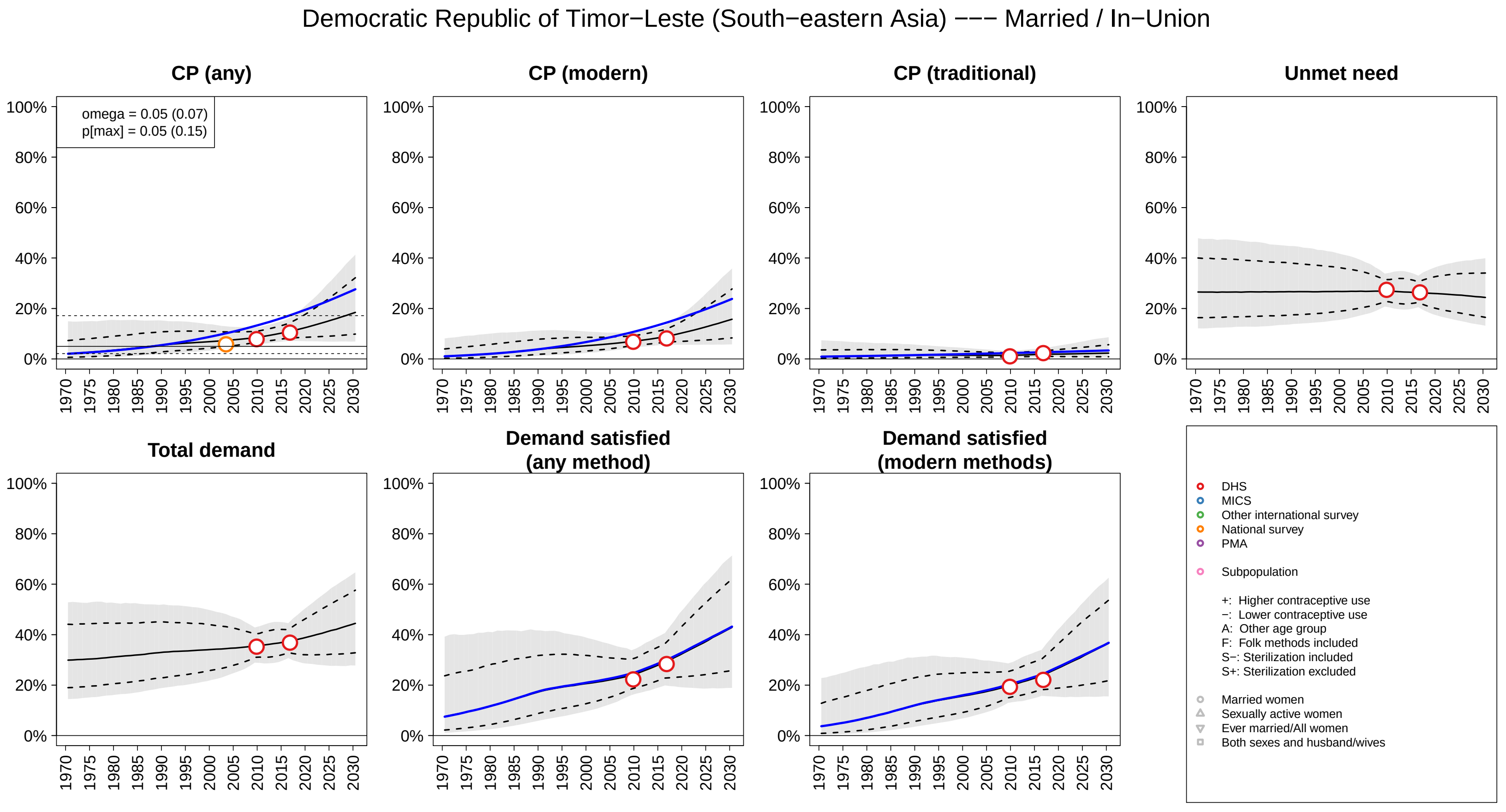
Democratic Republic of Timor−Leste (South−eastern Asia) −−− Married / In−Union
CP (any)
omega = 0.05 (0.07)
p[max] = 0.05 (0.15)
CP (modern)
CP (traditional)
Unmet need
Total demand
Demand satisfied (any method)
Demand satisfied (modern methods)
DHS
MICS
Other international survey
National survey
PMA
Subpopulation
+: Higher contraceptive use
−: Lower contraceptive use
A: Other age group
F: Folk methods included
S−: Sterilization included
S+: Sterilization excluded
Married women
Sexually active women
Ever married/All women
Both sexes and husband/wives

Djibouti (Eastern Africa) −−− Married / In−Union

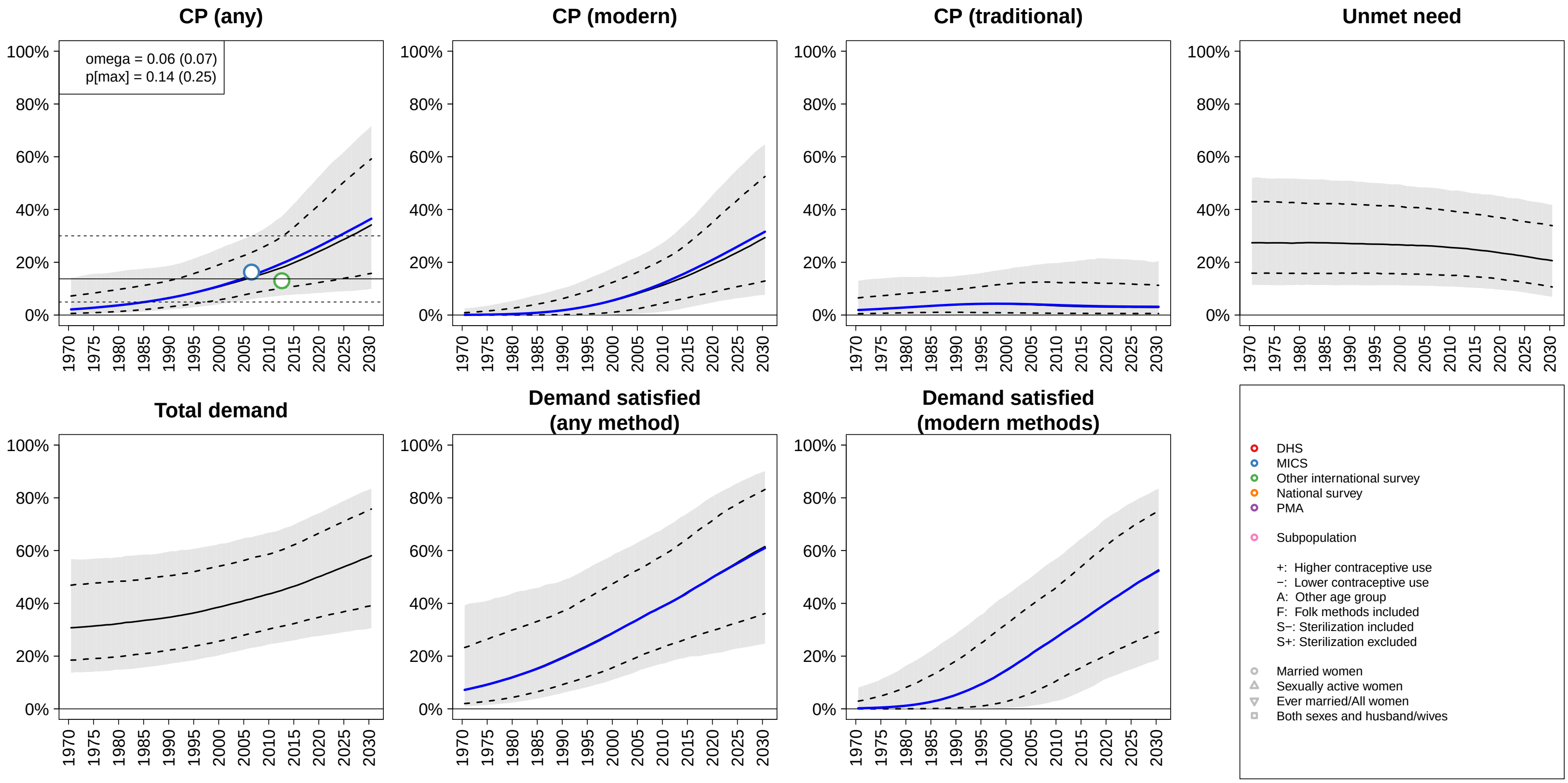

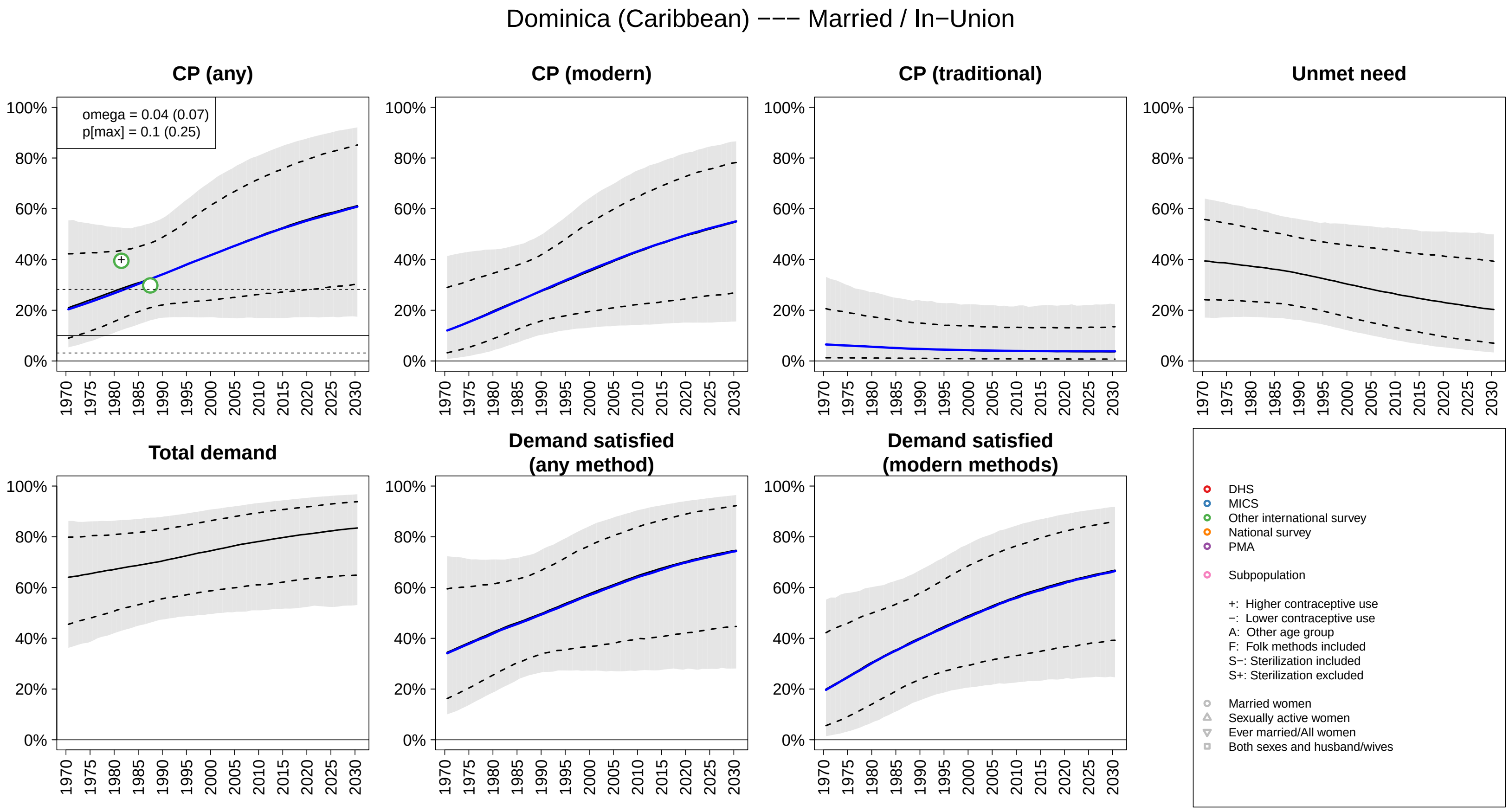
Dominica (Caribbean) −−− Married / In−Union
CP (any)
omega = 0.04 (0.07)
p[max] = 0.1 (0.25)
CP (modern)
CP (traditional)
Unmet need
Total demand
Demand satisfied (any method)
Demand satisfied (modern methods)
DHS
MICS
Other international survey
National survey
PMA
Subpopulation
+: Higher contraceptive use
−: Lower contraceptive use
A: Other age group
F: Folk methods included
S−: Sterilization included
S+: Sterilization excluded
Married women
Sexually active women
Ever married/All women
Both sexes and husband/wives

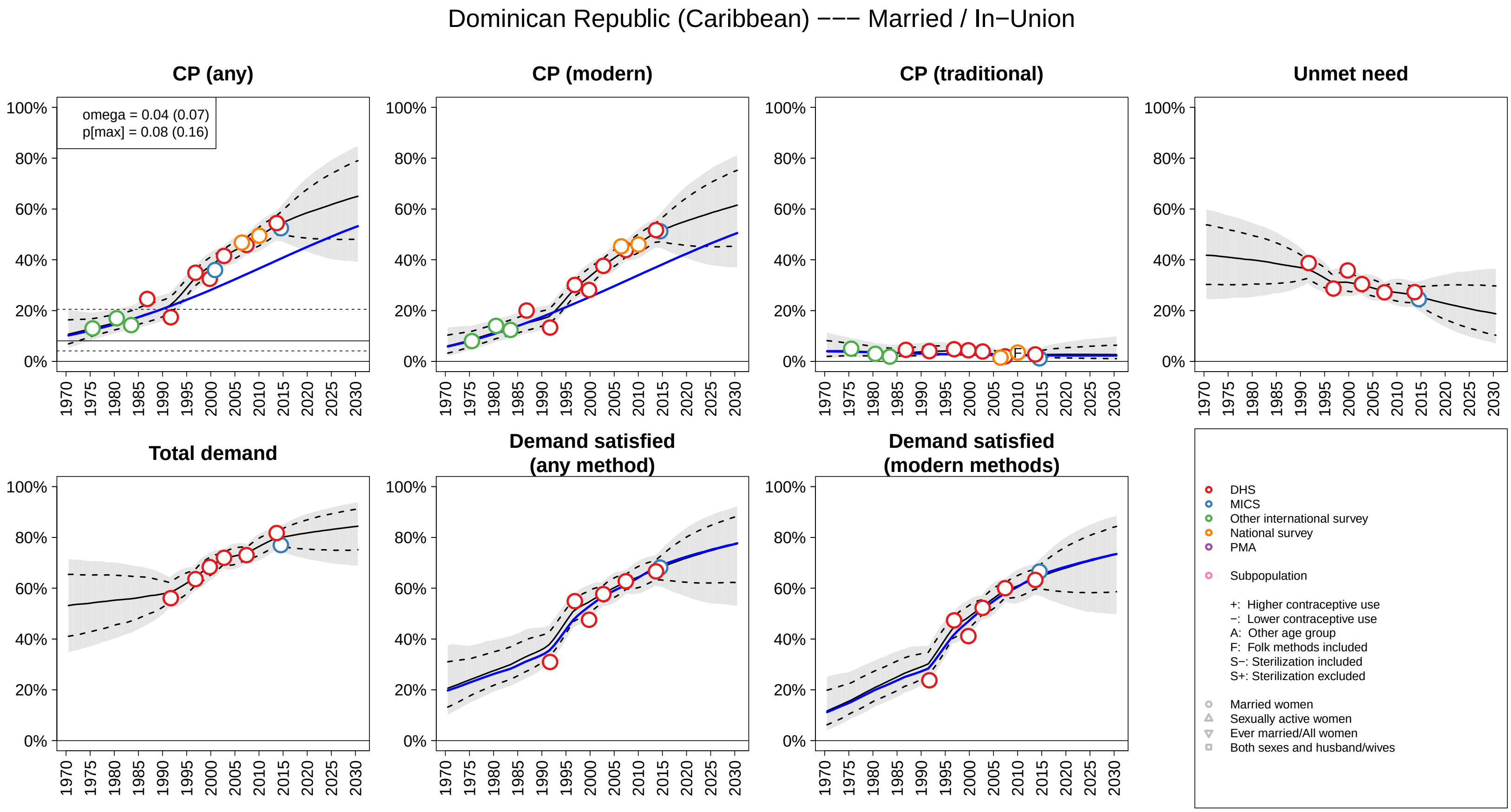

Dominican Republic (Caribbean) --- Married / In−Union
CP (any)
omega = 0.04 (0.07)
p[max] = 0.08 (0.16)
CP (modern)
CP (traditional)
Unmet need
Total demand
Demand satisfied (any method)
Demand satisfied (modern methods)
DHS
MICS
Other international survey
National survey
PMA
Subpopulation
+: Higher contraceptive use
−: Lower contraceptive use
A: Other age group
F: Folk methods included
S−: Sterilization included
S+: Sterilization excluded
Married women
Sexually active women
Ever married/All women
Both sexes and husband/wives

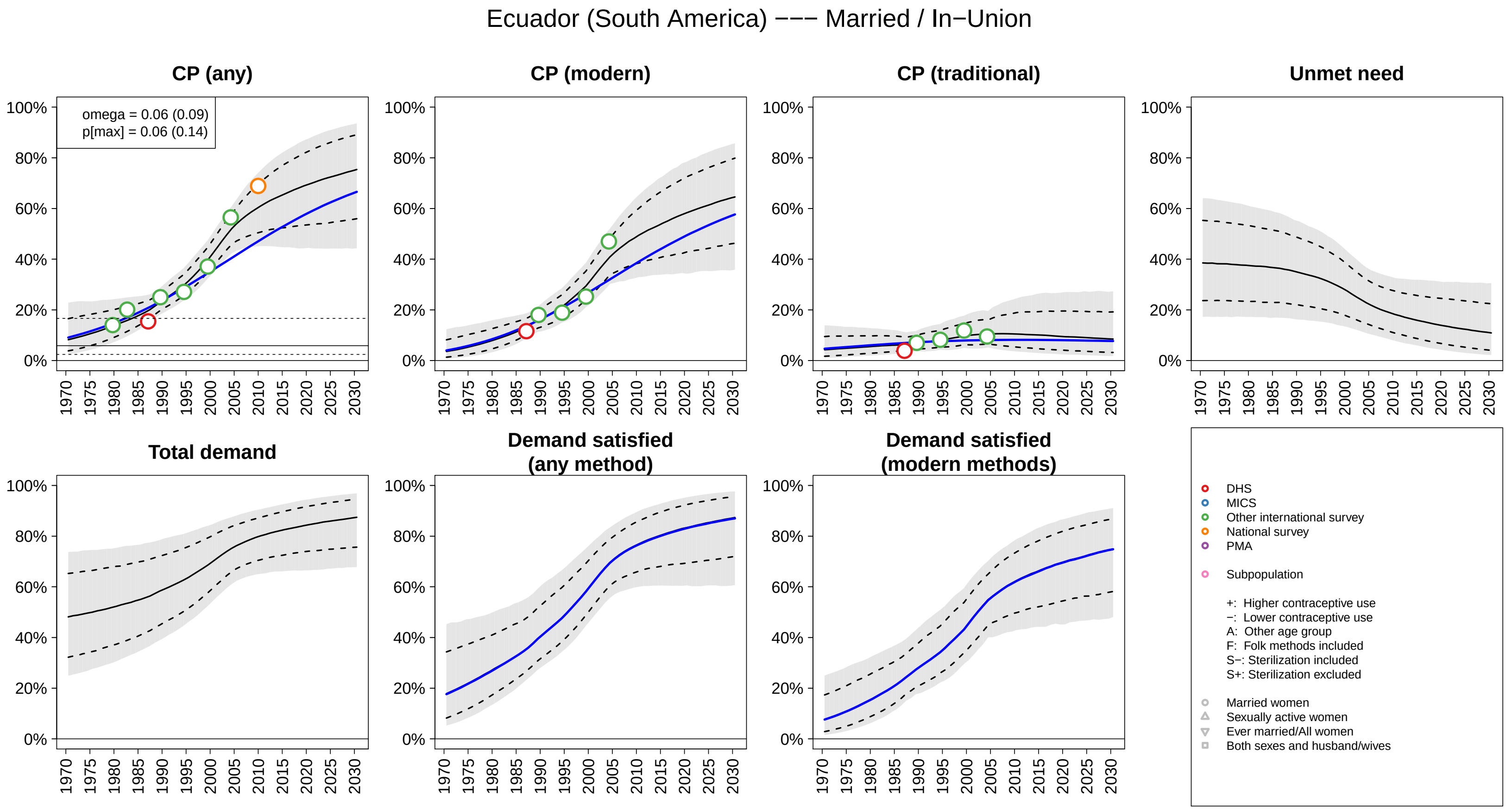

Ecuador (South America) ––– Married / In–Union
CP (any)
omega = 0.06 (0.09)
p[max] = 0.06 (0.14)
CP (modern)
CP (traditional)
Unmet need
Total demand
Demand satisfied (any method)
Demand satisfied (modern methods)
DHS
MICS
Other international survey
National survey
PMA
Subpopulation
+: Higher contraceptive use
−: Lower contraceptive use
A: Other age group
F: Folk methods included
S−: Sterilization included
S+: Sterilization excluded
Married women
Sexually active women
Ever married/All women
Both sexes and husband/wives

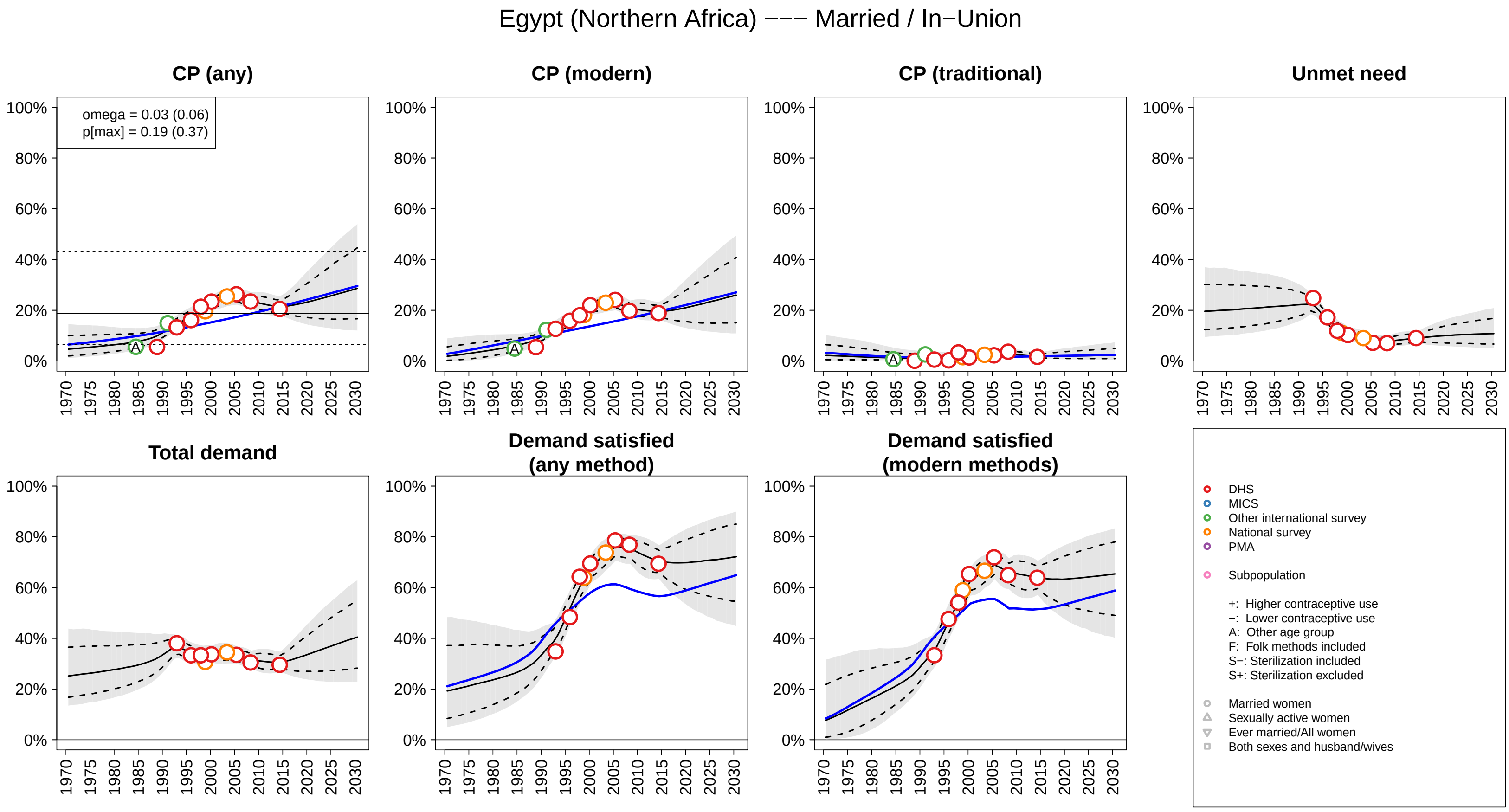
Egypt (Northern Africa) --- Married / In−Union
CP (any)
omega = 0.03 (0.06)
p[max] = 0.19 (0.37)
CP (modern)
CP (traditional)
Unmet need
Total demand
Demand satisfied (any method)
Demand satisfied (modern methods)
DHS
MICS
Other international survey
National survey
PMA
Subpopulation
+: Higher contraceptive use
−: Lower contraceptive use
A: Other age group
F: Folk methods included
S−: Sterilization included
S+: Sterilization excluded
Married women
Sexually active women
Ever married/All women
Both sexes and husband/wives

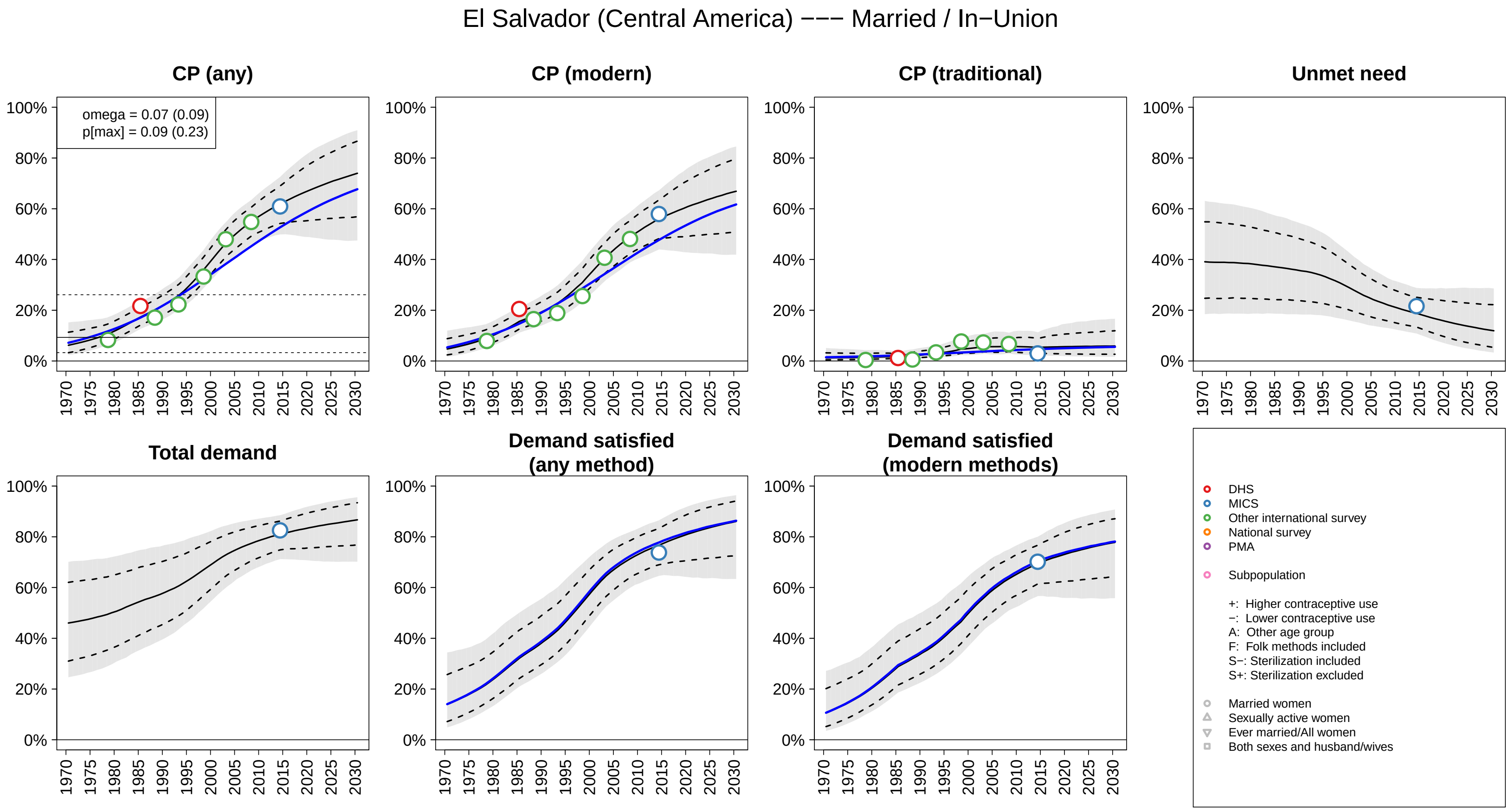
El Salvador (Central America) --- Married / In−Union
CP (any)
omega = 0.07 (0.09)
p[max] = 0.09 (0.23)
CP (modern)
CP (traditional)
Unmet need
Total demand
Demand satisfied (any method)
Demand satisfied (modern methods)
DHS
MICS
Other international survey
National survey
PMA
Subpopulation
+: Higher contraceptive use
−: Lower contraceptive use
A: Other age group
F: Folk methods included
S−: Sterilization included
S+: Sterilization excluded
Married women
Sexually active women
Ever married/All women
Both sexes and husband/wives

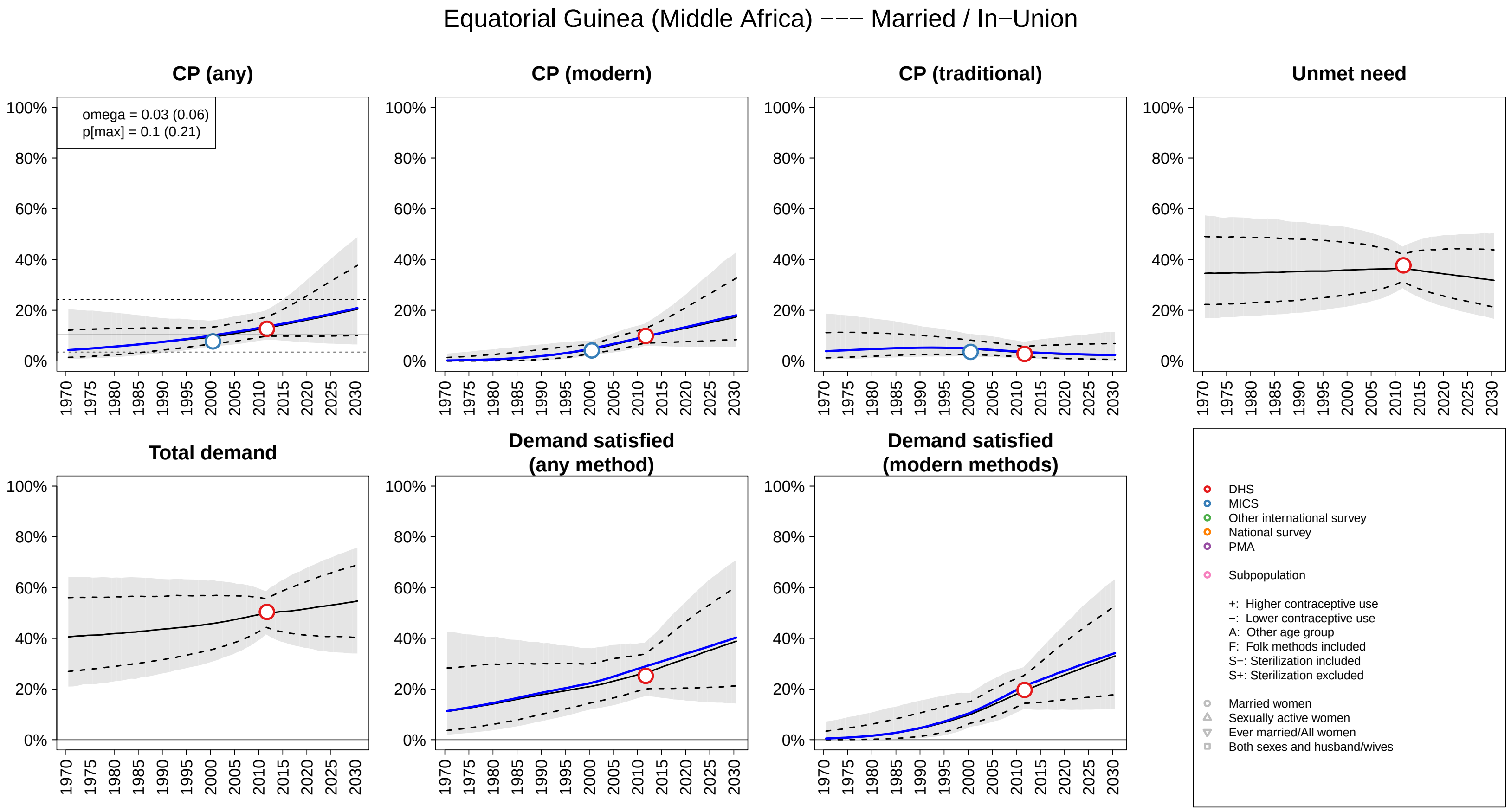
Equatorial Guinea (Middle Africa) --- Married / In−Union
CP (any)
omega = 0.03 (0.06)
p[max] = 0.1 (0.21)
CP (modern)
CP (traditional)
Unmet need
Total demand
Demand satisfied (any method)
Demand satisfied (modern methods)
DHS
MICS
Other international survey
National survey
PMA
Subpopulation
+: Higher contraceptive use
−: Lower contraceptive use
A: Other age group
F: Folk methods included
S−: Sterilization included
S+: Sterilization excluded
Married women
Sexually active women
Ever married/All women
Both sexes and husband/wives

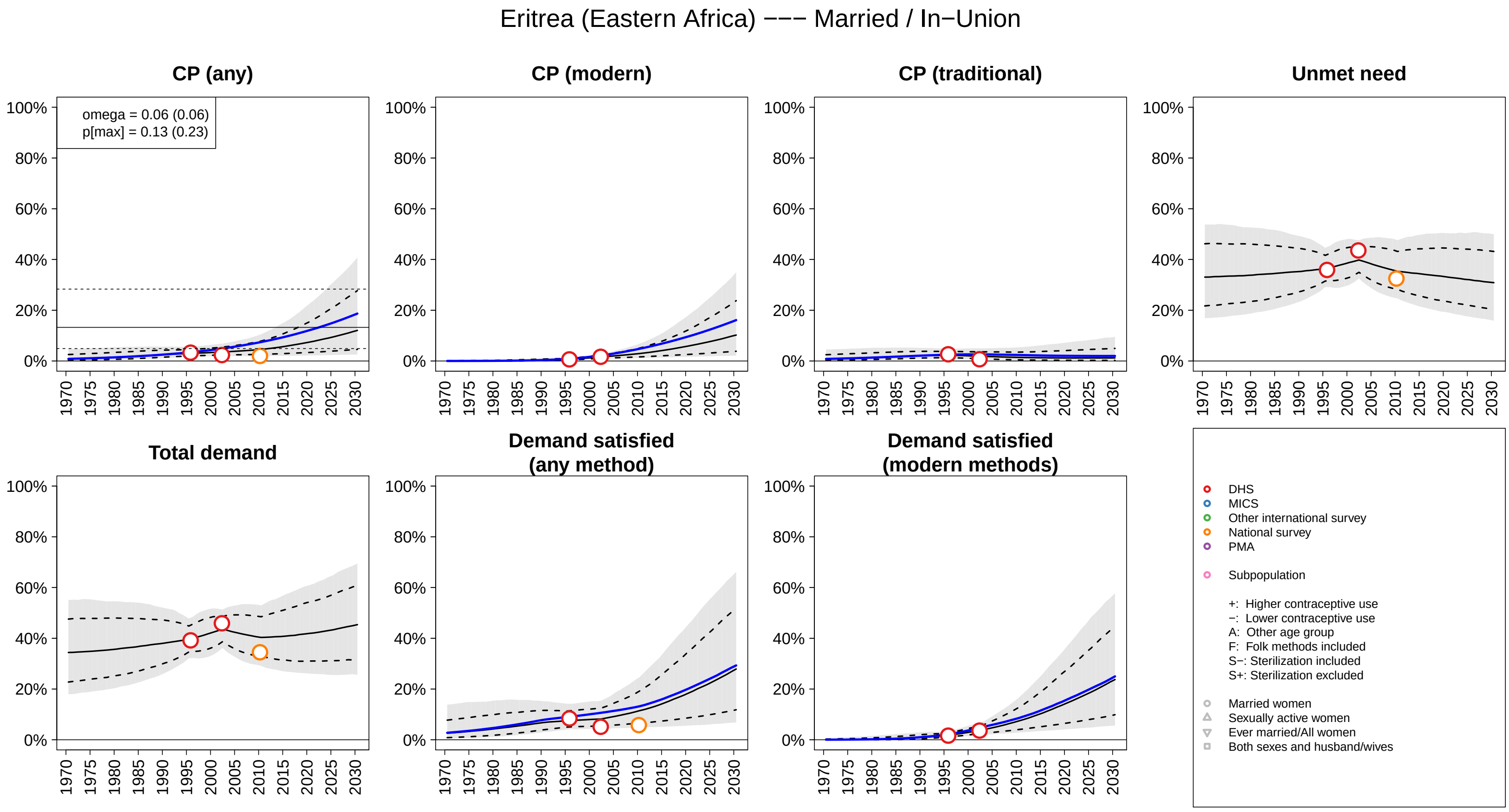
Eritrea (Eastern Africa) −−− Married / In−Union
CP (any)
omega = 0.06 (0.06)
p[max] = 0.13 (0.23)
CP (modern)
CP (traditional)
Unmet need
Total demand
Demand satisfied (any method)
Demand satisfied (modern methods)
DHS
MICS
Other international survey
National survey
PMA
Subpopulation
+: Higher contraceptive use
−: Lower contraceptive use
A: Other age group
F: Folk methods included
S−: Sterilization included
S+: Sterilization excluded
Married women
Sexually active women
Ever married/All women
Both sexes and husband/wives

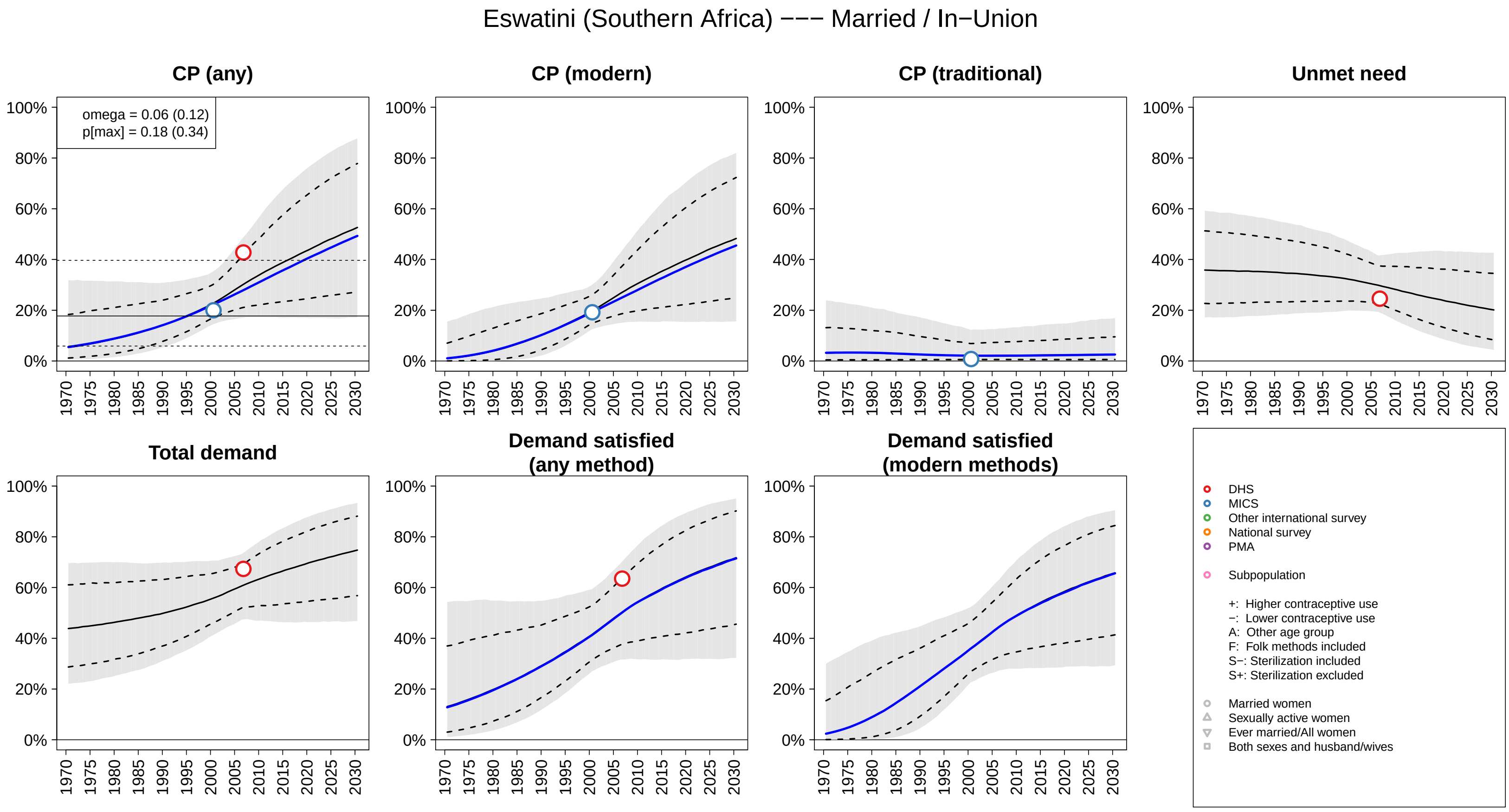
Eswatini (Southern Africa) --- Married / In−Union
CP (any)
omega = 0.06 (0.12)
p[max] = 0.18 (0.34)
CP (modern)
CP (traditional)
Unmet need
Total demand
Demand satisfied (any method)
Demand satisfied (modern methods)
0%
20%
40%
60%
80%
100%
1970
1975
1980
1985
1990
1995
2000
2005
2010
2015
2020
2025
2030
DHS
MICS
Other international survey
National survey
PMA
Subpopulation
+: Higher contraceptive use
−: Lower contraceptive use
A: Other age group
F: Folk methods included
S−: Sterilization included
S+: Sterilization excluded
Married women
Sexually active women
Ever married/All women
Both sexes and husband/wives

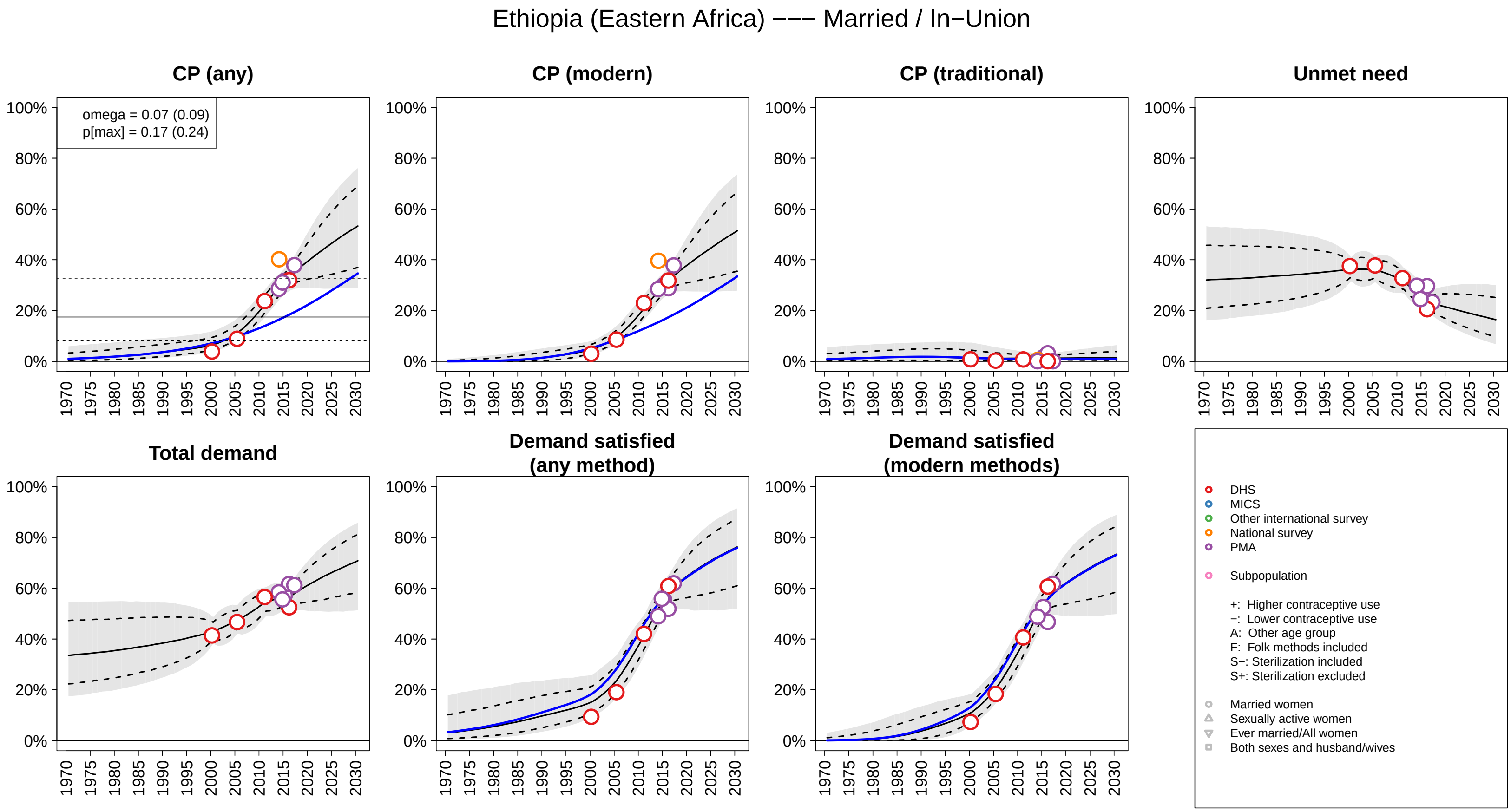
Ethiopia (Eastern Africa) ––– Married / In–Union
CP (any)
omega = 0.07 (0.09)
p[max] = 0.17 (0.24)
CP (modern)
CP (traditional)
Unmet need
Total demand
Demand satisfied (any method)
Demand satisfied (modern methods)
0%
20%
40%
60%
80%
100%
1970
1975
1980
1985
1990
1995
2000
2005
2010
2015
2020
2025
2030
DHS
MICS
Other international survey
National survey
PMA
Subpopulation
+: Higher contraceptive use
−: Lower contraceptive use
A: Other age group
F: Folk methods included
S−: Sterilization included
S+: Sterilization excluded
Married women
Sexually active women
Ever married/All women
Both sexes and husband/wives

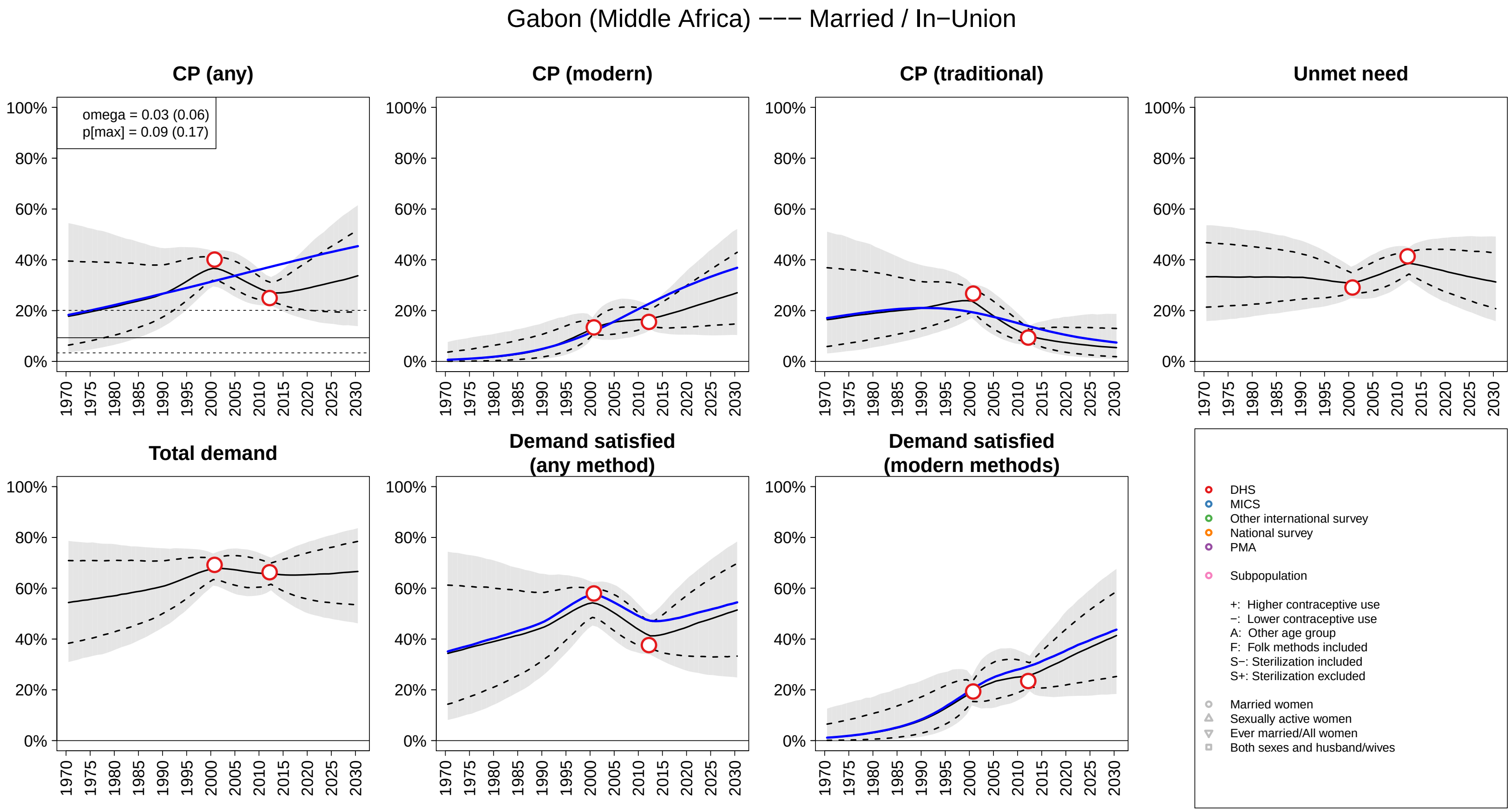
Gabon (Middle Africa) ––– Married / In−Union
CP (any)
omega = 0.03 (0.06)
p[max] = 0.09 (0.17)
CP (modern)
CP (traditional)
Unmet need
Total demand
Demand satisfied (any method)
Demand satisfied (modern methods)
DHS
MICS
Other international survey
National survey
PMA
Subpopulation
+: Higher contraceptive use
−: Lower contraceptive use
A: Other age group
F: Folk methods included
S−: Sterilization included
S+: Sterilization excluded
Married women
Sexually active women
Ever married/All women
Both sexes and husband/wives

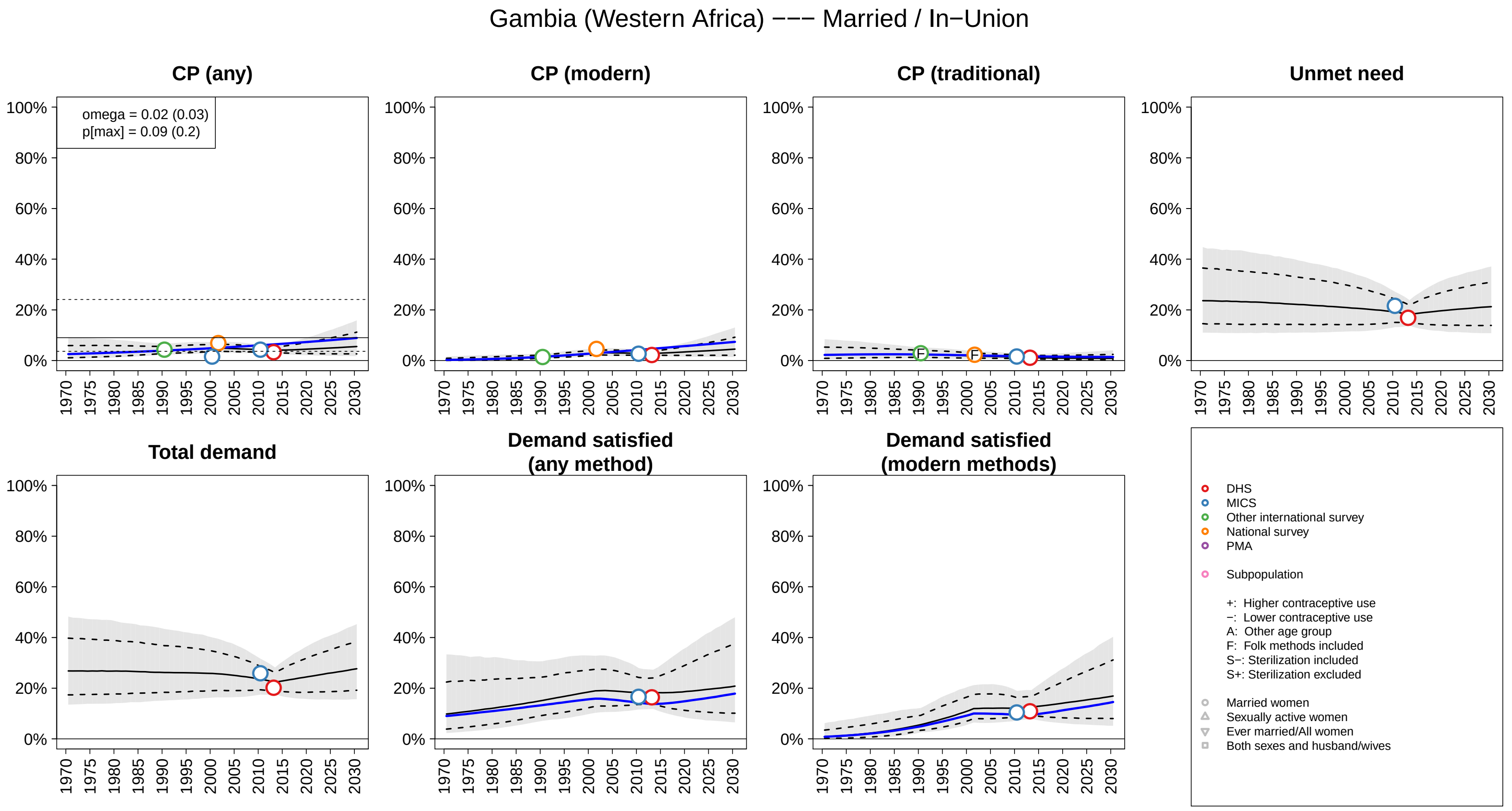

Gambia (Western Africa) --- Married / In-Union
CP (any)
omega = 0.02 (0.03)
p[max] = 0.09 (0.2)
CP (modern)
CP (traditional)
Unmet need
Total demand
Demand satisfied (any method)
Demand satisfied (modern methods)
DHS
MICS
Other international survey
National survey
PMA
Subpopulation
+: Higher contraceptive use
−: Lower contraceptive use
A: Other age group
F: Folk methods included
S−: Sterilization included
S+: Sterilization excluded
Married women
Sexually active women
Ever married/All women
Both sexes and husband/wives

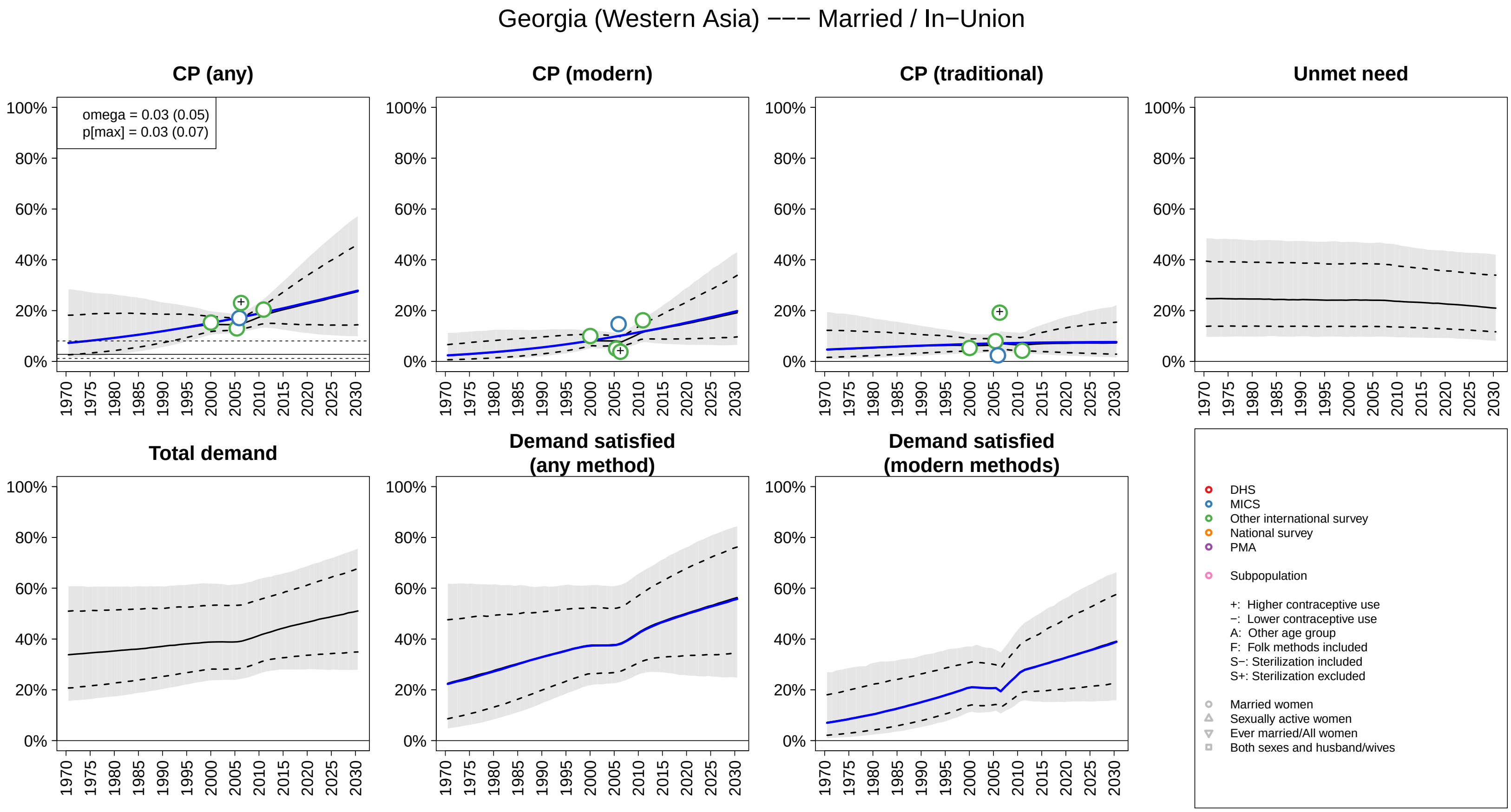
Georgia (Western Asia) --- Married / In−Union
CP (any)
omega = 0.03 (0.05)
p[max] = 0.03 (0.07)
CP (modern)
CP (traditional)
Unmet need
Total demand
Demand satisfied (any method)
Demand satisfied (modern methods)
DHS
MICS
Other international survey
National survey
PMA
Subpopulation
+: Higher contraceptive use
−: Lower contraceptive use
A: Other age group
F: Folk methods included
S−: Sterilization included
S+: Sterilization excluded
Married women
Sexually active women
Ever married/All women
Both sexes and husband/wives

Ghana (Western Africa) −−− Married / In−Union

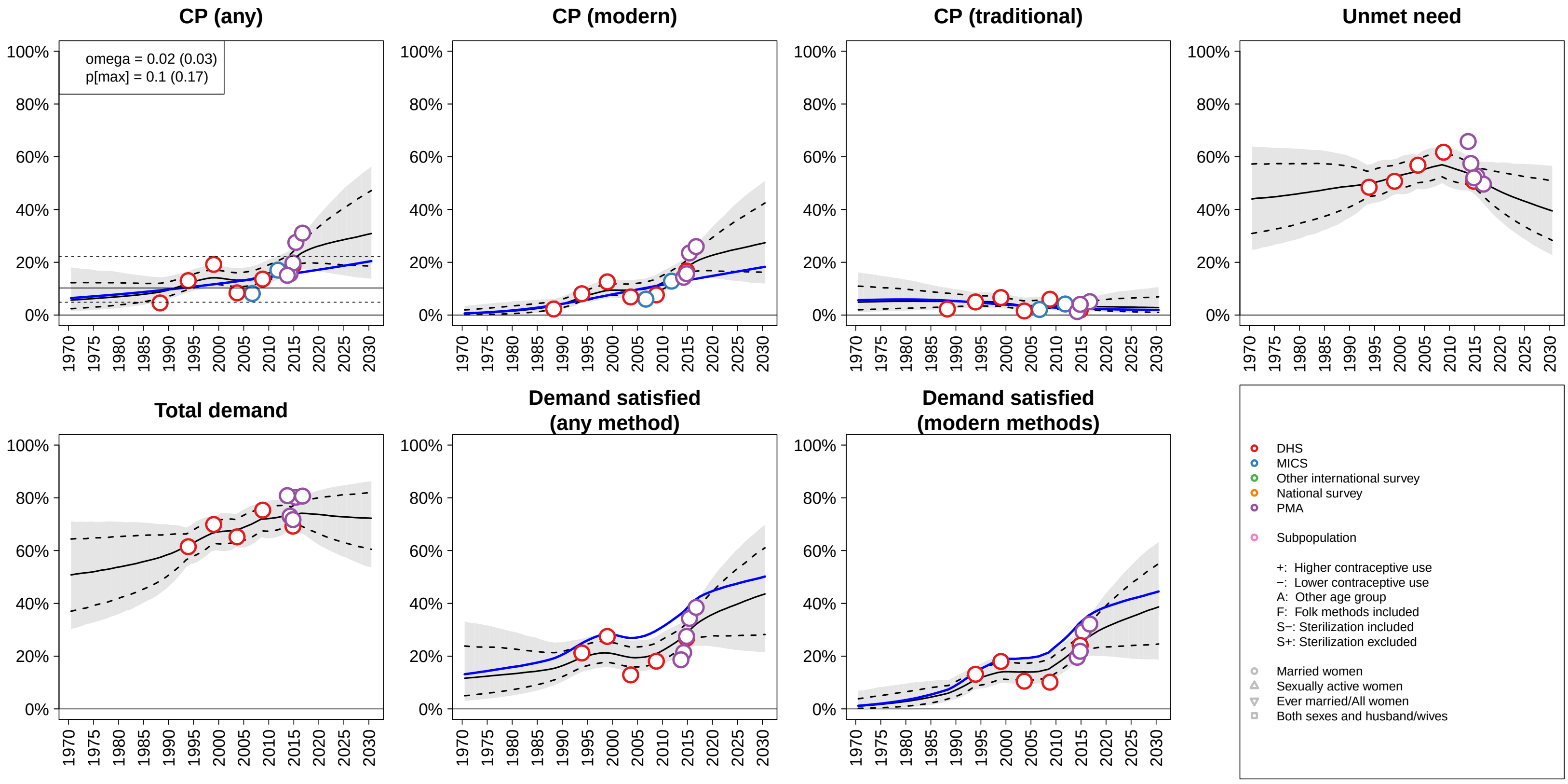

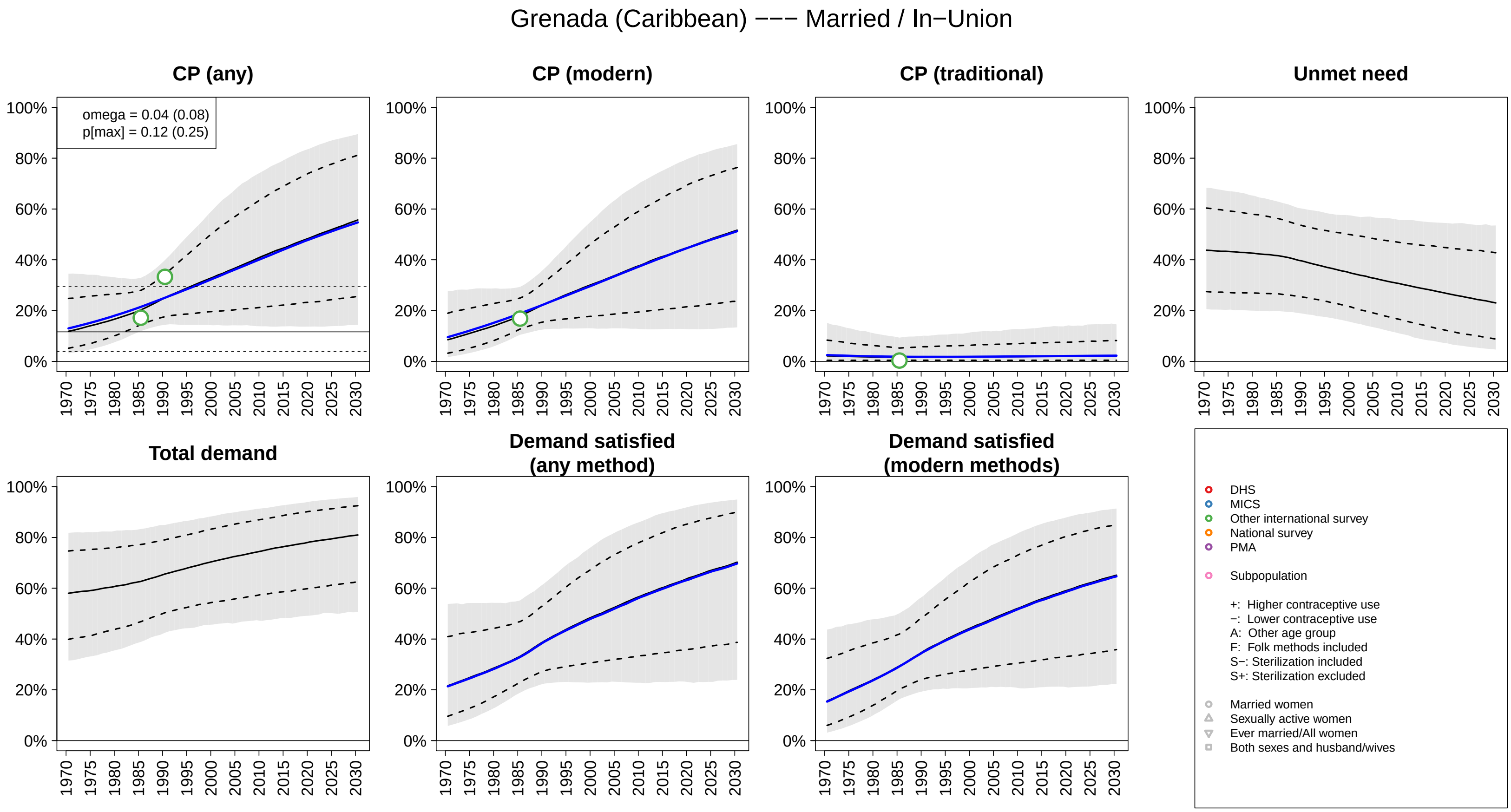
Grenada (Caribbean) ––– Married / In−Union
CP (any)
omega = 0.04 (0.08)
p[max] = 0.12 (0.25)
CP (modern)
CP (traditional)
Unmet need
Total demand
Demand satisfied (any method)
Demand satisfied (modern methods)
0%
20%
40%
60%
80%
100%
1970
1975
1980
1985
1990
1995
2000
2005
2010
2015
2020
2025
2030
DHS
MICS
Other international survey
National survey
PMA
Subpopulation
+: Higher contraceptive use
−: Lower contraceptive use
A: Other age group
F: Folk methods included
S−: Sterilization included
S+: Sterilization excluded
Married women
Sexually active women
Ever married/All women
Both sexes and husband/wives

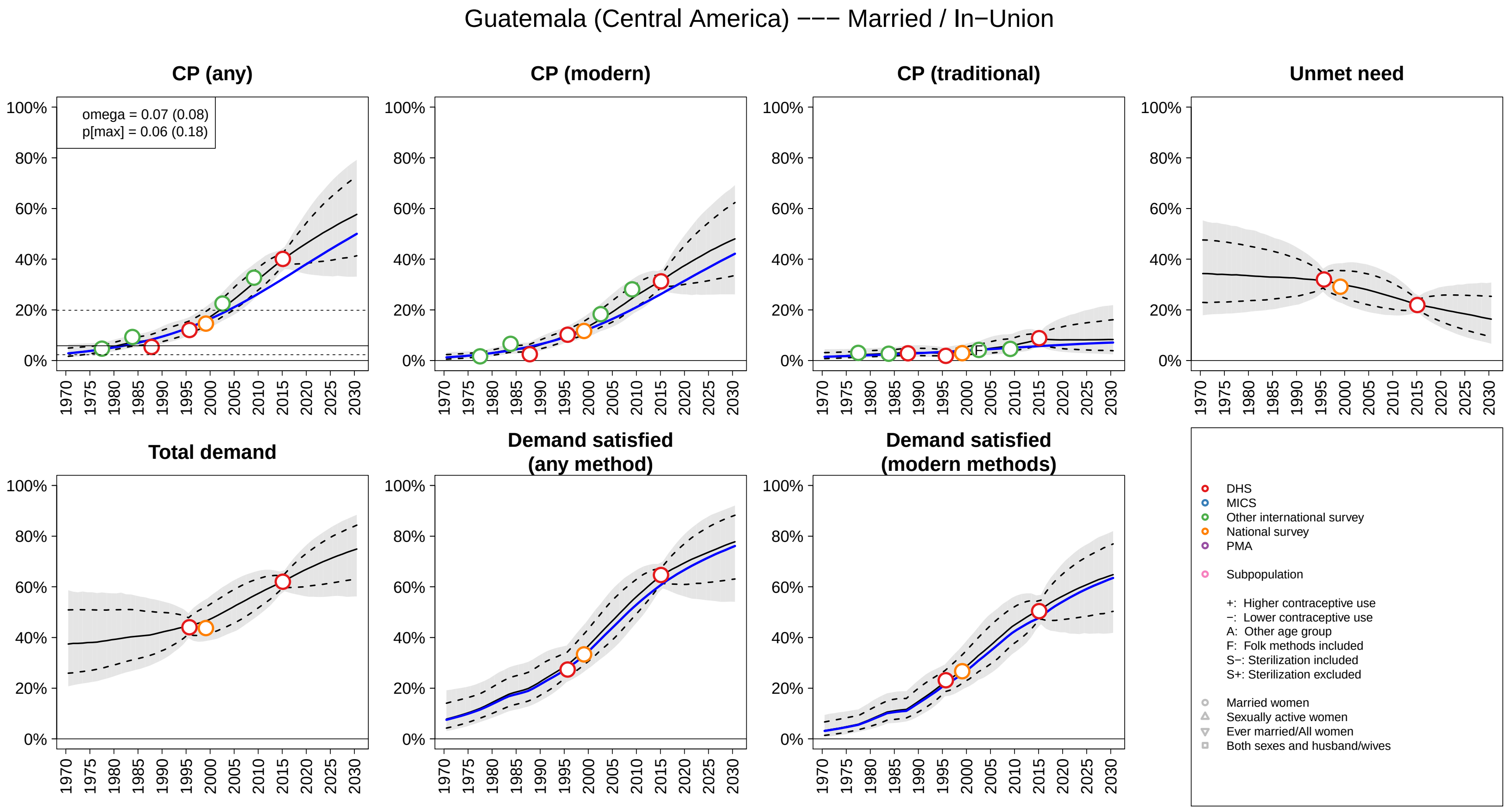

Guatemala (Central America) ––– Married / In−Union
CP (any)
omega = 0.07 (0.08)
p[max] = 0.06 (0.18)
CP (modern)
CP (traditional)
Unmet need
Total demand
Demand satisfied (any method)
Demand satisfied (modern methods)
0%
20%
40%
60%
80%
100%
1970
1975
1980
1985
1990
1995
2000
2005
2010
2015
2020
2025
2030
DHS
MICS
Other international survey
National survey
PMA
Subpopulation
+: Higher contraceptive use
−: Lower contraceptive use
A: Other age group
F: Folk methods included
S−: Sterilization included
S+: Sterilization excluded
Married women
Sexually active women
Ever married/All women
Both sexes and husband/wives

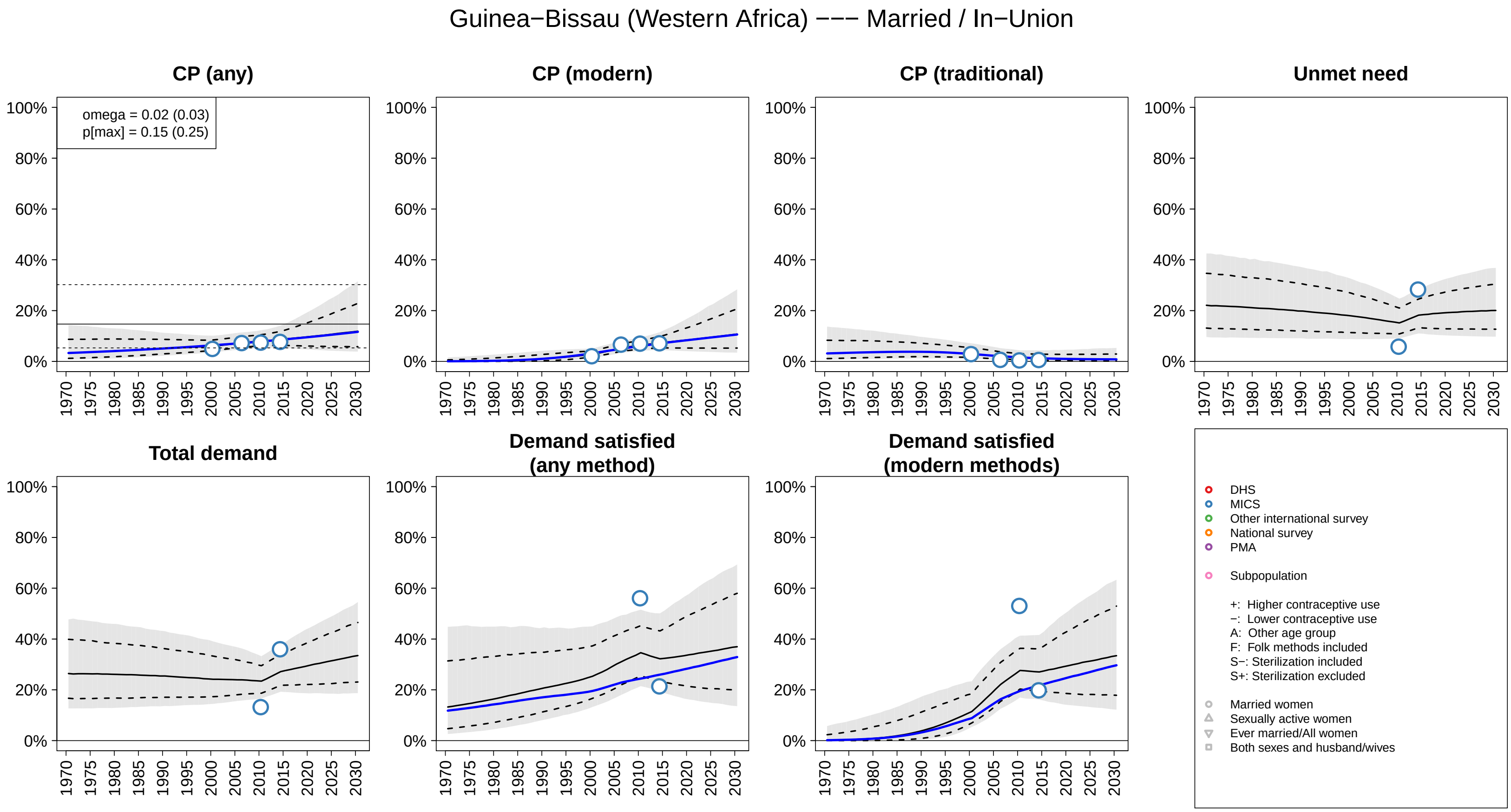
Guinea−Bissau (Western Africa) −−− Married / In−Union
CP (any)
omega = 0.02 (0.03)
p[max] = 0.15 (0.25)
CP (modern)
CP (traditional)
Unmet need
Total demand
Demand satisfied (any method)
Demand satisfied (modern methods)
DHS
MICS
Other international survey
National survey
PMA
Subpopulation
+: Higher contraceptive use
−: Lower contraceptive use
A: Other age group
F: Folk methods included
S−: Sterilization included
S+: Sterilization excluded
Married women
Sexually active women
Ever married/All women
Both sexes and husband/wives

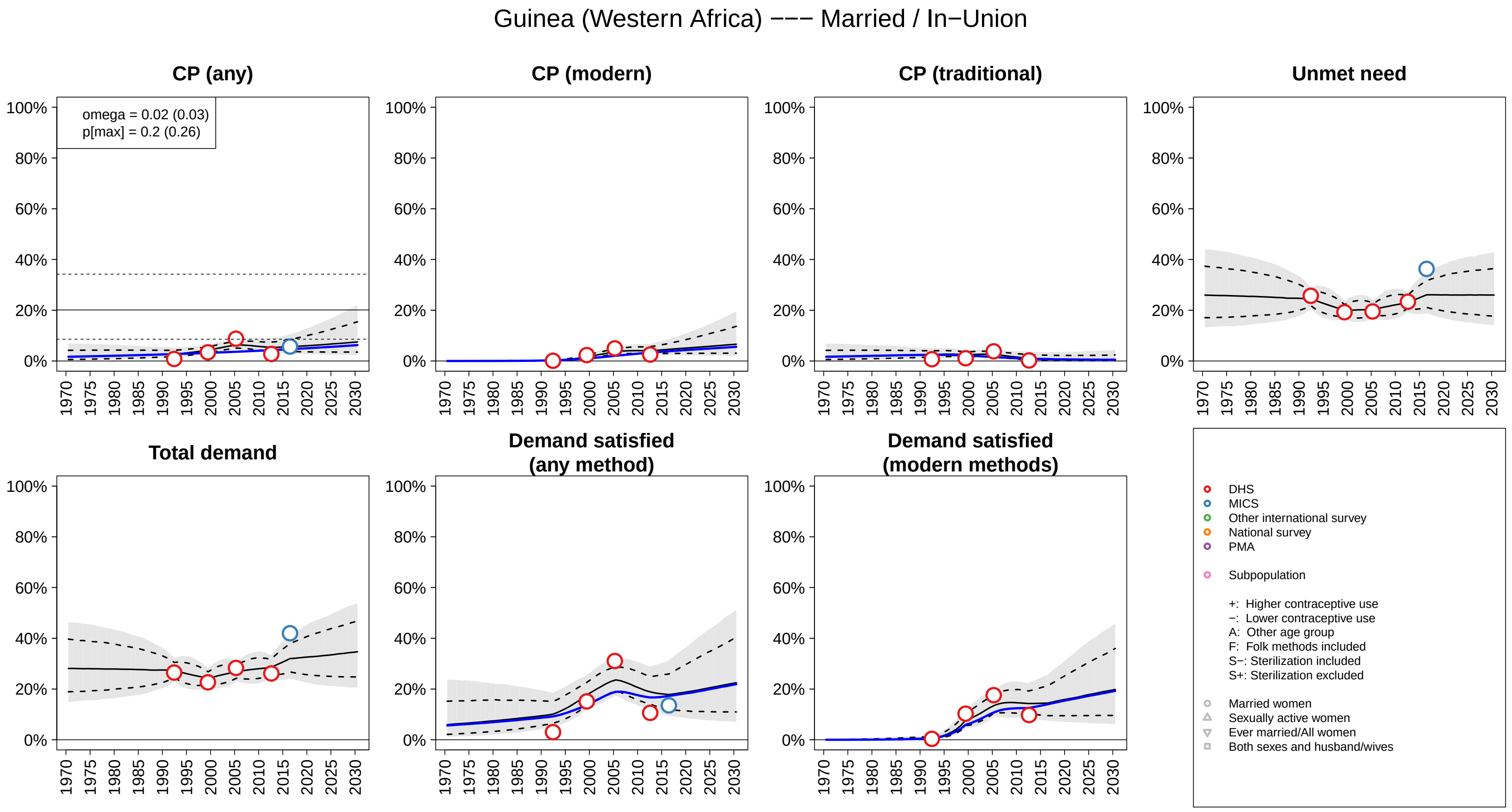
Guinea (Western Africa) ——— Married / In−Union
CP (any)
omega = 0.02 (0.03)
p[max] = 0.2 (0.26)
CP (modern)
CP (traditional)
Unmet need
Total demand
Demand satisfied (any method)
Demand satisfied (modern methods)
DHS
MICS
Other international survey
National survey
PMA
Subpopulation
+: Higher contraceptive use
−: Lower contraceptive use
A: Other age group
F: Folk methods included
S−: Sterilization included
S+: Sterilization excluded
Married women
Sexually active women
Ever married/All women
Both sexes and husband/wives

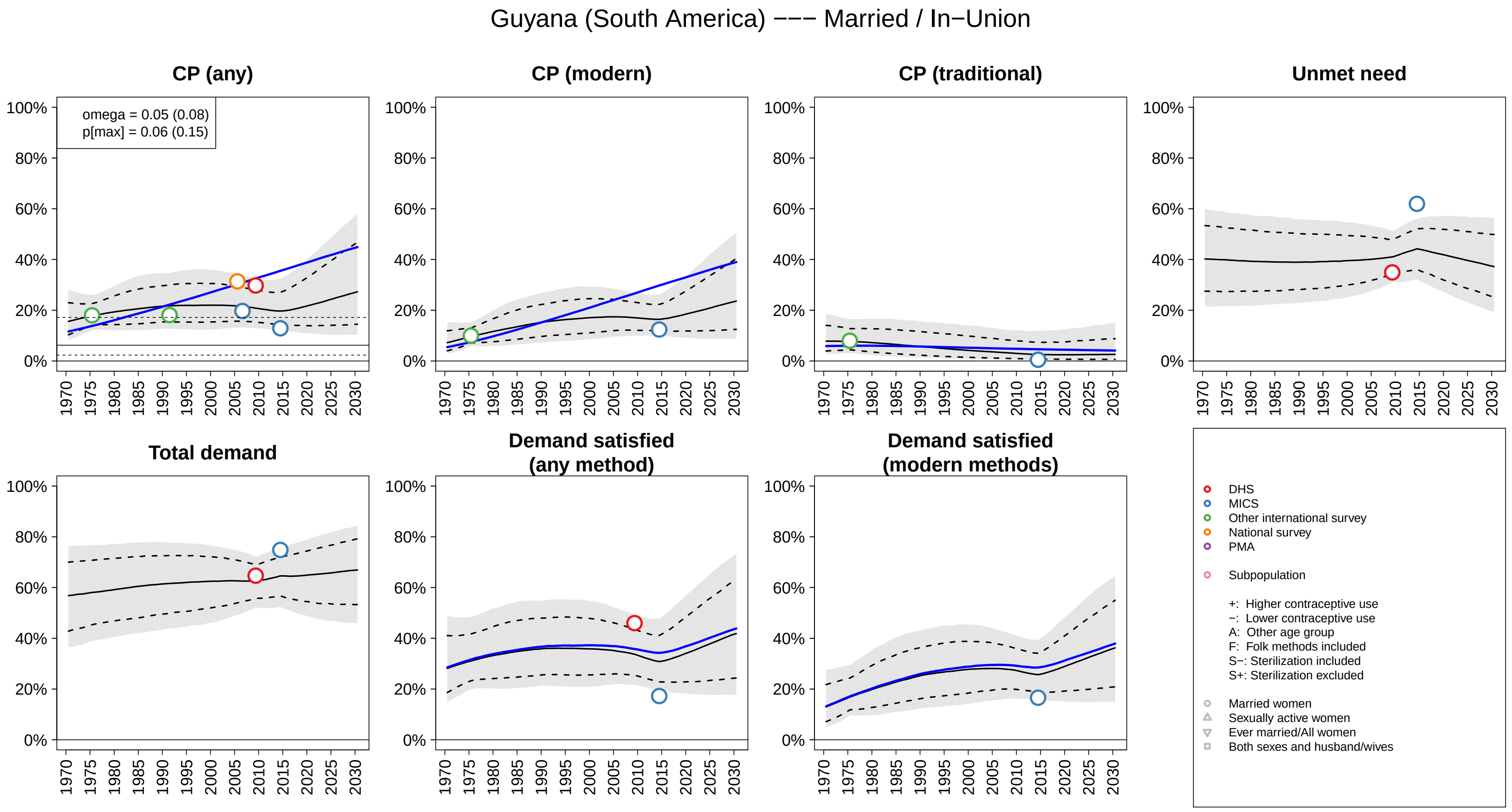
Guyana (South America) −−− Married / In−Union
CP (any)
omega = 0.05 (0.08)
p[max] = 0.06 (0.15)
CP (modern)
CP (traditional)
Unmet need
Total demand
Demand satisfied (any method)
Demand satisfied (modern methods)
DHS
MICS
Other international survey
National survey
PMA
Subpopulation
+: Higher contraceptive use
−: Lower contraceptive use
A: Other age group
F: Folk methods included
S−: Sterilization included
S+: Sterilization excluded
Married women
Sexually active women
Ever married/All women
Both sexes and husband/wives

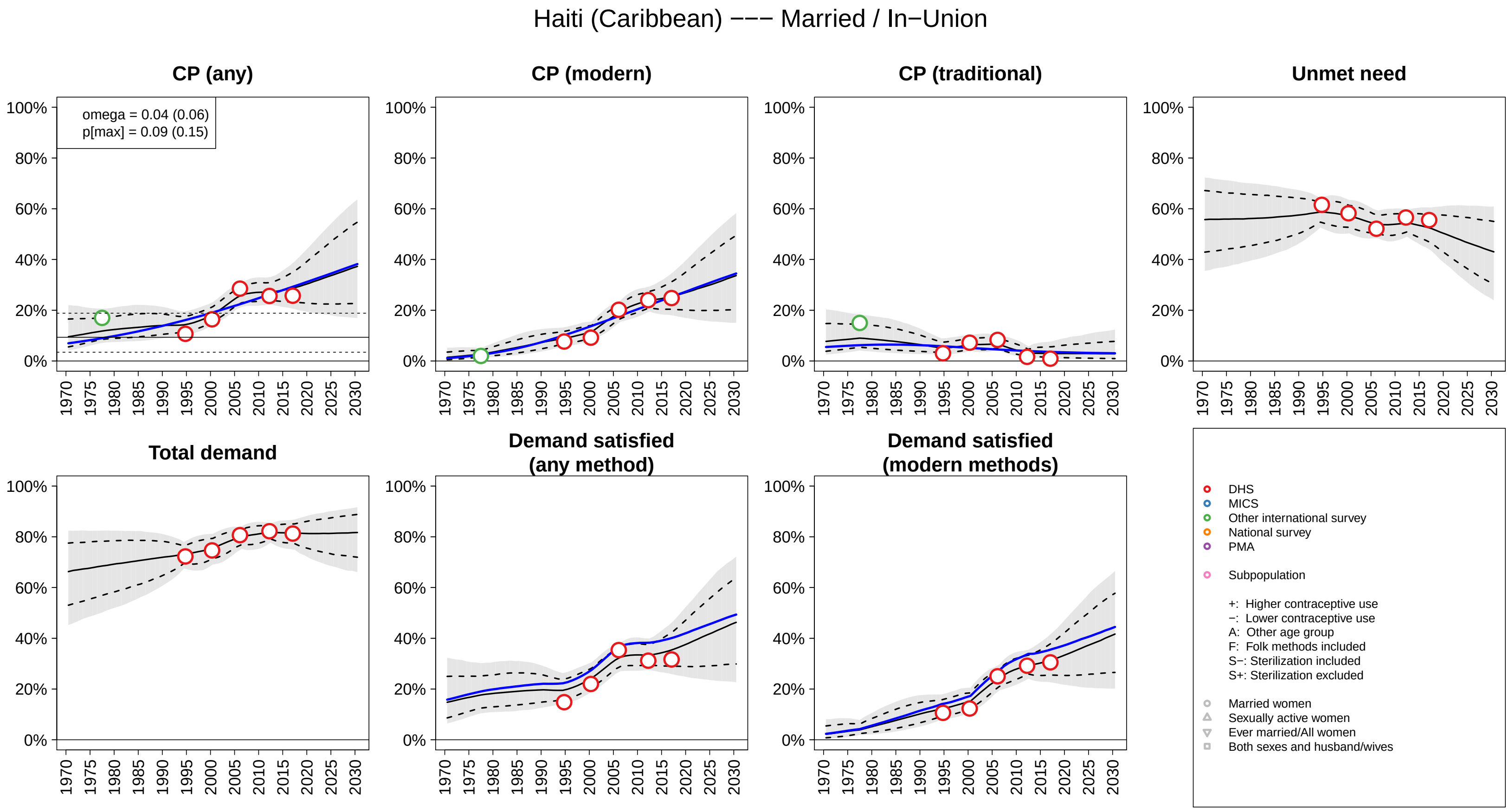
Haiti (Caribbean) ––– Married / In–Union
CP (any)
omega = 0.04 (0.06)
p[max] = 0.09 (0.15)
CP (modern)
CP (traditional)
Unmet need
Total demand
Demand satisfied (any method)
Demand satisfied (modern methods)
DHS
MICS
Other international survey
National survey
PMA
Subpopulation
+: Higher contraceptive use
−: Lower contraceptive use
A: Other age group
F: Folk methods included
S−: Sterilization included
S+: Sterilization excluded
Married women
Sexually active women
Ever married/All women
Both sexes and husband/wives

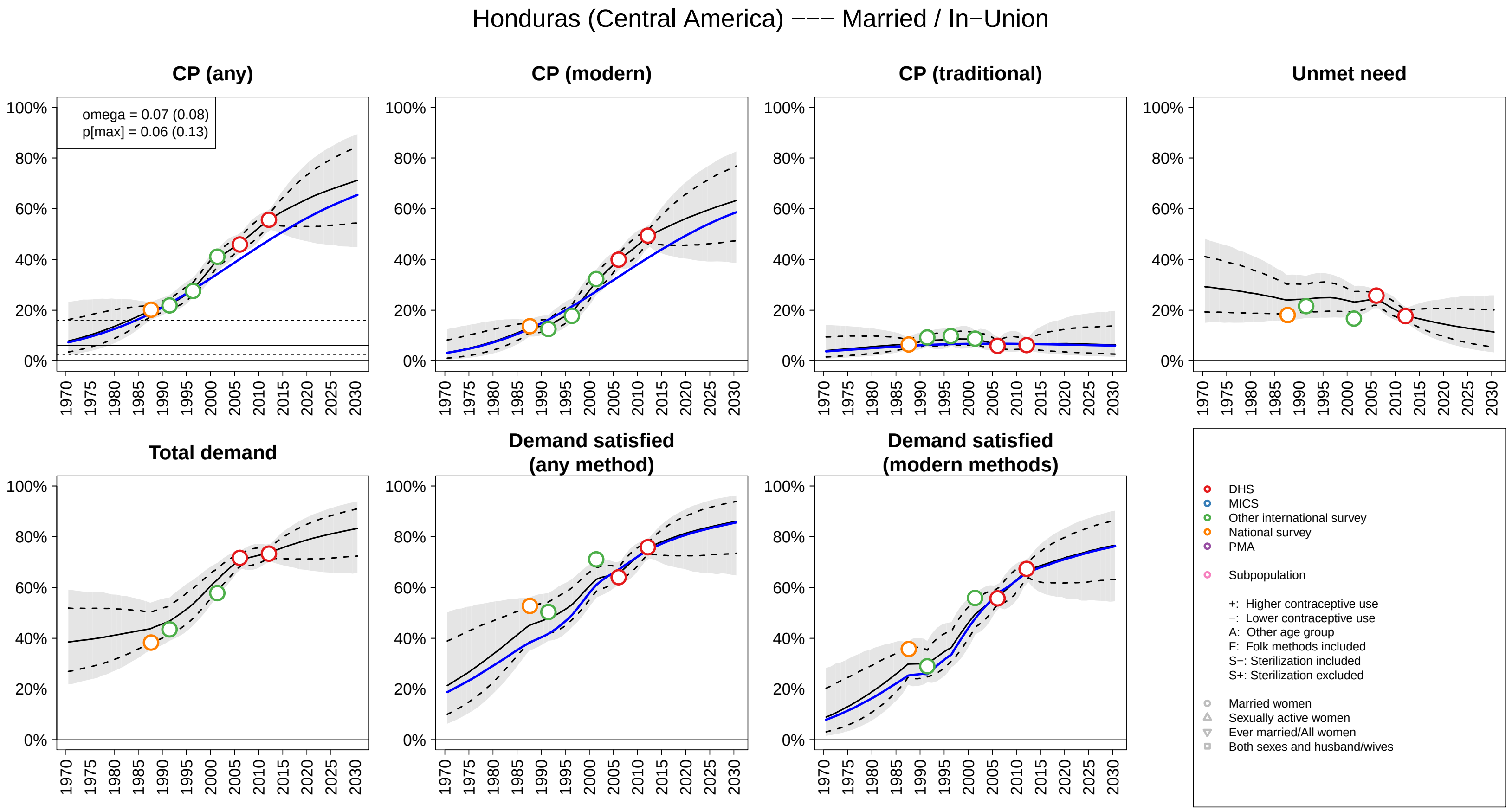
Honduras (Central America) ––– Married / In−Union
CP (any)
omega = 0.07 (0.08)
p[max] = 0.06 (0.13)
CP (modern)
CP (traditional)
Unmet need
Total demand
Demand satisfied (any method)
Demand satisfied (modern methods)
DHS
MICS
Other international survey
National survey
PMA
Subpopulation
+: Higher contraceptive use
−: Lower contraceptive use
A: Other age group
F: Folk methods included
S−: Sterilization included
S+: Sterilization excluded
Married women
Sexually active women
Ever married/All women
Both sexes and husband/wives

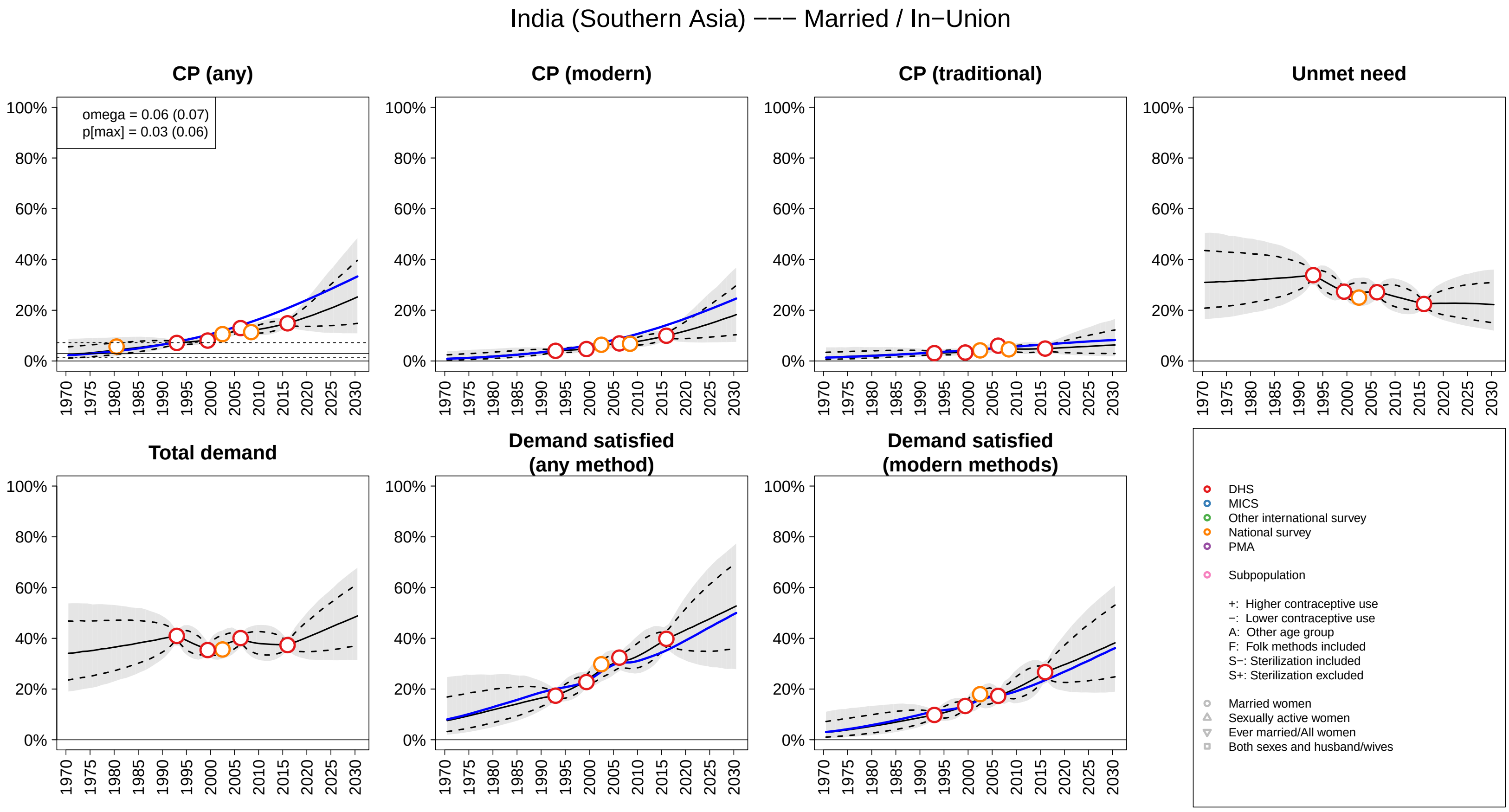
India (Southern Asia) --- Married / In−Union
CP (any)
omega = 0.06 (0.07)
p[max] = 0.03 (0.06)
CP (modern)
CP (traditional)
Unmet need
Total demand
Demand satisfied (any method)
Demand satisfied (modern methods)
DHS
MICS
Other international survey
National survey
PMA
Subpopulation
+: Higher contraceptive use
−: Lower contraceptive use
A: Other age group
F: Folk methods included
S−: Sterilization included
S+: Sterilization excluded
Married women
Sexually active women
Ever married/All women
Both sexes and husband/wives

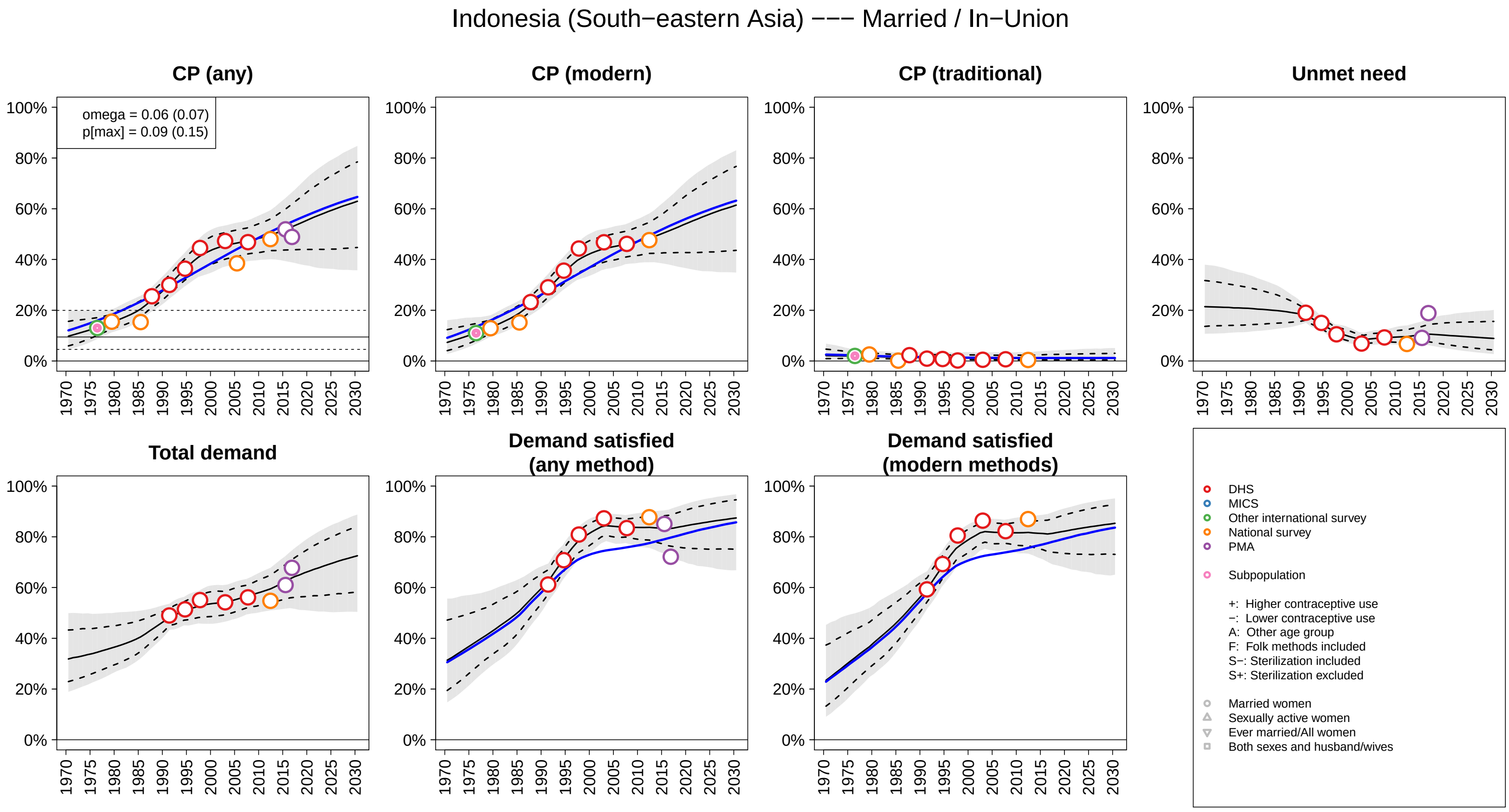
Indonesia (South−eastern Asia) −−− Married / In−Union
CP (any)
omega = 0.06 (0.07)
p[max] = 0.09 (0.15)
CP (modern)
CP (traditional)
Unmet need
Total demand
Demand satisfied (any method)
Demand satisfied (modern methods)
DHS
MICS
Other international survey
National survey
PMA
Subpopulation
+: Higher contraceptive use
−: Lower contraceptive use
A: Other age group
F: Folk methods included
S−: Sterilization included
S+: Sterilization excluded
Married women
Sexually active women
Ever married/All women
Both sexes and husband/wives

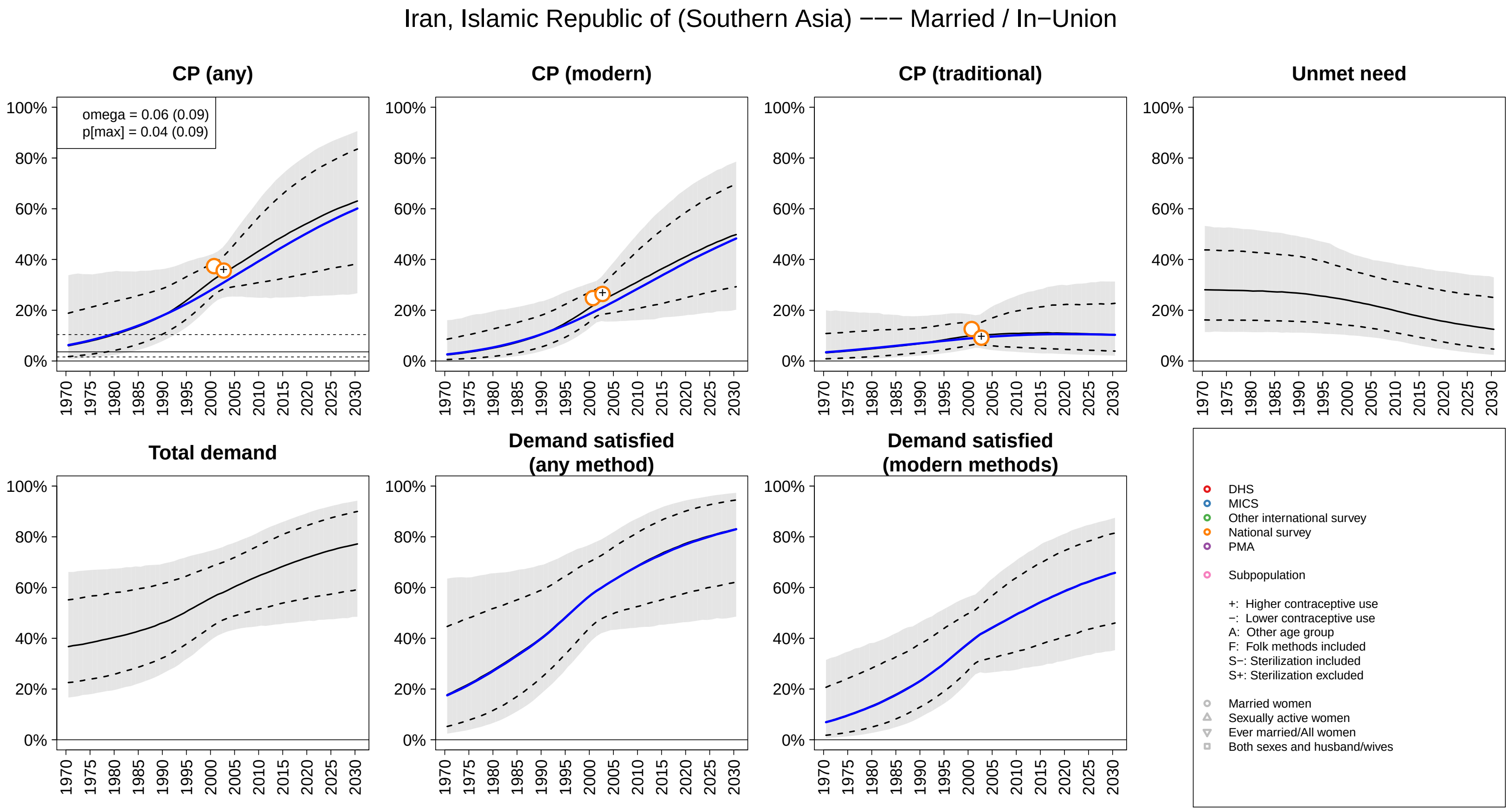
Iran, Islamic Republic of (Southern Asia) ––– Married / In–Union
CP (any)
omega = 0.06 (0.09)
p[max] = 0.04 (0.09)
CP (modern)
CP (traditional)
Unmet need
Total demand
Demand satisfied (any method)
Demand satisfied (modern methods)
DHS
MICS
Other international survey
National survey
PMA
Subpopulation
+: Higher contraceptive use
−: Lower contraceptive use
A: Other age group
F: Folk methods included
S−: Sterilization included
S+: Sterilization excluded
Married women
Sexually active women
Ever married/All women
Both sexes and husband/wives

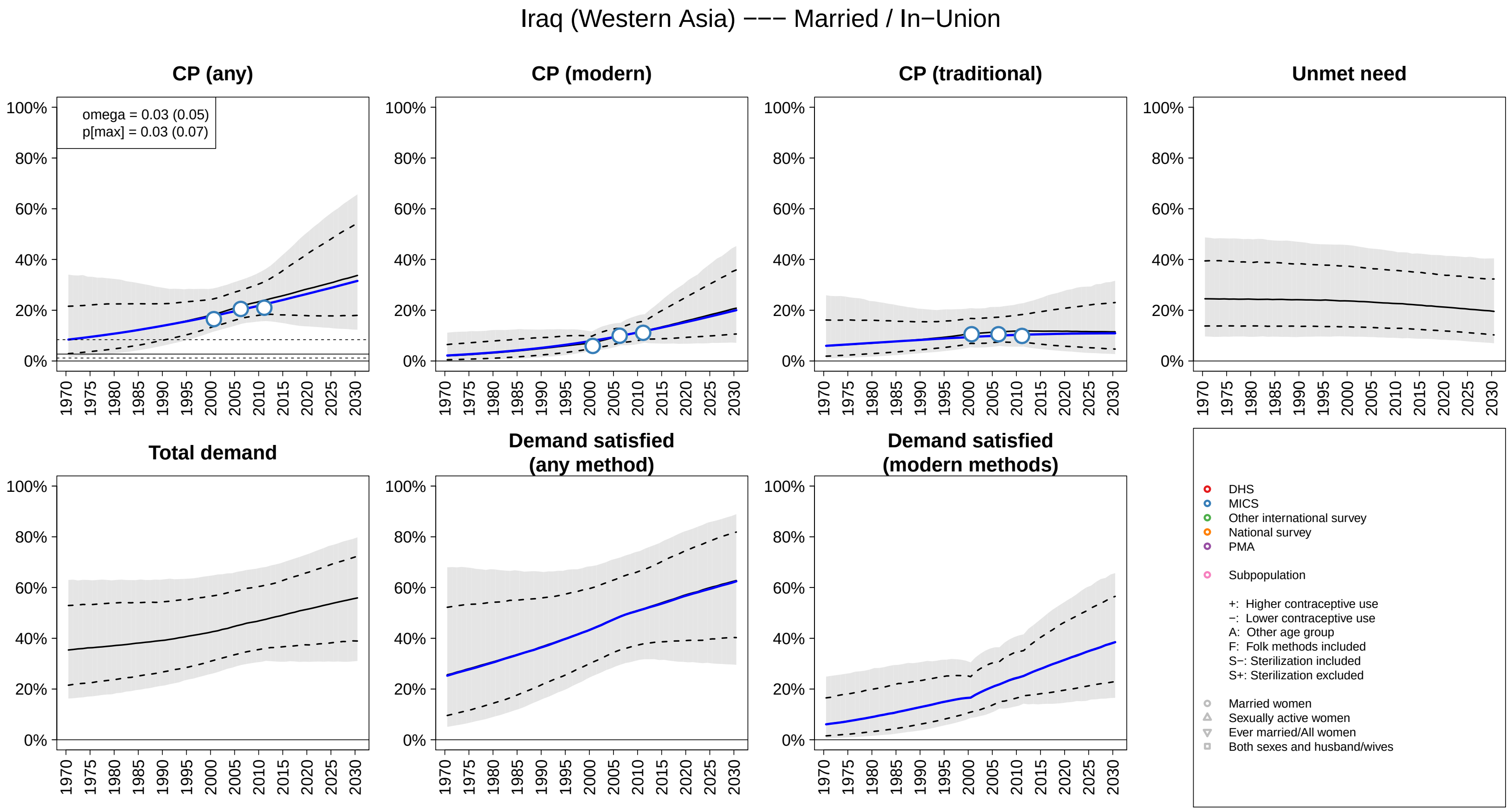
Iraq (Western Asia) ––– Married / In−Union
CP (any)
omega = 0.03 (0.05)
p[max] = 0.03 (0.07)
CP (modern)
CP (traditional)
Unmet need
Total demand
Demand satisfied (any method)
Demand satisfied (modern methods)
0%
20%
40%
60%
80%
100%
1970
1975
1980
1985
1990
1995
2000
2005
2010
2015
2020
2025
2030
DHS
MICS
Other international survey
National survey
PMA
Subpopulation
+: Higher contraceptive use
−: Lower contraceptive use
A: Other age group
F: Folk methods included
S−: Sterilization included
S+: Sterilization excluded
Married women
Sexually active women
Ever married/All women
Both sexes and husband/wives

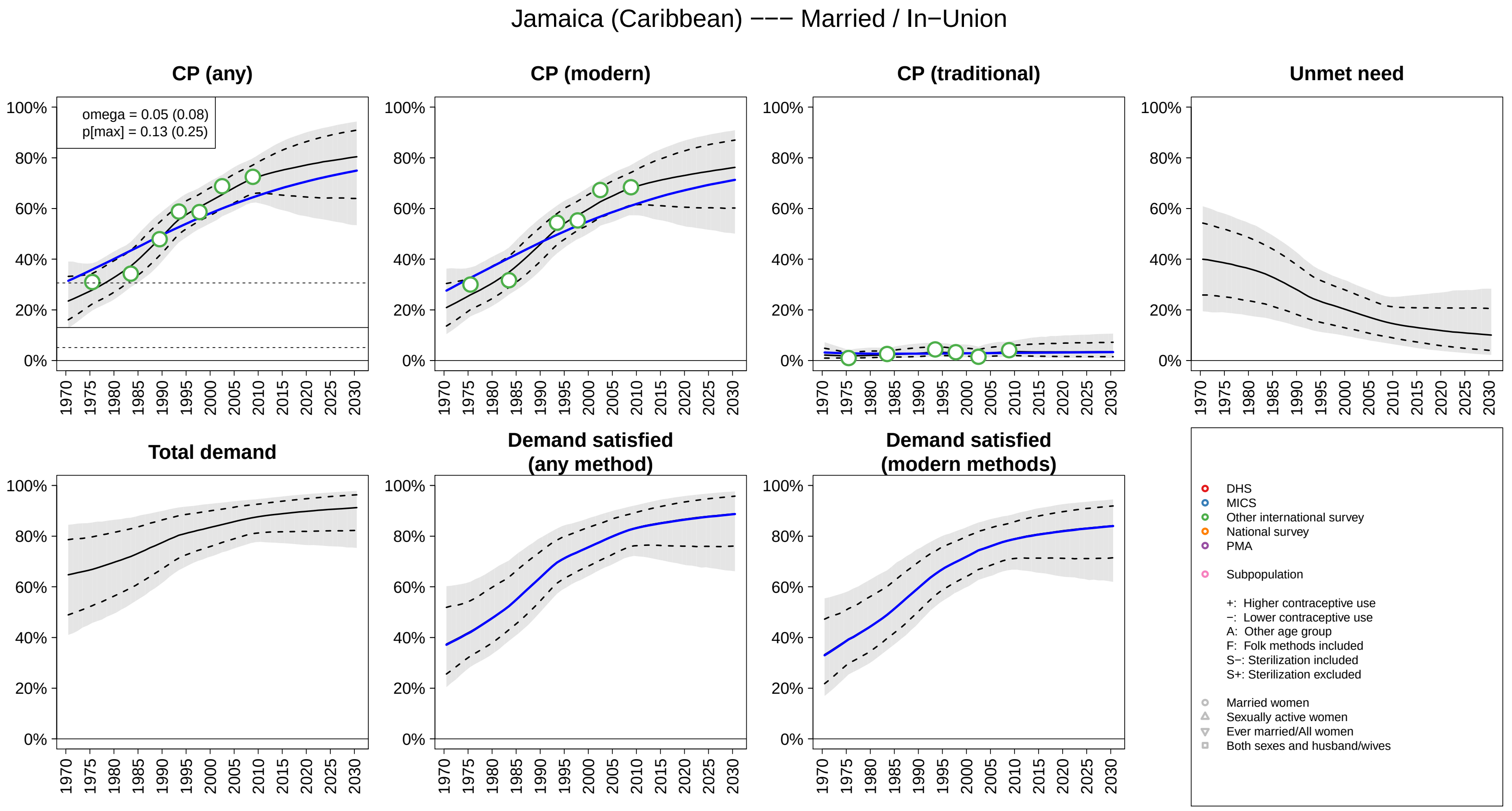
Jamaica (Caribbean) --- Married / In−Union
CP (any)
omega = 0.05 (0.08)
p[max] = 0.13 (0.25)
CP (modern)
CP (traditional)
Unmet need
Total demand
Demand satisfied (any method)
Demand satisfied (modern methods)
0%
20%
40%
60%
80%
100%
1970
1975
1980
1985
1990
1995
2000
2005
2010
2015
2020
2025
2030
DHS
MICS
Other international survey
National survey
PMA
Subpopulation
+: Higher contraceptive use
−: Lower contraceptive use
A: Other age group
F: Folk methods included
S−: Sterilization included
S+: Sterilization excluded
Married women
Sexually active women
Ever married/All women
Both sexes and husband/wives

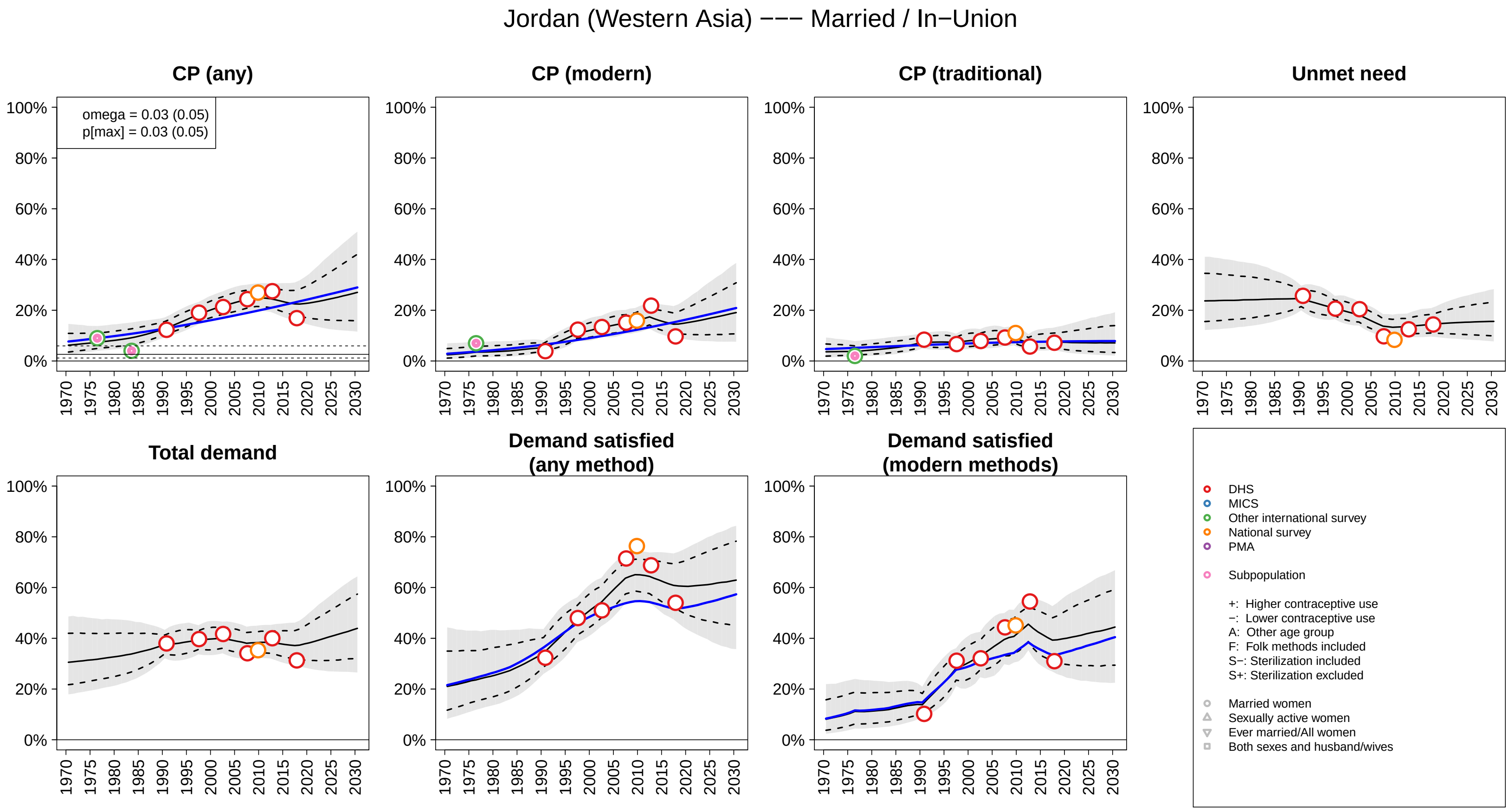

Jordan (Western Asia) −−− Married / In−Union
CP (any)
omega = 0.03 (0.05)
p[max] = 0.03 (0.05)
CP (modern)
CP (traditional)
Unmet need
Total demand
Demand satisfied (any method)
Demand satisfied (modern methods)
DHS
MICS
Other international survey
National survey
PMA
Subpopulation
+: Higher contraceptive use
−: Lower contraceptive use
A: Other age group
F: Folk methods included
S−: Sterilization included
S+: Sterilization excluded
Married women
Sexually active women
Ever married/All women
Both sexes and husband/wives

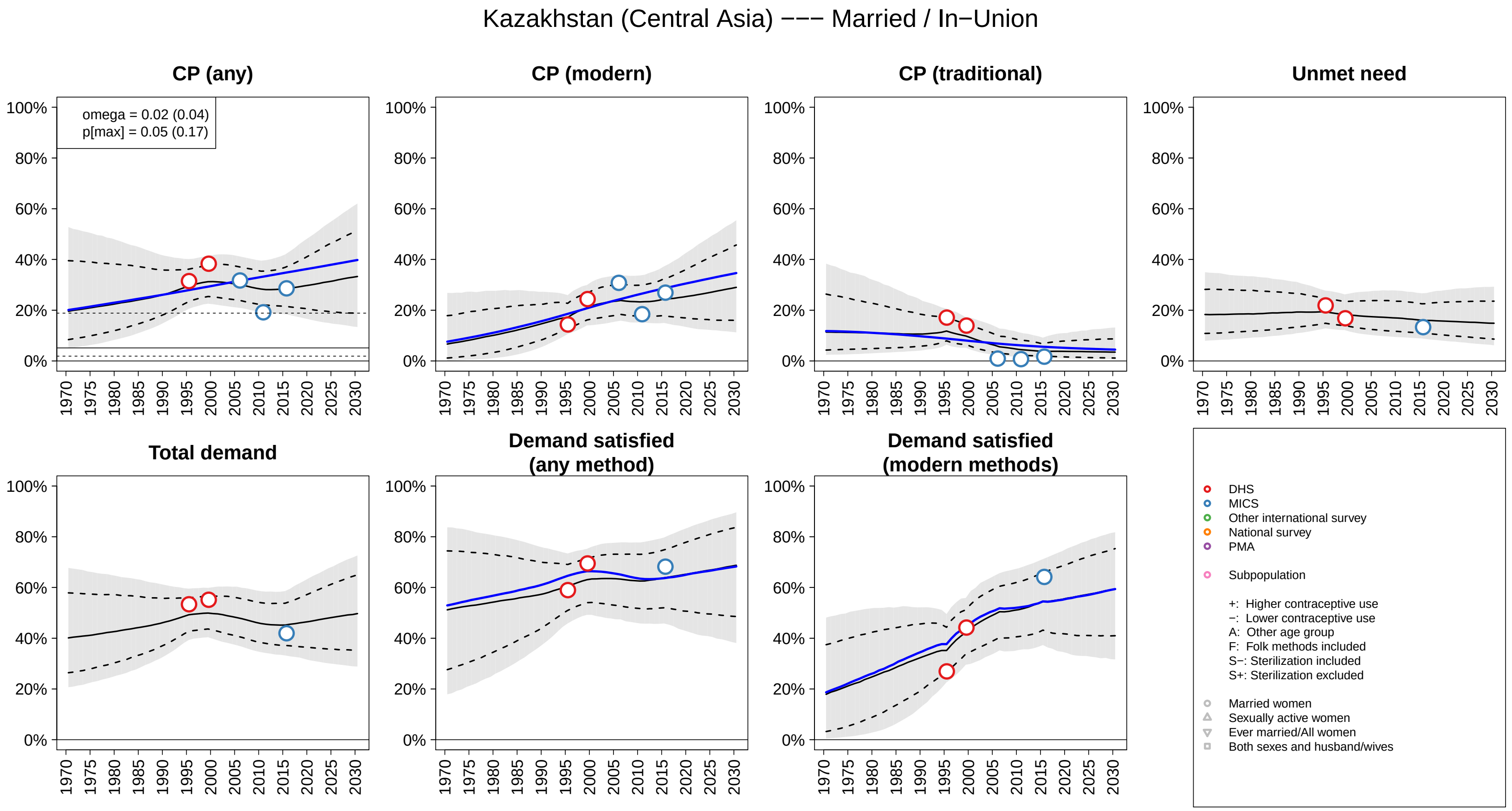
Kazakhstan (Central Asia) ––– Married / In−Union
CP (any)
omega = 0.02 (0.04)
p[max] = 0.05 (0.17)
CP (modern)
CP (traditional)
Unmet need
Total demand
Demand satisfied (any method)
Demand satisfied (modern methods)
0%
20%
40%
60%
80%
100%
1970
1975
1980
1985
1990
1995
2000
2005
2010
2015
2020
2025
2030
DHS
MICS
Other international survey
National survey
PMA
Subpopulation
+: Higher contraceptive use
−: Lower contraceptive use
A: Other age group
F: Folk methods included
S−: Sterilization included
S+: Sterilization excluded
Married women
Sexually active women
Ever married/All women
Both sexes and husband/wives

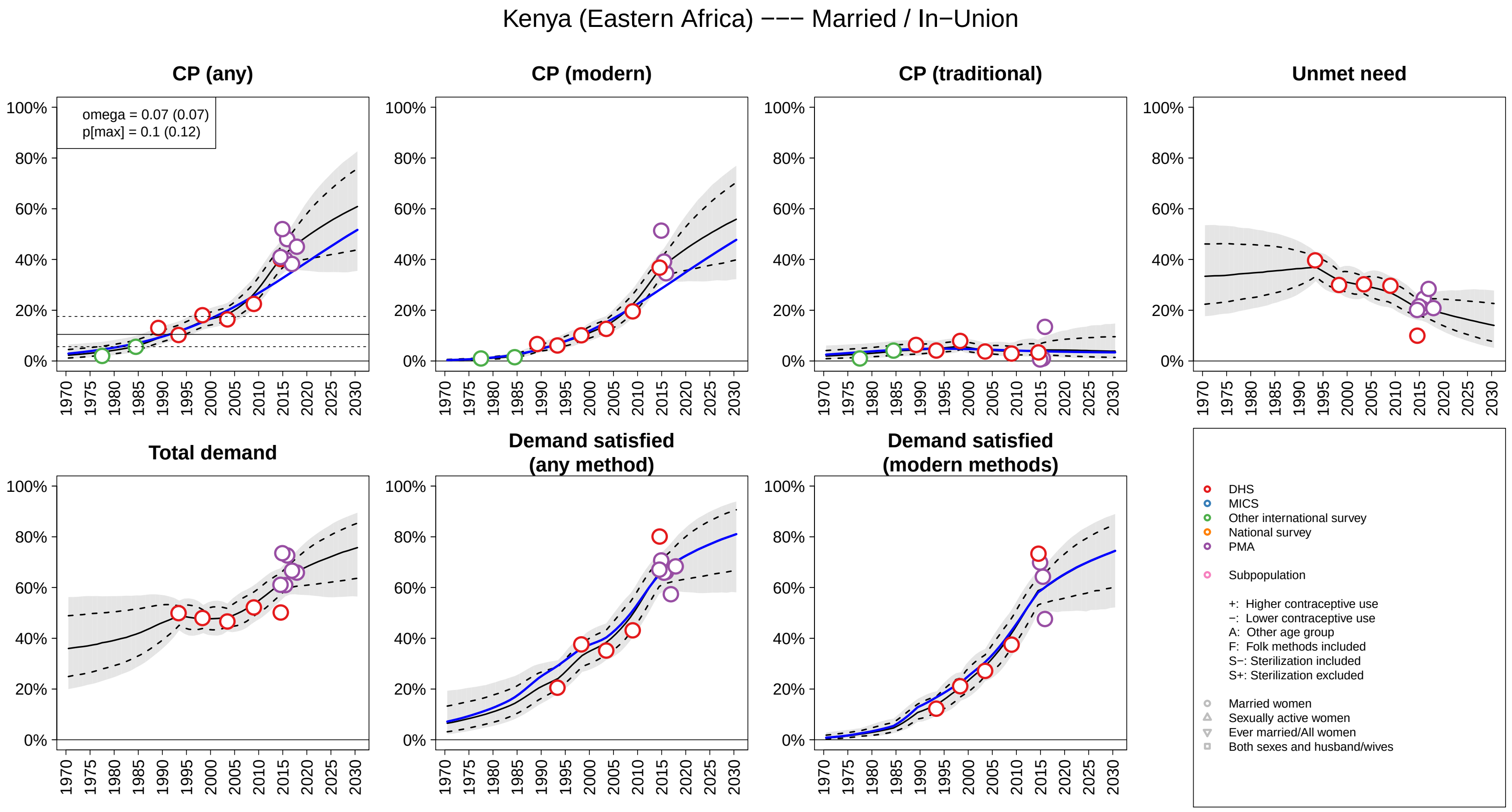
Kenya (Eastern Africa) --- Married / In-Union
CP (any)
omega = 0.07 (0.07)
p[max] = 0.1 (0.12)
CP (modern)
CP (traditional)
Unmet need
Total demand
Demand satisfied (any method)
Demand satisfied (modern methods)
DHS
MICS
Other international survey
National survey
PMA
Subpopulation
+: Higher contraceptive use
−: Lower contraceptive use
A: Other age group
F: Folk methods included
S−: Sterilization included
S+: Sterilization excluded
Married women
Sexually active women
Ever married/All women
Both sexes and husband/wives

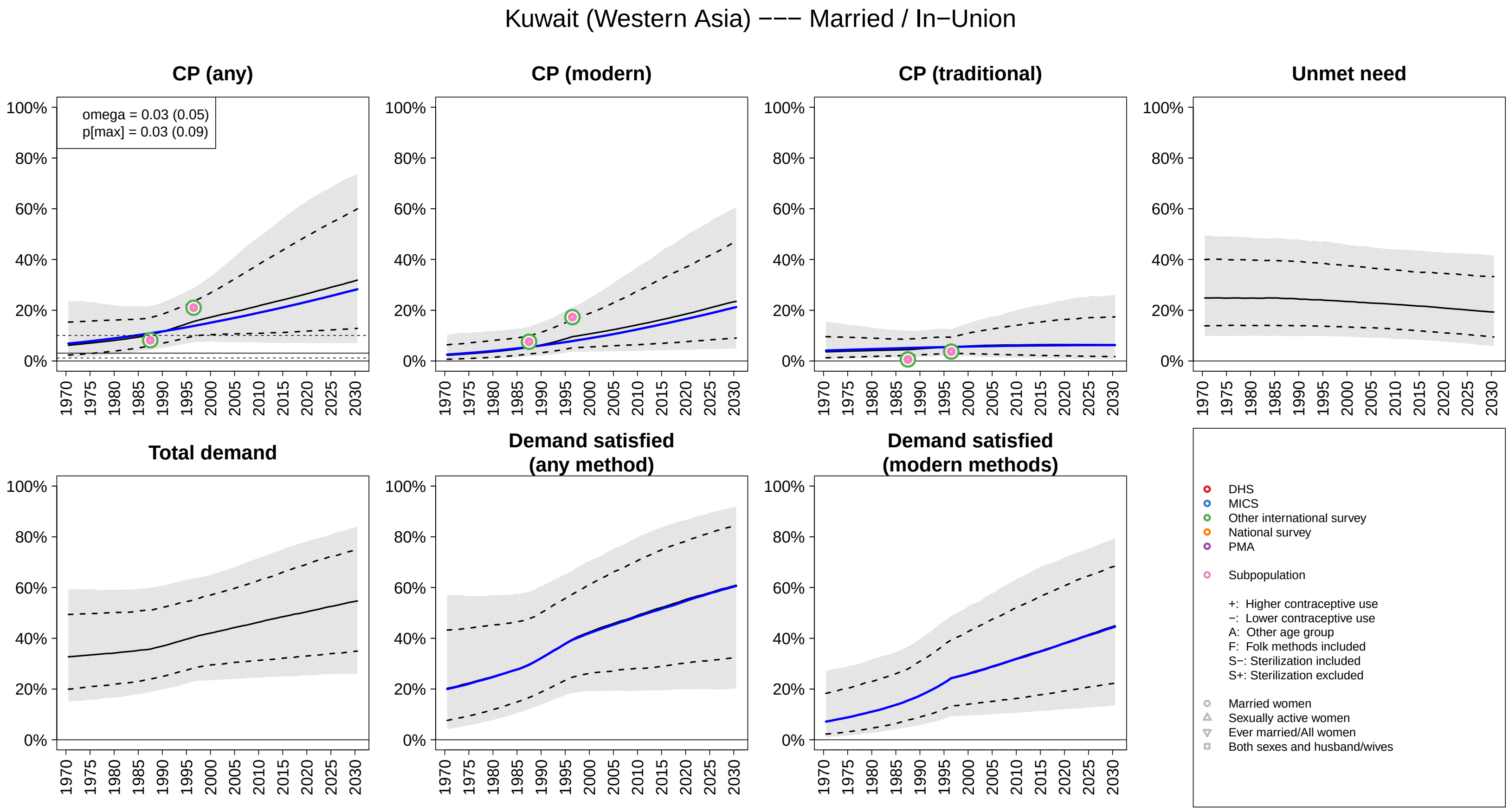
Kuwait (Western Asia) −−− Married / In−Union
CP (any)
omega = 0.03 (0.05)
p[max] = 0.03 (0.09)
CP (modern)
CP (traditional)
Unmet need
Total demand
Demand satisfied (any method)
Demand satisfied (modern methods)
0%
20%
40%
60%
80%
100%
1970
1975
1980
1985
1990
1995
2000
2005
2010
2015
2020
2025
2030
DHS
MICS
Other international survey
National survey
PMA
Subpopulation
+: Higher contraceptive use
−: Lower contraceptive use
A: Other age group
F: Folk methods included
S−: Sterilization included
S+: Sterilization excluded
Married women
Sexually active women
Ever married/All women
Both sexes and husband/wives

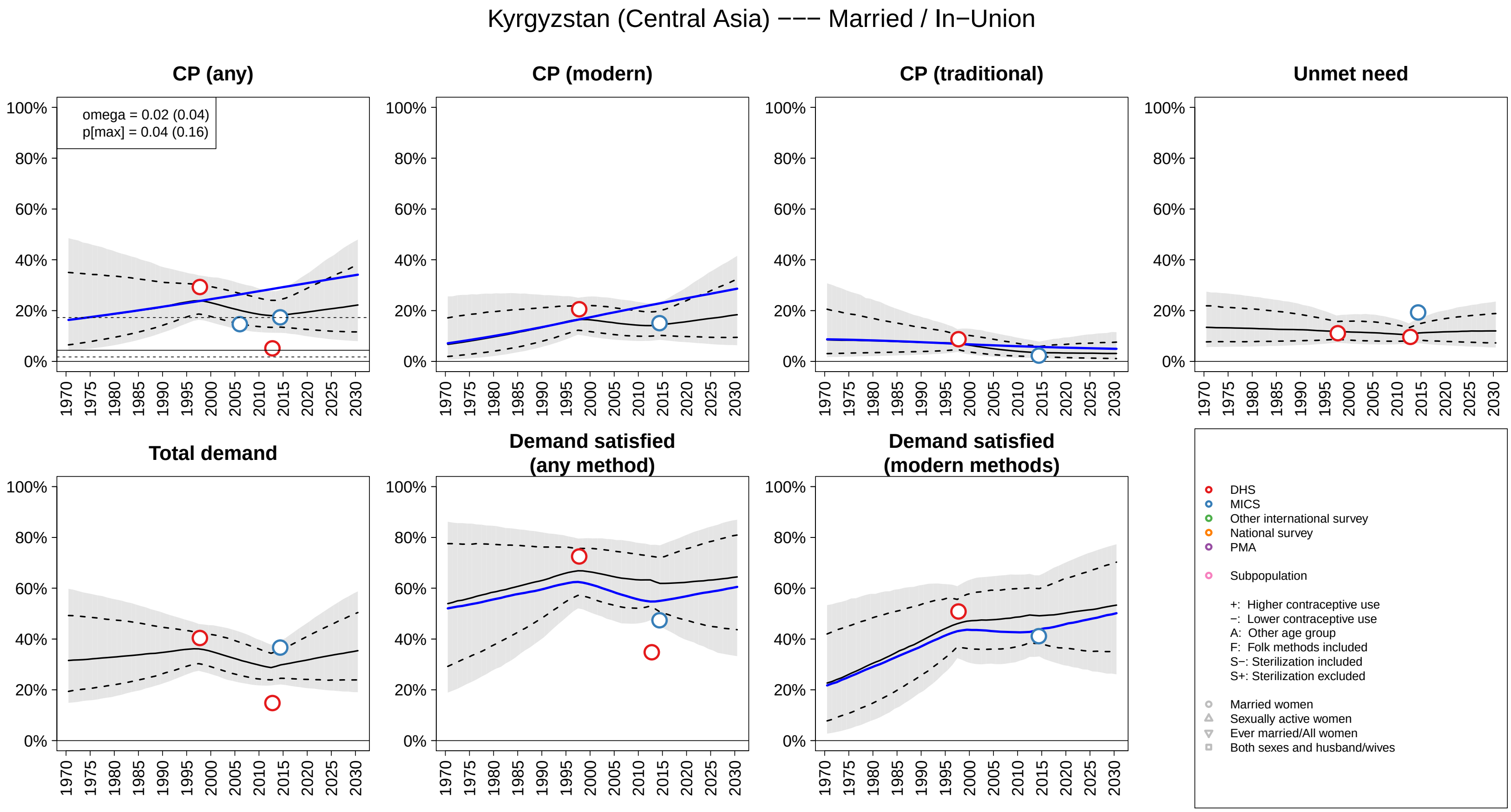
Kyrgyzstan (Central Asia) −−− Married / In−Union
CP (any)
omega = 0.02 (0.04)
p[max] = 0.04 (0.16)
CP (modern)
CP (traditional)
Unmet need
Total demand
Demand satisfied (any method)
Demand satisfied (modern methods)
DHS
MICS
Other international survey
National survey
PMA
Subpopulation
+: Higher contraceptive use
−: Lower contraceptive use
A: Other age group
F: Folk methods included
S−: Sterilization included
S+: Sterilization excluded
Married women
Sexually active women
Ever married/All women
Both sexes and husband/wives

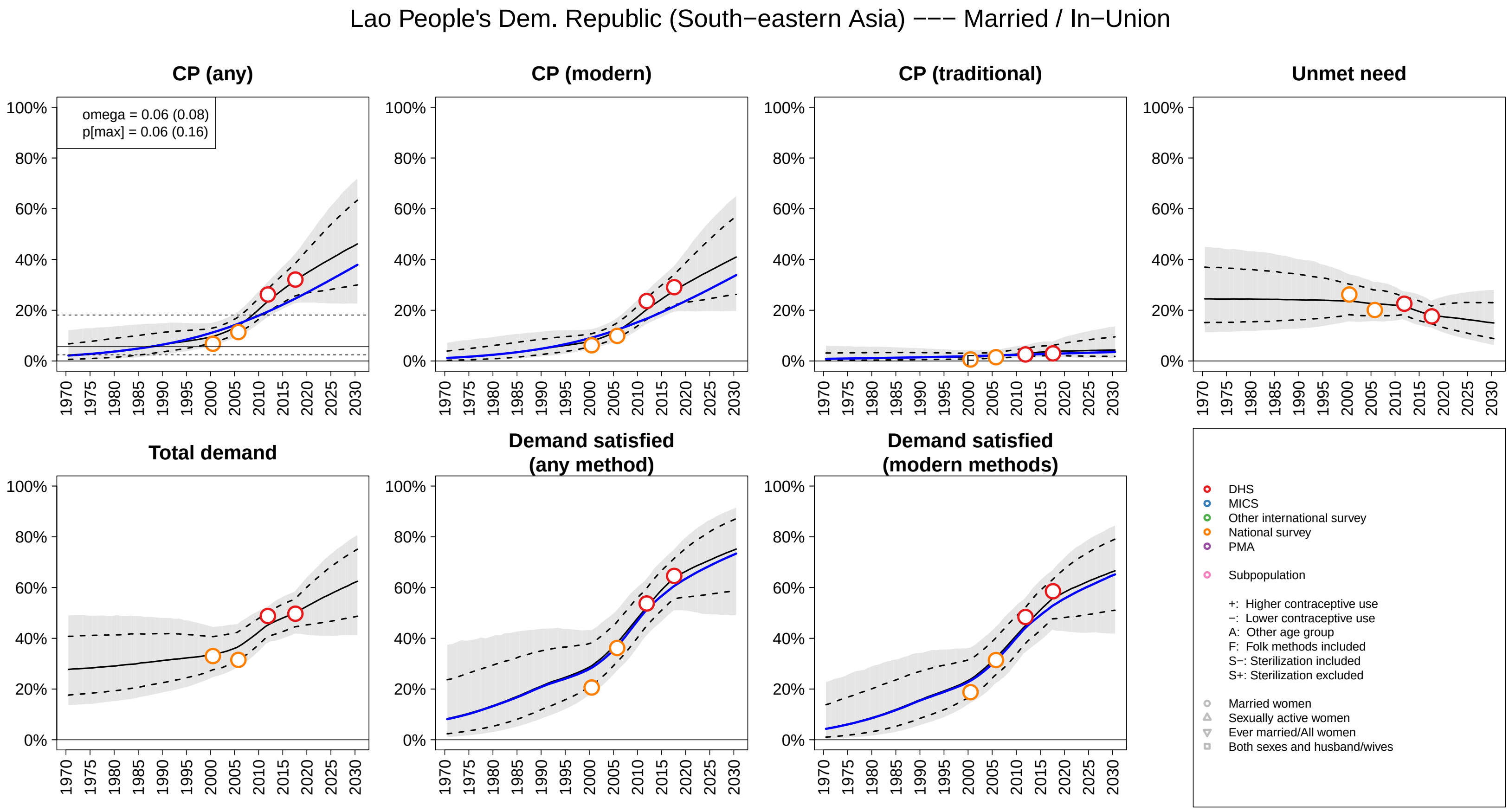

Lao People's Dem. Republic (South−eastern Asia) −−− Married / In−Union
CP (any)
omega = 0.06 (0.08)
p[max] = 0.06 (0.16)
CP (modern)
CP (traditional)
Unmet need
Total demand
Demand satisfied (any method)
Demand satisfied (modern methods)
DHS
MICS
Other international survey
National survey
PMA
Subpopulation
+: Higher contraceptive use
−: Lower contraceptive use
A: Other age group
F: Folk methods included
S−: Sterilization included
S+: Sterilization excluded
Married women
Sexually active women
Ever married/All women
Both sexes and husband/wives

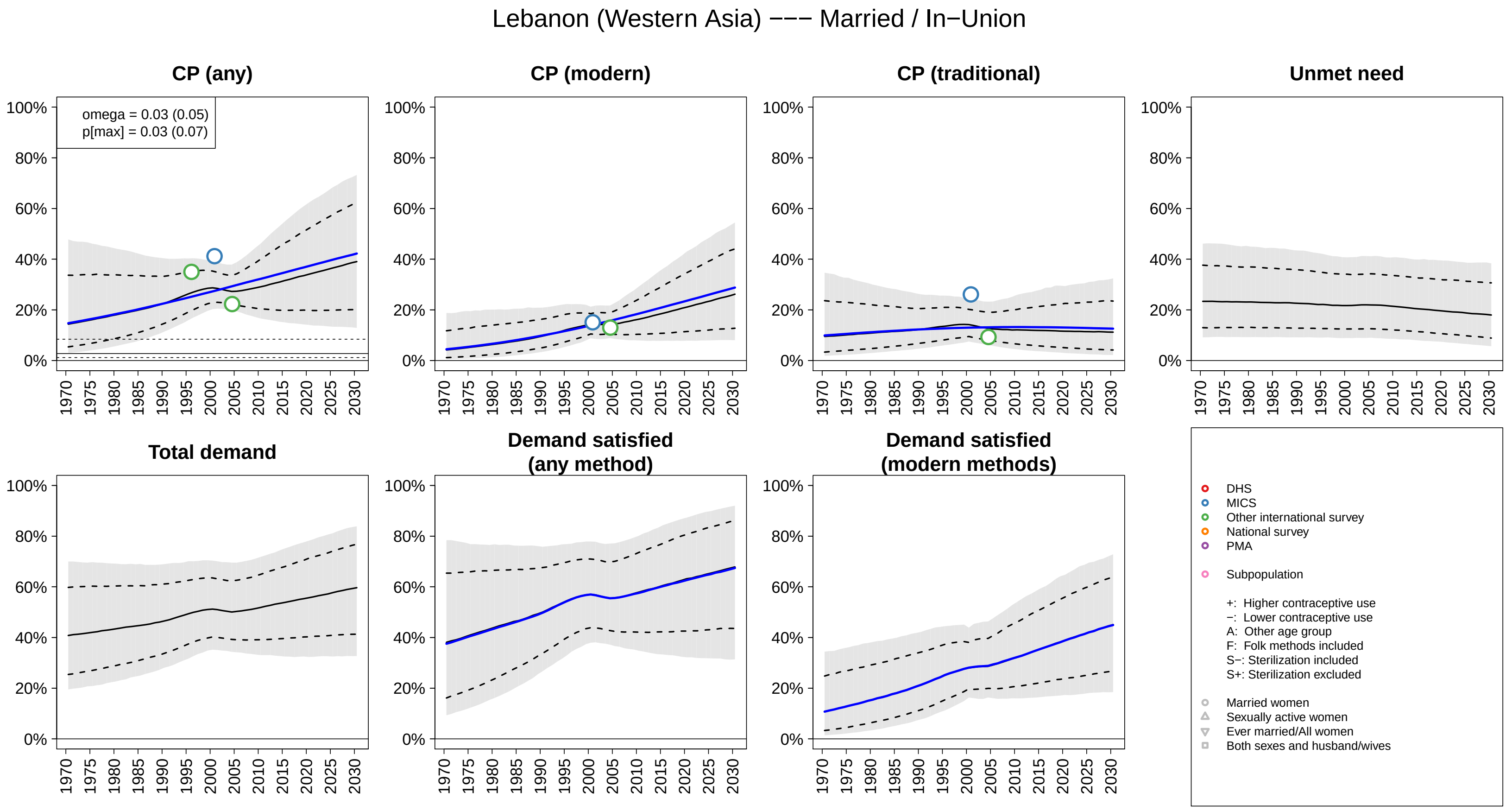
Lebanon (Western Asia) −−− Married / In−Union
CP (any)
omega = 0.03 (0.05)
p[max] = 0.03 (0.07)
CP (modern)
CP (traditional)
Unmet need
Total demand
Demand satisfied (any method)
Demand satisfied (modern methods)
DHS
MICS
Other international survey
National survey
PMA
Subpopulation
+: Higher contraceptive use
−: Lower contraceptive use
A: Other age group
F: Folk methods included
S−: Sterilization included
S+: Sterilization excluded
Married women
Sexually active women
Ever married/All women
Both sexes and husband/wives

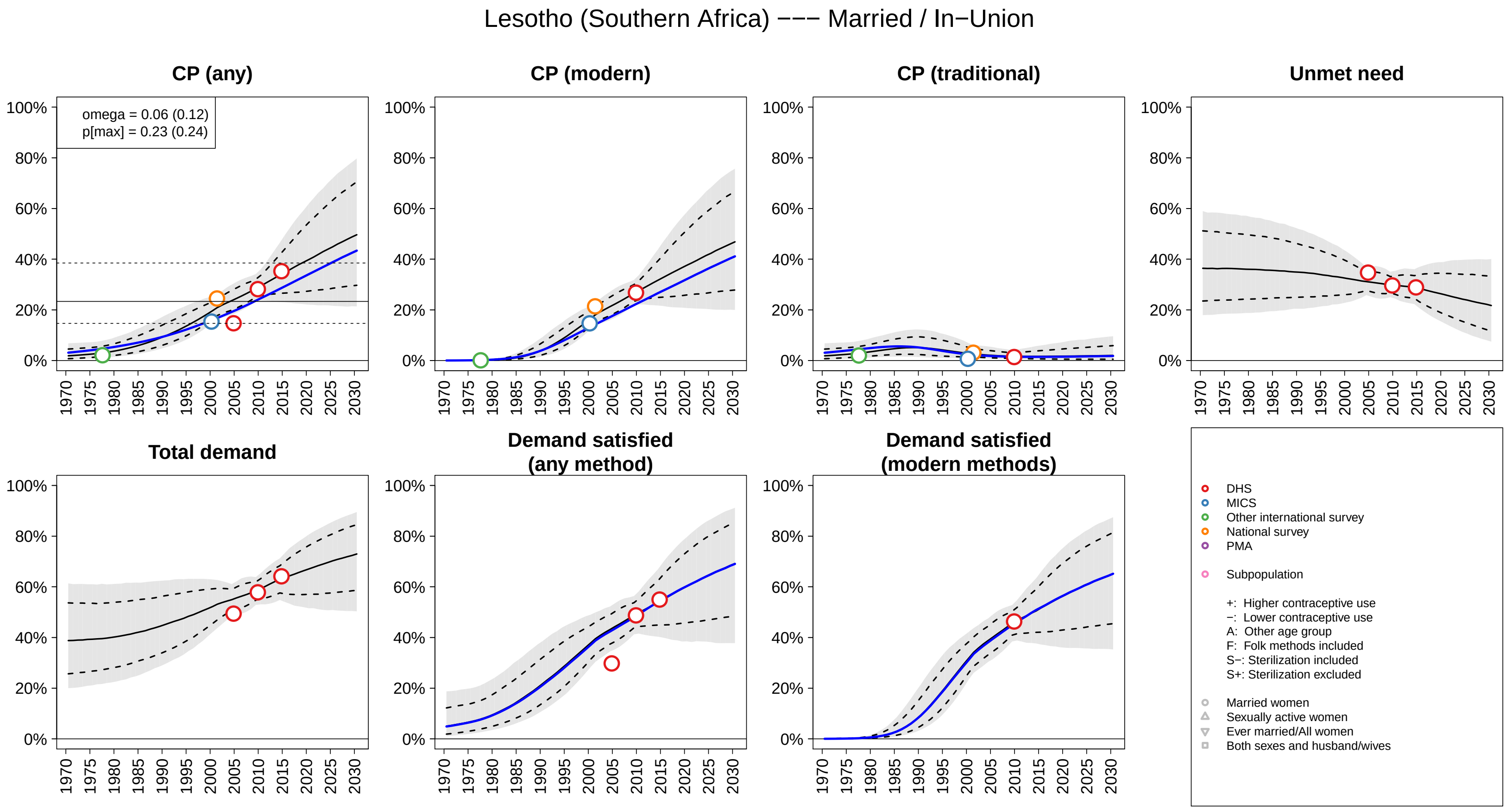
Lesotho (Southern Africa) ––– Married / In−Union
CP (any)
omega = 0.06 (0.12)
p[max] = 0.23 (0.24)
CP (modern)
CP (traditional)
Unmet need
Total demand
Demand satisfied (any method)
Demand satisfied (modern methods)
0%
20%
40%
60%
80%
100%
1970
1975
1980
1985
1990
1995
2000
2005
2010
2015
2020
2025
2030
DHS
MICS
Other international survey
National survey
PMA
Subpopulation
+: Higher contraceptive use
−: Lower contraceptive use
A: Other age group
F: Folk methods included
S−: Sterilization included
S+: Sterilization excluded
Married women
Sexually active women
Ever married/All women
Both sexes and husband/wives

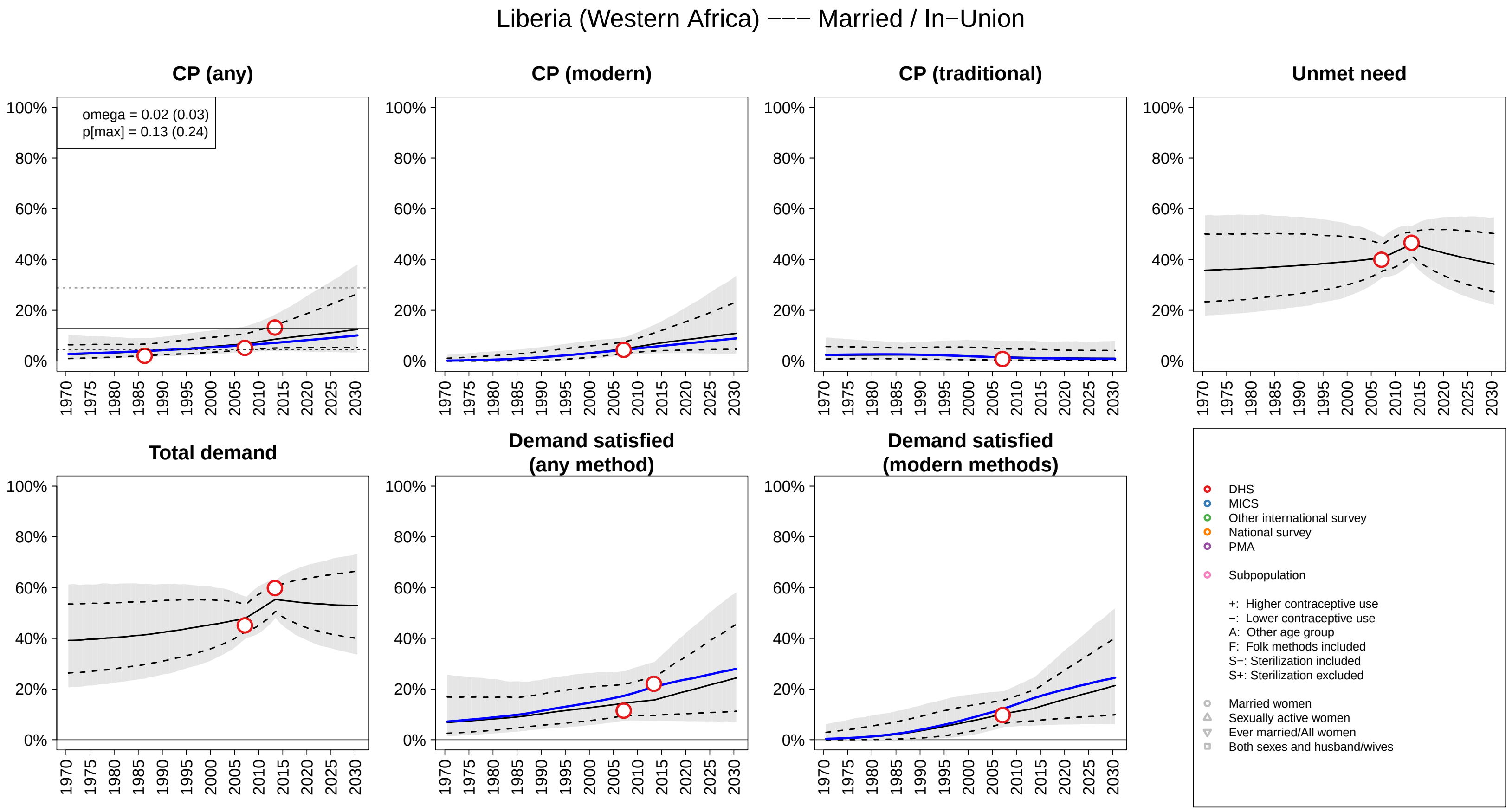
Liberia (Western Africa) --- Married / In−Union
CP (any)
omega = 0.02 (0.03)
p[max] = 0.13 (0.24)
CP (modern)
CP (traditional)
Unmet need
Total demand
Demand satisfied (any method)
Demand satisfied (modern methods)
DHS
MICS
Other international survey
National survey
PMA
Subpopulation
+: Higher contraceptive use
−: Lower contraceptive use
A: Other age group
F: Folk methods included
S−: Sterilization included
S+: Sterilization excluded
Married women
Sexually active women
Ever married/All women
Both sexes and husband/wives

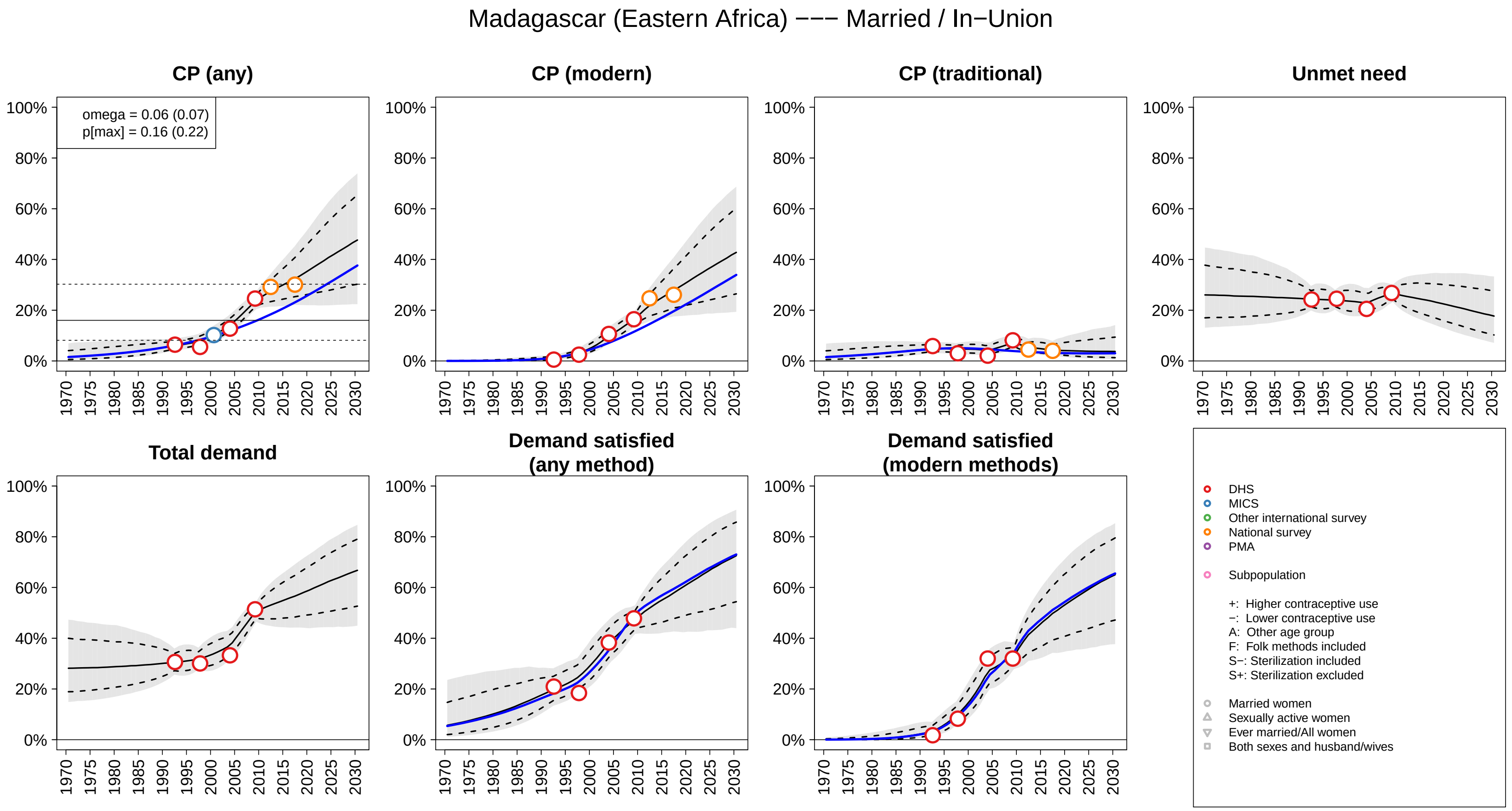
Madagascar (Eastern Africa) --- Married / In−Union
CP (any)
omega = 0.06 (0.07)
p[max] = 0.16 (0.22)
CP (modern)
CP (traditional)
Unmet need
Total demand
Demand satisfied (any method)
Demand satisfied (modern methods)
DHS
MICS
Other international survey
National survey
PMA
Subpopulation
+: Higher contraceptive use
−: Lower contraceptive use
A: Other age group
F: Folk methods included
S−: Sterilization included
S+: Sterilization excluded
Married women
Sexually active women
Ever married/All women
Both sexes and husband/wives

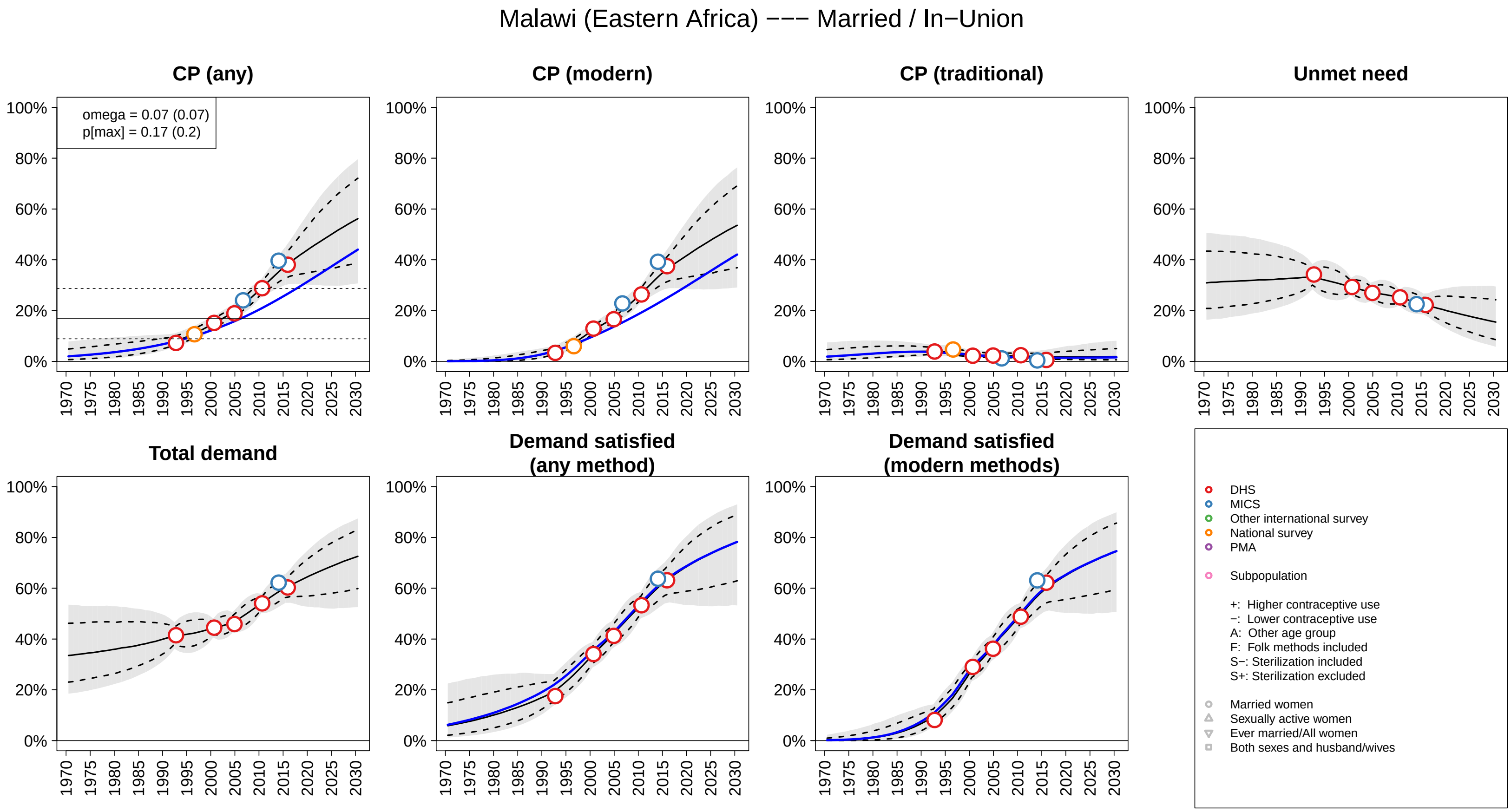
Malawi (Eastern Africa) −−− Married / In−Union
CP (any)
omega = 0.07 (0.07)
p[max] = 0.17 (0.2)
CP (modern)
CP (traditional)
Unmet need
Total demand
Demand satisfied (any method)
Demand satisfied (modern methods)
0%
20%
40%
60%
80%
100%
1970
1975
1980
1985
1990
1995
2000
2005
2010
2015
2020
2025
2030
DHS
MICS
Other international survey
National survey
PMA
Subpopulation
+: Higher contraceptive use
−: Lower contraceptive use
A: Other age group
F: Folk methods included
S−: Sterilization included
S+: Sterilization excluded
Married women
Sexually active women
Ever married/All women
Both sexes and husband/wives

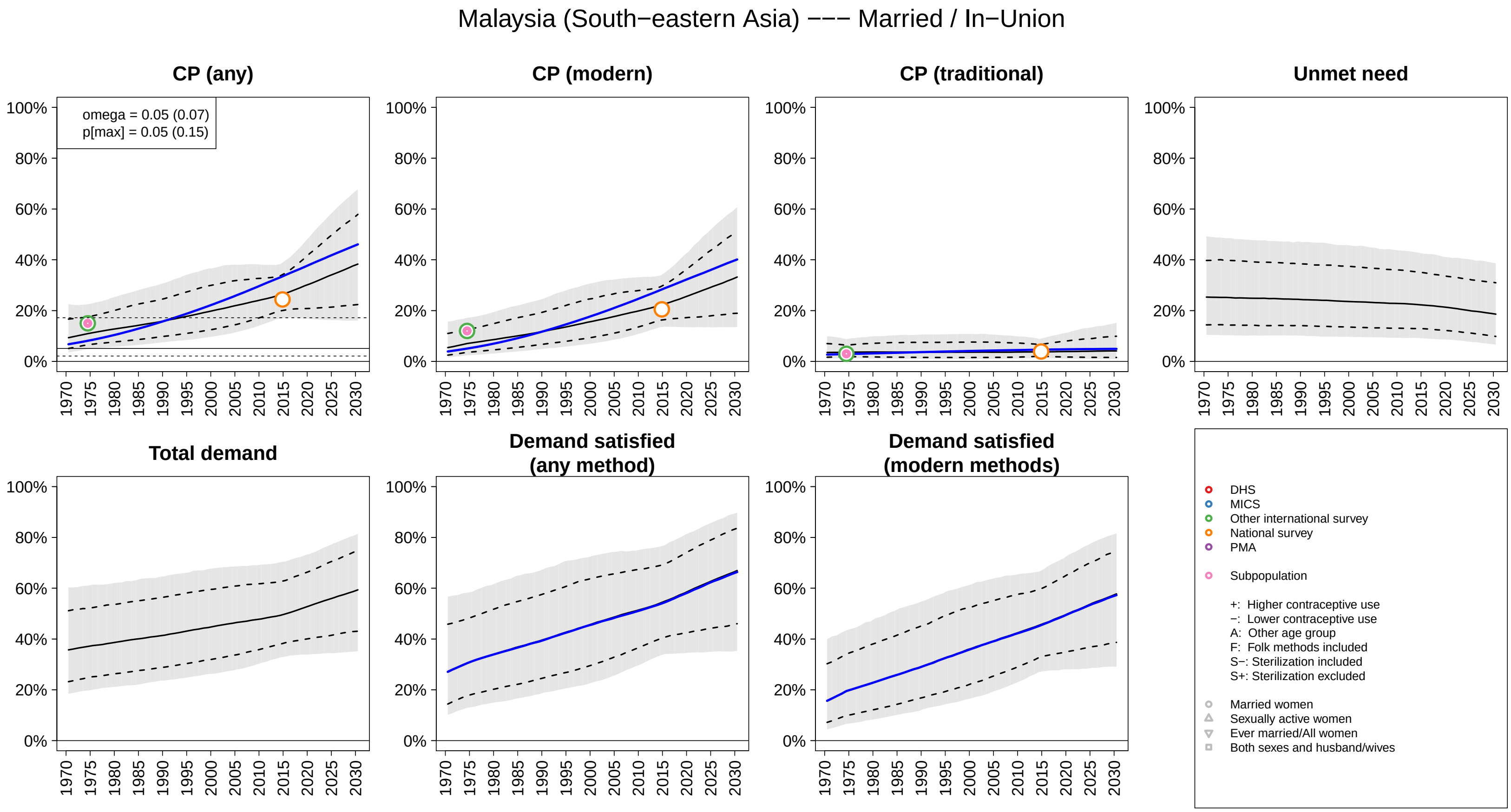
Malaysia (South−eastern Asia) −−− Married / In−Union
CP (any)
omega = 0.05 (0.07)
p[max] = 0.05 (0.15)
CP (modern)
CP (traditional)
Unmet need
Total demand
Demand satisfied (any method)
Demand satisfied (modern methods)
DHS
MICS
Other international survey
National survey
PMA
Subpopulation
+: Higher contraceptive use
−: Lower contraceptive use
A: Other age group
F: Folk methods included
S−: Sterilization included
S+: Sterilization excluded
Married women
Sexually active women
Ever married/All women
Both sexes and husband/wives

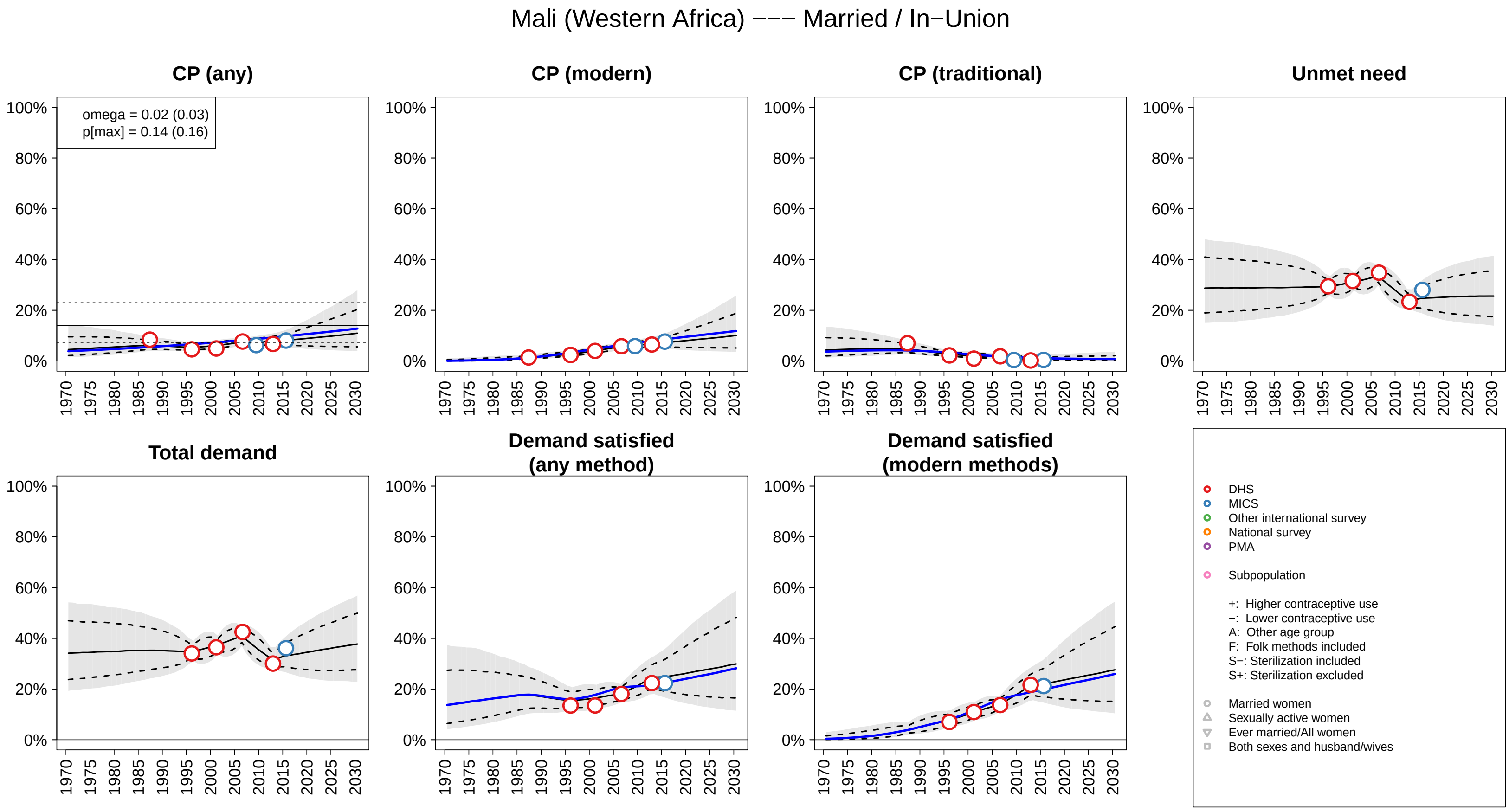
Mali (Western Africa) ––– Married / In–Union
CP (any)
omega = 0.02 (0.03)
p[max] = 0.14 (0.16)
CP (modern)
CP (traditional)
Unmet need
Total demand
Demand satisfied (any method)
Demand satisfied (modern methods)
0%
20%
40%
60%
80%
100%
1970
1975
1980
1985
1990
1995
2000
2005
2010
2015
2020
2025
2030
DHS
MICS
Other international survey
National survey
PMA
Subpopulation
+: Higher contraceptive use
−: Lower contraceptive use
A: Other age group
F: Folk methods included
S−: Sterilization included
S+: Sterilization excluded
Married women
Sexually active women
Ever married/All women
Both sexes and husband/wives

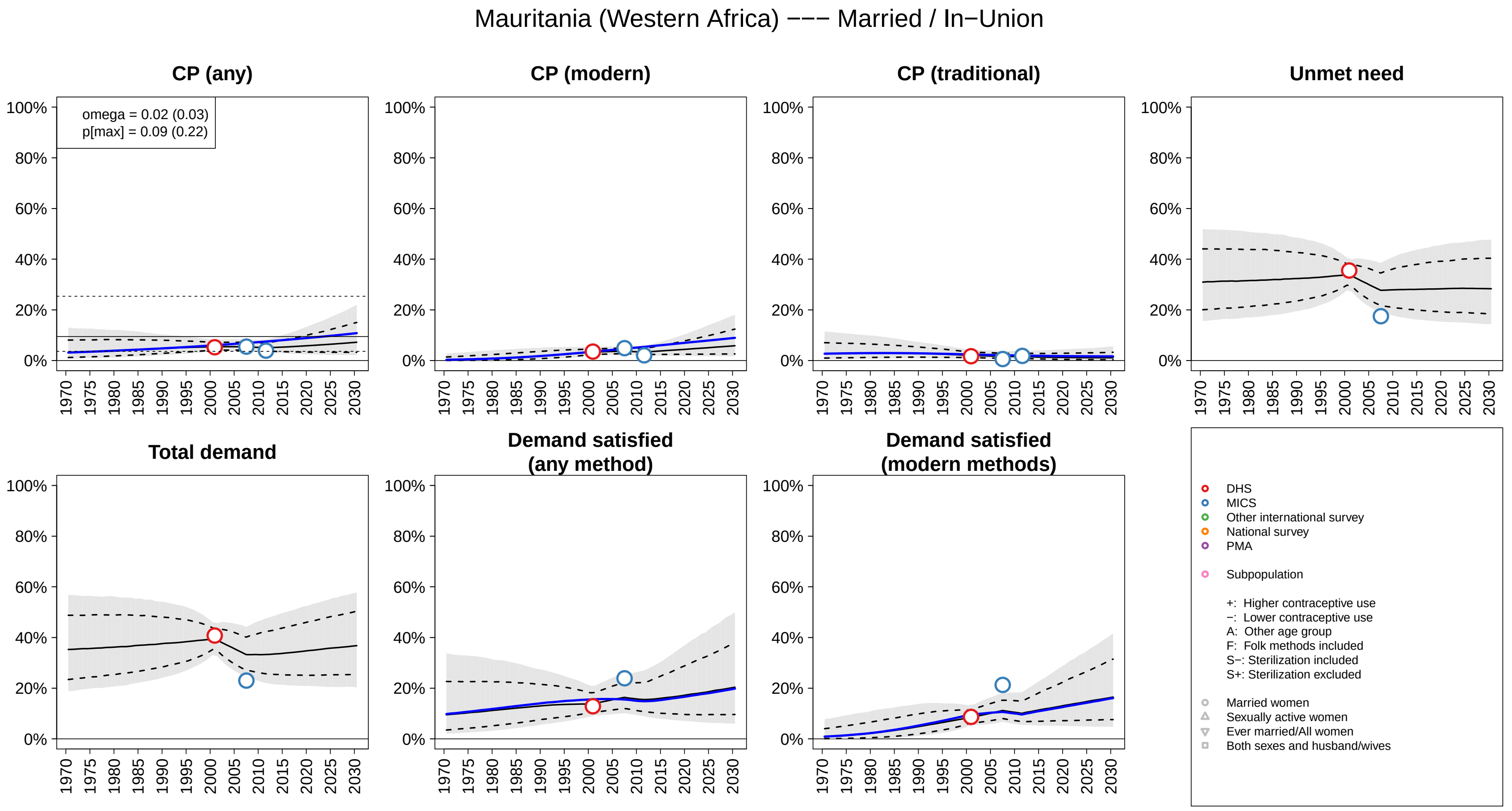
Mauritania (Western Africa) --- Married / In−Union
CP (any)
omega = 0.02 (0.03)
p[max] = 0.09 (0.22)
CP (modern)
CP (traditional)
Unmet need
Total demand
Demand satisfied (any method)
Demand satisfied (modern methods)
DHS
MICS
Other international survey
National survey
PMA
Subpopulation
+: Higher contraceptive use
−: Lower contraceptive use
A: Other age group
F: Folk methods included
S−: Sterilization included
S+: Sterilization excluded
Married women
Sexually active women
Ever married/All women
Both sexes and husband/wives

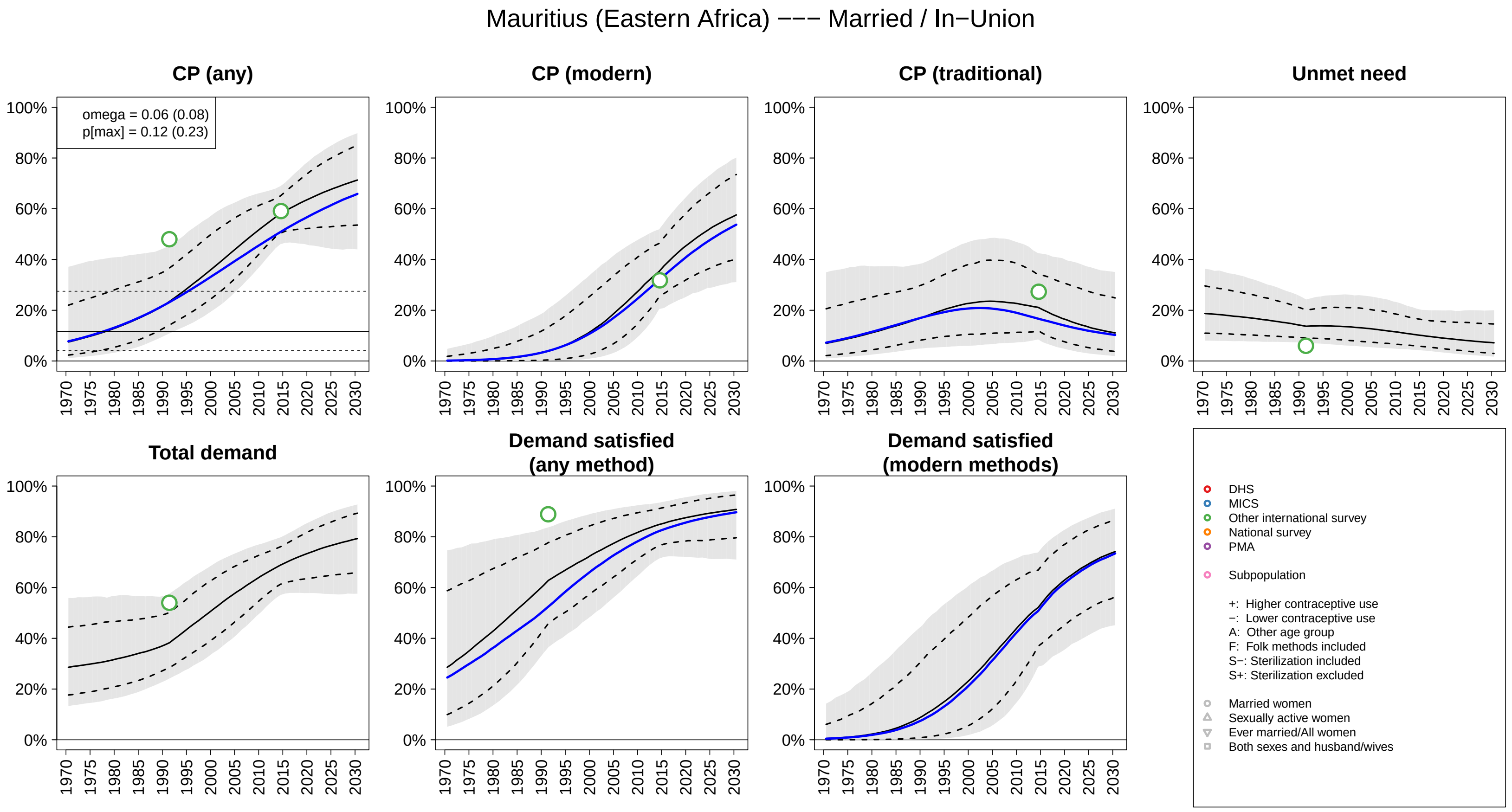
Mauritius (Eastern Africa) --- Married / In−Union
CP (any)
omega = 0.06 (0.08)
p[max] = 0.12 (0.23)
CP (modern)
CP (traditional)
Unmet need
Total demand
Demand satisfied (any method)
Demand satisfied (modern methods)
DHS
MICS
Other international survey
National survey
PMA
Subpopulation
+: Higher contraceptive use
−: Lower contraceptive use
A: Other age group
F: Folk methods included
S−: Sterilization included
S+: Sterilization excluded
Married women
Sexually active women
Ever married/All women
Both sexes and husband/wives

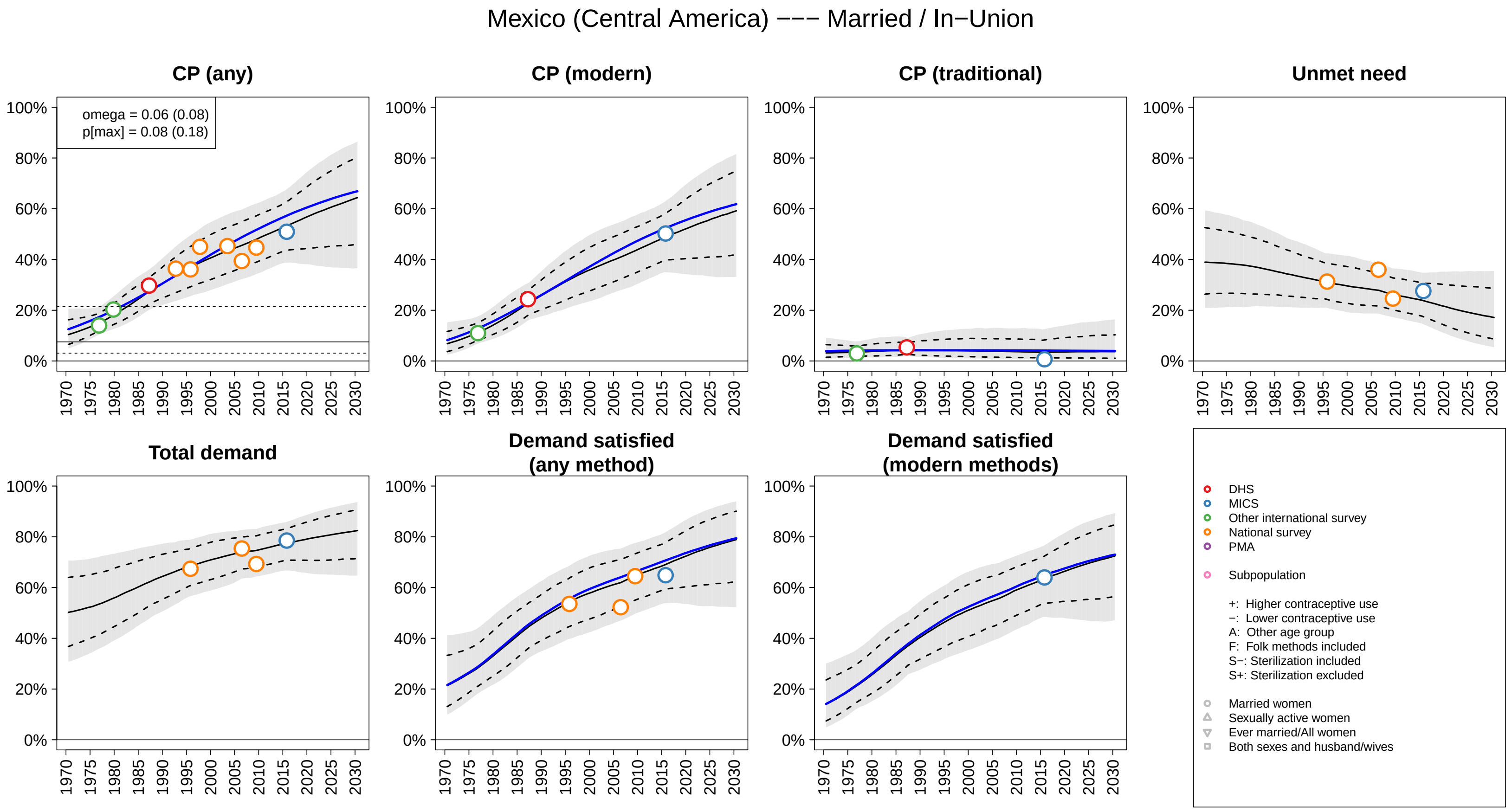
Mexico (Central America) --- Married / In−Union
CP (any)
omega = 0.06 (0.08)
p[max] = 0.08 (0.18)
CP (modern)
CP (traditional)
Unmet need
Total demand
Demand satisfied (any method)
Demand satisfied (modern methods)
DHS
MICS
Other international survey
National survey
PMA
Subpopulation
+: Higher contraceptive use
−: Lower contraceptive use
A: Other age group
F: Folk methods included
S−: Sterilization included
S+: Sterilization excluded
Married women
Sexually active women
Ever married/All women
Both sexes and husband/wives

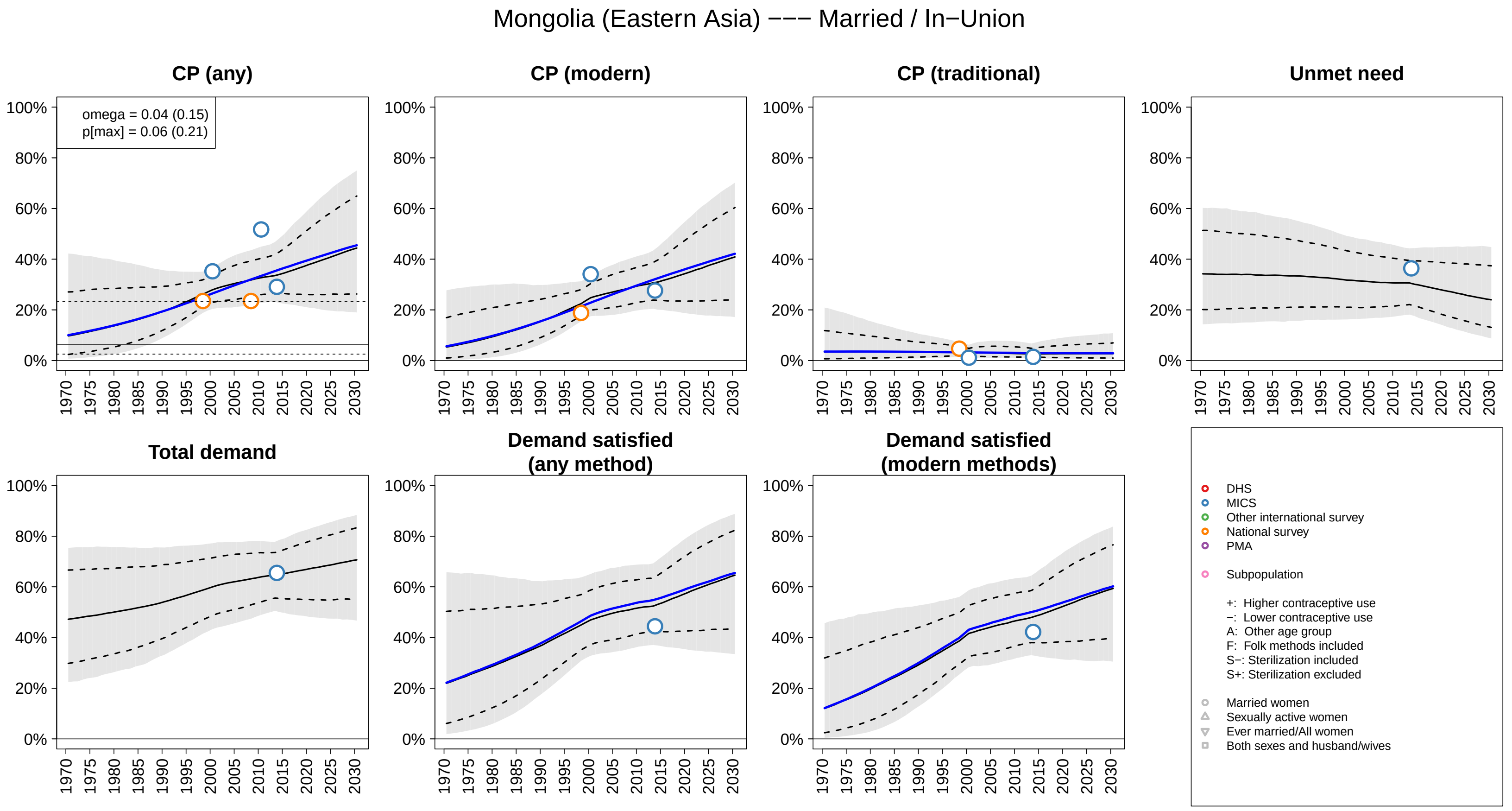
Mongolia (Eastern Asia) −−− Married / In−Union
CP (any)
omega = 0.04 (0.15)
p[max] = 0.06 (0.21)
CP (modern)
CP (traditional)
Unmet need
Total demand
Demand satisfied (any method)
Demand satisfied (modern methods)
DHS
MICS
Other international survey
National survey
PMA
Subpopulation
+: Higher contraceptive use
−: Lower contraceptive use
A: Other age group
F: Folk methods included
S−: Sterilization included
S+: Sterilization excluded
Married women
Sexually active women
Ever married/All women
Both sexes and husband/wives

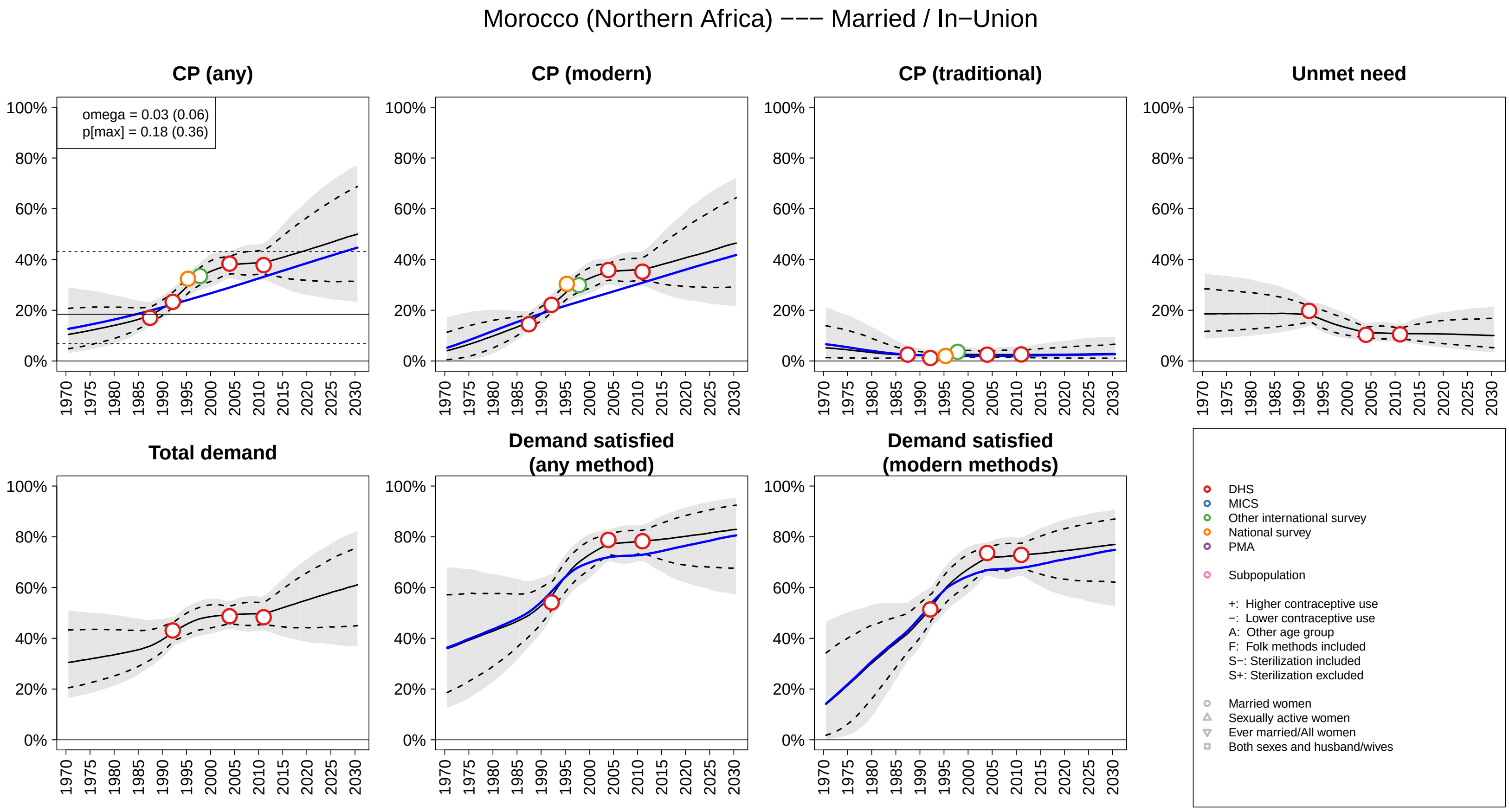
Morocco (Northern Africa) −−− Married / In−Union
CP (any)
omega = 0.03 (0.06)
p[max] = 0.18 (0.36)
CP (modern)
CP (traditional)
Unmet need
Total demand
Demand satisfied (any method)
Demand satisfied (modern methods)
DHS
MICS
Other international survey
National survey
PMA
Subpopulation
+: Higher contraceptive use
−: Lower contraceptive use
A: Other age group
F: Folk methods included
S−: Sterilization included
S+: Sterilization excluded
Married women
Sexually active women
Ever married/All women
Both sexes and husband/wives

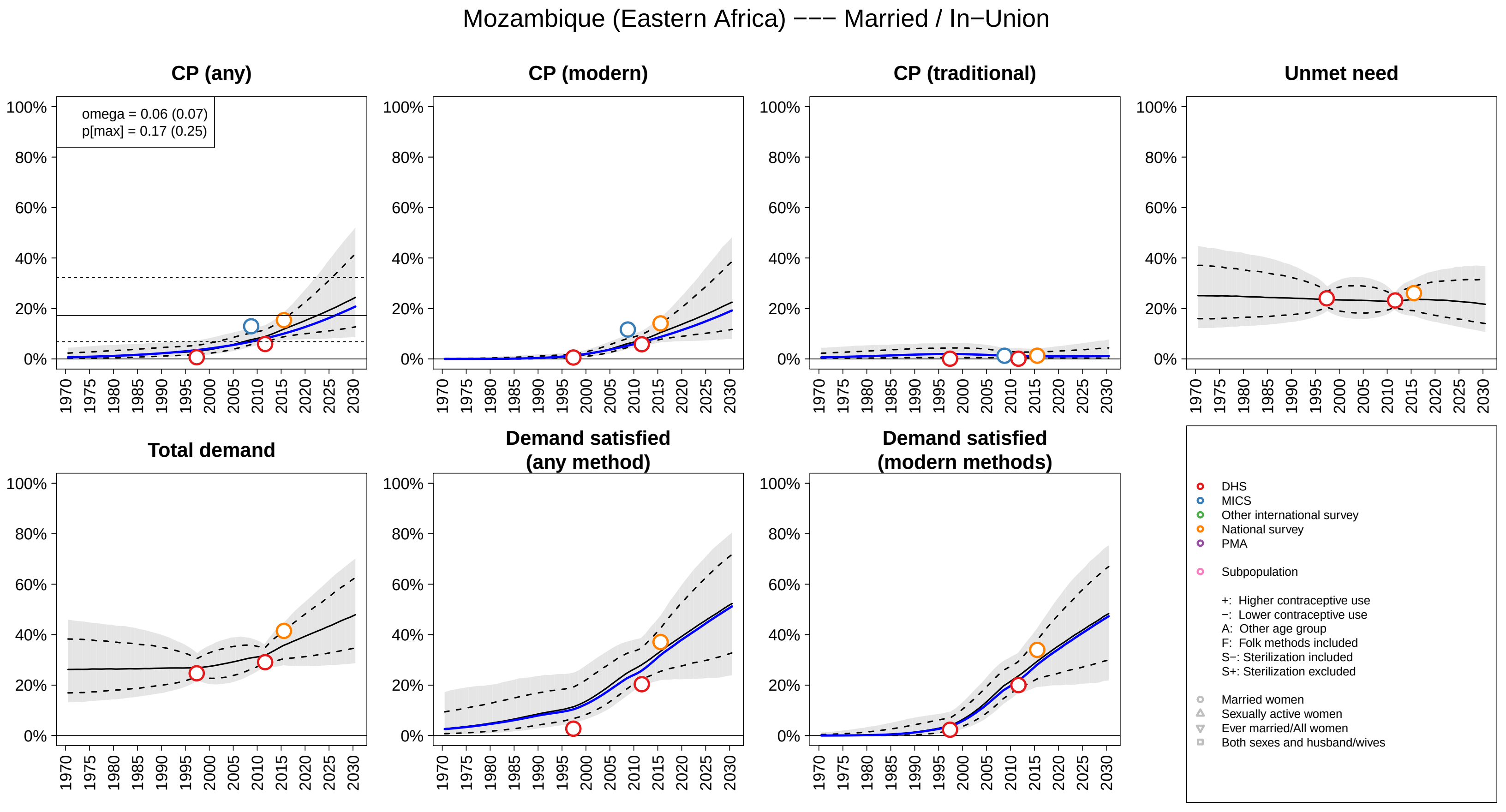

Mozambique (Eastern Africa) --- Married / In−Union
CP (any)
omega = 0.06 (0.07)
p[max] = 0.17 (0.25)
CP (modern)
CP (traditional)
Unmet need
Total demand
Demand satisfied (any method)
Demand satisfied (modern methods)
0%
20%
40%
60%
80%
100%
1970
1975
1980
1985
1990
1995
2000
2005
2010
2015
2020
2025
2030
DHS
MICS
Other international survey
National survey
PMA
Subpopulation
+: Higher contraceptive use
−: Lower contraceptive use
A: Other age group
F: Folk methods included
S−: Sterilization included
S+: Sterilization excluded
Married women
Sexually active women
Ever married/All women
Both sexes and husband/wives

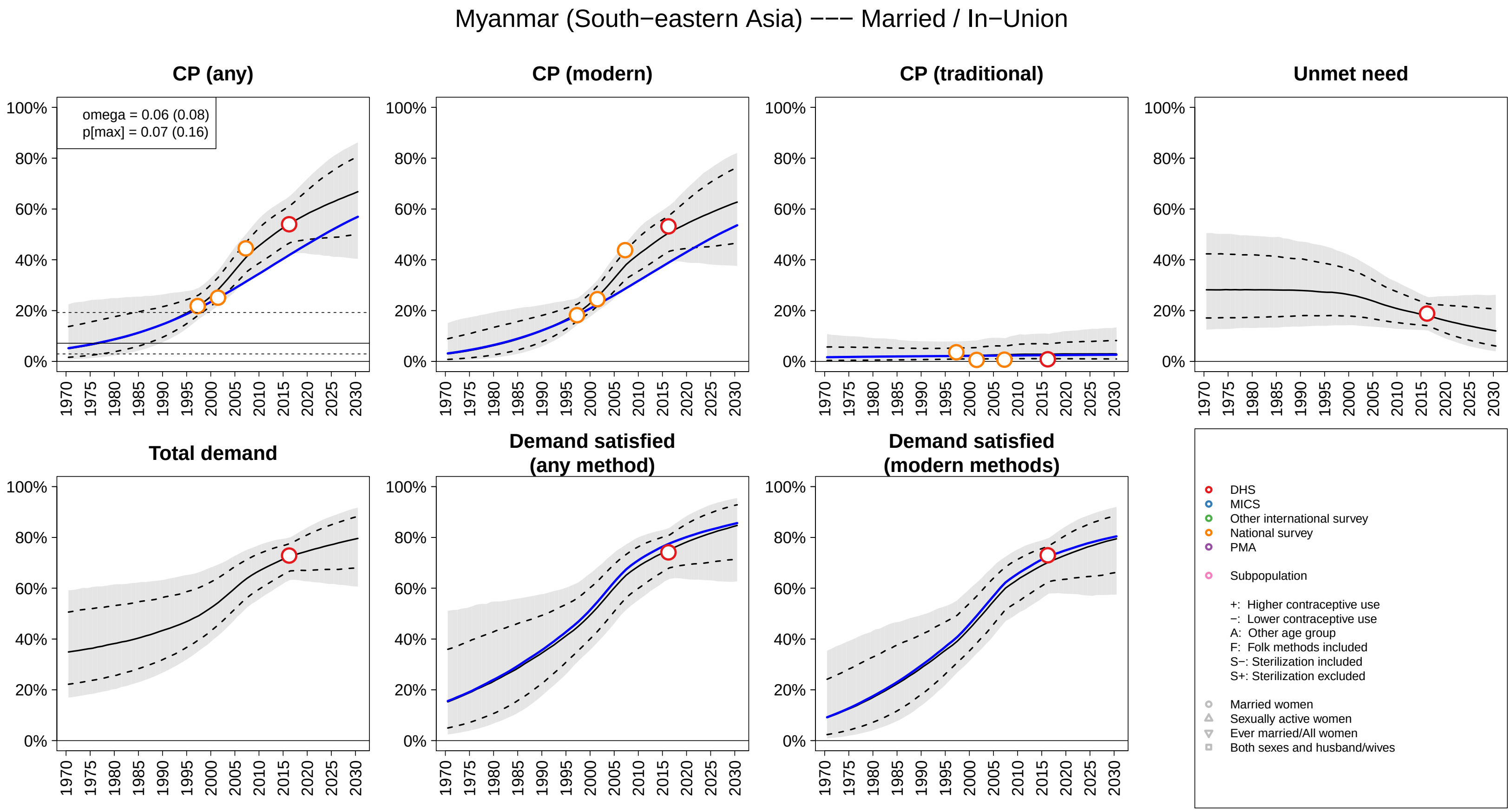
Myanmar (South−eastern Asia) −−− Married / In−Union
CP (any)
omega = 0.06 (0.08)
p[max] = 0.07 (0.16)
CP (modern)
CP (traditional)
Unmet need
Total demand
Demand satisfied (any method)
Demand satisfied (modern methods)
100%
80%
60%
40%
20%
0%
1970 1975 1980 1985 1990 1995 2000 2005 2010 2015 2020 2025 2030
DHS
MICS
Other international survey
National survey
PMA
Subpopulation
+: Higher contraceptive use
−: Lower contraceptive use
A: Other age group
F: Folk methods included
S−: Sterilization included
S+: Sterilization excluded
Married women
Sexually active women
Ever married/All women
Both sexes and husband/wives

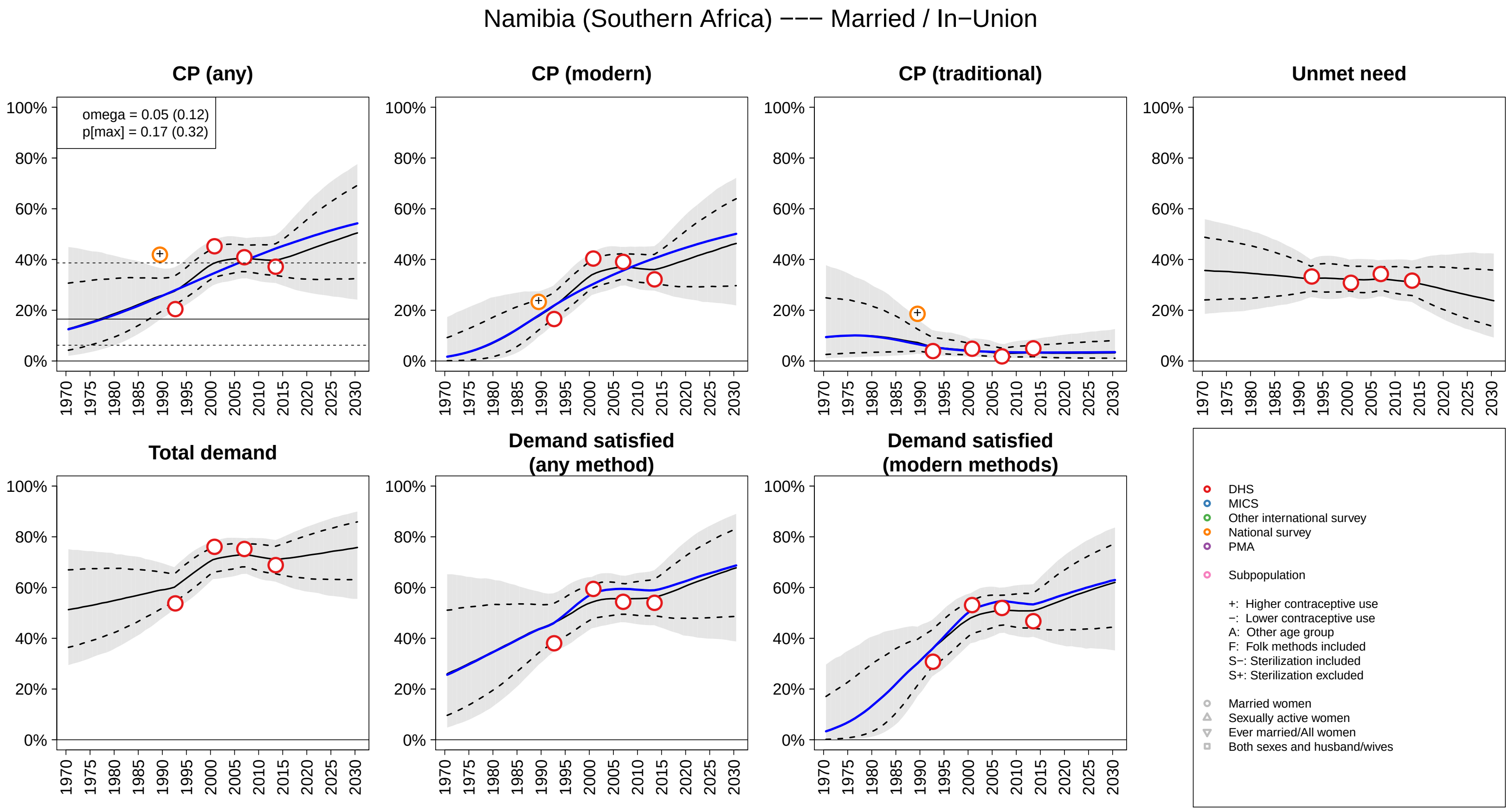

Namibia (Southern Africa) --- Married / In−Union
CP (any)
omega = 0.05 (0.12)
p[max] = 0.17 (0.32)
CP (modern)
CP (traditional)
Unmet need
Total demand
Demand satisfied (any method)
Demand satisfied (modern methods)
DHS
MICS
Other international survey
National survey
PMA
Subpopulation
+: Higher contraceptive use
−: Lower contraceptive use
A: Other age group
F: Folk methods included
S−: Sterilization included
S+: Sterilization excluded
Married women
Sexually active women
Ever married/All women
Both sexes and husband/wives

Nepal (Southern Asia) --- Married / In−Union

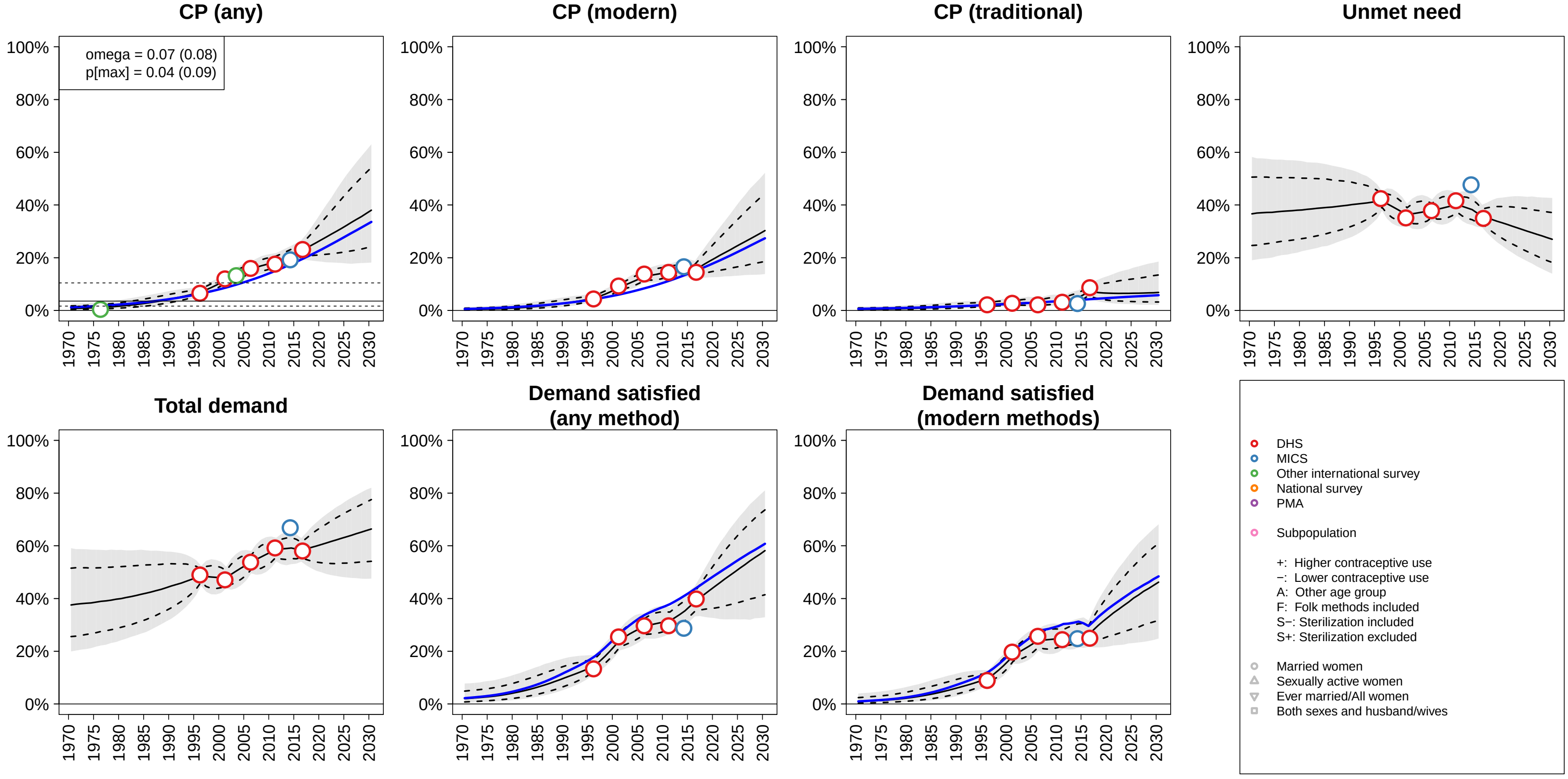

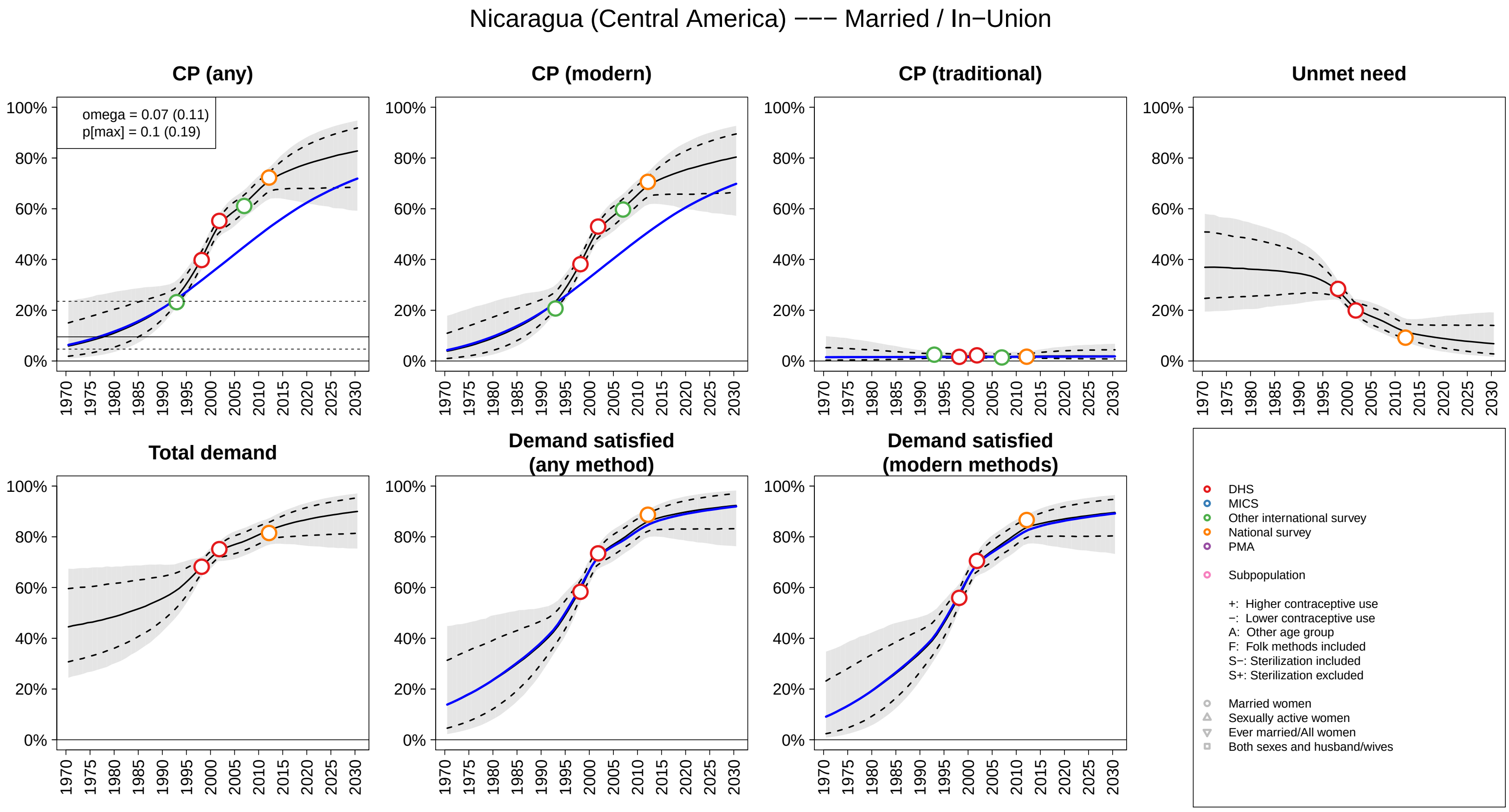
Nicaragua (Central America) ––– Married / In−Union
CP (any)
omega = 0.07 (0.11)
p[max] = 0.1 (0.19)
CP (modern)
CP (traditional)
Unmet need
Total demand
Demand satisfied (any method)
Demand satisfied (modern methods)
DHS
MICS
Other international survey
National survey
PMA
Subpopulation
+: Higher contraceptive use
−: Lower contraceptive use
A: Other age group
F: Folk methods included
S−: Sterilization included
S+: Sterilization excluded
Married women
Sexually active women
Ever married/All women
Both sexes and husband/wives

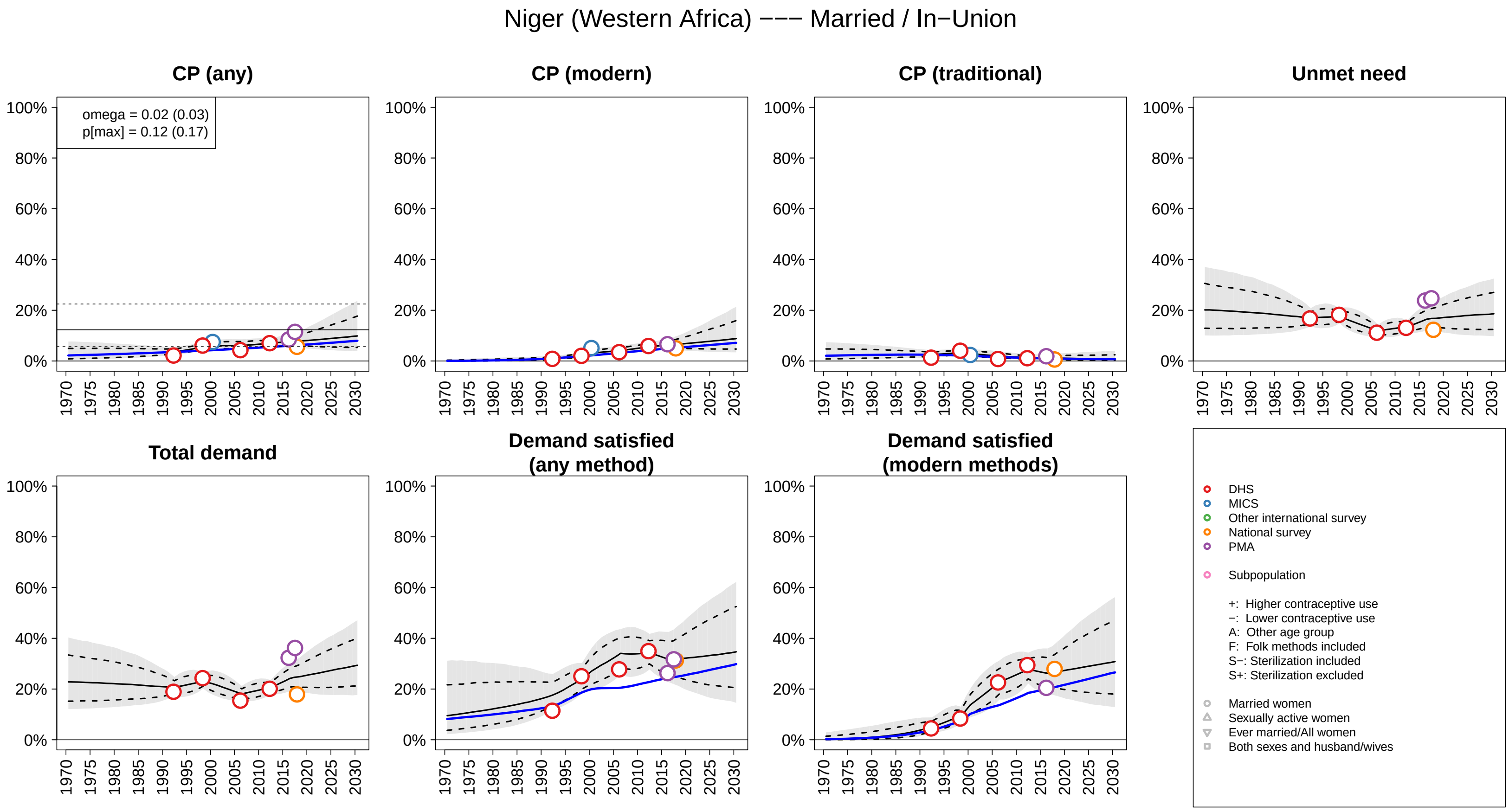

Niger (Western Africa) --- Married / In−Union
CP (any)
omega = 0.02 (0.03)
p[max] = 0.12 (0.17)
CP (modern)
CP (traditional)
Unmet need
Total demand
Demand satisfied (any method)
Demand satisfied (modern methods)
DHS
MICS
Other international survey
National survey
PMA
Subpopulation
+: Higher contraceptive use
−: Lower contraceptive use
A: Other age group
F: Folk methods included
S−: Sterilization included
S+: Sterilization excluded
Married women
Sexually active women
Ever married/All women
Both sexes and husband/wives

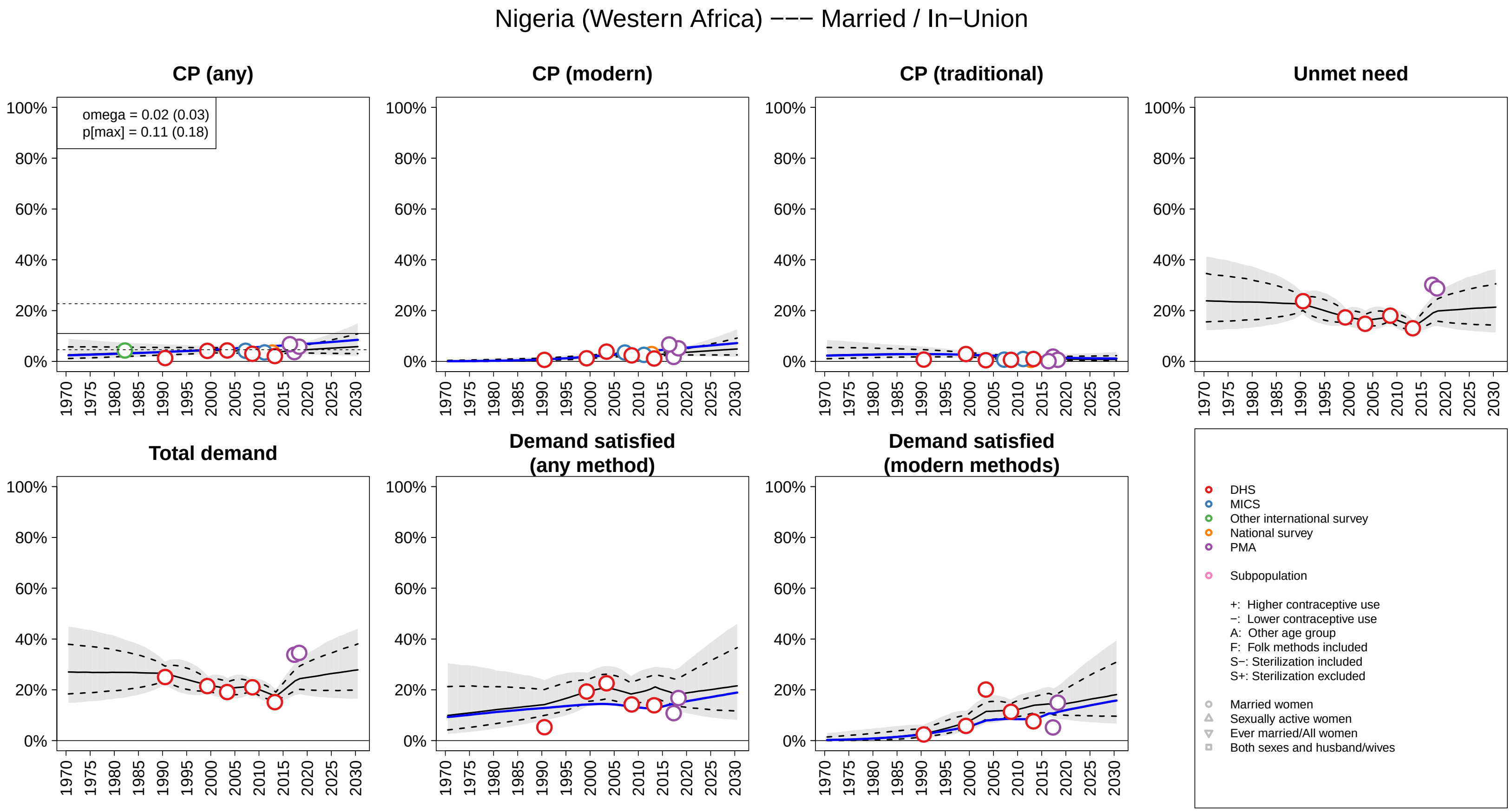
Nigeria (Western Africa) ––– Married / In−Union
CP (any)
omega = 0.02 (0.03)
p[max] = 0.11 (0.18)
CP (modern)
CP (traditional)
Unmet need
Total demand
Demand satisfied
(any method)
Demand satisfied
(modern methods)
DHS
MICS
Other international survey
National survey
PMA
Subpopulation
+: Higher contraceptive use
−: Lower contraceptive use
A: Other age group
F: Folk methods included
S−: Sterilization included
S+: Sterilization excluded
Married women
Sexually active women
Ever married/All women
Both sexes and husband/wives

Oman (Western Asia) --- Married / In−Union

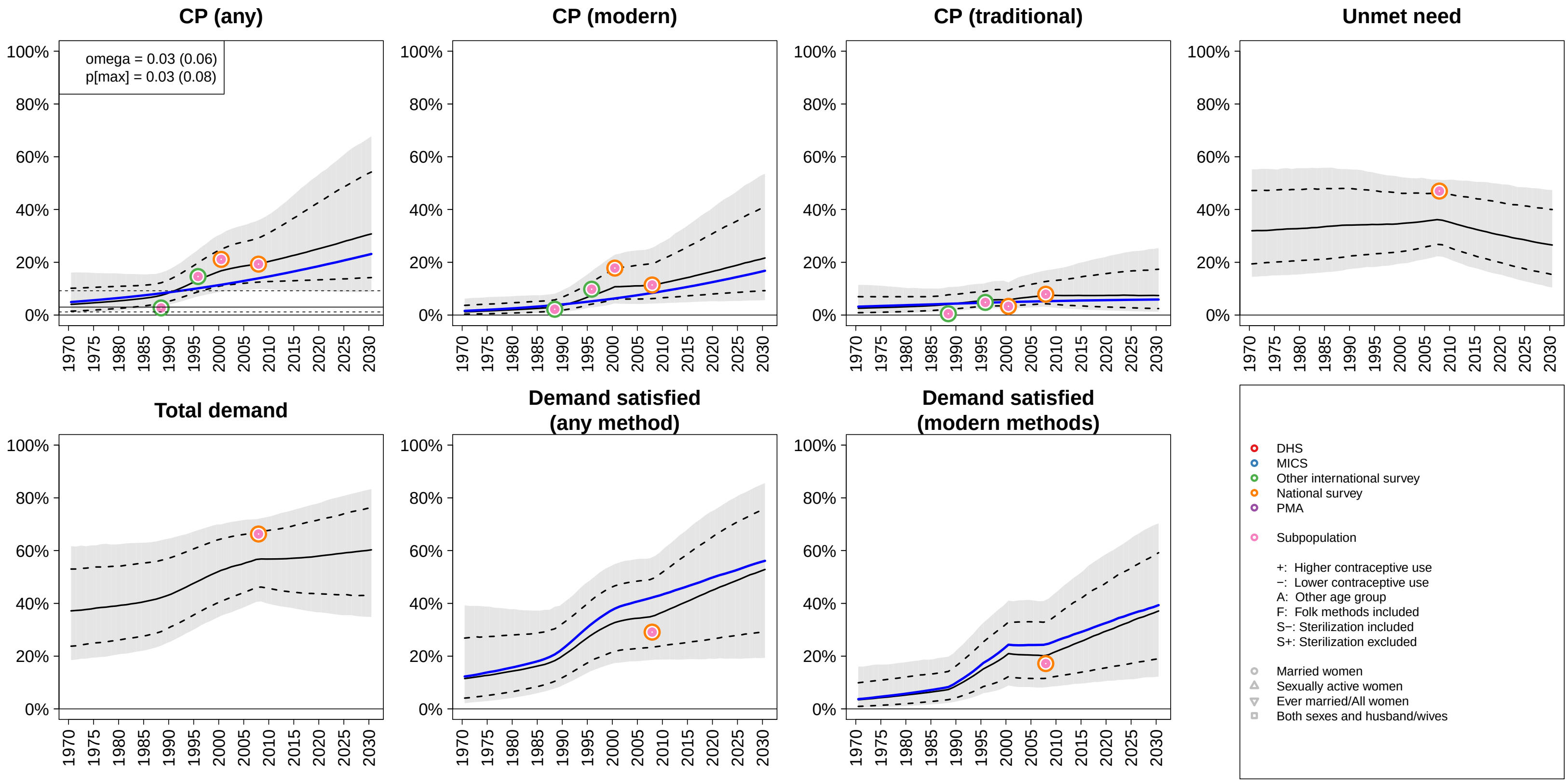

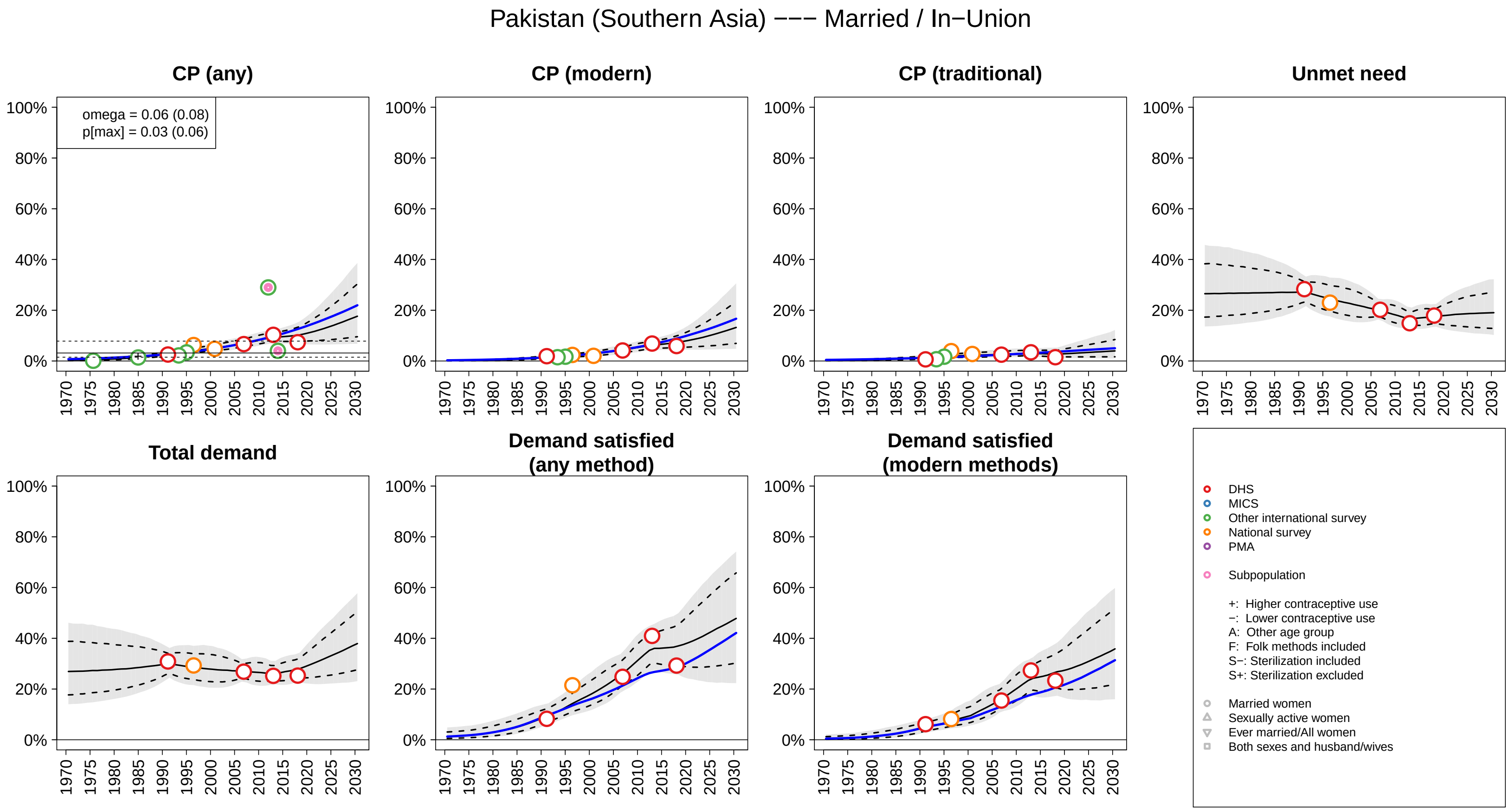

Pakistan (Southern Asia) --- Married / In−Union
CP (any)
omega = 0.06 (0.08)
p[max] = 0.03 (0.06)
CP (modern)
CP (traditional)
Unmet need
Total demand
Demand satisfied (any method)
Demand satisfied (modern methods)
100%
80%
60%
40%
20%
0%
1970 1975 1980 1985 1990 1995 2000 2005 2010 2015 2020 2025 2030
DHS
MICS
Other international survey
National survey
PMA
Subpopulation
+: Higher contraceptive use
−: Lower contraceptive use
A: Other age group
F: Folk methods included
S−: Sterilization included
S+: Sterilization excluded
Married women
Sexually active women
Ever married/All women
Both sexes and husband/wives

Panama (Central America) ––– Married / In−Union

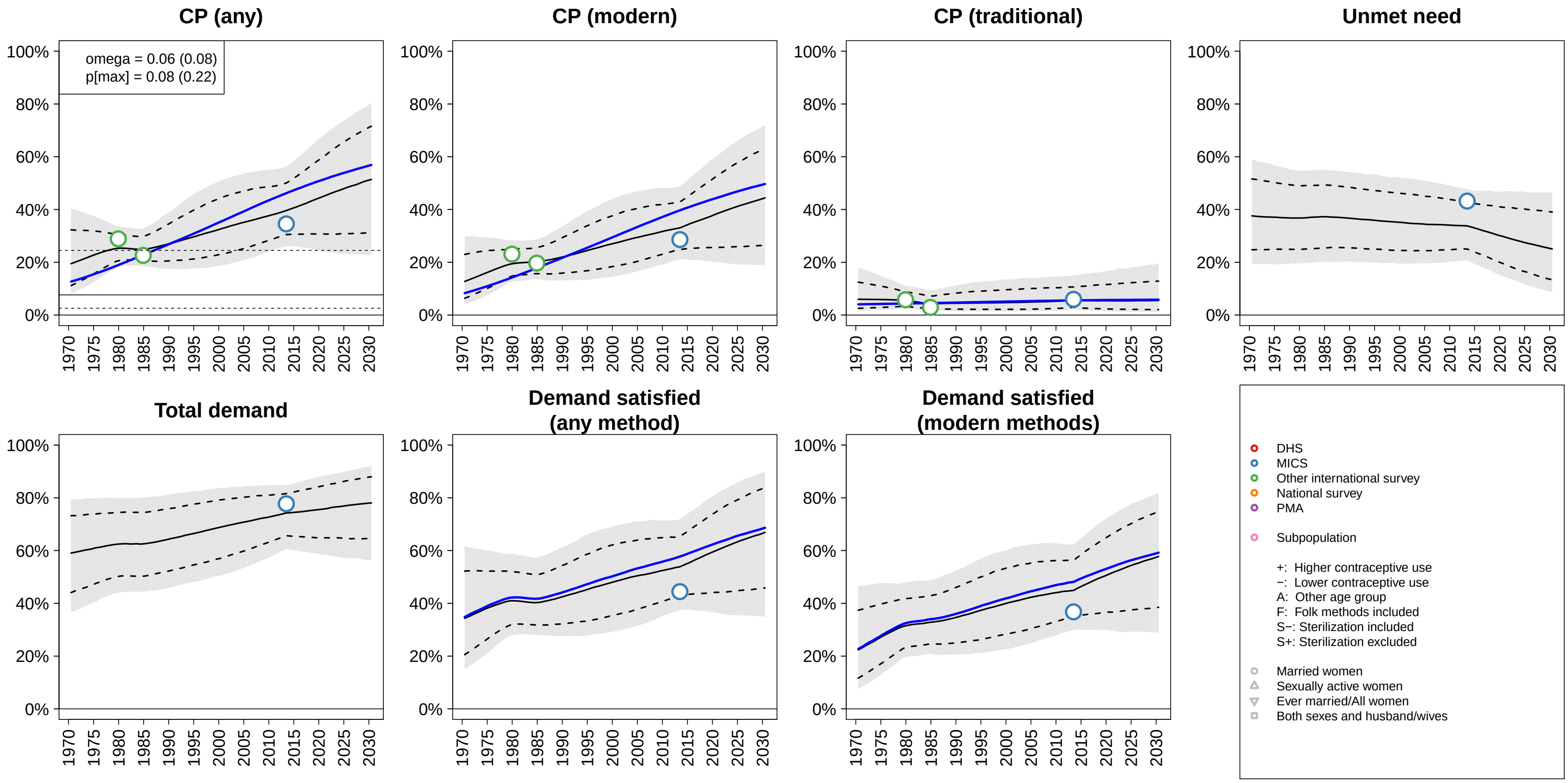

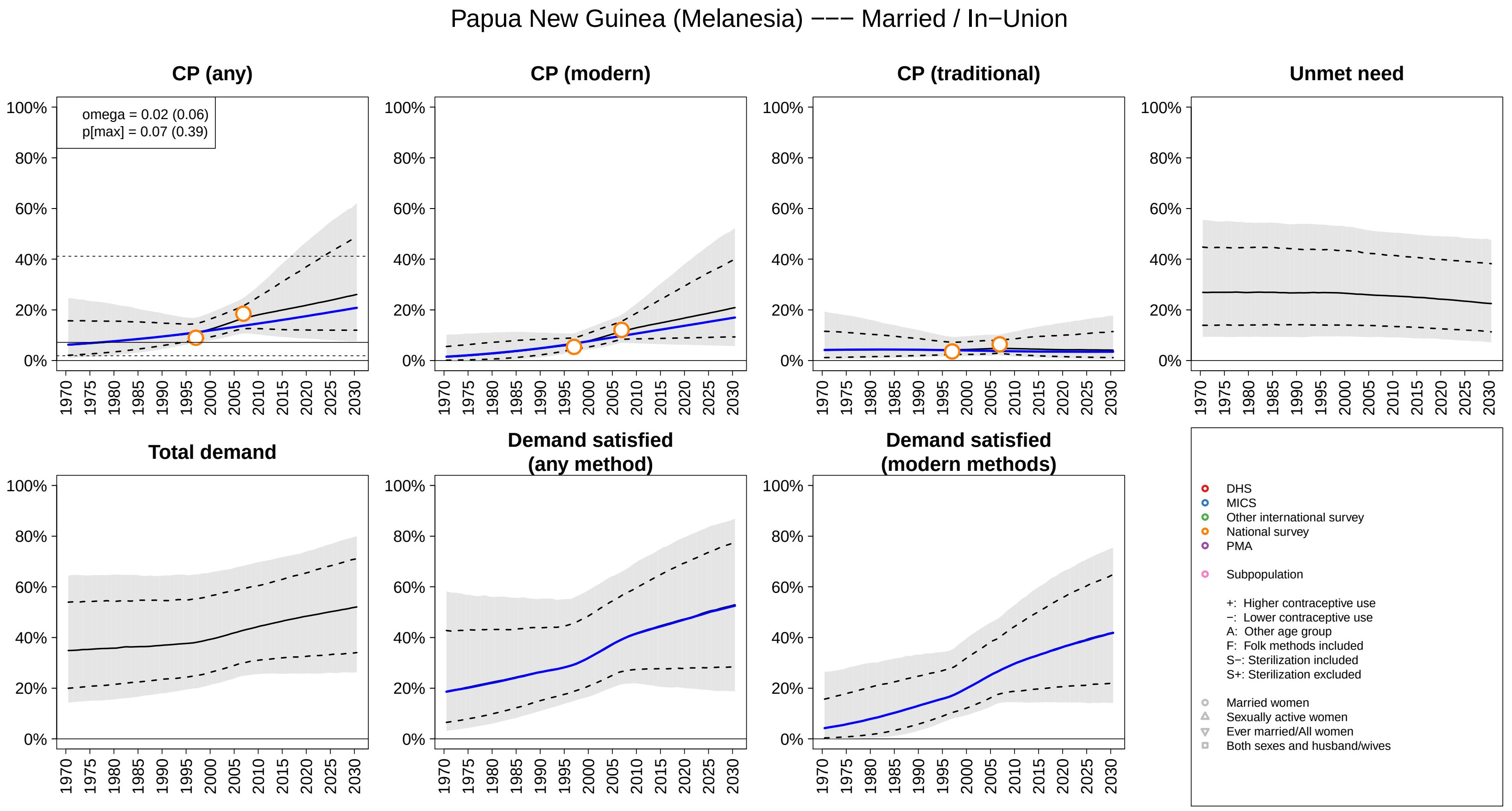
Papua New Guinea (Melanesia) −−− Married / In−Union
CP (any)
omega = 0.02 (0.06)
p[max] = 0.07 (0.39)
CP (modern)
CP (traditional)
Unmet need
Total demand
Demand satisfied (any method)
Demand satisfied (modern methods)
100%
80%
60%
40%
20%
0%
1970 1975 1980 1985 1990 1995 2000 2005 2010 2015 2020 2025 2030
DHS
MICS
Other international survey
National survey
PMA
Subpopulation
+: Higher contraceptive use
−: Lower contraceptive use
A: Other age group
F: Folk methods included
S−: Sterilization included
S+: Sterilization excluded
Married women
Sexually active women
Ever married/All women
Both sexes and husband/wives

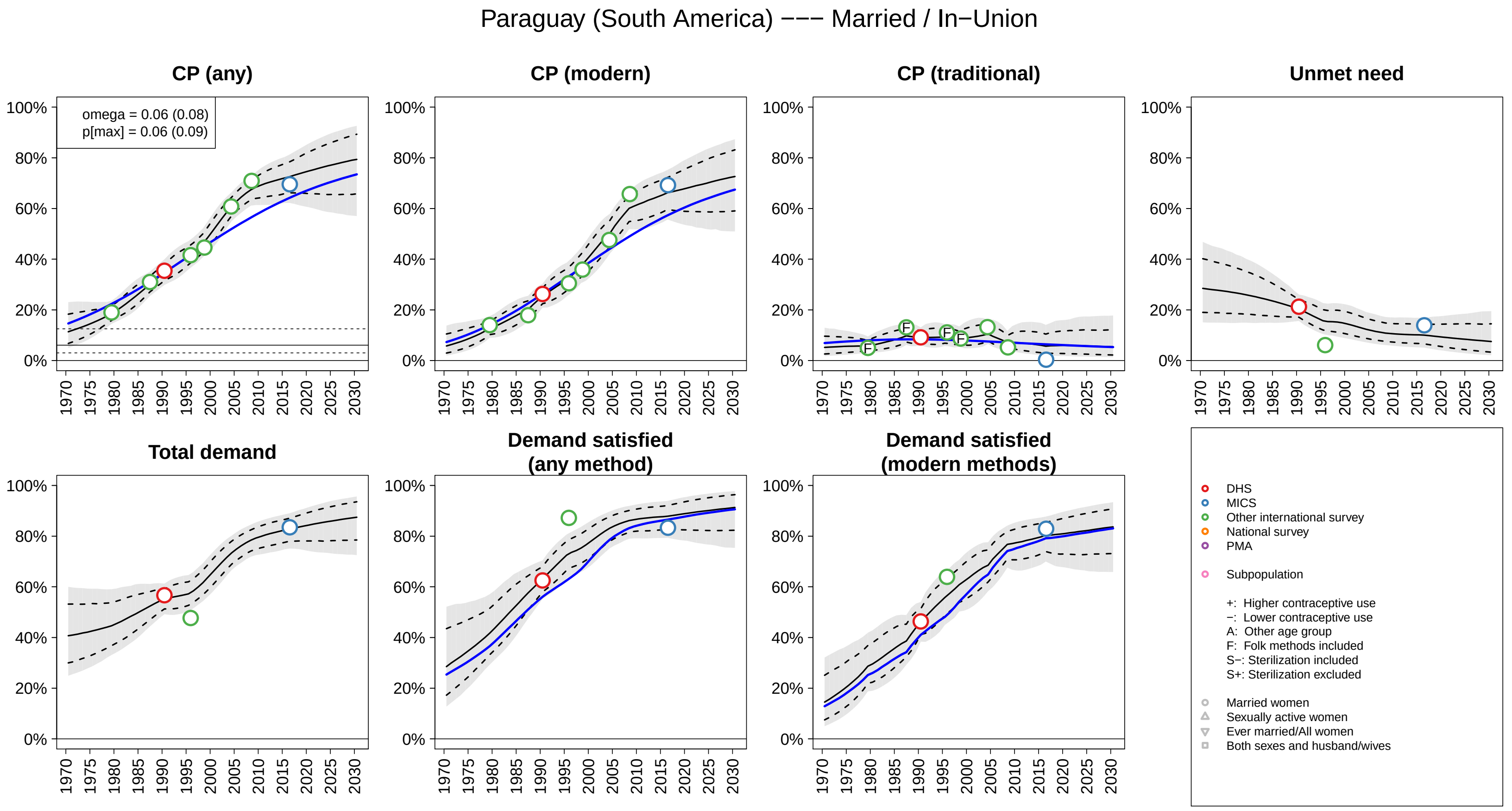
Paraguay (South America) ––– Married / In–Union
CP (any)
omega = 0.06 (0.08)
p[max] = 0.06 (0.09)
CP (modern)
CP (traditional)
Unmet need
Total demand
Demand satisfied (any method)
Demand satisfied (modern methods)
DHS
MICS
Other international survey
National survey
PMA
Subpopulation
+: Higher contraceptive use
−: Lower contraceptive use
A: Other age group
F: Folk methods included
S−: Sterilization included
S+: Sterilization excluded
Married women
Sexually active women
Ever married/All women
Both sexes and husband/wives

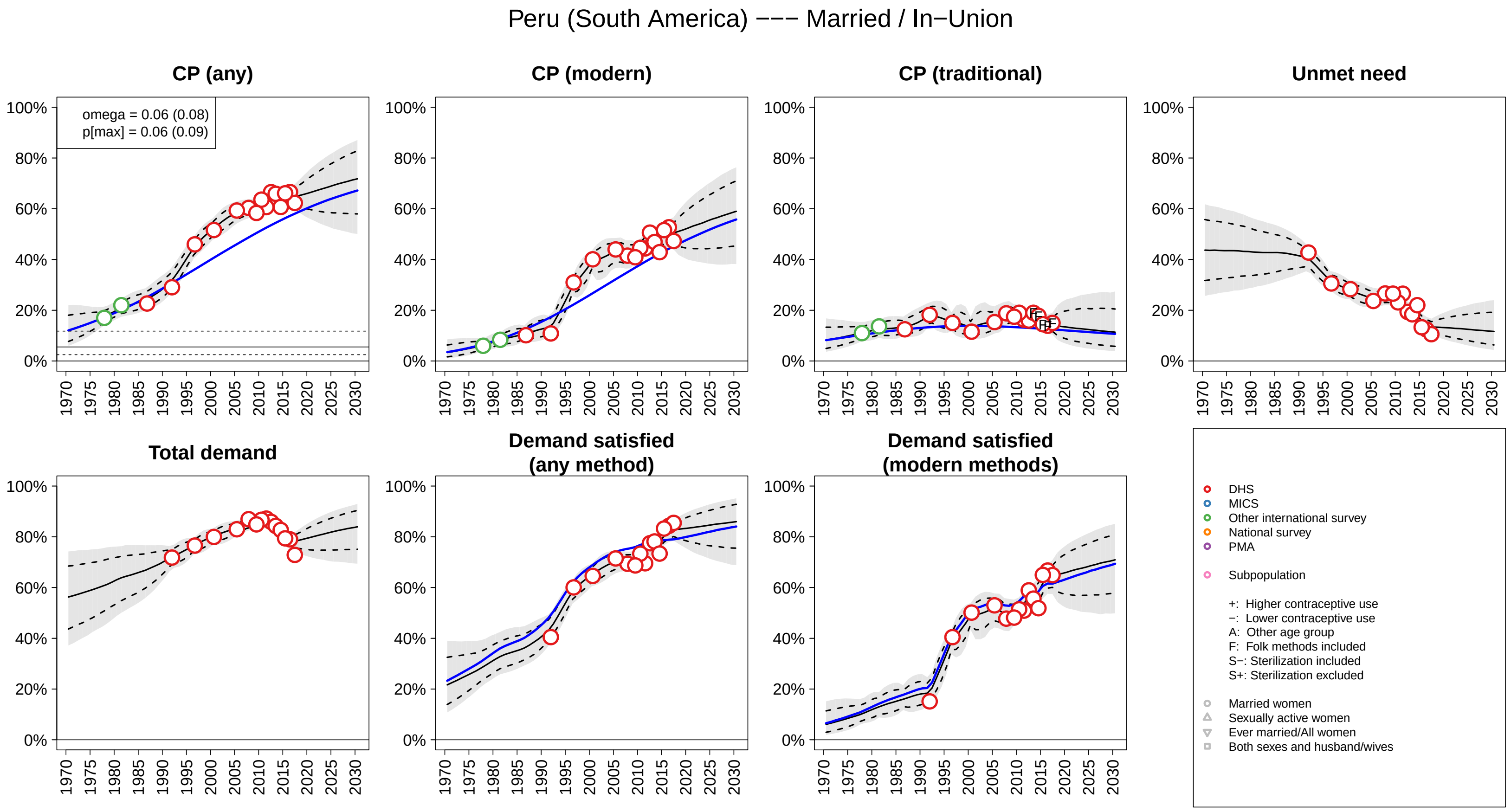

Peru (South America) --- Married / In−Union
CP (any)
omega = 0.06 (0.08)
p[max] = 0.06 (0.09)
CP (modern)
CP (traditional)
Unmet need
Total demand
Demand satisfied (any method)
Demand satisfied (modern methods)
DHS
MICS
Other international survey
National survey
PMA
Subpopulation
+: Higher contraceptive use
−: Lower contraceptive use
A: Other age group
F: Folk methods included
S−: Sterilization included
S+: Sterilization excluded
Married women
Sexually active women
Ever married/All women
Both sexes and husband/wives

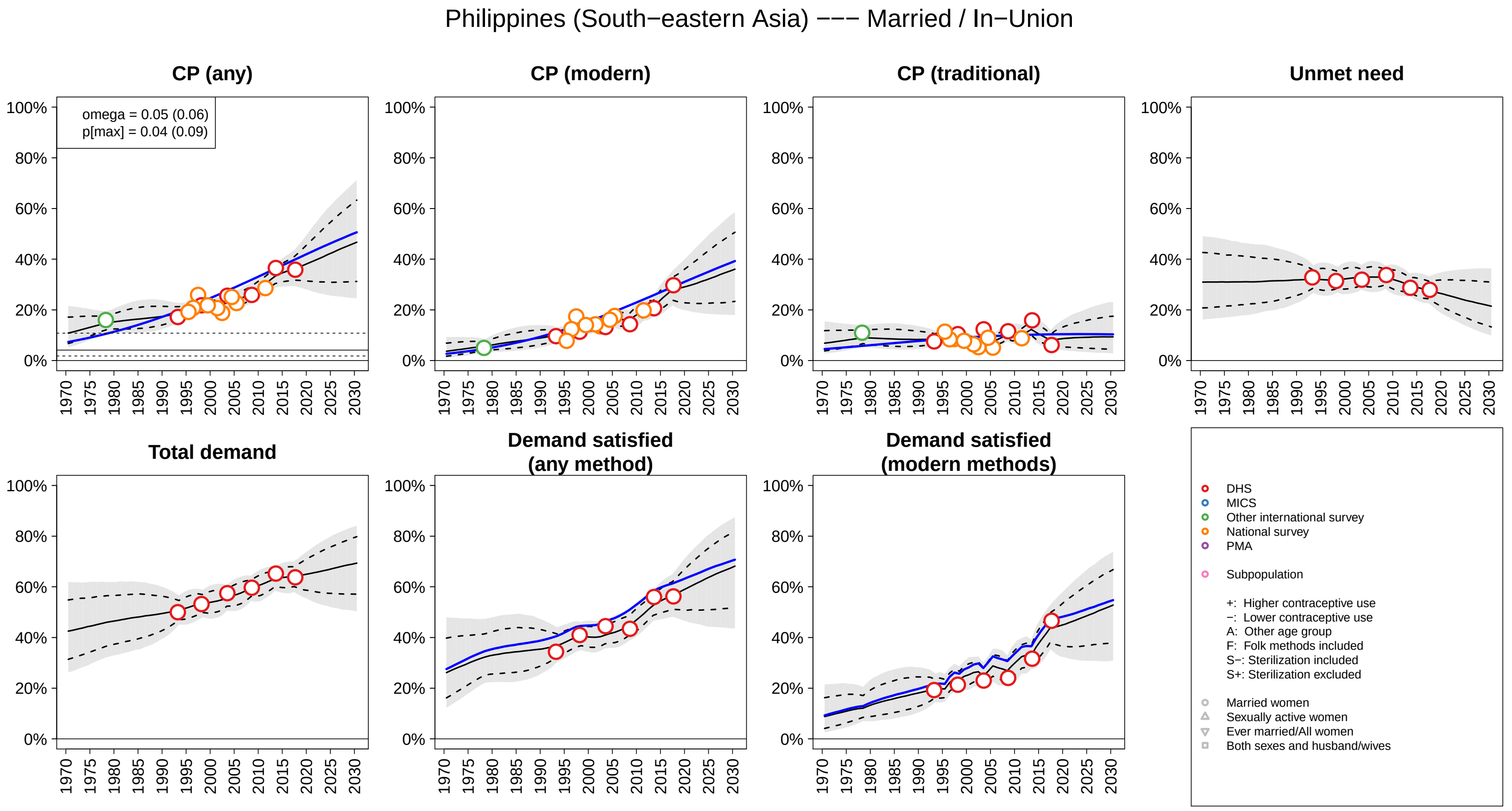
Philippines (South−eastern Asia) −−− Married / In−Union
CP (any)
omega = 0.05 (0.06)
p[max] = 0.04 (0.09)
CP (modern)
CP (traditional)
Unmet need
Total demand
Demand satisfied (any method)
Demand satisfied (modern methods)
DHS
MICS
Other international survey
National survey
PMA
Subpopulation
+: Higher contraceptive use
−: Lower contraceptive use
A: Other age group
F: Folk methods included
S−: Sterilization included
S+: Sterilization excluded
Married women
Sexually active women
Ever married/All women
Both sexes and husband/wives

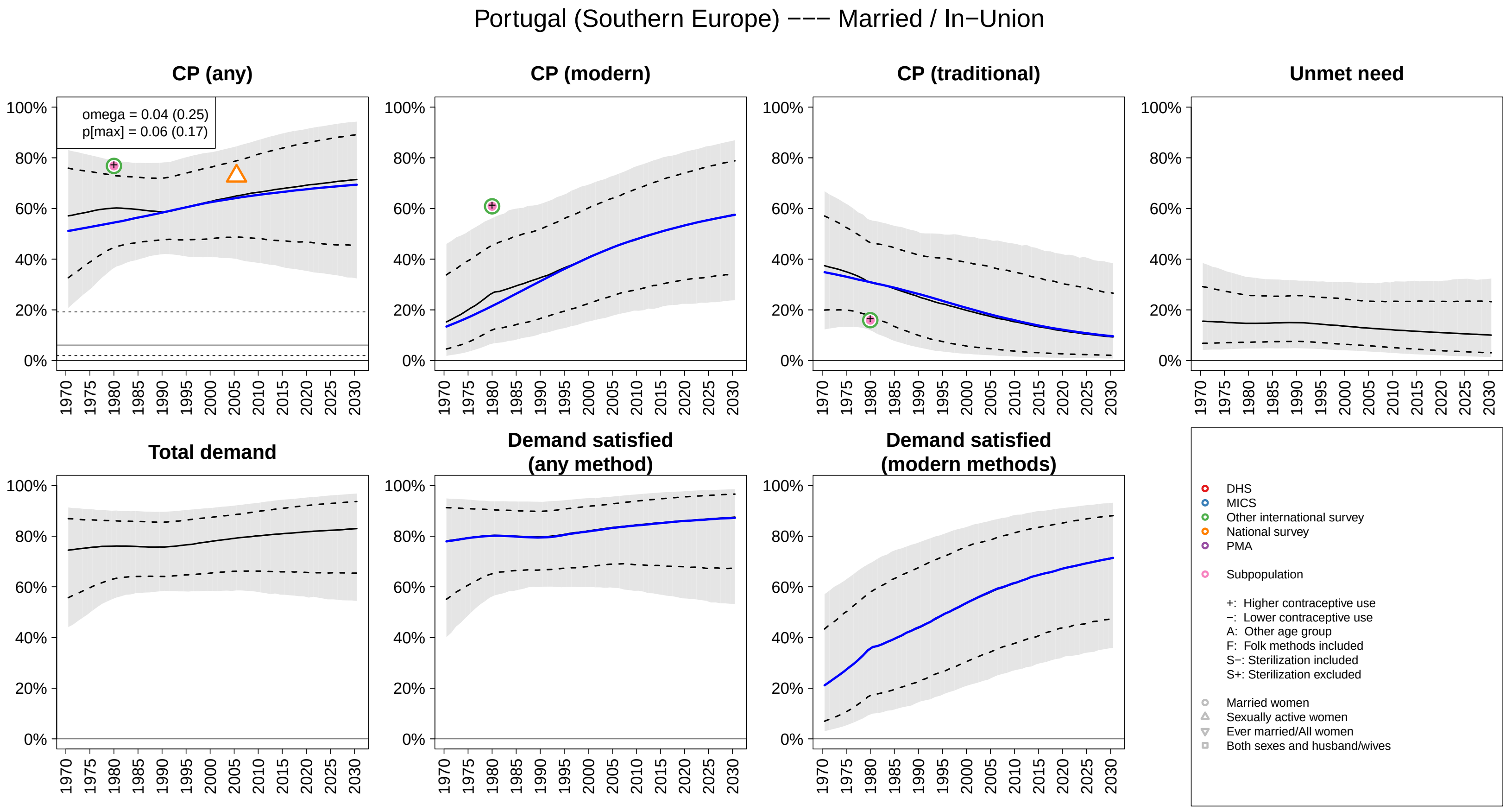
Portugal (Southern Europe) −−− Married / In−Union
CP (any)
omega = 0.04 (0.25)
p[max] = 0.06 (0.17)
CP (modern)
CP (traditional)
Unmet need
Total demand
Demand satisfied (any method)
Demand satisfied (modern methods)
DHS
MICS
Other international survey
National survey
PMA
Subpopulation
+: Higher contraceptive use
−: Lower contraceptive use
A: Other age group
F: Folk methods included
S−: Sterilization included
S+: Sterilization excluded
Married women
Sexually active women
Ever married/All women
Both sexes and husband/wives

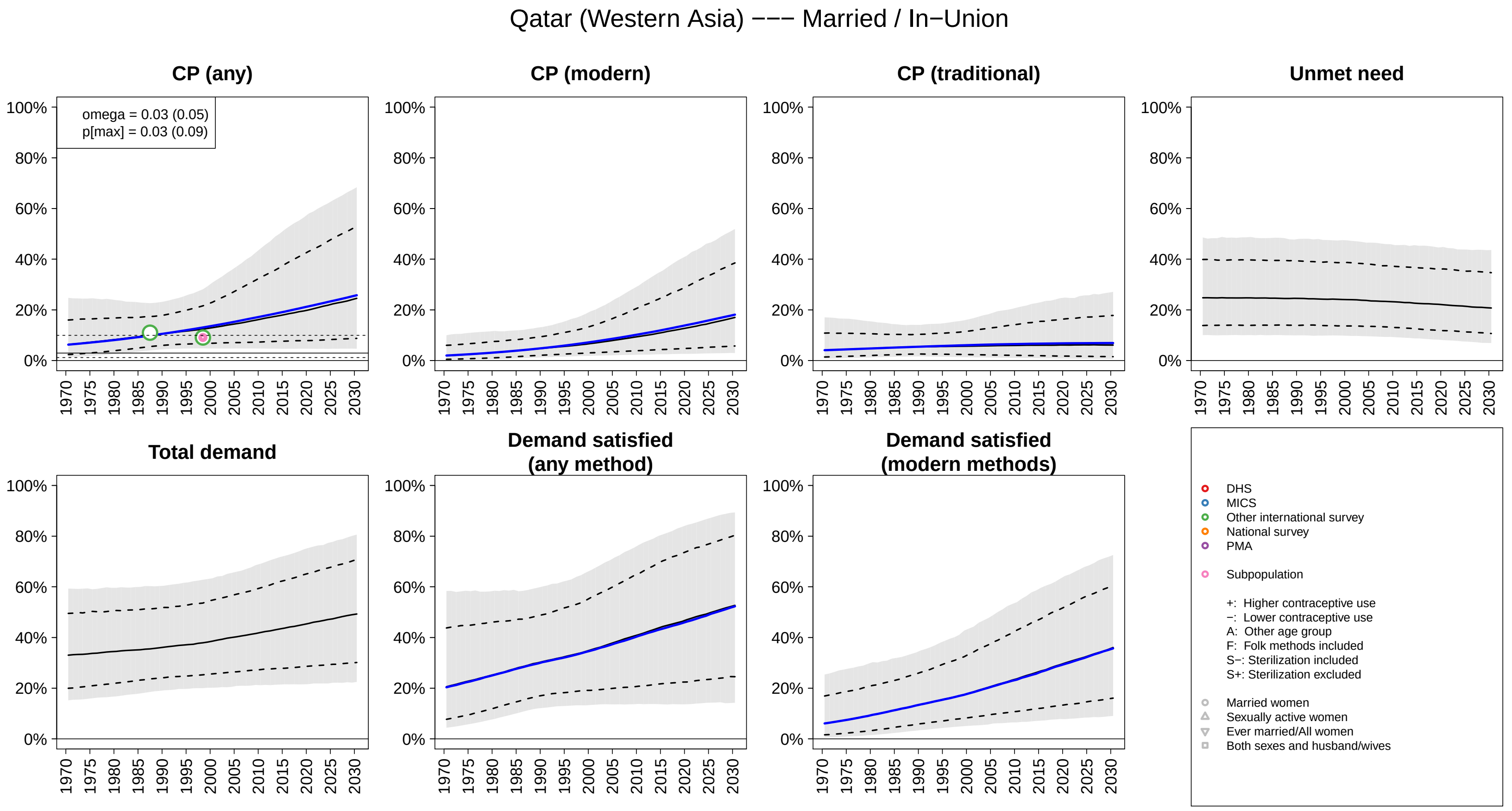
Qatar (Western Asia) ––– Married / In–Union
CP (any)
omega = 0.03 (0.05)
p[max] = 0.03 (0.09)
CP (modern)
CP (traditional)
Unmet need
Total demand
Demand satisfied (any method)
Demand satisfied (modern methods)
DHS
MICS
Other international survey
National survey
PMA
Subpopulation
+: Higher contraceptive use
−: Lower contraceptive use
A: Other age group
F: Folk methods included
S−: Sterilization included
S+: Sterilization excluded
Married women
Sexually active women
Ever married/All women
Both sexes and husband/wives

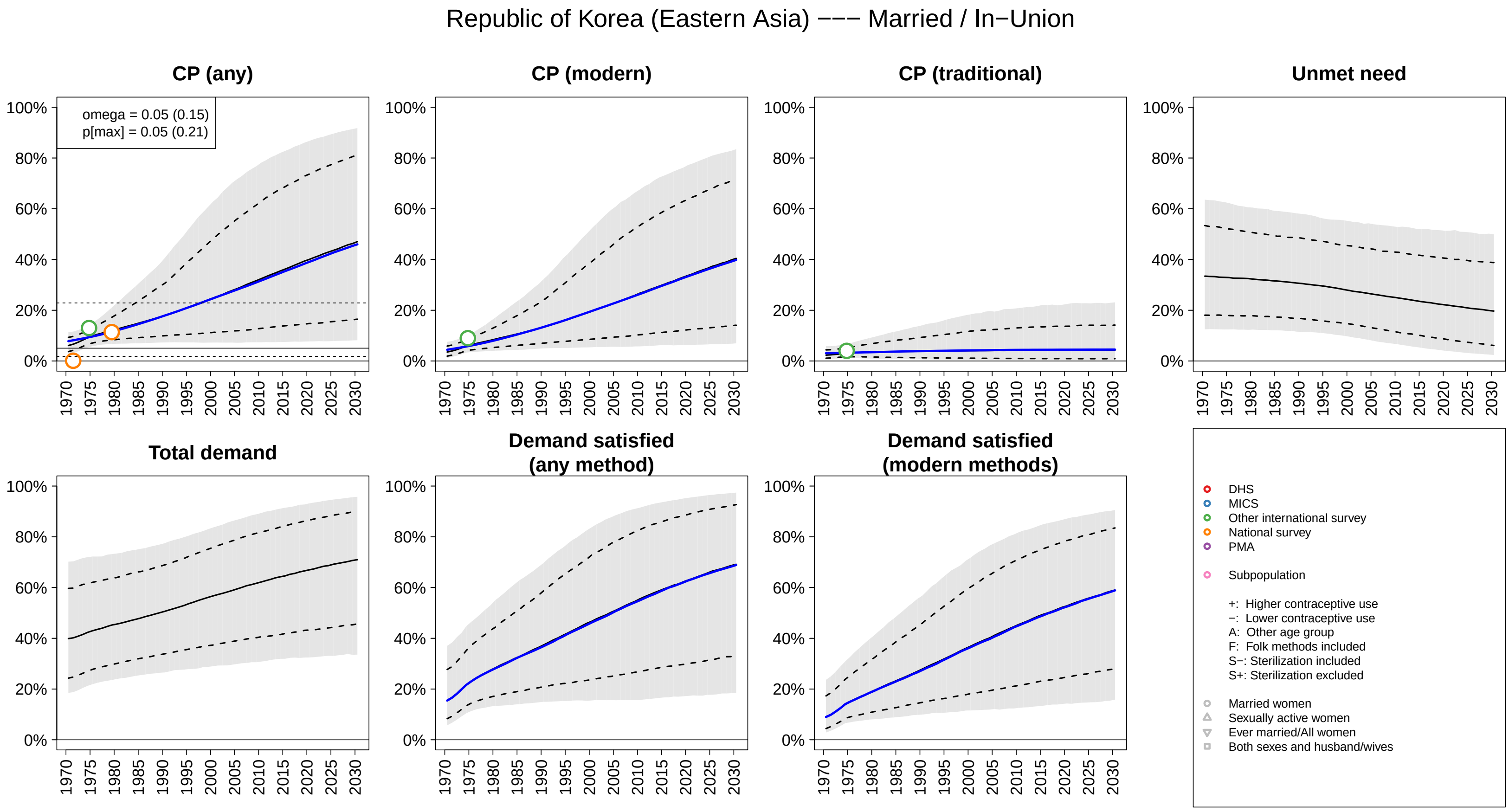
Republic of Korea (Eastern Asia) --- Married / In−Union
CP (any)
omega = 0.05 (0.15)
p[max] = 0.05 (0.21)
CP (modern)
CP (traditional)
Unmet need
Total demand
Demand satisfied (any method)
Demand satisfied (modern methods)
DHS
MICS
Other international survey
National survey
PMA
Subpopulation
+: Higher contraceptive use
−: Lower contraceptive use
A: Other age group
F: Folk methods included
S−: Sterilization included
S+: Sterilization excluded
Married women
Sexually active women
Ever married/All women
Both sexes and husband/wives

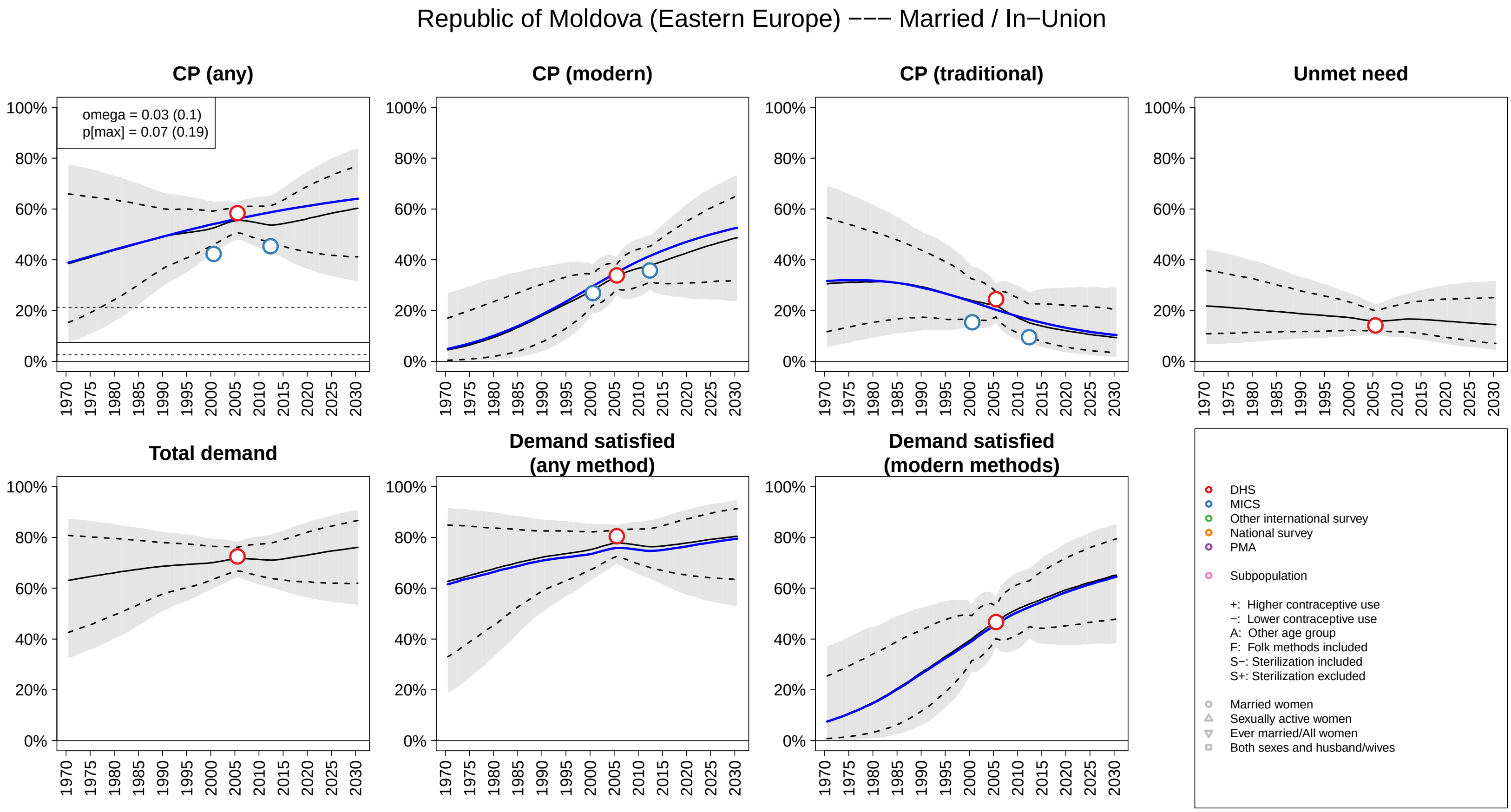

Republic of Moldova (Eastern Europe) --- Married / In−Union
CP (any)
omega = 0.03 (0.1)
p[max] = 0.07 (0.19)
CP (modern)
CP (traditional)
Unmet need
Total demand
Demand satisfied (any method)
Demand satisfied (modern methods)
DHS
MICS
Other international survey
National survey
PMA
Subpopulation
+: Higher contraceptive use
−: Lower contraceptive use
A: Other age group
F: Folk methods included
S−: Sterilization included
S+: Sterilization excluded
Married women
Sexually active women
Ever married/All women
Both sexes and husband/wives

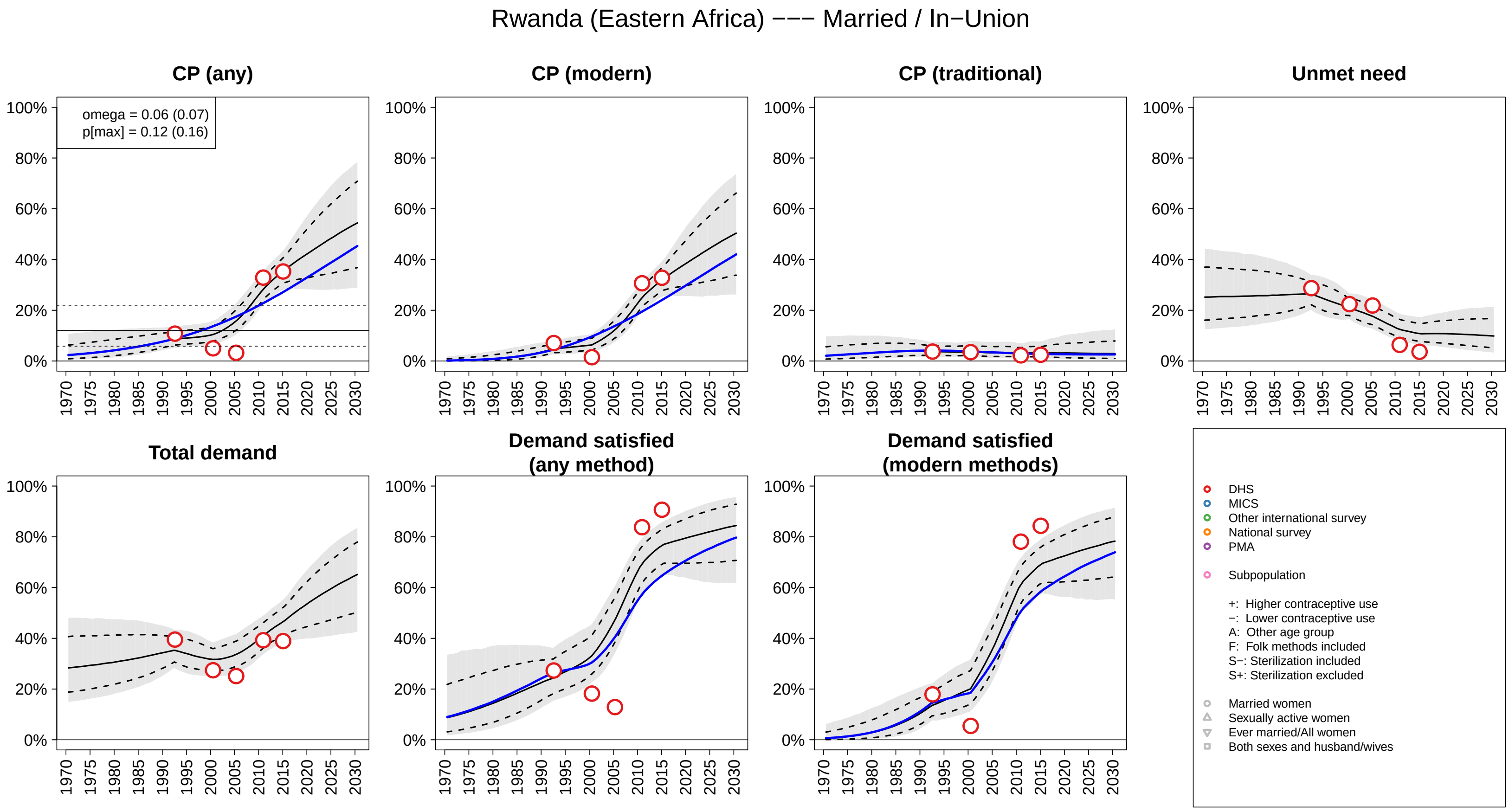
Rwanda (Eastern Africa) --- Married / In-Union
CP (any)
omega = 0.06 (0.07)
p[max] = 0.12 (0.16)
CP (modern)
CP (traditional)
Unmet need
Total demand
Demand satisfied (any method)
Demand satisfied (modern methods)
100%
80%
60%
40%
20%
0%
1970 1975 1980 1985 1990 1995 2000 2005 2010 2015 2020 2025 2030
DHS
MICS
Other international survey
National survey
PMA
Subpopulation
+: Higher contraceptive use
−: Lower contraceptive use
A: Other age group
F: Folk methods included
S−: Sterilization included
S+: Sterilization excluded
Married women
Sexually active women
Ever married/All women
Both sexes and husband/wives

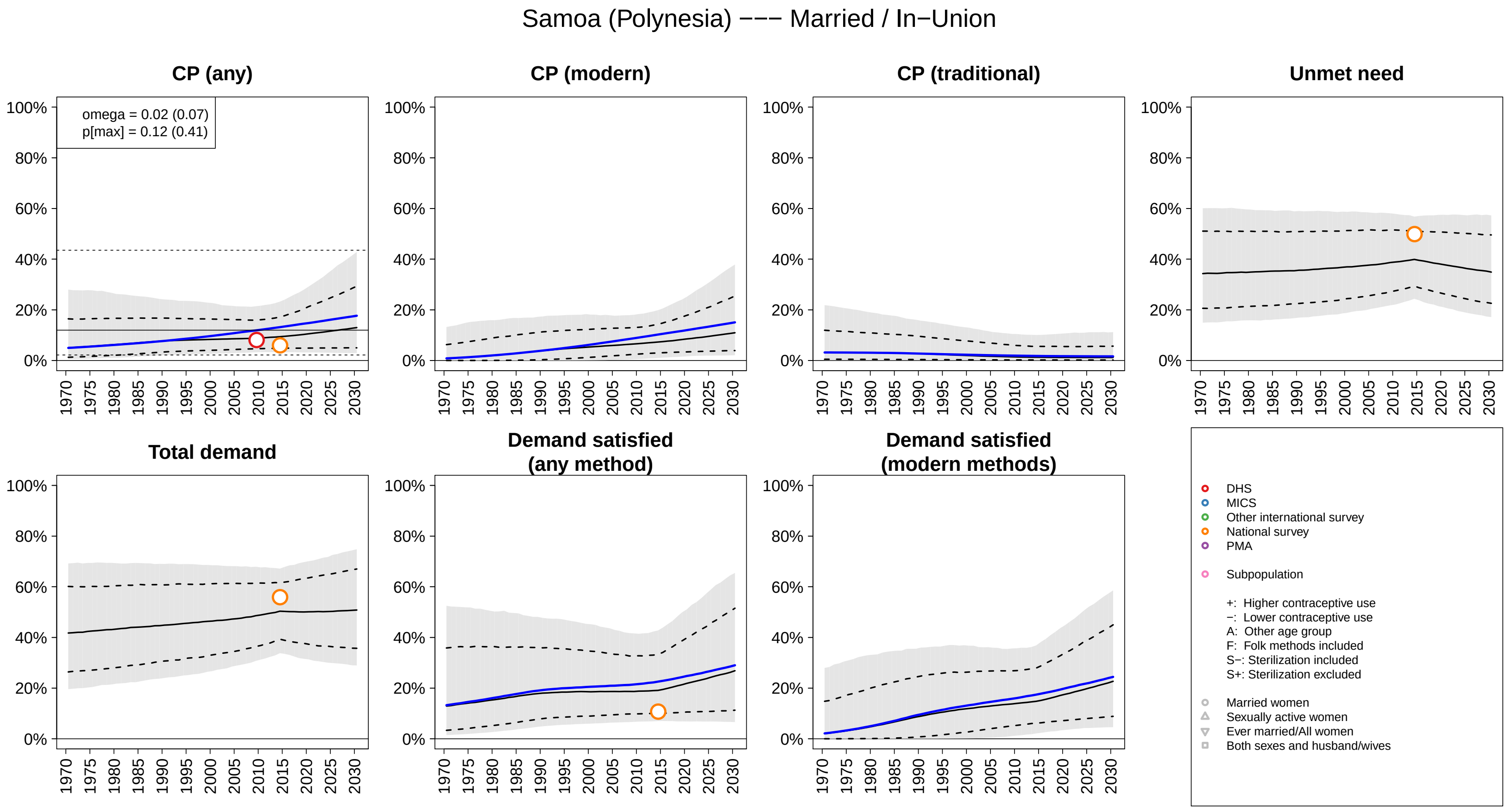
Samoa (Polynesia) −−− Married / In−Union
CP (any)
omega = 0.02 (0.07)
p[max] = 0.12 (0.41)
CP (modern)
CP (traditional)
Unmet need
Total demand
Demand satisfied (any method)
Demand satisfied (modern methods)
DHS
MICS
Other international survey
National survey
PMA
Subpopulation
+: Higher contraceptive use
−: Lower contraceptive use
A: Other age group
F: Folk methods included
S−: Sterilization included
S+: Sterilization excluded
Married women
Sexually active women
Ever married/All women
Both sexes and husband/wives

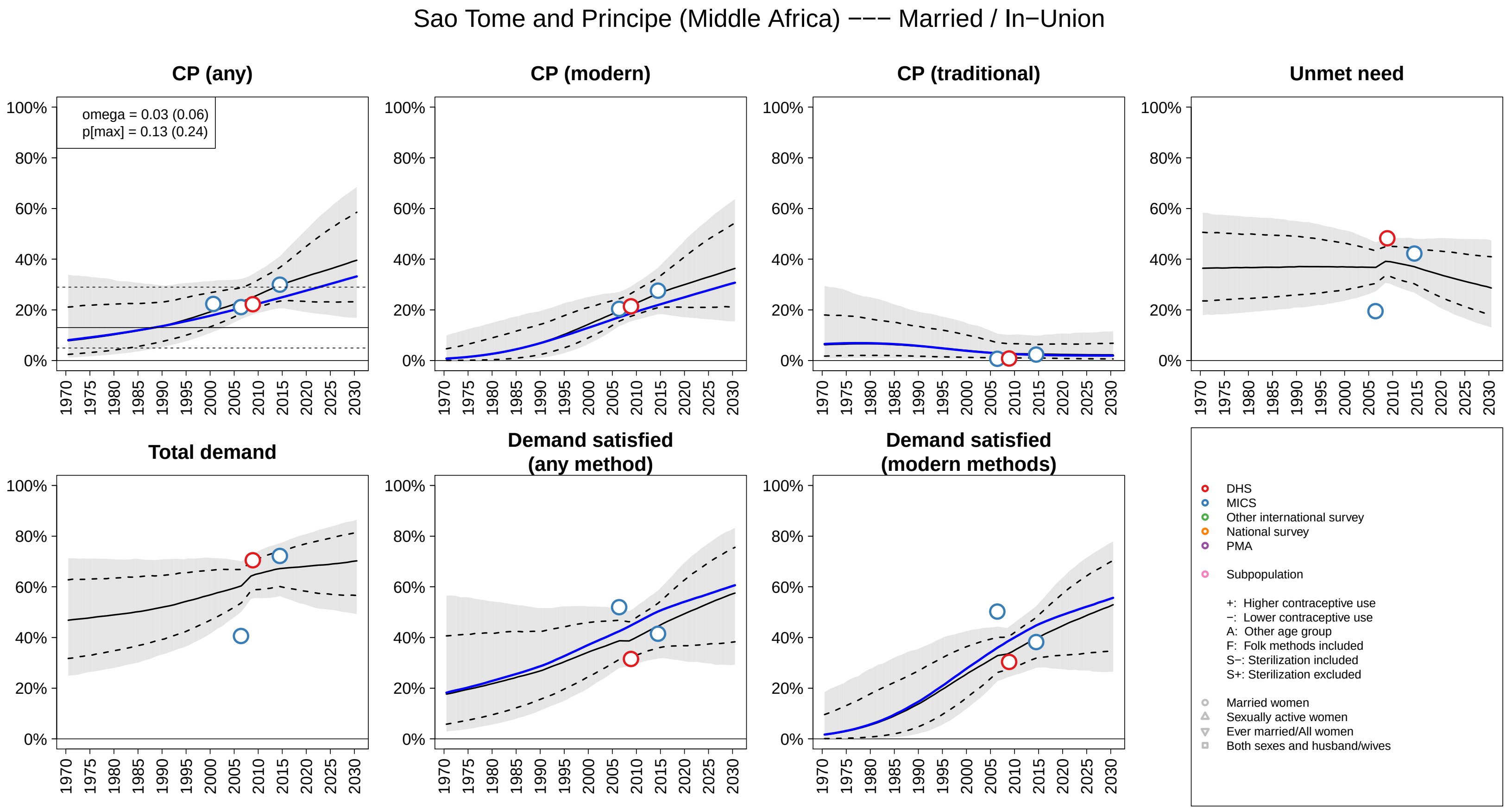
Sao Tome and Principe (Middle Africa) --- Married / In−Union
CP (any)
omega = 0.03 (0.06)
p[max] = 0.13 (0.24)
CP (modern)
CP (traditional)
Unmet need
Total demand
Demand satisfied (any method)
Demand satisfied (modern methods)
DHS
MICS
Other international survey
National survey
PMA
Subpopulation
+: Higher contraceptive use
−: Lower contraceptive use
A: Other age group
F: Folk methods included
S−: Sterilization included
S+: Sterilization excluded
Married women
Sexually active women
Ever married/All women
Both sexes and husband/wives

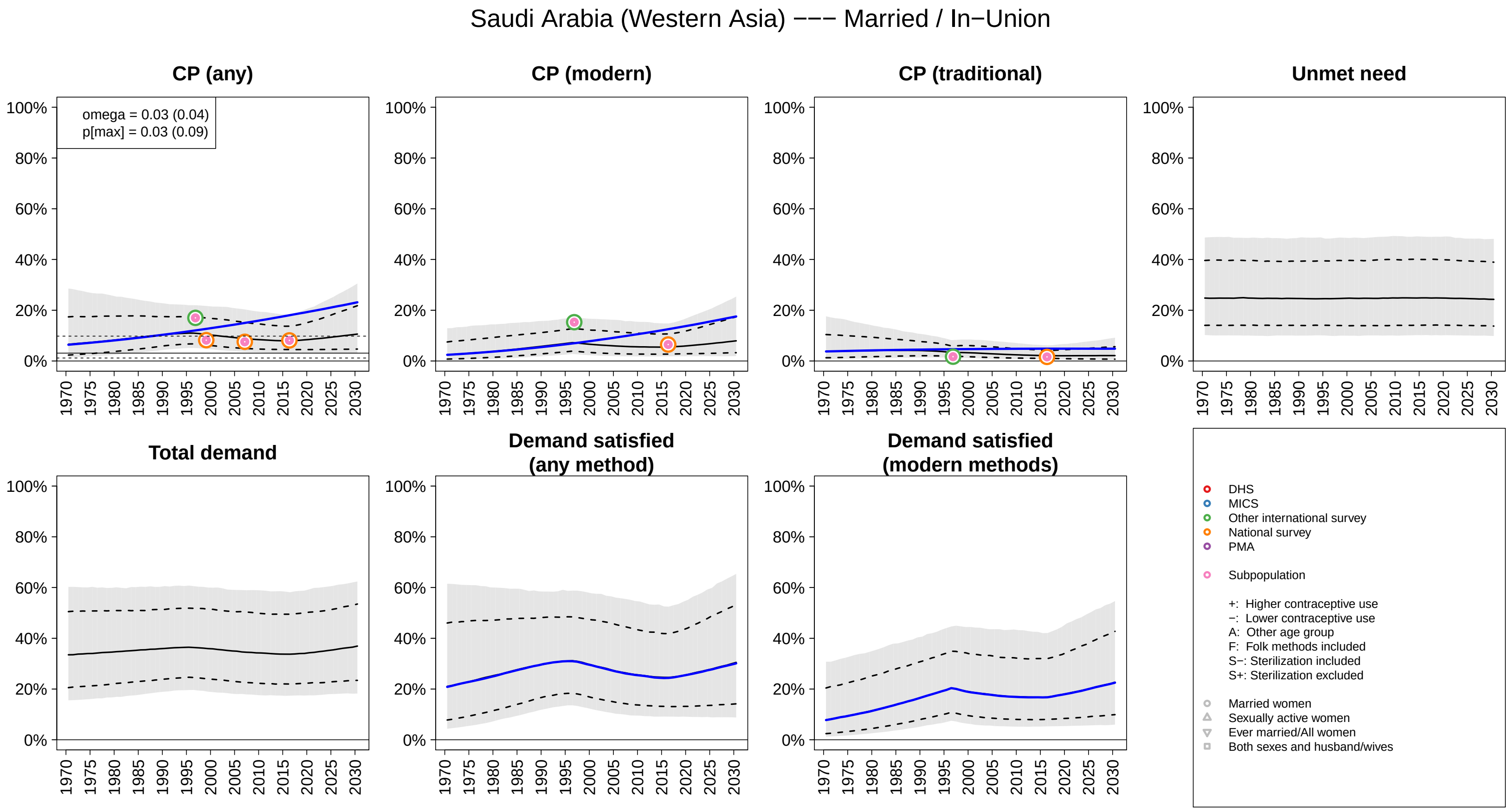

Saudi Arabia (Western Asia) ––– Married / In−Union
CP (any)
omega = 0.03 (0.04)
p[max] = 0.03 (0.09)
CP (modern)
CP (traditional)
Unmet need
Total demand
Demand satisfied (any method)
Demand satisfied (modern methods)
DHS
MICS
Other international survey
National survey
PMA
Subpopulation
+: Higher contraceptive use
−: Lower contraceptive use
A: Other age group
F: Folk methods included
S−: Sterilization included
S+: Sterilization excluded
Married women
Sexually active women
Ever married/All women
Both sexes and husband/wives

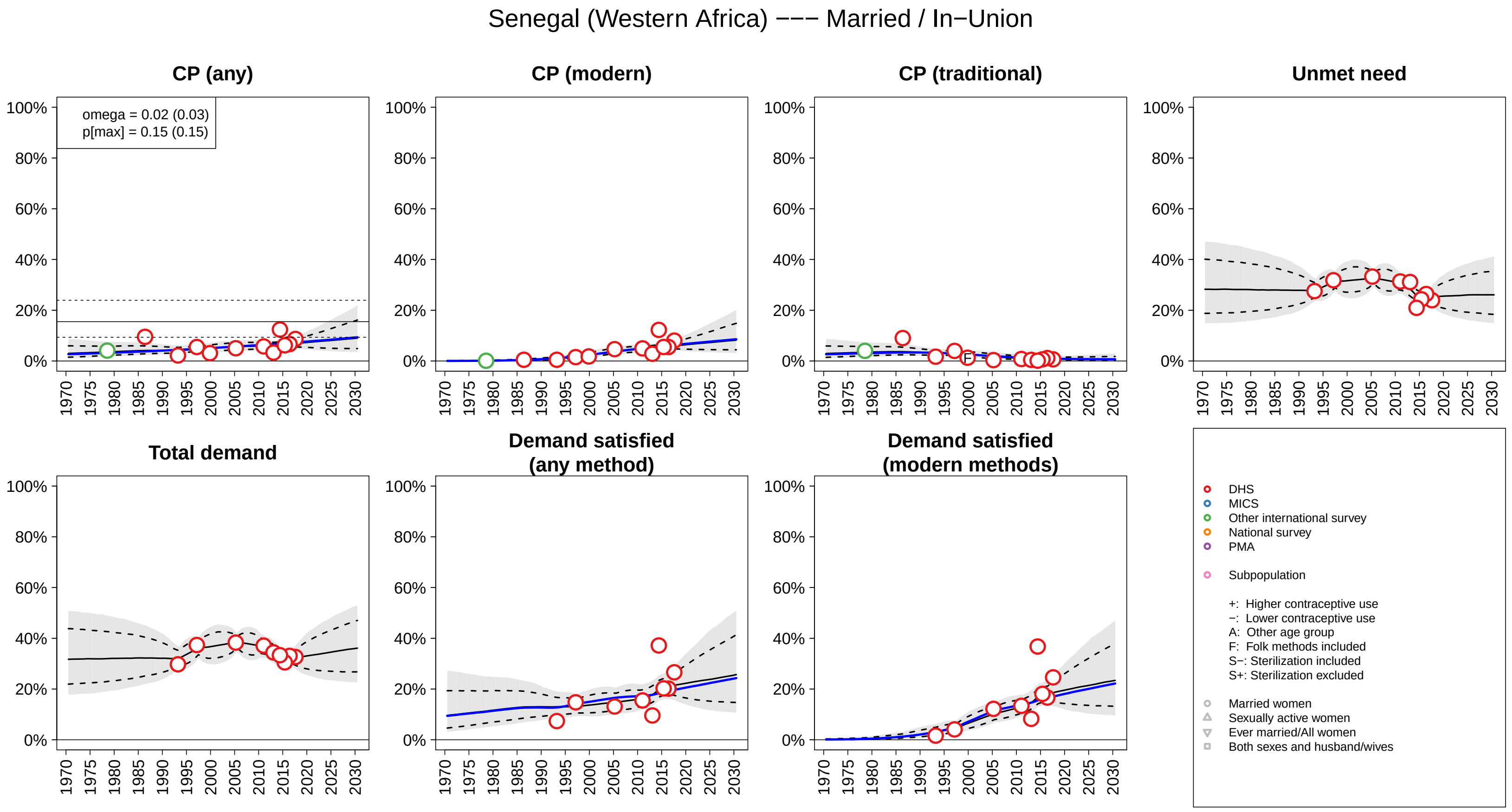
Senegal (Western Africa) −−− Married / In−Union
CP (any)
omega = 0.02 (0.03)
p[max] = 0.15 (0.15)
CP (modern)
CP (traditional)
Unmet need
Total demand
Demand satisfied (any method)
Demand satisfied (modern methods)
DHS
MICS
Other international survey
National survey
PMA
Subpopulation
+: Higher contraceptive use
−: Lower contraceptive use
A: Other age group
F: Folk methods included
S−: Sterilization included
S+: Sterilization excluded
Married women
Sexually active women
Ever married/All women
Both sexes and husband/wives

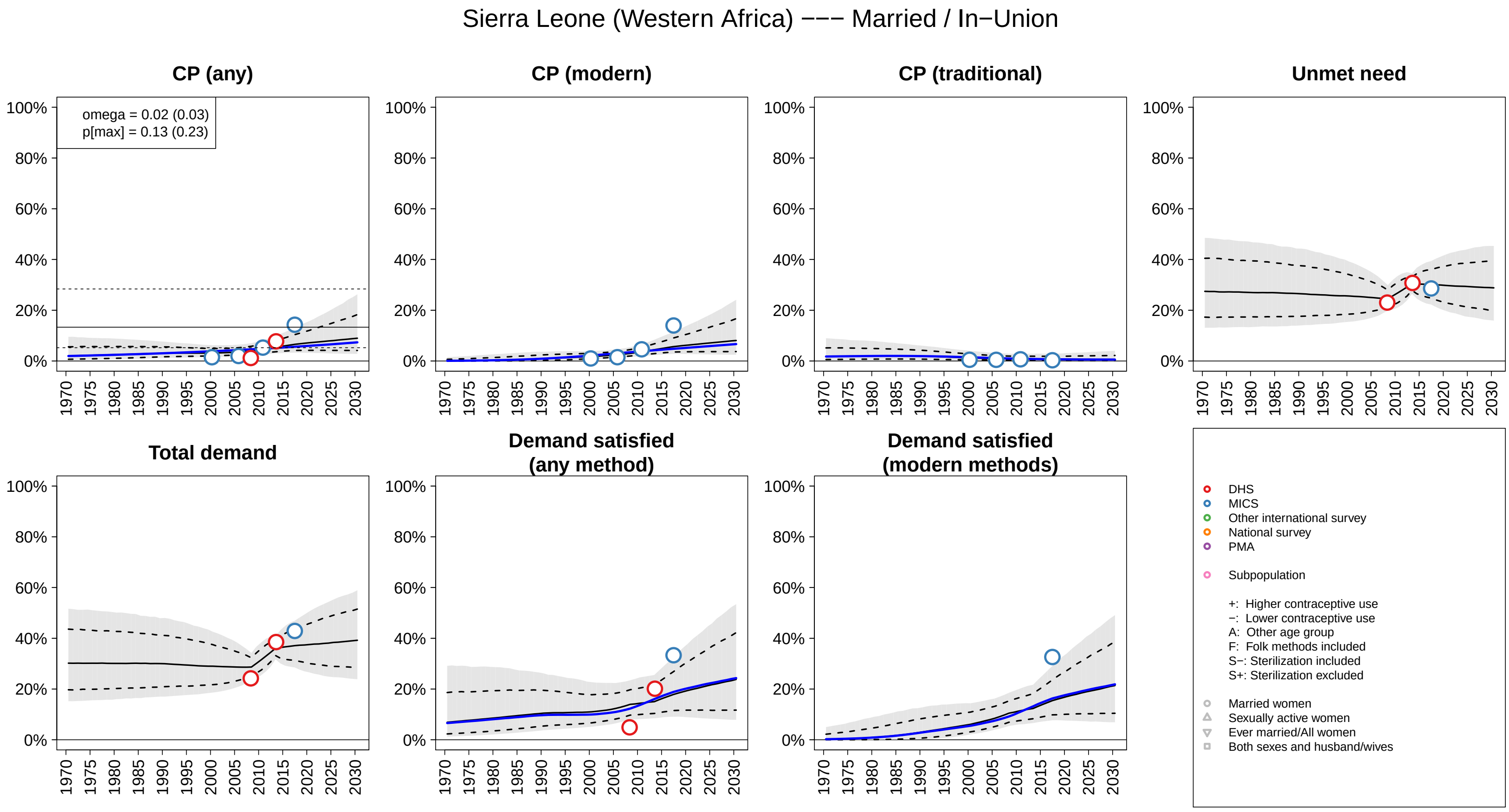

Sierra Leone (Western Africa) --- Married / In−Union
CP (any)
omega = 0.02 (0.03)
p[max] = 0.13 (0.23)
CP (modern)
CP (traditional)
Unmet need
Total demand
Demand satisfied (any method)
Demand satisfied (modern methods)
100%
80%
60%
40%
20%
0%
1970 1975 1980 1985 1990 1995 2000 2005 2010 2015 2020 2025 2030
DHS
MICS
Other international survey
National survey
PMA
Subpopulation
+: Higher contraceptive use
−: Lower contraceptive use
A: Other age group
F: Folk methods included
S−: Sterilization included
S+: Sterilization excluded
Married women
Sexually active women
Ever married/All women
Both sexes and husband/wives

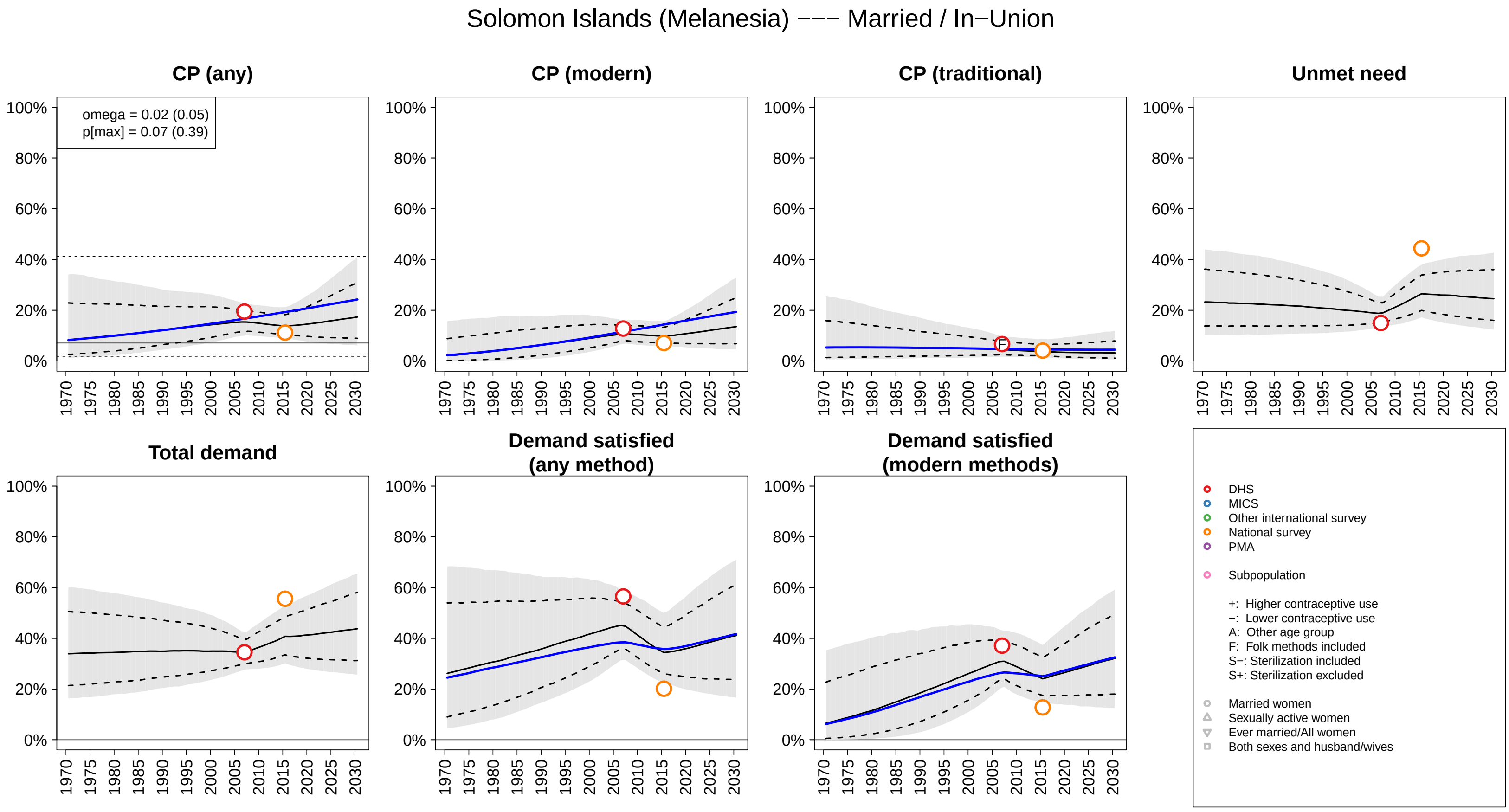

Solomon Islands (Melanesia) −−− Married / In−Union
CP (any)
omega = 0.02 (0.05)
p[max] = 0.07 (0.39)
CP (modern)
CP (traditional)
Unmet need
Total demand
Demand satisfied (any method)
Demand satisfied (modern methods)
100%
80%
60%
40%
20%
0%
1970 1975 1980 1985 1990 1995 2000 2005 2010 2015 2020 2025 2030
DHS
MICS
Other international survey
National survey
PMA
Subpopulation
+: Higher contraceptive use
−: Lower contraceptive use
A: Other age group
F: Folk methods included
S−: Sterilization included
S+: Sterilization excluded
Married women
Sexually active women
Ever married/All women
Both sexes and husband/wives

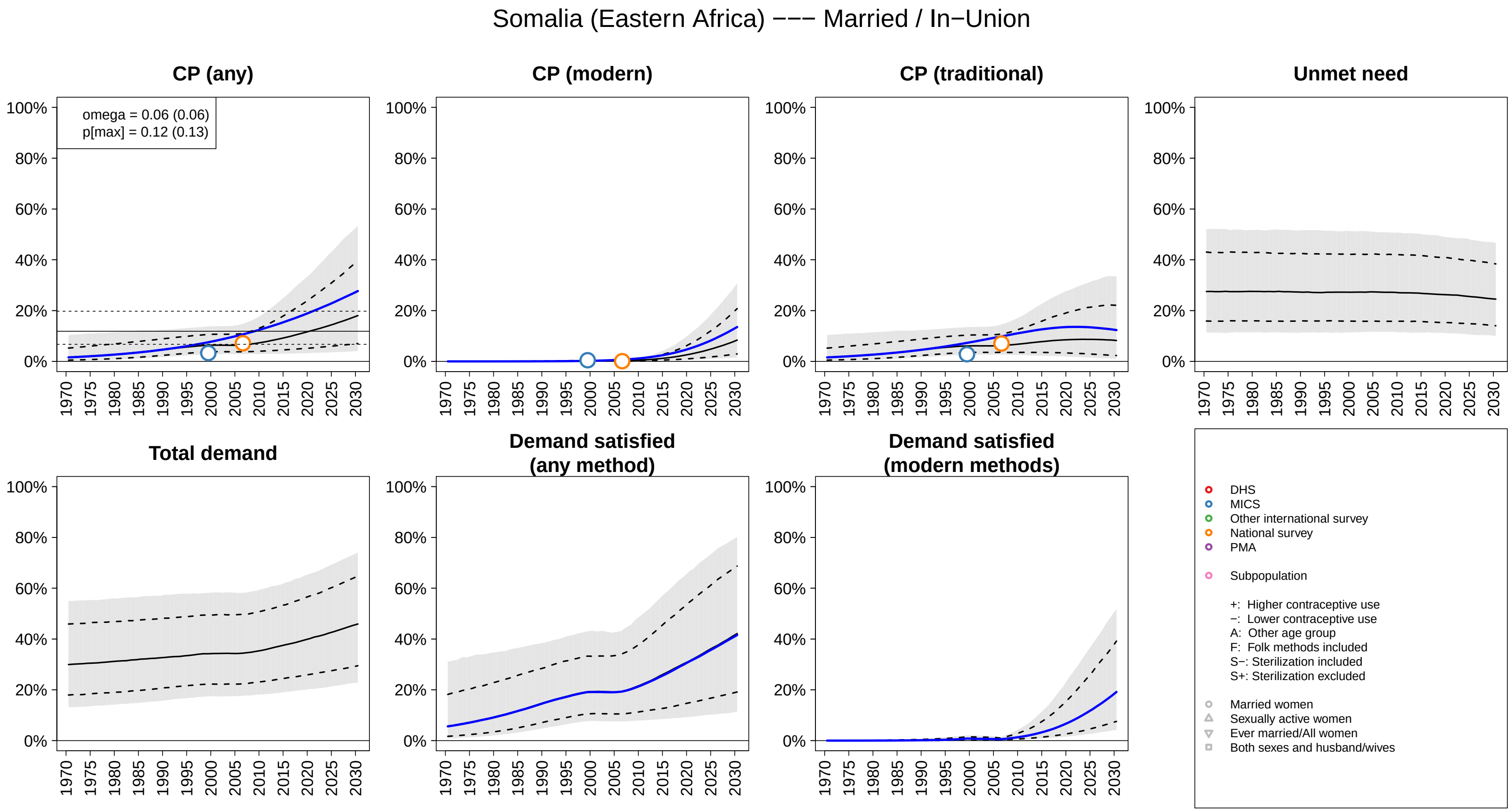
Somalia (Eastern Africa) --- Married / In-Union
CP (any)
omega = 0.06 (0.06)
p[max] = 0.12 (0.13)
CP (modern)
CP (traditional)
Unmet need
Total demand
Demand satisfied (any method)
Demand satisfied (modern methods)
DHS
MICS
Other international survey
National survey
PMA
Subpopulation
+: Higher contraceptive use
−: Lower contraceptive use
A: Other age group
F: Folk methods included
S−: Sterilization included
S+: Sterilization excluded
Married women
Sexually active women
Ever married/All women
Both sexes and husband/wives

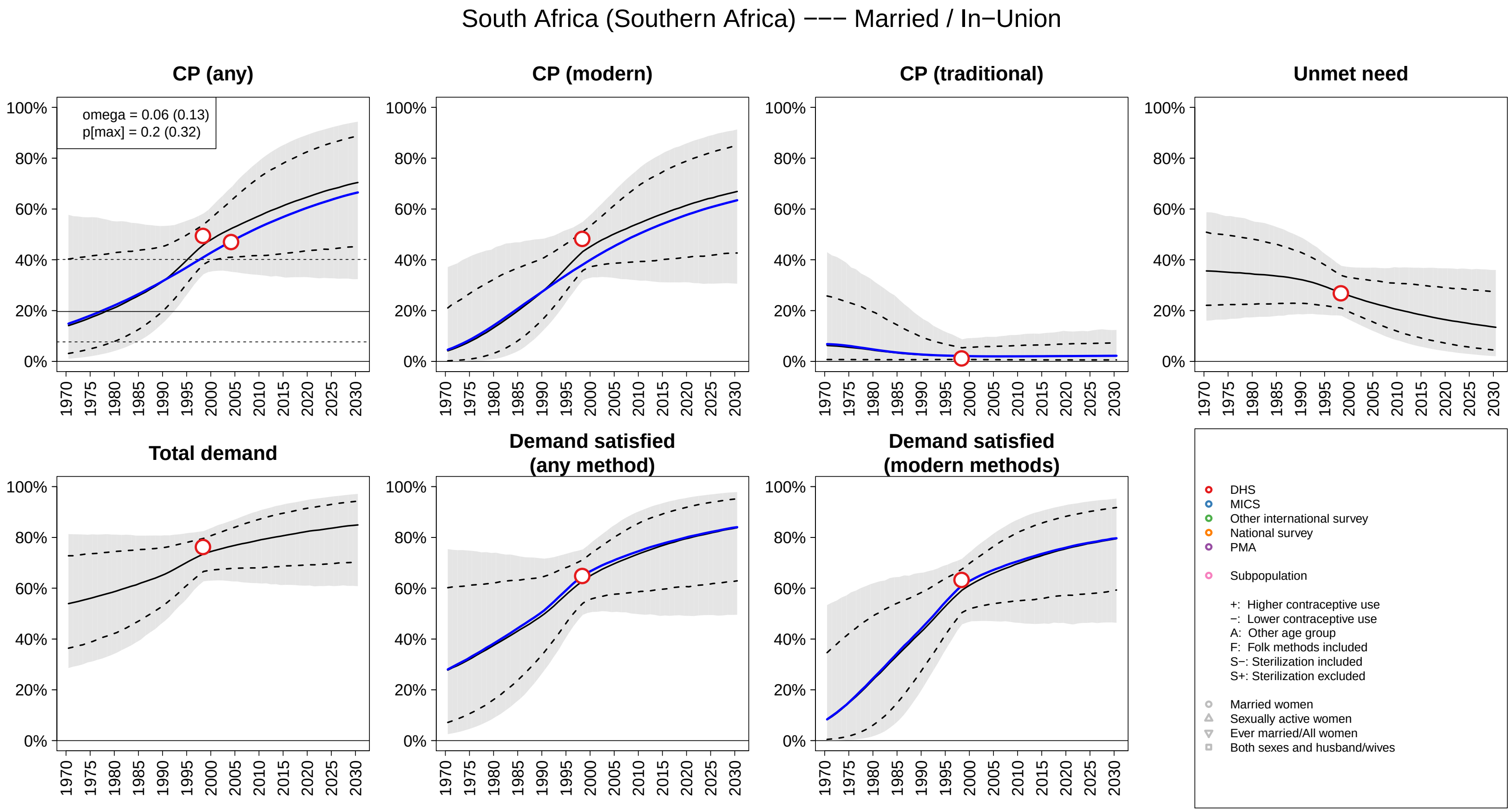
South Africa (Southern Africa) −−− Married / In−Union
CP (any)
omega = 0.06 (0.13)
p[max] = 0.2 (0.32)
CP (modern)
CP (traditional)
Unmet need
Total demand
Demand satisfied
(any method)
Demand satisfied
(modern methods)
DHS
MICS
Other international survey
National survey
PMA
Subpopulation
+: Higher contraceptive use
−: Lower contraceptive use
A: Other age group
F: Folk methods included
S−: Sterilization included
S+: Sterilization excluded
Married women
Sexually active women
Ever married/All women
Both sexes and husband/wives

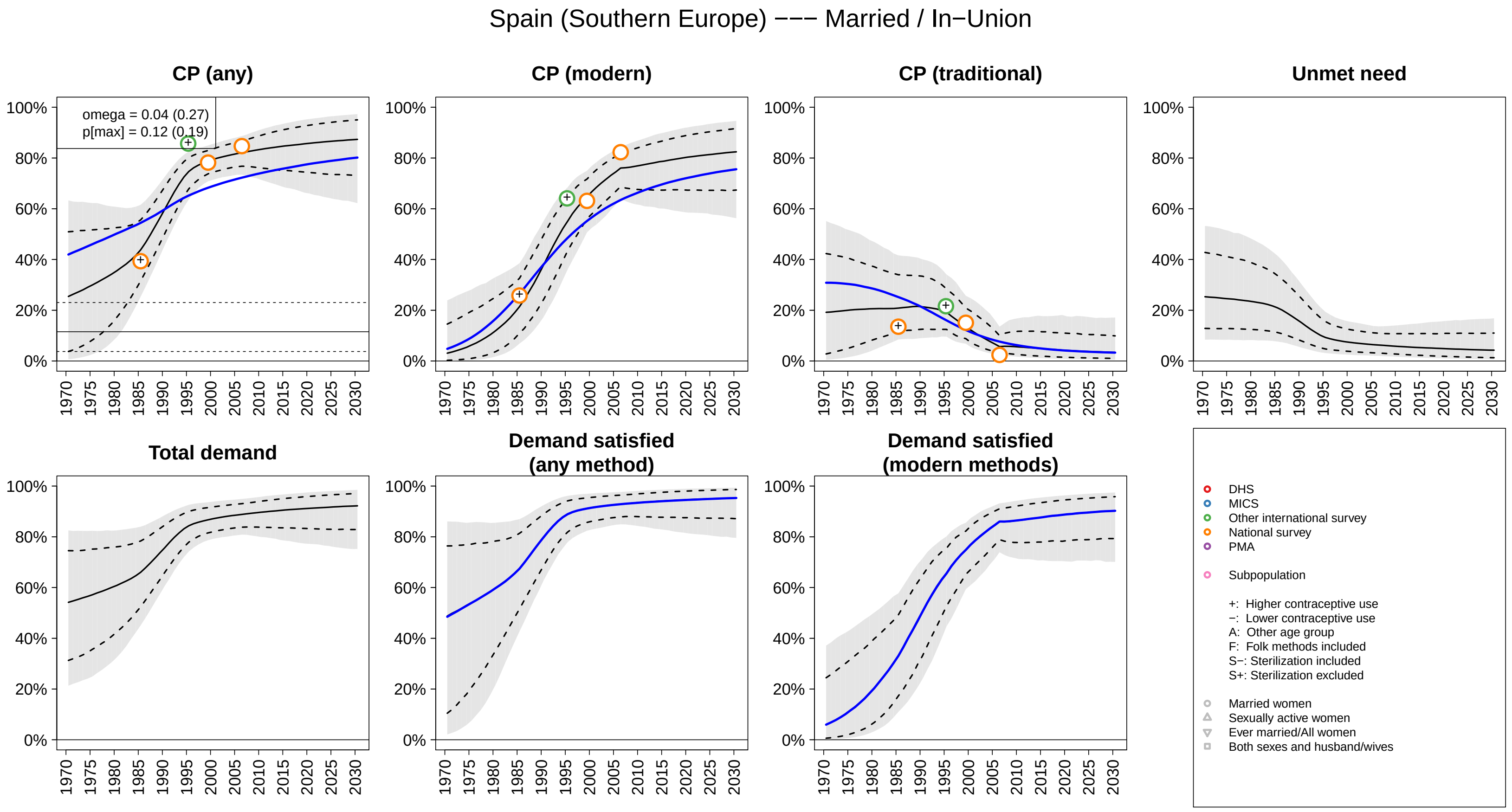

Spain (Southern Europe) ––– Married / In–Union
CP (any)
omega = 0.04 (0.27)
p[max] = 0.12 (0.19)
CP (modern)
CP (traditional)
Unmet need
Total demand
Demand satisfied (any method)
Demand satisfied (modern methods)
0%
20%
40%
60%
80%
100%
1970
1975
1980
1985
1990
1995
2000
2005
2010
2015
2020
2025
2030
DHS
MICS
Other international survey
National survey
PMA
Subpopulation
+: Higher contraceptive use
−: Lower contraceptive use
A: Other age group
F: Folk methods included
S−: Sterilization included
S+: Sterilization excluded
Married women
Sexually active women
Ever married/All women
Both sexes and husband/wives

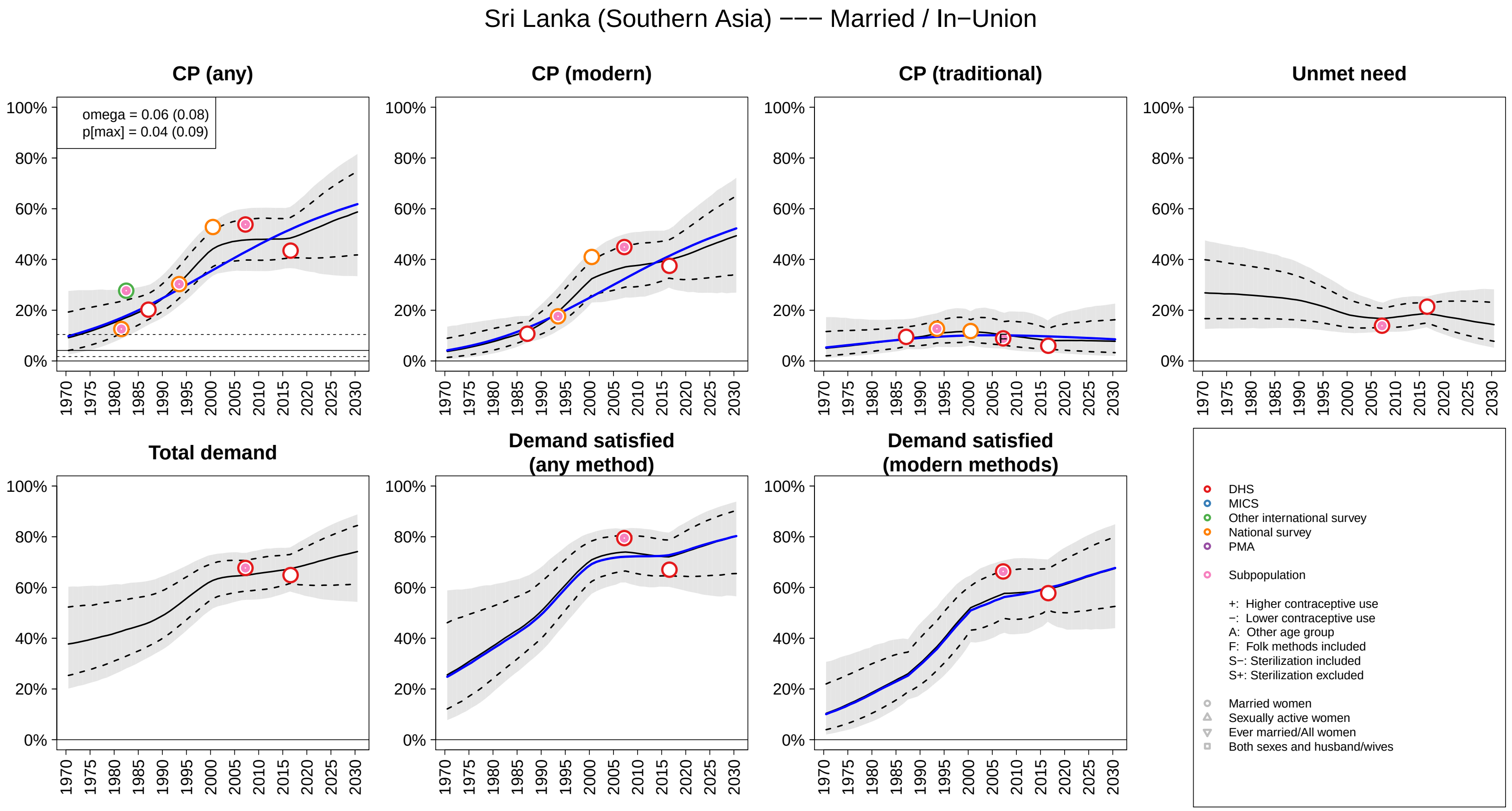
Sri Lanka (Southern Asia) --- Married / In−Union
CP (any)
omega = 0.06 (0.08)
p[max] = 0.04 (0.09)
CP (modern)
CP (traditional)
Unmet need
Total demand
Demand satisfied (any method)
Demand satisfied (modern methods)
0%
20%
40%
60%
80%
100%
1970
1975
1980
1985
1990
1995
2000
2005
2010
2015
2020
2025
2030
DHS
MICS
Other international survey
National survey
PMA
Subpopulation
+: Higher contraceptive use
−: Lower contraceptive use
A: Other age group
F: Folk methods included
S−: Sterilization included
S+: Sterilization excluded
Married women
Sexually active women
Ever married/All women
Both sexes and husband/wives

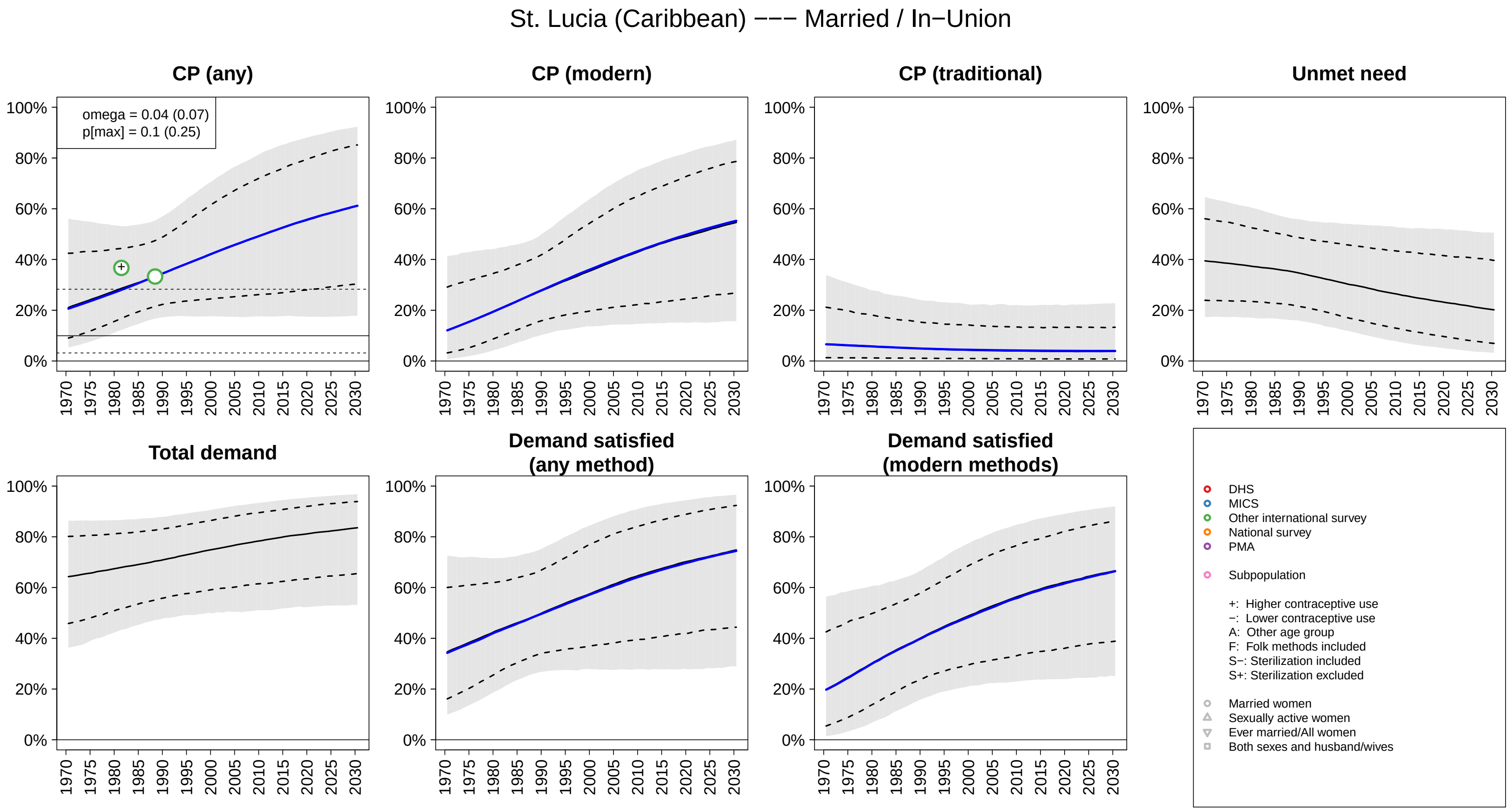
St. Lucia (Caribbean) --- Married / In-Union
CP (any)
omega = 0.04 (0.07)
p[max] = 0.1 (0.25)
CP (modern)
CP (traditional)
Unmet need
Total demand
Demand satisfied (any method)
Demand satisfied (modern methods)
DHS
MICS
Other international survey
National survey
PMA
Subpopulation
+: Higher contraceptive use
−: Lower contraceptive use
A: Other age group
F: Folk methods included
S−: Sterilization included
S+: Sterilization excluded
Married women
Sexually active women
Ever married/All women
Both sexes and husband/wives

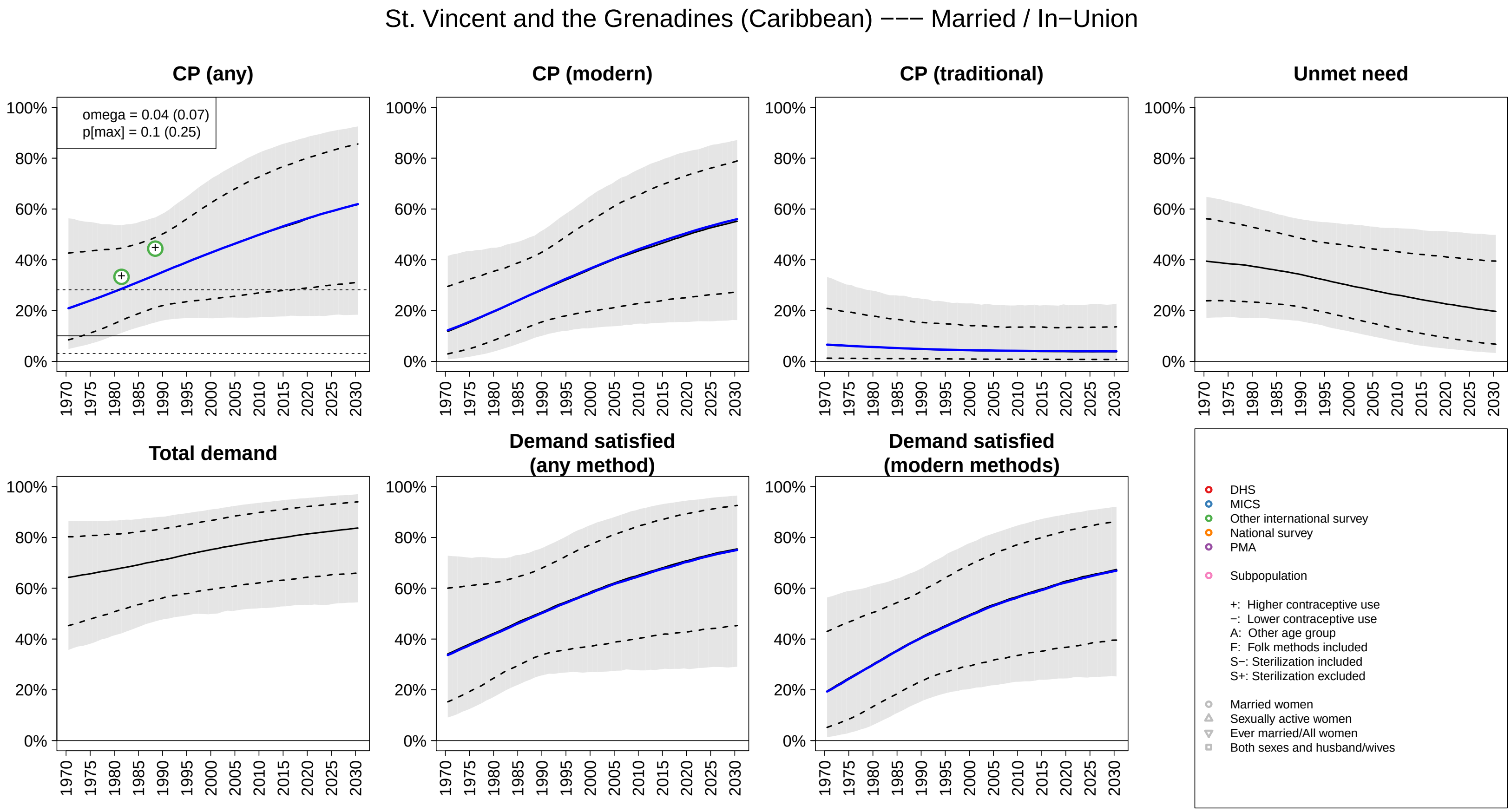
St. Vincent and the Grenadines (Caribbean) −−− Married / In−Union
CP (any)
omega = 0.04 (0.07)
p[max] = 0.1 (0.25)
CP (modern)
CP (traditional)
Unmet need
Total demand
Demand satisfied
(any method)
Demand satisfied
(modern methods)
DHS
MICS
Other international survey
National survey
PMA
Subpopulation
+: Higher contraceptive use
−: Lower contraceptive use
A: Other age group
F: Folk methods included
S−: Sterilization included
S+: Sterilization excluded
Married women
Sexually active women
Ever married/All women
Both sexes and husband/wives

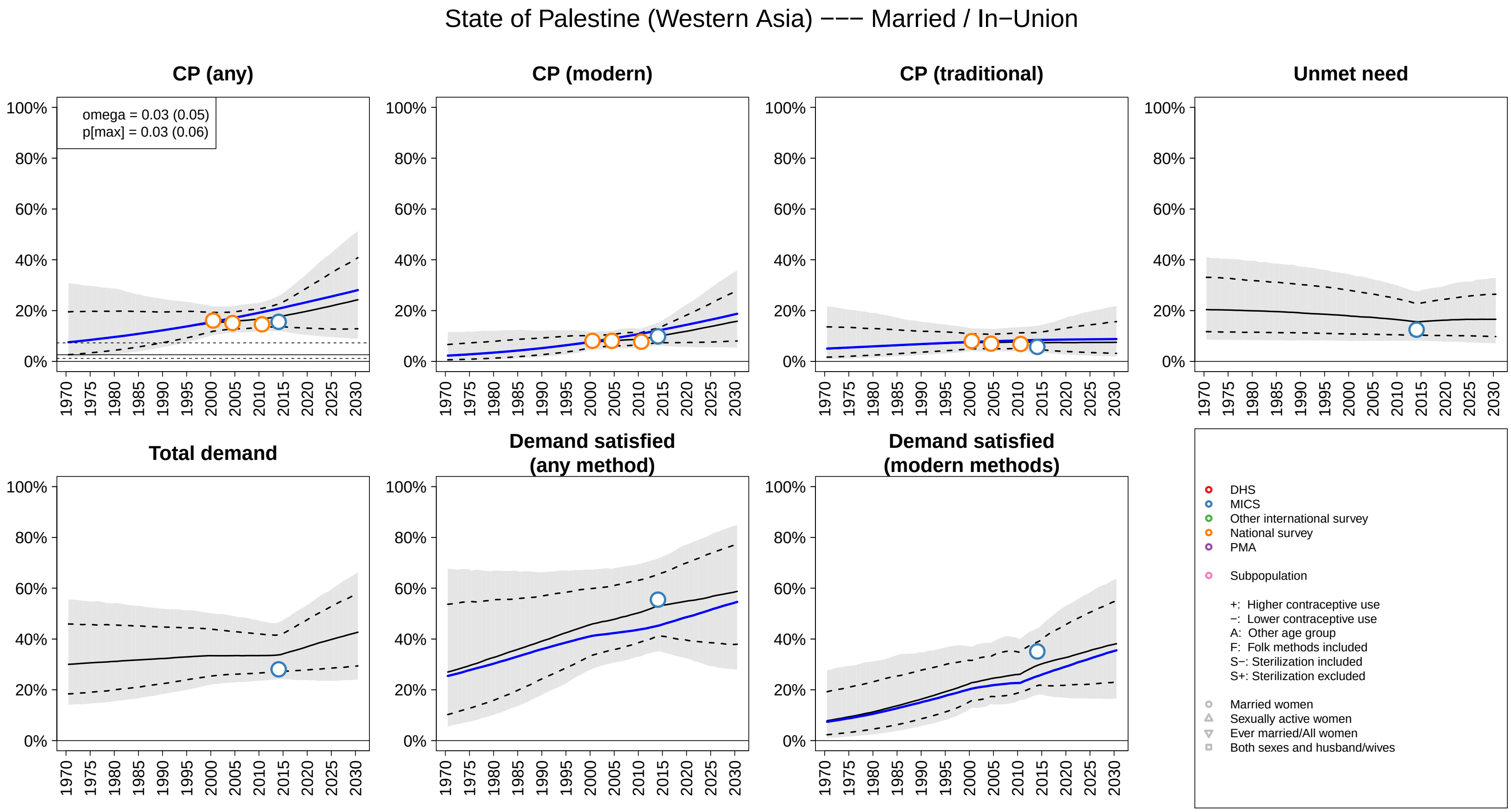
State of Palestine (Western Asia) ––– Married / In−Union
CP (any)
omega = 0.03 (0.05)
p[max] = 0.03 (0.06)
CP (modern)
CP (traditional)
Unmet need
Total demand
Demand satisfied (any method)
Demand satisfied (modern methods)
DHS
MICS
Other international survey
National survey
PMA
Subpopulation
+: Higher contraceptive use
−: Lower contraceptive use
A: Other age group
F: Folk methods included
S−: Sterilization included
S+: Sterilization excluded
Married women
Sexually active women
Ever married/All women
Both sexes and husband/wives

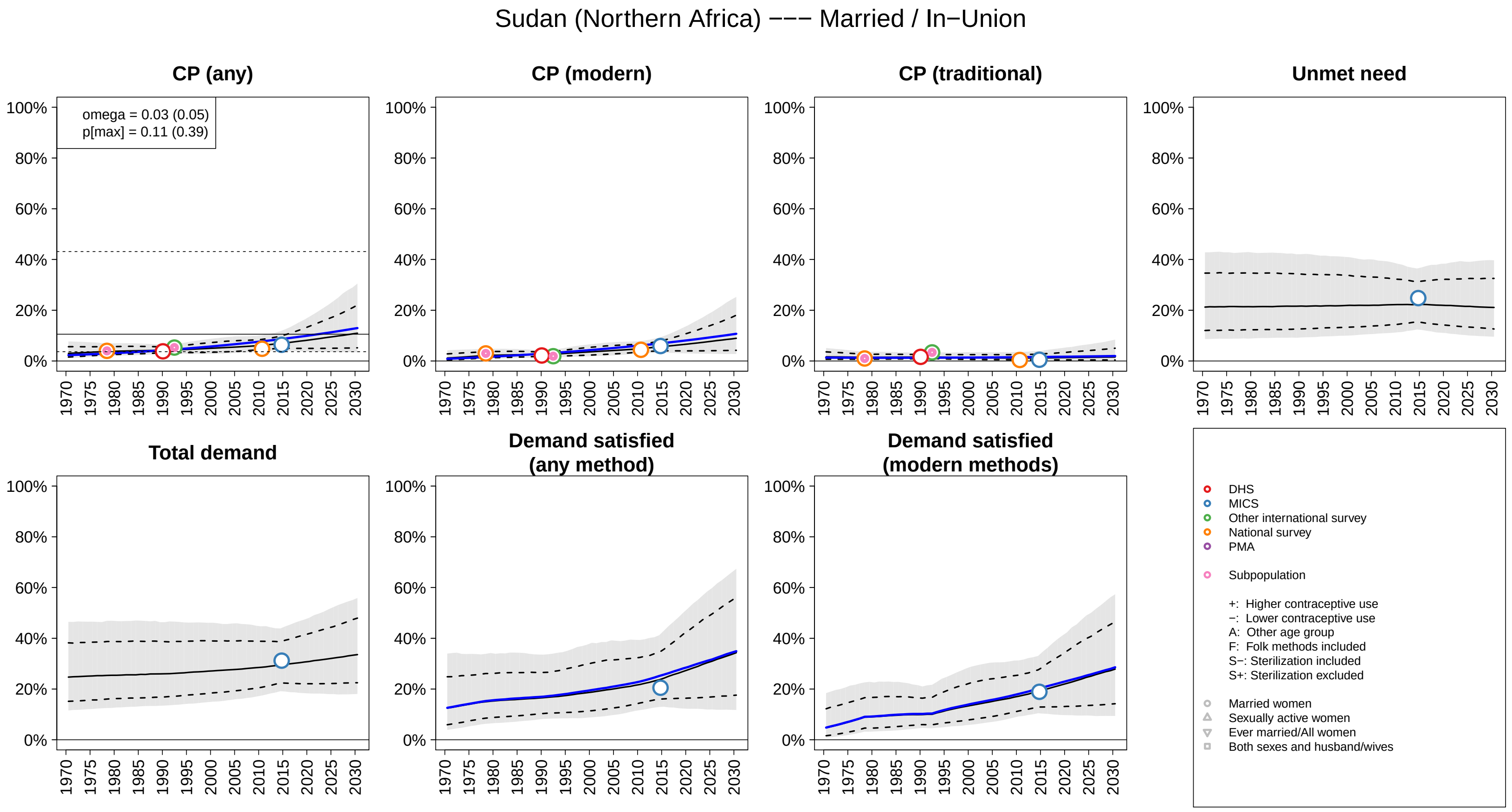

Sudan (Northern Africa) ––– Married / In−Union
CP (any)
omega = 0.03 (0.05)
p[max] = 0.11 (0.39)
CP (modern)
CP (traditional)
Unmet need
Total demand
Demand satisfied (any method)
Demand satisfied (modern methods)
100%
80%
60%
40%
20%
0%
1970 1975 1980 1985 1990 1995 2000 2005 2010 2015 2020 2025 2030
DHS
MICS
Other international survey
National survey
PMA
Subpopulation
+: Higher contraceptive use
−: Lower contraceptive use
A: Other age group
F: Folk methods included
S−: Sterilization included
S+: Sterilization excluded
Married women
Sexually active women
Ever married/All women
Both sexes and husband/wives

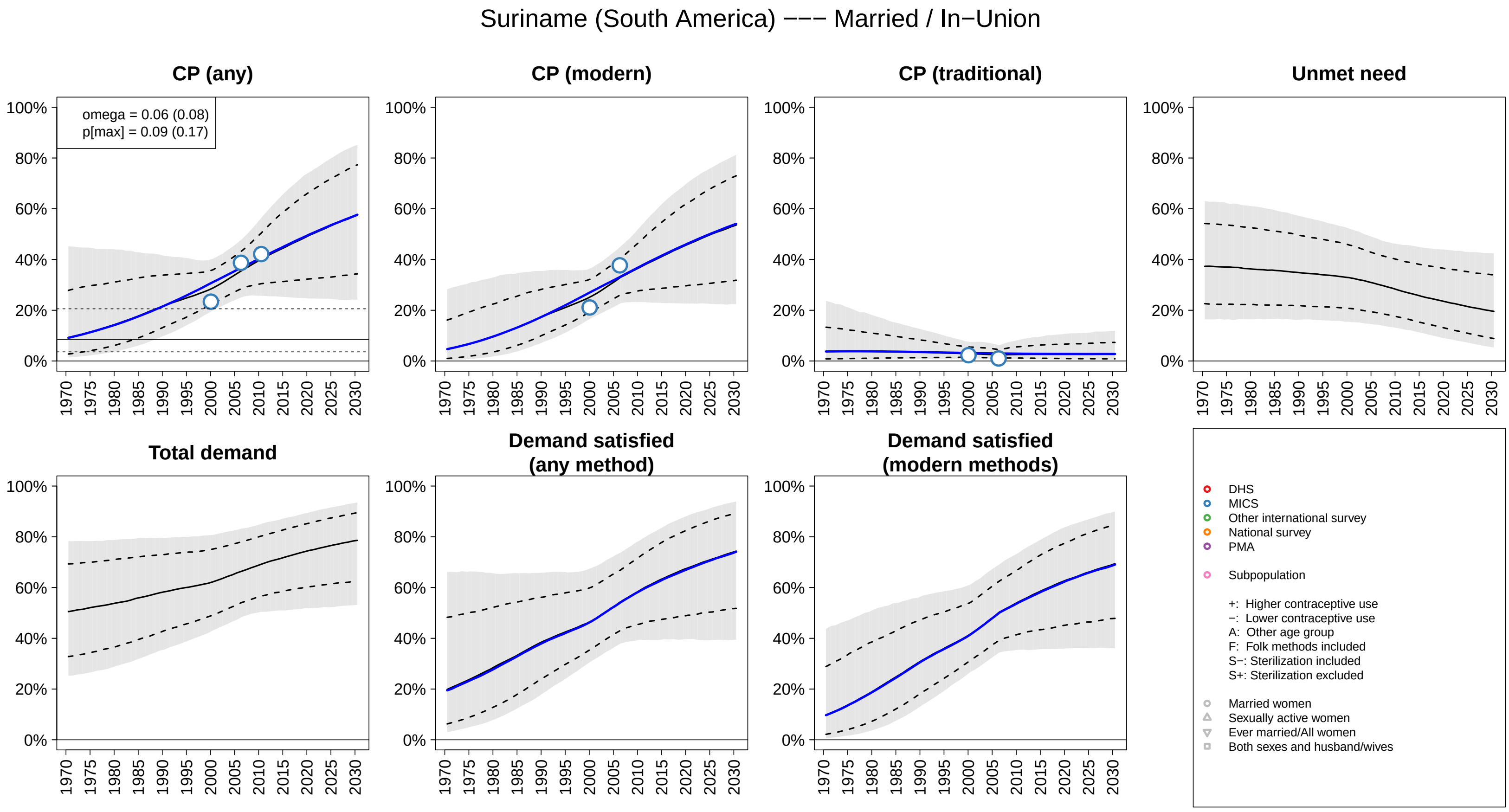
Suriname (South America) ––– Married / In−Union
CP (any)
omega = 0.06 (0.08)
p[max] = 0.09 (0.17)
CP (modern)
CP (traditional)
Unmet need
Total demand
Demand satisfied (any method)
Demand satisfied (modern methods)
0%
20%
40%
60%
80%
100%
1970
1975
1980
1985
1990
1995
2000
2005
2010
2015
2020
2025
2030
DHS
MICS
Other international survey
National survey
PMA
Subpopulation
+: Higher contraceptive use
−: Lower contraceptive use
A: Other age group
F: Folk methods included
S−: Sterilization included
S+: Sterilization excluded
Married women
Sexually active women
Ever married/All women
Both sexes and husband/wives

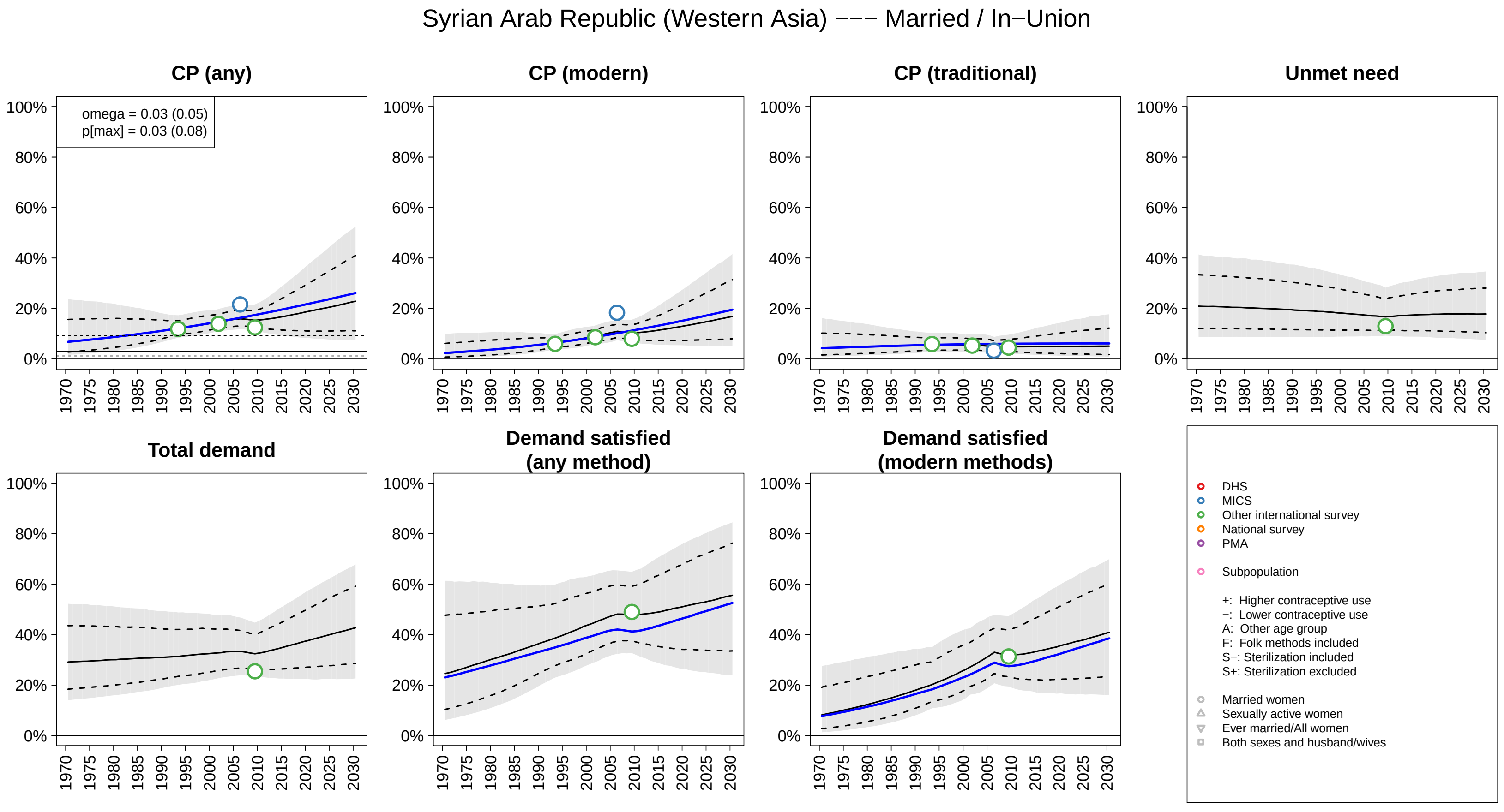
Syrian Arab Republic (Western Asia) −−− Married / In−Union
CP (any)
omega = 0.03 (0.05)
p[max] = 0.03 (0.08)
CP (modern)
CP (traditional)
Unmet need
Total demand
Demand satisfied (any method)
Demand satisfied (modern methods)
DHS
MICS
Other international survey
National survey
PMA
Subpopulation
+: Higher contraceptive use
−: Lower contraceptive use
A: Other age group
F: Folk methods included
S−: Sterilization included
S+: Sterilization excluded
Married women
Sexually active women
Ever married/All women
Both sexes and husband/wives

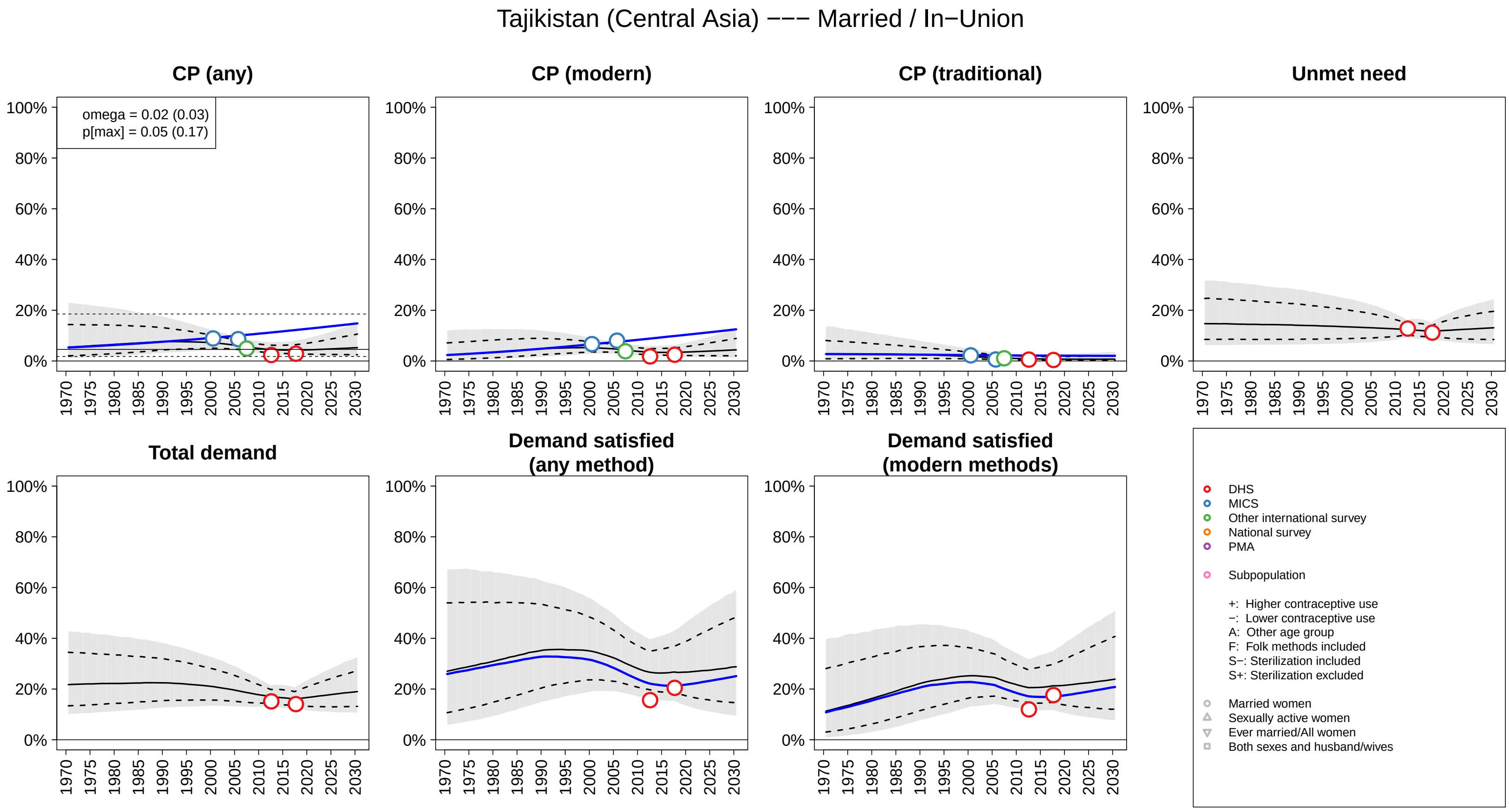
Tajikistan (Central Asia) ––– Married / In–Union
CP (any)
omega = 0.02 (0.03)
p[max] = 0.05 (0.17)
CP (modern)
CP (traditional)
Unmet need
Total demand
Demand satisfied (any method)
Demand satisfied (modern methods)
DHS
MICS
Other international survey
National survey
PMA
Subpopulation
+: Higher contraceptive use
−: Lower contraceptive use
A: Other age group
F: Folk methods included
S−: Sterilization included
S+: Sterilization excluded
Married women
Sexually active women
Ever married/All women
Both sexes and husband/wives

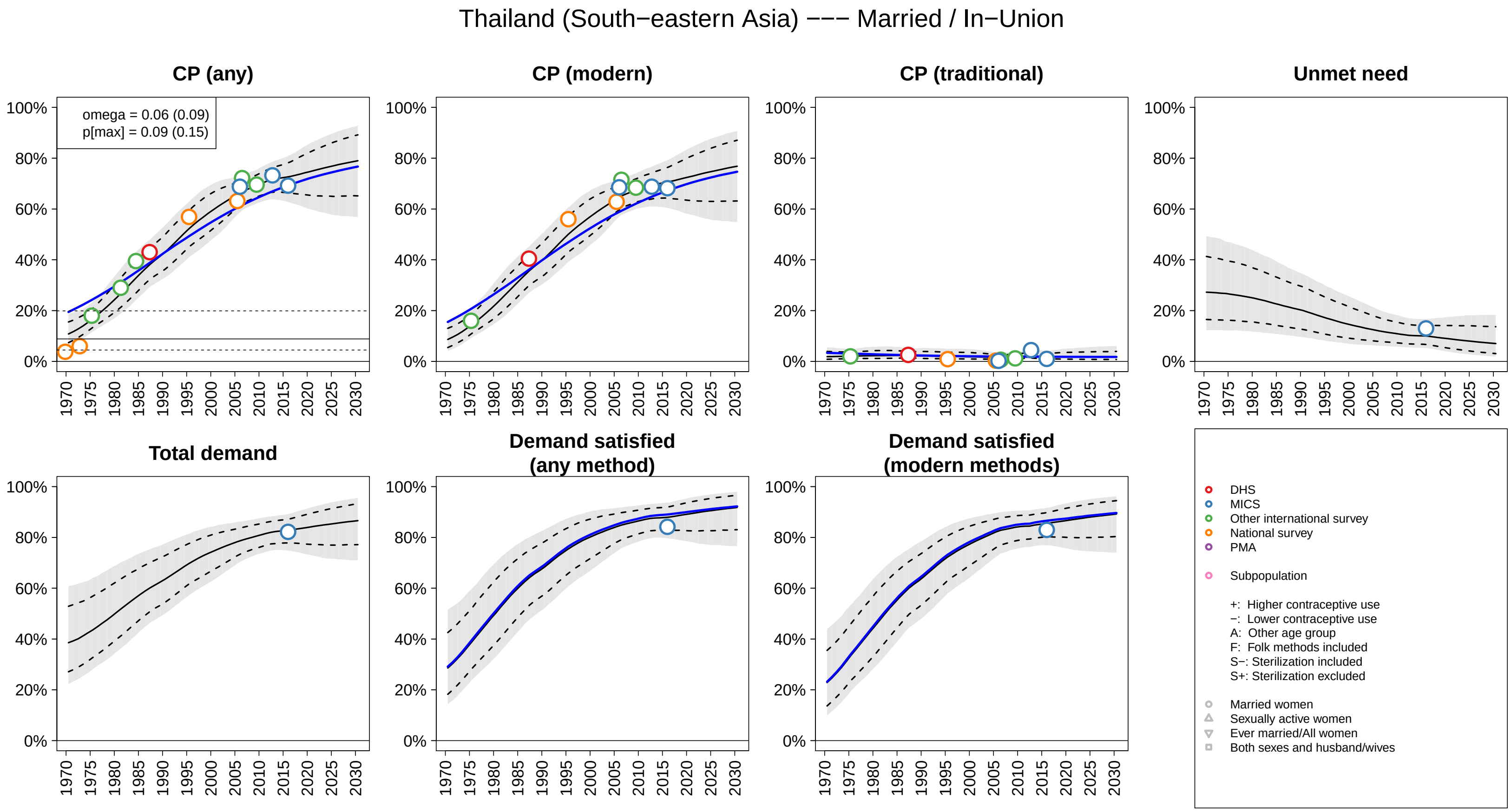
Thailand (South−eastern Asia) −−− Married / In−Union
CP (any)
omega = 0.06 (0.09)
p[max] = 0.09 (0.15)
CP (modern)
CP (traditional)
Unmet need
Total demand
Demand satisfied (any method)
Demand satisfied (modern methods)
DHS
MICS
Other international survey
National survey
PMA
Subpopulation
+: Higher contraceptive use
−: Lower contraceptive use
A: Other age group
F: Folk methods included
S−: Sterilization included
S+: Sterilization excluded
Married women
Sexually active women
Ever married/All women
Both sexes and husband/wives

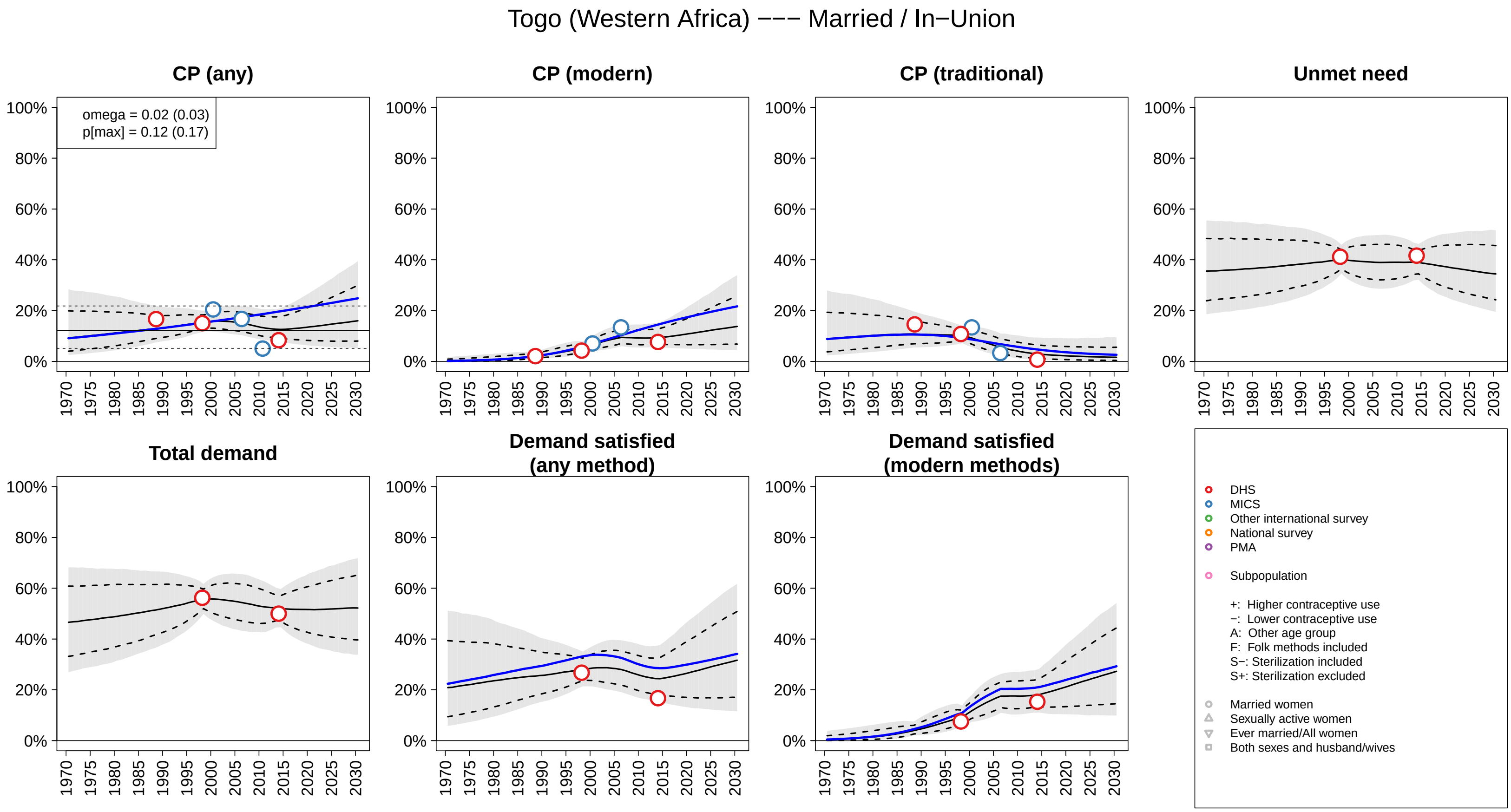

Togo (Western Africa) --- Married / In−Union
CP (any)
omega = 0.02 (0.03)
p[max] = 0.12 (0.17)
CP (modern)
CP (traditional)
Unmet need
Total demand
Demand satisfied (any method)
Demand satisfied (modern methods)
0%
20%
40%
60%
80%
100%
1970
1975
1980
1985
1990
1995
2000
2005
2010
2015
2020
2025
2030
DHS
MICS
Other international survey
National survey
PMA
Subpopulation
+: Higher contraceptive use
−: Lower contraceptive use
A: Other age group
F: Folk methods included
S−: Sterilization included
S+: Sterilization excluded
Married women
Sexually active women
Ever married/All women
Both sexes and husband/wives

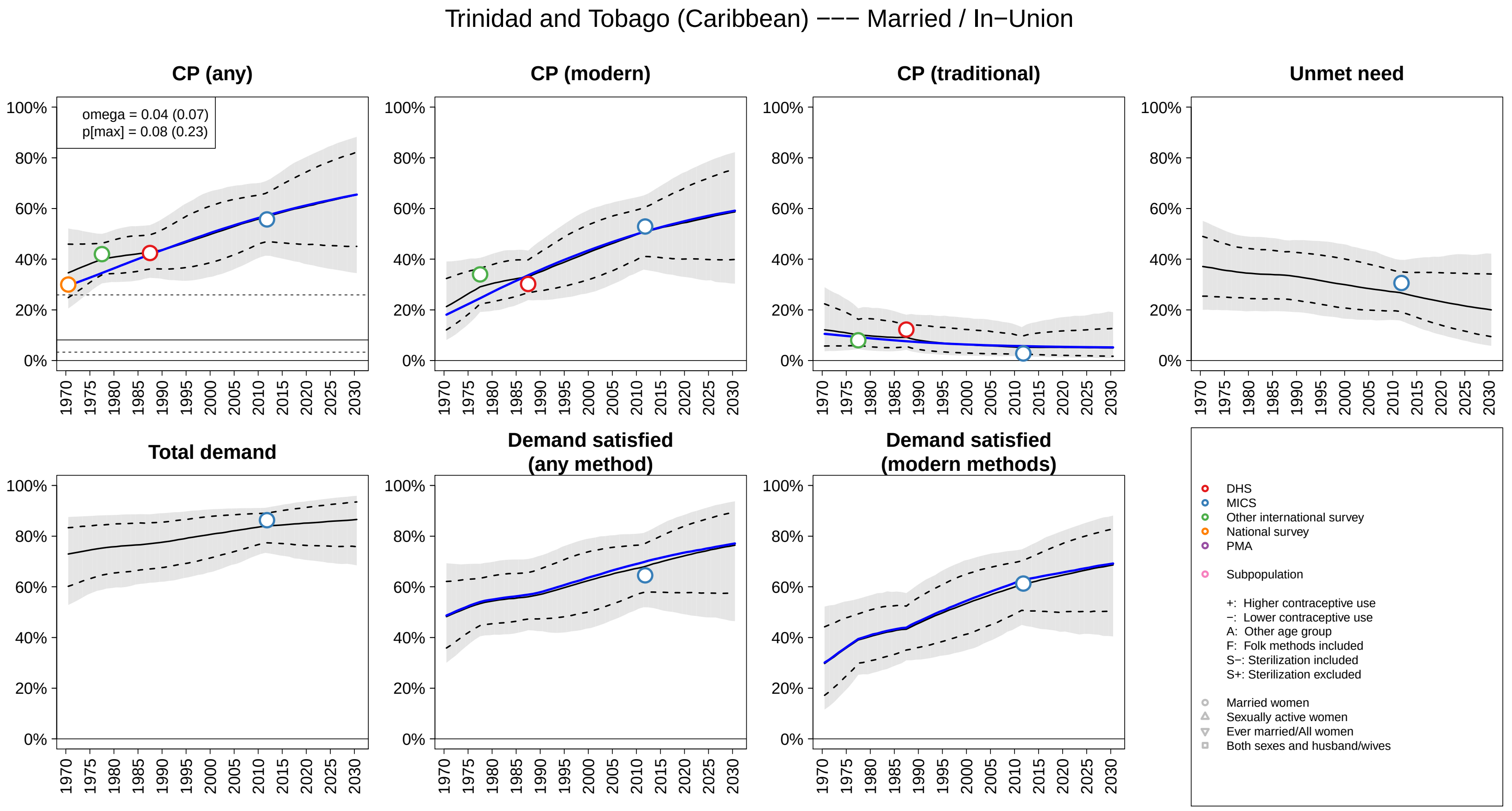
Trinidad and Tobago (Caribbean) ––– Married / In–Union
CP (any)
omega = 0.04 (0.07)
p[max] = 0.08 (0.23)
CP (modern)
CP (traditional)
Unmet need
Total demand
Demand satisfied (any method)
Demand satisfied (modern methods)
DHS
MICS
Other international survey
National survey
PMA
Subpopulation
+: Higher contraceptive use
−: Lower contraceptive use
A: Other age group
F: Folk methods included
S−: Sterilization included
S+: Sterilization excluded
Married women
Sexually active women
Ever married/All women
Both sexes and husband/wives

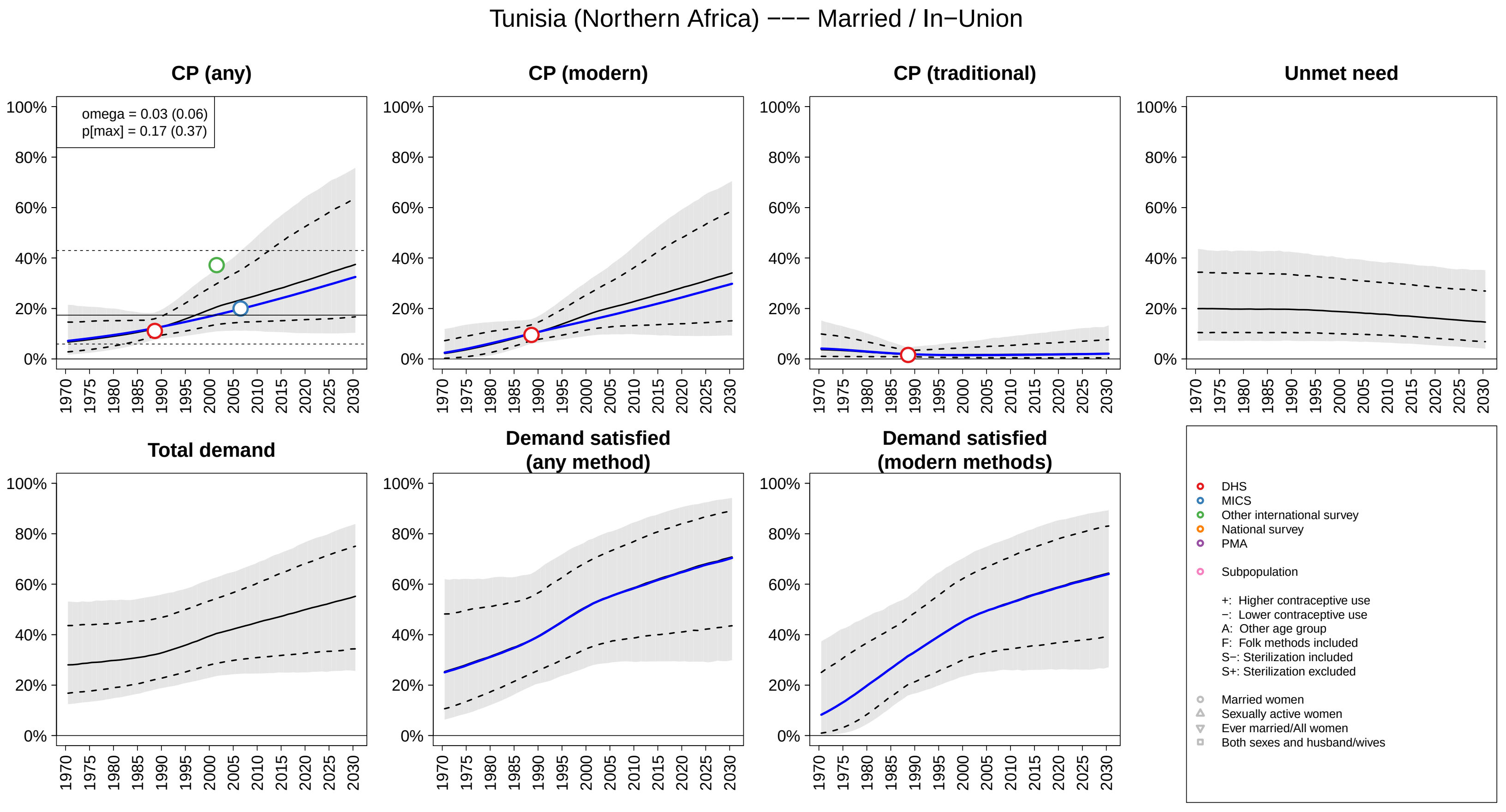
Tunisia (Northern Africa) −−− Married / In−Union
CP (any)
omega = 0.03 (0.06)
p[max] = 0.17 (0.37)
CP (modern)
CP (traditional)
Unmet need
Total demand
Demand satisfied (any method)
Demand satisfied (modern methods)
100%
80%
60%
40%
20%
0%
1970 1975 1980 1985 1990 1995 2000 2005 2010 2015 2020 2025 2030
DHS
MICS
Other international survey
National survey
PMA
Subpopulation
+: Higher contraceptive use
−: Lower contraceptive use
A: Other age group
F: Folk methods included
S−: Sterilization included
S+: Sterilization excluded
Married women
Sexually active women
Ever married/All women
Both sexes and husband/wives

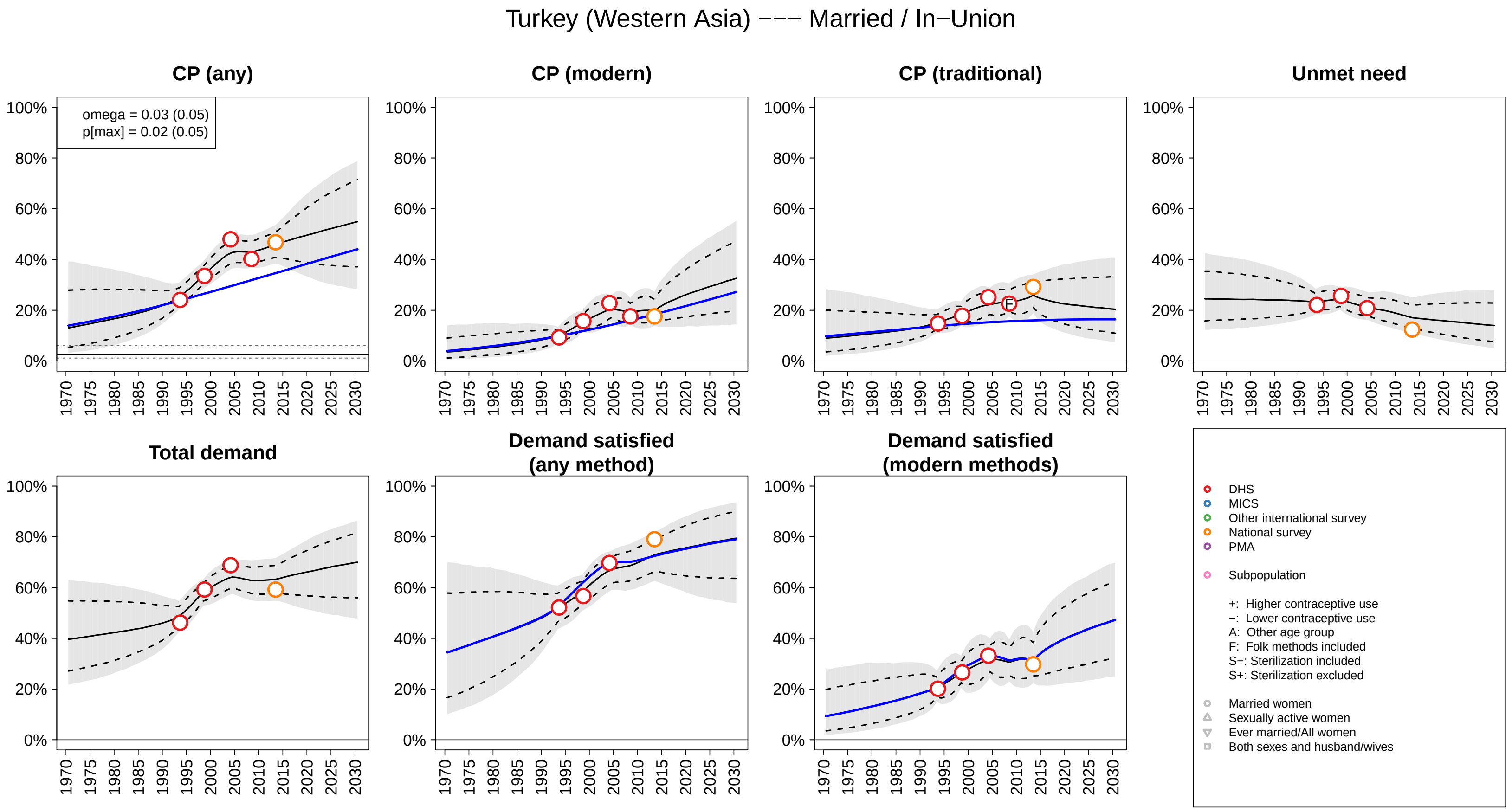
Turkey (Western Asia) --- Married / In−Union
CP (any)
omega = 0.03 (0.05)
p[max] = 0.02 (0.05)
CP (modern)
CP (traditional)
Unmet need
Total demand
Demand satisfied (any method)
Demand satisfied (modern methods)
DHS
MICS
Other international survey
National survey
PMA
Subpopulation
+: Higher contraceptive use
−: Lower contraceptive use
A: Other age group
F: Folk methods included
S−: Sterilization included
S+: Sterilization excluded
Married women
Sexually active women
Ever married/All women
Both sexes and husband/wives

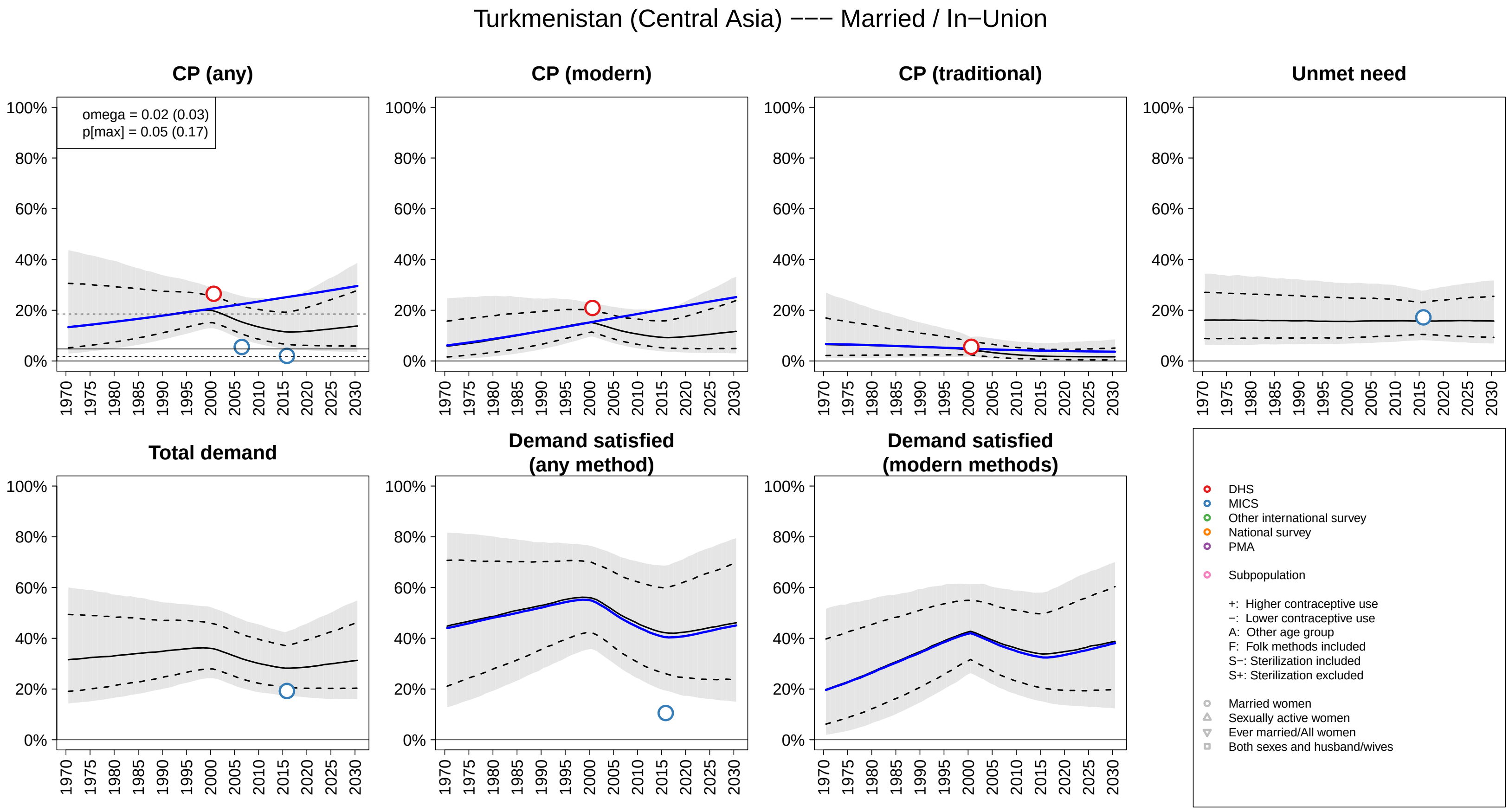
Turkmenistan (Central Asia) −−− Married / In−Union
CP (any)
omega = 0.02 (0.03)
p[max] = 0.05 (0.17)
CP (modern)
CP (traditional)
Unmet need
Total demand
Demand satisfied (any method)
Demand satisfied (modern methods)
0%
20%
40%
60%
80%
100%
1970
1975
1980
1985
1990
1995
2000
2005
2010
2015
2020
2025
2030
DHS
MICS
Other international survey
National survey
PMA
Subpopulation
+: Higher contraceptive use
−: Lower contraceptive use
A: Other age group
F: Folk methods included
S−: Sterilization included
S+: Sterilization excluded
Married women
Sexually active women
Ever married/All women
Both sexes and husband/wives

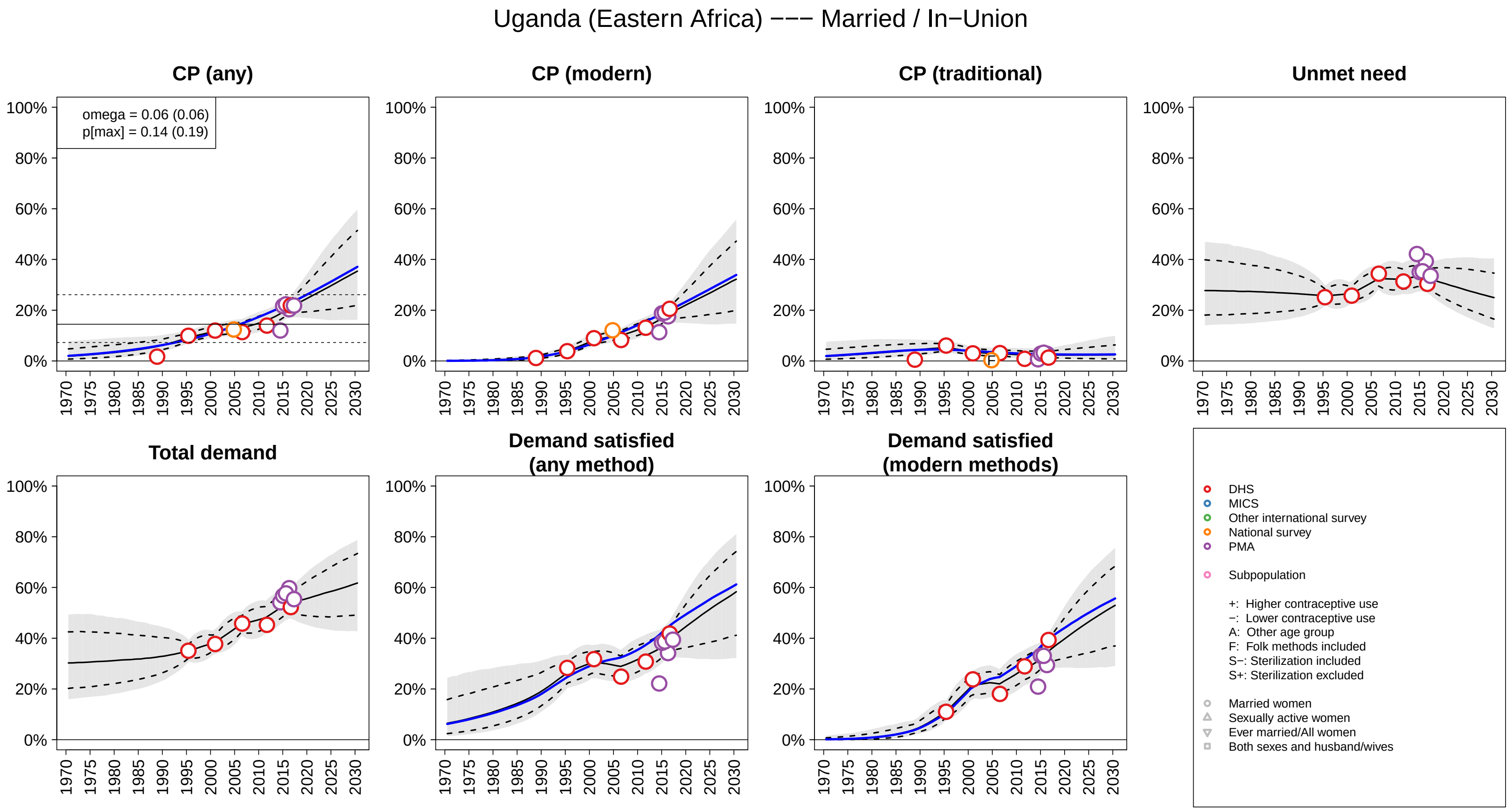
Uganda (Eastern Africa) ––– Married / In–Union
CP (any)
omega = 0.06 (0.06)
p[max] = 0.14 (0.19)
CP (modern)
CP (traditional)
Unmet need
Total demand
Demand satisfied (any method)
Demand satisfied (modern methods)
DHS
MICS
Other international survey
National survey
PMA
Subpopulation
+: Higher contraceptive use
−: Lower contraceptive use
A: Other age group
F: Folk methods included
S−: Sterilization included
S+: Sterilization excluded
Married women
Sexually active women
Ever married/All women
Both sexes and husband/wives

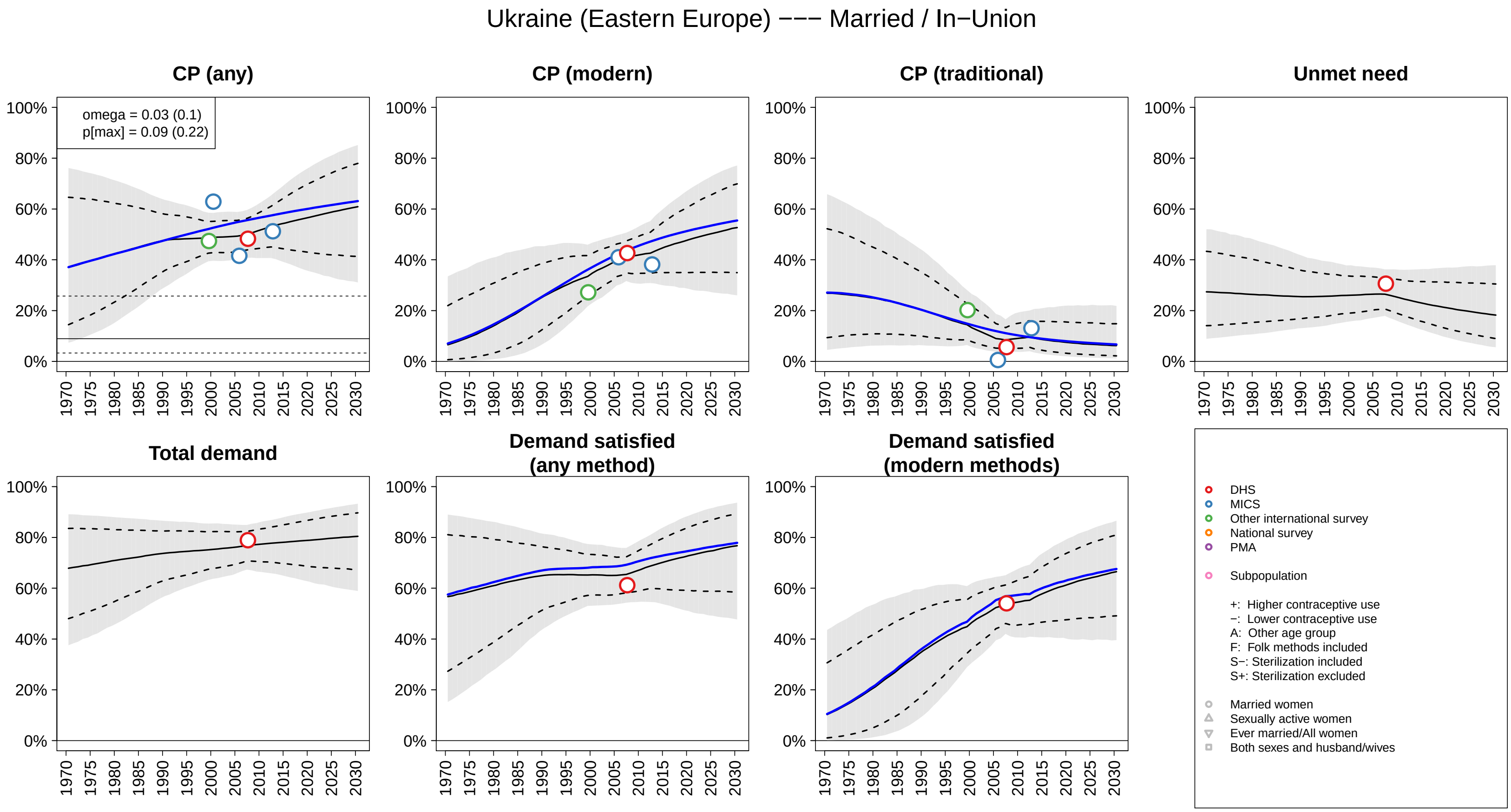

Ukraine (Eastern Europe) --- Married / In−Union
CP (any)
omega = 0.03 (0.1)
p[max] = 0.09 (0.22)
CP (modern)
CP (traditional)
Unmet need
Total demand
Demand satisfied (any method)
Demand satisfied (modern methods)
100%
80%
60%
40%
20%
0%
1970
1975
1980
1985
1990
1995
2000
2005
2010
2015
2020
2025
2030
DHS
MICS
Other international survey
National survey
PMA
Subpopulation
+: Higher contraceptive use
−: Lower contraceptive use
A: Other age group
F: Folk methods included
S−: Sterilization included
S+: Sterilization excluded
Married women
Sexually active women
Ever married/All women
Both sexes and husband/wives

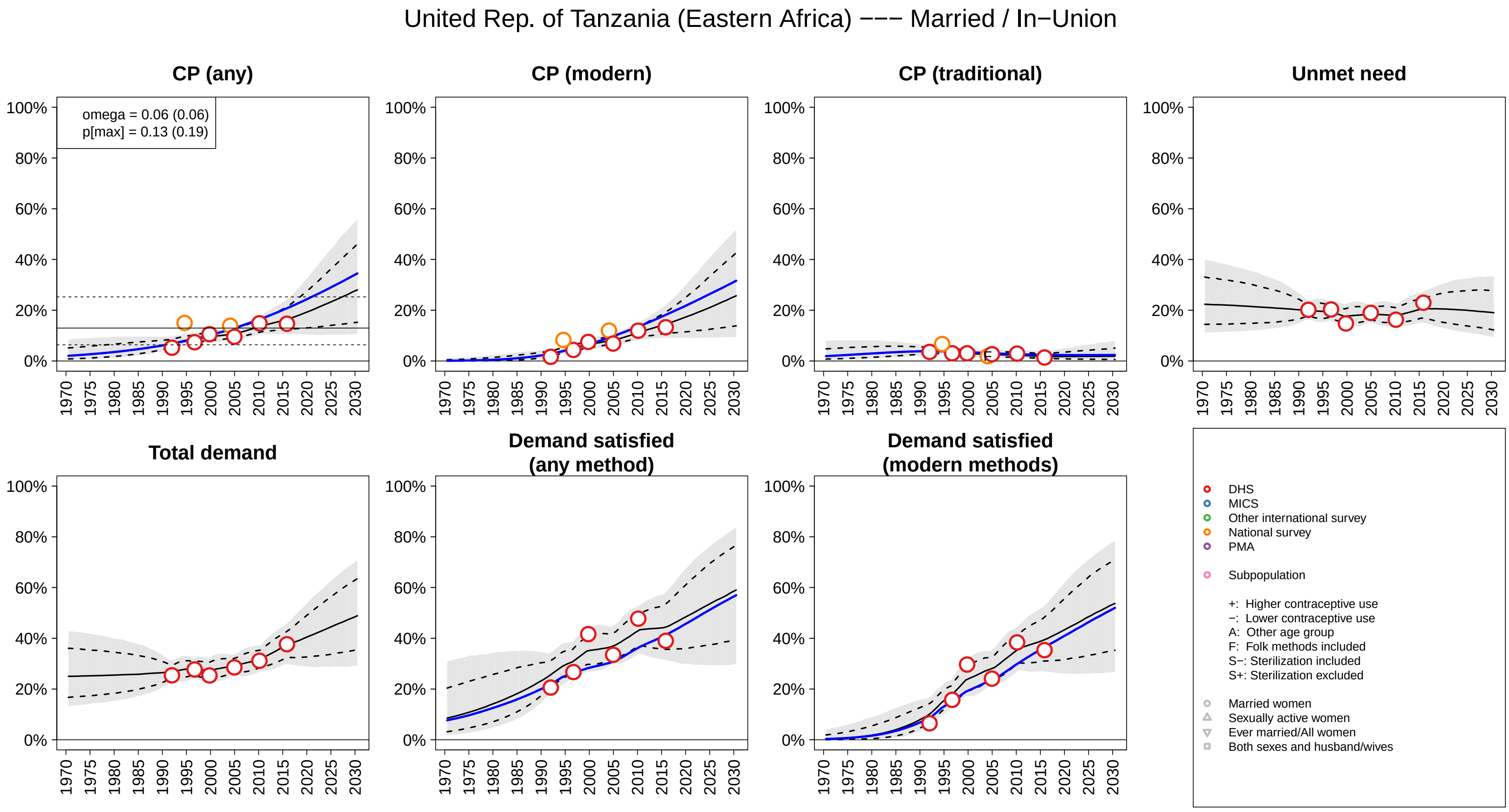
United Rep. of Tanzania (Eastern Africa) −−− Married / In−Union
CP (any)
omega = 0.06 (0.06)
p[max] = 0.13 (0.19)
CP (modern)
CP (traditional)
Unmet need
Total demand
Demand satisfied (any method)
Demand satisfied (modern methods)
DHS
MICS
Other international survey
National survey
PMA
Subpopulation
+: Higher contraceptive use
−: Lower contraceptive use
A: Other age group
F: Folk methods included
S−: Sterilization included
S+: Sterilization excluded
Married women
Sexually active women
Ever married/All women
Both sexes and husband/wives

United States of America (Northern America) ––– Married / In–Union

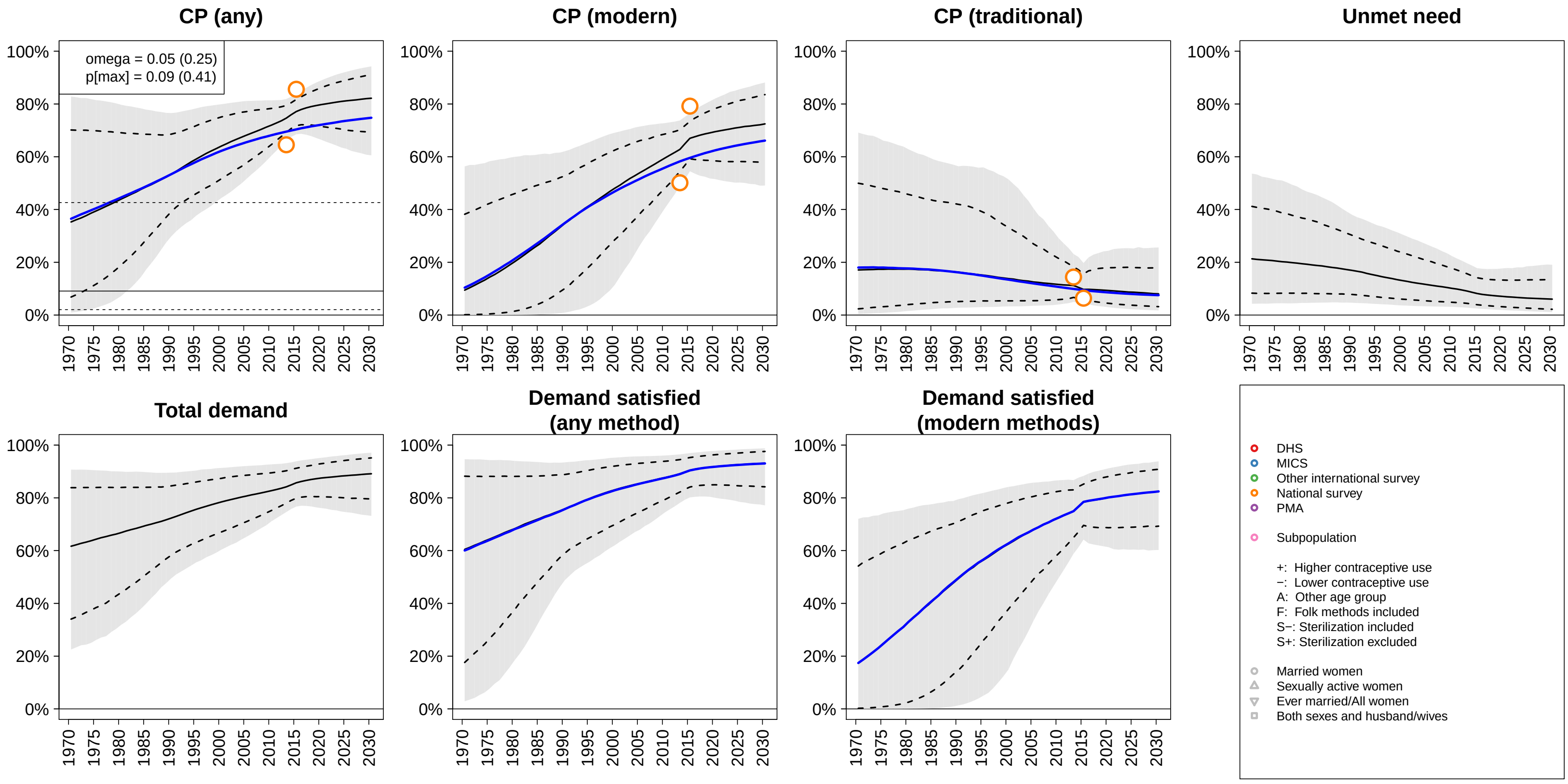

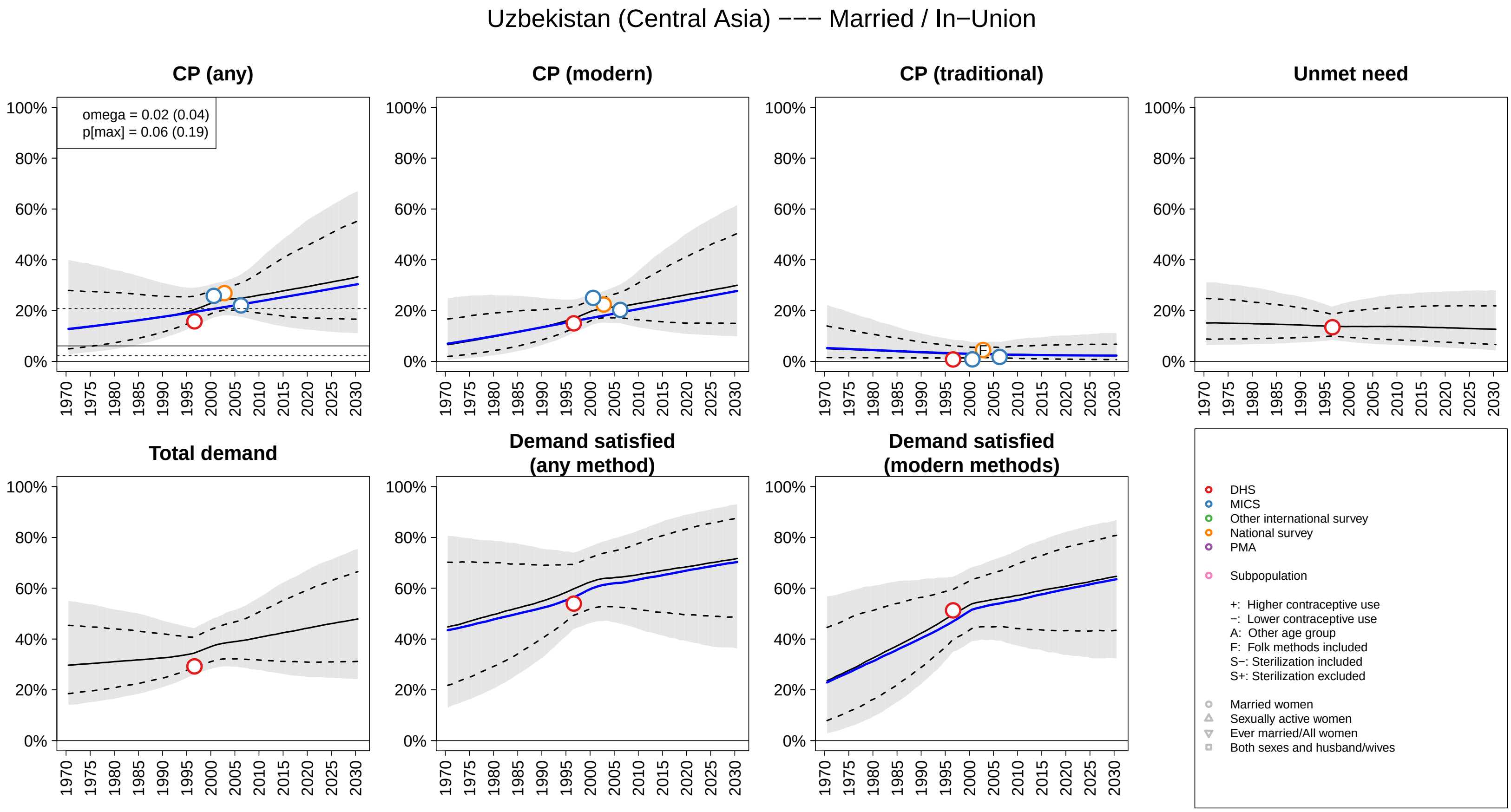
Uzbekistan (Central Asia) --- Married / In-Union
CP (any)
omega = 0.02 (0.04)
p[max] = 0.06 (0.19)
CP (modern)
CP (traditional)
Unmet need
Total demand
Demand satisfied (any method)
Demand satisfied (modern methods)
DHS
MICS
Other international survey
National survey
PMA
Subpopulation
+: Higher contraceptive use
−: Lower contraceptive use
A: Other age group
F: Folk methods included
S−: Sterilization included
S+: Sterilization excluded
Married women
Sexually active women
Ever married/All women
Both sexes and husband/wives

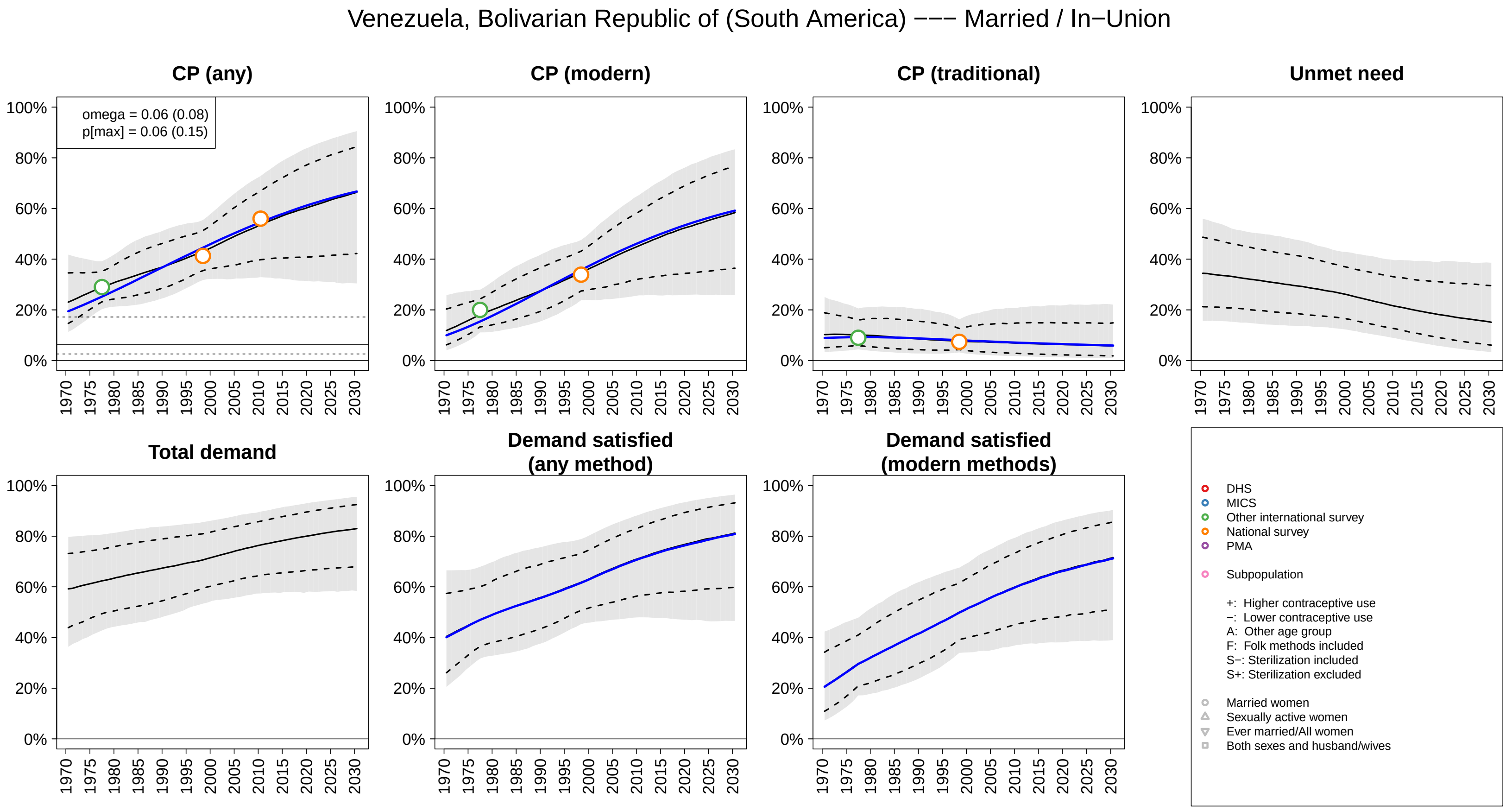
Venezuela, Bolivarian Republic of (South America) −−− Married / In−Union
CP (any)
omega = 0.06 (0.08)
p[max] = 0.06 (0.15)
CP (modern)
CP (traditional)
Unmet need
Total demand
Demand satisfied (any method)
Demand satisfied (modern methods)
DHS
MICS
Other international survey
National survey
PMA
Subpopulation
+: Higher contraceptive use
−: Lower contraceptive use
A: Other age group
F: Folk methods included
S−: Sterilization included
S+: Sterilization excluded
Married women
Sexually active women
Ever married/All women
Both sexes and husband/wives

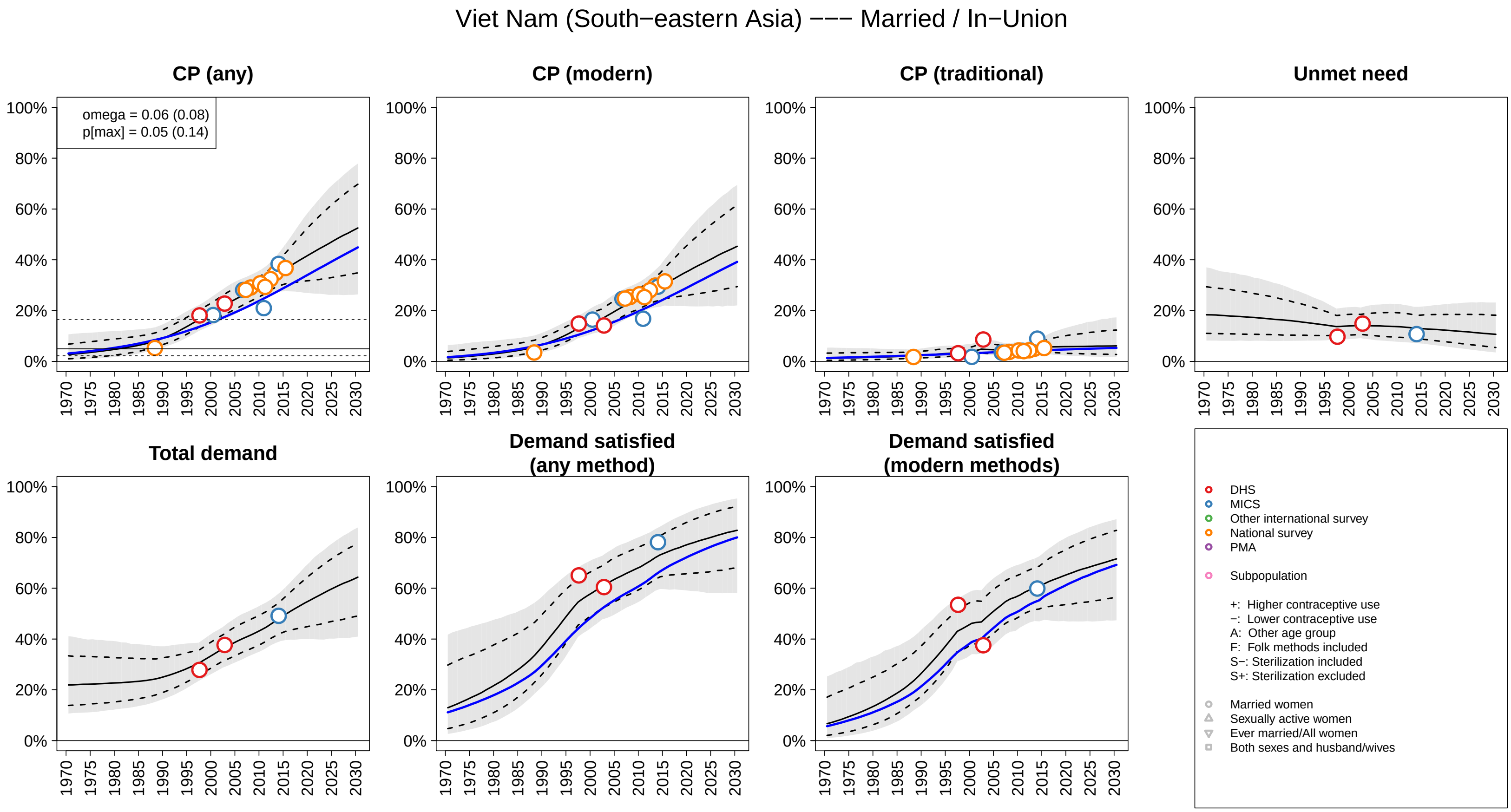
Viet Nam (South−eastern Asia) −−− Married / In−Union
CP (any)
omega = 0.06 (0.08)
p[max] = 0.05 (0.14)
CP (modern)
CP (traditional)
Unmet need
Total demand
Demand satisfied (any method)
Demand satisfied (modern methods)
DHS
MICS
Other international survey
National survey
PMA
Subpopulation
+: Higher contraceptive use
−: Lower contraceptive use
A: Other age group
F: Folk methods included
S−: Sterilization included
S+: Sterilization excluded
Married women
Sexually active women
Ever married/All women
Both sexes and husband/wives

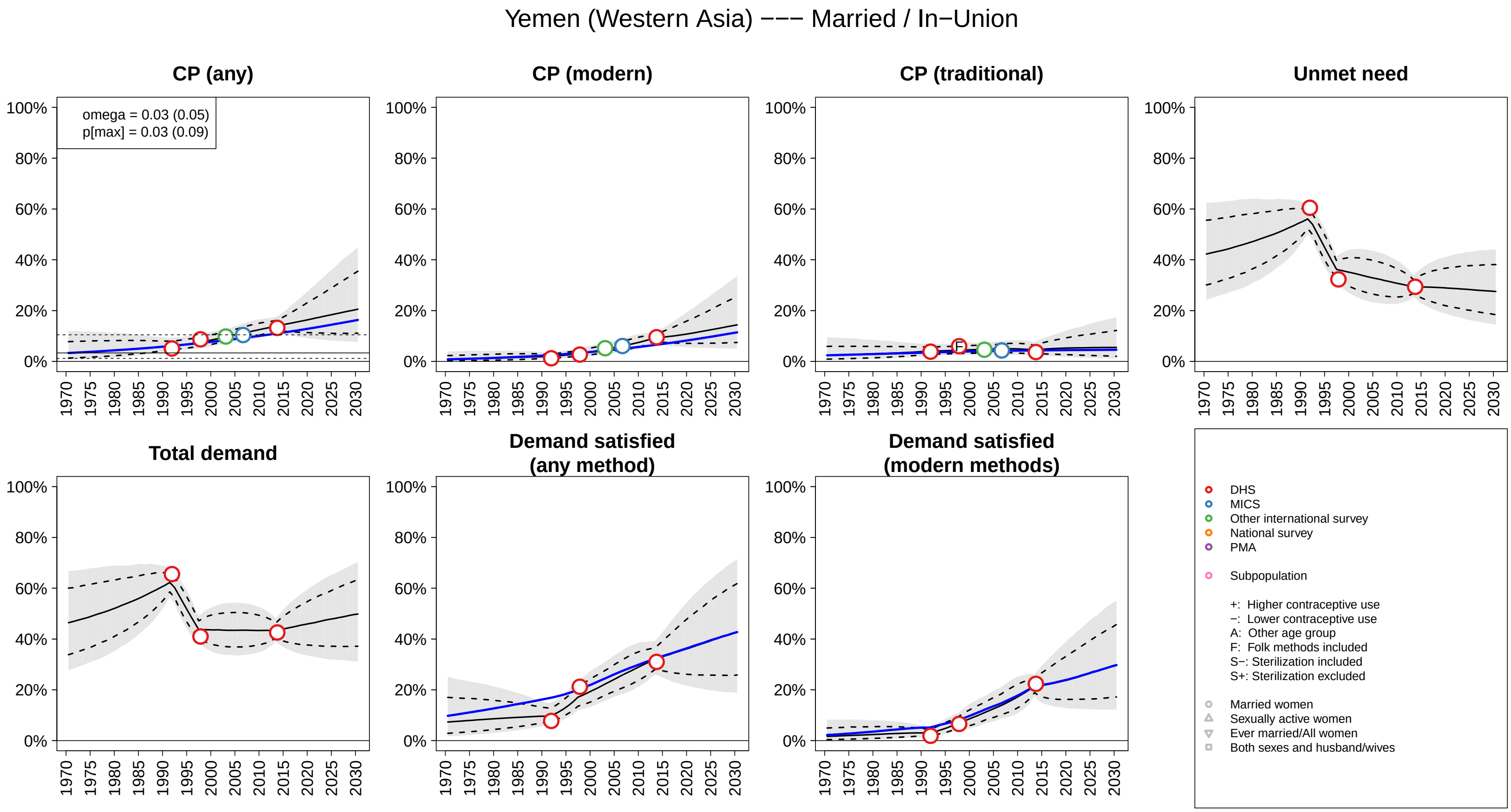
Yemen (Western Asia) ––– Married / In–Union
CP (any)
omega = 0.03 (0.05)
p[max] = 0.03 (0.09)
CP (modern)
CP (traditional)
Unmet need
Total demand
Demand satisfied (any method)
Demand satisfied (modern methods)
0%
20%
40%
60%
80%
100%
1970
1975
1980
1985
1990
1995
2000
2005
2010
2015
2020
2025
2030
F
DHS
MICS
Other international survey
National survey
PMA
Subpopulation
+: Higher contraceptive use
−: Lower contraceptive use
A: Other age group
F: Folk methods included
S−: Sterilization included
S+: Sterilization excluded
Married women
Sexually active women
Ever married/All women
Both sexes and husband/wives

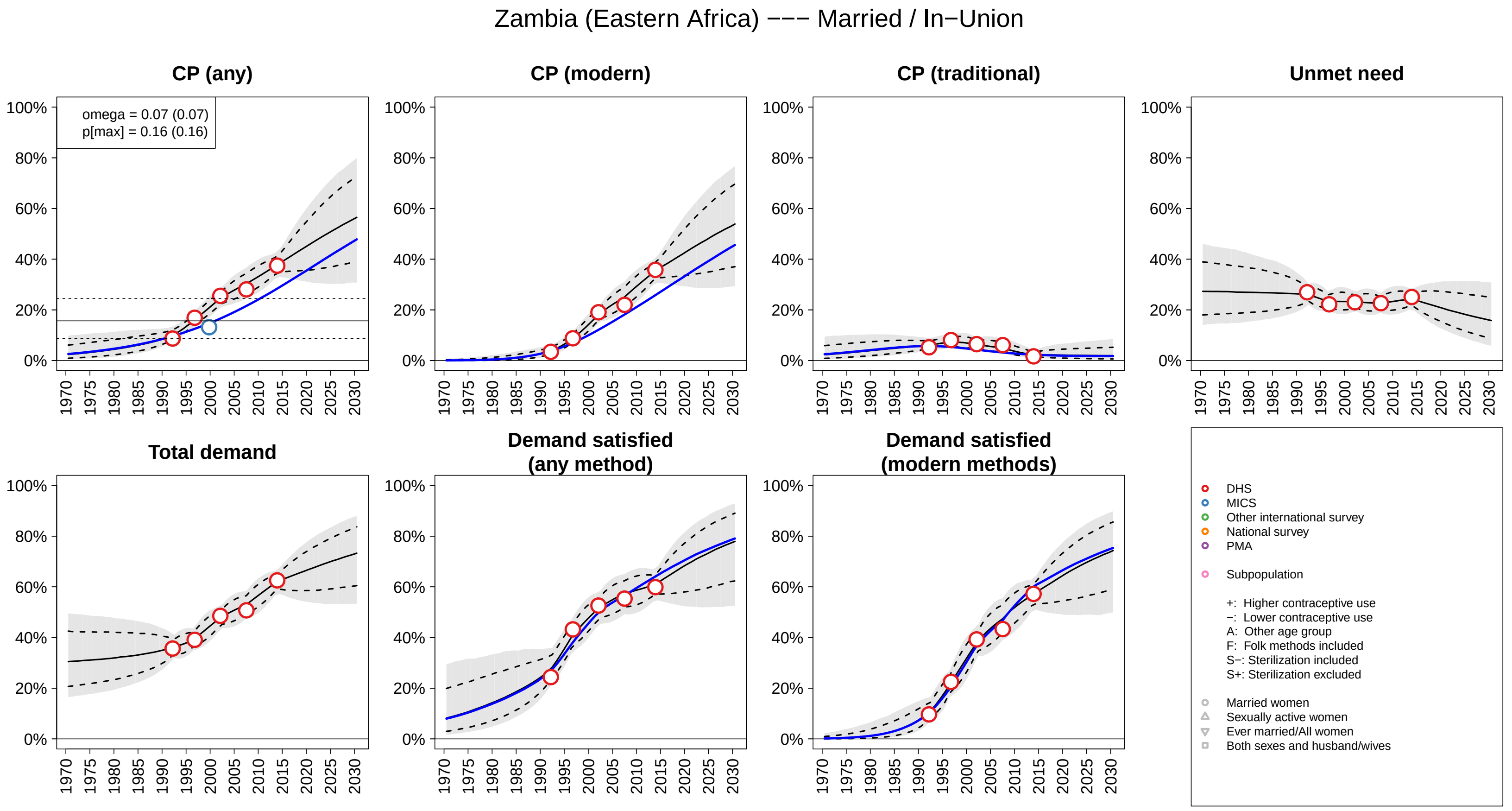
Zambia (Eastern Africa) −−− Married / In−Union
CP (any)
omega = 0.07 (0.07)
p[max] = 0.16 (0.16)
CP (modern)
CP (traditional)
Unmet need
Total demand
Demand satisfied (any method)
Demand satisfied (modern methods)
DHS
MICS
Other international survey
National survey
PMA
Subpopulation
+: Higher contraceptive use
−: Lower contraceptive use
A: Other age group
F: Folk methods included
S−: Sterilization included
S+: Sterilization excluded
Married women
Sexually active women
Ever married/All women
Both sexes and husband/wives

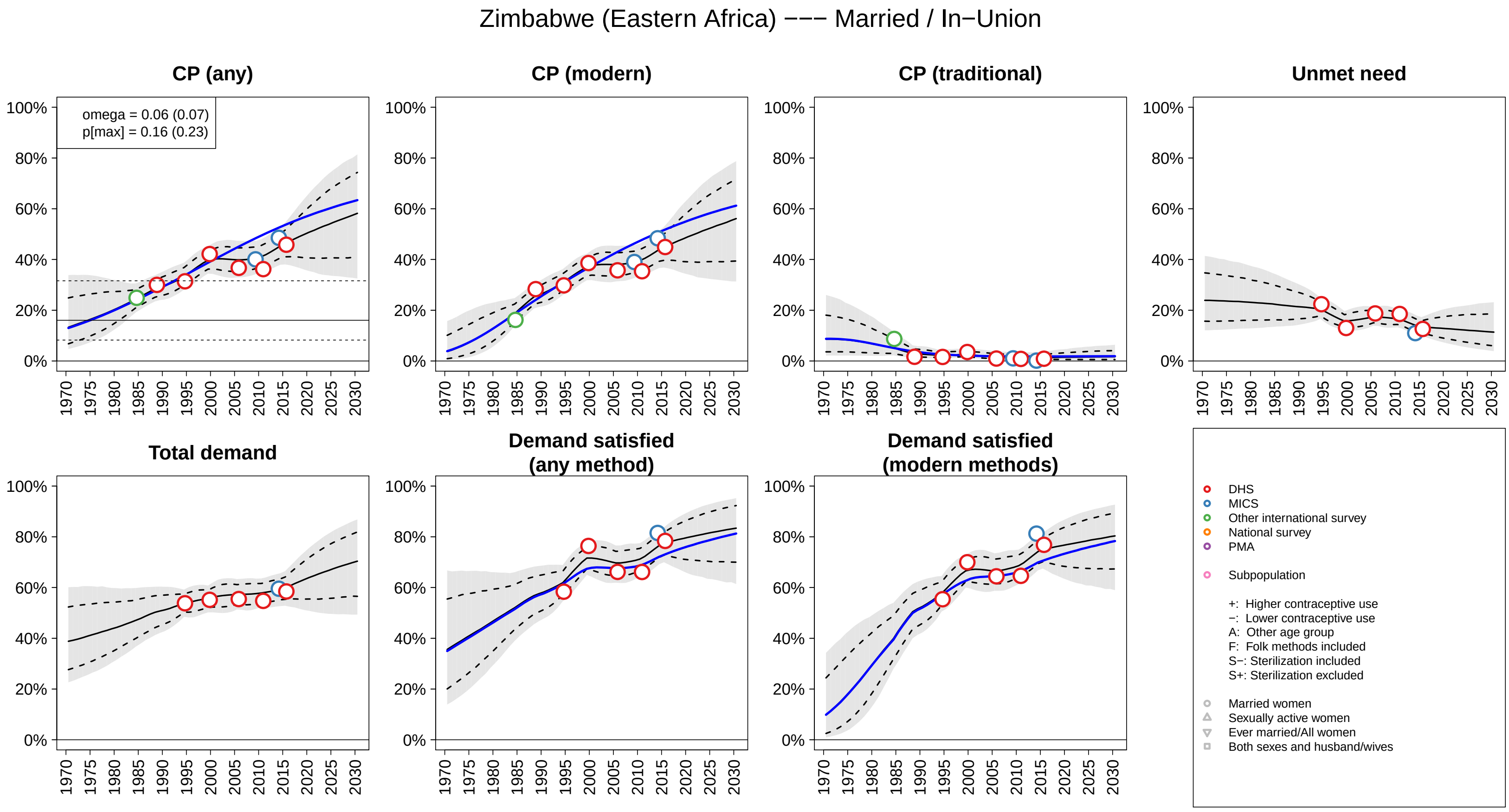
Zimbabwe (Eastern Africa) --- Married / In−Union
CP (any)
omega = 0.06 (0.07)
p[max] = 0.16 (0.23)
CP (modern)
CP (traditional)
Unmet need
Total demand
Demand satisfied (any method)
Demand satisfied (modern methods)
DHS
MICS
Other international survey
National survey
PMA
Subpopulation
+: Higher contraceptive use
−: Lower contraceptive use
A: Other age group
F: Folk methods included
S−: Sterilization included
S+: Sterilization excluded
Married women
Sexually active women
Ever married/All women
Both sexes and husband/wives

4.3 All Adolescent Women

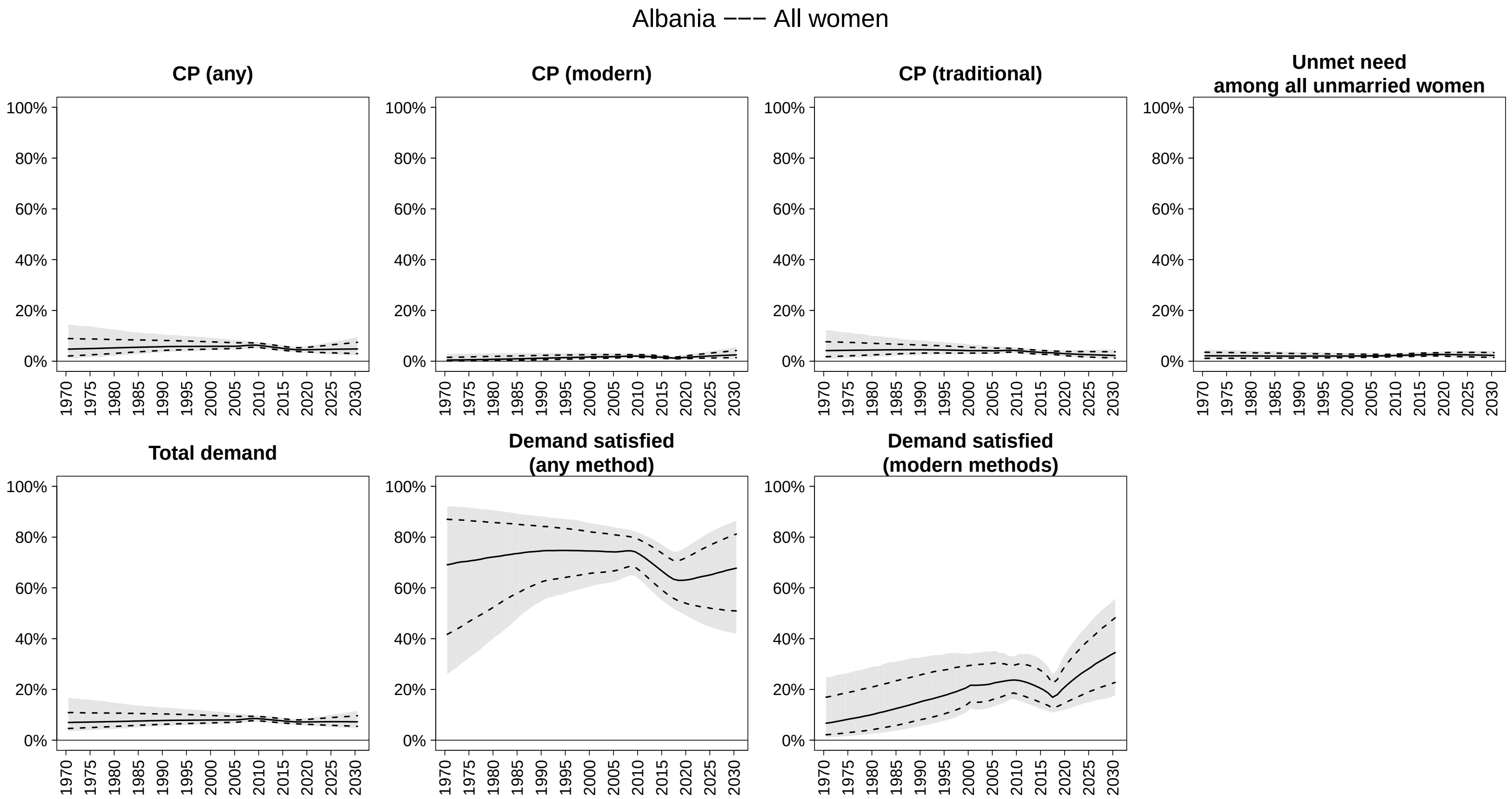
Albania −−− All women
CP (any)
CP (modern)
CP (traditional)
Unmet need
among all unmarried women
Total demand
Demand satisfied
(any method)
Demand satisfied
(modern methods)
0%
20%
40%
60%
80%
100%
1970
1975
1980
1985
1990
1995
2000
2005
2010
2015
2020
2025
2030

Angola −−− All women

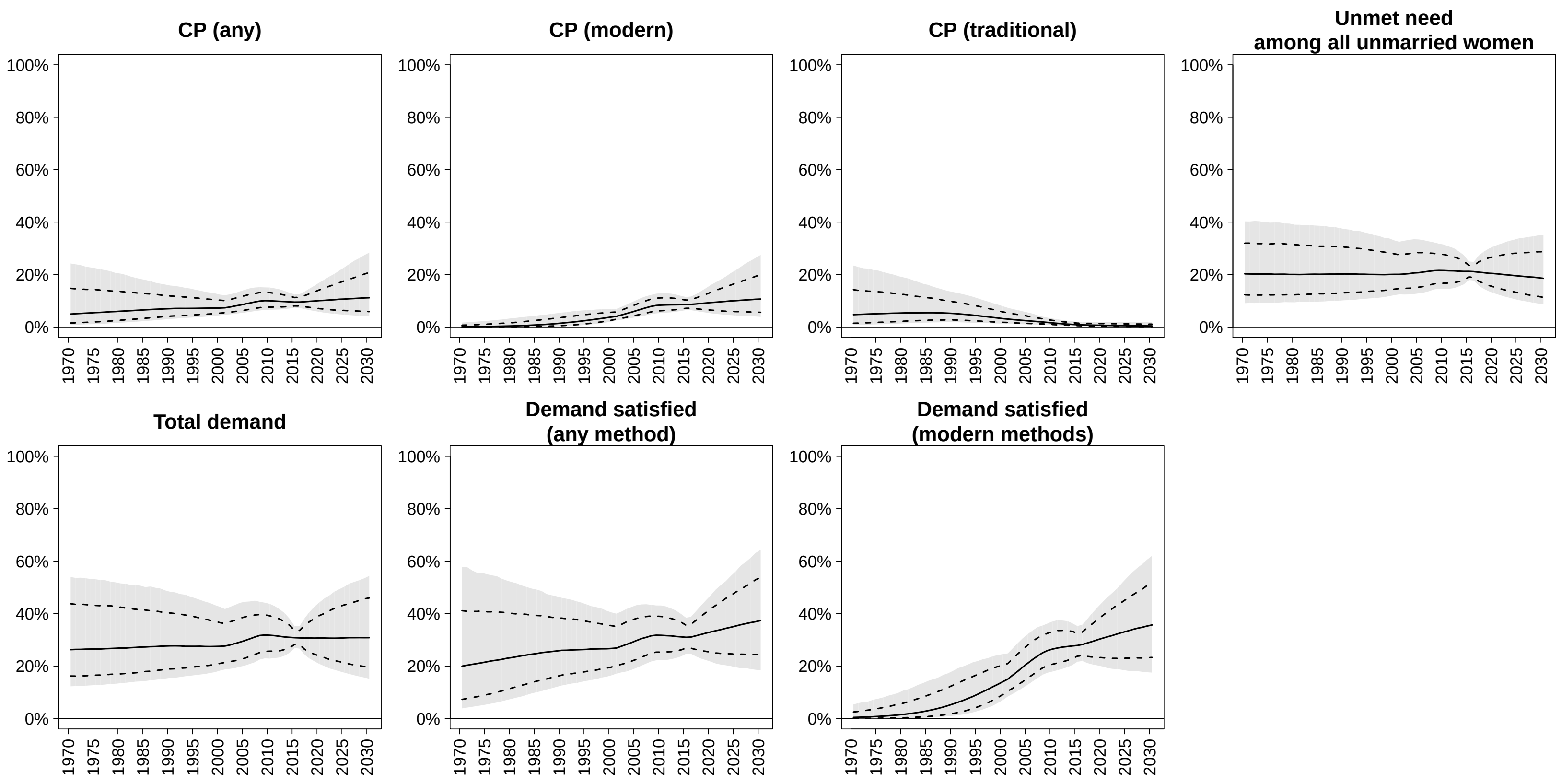

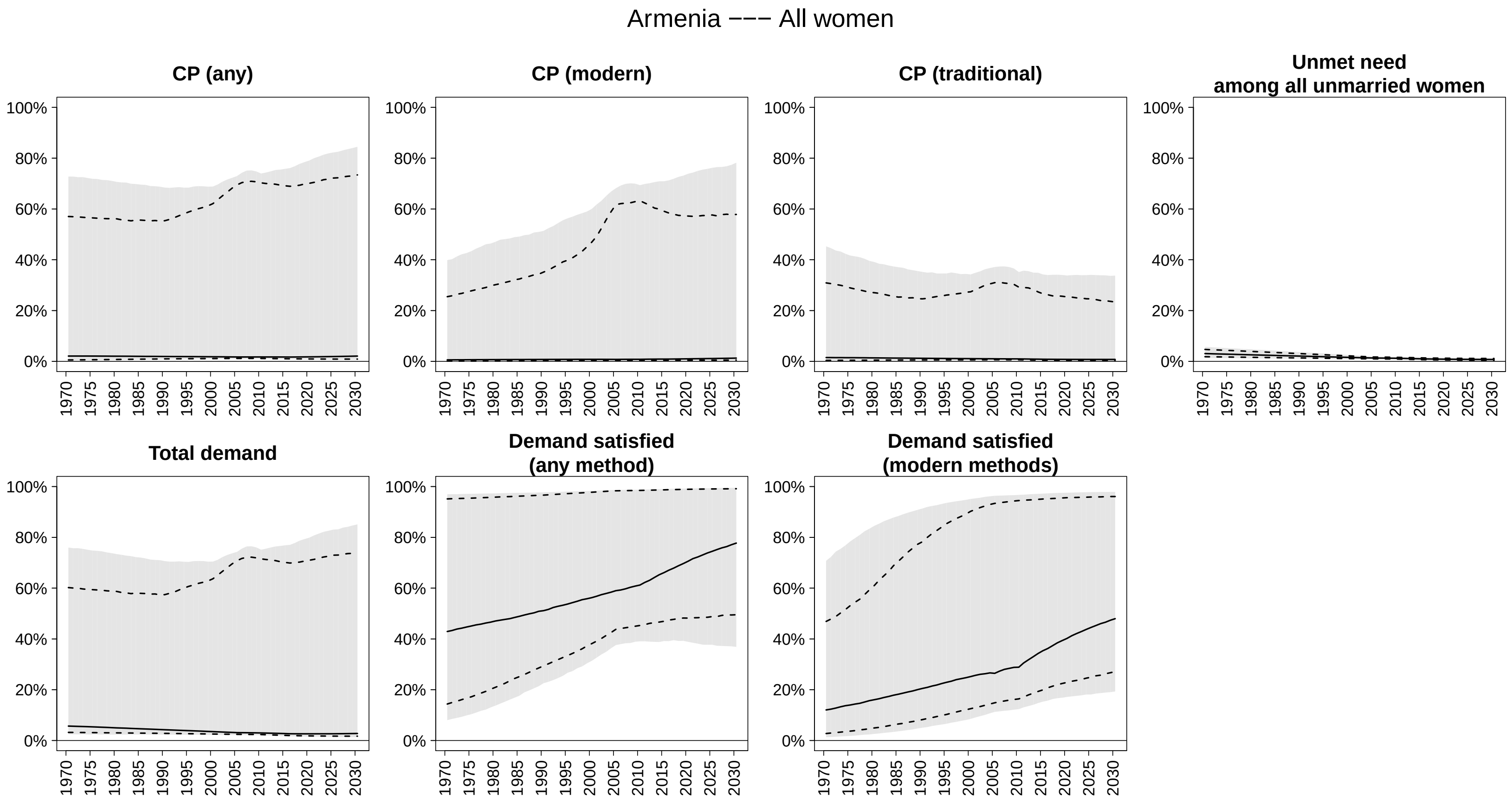
Armenia --- All women
CP (any)
CP (modern)
CP (traditional)
Unmet need among all unmarried women
Total demand
Demand satisfied (any method)
Demand satisfied (modern methods)
0%
20%
40%
60%
80%
100%
1970
1975
1980
1985
1990
1995
2000
2005
2010
2015
2020
2025
2030

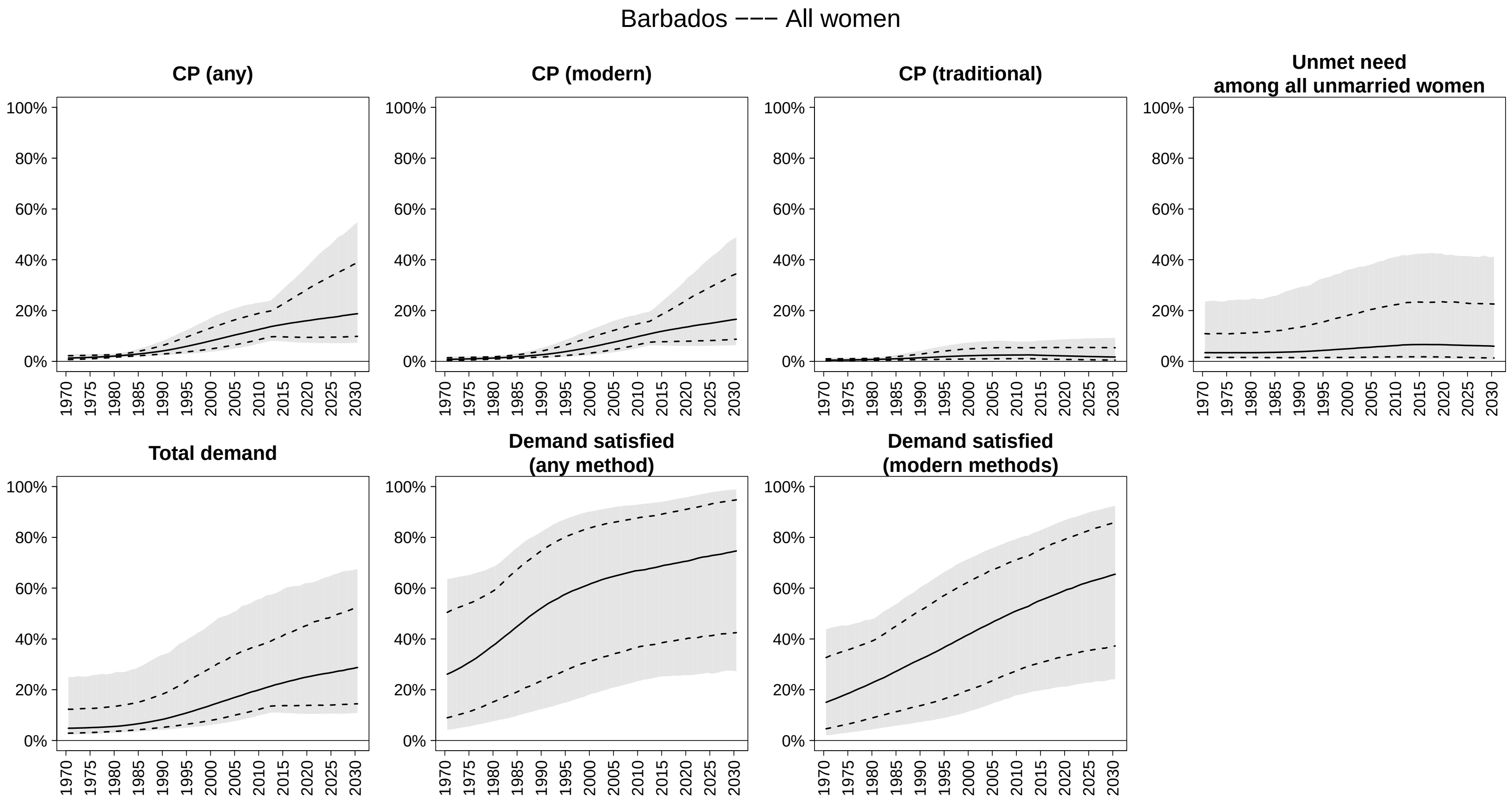
Barbados −−− All women
CP (any)
CP (modern)
CP (traditional)
Unmet need
among all unmarried women
Total demand
Demand satisfied
(any method)
Demand satisfied
(modern methods)
0%
20%
40%
60%
80%
100%
1970
1975
1980
1985
1990
1995
2000
2005
2010
2015
2020
2025
2030

Benin −−− All women

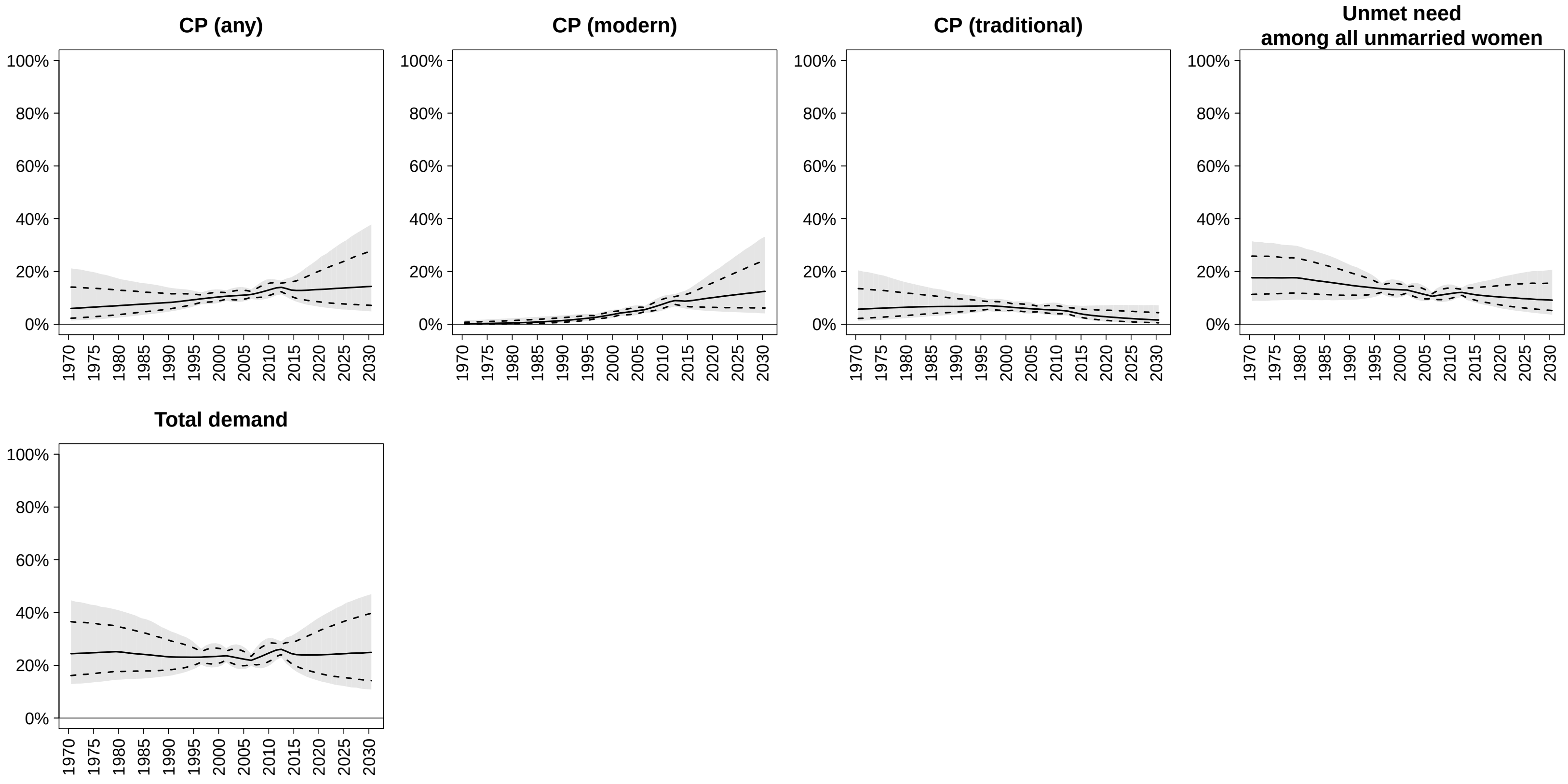

Bolivia (Plurinational State of) −−− All women

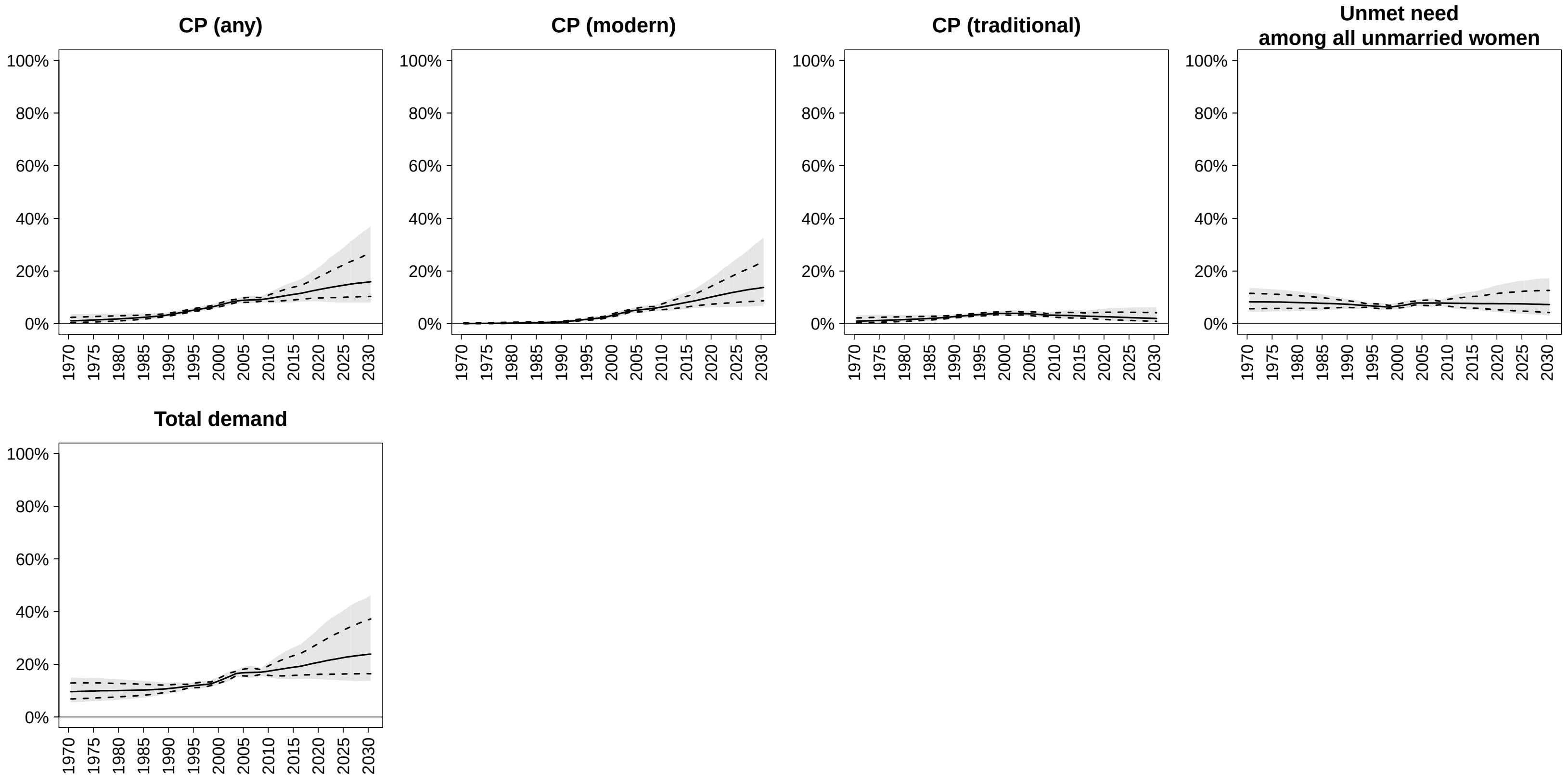

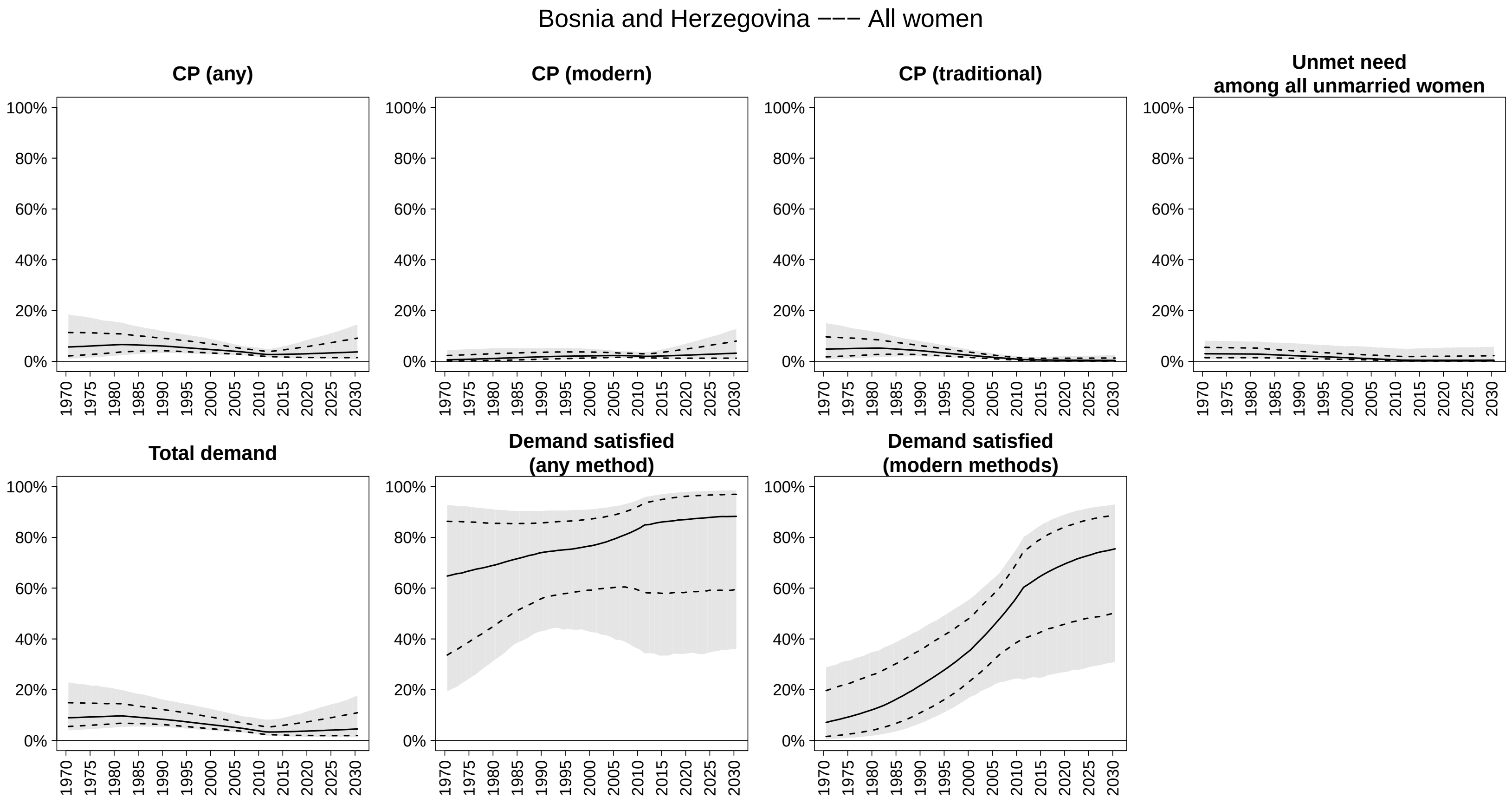
Bosnia and Herzegovina — All women
CP (any)
CP (modern)
CP (traditional)
Unmet need among all unmarried women
Total demand
Demand satisfied (any method)
Demand satisfied (modern methods)
0%
20%
40%
60%
80%
100%
1970
1975
1980
1985
1990
1995
2000
2005
2010
2015
2020
2025
2030

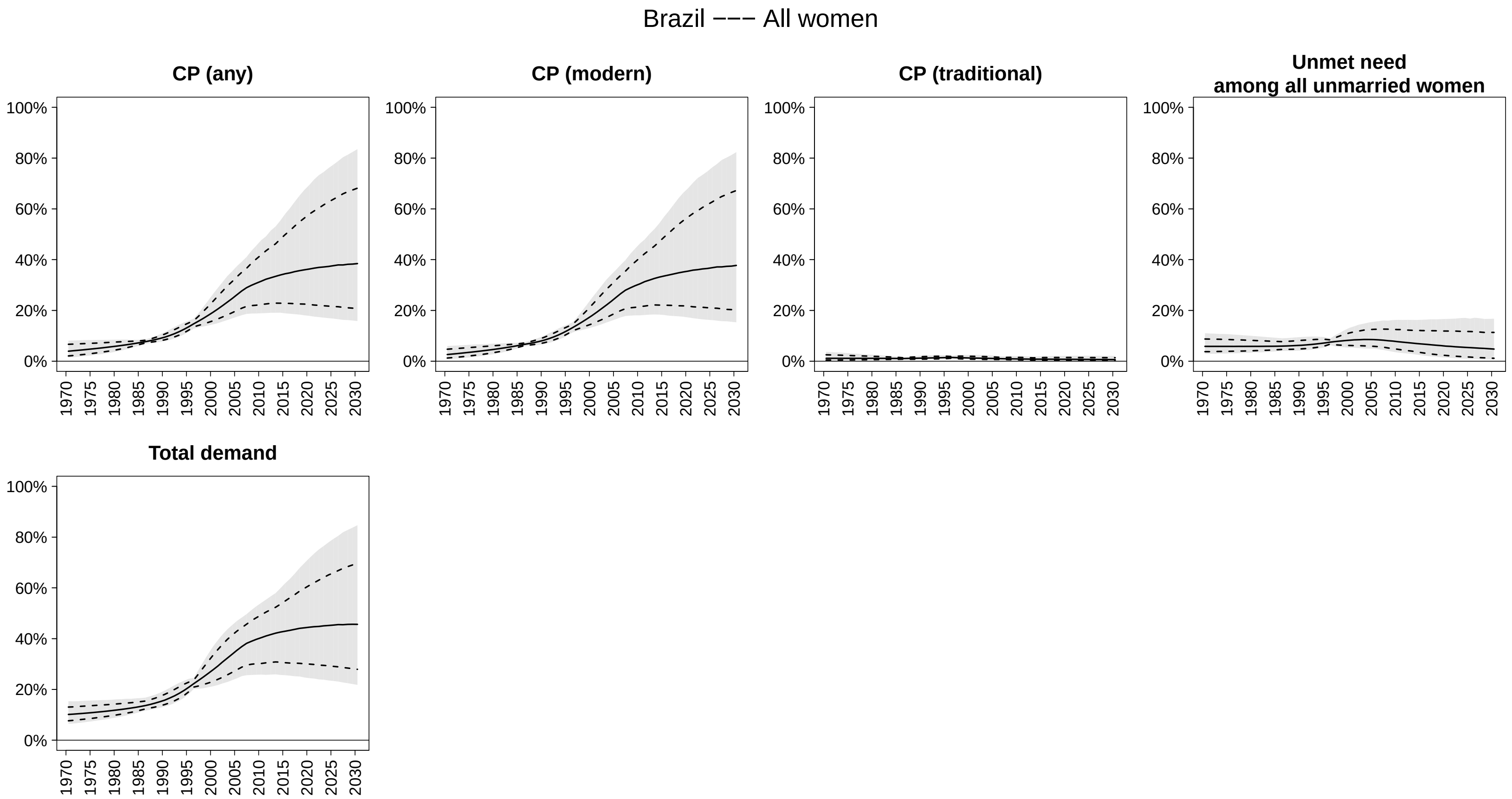
Brazil −−− All women
CP (any)
CP (modern)
CP (traditional)
Unmet need among all unmarried women
Total demand

Burkina Faso −−− All women

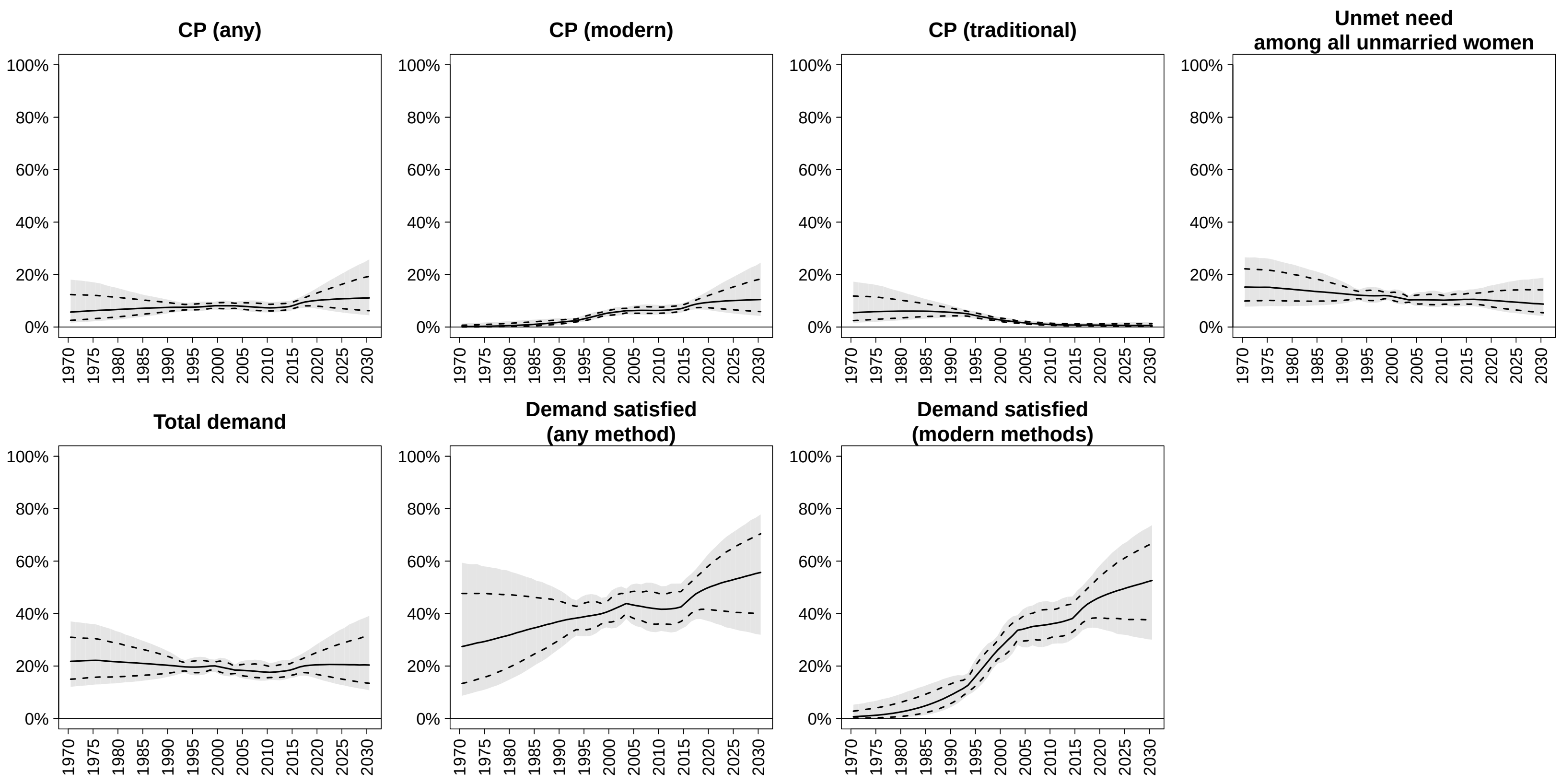

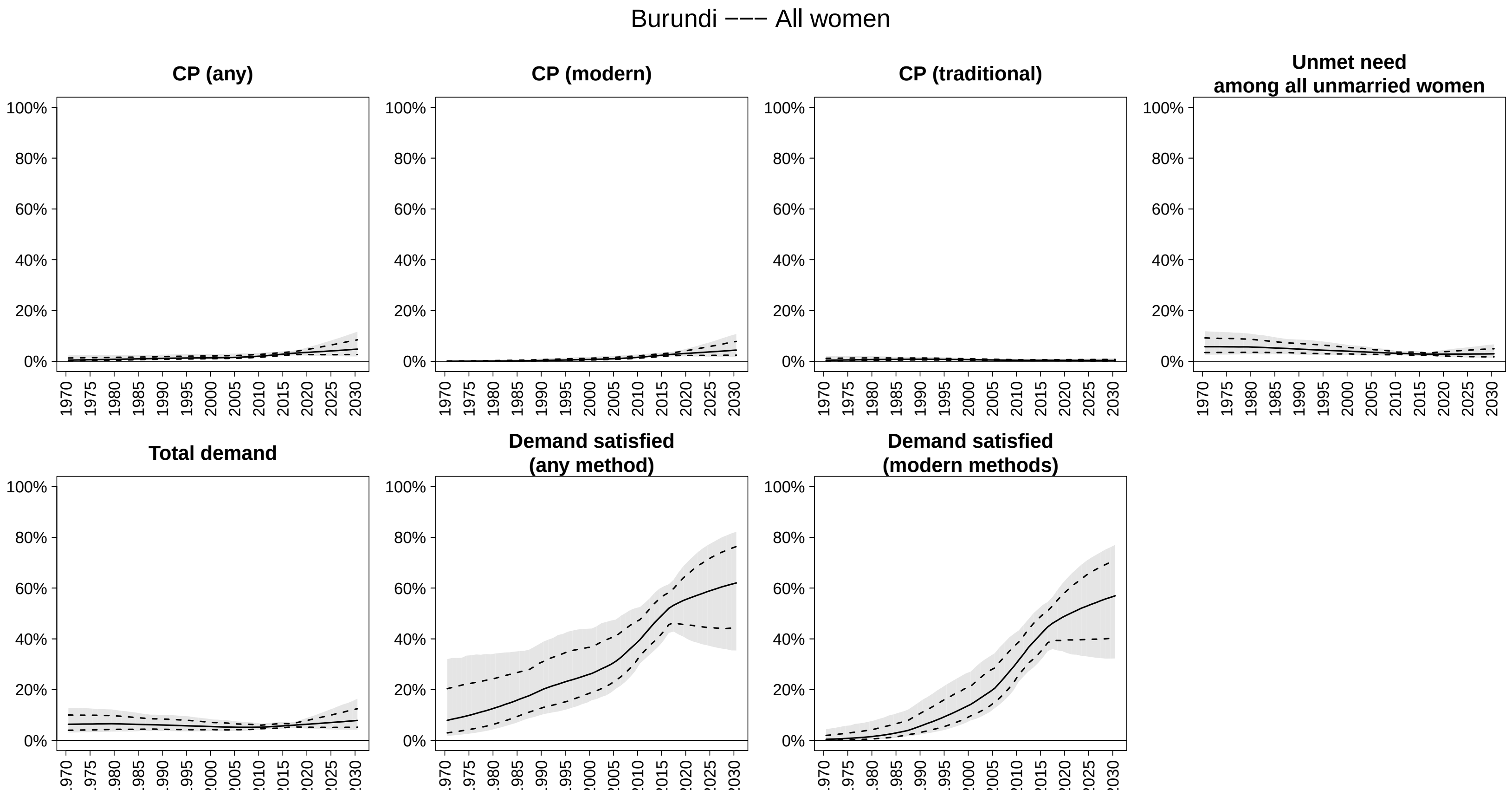
Burundi −−− All women
CP (any)
CP (modern)
CP (traditional)
Unmet need
among all unmarried women
Total demand
Demand satisfied
(any method)
Demand satisfied
(modern methods)
0%
20%
40%
60%
80%
100%
1970
1975
1980
1985
1990
1995
2000
2005
2010
2015
2020
2025
2030

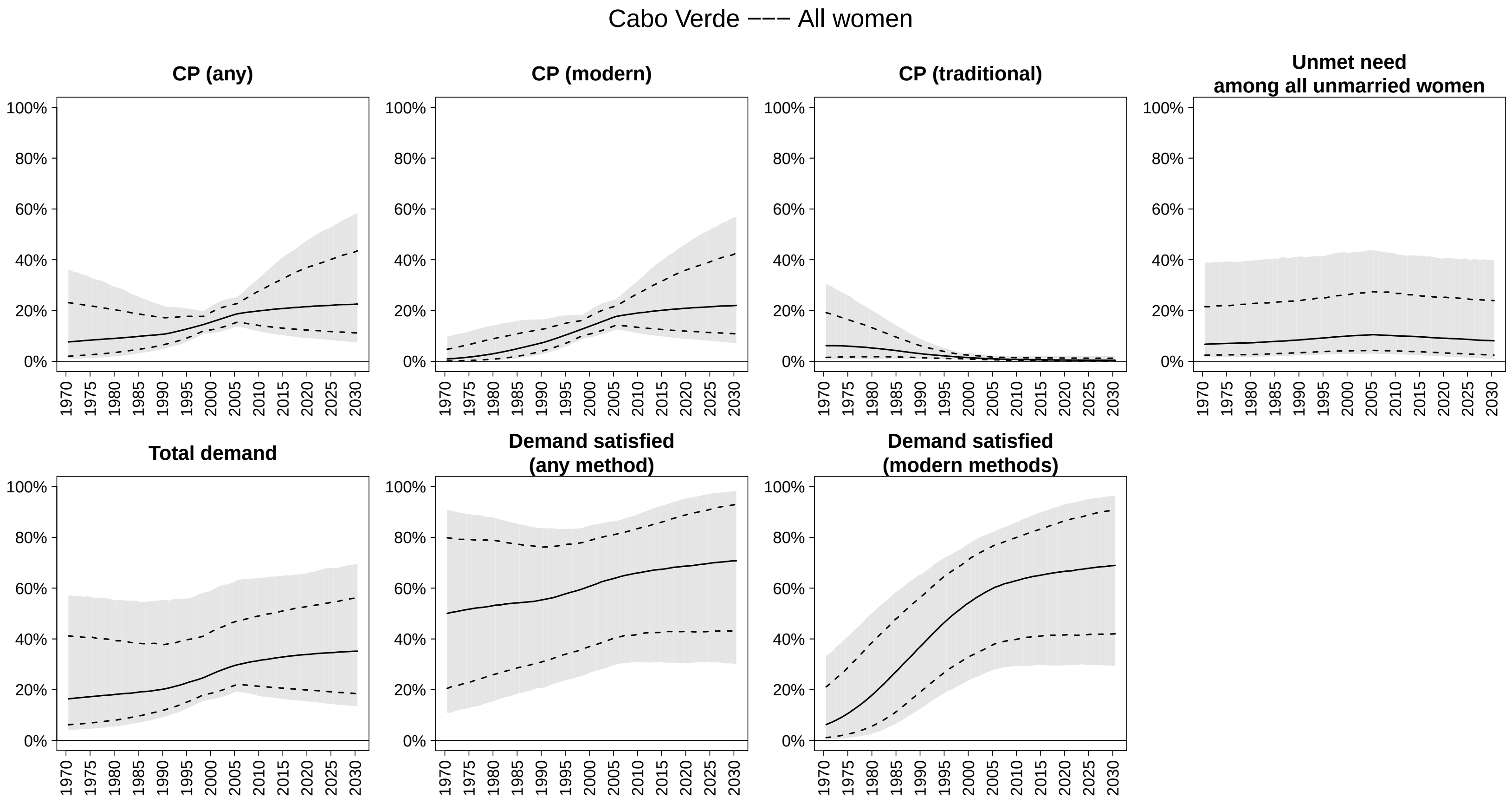
Cabo Verde −−− All women
CP (any)
CP (modern)
CP (traditional)
Unmet need
among all unmarried women
Total demand
Demand satisfied
(any method)
Demand satisfied
(modern methods)
0%
20%
40%
60%
80%
100%
1970
1975
1980
1985
1990
1995
2000
2005
2010
2015
2020
2025
2030

Cambodia −−− All women

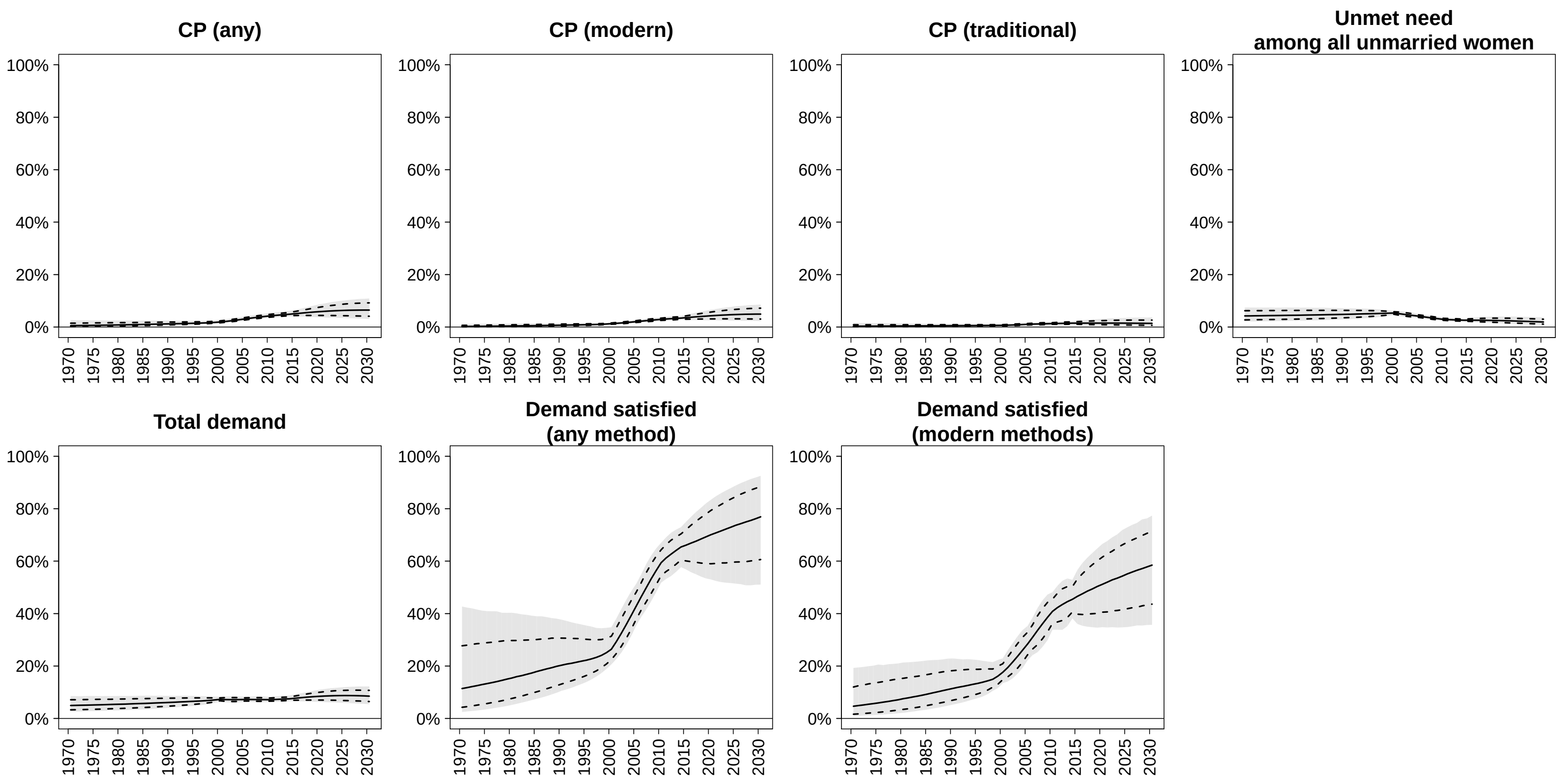

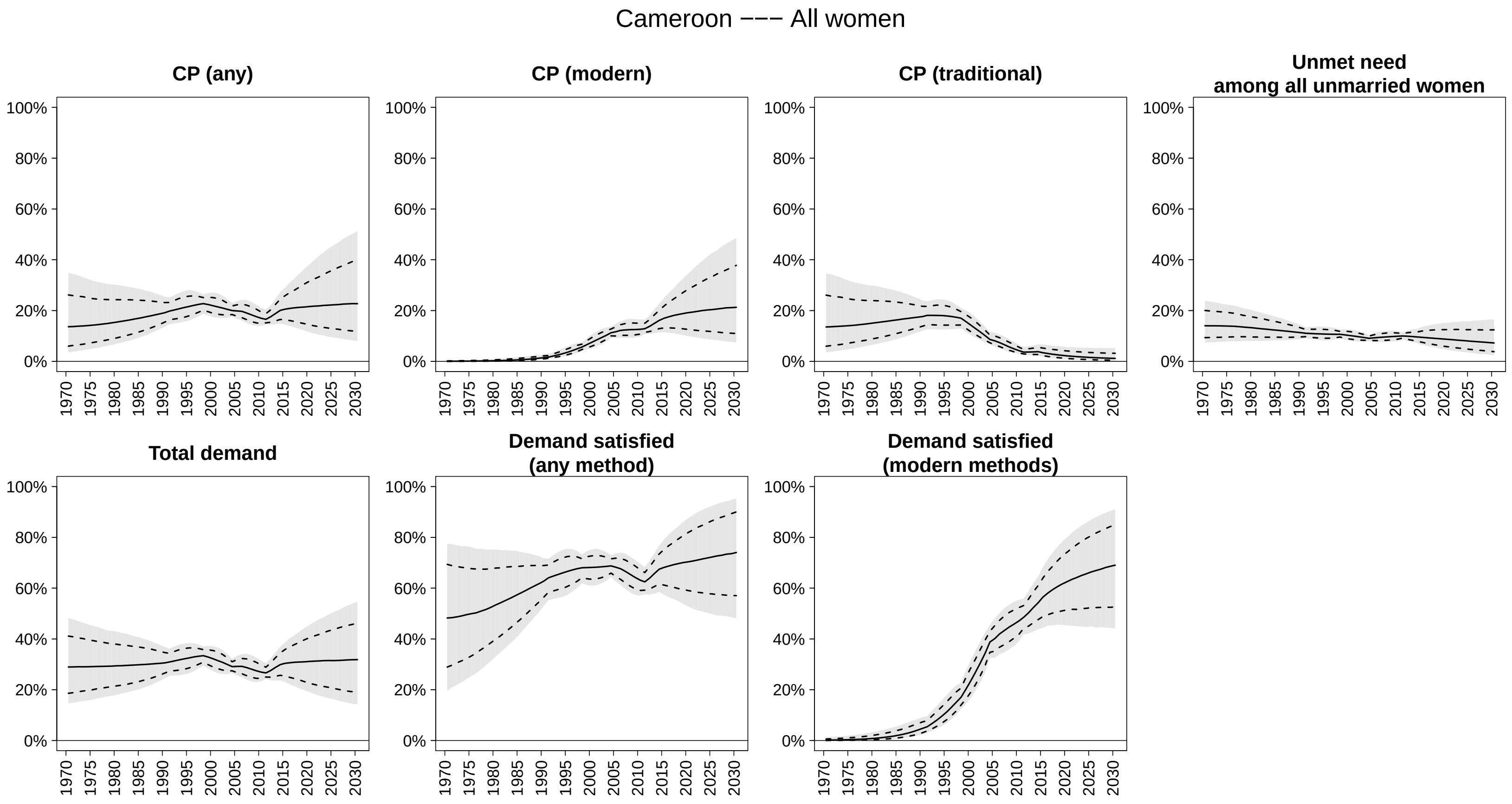
Cameroon −−− All women
CP (any)
CP (modern)
CP (traditional)
Unmet need
among all unmarried women
Total demand
Demand satisfied
(any method)
Demand satisfied
(modern methods)
0%
20%
40%
60%
80%
100%
1970
1975
1980
1985
1990
1995
2000
2005
2010
2015
2020
2025
2030

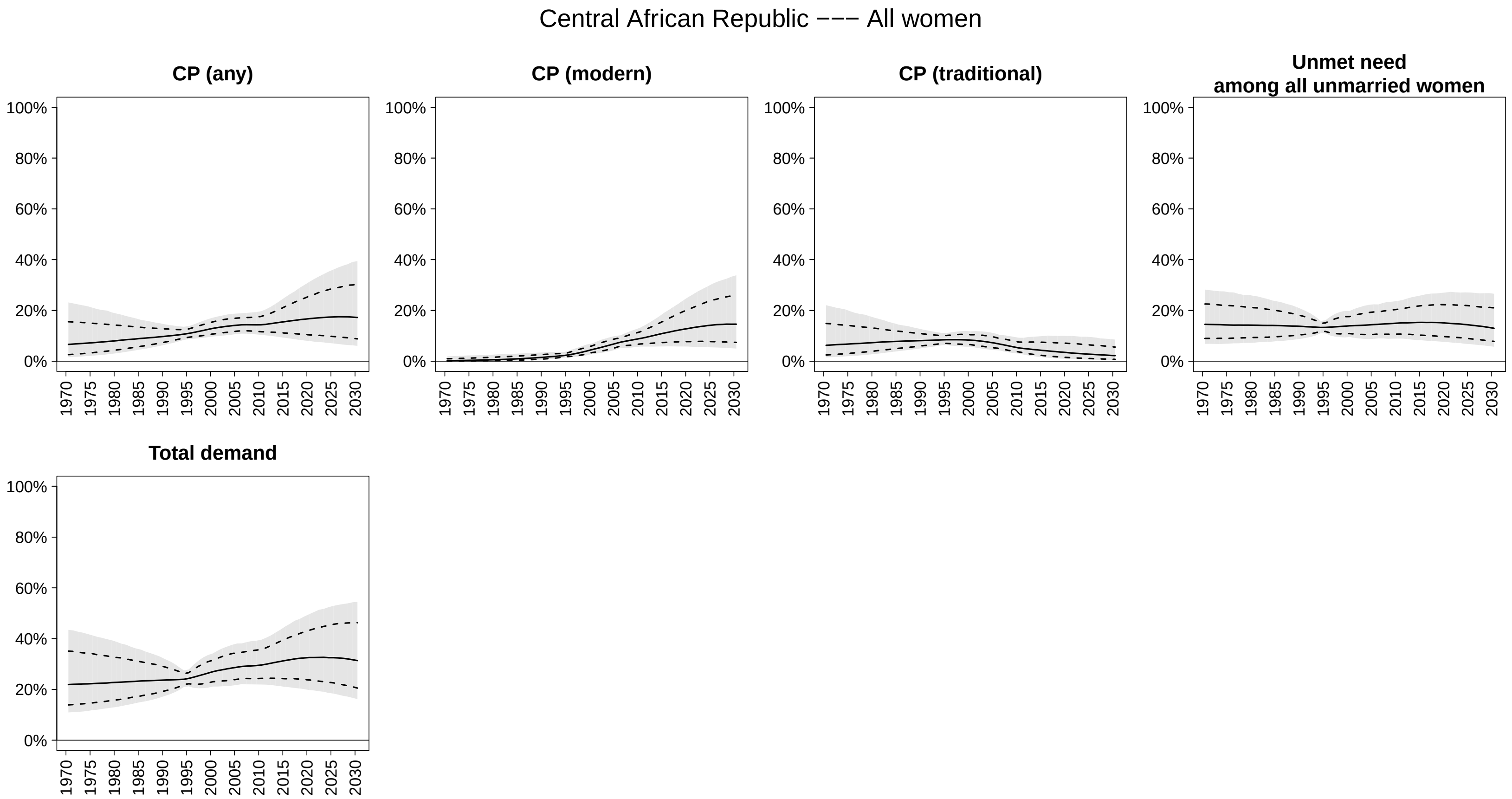
Central African Republic −−− All women
CP (any)
CP (modern)
CP (traditional)
Unmet need
among all unmarried women
Total demand
0%
20%
40%
60%
80%
100%
1970
1975
1980
1985
1990
1995
2000
2005
2010
2015
2020
2025
2030

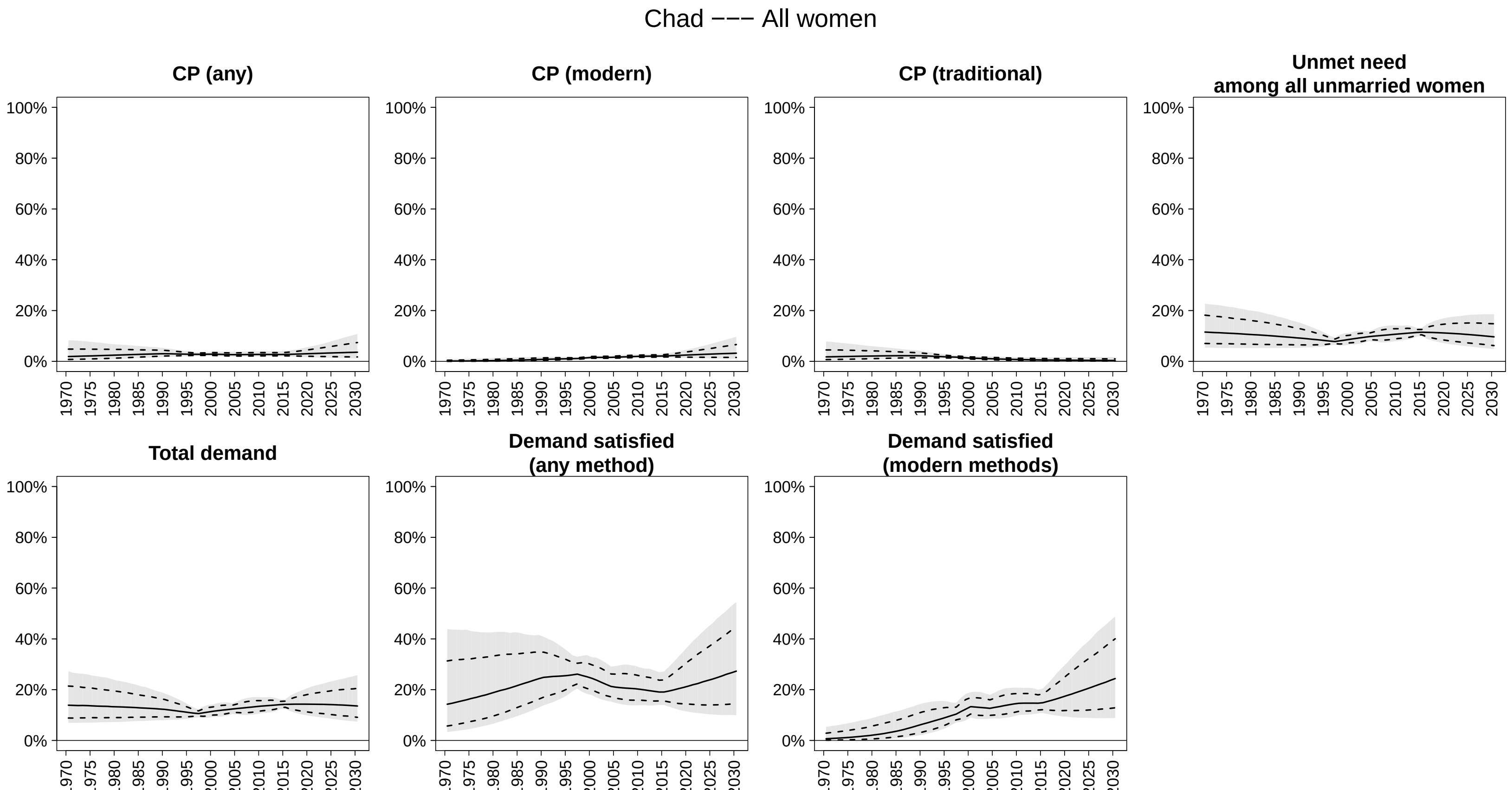
Chad −−− All women
CP (any)
CP (modern)
CP (traditional)
Unmet need among all unmarried women
Total demand
Demand satisfied (any method)
Demand satisfied (modern methods)
0%
20%
40%
60%
80%
100%
1970
1975
1980
1985
1990
1995
2000
2005
2010
2015
2020
2025
2030

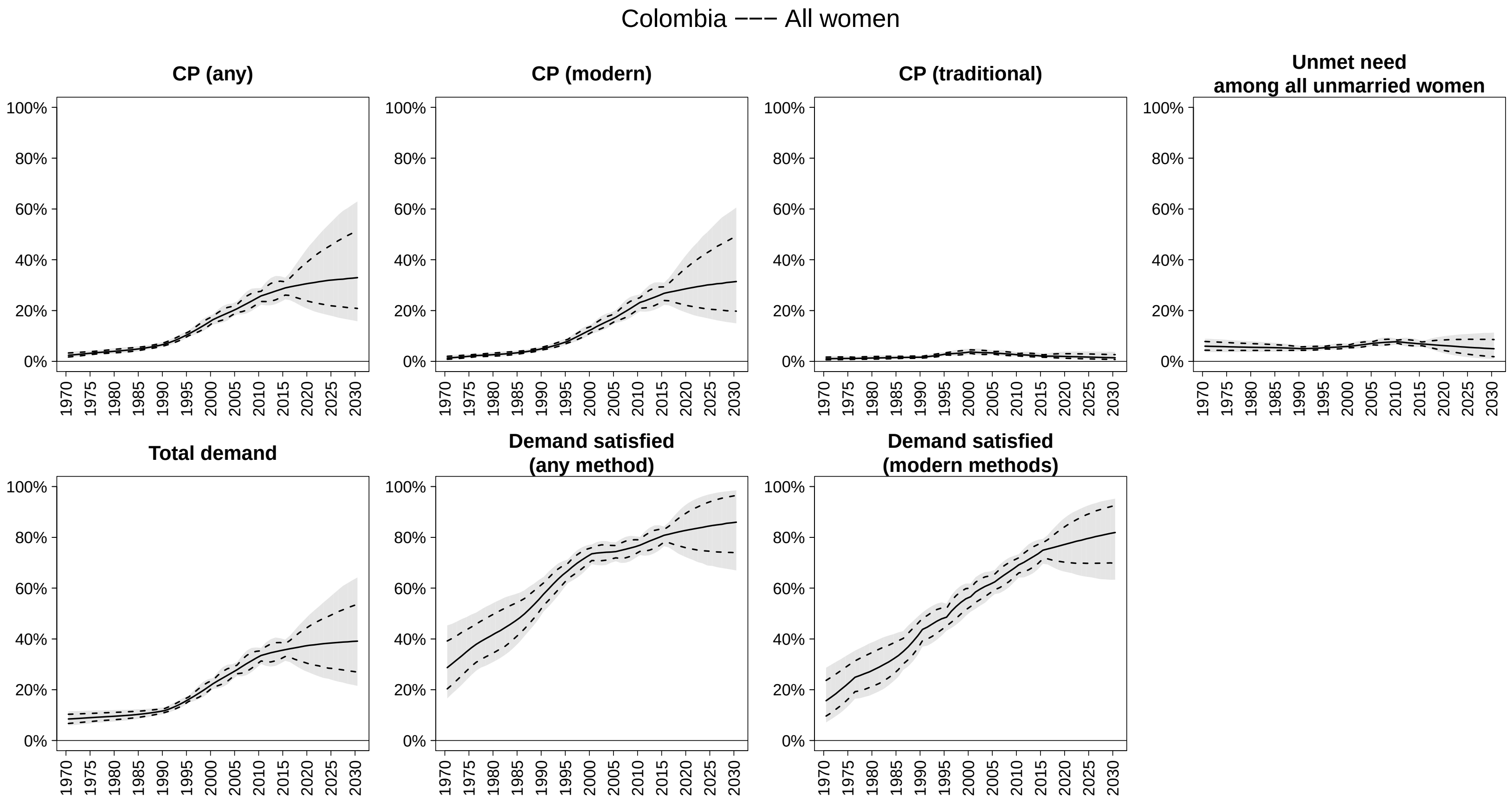
Colombia −−− All women
CP (any)
CP (modern)
CP (traditional)
Unmet need
among all unmarried women
Total demand
Demand satisfied
(any method)
Demand satisfied
(modern methods)
100%
80%
60%
40%
20%
0%
1970
1975
1980
1985
1990
1995
2000
2005
2010
2015
2020
2025
2030

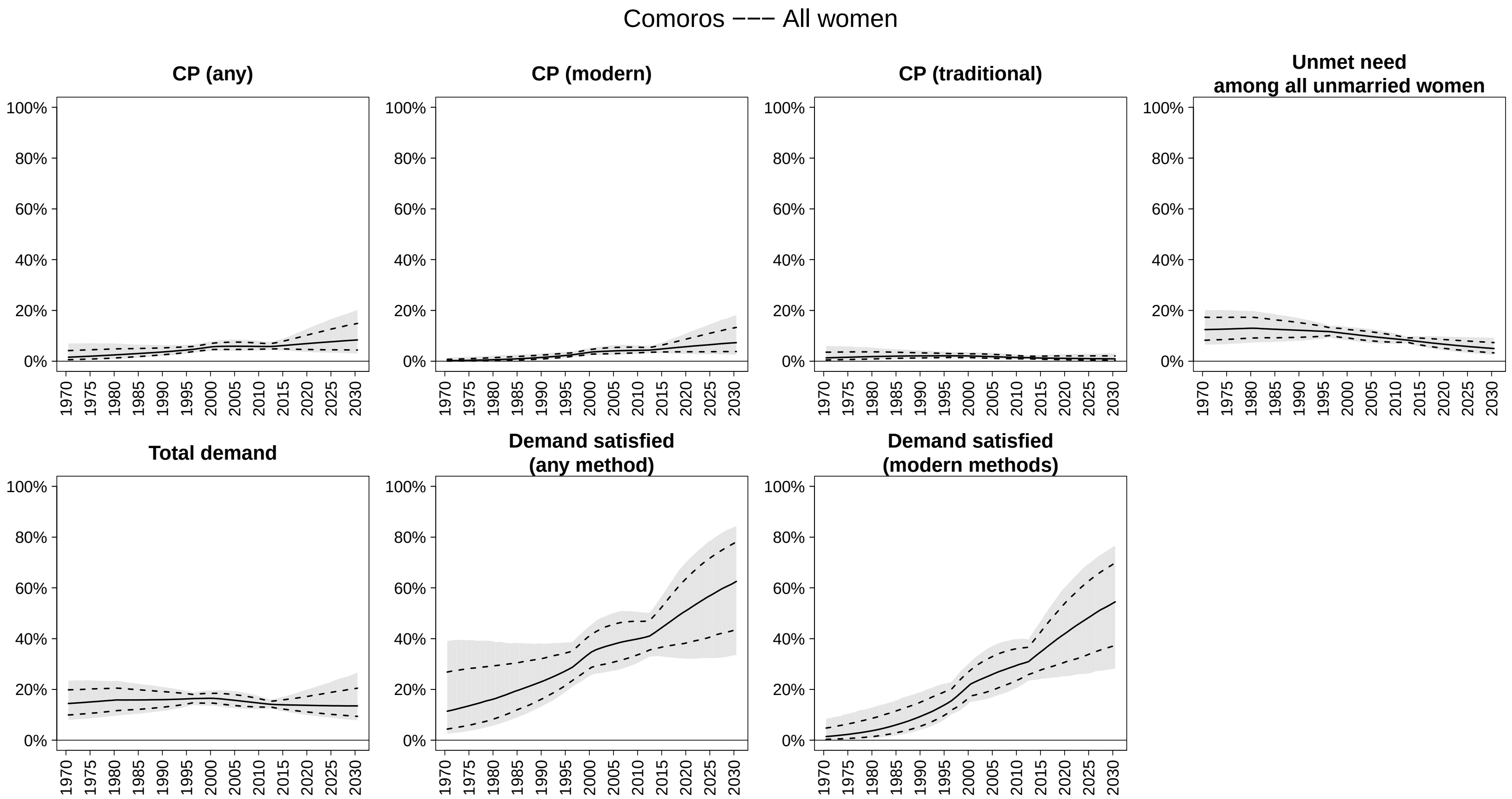
Comoros −−− All women
CP (any)
CP (modern)
CP (traditional)
Unmet need
among all unmarried women
Total demand
Demand satisfied
(any method)
Demand satisfied
(modern methods)
0%
20%
40%
60%
80%
100%
1970
1975
1980
1985
1990
1995
2000
2005
2010
2015
2020
2025
2030

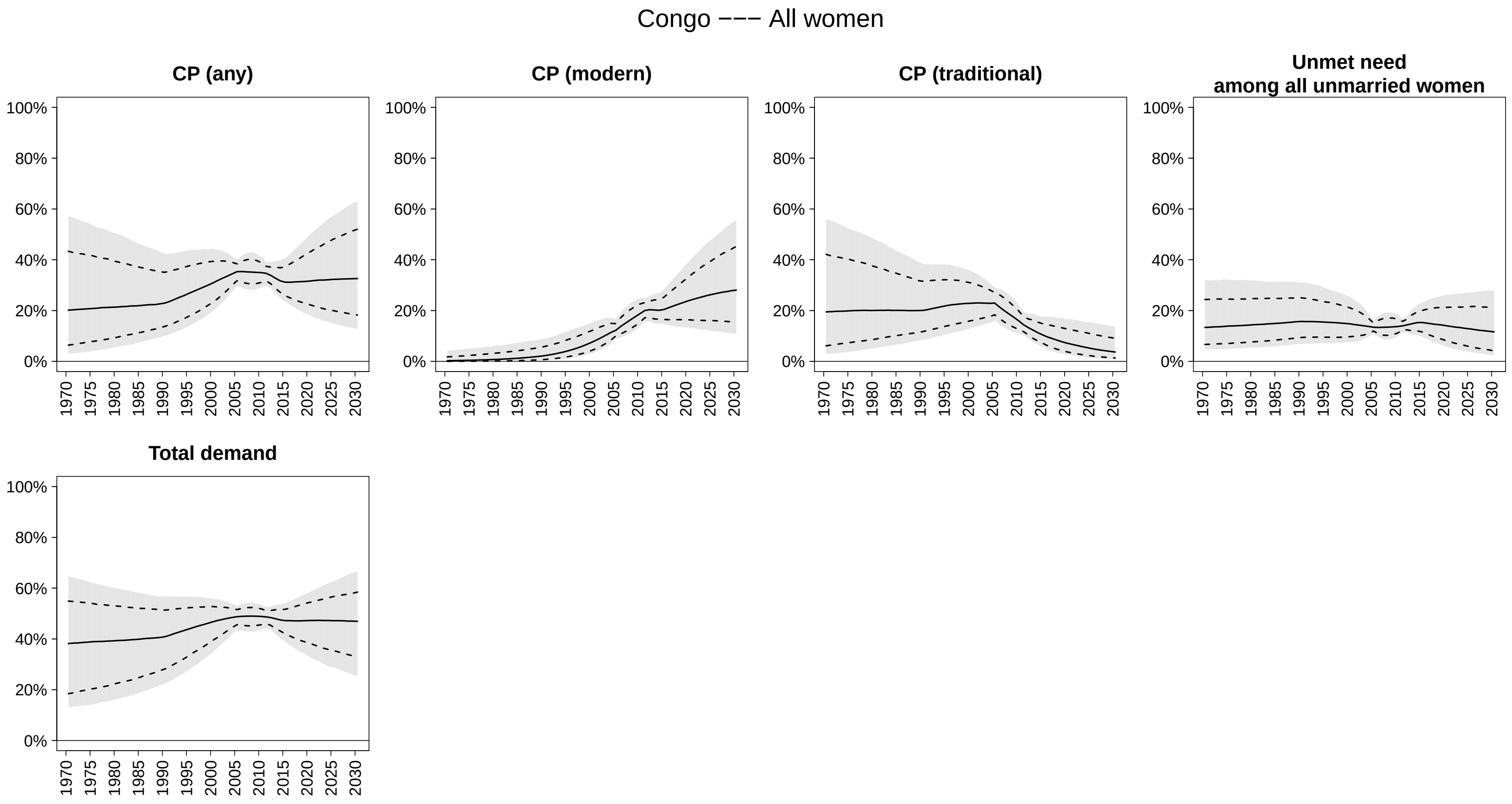
Congo --- All women
CP (any)
CP (modern)
CP (traditional)
Unmet need
among all unmarried women
Total demand
0%
20%
40%
60%
80%
100%
1970
1975
1980
1985
1990
1995
2000
2005
2010
2015
2020
2025
2030

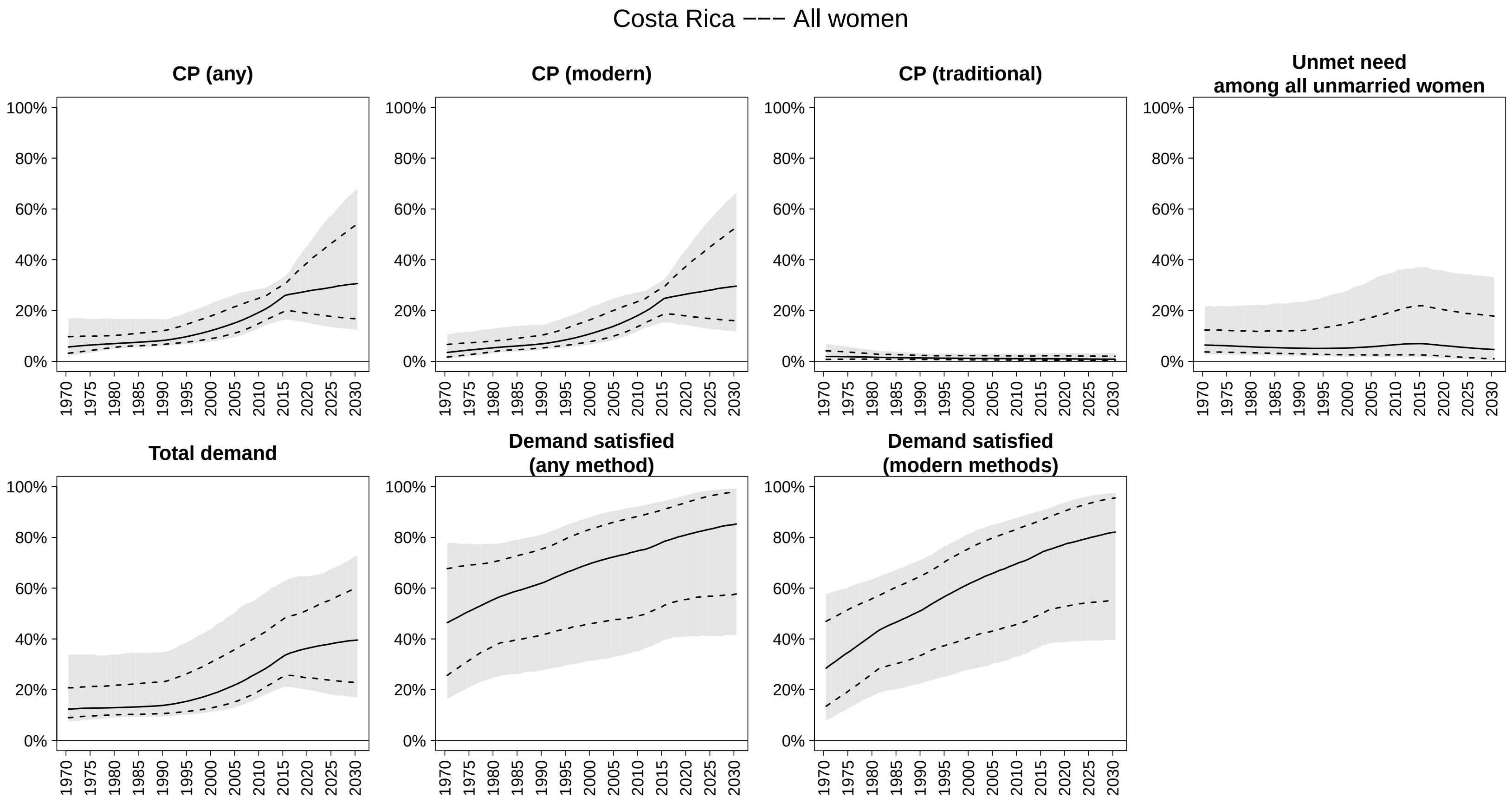
Costa Rica --- All women
CP (any)
CP (modern)
CP (traditional)
Unmet need
among all unmarried women
Total demand
Demand satisfied
(any method)
Demand satisfied
(modern methods)
100%
80%
60%
40%
20%
0%
1970
1975
1980
1985
1990
1995
2000
2005
2010
2015
2020
2025
2030

Côte d'Ivoire −−− All women

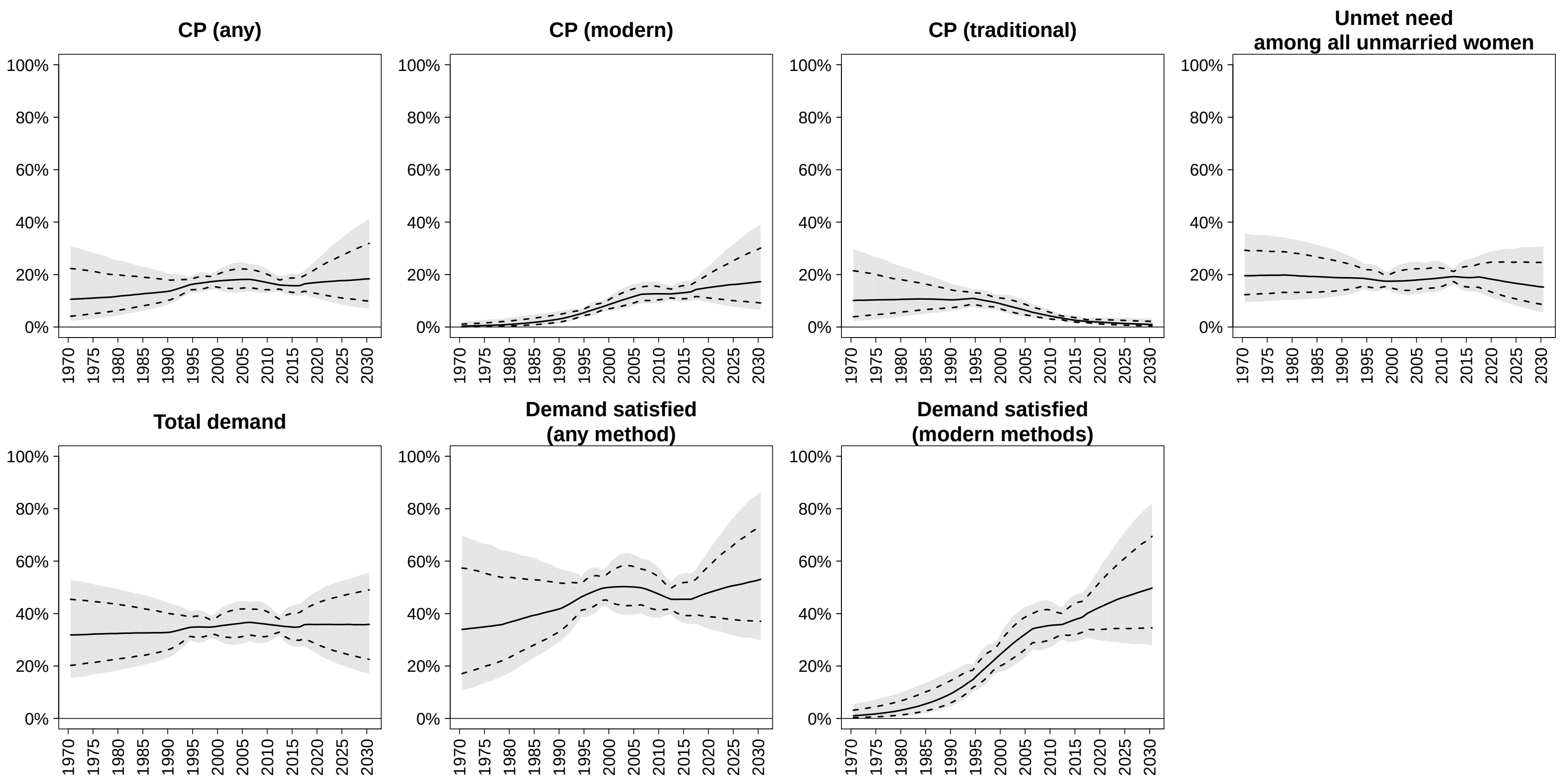

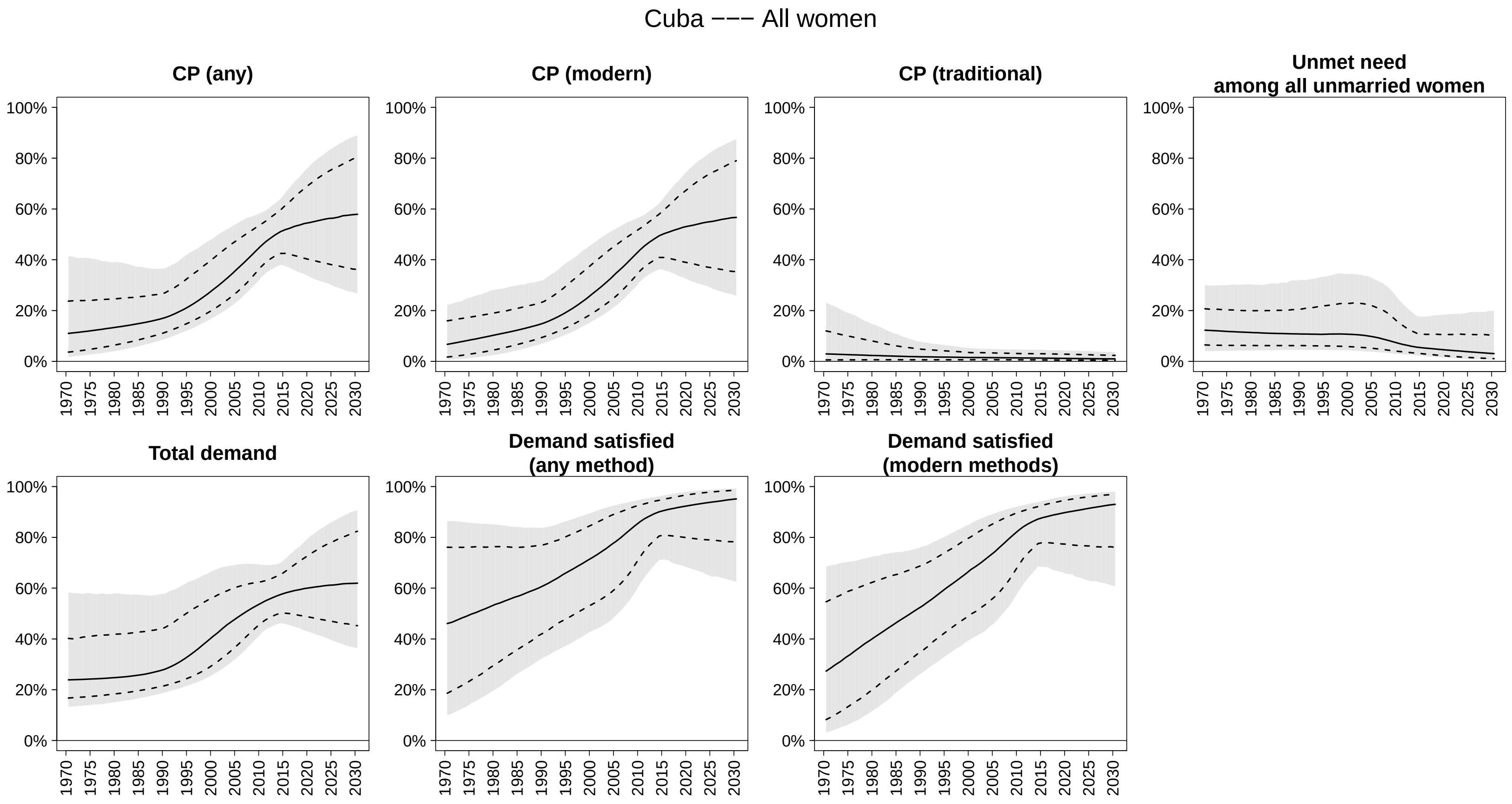

Cuba −−− All women
CP (any)
CP (modern)
CP (traditional)
Unmet need among all unmarried women
Total demand
Demand satisfied (any method)
Demand satisfied (modern methods)
0%
20%
40%
60%
80%
100%
1970
1975
1980
1985
1990
1995
2000
2005
2010
2015
2020
2025
2030

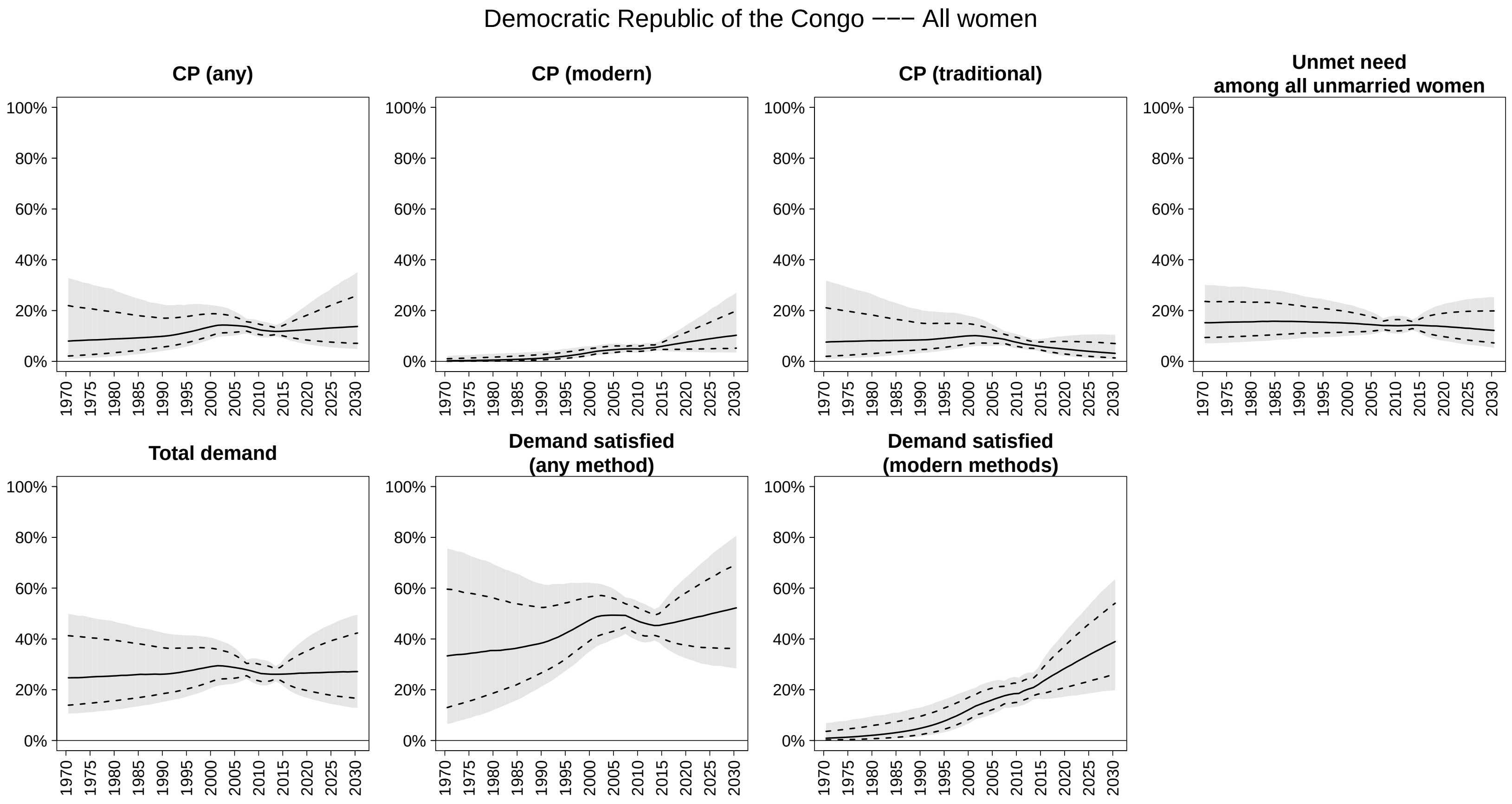
Democratic Republic of the Congo −−− All women
CP (any)
CP (modern)
CP (traditional)
Unmet need among all unmarried women
Total demand
Demand satisfied (any method)
Demand satisfied (modern methods)
0%
20%
40%
60%
80%
100%
1970
1975
1980
1985
1990
1995
2000
2005
2010
2015
2020
2025
2030

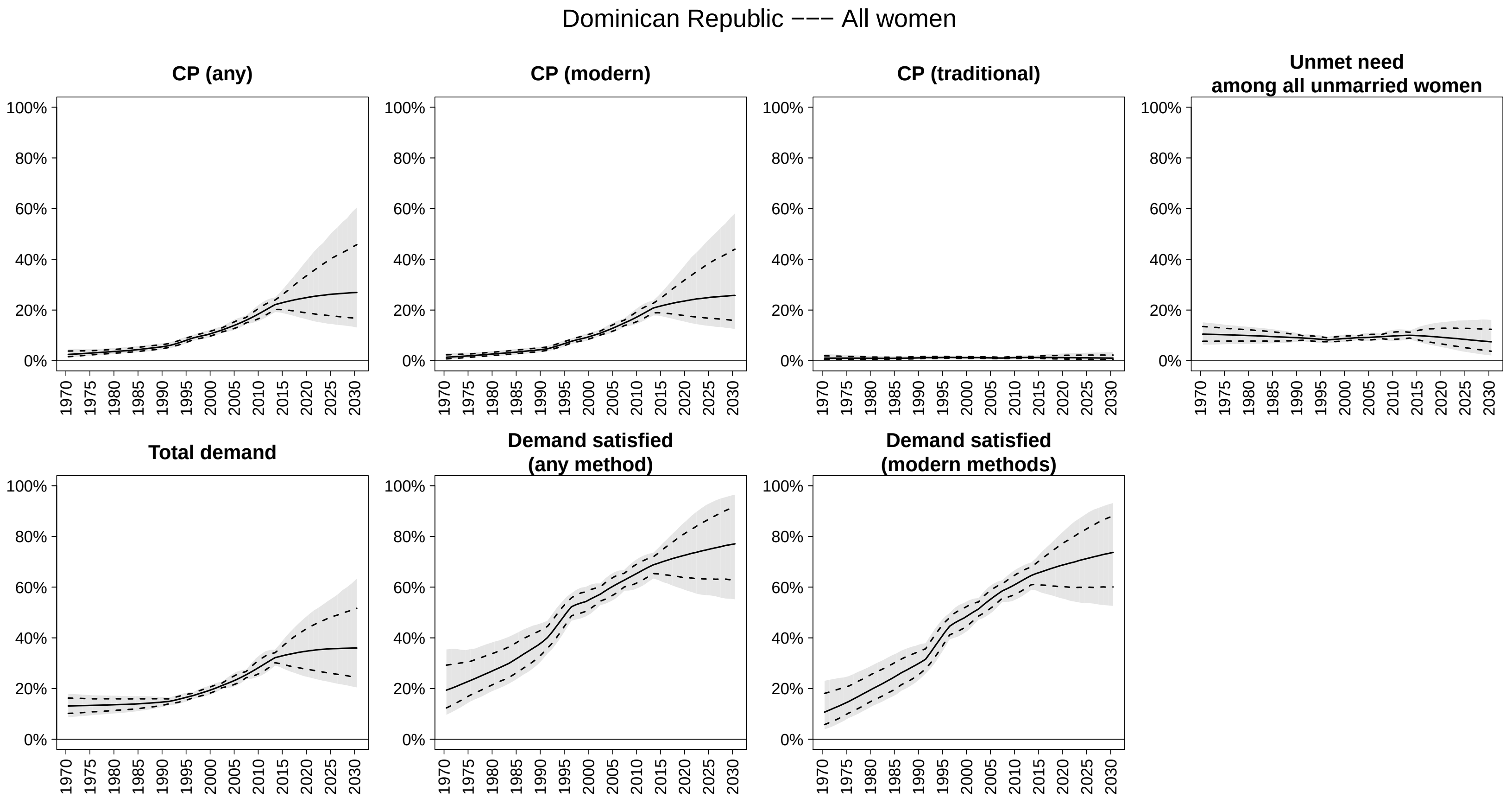
Dominican Republic −−− All women
CP (any)
CP (modern)
CP (traditional)
Unmet need among all unmarried women
Total demand
Demand satisfied (any method)
Demand satisfied (modern methods)
0%
20%
40%
60%
80%
100%
1970
1975
1980
1985
1990
1995
2000
2005
2010
2015
2020
2025
2030

Ecuador ––– All women

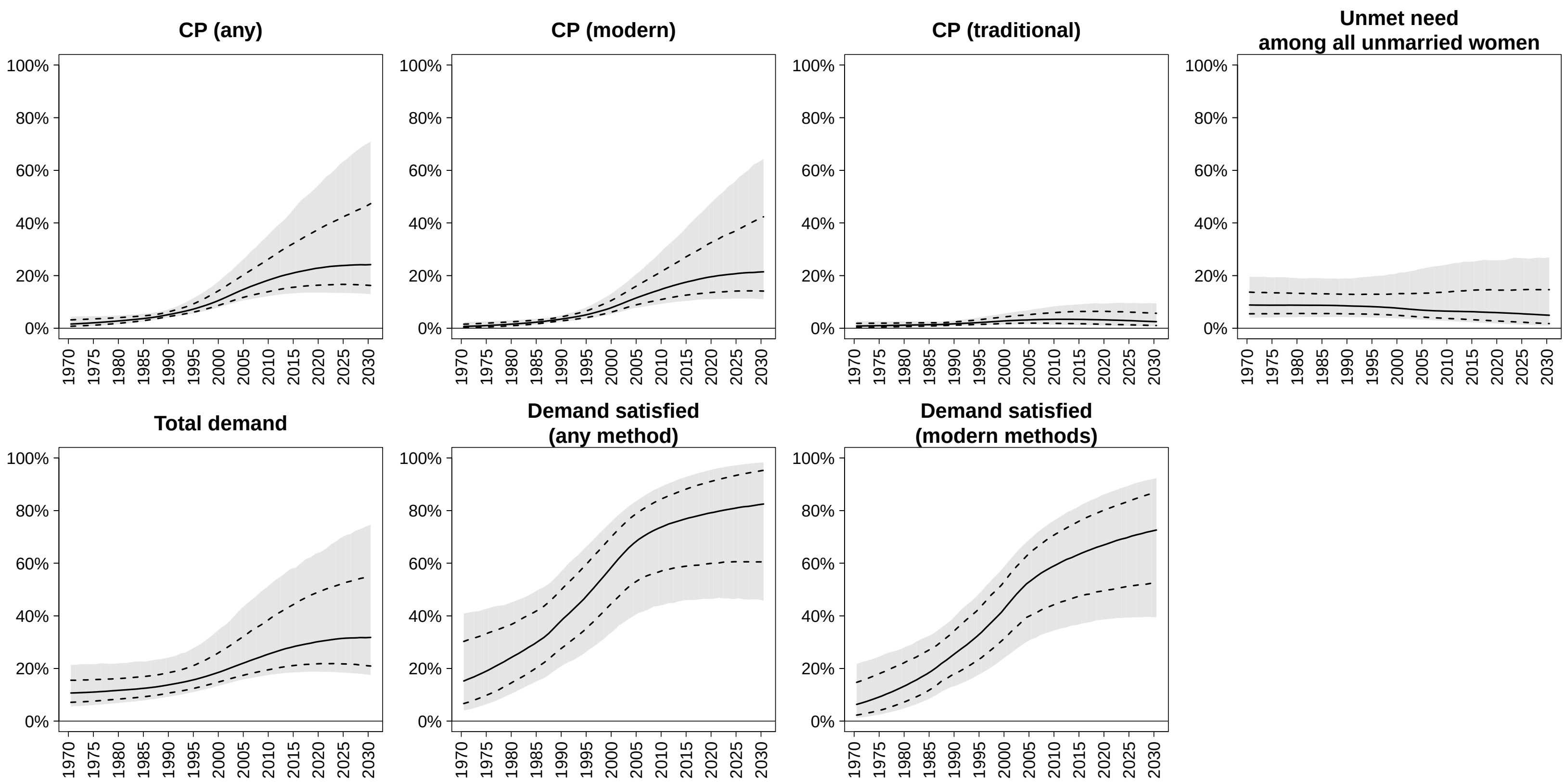

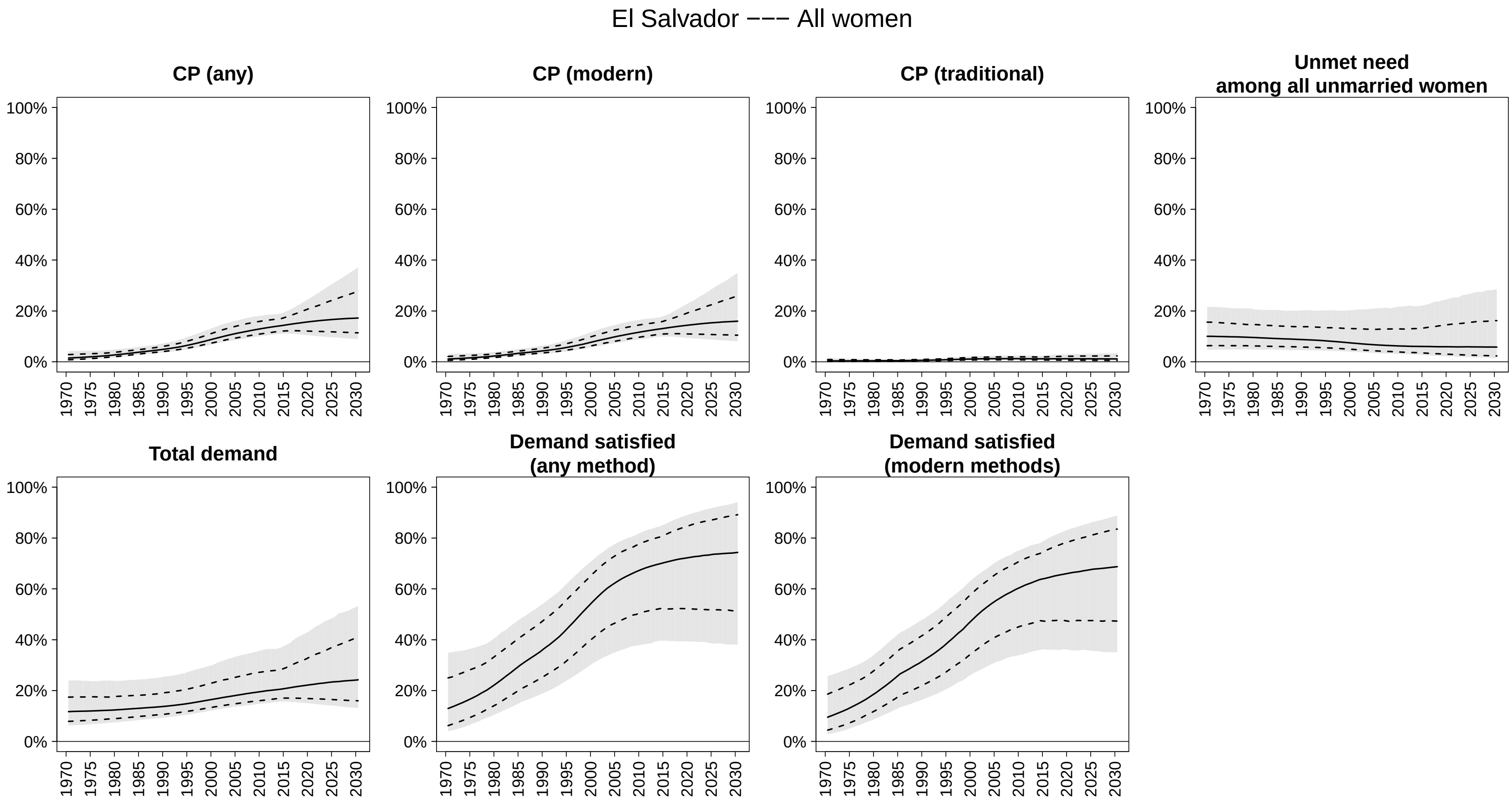
El Salvador −−− All women
CP (any)
CP (modern)
CP (traditional)
Unmet need
among all unmarried women
Total demand
Demand satisfied
(any method)
Demand satisfied
(modern methods)
0%
20%
40%
60%
80%
100%
1970
1975
1980
1985
1990
1995
2000
2005
2010
2015
2020
2025
2030

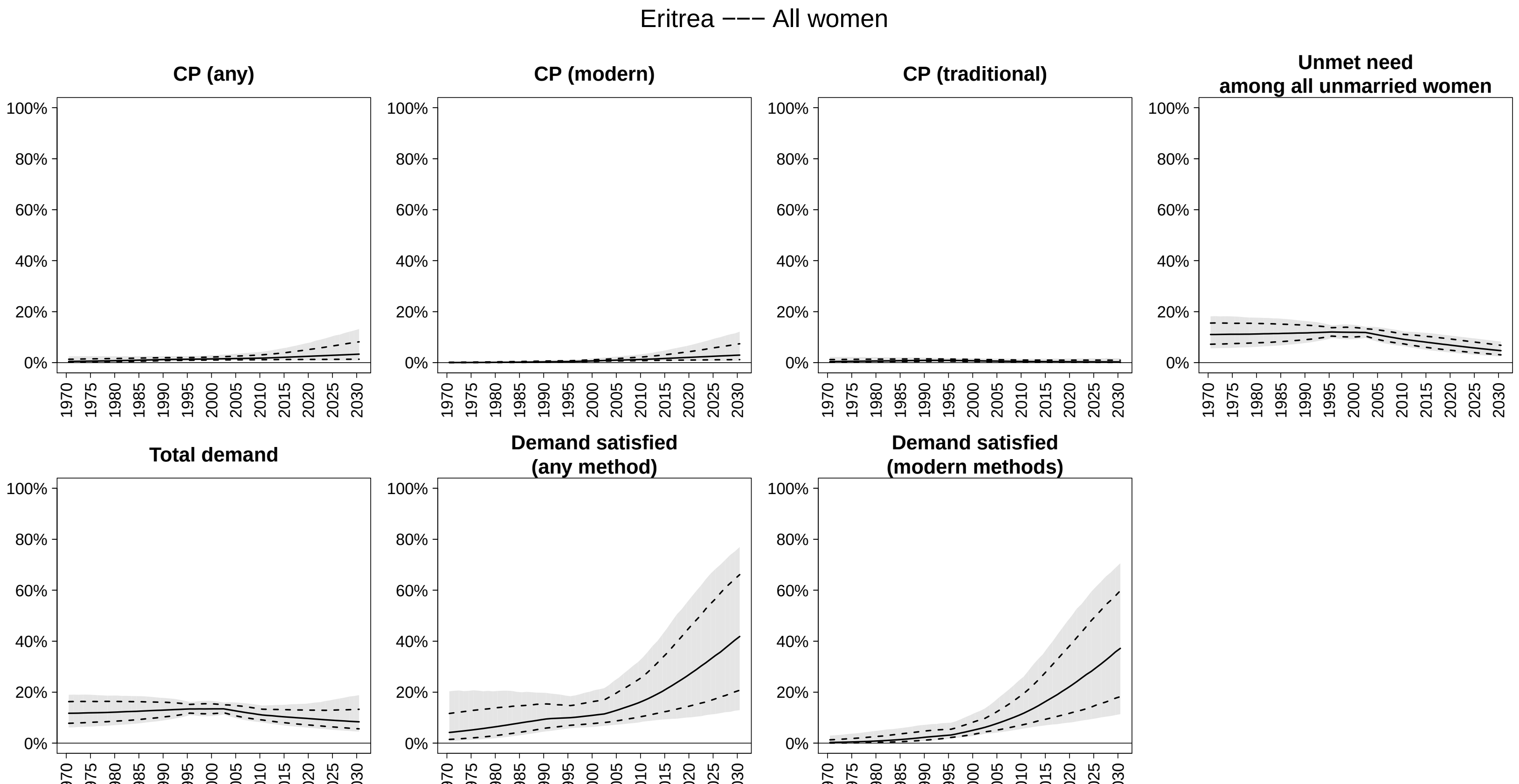
Eritrea −−− All women
CP (any)
CP (modern)
CP (traditional)
Unmet need
among all unmarried women
Total demand
Demand satisfied
(any method)
Demand satisfied
(modern methods)
100%
80%
60%
40%
20%
0%
1970
1975
1980
1985
1990
1995
2000
2005
2010
2015
2020
2025
2030

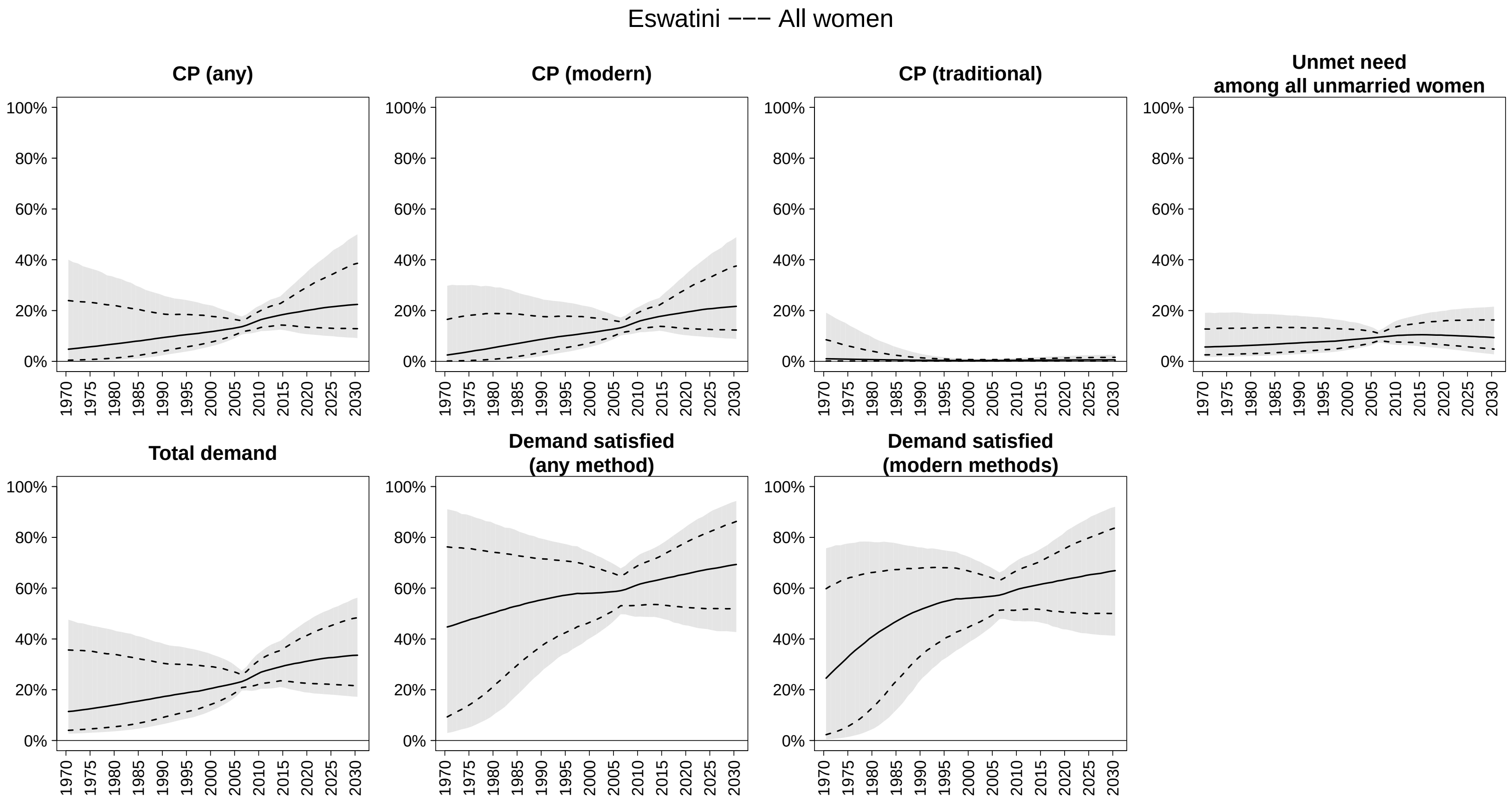
Eswatini −−− All women
CP (any)
CP (modern)
CP (traditional)
Unmet need among all unmarried women
Total demand
Demand satisfied (any method)
Demand satisfied (modern methods)
0%
20%
40%
60%
80%
100%
1970
1975
1980
1985
1990
1995
2000
2005
2010
2015
2020
2025
2030

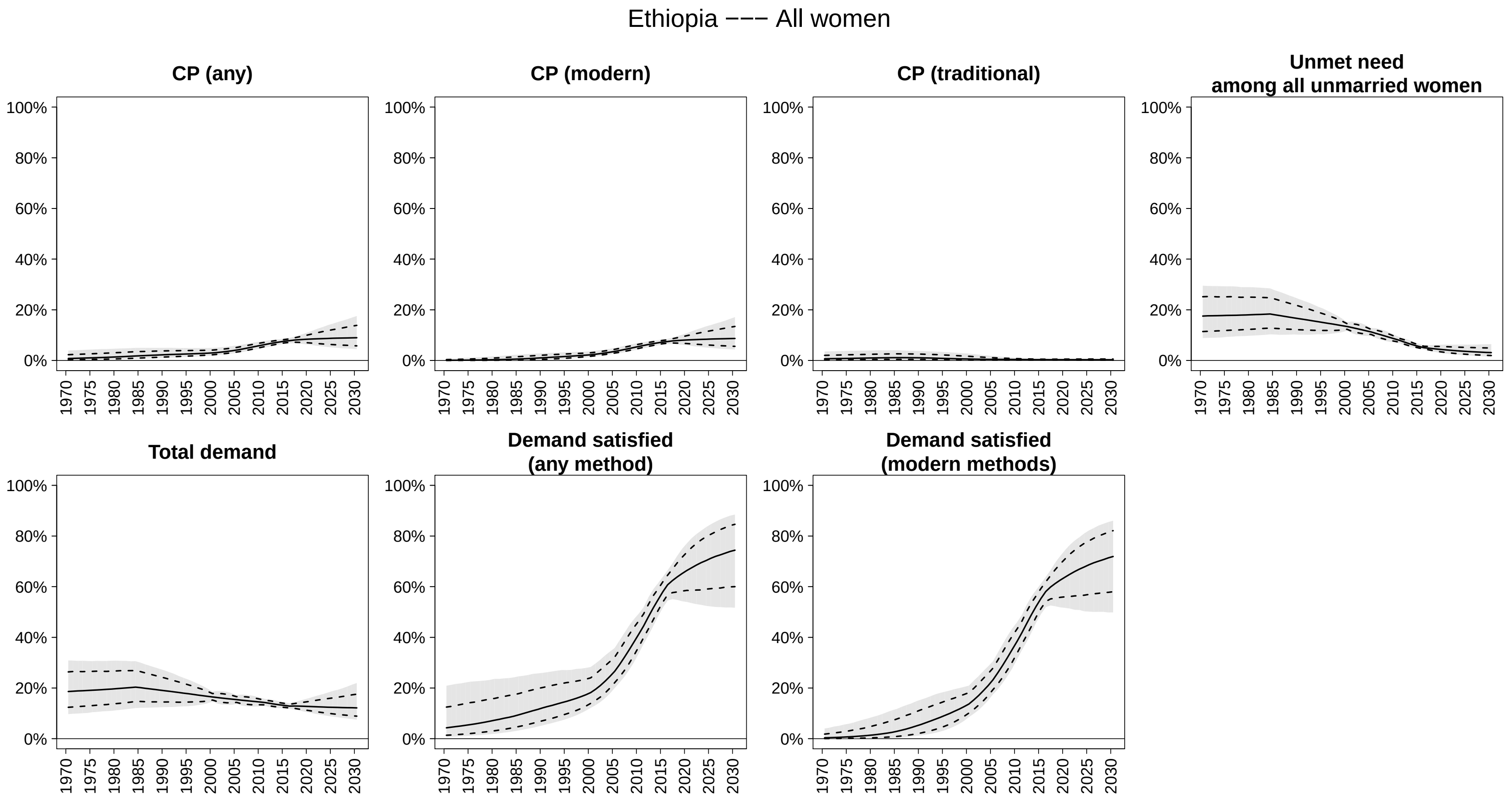
Ethiopia −−− All women
CP (any)
CP (modern)
CP (traditional)
Unmet need
among all unmarried women
Total demand
Demand satisfied
(any method)
Demand satisfied
(modern methods)
0%
20%
40%
60%
80%
100%
1970
1975
1980
1985
1990
1995
2000
2005
2010
2015
2020
2025
2030

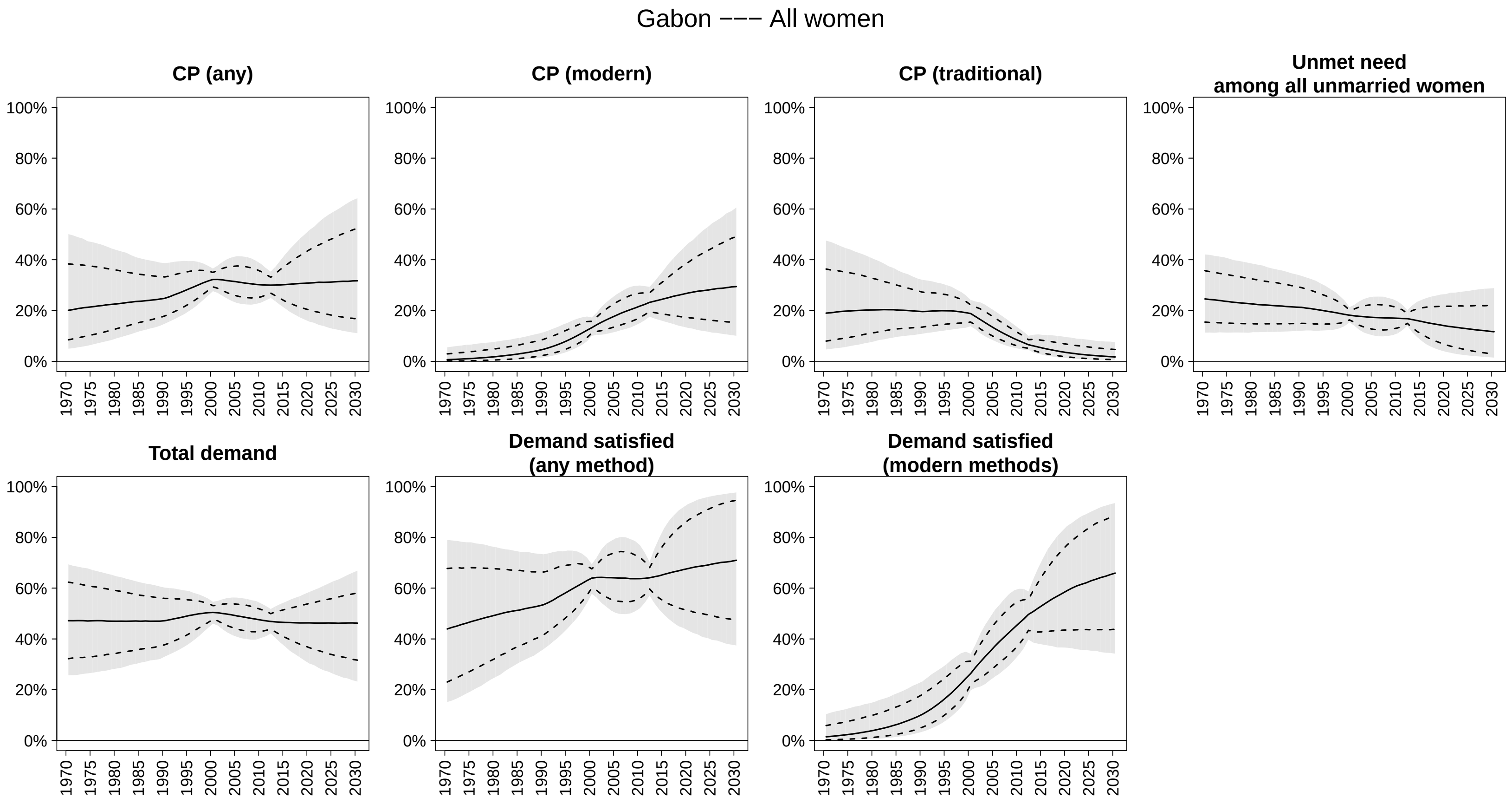
Gabon −−− All women
CP (any)
CP (modern)
CP (traditional)
Unmet need among all unmarried women
Total demand
Demand satisfied (any method)
Demand satisfied (modern methods)
0%
20%
40%
60%
80%
100%
1970
1975
1980
1985
1990
1995
2000
2005
2010
2015
2020
2025
2030

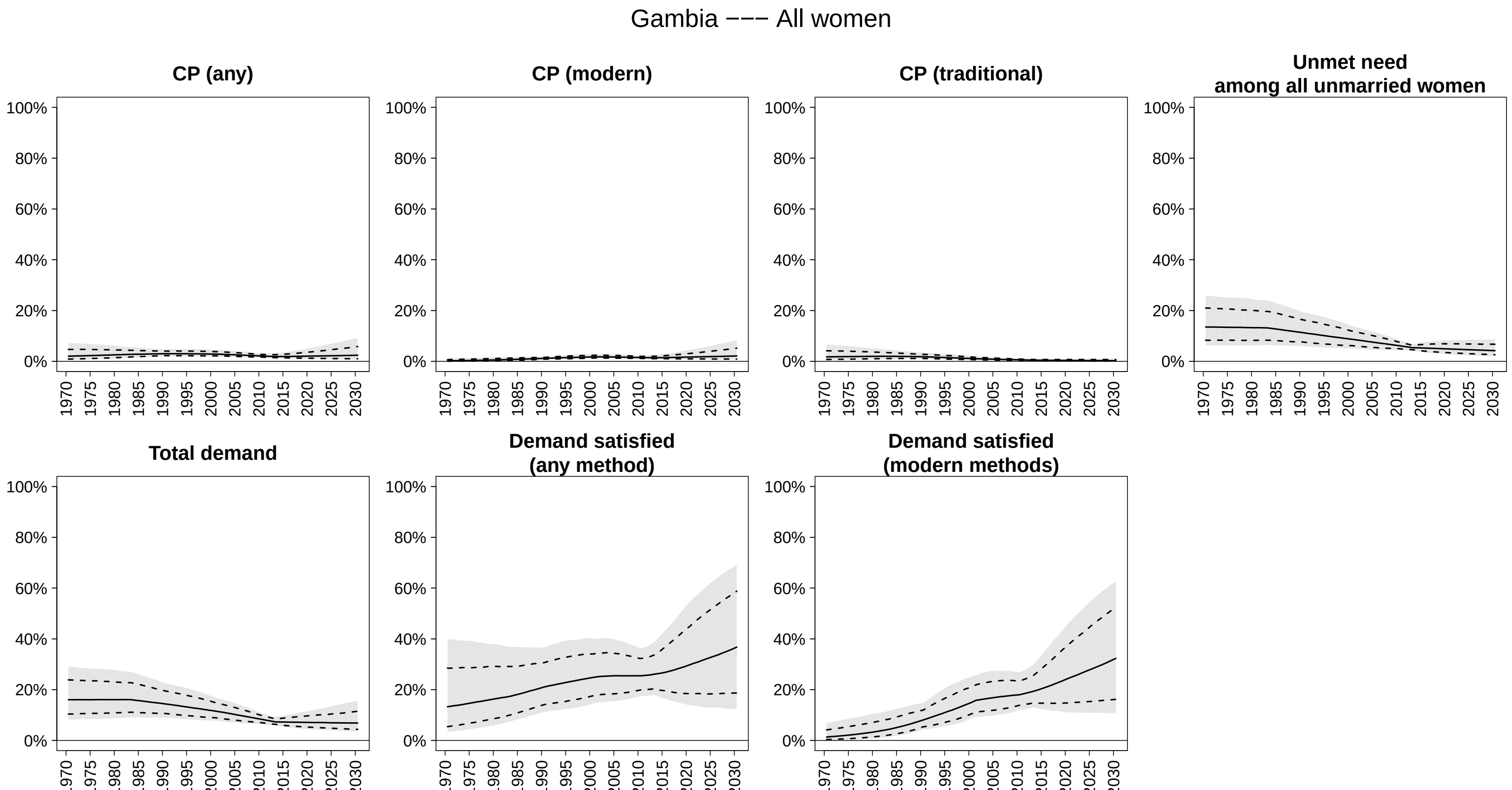
Gambia −−− All women
CP (any)
CP (modern)
CP (traditional)
Unmet need among all unmarried women
Total demand
Demand satisfied (any method)
Demand satisfied (modern methods)
0%
20%
40%
60%
80%
100%
1970
1975
1980
1985
1990
1995
2000
2005
2010
2015
2020
2025
2030

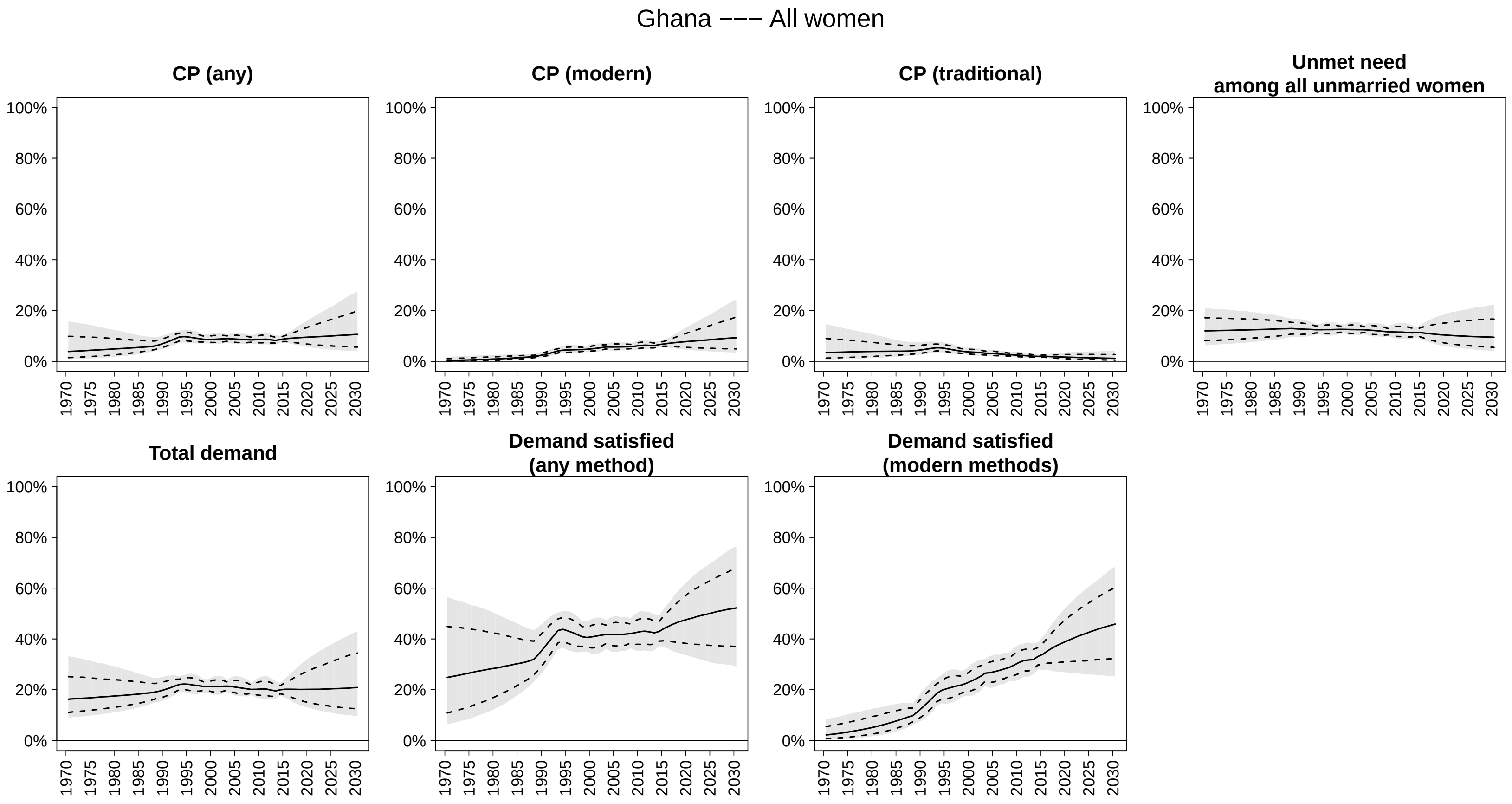
Ghana −−− All women
CP (any)
CP (modern)
CP (traditional)
Unmet need
among all unmarried women
Total demand
Demand satisfied
(any method)
Demand satisfied
(modern methods)
100%
80%
60%
40%
20%
0%
1970
1975
1980
1985
1990
1995
2000
2005
2010
2015
2020
2025
2030

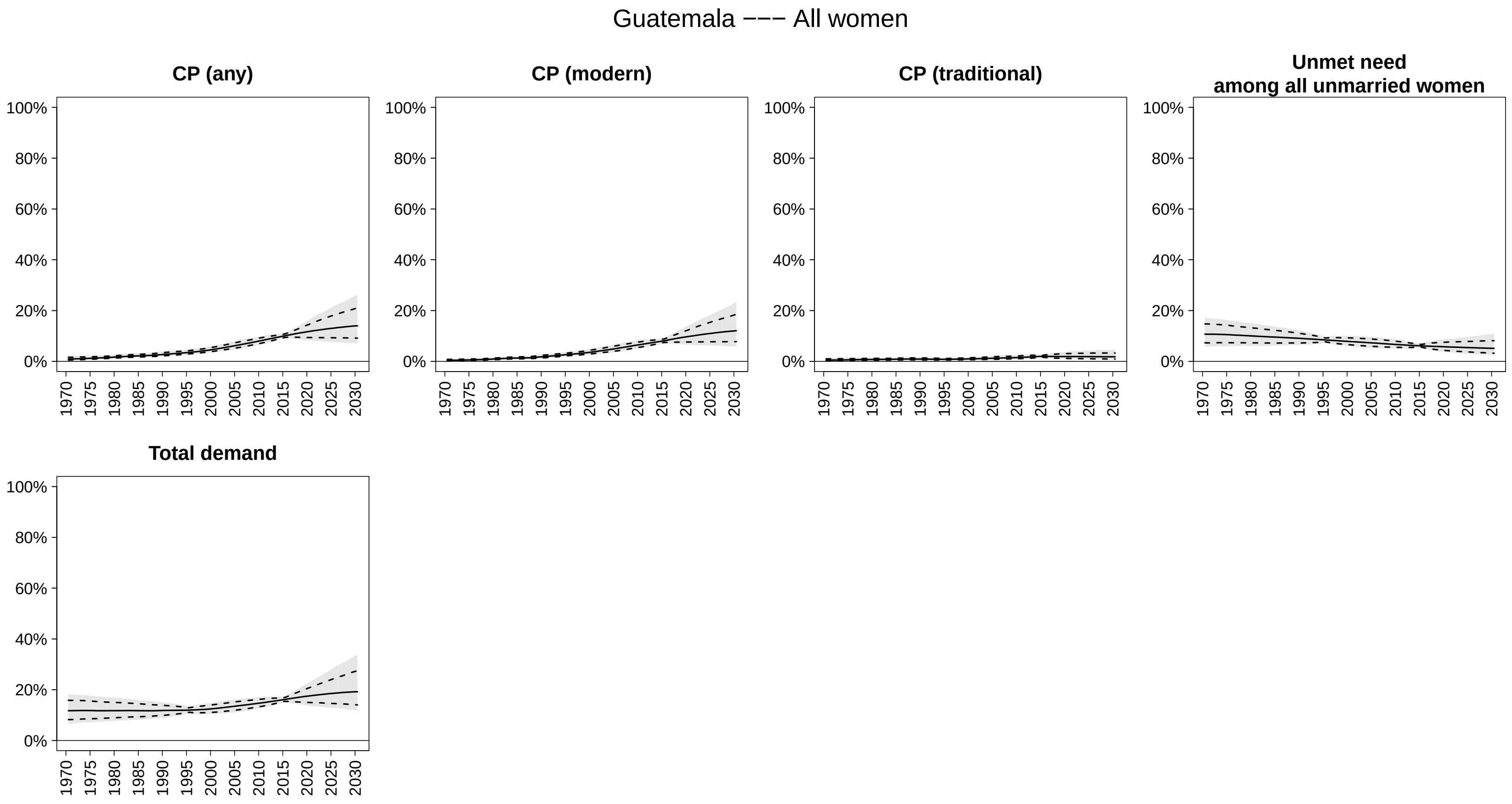
Guatemala −−− All women
CP (any)
CP (modern)
CP (traditional)
Unmet need
among all unmarried women
Total demand
100%
80%
60%
40%
20%
0%
1970
1975
1980
1985
1990
1995
2000
2005
2010
2015
2020
2025
2030

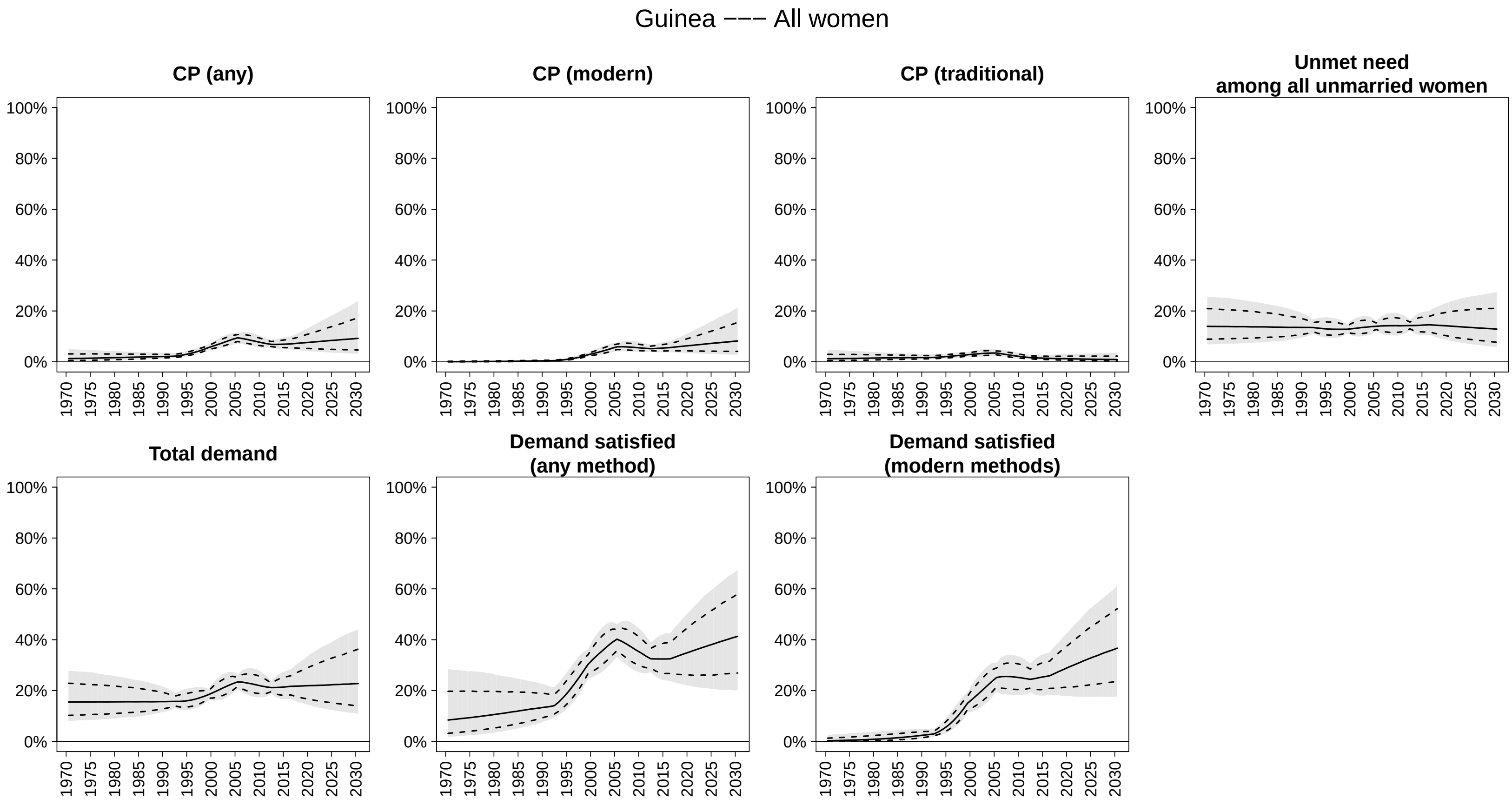
Guinea −−− All women
CP (any)
CP (modern)
CP (traditional)
Unmet need among all unmarried women
Total demand
Demand satisfied (any method)
Demand satisfied (modern methods)
0%
20%
40%
60%
80%
100%
1970
1975
1980
1985
1990
1995
2000
2005
2010
2015
2020
2025
2030

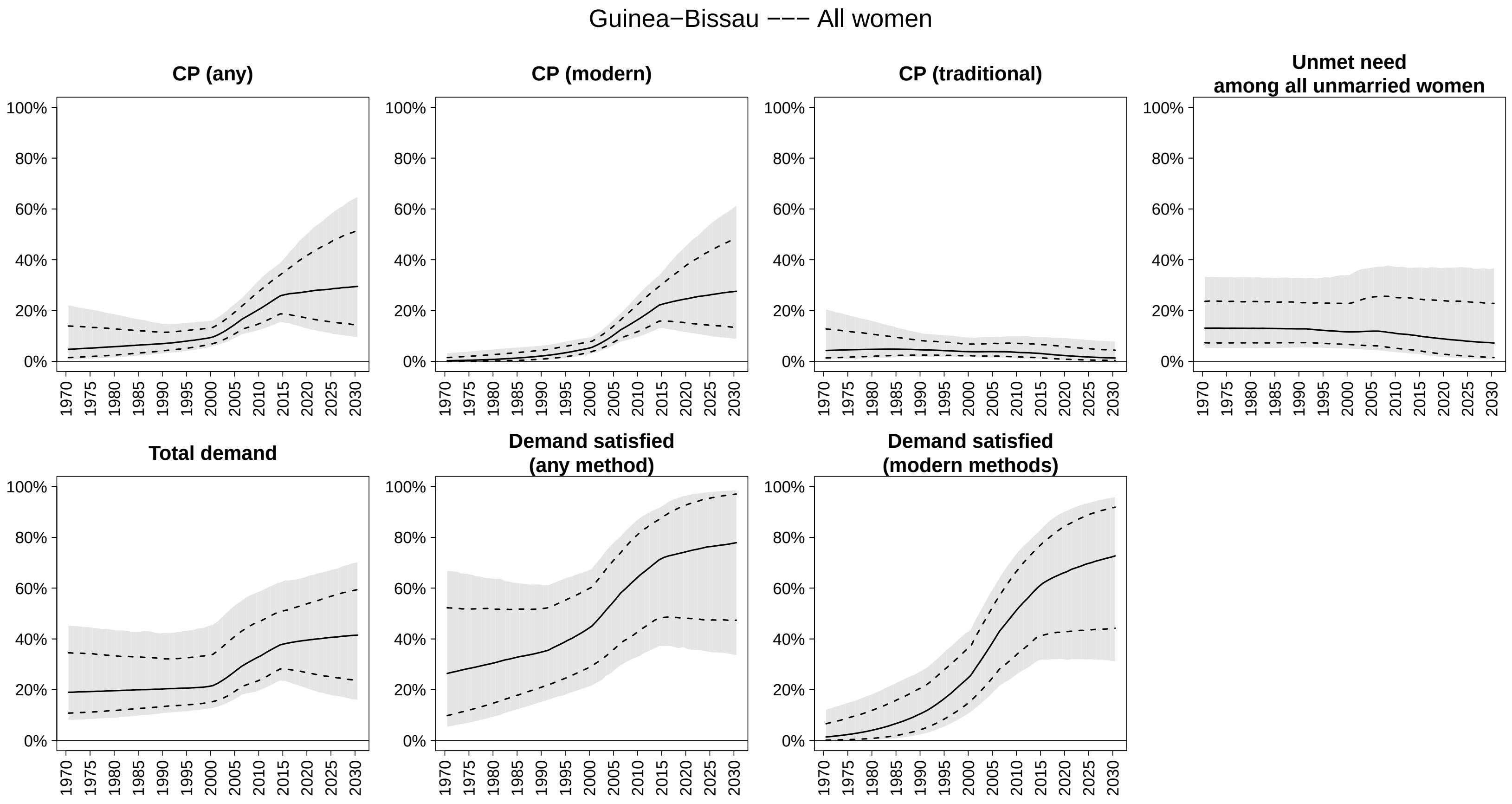
Guinea−Bissau −−− All women
CP (any)
CP (modern)
CP (traditional)
Unmet need among all unmarried women
Total demand
Demand satisfied (any method)
Demand satisfied (modern methods)
0%
20%
40%
60%
80%
100%
1970
1975
1980
1985
1990
1995
2000
2005
2010
2015
2020
2025
2030

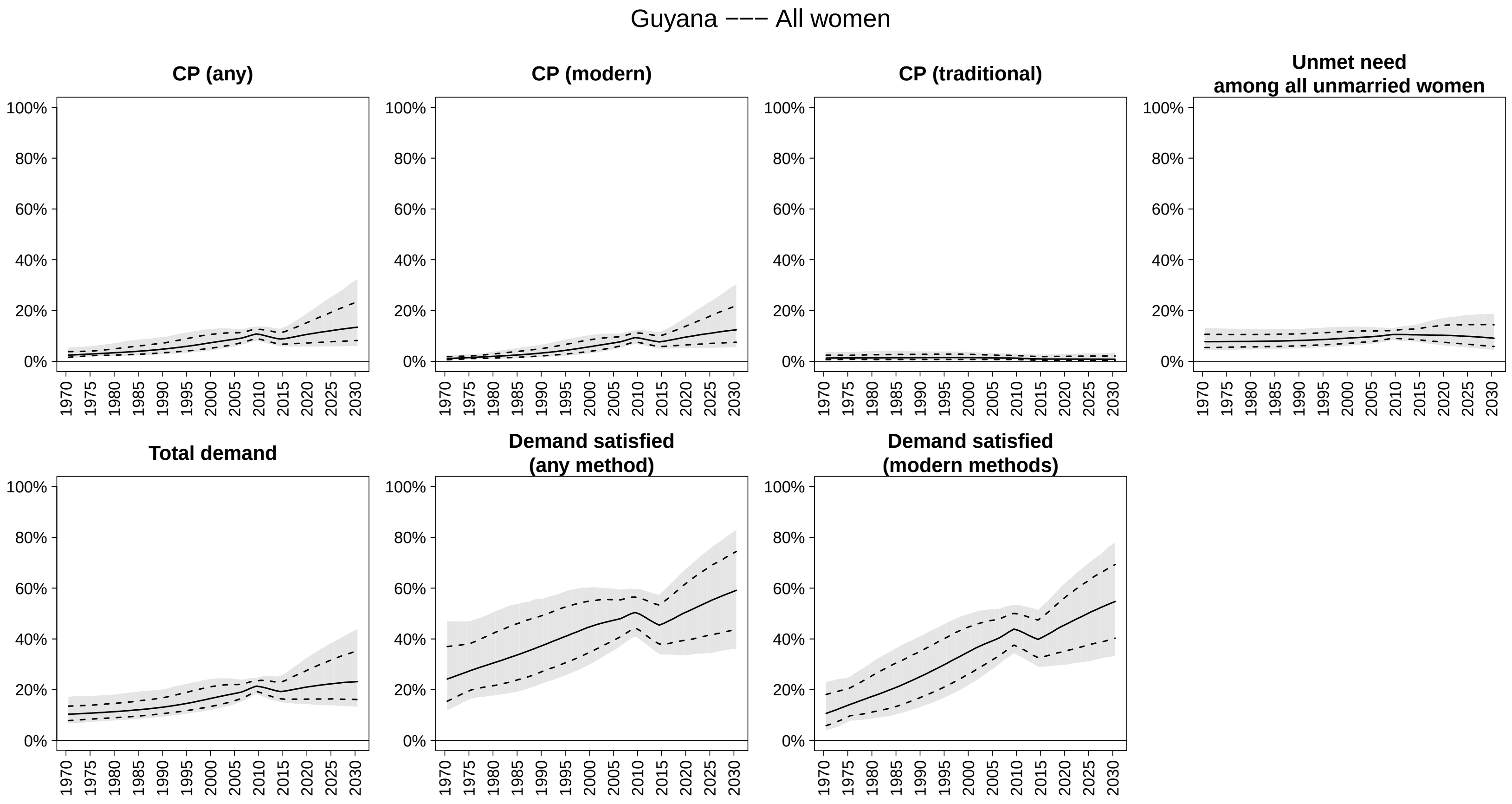
Guyana −−− All women
CP (any)
CP (modern)
CP (traditional)
Unmet need among all unmarried women
Total demand
Demand satisfied (any method)
Demand satisfied (modern methods)
0%
20%
40%
60%
80%
100%
1970
1975
1980
1985
1990
1995
2000
2005
2010
2015
2020
2025
2030

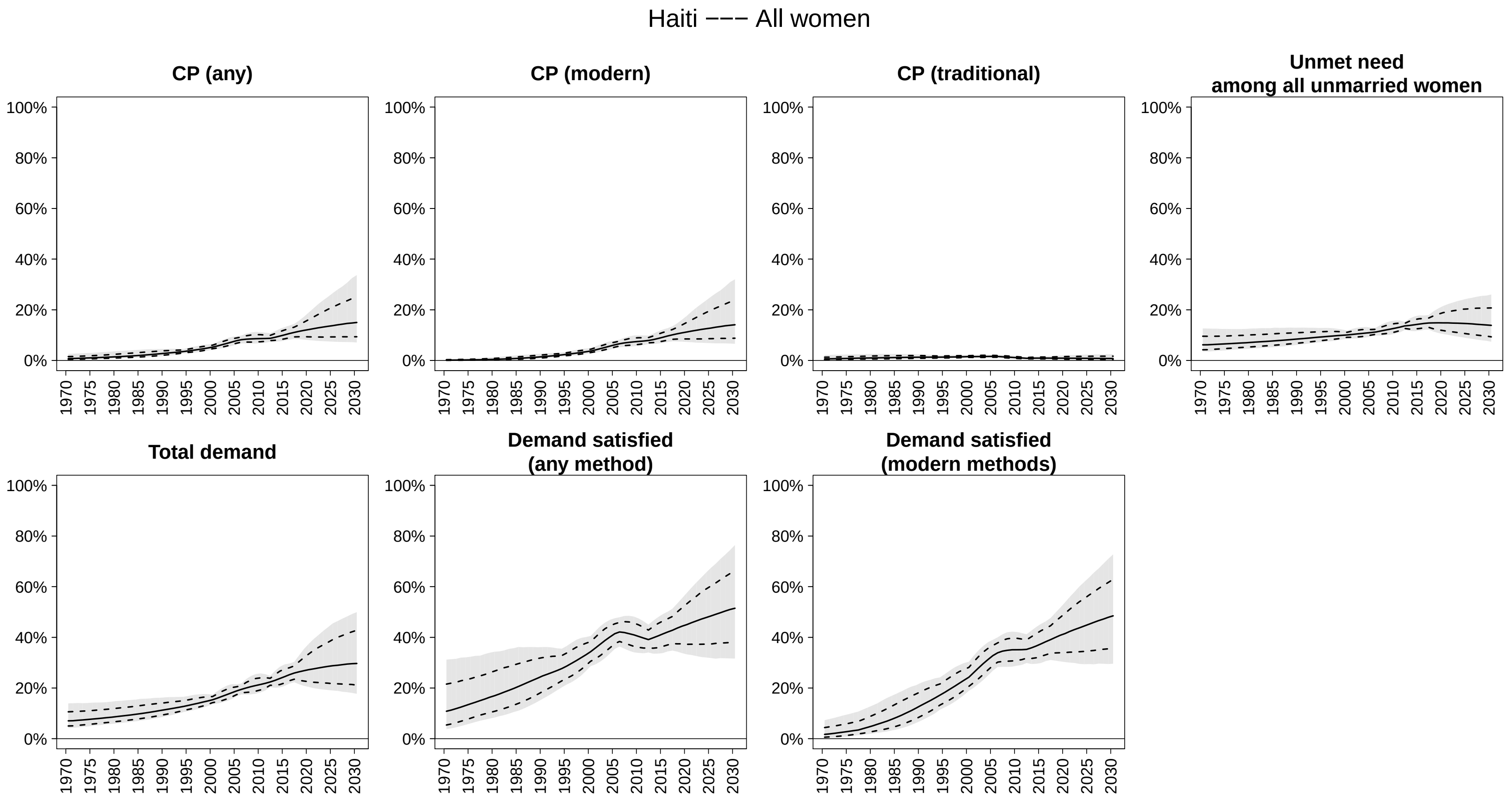
Haiti −−− All women
CP (any)
CP (modern)
CP (traditional)
Unmet need among all unmarried women
Total demand
Demand satisfied (any method)
Demand satisfied (modern methods)
0%
20%
40%
60%
80%
100%
1970
1975
1980
1985
1990
1995
2000
2005
2010
2015
2020
2025
2030

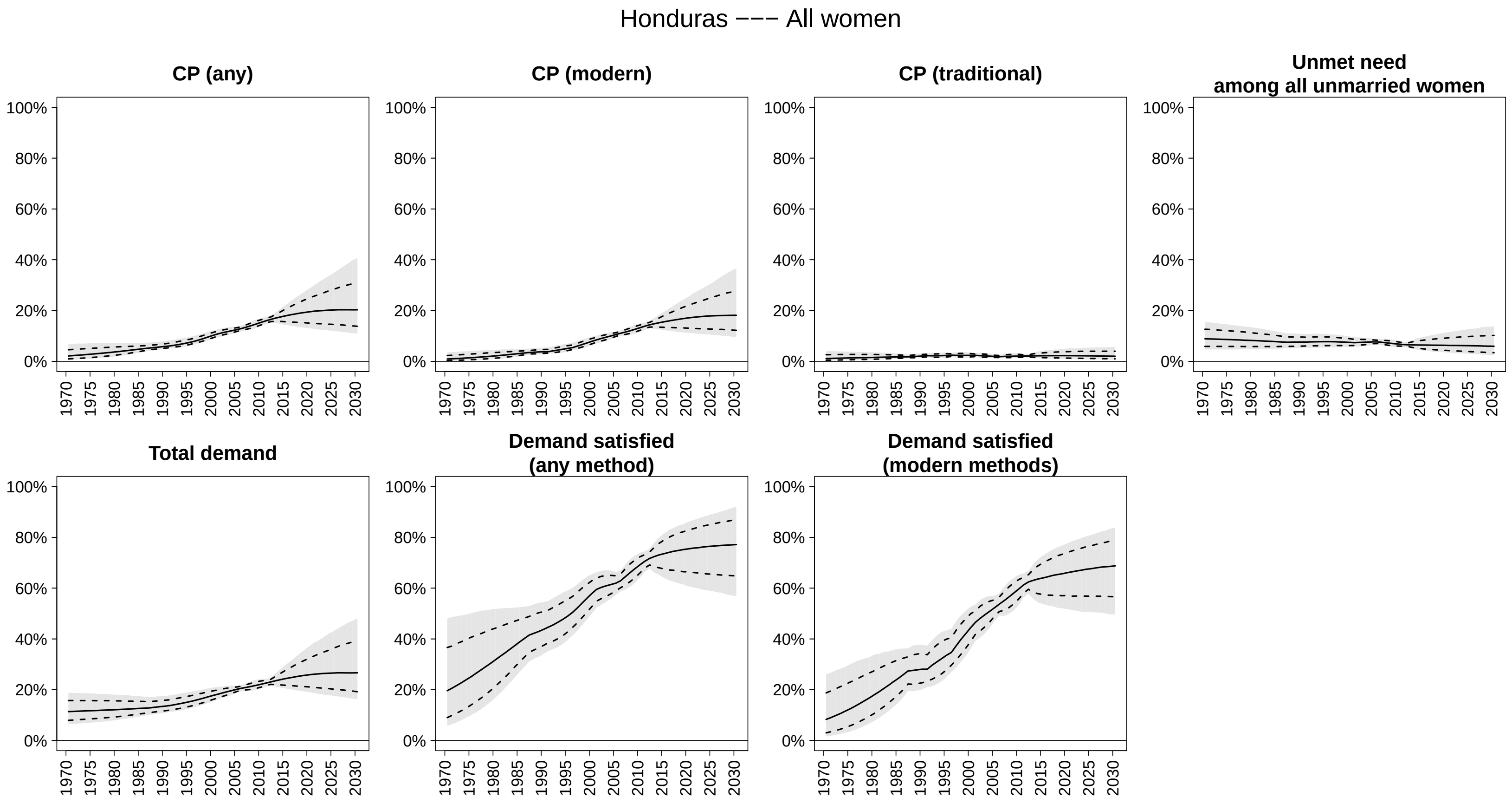
Honduras −−− All women
CP (any)
CP (modern)
CP (traditional)
Unmet need among all unmarried women
Total demand
Demand satisfied (any method)
Demand satisfied (modern methods)
0%
20%
40%
60%
80%
100%
1970
1975
1980
1985
1990
1995
2000
2005
2010
2015
2020
2025
2030

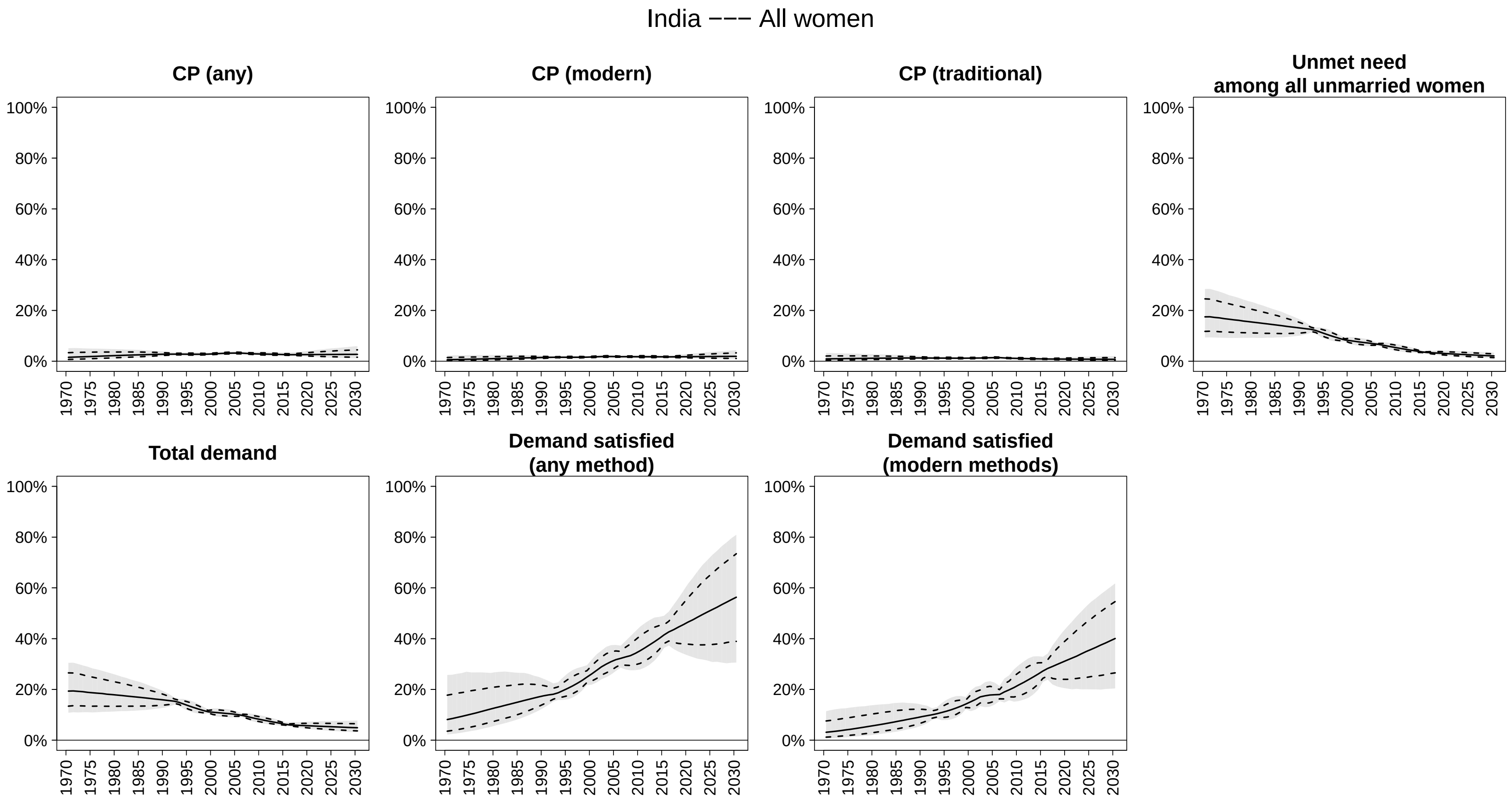
India −−− All women
CP (any)
CP (modern)
CP (traditional)
Unmet need
among all unmarried women
Total demand
Demand satisfied
(any method)
Demand satisfied
(modern methods)
0%
20%
40%
60%
80%
100%
1970
1975
1980
1985
1990
1995
2000
2005
2010
2015
2020
2025
2030

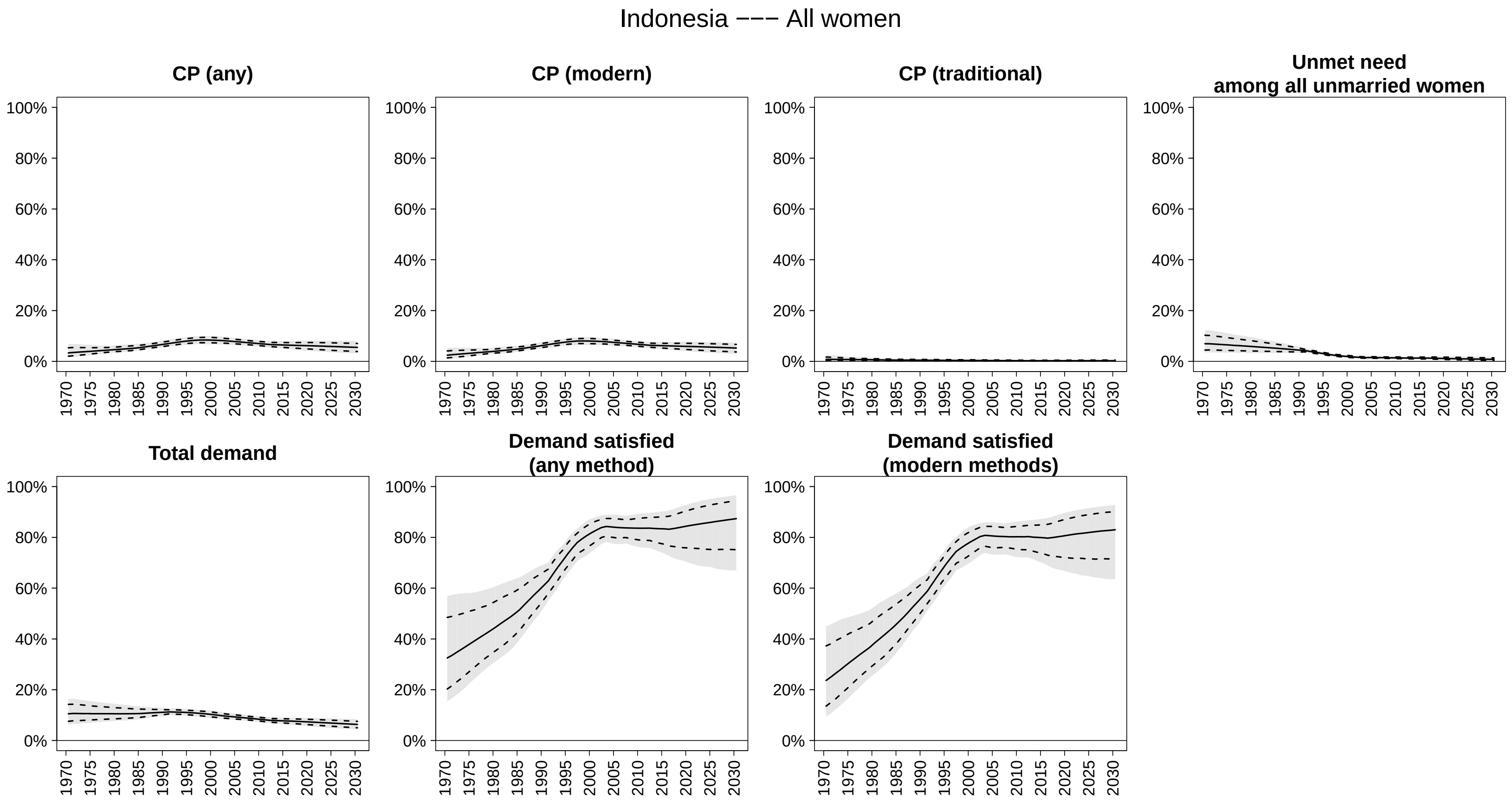
Indonesia −−− All women
CP (any)
CP (modern)
CP (traditional)
Unmet need among all unmarried women
Total demand
Demand satisfied (any method)
Demand satisfied (modern methods)
0%
20%
40%
60%
80%
100%
1970
1975
1980
1985
1990
1995
2000
2005
2010
2015
2020
2025
2030

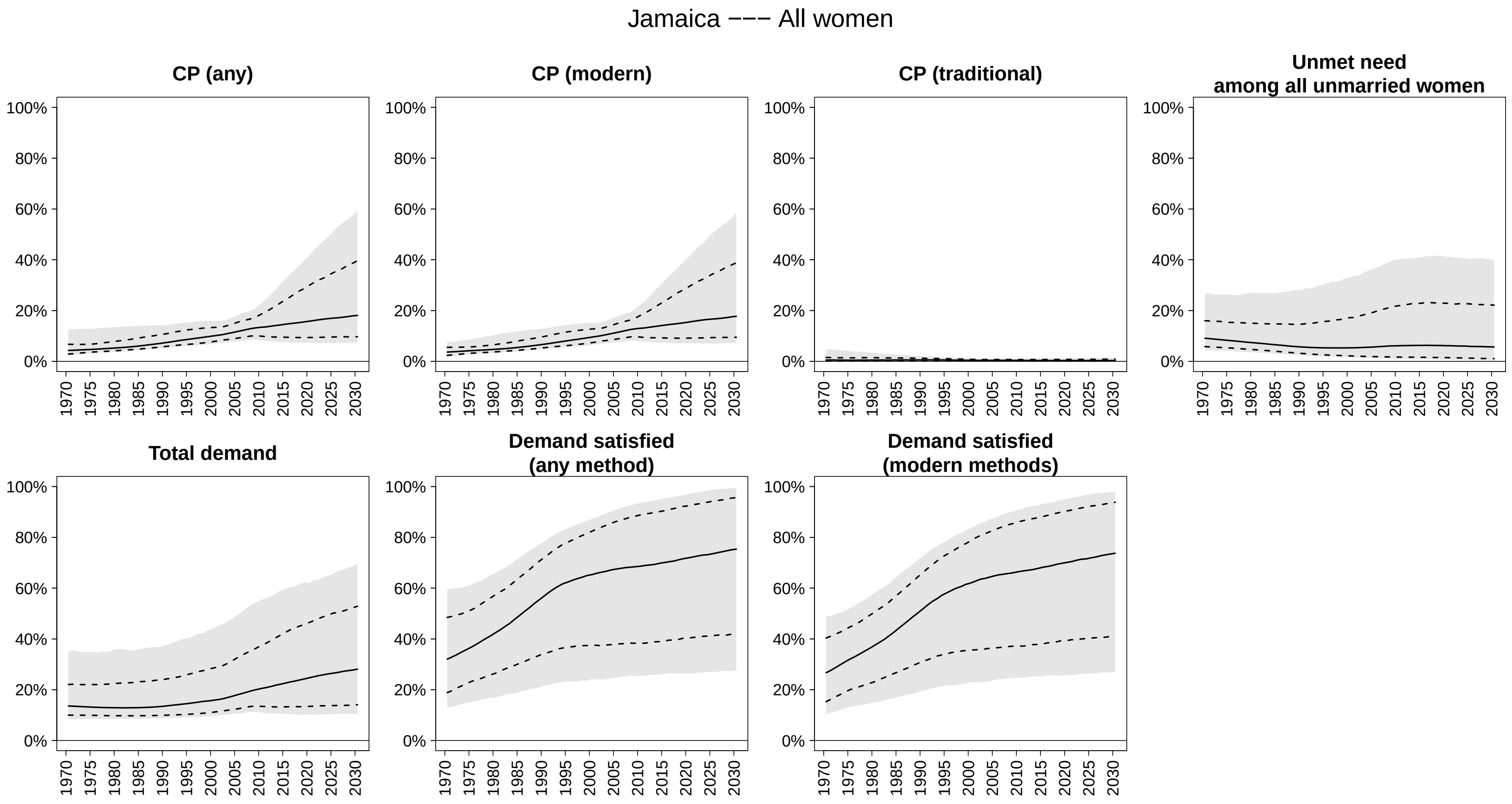
Jamaica −−− All women
CP (any)
CP (modern)
CP (traditional)
Unmet need
among all unmarried women
Total demand
Demand satisfied
(any method)
Demand satisfied
(modern methods)
0%
20%
40%
60%
80%
100%
1970
1975
1980
1985
1990
1995
2000
2005
2010
2015
2020
2025
2030

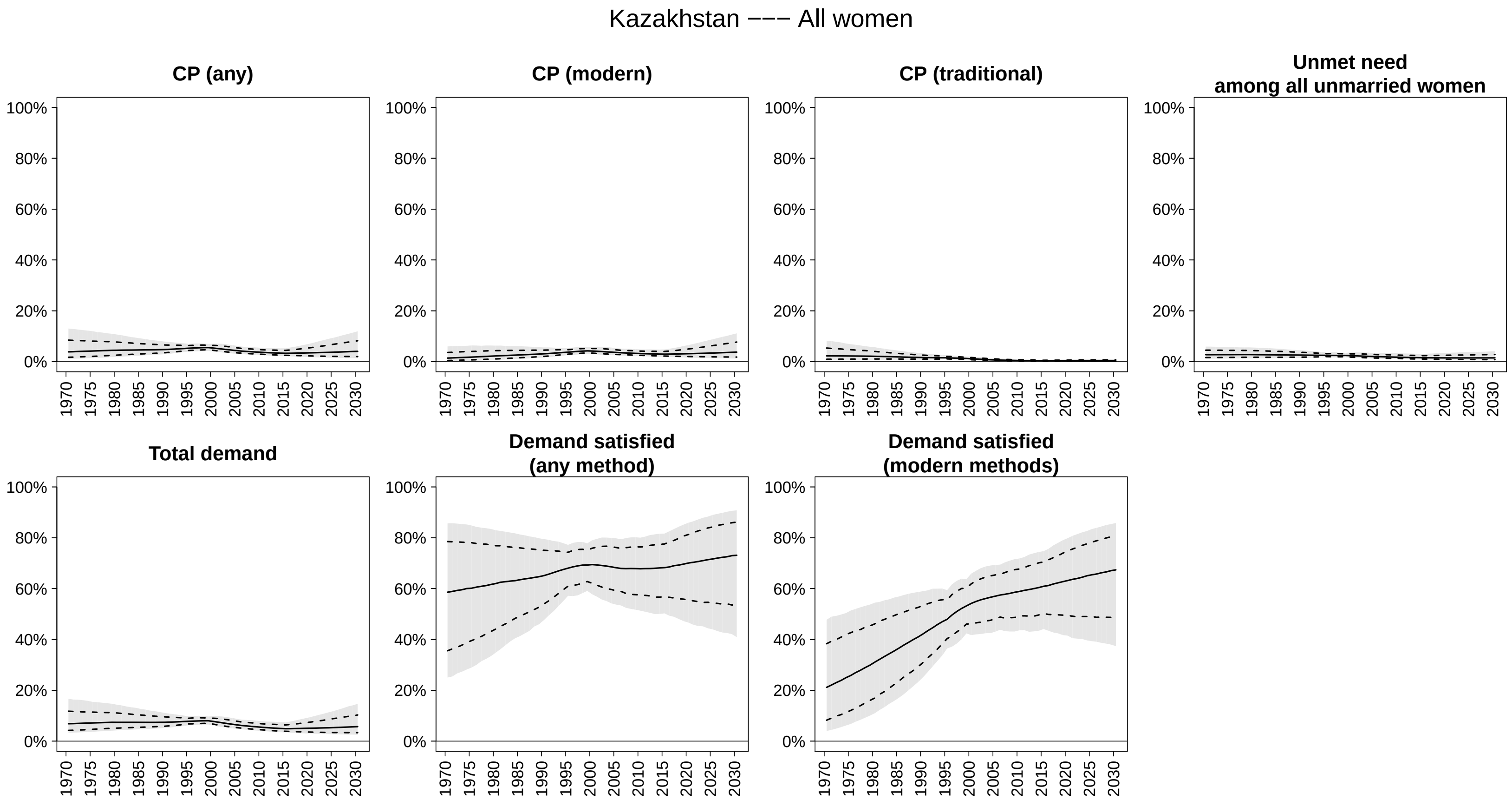
Kazakhstan −−− All women
CP (any)
CP (modern)
CP (traditional)
Unmet need among all unmarried women
Total demand
Demand satisfied (any method)
Demand satisfied (modern methods)
0%
20%
40%
60%
80%
100%
1970
1975
1980
1985
1990
1995
2000
2005
2010
2015
2020
2025
2030

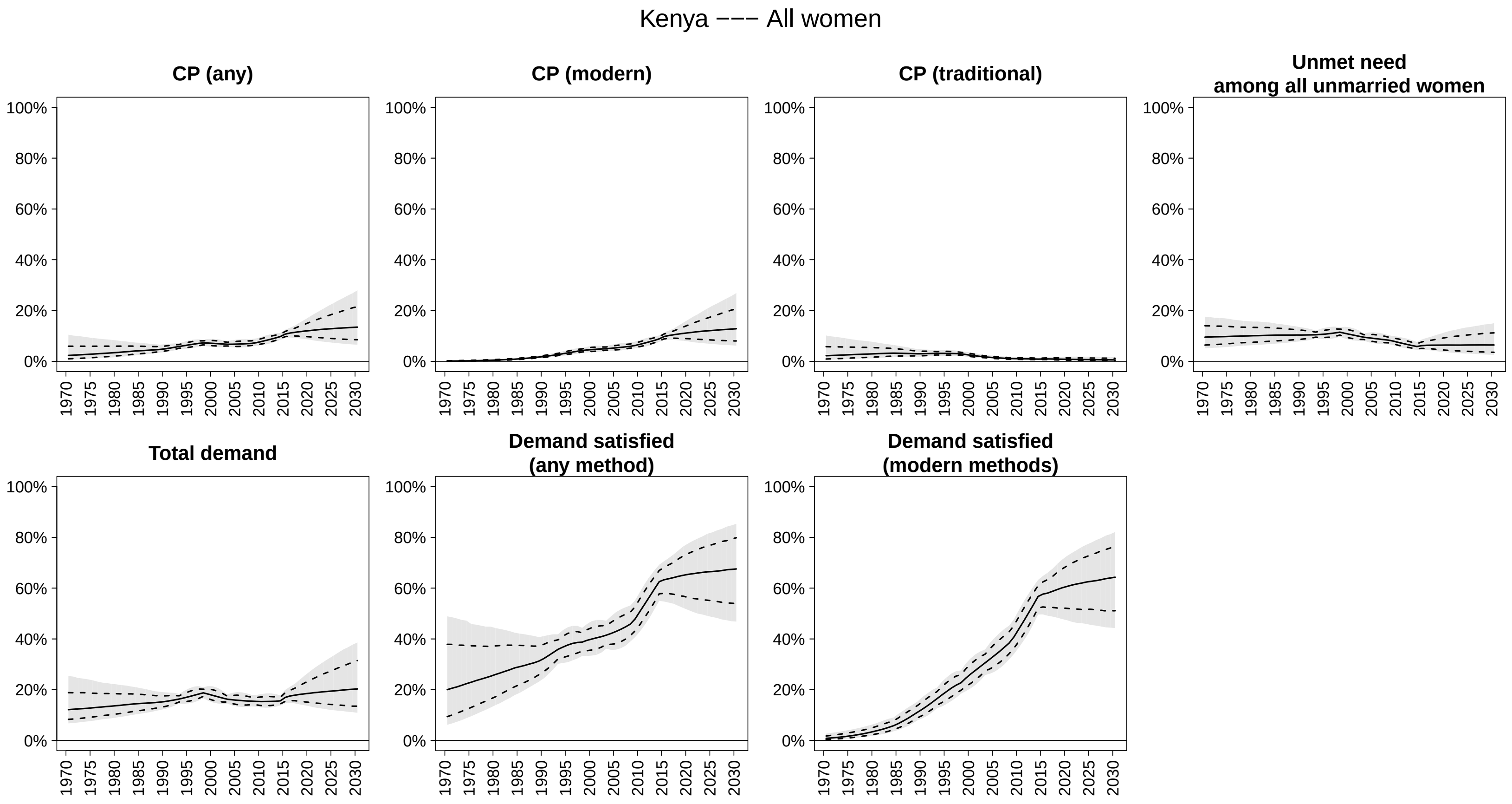
Kenya −−− All women
CP (any)
CP (modern)
CP (traditional)
Unmet need
among all unmarried women
Total demand
Demand satisfied
(any method)
Demand satisfied
(modern methods)
100%
80%
60%
40%
20%
0%
1970
1975
1980
1985
1990
1995
2000
2005
2010
2015
2020
2025
2030

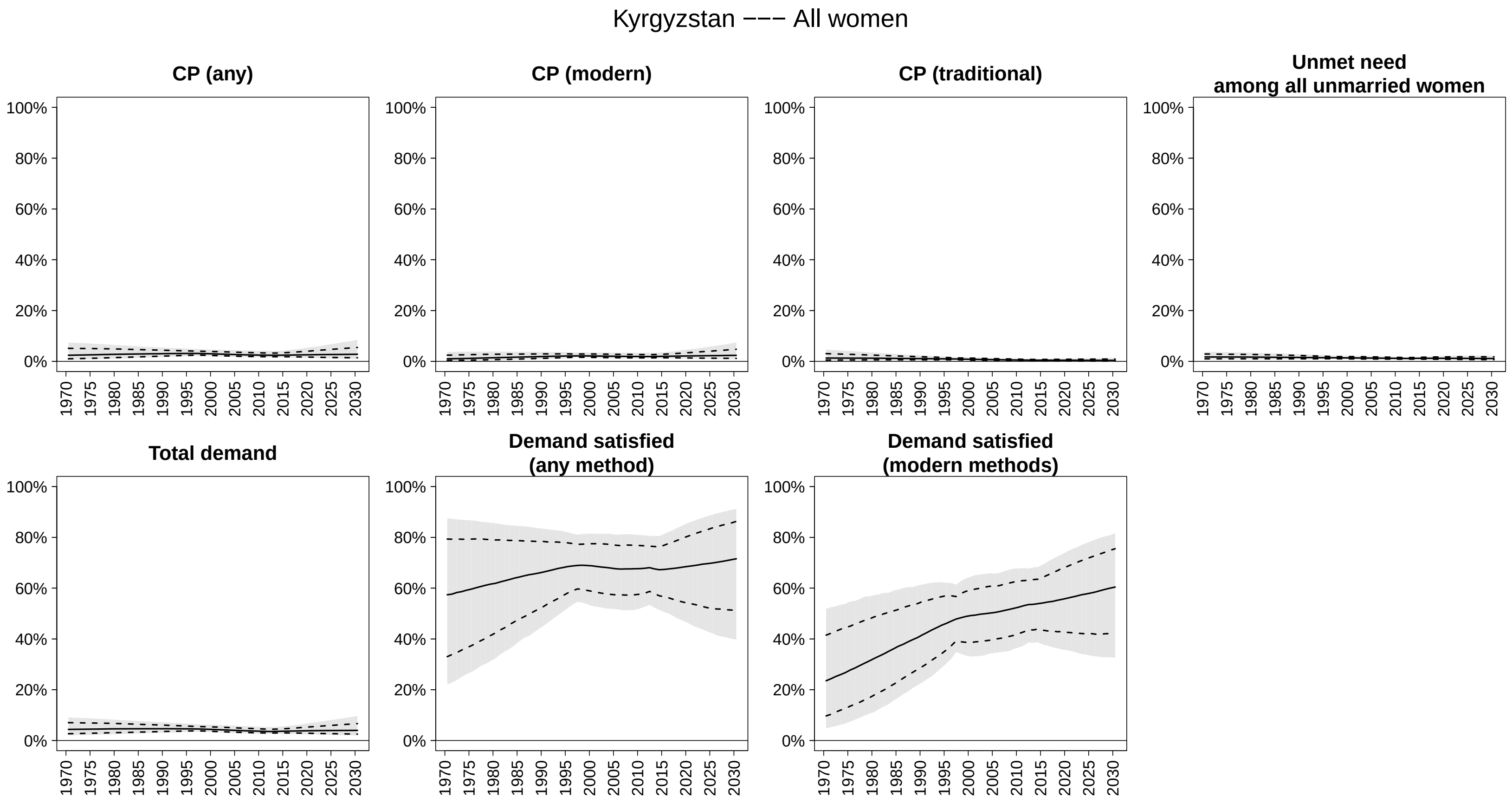
Kyrgyzstan −−− All women
CP (any)
CP (modern)
CP (traditional)
Unmet need among all unmarried women
Total demand
Demand satisfied (any method)
Demand satisfied (modern methods)
0%
20%
40%
60%
80%
100%
1970
1975
1980
1985
1990
1995
2000
2005
2010
2015
2020
2025
2030

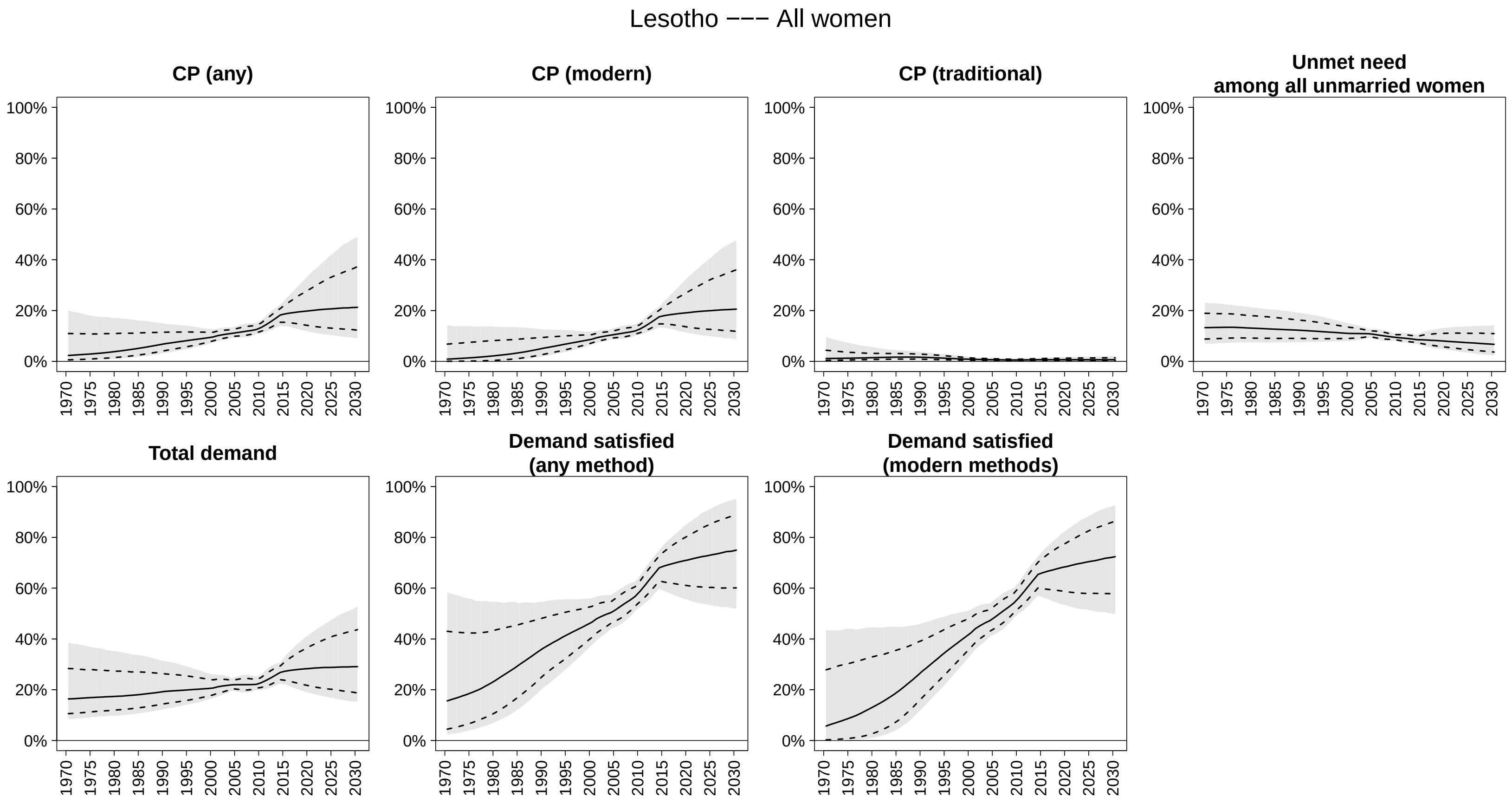
Lesotho −−− All women
CP (any)
CP (modern)
CP (traditional)
Unmet need
among all unmarried women
Total demand
Demand satisfied
(any method)
Demand satisfied
(modern methods)
0%
20%
40%
60%
80%
100%
1970
1975
1980
1985
1990
1995
2000
2005
2010
2015
2020
2025
2030

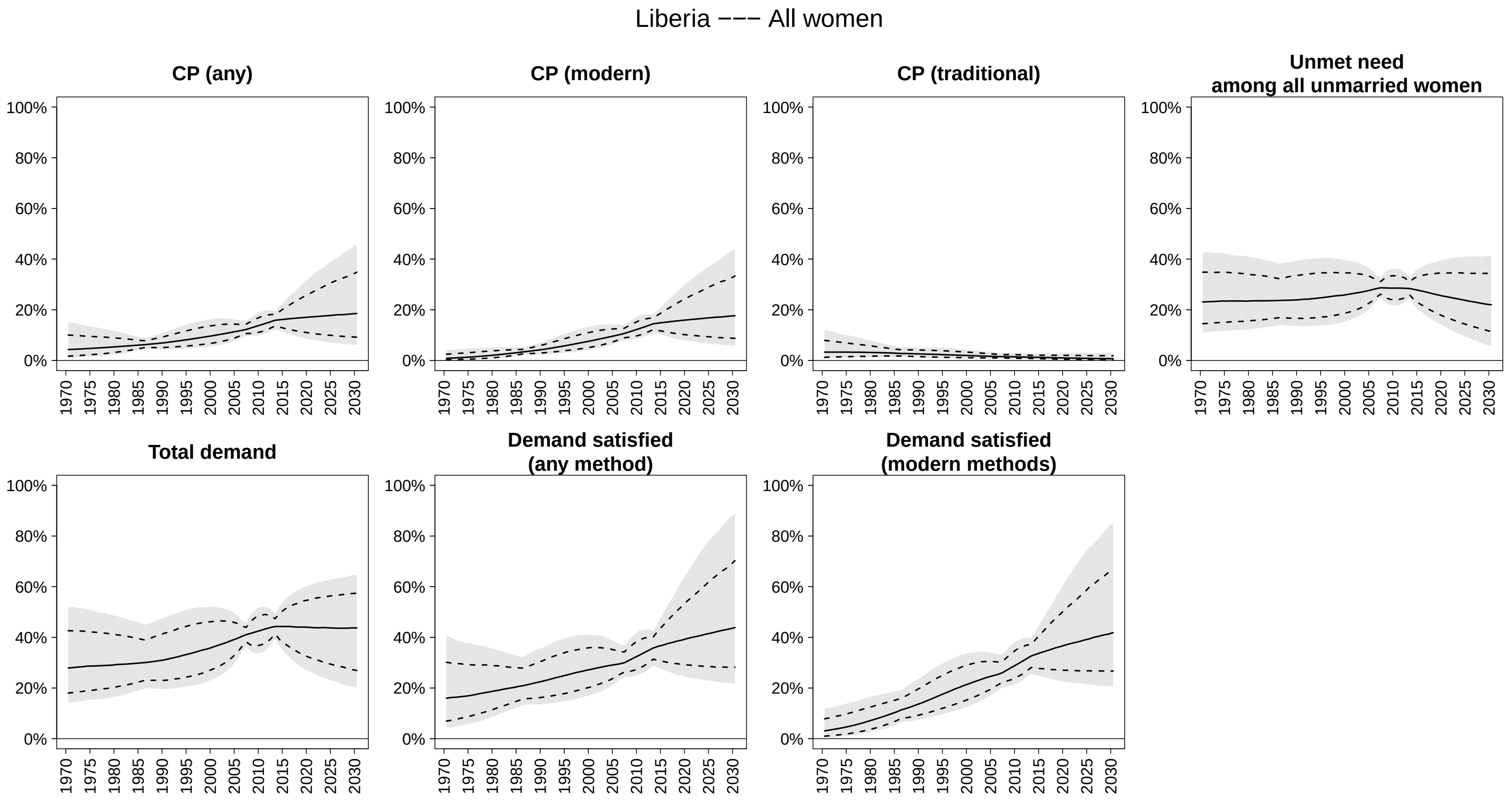
Liberia --- All women
CP (any)
CP (modern)
CP (traditional)
Unmet need among all unmarried women
Total demand
Demand satisfied (any method)
Demand satisfied (modern methods)
100%
80%
60%
40%
20%
0%
1970
1975
1980
1985
1990
1995
2000
2005
2010
2015
2020
2025
2030

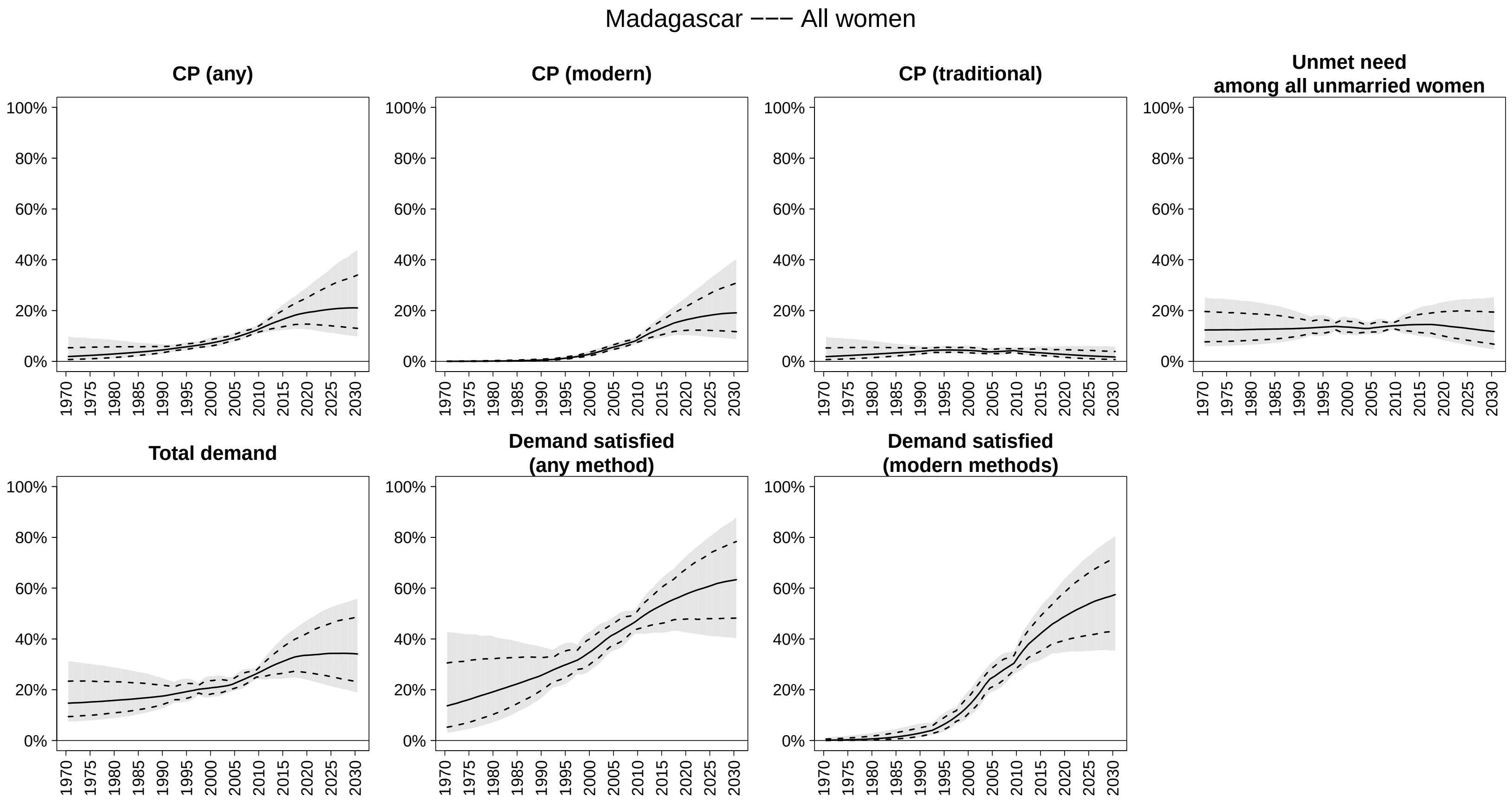
Madagascar −−− All women
CP (any)
CP (modern)
CP (traditional)
Unmet need among all unmarried women
Total demand
Demand satisfied (any method)
Demand satisfied (modern methods)
0%
20%
40%
60%
80%
100%
1970
1975
1980
1985
1990
1995
2000
2005
2010
2015
2020
2025
2030

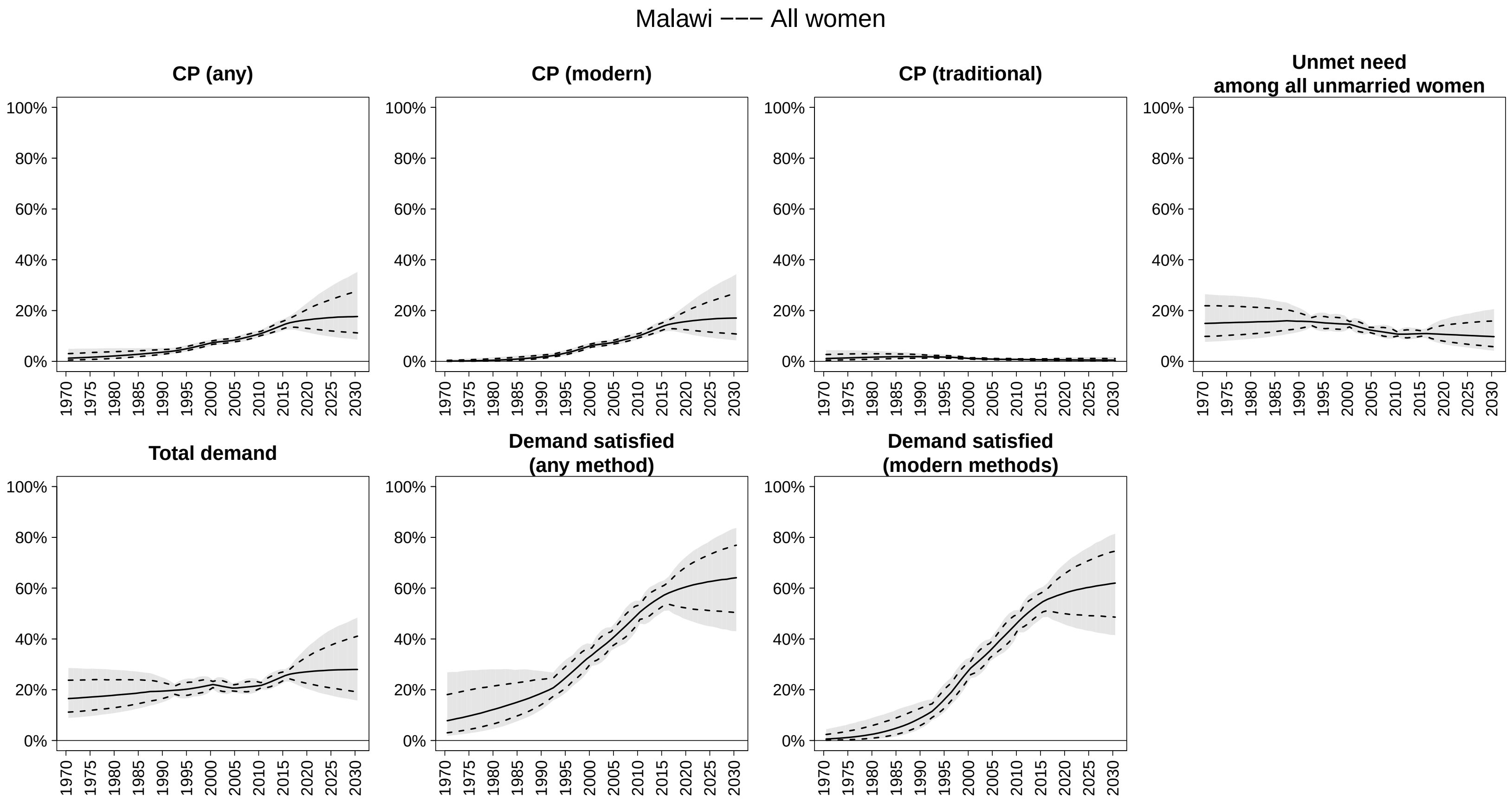
Malawi −−− All women
CP (any)
CP (modern)
CP (traditional)
Unmet need
among all unmarried women
Total demand
Demand satisfied
(any method)
Demand satisfied
(modern methods)
0%
20%
40%
60%
80%
100%
1970
1975
1980
1985
1990
1995
2000
2005
2010
2015
2020
2025
2030

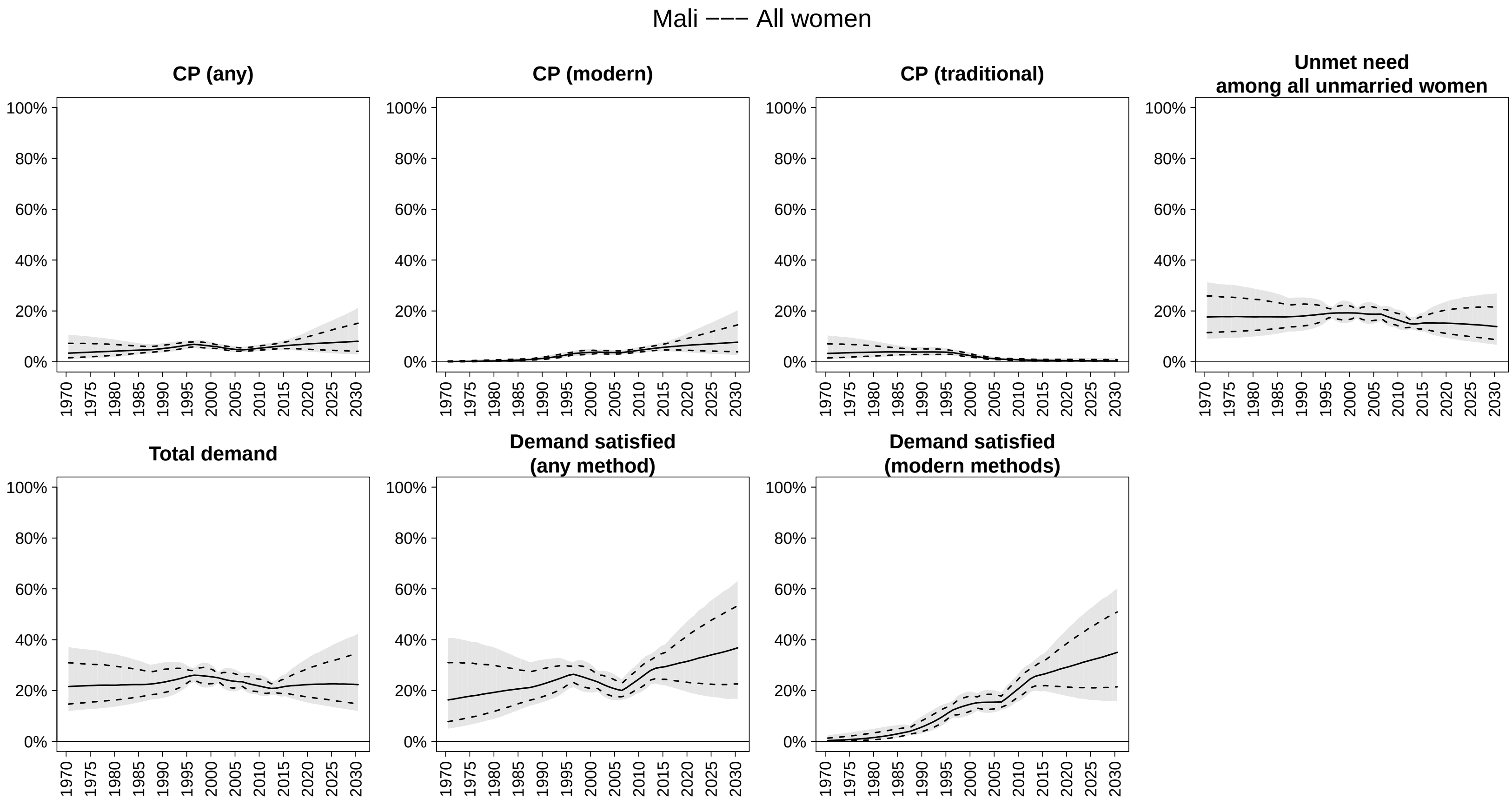
Mali --- All women
CP (any)
CP (modern)
CP (traditional)
Unmet need
among all unmarried women
Total demand
Demand satisfied
(any method)
Demand satisfied
(modern methods)
0%
20%
40%
60%
80%
100%
1970
1975
1980
1985
1990
1995
2000
2005
2010
2015
2020
2025
2030

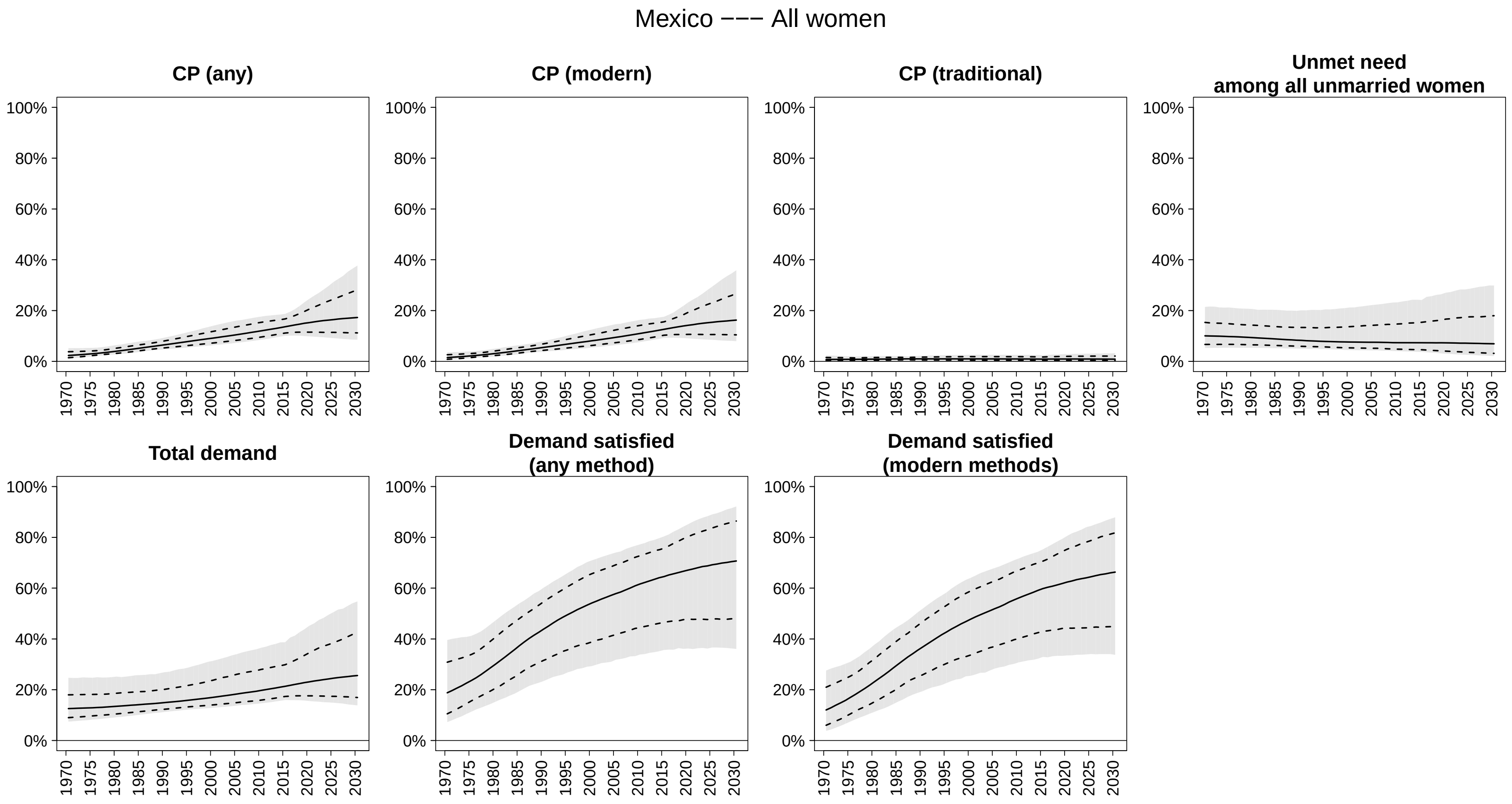

Mexico ——— All women
CP (any)
CP (modern)
CP (traditional)
Unmet need
among all unmarried women
Total demand
Demand satisfied
(any method)
Demand satisfied
(modern methods)
0%
20%
40%
60%
80%
100%
1970
1975
1980
1985
1990
1995
2000
2005
2010
2015
2020
2025
2030

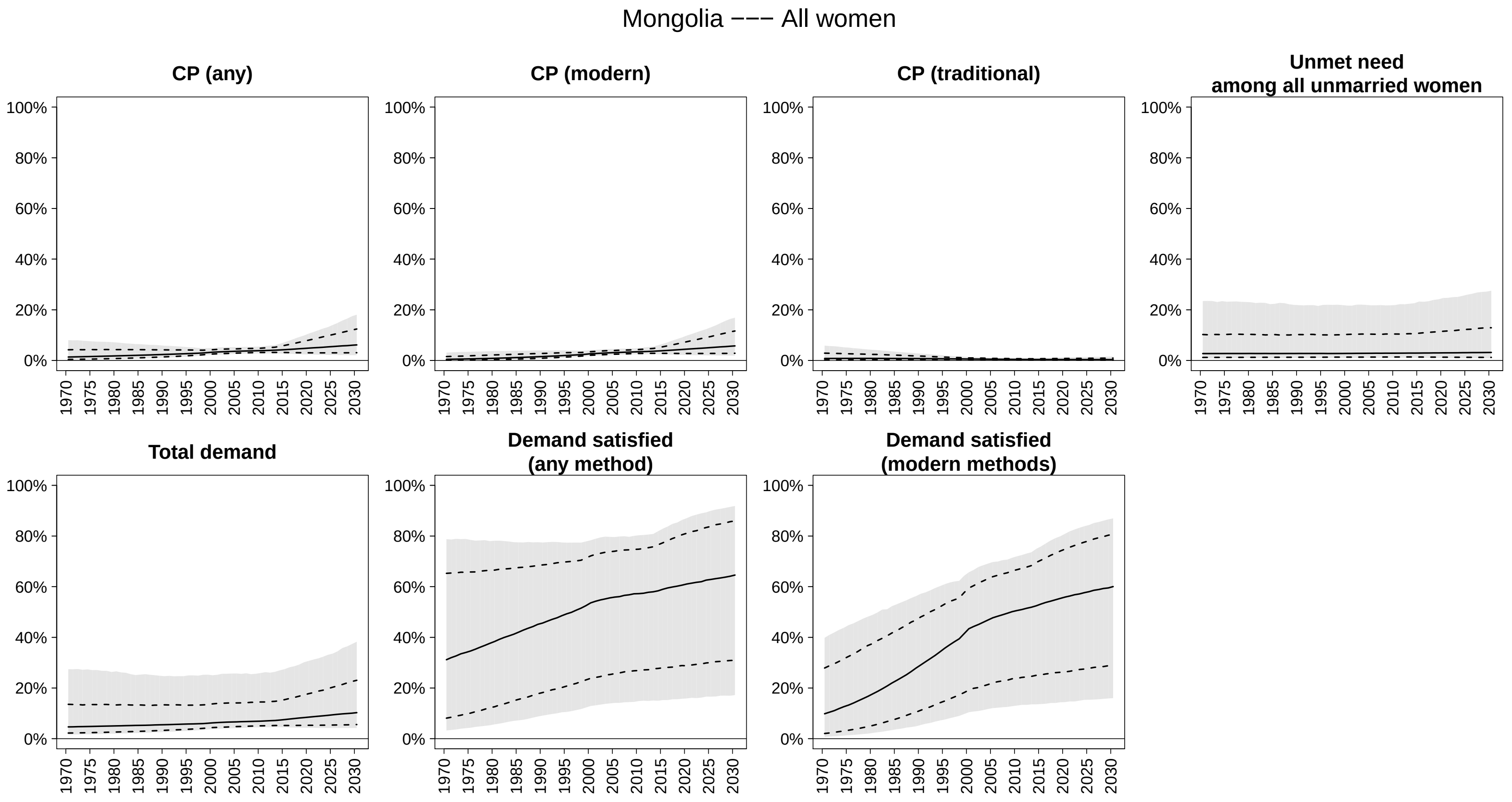
Mongolia −−− All women
CP (any)
CP (modern)
CP (traditional)
Unmet need among all unmarried women
Total demand
Demand satisfied (any method)
Demand satisfied (modern methods)
0%
20%
40%
60%
80%
100%
1970
1975
1980
1985
1990
1995
2000
2005
2010
2015
2020
2025
2030

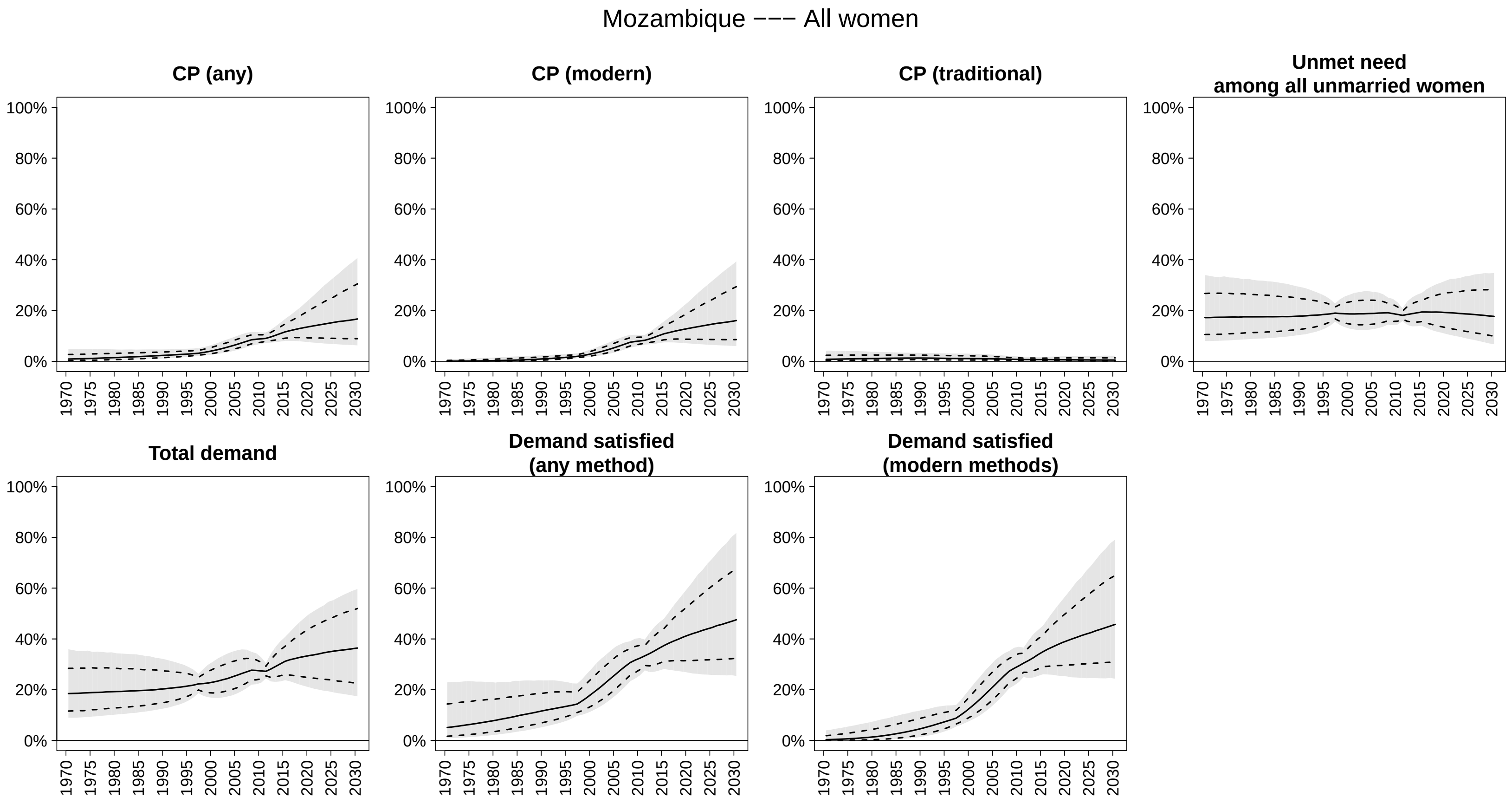
Mozambique −−− All women
CP (any)
CP (modern)
CP (traditional)
Unmet need
among all unmarried women
Total demand
Demand satisfied
(any method)
Demand satisfied
(modern methods)
0%
20%
40%
60%
80%
100%
1970
1975
1980
1985
1990
1995
2000
2005
2010
2015
2020
2025
2030

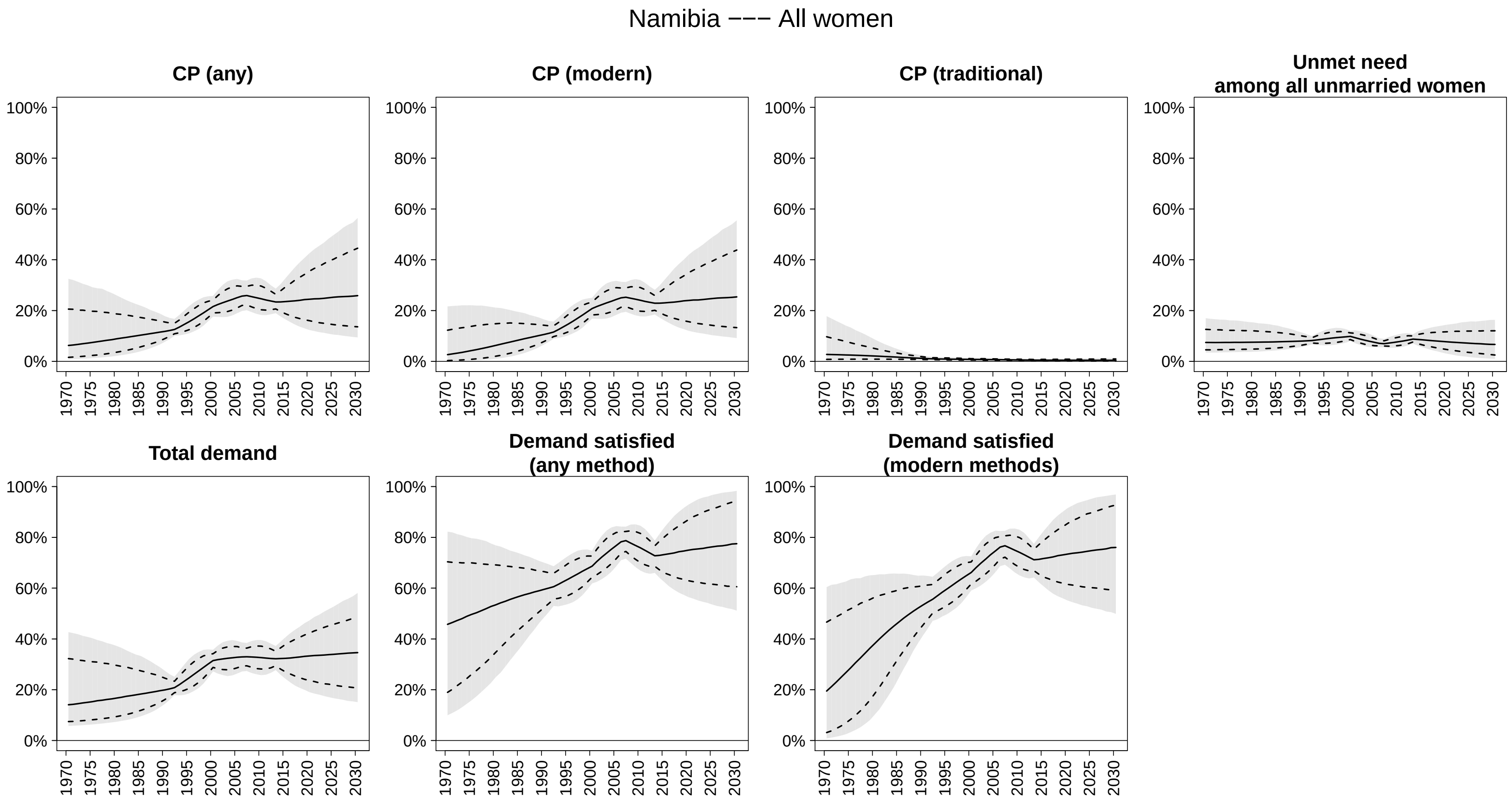
Namibia −−− All women
CP (any)
CP (modern)
CP (traditional)
Unmet need
among all unmarried women
Total demand
Demand satisfied
(any method)
Demand satisfied
(modern methods)
0%
20%
40%
60%
80%
100%
1970
1975
1980
1985
1990
1995
2000
2005
2010
2015
2020
2025
2030

Nepal −−− All women

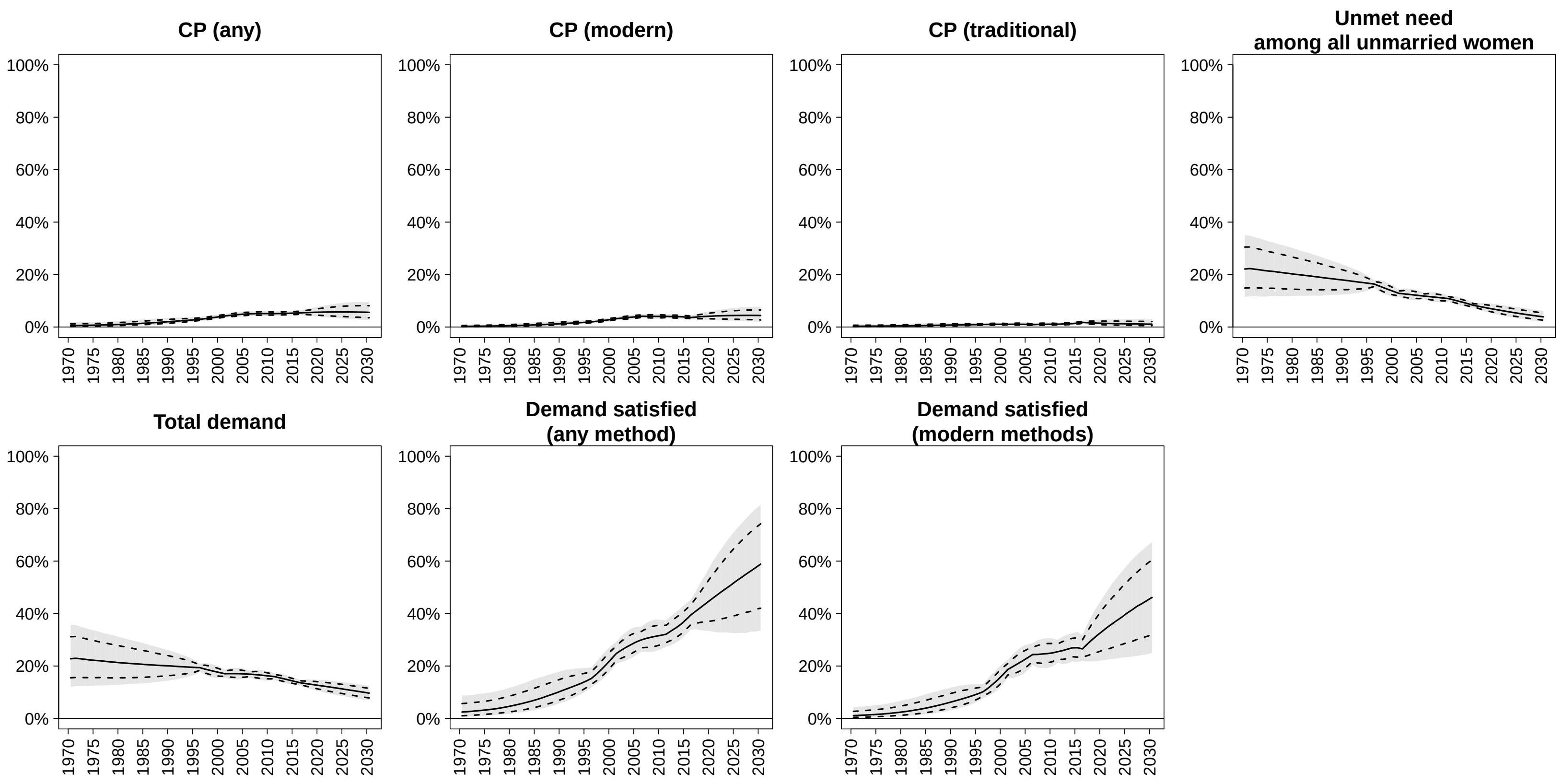

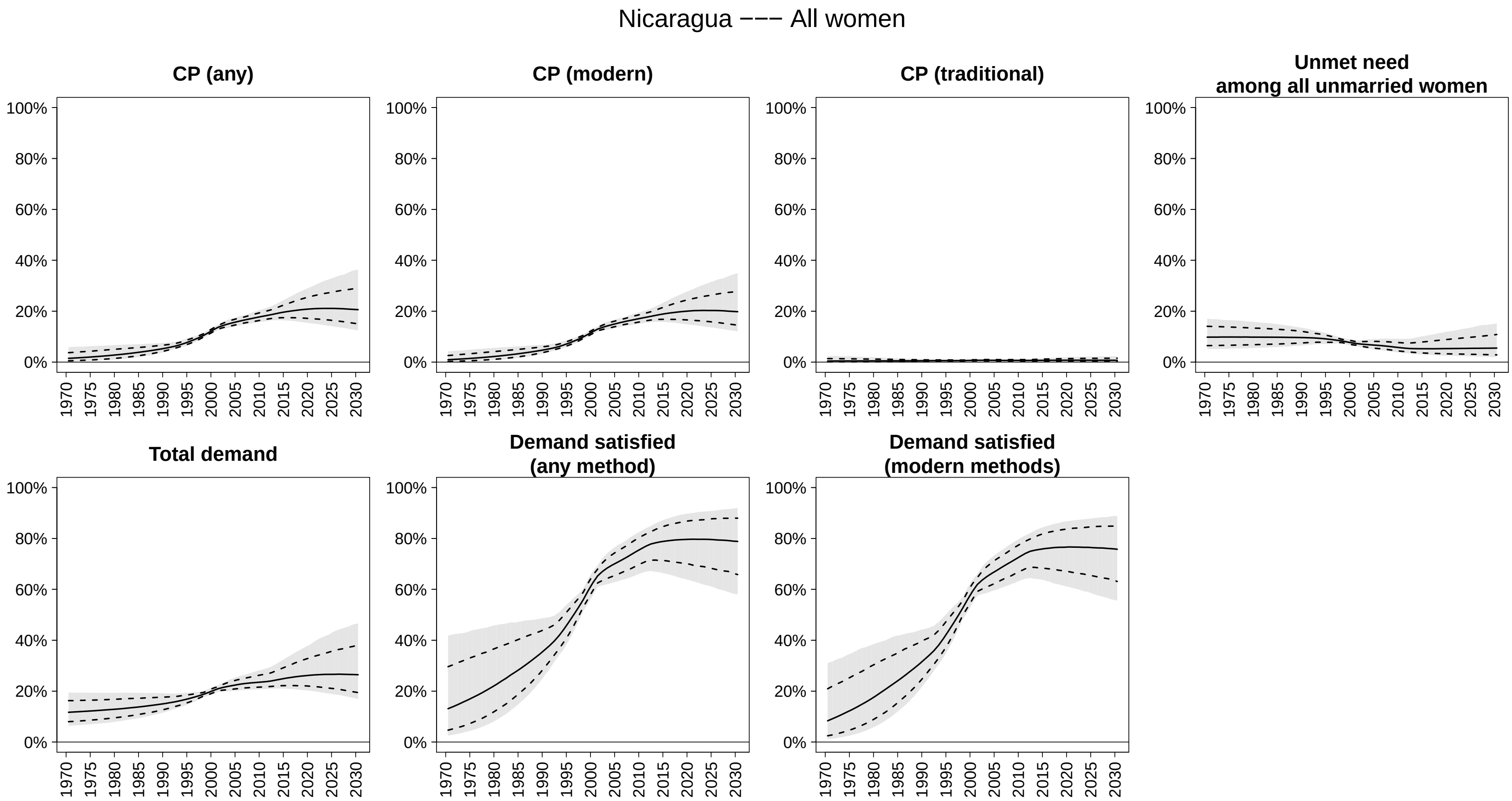
Nicaragua --- All women
CP (any)
CP (modern)
CP (traditional)
Unmet need among all unmarried women
Total demand
Demand satisfied (any method)
Demand satisfied (modern methods)
0%
20%
40%
60%
80%
100%
1970
1975
1980
1985
1990
1995
2000
2005
2010
2015
2020
2025
2030

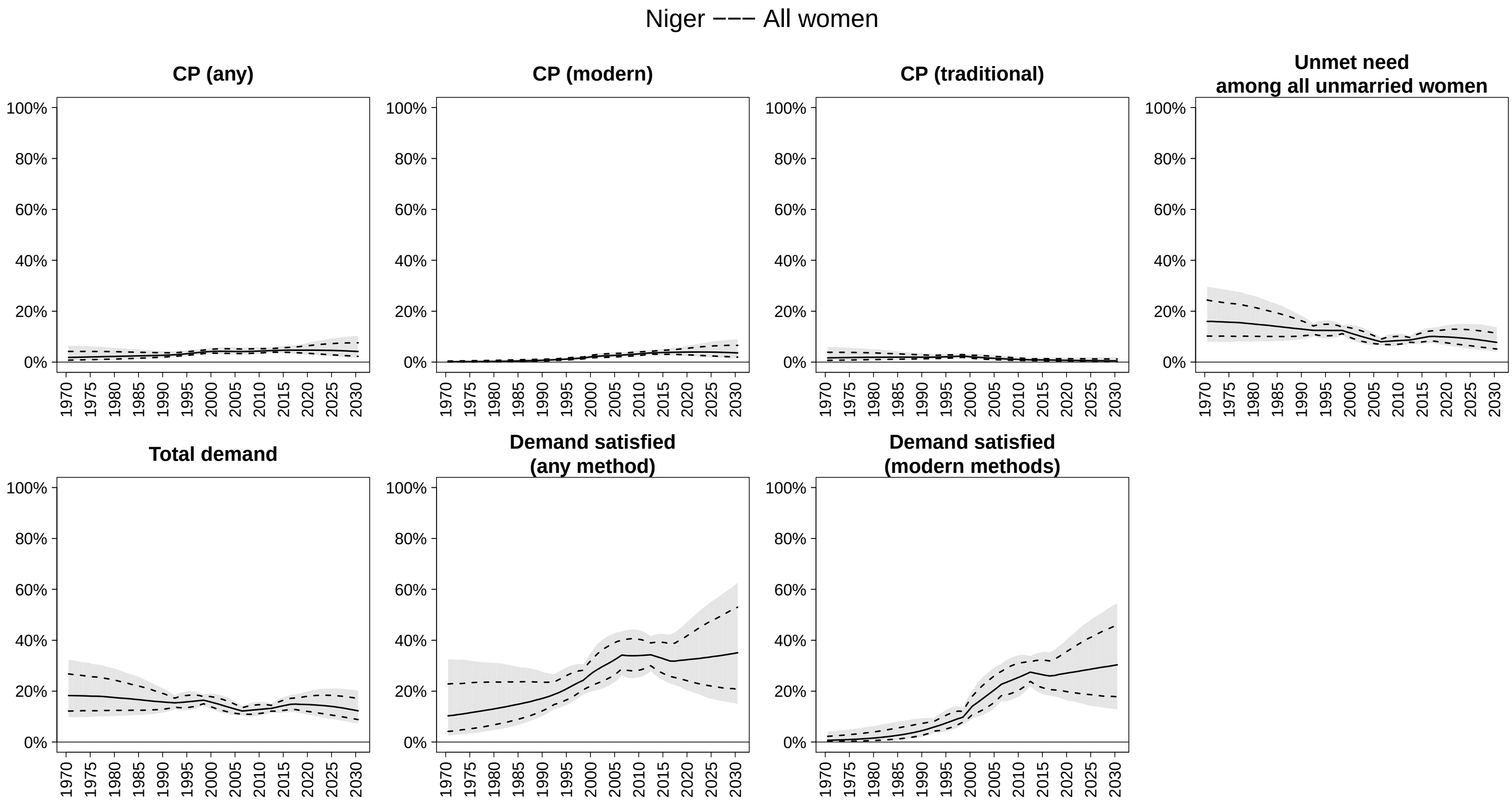
Niger −−− All women
CP (any)
CP (modern)
CP (traditional)
Unmet need among all unmarried women
Total demand
Demand satisfied (any method)
Demand satisfied (modern methods)
0%
20%
40%
60%
80%
100%
1970
1975
1980
1985
1990
1995
2000
2005
2010
2015
2020
2025
2030

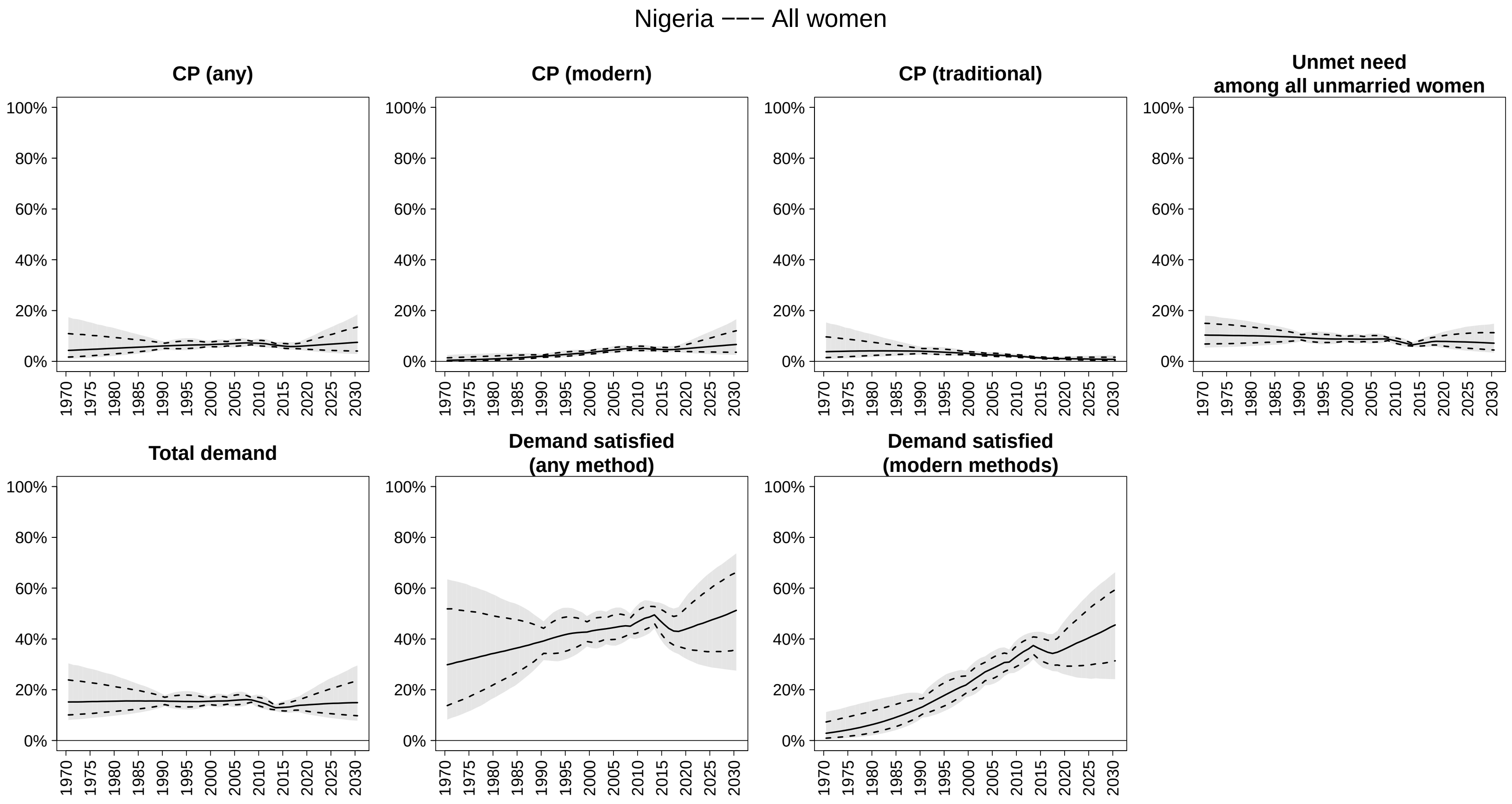
Nigeria −−− All women
CP (any)
CP (modern)
CP (traditional)
Unmet need among all unmarried women
Total demand
Demand satisfied (any method)
Demand satisfied (modern methods)
0%
20%
40%
60%
80%
100%
1970
1975
1980
1985
1990
1995
2000
2005
2010
2015
2020
2025
2030

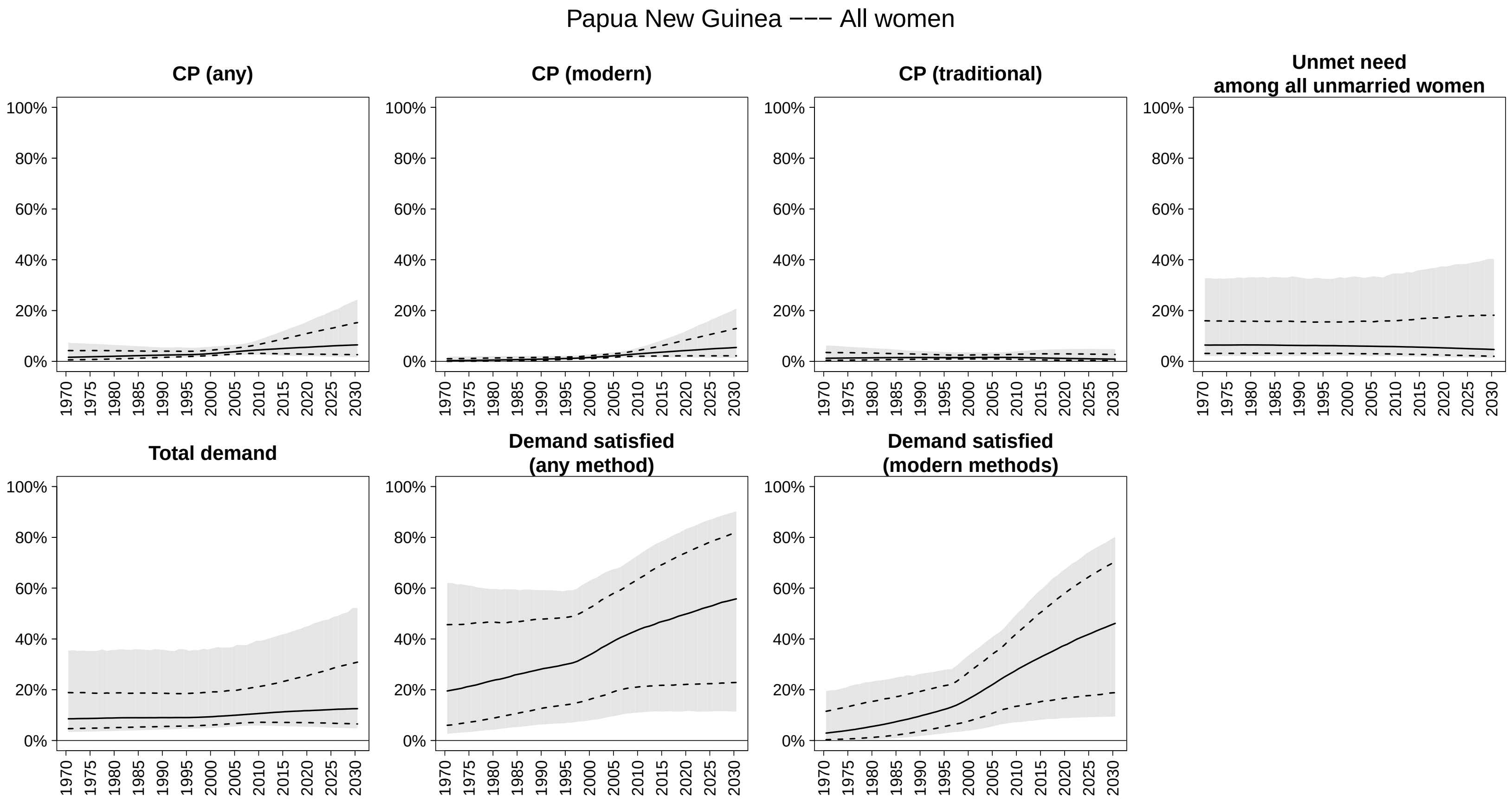
Papua New Guinea −−− All women
CP (any)
CP (modern)
CP (traditional)
Unmet need among all unmarried women
Total demand
Demand satisfied (any method)
Demand satisfied (modern methods)
0%
20%
40%
60%
80%
100%
1970
1975
1980
1985
1990
1995
2000
2005
2010
2015
2020
2025
2030

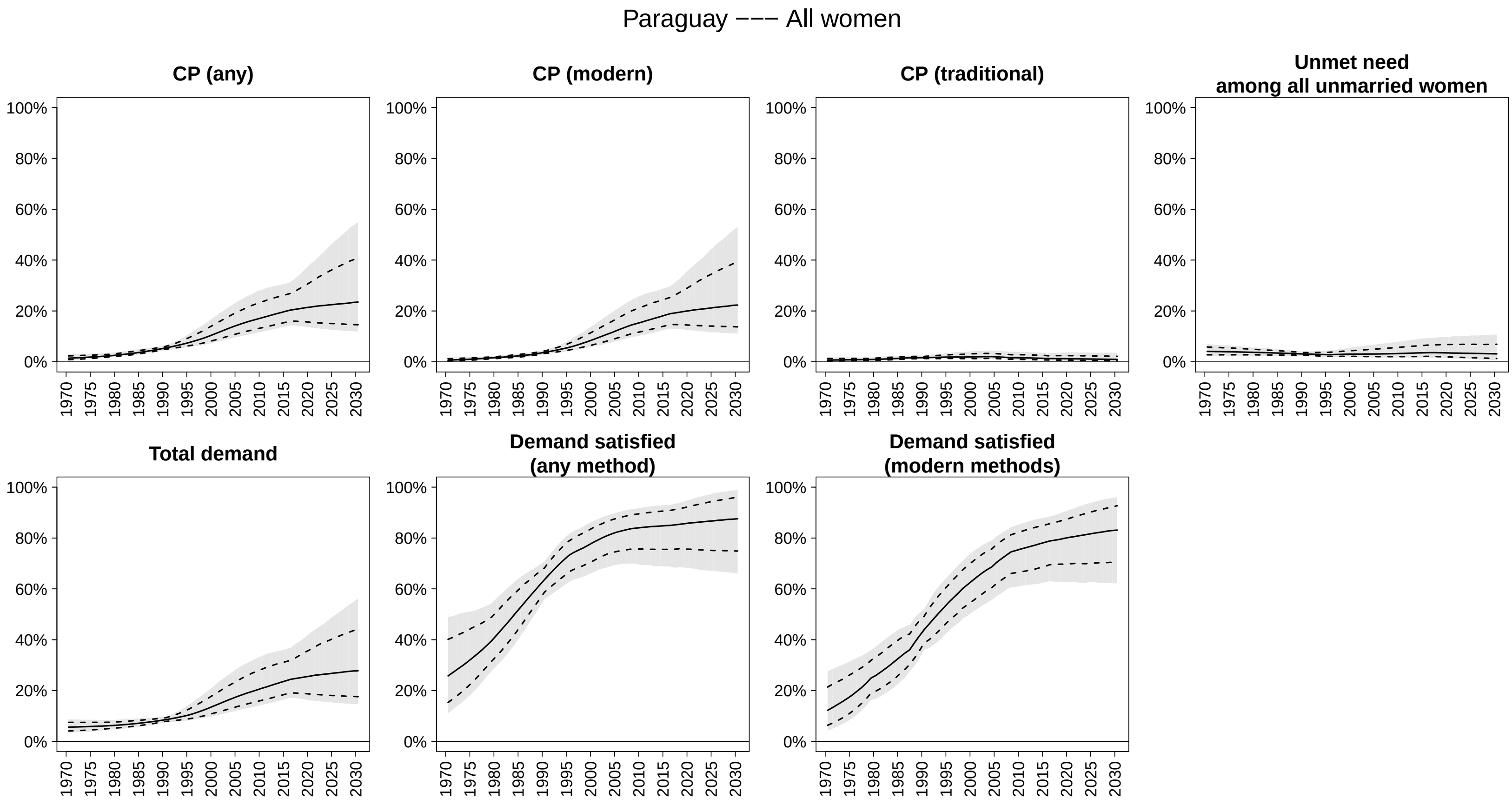
Paraguay −−− All women
CP (any)
CP (modern)
CP (traditional)
Unmet need
among all unmarried women
Total demand
Demand satisfied
(any method)
Demand satisfied
(modern methods)
0%
20%
40%
60%
80%
100%
1970
1975
1980
1985
1990
1995
2000
2005
2010
2015
2020
2025
2030

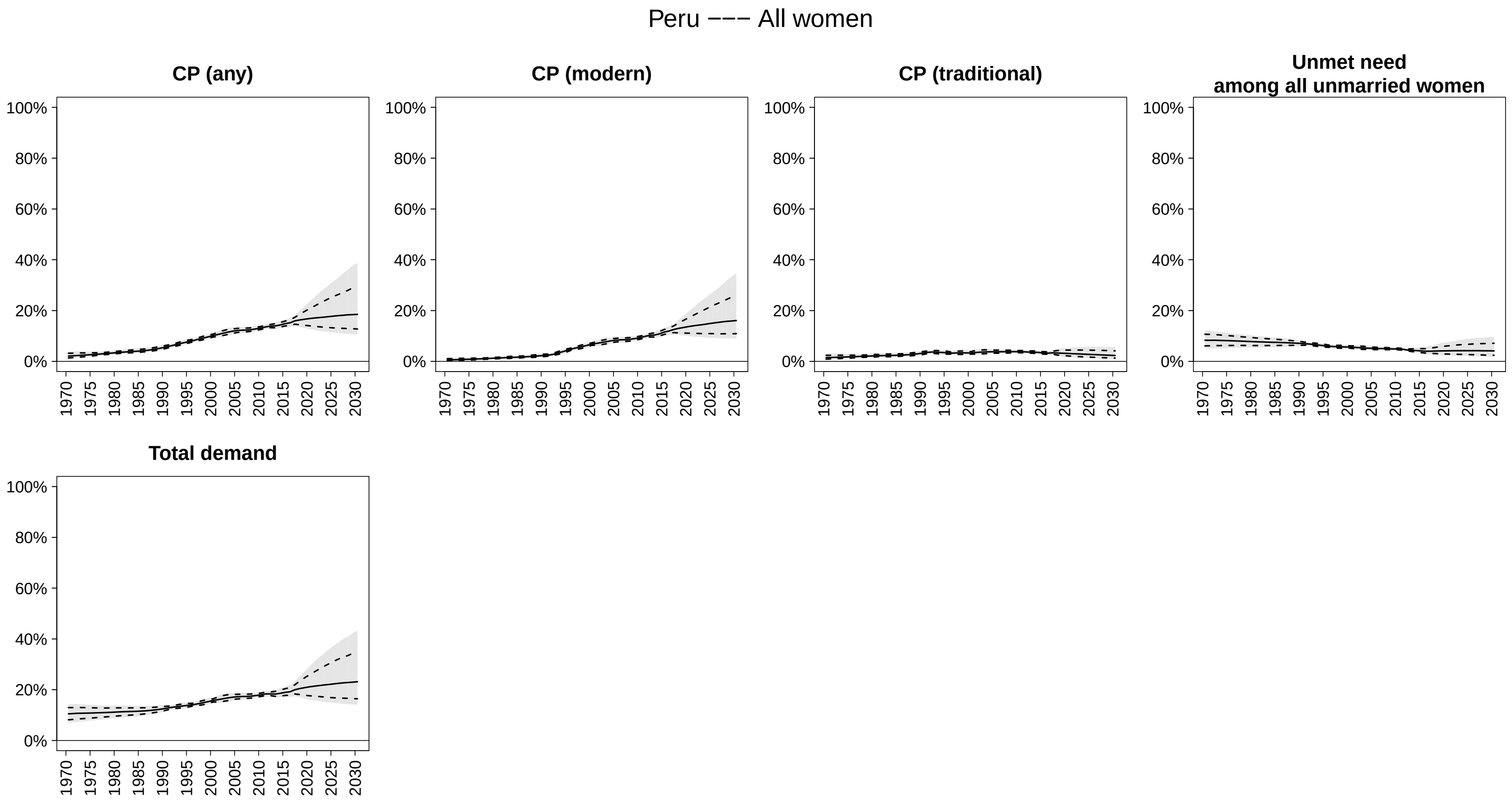
Peru ——— All women
CP (any)
CP (modern)
CP (traditional)
Unmet need
among all unmarried women
Total demand
0%
20%
40%
60%
80%
100%
1970
1975
1980
1985
1990
1995
2000
2005
2010
2015
2020
2025
2030

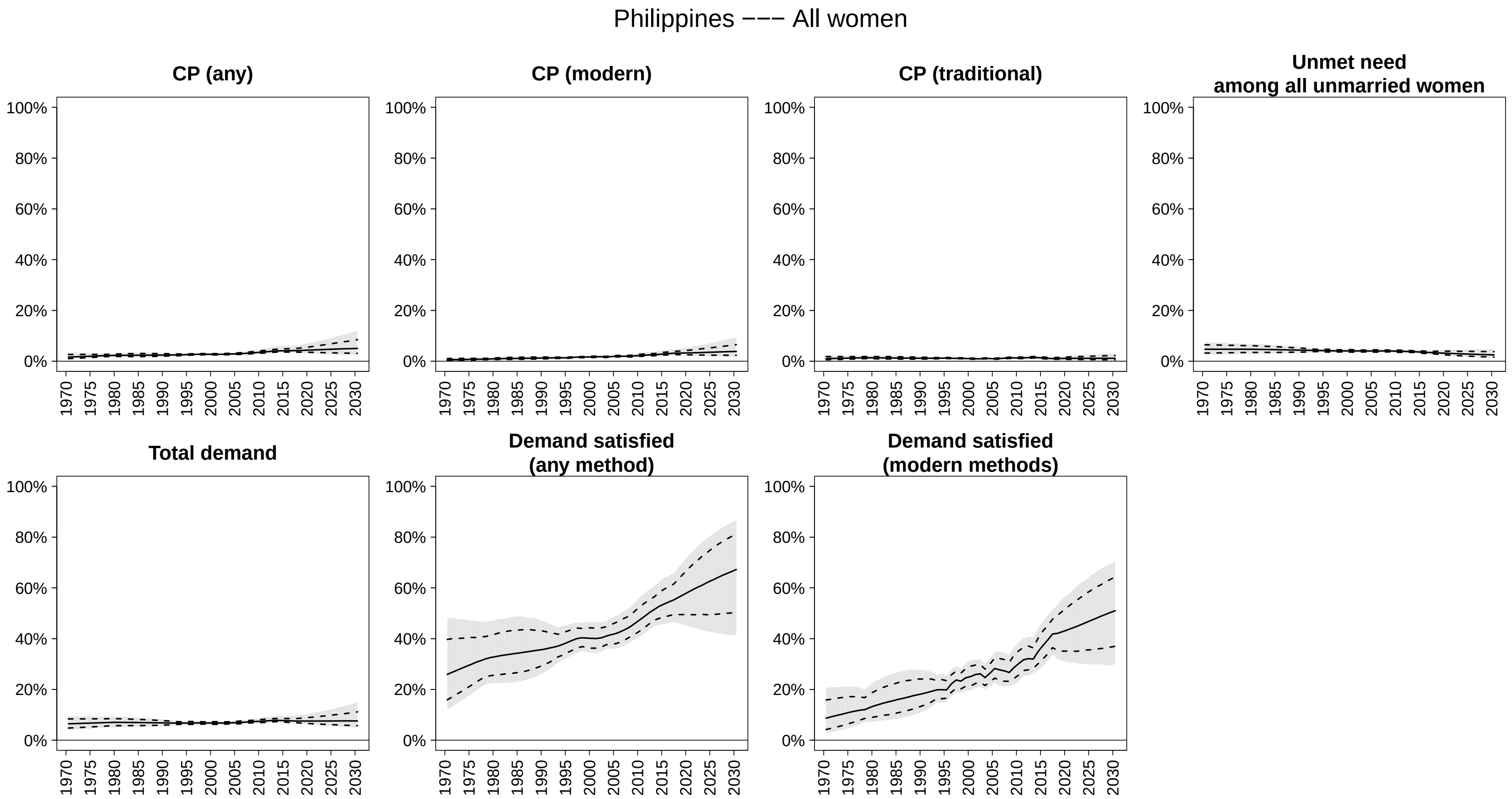
Philippines --- All women
CP (any)
CP (modern)
CP (traditional)
Unmet need among all unmarried women
Total demand
Demand satisfied (any method)
Demand satisfied (modern methods)
0%
20%
40%
60%
80%
100%
1970
1975
1980
1985
1990
1995
2000
2005
2010
2015
2020
2025
2030

Republic of Moldova −−− All women

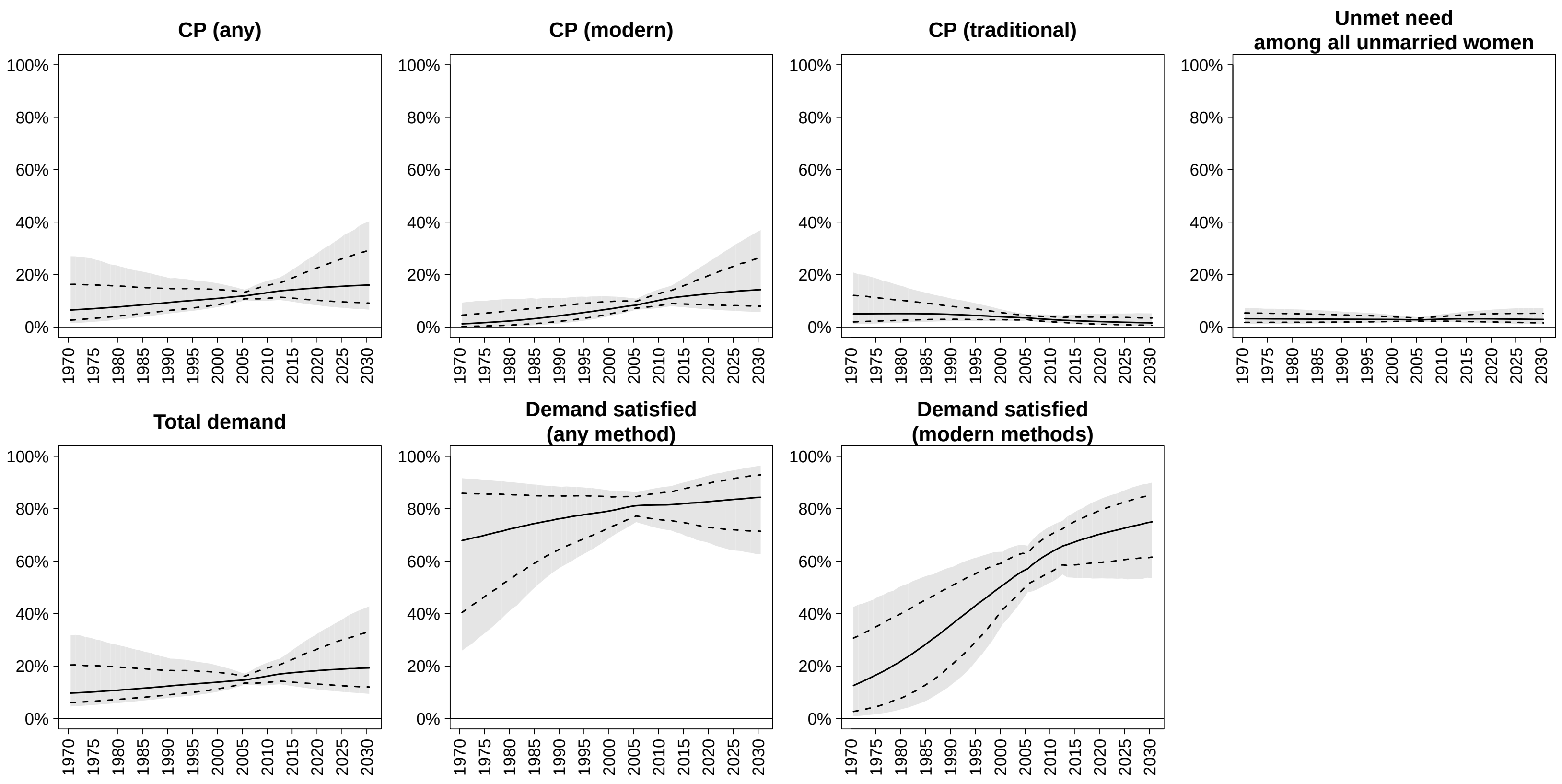

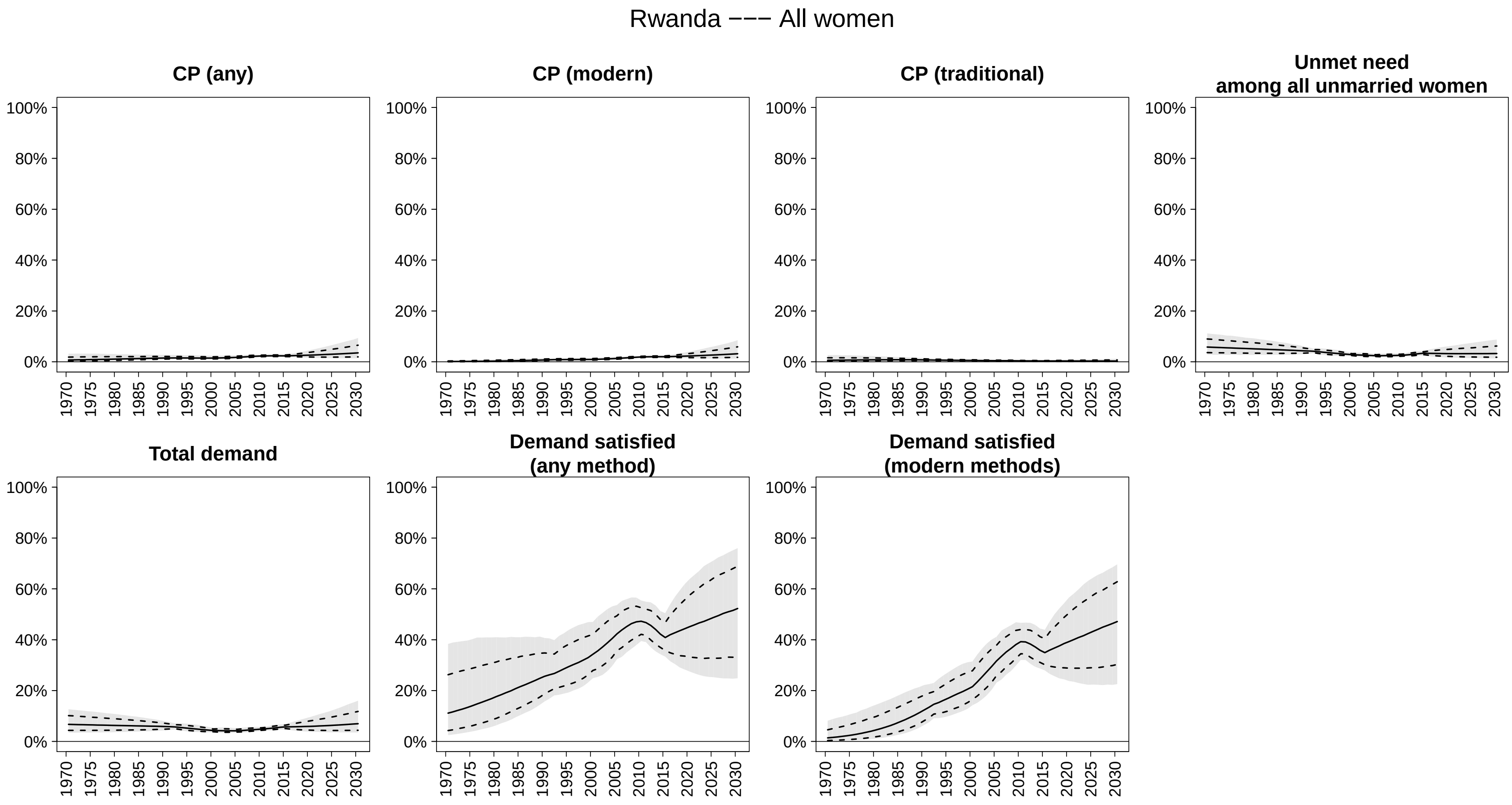
Rwanda −−− All women
CP (any)
CP (modern)
CP (traditional)
Unmet need
among all unmarried women
Total demand
Demand satisfied
(any method)
Demand satisfied
(modern methods)
100%
80%
60%
40%
20%
0%
1970
1975
1980
1985
1990
1995
2000
2005
2010
2015
2020
2025
2030

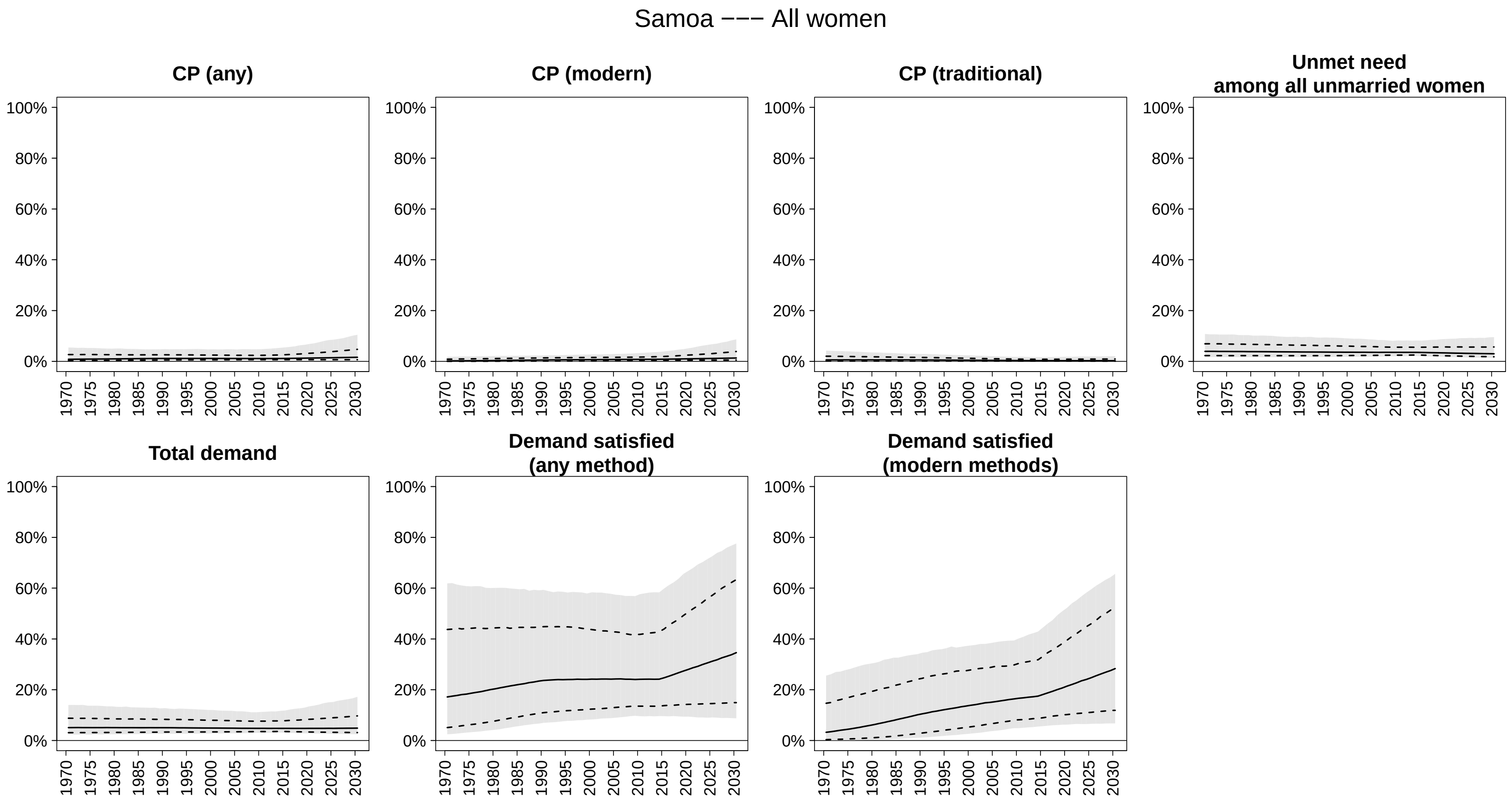
Samoa −−− All women
CP (any)
CP (modern)
CP (traditional)
Unmet need among all unmarried women
Total demand
Demand satisfied (any method)
Demand satisfied (modern methods)
0%
20%
40%
60%
80%
100%
1970
1975
1980
1985
1990
1995
2000
2005
2010
2015
2020
2025
2030

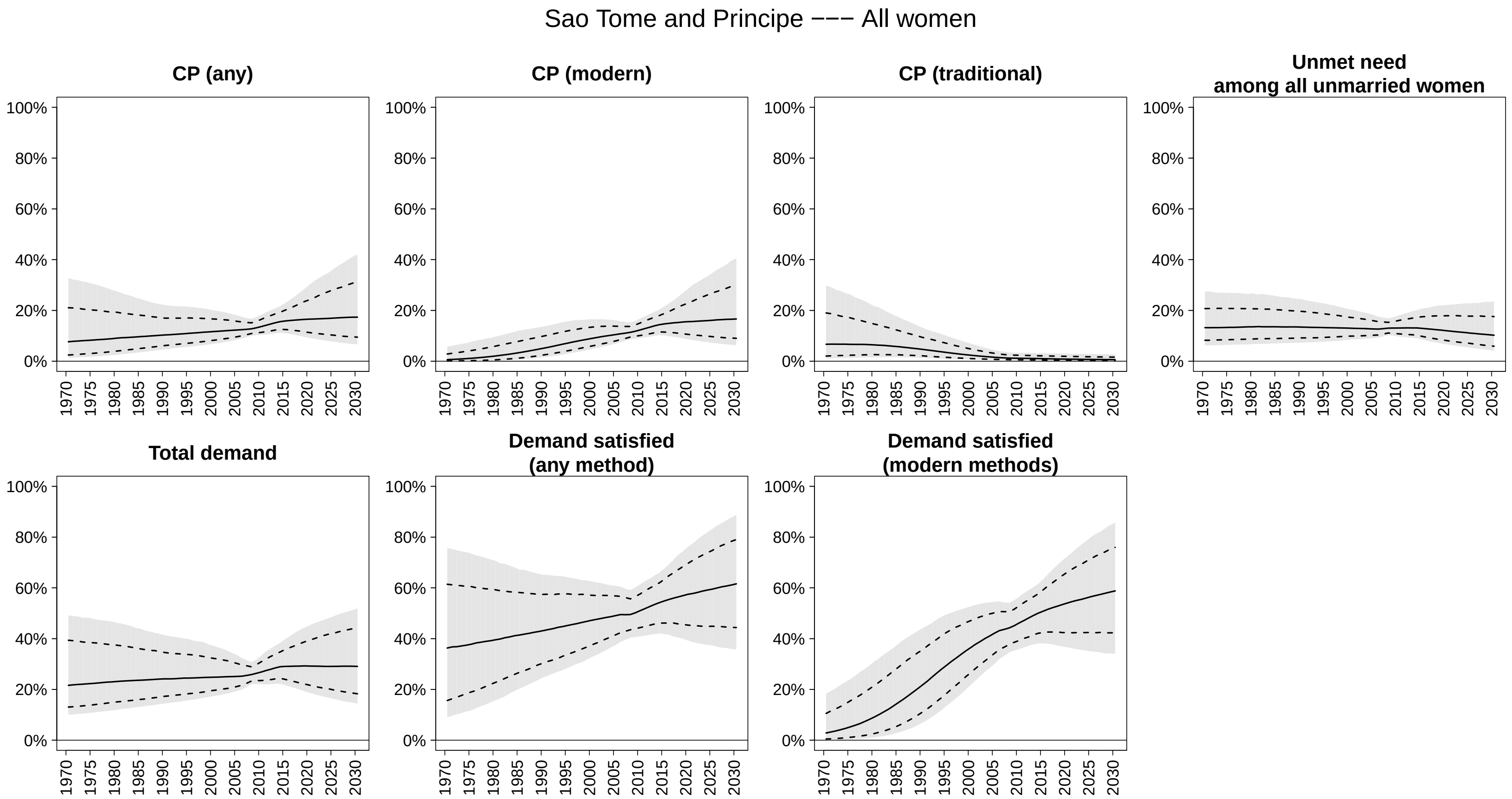
Sao Tome and Principe −−− All women
CP (any)
CP (modern)
CP (traditional)
Unmet need among all unmarried women
Total demand
Demand satisfied (any method)
Demand satisfied (modern methods)
0%
20%
40%
60%
80%
100%
1970
1975
1980
1985
1990
1995
2000
2005
2010
2015
2020
2025
2030

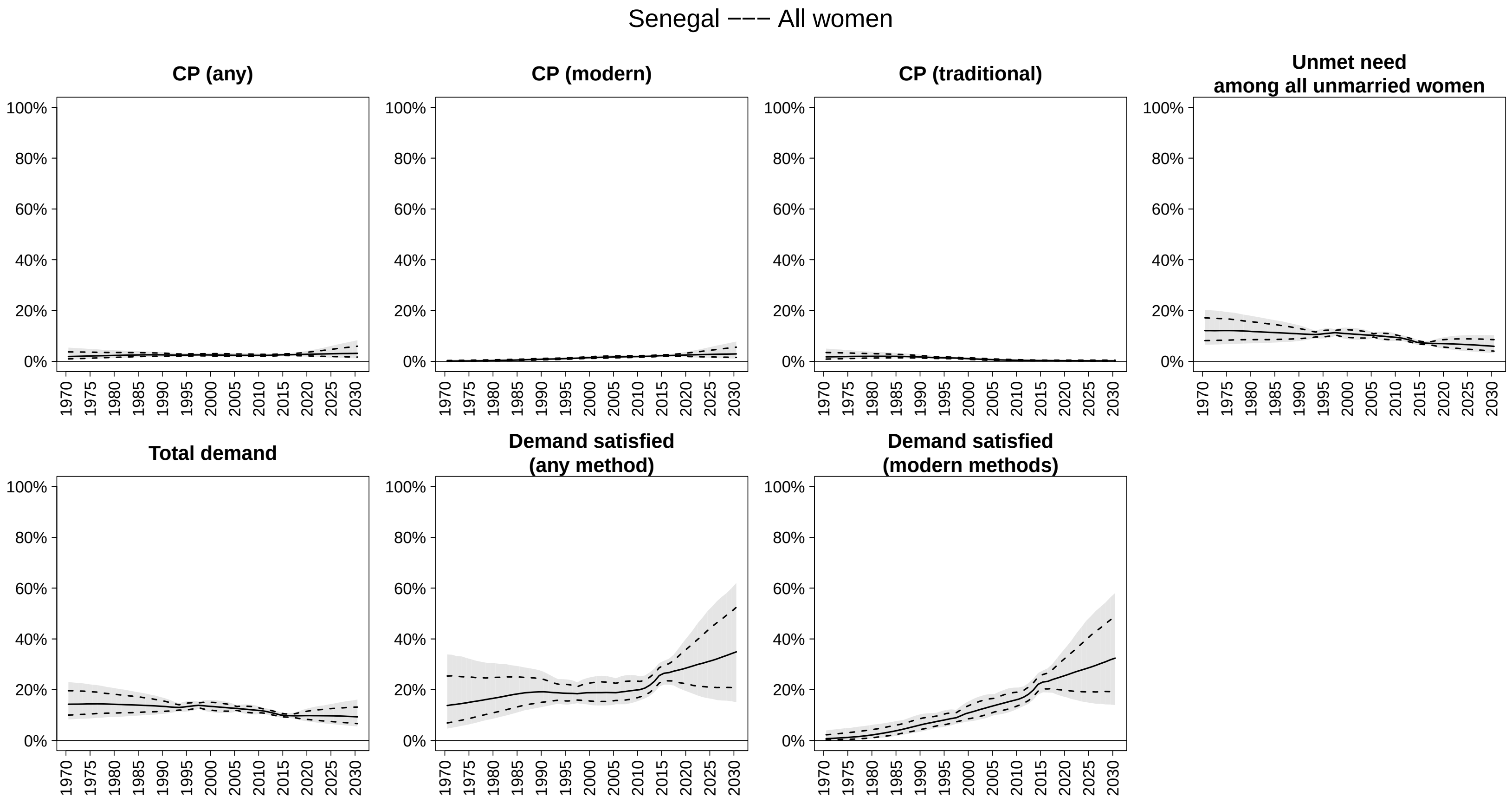
Senegal −−− All women
CP (any)
CP (modern)
CP (traditional)
Unmet need
among all unmarried women
Total demand
Demand satisfied
(any method)
Demand satisfied
(modern methods)
0%
20%
40%
60%
80%
100%
1970
1975
1980
1985
1990
1995
2000
2005
2010
2015
2020
2025
2030

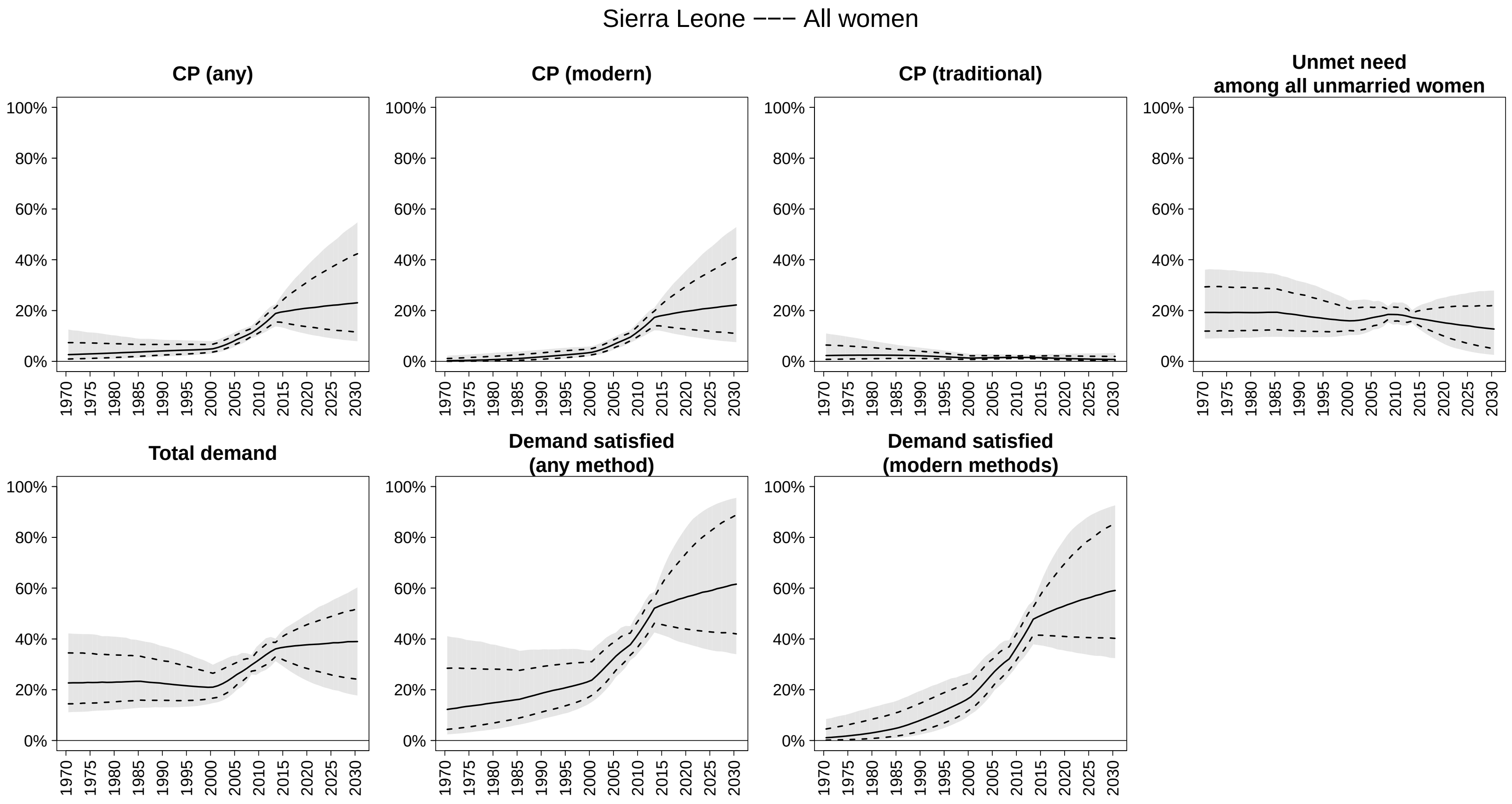
Sierra Leone −−− All women
CP (any)
CP (modern)
CP (traditional)
Unmet need
among all unmarried women
Total demand
Demand satisfied
(any method)
Demand satisfied
(modern methods)
0%
20%
40%
60%
80%
100%
1970
1975
1980
1985
1990
1995
2000
2005
2010
2015
2020
2025
2030

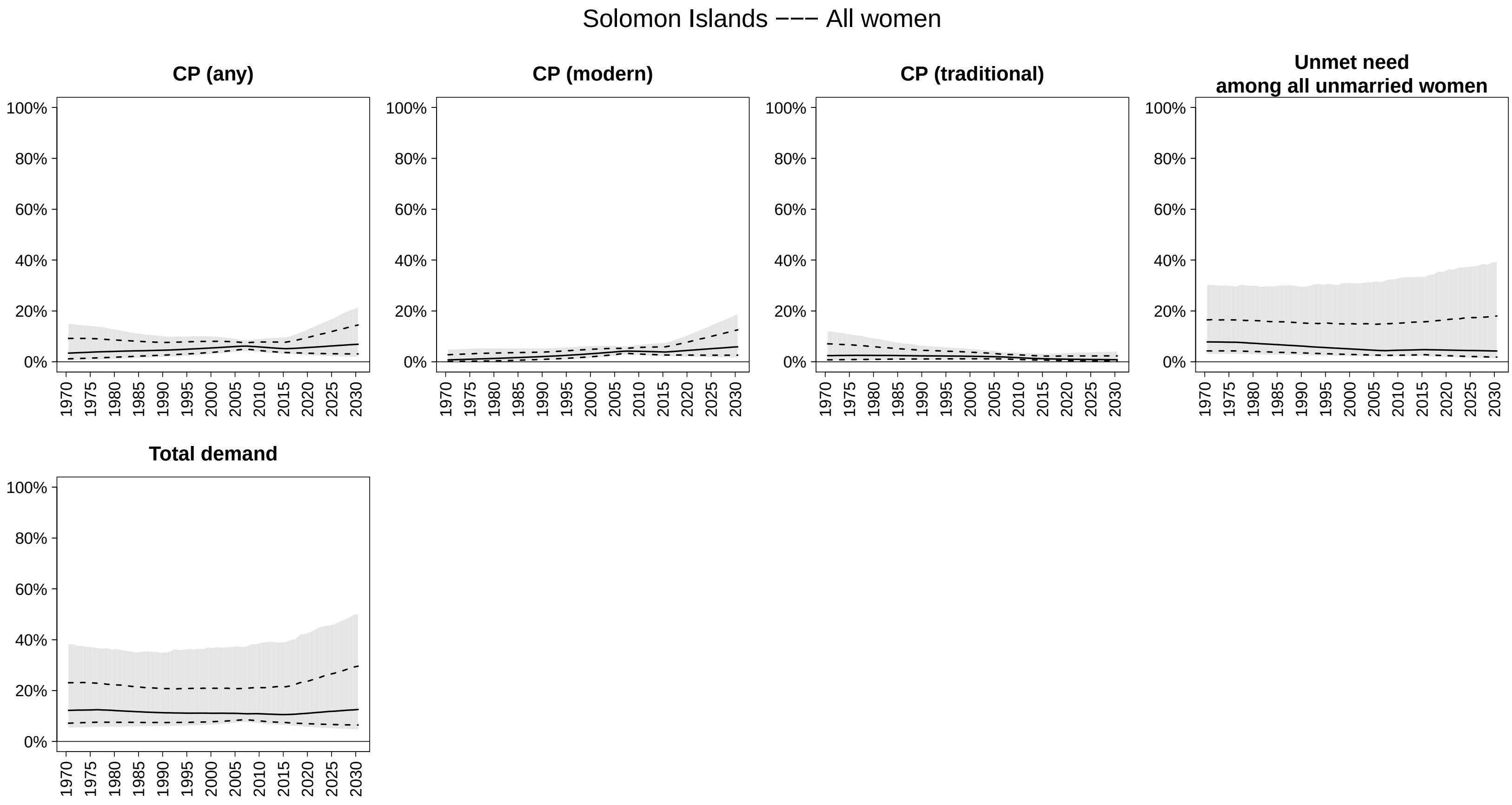
Solomon Islands −−− All women
CP (any)
CP (modern)
CP (traditional)
Unmet need among all unmarried women
Total demand
0%
20%
40%
60%
80%
100%
1970
1975
1980
1985
1990
1995
2000
2005
2010
2015
2020
2025
2030

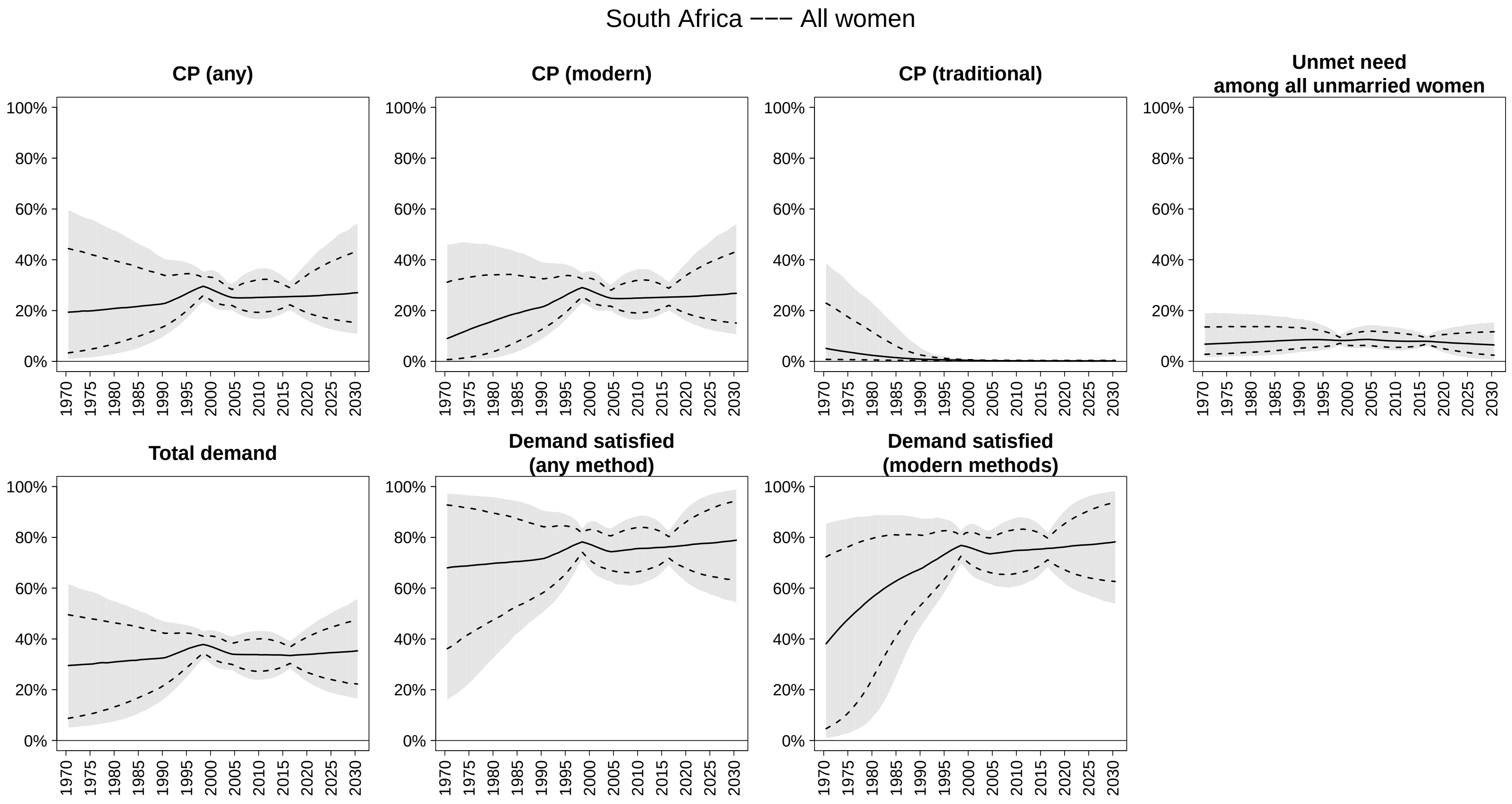
South Africa −−− All women
CP (any)
CP (modern)
CP (traditional)
Unmet need
among all unmarried women
Total demand
Demand satisfied
(any method)
Demand satisfied
(modern methods)
0%
20%
40%
60%
80%
100%
1970
1975
1980
1985
1990
1995
2000
2005
2010
2015
2020
2025
2030

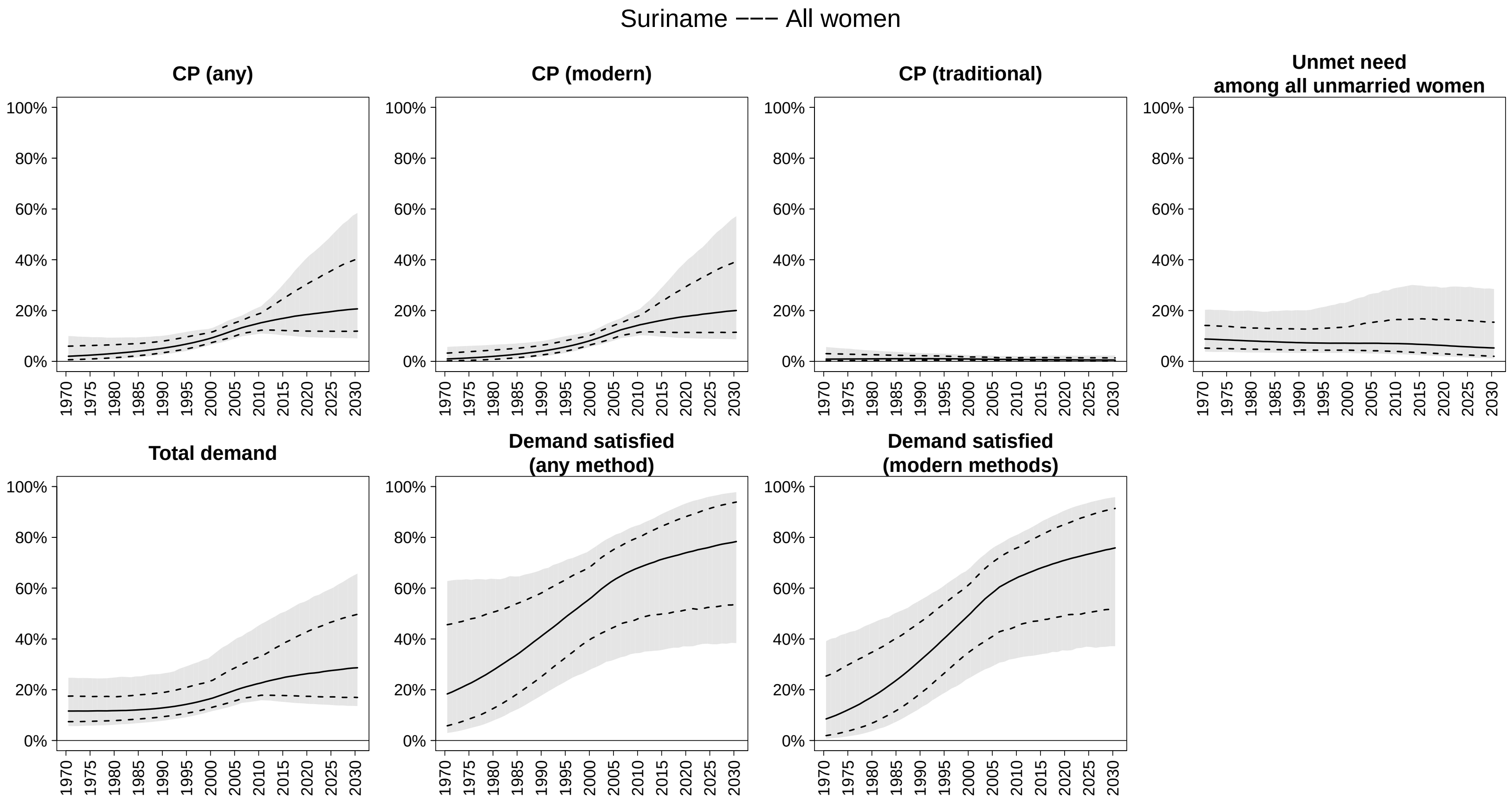
Suriname −−− All women
CP (any)
CP (modern)
CP (traditional)
Unmet need
among all unmarried women
Total demand
Demand satisfied
(any method)
Demand satisfied
(modern methods)
0%
20%
40%
60%
80%
100%
1970
1975
1980
1985
1990
1995
2000
2005
2010
2015
2020
2025
2030

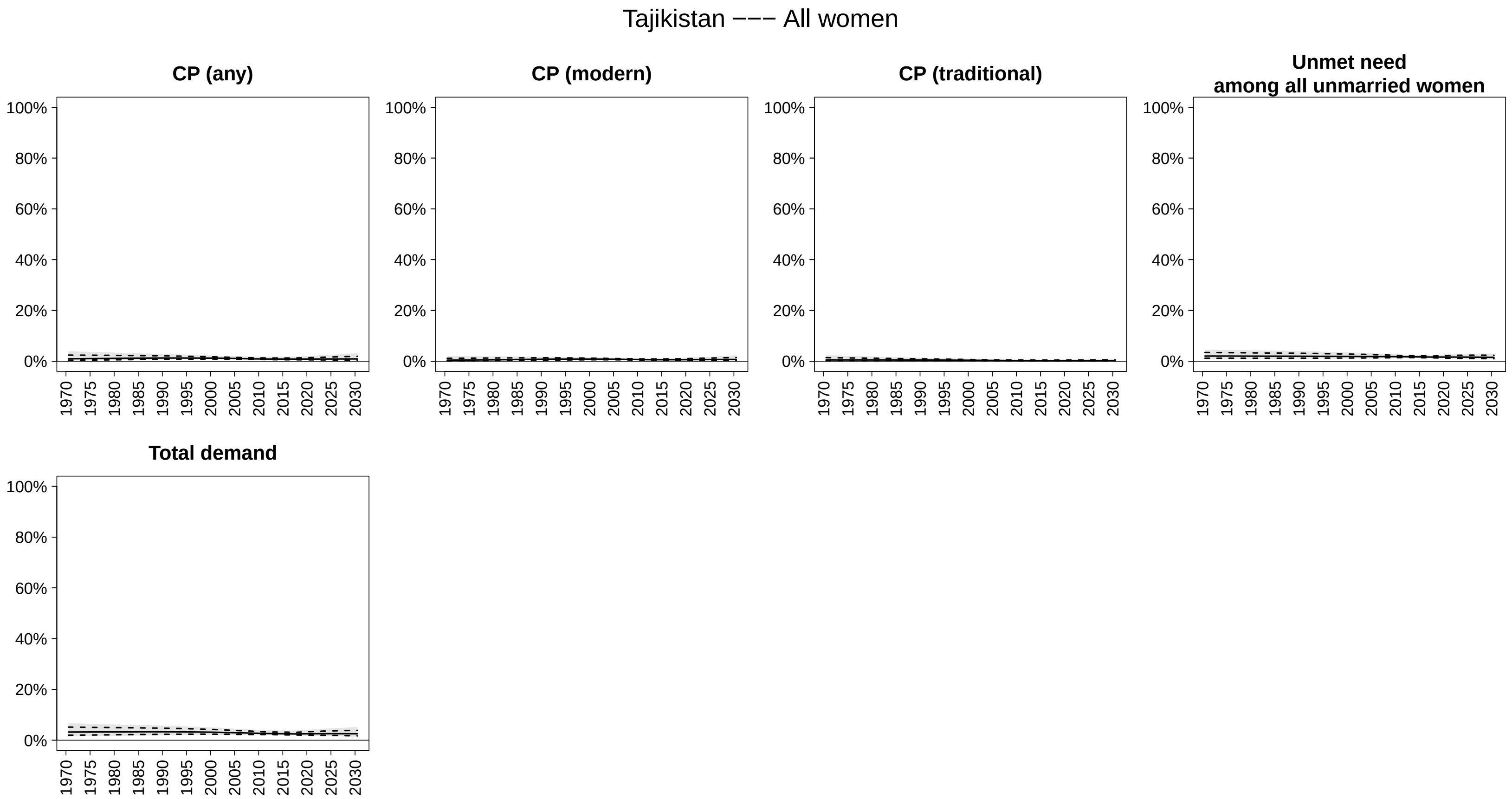
Tajikistan −−− All women
CP (any)
CP (modern)
CP (traditional)
Unmet need
among all unmarried women
Total demand
100%
80%
60%
40%
20%
0%
1970
1975
1980
1985
1990
1995
2000
2005
2010
2015
2020
2025
2030

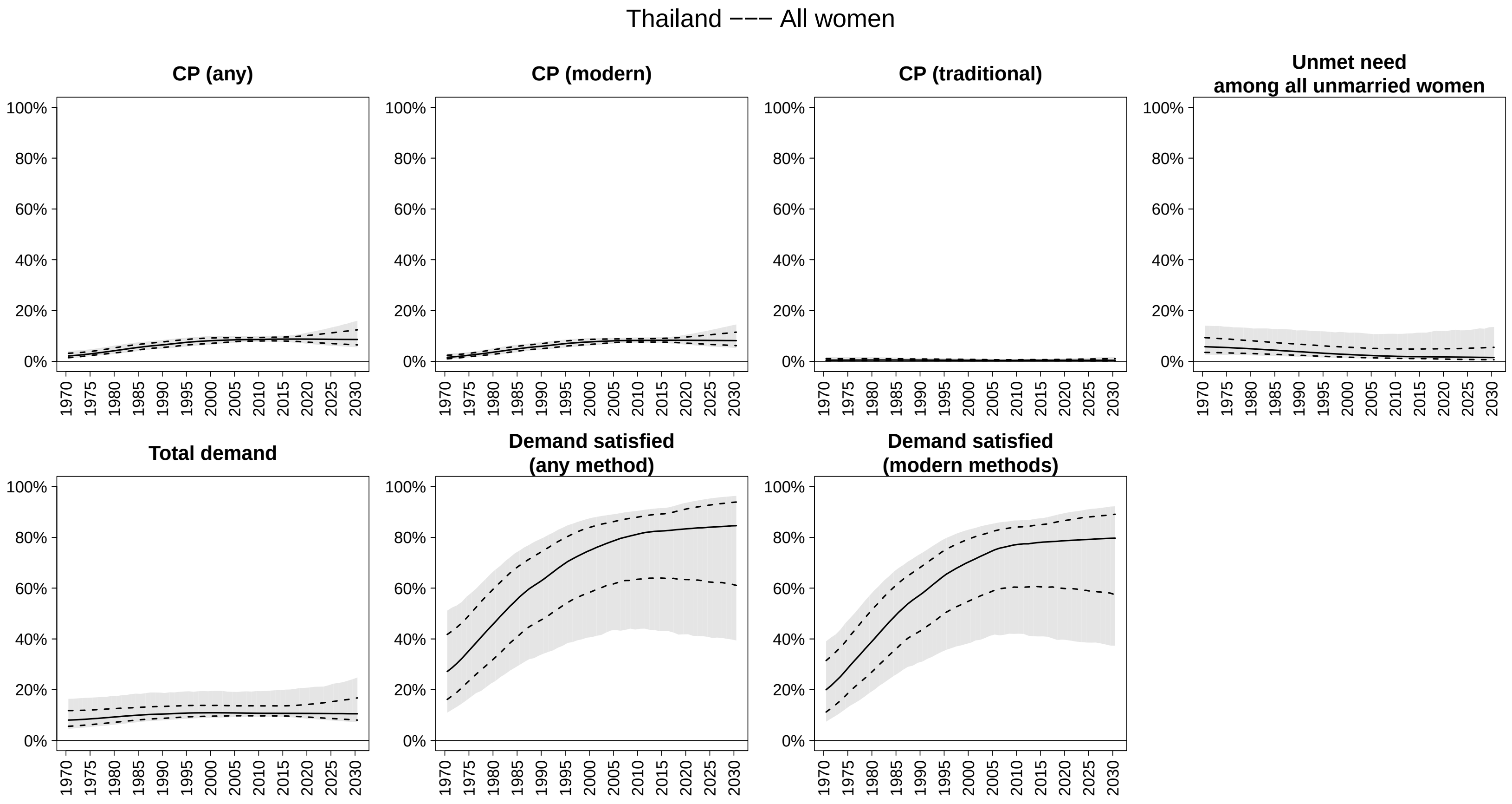
Thailand −−− All women
CP (any)
CP (modern)
CP (traditional)
Unmet need among all unmarried women
Total demand
Demand satisfied (any method)
Demand satisfied (modern methods)
0%
20%
40%
60%
80%
100%
1970
1975
1980
1985
1990
1995
2000
2005
2010
2015
2020
2025
2030

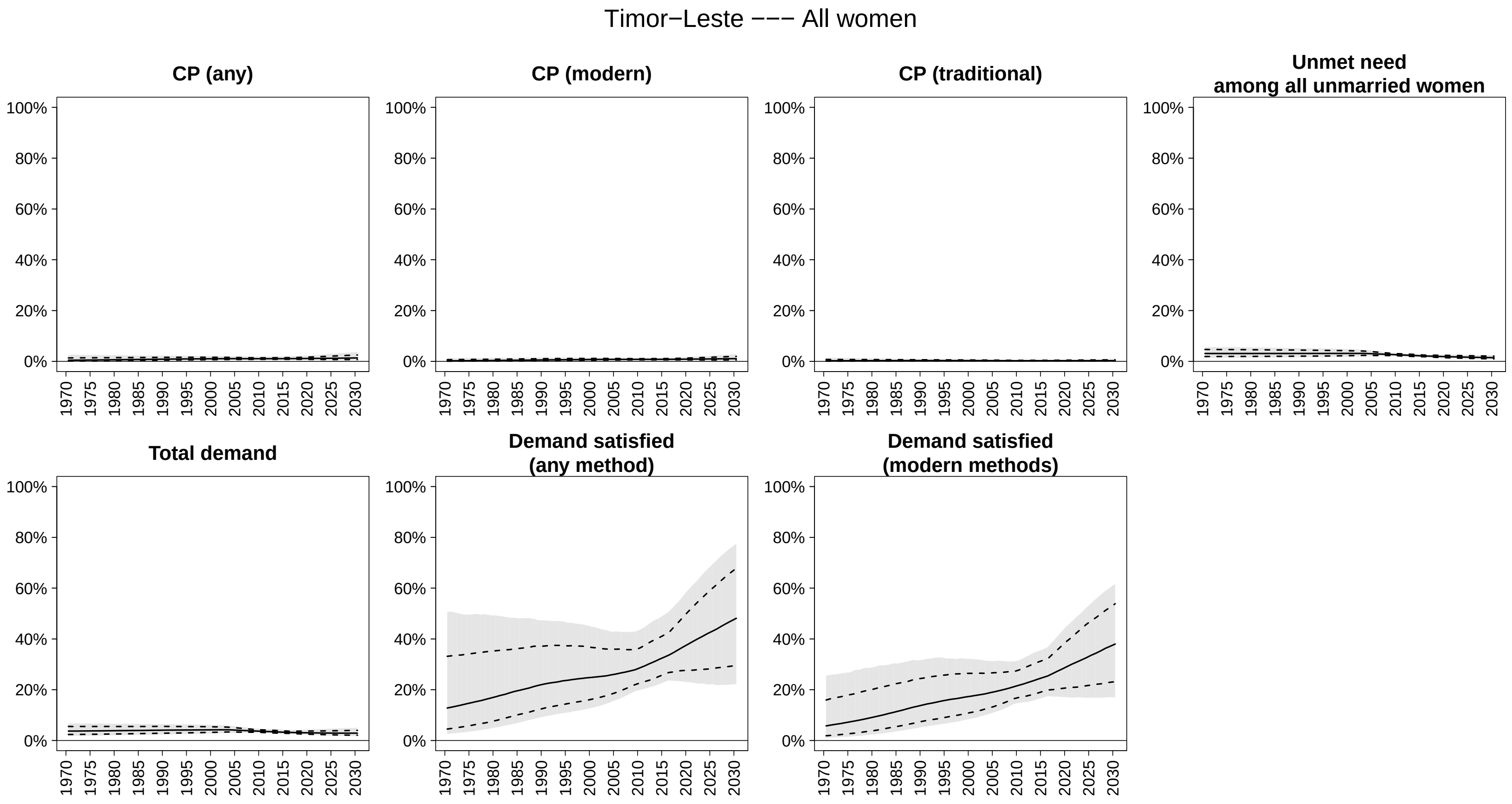
Timor−Leste −−− All women
CP (any)
CP (modern)
CP (traditional)
Unmet need among all unmarried women
Total demand
Demand satisfied (any method)
Demand satisfied (modern methods)
0%
20%
40%
60%
80%
100%
1970
1975
1980
1985
1990
1995
2000
2005
2010
2015
2020
2025
2030

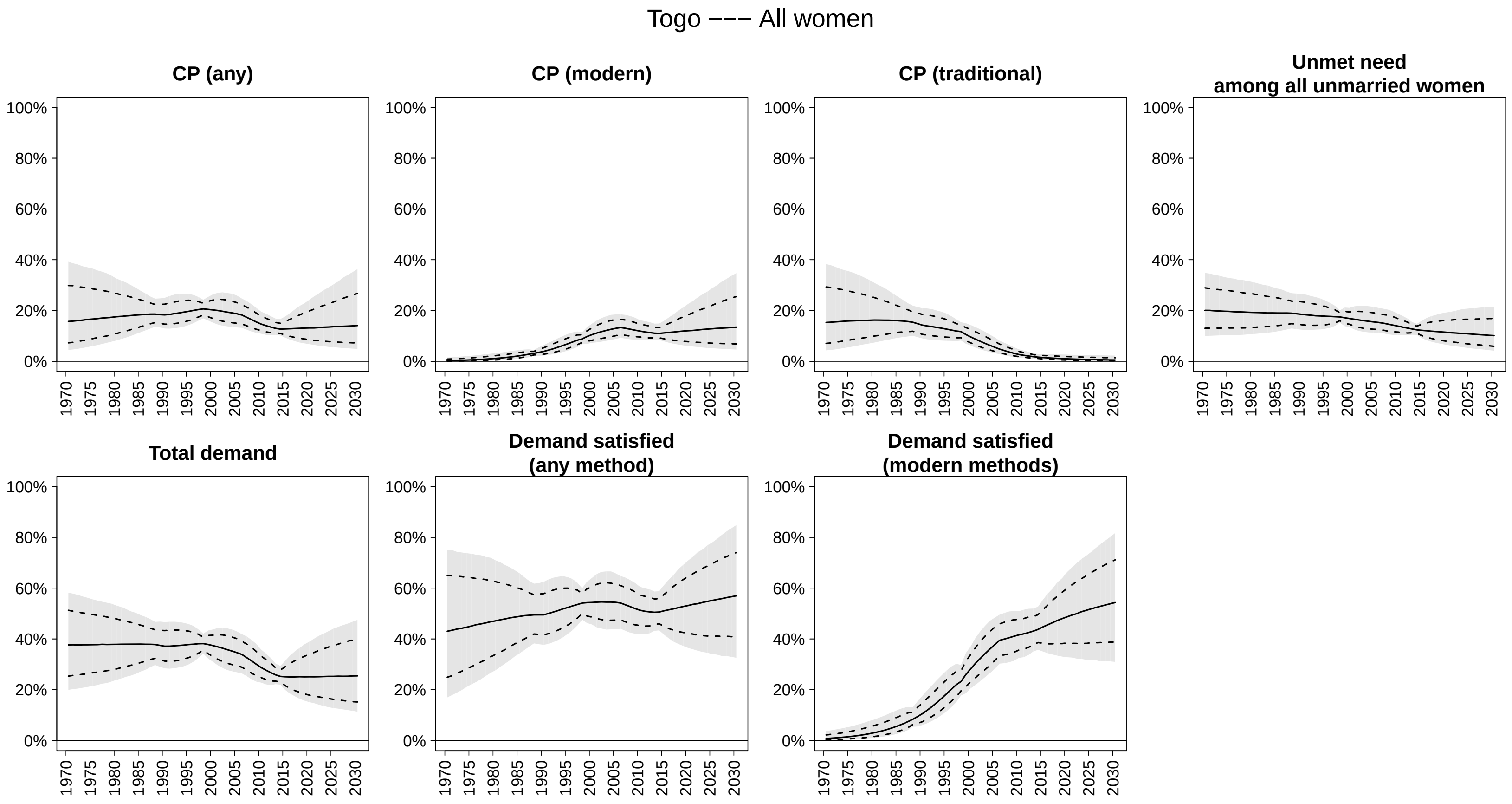
Togo −−− All women
CP (any)
CP (modern)
CP (traditional)
Unmet need
among all unmarried women
Total demand
Demand satisfied
(any method)
Demand satisfied
(modern methods)
0%
20%
40%
60%
80%
100%
1970
1975
1980
1985
1990
1995
2000
2005
2010
2015
2020
2025
2030

Trinidad and Tobago −−− All women

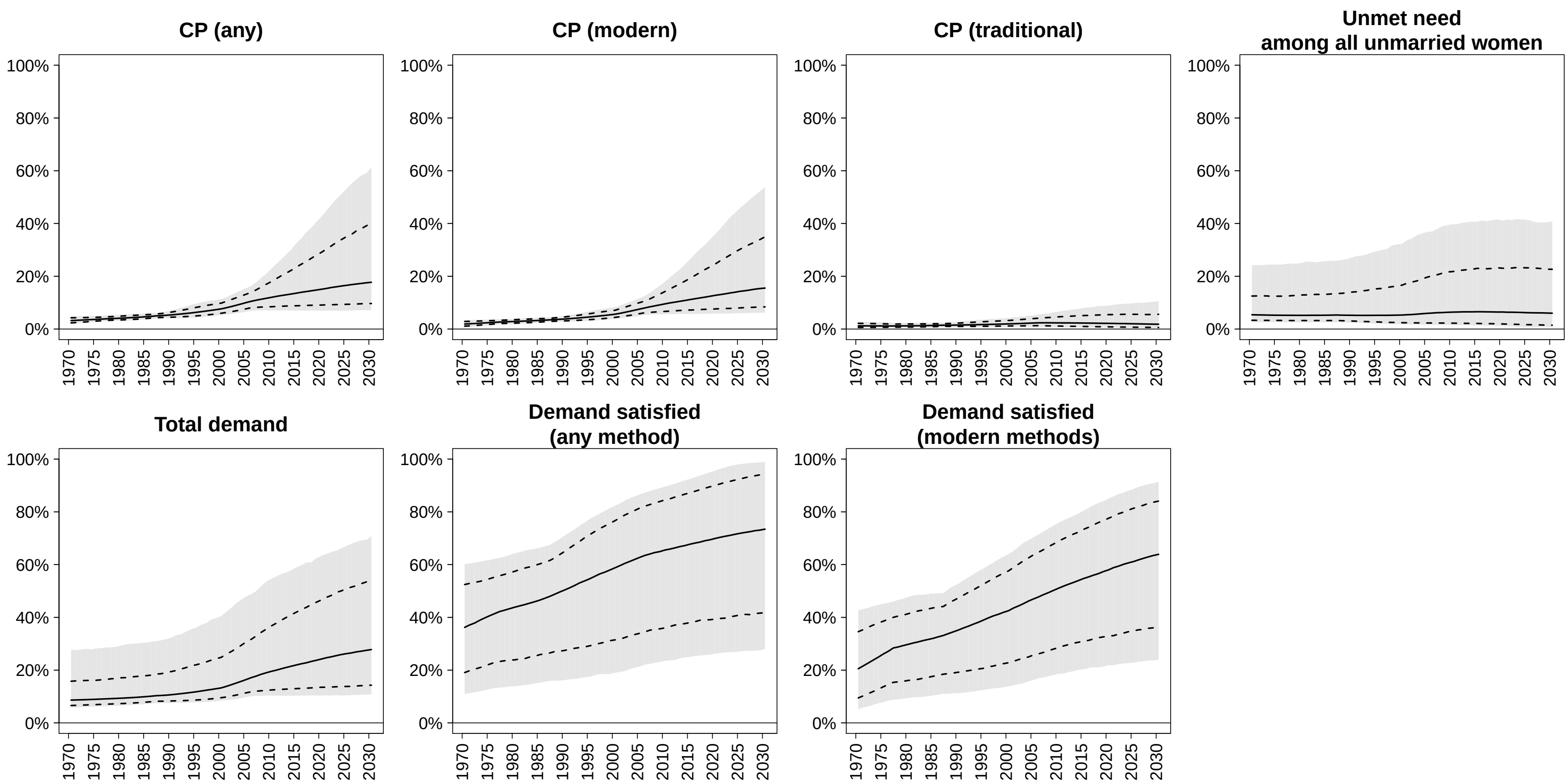

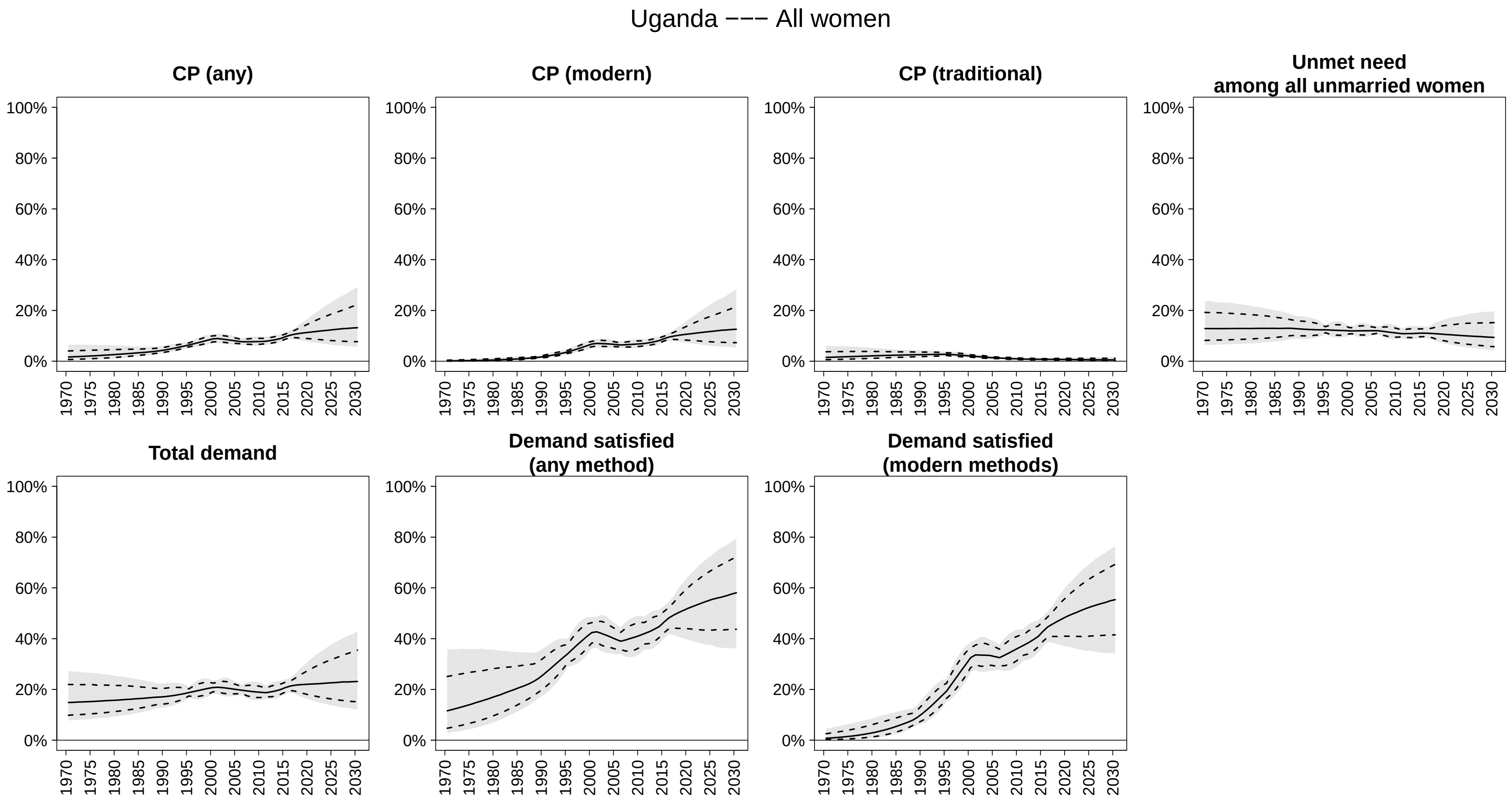
Uganda −−− All women
CP (any)
CP (modern)
CP (traditional)
Unmet need
among all unmarried women
Total demand
Demand satisfied
(any method)
Demand satisfied
(modern methods)
100%
80%
60%
40%
20%
0%
1970
1975
1980
1985
1990
1995
2000
2005
2010
2015
2020
2025
2030

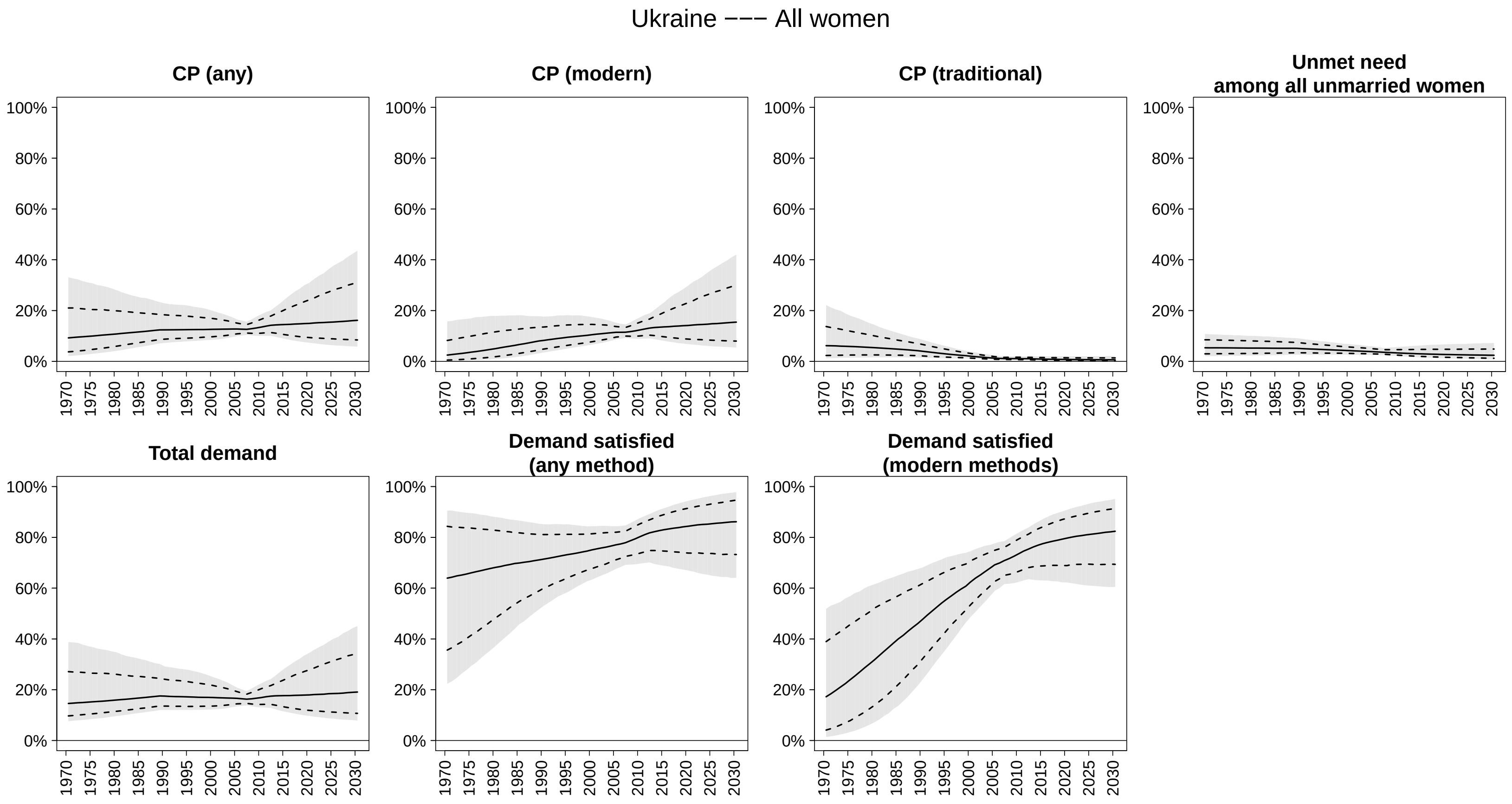
Ukraine −−− All women
CP (any)
CP (modern)
CP (traditional)
Unmet need
among all unmarried women
Total demand
Demand satisfied
(any method)
Demand satisfied
(modern methods)
0%
20%
40%
60%
80%
100%
1970
1975
1980
1985
1990
1995
2000
2005
2010
2015
2020
2025
2030

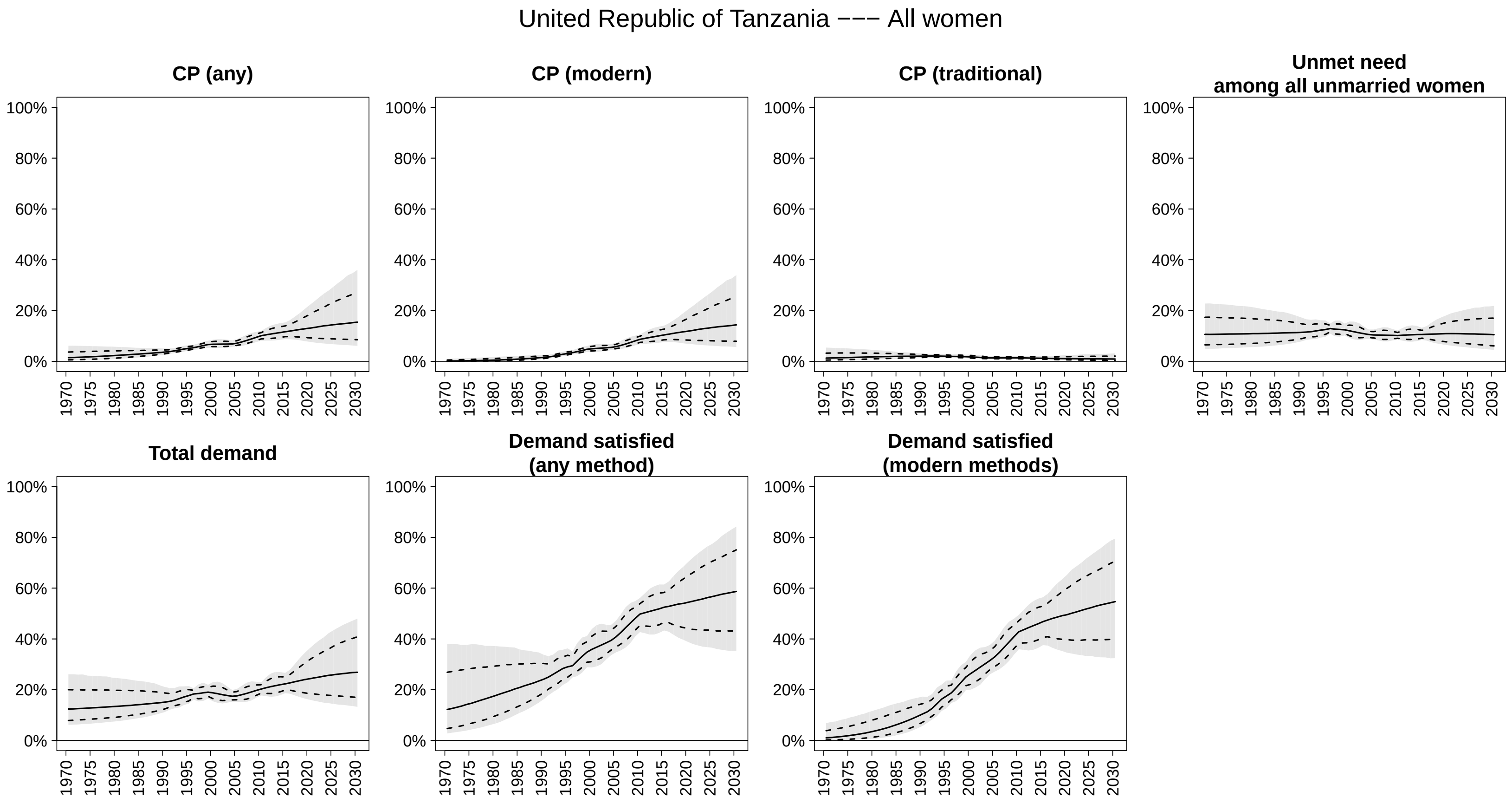
United Republic of Tanzania −−− All women
CP (any)
CP (modern)
CP (traditional)
Unmet need among all unmarried women
Total demand
Demand satisfied (any method)
Demand satisfied (modern methods)
0%
20%
40%
60%
80%
100%
1970
1975
1980
1985
1990
1995
2000
2005
2010
2015
2020
2025
2030

United States of America −−− All women

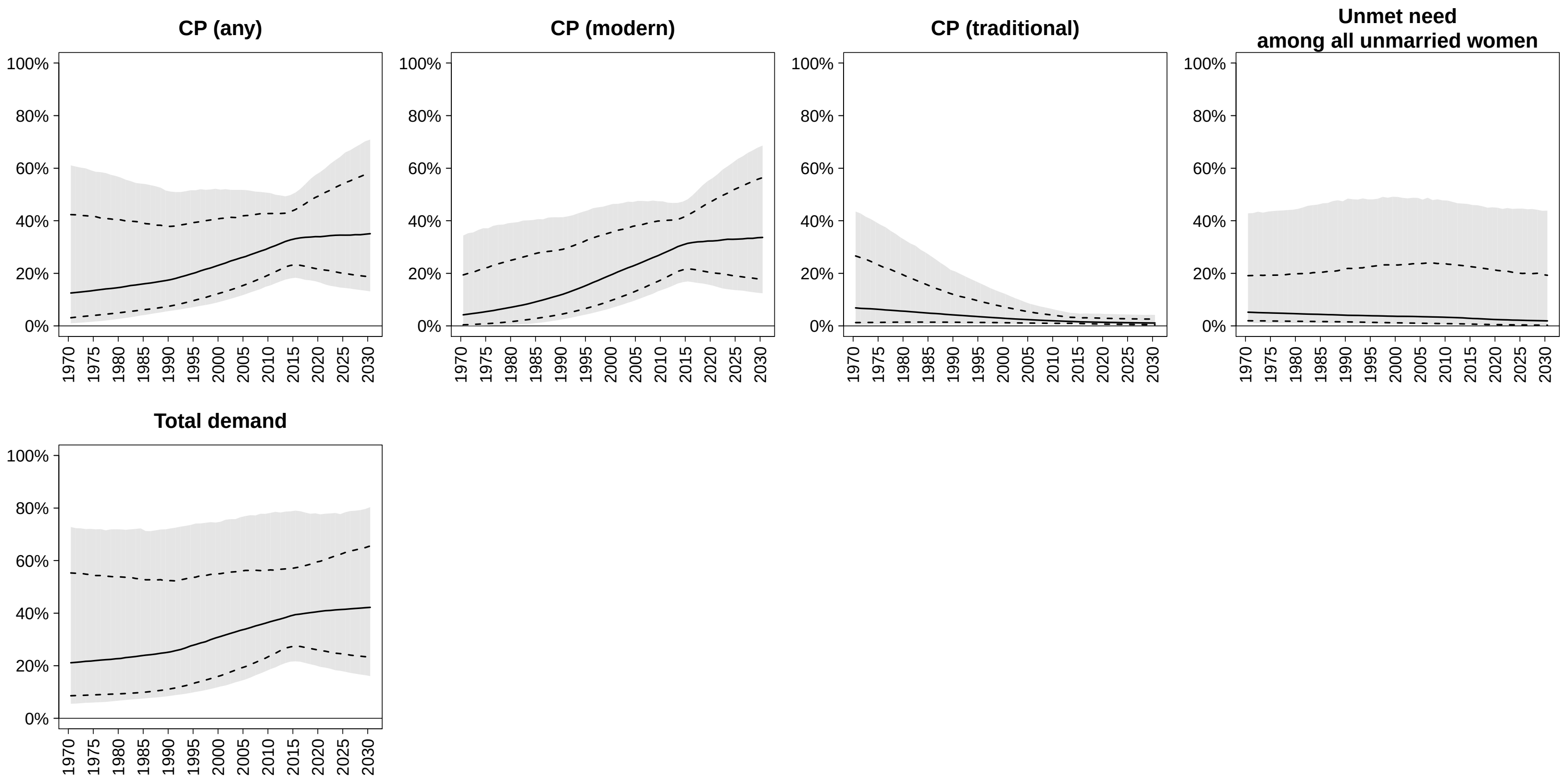

Uzbekistan −−− All women

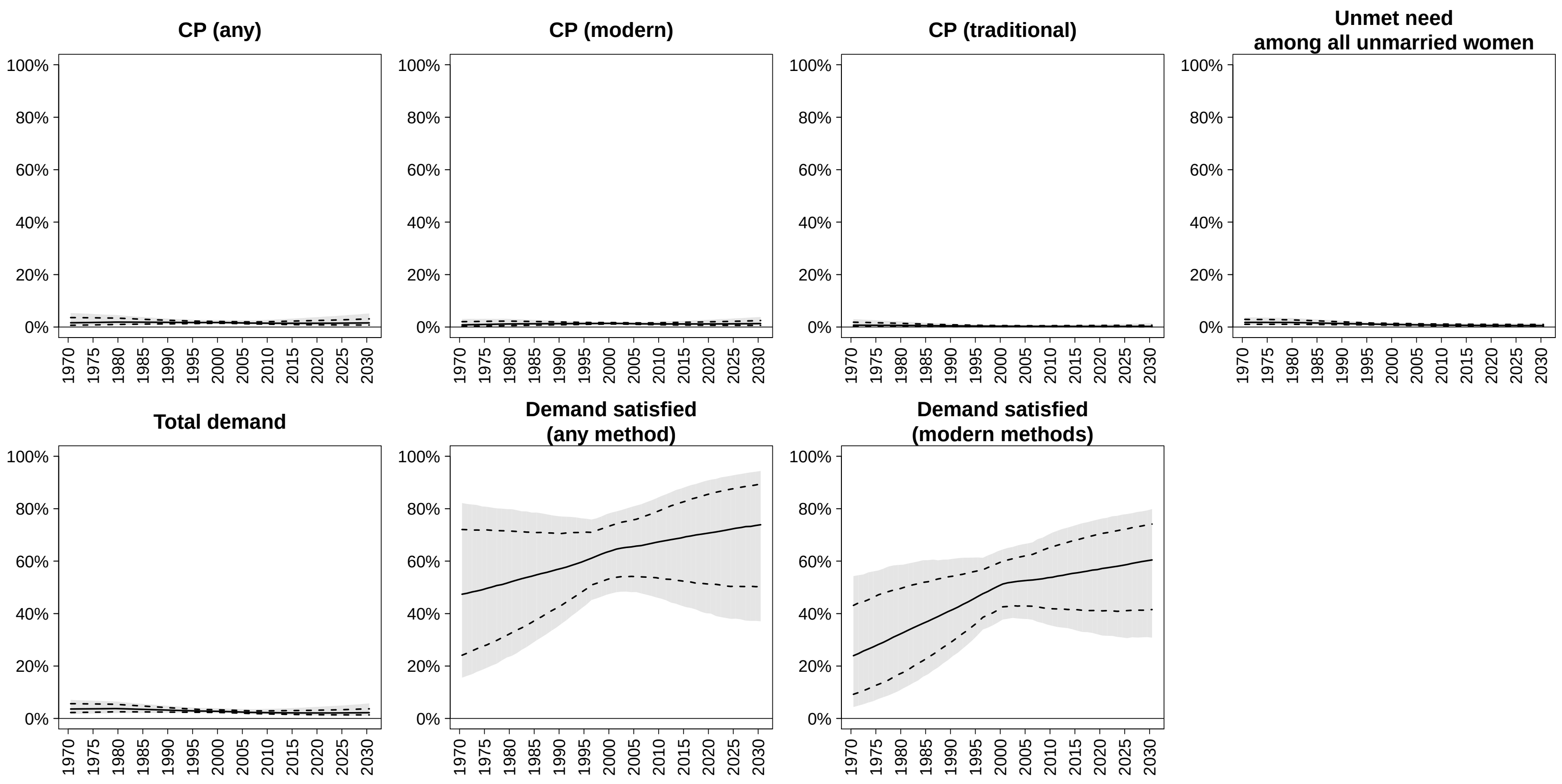

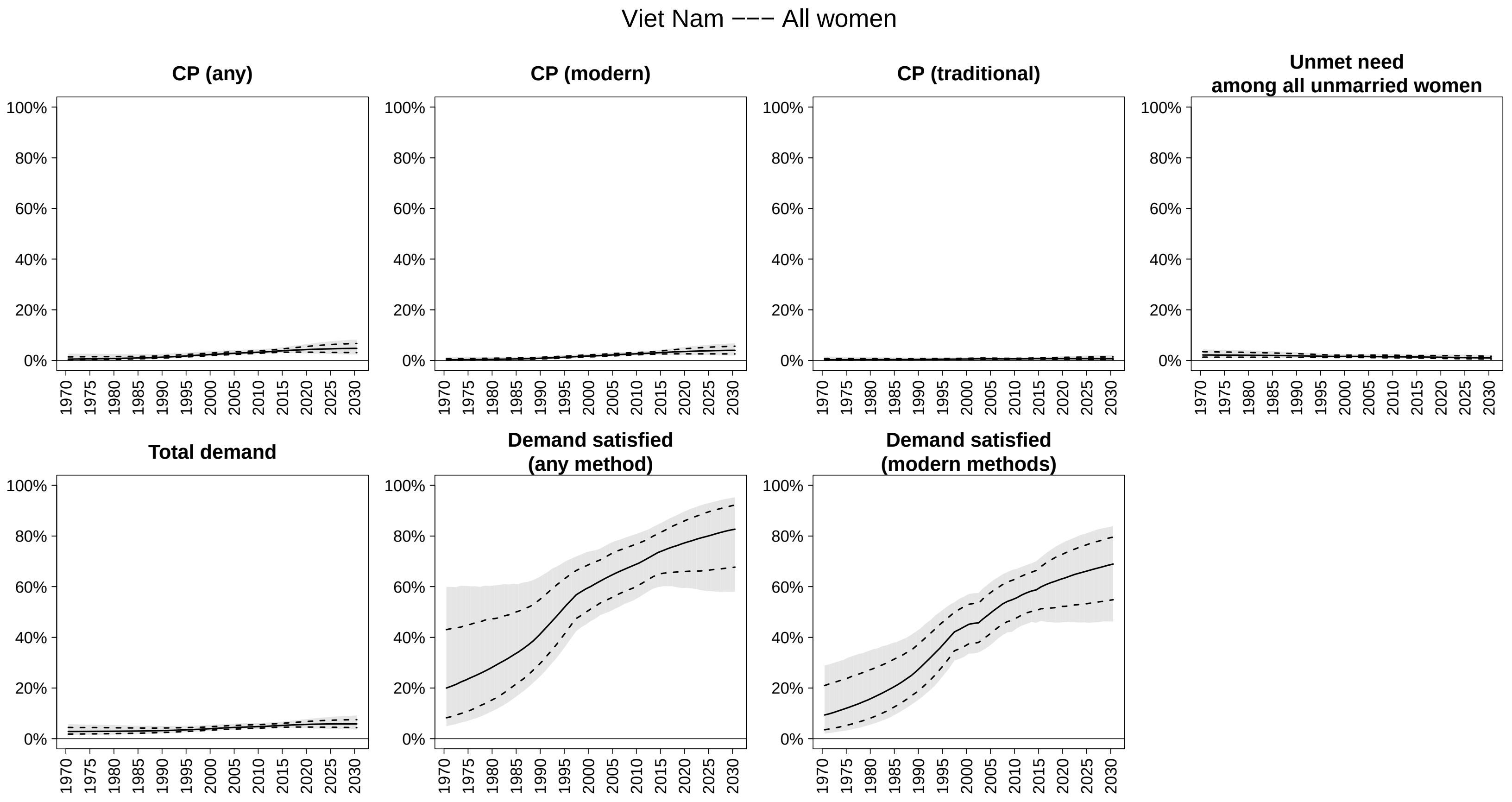
Viet Nam −−− All women
CP (any)
CP (modern)
CP (traditional)
Unmet need among all unmarried women
Total demand
Demand satisfied (any method)
Demand satisfied (modern methods)
0%
20%
40%
60%
80%
100%
1970
1975
1980
1985
1990
1995
2000
2005
2010
2015
2020
2025
2030

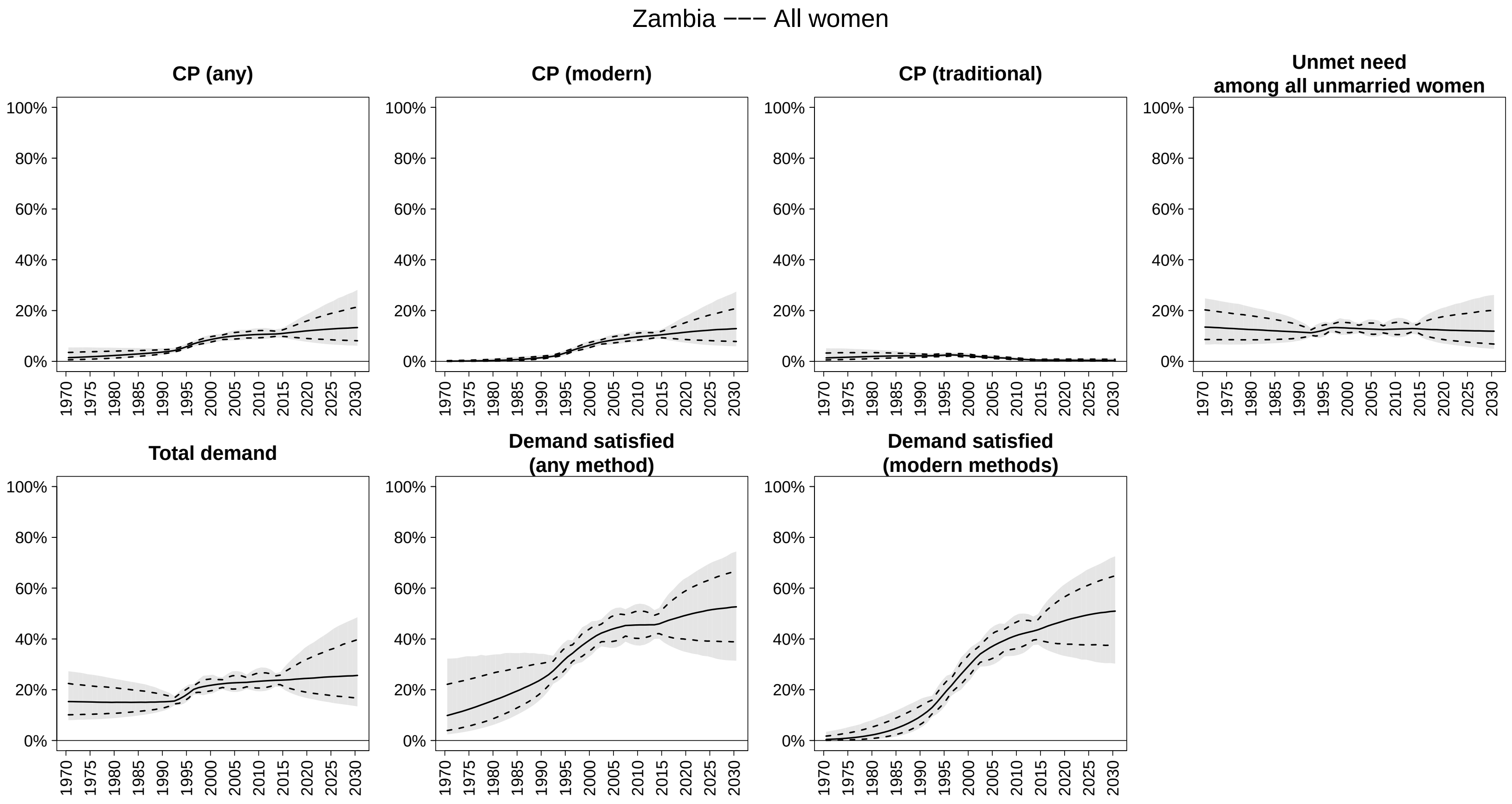
Zambia −−− All women
CP (any)
CP (modern)
CP (traditional)
Unmet need among all unmarried women
Total demand
Demand satisfied (any method)
Demand satisfied (modern methods)
0%
20%
40%
60%
80%
100%
1970
1975
1980
1985
1990
1995
2000
2005
2010
2015
2020
2025
2030

Zimbabwe −−− All women

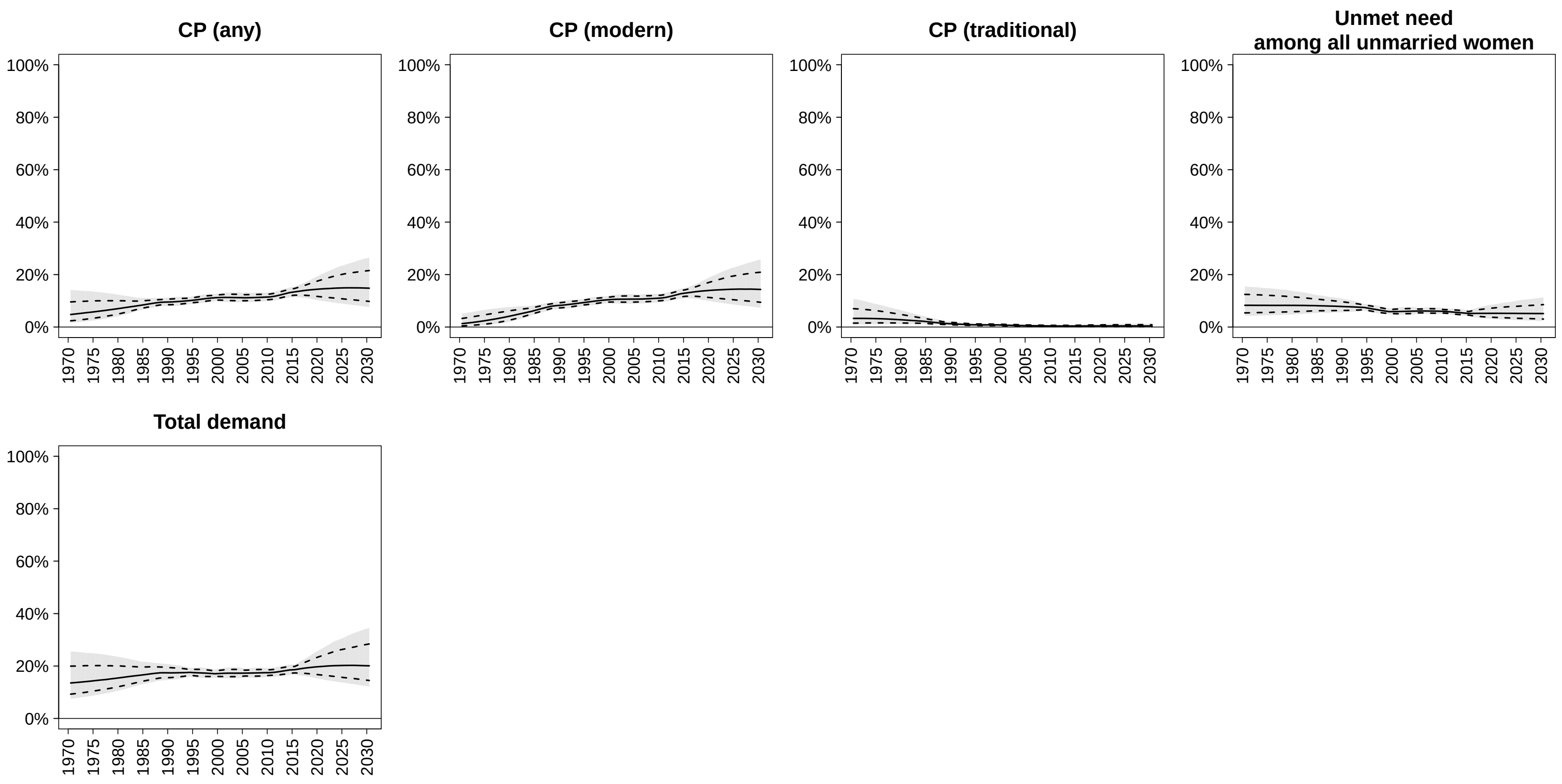

References

Pawlowsky-Glahn, V., J. J. Egozcue and R. Tolosana-Delgado (2015). *Modeling and Analysis of Compositional Data: Modeling and Analysis of Compositional Data*. New York, NY: John Wiley & Sons, Incorporated.
