# Supplementary material for: Contraceptive use and needs among adolescent women aged 15–19: Regional and global estimates and projections from 1990 to 2030 from a Bayesian hierarchical modelling study
Source: PLoS One. 2021 Mar 4;16(3):e0247479. doi: 10.1371/journal.pone.0247479 (PMC7932081; doi:10.1371/journal.pone.0247479)
Supplement: Attachment — Submitted filename: renamed_8cd25.pdf [file pone.0247479.s012.pdf]

Review of PLoS manuscript "Contraceptive use and needs among adolescent women aged 15-19: regional and global estimates and projections from 1990 to 2030 from a Bayesian hierarchical modeling study"

I think this study could constitute a valuable and needed contribution to the literature. The authors' approach allows for comparison of trends over time; is global in scope; represents a massive compilation of data; and they estimated uncertainty intervals which can help readers interpret and understand differences between regions/countries and over time.

I also think this study could be strengthened in several areas.

Methods

The section on statistical methods begins,

> *A Bayesian hierarchical model was used to estimate and project contraceptive prevalence and the unmet need for family planning among women aged 15-19 years. The estimates for women who are married or in a union were derived separately from those for women who are not married or in a union, using methods developed for estimating and projecting family planning indicators among all women of reproductive age (15-49 years) [19-21].*

The paragraph cites Alkema et al (2013), the Lancet paper which describes how contraceptive needs and use were estimated for married women, to a review of survey data, and Wheldon et al (2018), a UNPD technical paper which describes how that model was revised for unmarried women. I assume from this, and also because the manuscript doesn't include technical details on the modeling approach, that these citations were to convey that the authors applied the same methodology but to a subset of those data. However, this should be clarified. I.e., please specify whether the methods were exactly the same except for the data restrictions (or if not, please specify what the differences were).

I think it's probably fine to apply this model. However, the authors have not presented out-of-sample validation exercises. These are needed to assess how well the model performs for years lacking reliable data and for countries for which data are unavailable, and to assess the forward-projections.

The authors define *sexually active* as having had intercourse within the past four weeks. The authors state that they used four-week intervals because those are "comparable to those generally published in the survey reports." However, this definition strikes me as closer to *having recently had sex* than *sexually active*. In contrast, the *Adding It Up* study defines *sexually active* as having had sex within the past three months. The other analysis which the authors cited, Behrman et al (2018), uses as the denominator women who have *ever* had sex. Both of these studies used DHS data. Among the reasons a 28-day window surprised me is that this would result in smaller sample sizes in contrast to a 3-month window, exacerbating the data limitations. Another concern I have is whether this shorter interval is more likely to capture individuals who have sex more frequently. Their contraceptive behaviors could differ, and this could could bias the estimates of demand satisfied.

The authors write that for married women, countries were clustered within subregions, and subregions, in turn, were clustered within regions. In contrast, for unmarried women, subregions were not clustered within regions. Rather, subregions were clustered into a very-low-sex cluster or an everywhere-else cluster. If I follow, then, this would mean that, for married women, the subregional parameters for, e.g., Western Africa, would be centered around an Africa mean, and for Western Europe, centered around a Europe mean, but, for unmarried women, the subregional parameters for Western Africa and Western Europe would be centered around the same mean because they're both grouped in the everywhere-else region.

The authors explain that they clustered countries in this way because, "For unmarried adolescent women, need for family planning is closely related to level of sexual activity and sexual activity among unmarried adolescent women varies considerably between countries."

However, sexual activity -- i.e. whether a women is in the "not in need" group or not -- is an unknown that they're modeling, and it's in their data.

Reviewing the technical paper that they cited, Figure 7 on page 11 of Wheldon et al appears to show that "very low" sexual activity roughly corresponds to the Middle East and North Africa, and to LMIC Asia minus China. In several of those countries, my understanding is that data for unmarried women's sexual activity are often unavailable, particularly in earlier periods.

That the DHS doesn't ask these questions in several countries could reflect greater stigma about sexual activity. This could predict lower levels of sexual activity, and also differences in access to and use of contraception among those who *are* sexually active. Thus, given the non-random availability of data for unmarried women, I can see how exchanging information across countries using a geographic regional schema could result in biased estimates.

However, as written, the authors' description of the clustering schema for unmarried women does not address these points, and it could come across as grouping countries based on the values of one of the dependent variables that they are estimating. For these reasons, I think the manuscript would be improved were these issues explained in the manuscript, in contrast to how the clustering schema is described in the text as presently written.

It's possible than an underlying issue is that the UNPD regional groupings weren't designed with modeling in mind. I suspect that using GBD regions and super-regions would make more sense for the authors' models. This could make it possible for the model hierarchy to be the same for married women and for unmarried women.

Related to this, it would be very valuable to perform sensitivity analyses around aspects of the model which are subjective. At a minimum, it would be informative to compare model validation exercises using alternate clustering schemas (e.g., subregions grouped within regions; subregions grouped by sexual activity; GBD regions and super-regions), and to what extent are the estimates of contraceptive needs and use are affected by alternate sexual activity windows.

Results

The "need for family planning" indicates the proportion of adolescent women who are sexually active and do not want to have a(nother) child. Among these, it is further informative to know the demand satisfied -- i.e., the percent of those "in need" who are using a (modern) method of contraception.

However, the results section begins with contraceptive prevalence. Since levels of sexual activity differ across countries and change over time, it's not clear to me how to interpret this.

The results section then discusses "unmet need". However, a country could have higher levels of unmet need because more people are having sex, while at the same time a smaller proportion *of those having sex* (who do not want to have a(nother) child) could have an unmet need for contraception. For these reasons, I think the authors need to do more to explain how this indicator should be interpreted. I can imagine that its utility could be that while it obscures sexual activity and demand satisfied, it predicts population-level adolescents unintended pregnancy among.

The results section then discusses "need for family planning", and, after that, demand satisfied ("need for family planning satisfied by modern methods").

I think it would make more sense if the results section first discussed "need for family planning," then "need for family planning satisfied by modern methods", and then "unmet need for family planning." This is because order to understand comparisons, I think a reader needs to first understand differences in sexual activity levels. The discussion demand satisfied should also, instead of just describing differences in demand satisfied, contextualize these differences. One way to do this is to describe need; then demand satisfied; and then in a third subsection to explain how these two combine to produce unmet need.

The authors also include ternary maps which blend three hues to jointly illustrate % unmet need + % using contraception + % no need. These maps show results by country. However, the authors do not discuss country results. They only discuss subregional results. The

maps also obscure differences across countries in the uncertainty in the country estimates. The maps are also difficult to read, and it could be more useful if presenting maps to illustrate heat maps which report just one indicator. E.g., a map of % in need + a map of % demand satisfied. However, given that the authors don't actually discuss country results in their manuscript, country maps might not actually be appropriate to include alongside the main text.

Global average levels and trends were described. However, regional results were summarized for 2019 only. I think it would be valuable to also discuss regional trends.
